# Down-Regulation of Insulin Like Growth Factor 1 Involved in Alzheimer's Disease via MAPK, Ras, and FoxO Signaling Pathways

**DOI:** 10.1155/2022/8169981

**Published:** 2022-05-04

**Authors:** Kexin Kang, Jun Bai, Shanshan Zhong, Rongwei Zhang, Xiaoqian Zhang, Ying Xu, Mei Zhao, Chuansheng Zhao, Zhike Zhou

**Affiliations:** ^1^Department of Geriatrics, The First Affiliated Hospital, China Medical University, Shenyang, 110001 Liaoning, China; ^2^Cancer Systems Biology Center, The China-Japan Union Hospital, Jilin University, Changchun, 130033 Jilin, China; ^3^Department of Neurology, The First Affiliated Hospital, China Medical University, Shenyang, 110001 Liaoning, China; ^4^Computational Systems Biology Lab, Department of Biochemistry and Molecular Biology and Institute of Bioinformatics, The University of Georgia, USA; ^5^Department of Cardiology, The Shengjing Affiliated Hospital, China Medical University, Shenyang, 110004 Liaoning, China

## Abstract

The inability to halt or even delay the course of Alzheimer's disease (AD) forces the development of new molecular signatures and therapeutic strategies. Insulin like growth factor 1 (IGF1) is a promising target for AD treatment, yet exact mechanisms of AD ascribed to IGF1 remain elusive. Herein, gene expression profiles of 195 samples were analyzed and 19,245 background genes were generated, among which 4,424 differentially expressed genes (DEGs) were overlapped between AD/control and IGF1-low/high groups. Based on such DEGs, seven co-expression modules were established by weight gene correlation network analysis (WGCNA). The turquoise module had the strongest correlation with AD and IGF1-low expression, the DEGs of which were enriched in GABAergic synapse, long-term potentiation, mitogen-activated protein kinase (MAPK), Ras, and forkhead box O (FoxO) signaling pathways. Furthermore, cross-talking pathways of IGF1, including MAPK, Ras, and FoxO signaling pathways were identified in the protein-protein interaction network. According to the area under the curve (AUC) analysis, down-regulation of IGF1 exhibited good diagnostic performance in AD prediction. Collectively, our findings highlight the involvement of low IGF1 in AD pathogenesis via MAPK, Ras, and FoxO signaling pathways, which might advance strategies for the prevention and therapy of AD based on IGF1 target.

## 1. Introduction

Alzheimer's disease (AD), an age-related and progressive neurodegenerative disorder in the elderly, is characterized by extracellular deposits of amyloid-beta (A*β*) peptides and intracellular accumulation of hyperphosphorylated tau proteins [1]. In addition to these aggregates, it shares additional pathological alterations with diabetes mellitus, including abnormal glucose metabolism, insulin resistance, oxidative stress, inflammation, and neuron atrophy [2–4]. Such common hallmarks possibly represent the identical etiology for both clinical entities, a viewpoint supported by an extensive array of epidemiological observations that patients with diabetes mellitus remarkably and independently increase the risk of AD onset [5–7]. Among multiple metabolic hormones in diabetes mellitus, insulin like growth factor 1 (IGF1) is found to be closely related to A*β* clearance and tau phosphorylation, suggesting IGF1 as an important mediator with a wide spectrum of effects on the pathophysiology of diabetes mellitus and AD [7, 8].

IGF1, similar in function and structure to insulin, encodes a regulatory protein involving a series of intracellular biological actions, ranging from DNA synthesis to cell metabolism, cycle, and death [9]. It has been shown that systemic IGF1 enhances insulin activity and neuronal excitability, thus to play a neuroprotective role in brain insults with different etiologies and anatomies [10]. In neurodegenerative conditions, particularly AD, subnormal levels of IGF1 are detected in the brain and serum of affected individuals, a large proportion of whom are accompanied with insulin resistance [11, 12]. Indeed, increased accumulation of soluble A*β* rather than overproduction is thought to be responsible for AD senile-plaque pathology, due at least in part to impaired A*β* clearance mediated by low IGF1 levels [13]. One plausible interpretation is that IGF1 binding to tyrosine kinases of insulin receptors and auto-receptors not only facilitates A*β* clearance but also protects cells against apoptosis in a IGF1-binding protein-sensitive manner [14, 15]. Moreover, a substantial amount of evidence [16–18] has confirmed that IGF1 elicits the bond of tau protein with microtubules to reduce tau phosphorylation, pointing to a direct role of IGF1 in neurofibrillary-tangle pathology of AD. Despite the causal relationship between IGF1 and AD, precise mechanisms of IGF1 underlying AD occurrence have not been well defined. There is convincing evidence that bioinformatics analysis is a promising method to identify potential biomarkers of AD (e.g., NPIPA1) and validated them through functional enrichment annotations [19]. Herein, we performed an integrative genomic analysis to investigate the role played by IGF1 during AD development on basis of gene expression data and functional annotations, aiming to elucidate the mechanistic pathways of IGF1 in AD.

## 2. Materials and Methods

### 2.1. Data Collection

Based on our previous study [20], microarray chips of temporal cortex tissues between AD and non-dementia controls were obtained from the Gene Expression Omnibus (GEO) database (https://www.ncbi.nlm.nih.gov/geo/). We selected the GSE132903 dataset as the test set for genomic analysis, while GSE5281 and GSE37264 are served as the training sets (Table 1). Clinical phenotypic information (e.g., age, gender, AD, and IGF1 expression) for each sample were collected in Supplementary Table 1. In *limma* package of R software, the *normalizeBetweenArrays* function was used to normalize ribonucleic acid sequencing (RNA-seq) data from all datasets.

### 2.2. Differential Expression Analysis

Using *lmFit* and *eBayes* functions in R package *limma*, RNA-seq data were processed to screen for differentially expressed genes (DEGs) between AD and non-dementia controls [21]. Based on DEGs values, the samples were grouped by two-dimensional hierarchical clustering method, and the results were displayed in the forms of volcano plot and heatmap. The threshold value for DEGs identification was |logFC (fold change)|>0.15 with a false discovery rate (FDR)-adjusted *p* value of <0.05.

### 2.3. Co-Expression Network Analysis

The co-expression network on DEGs overlapped from AD/control and IGF1-low/high groups was established by weight gene correlation network analysis (WGCNA). The procedure for co-expression network construction using WGCNA package consisted of the following steps: (1) remove outliers from sample clustering diagram; (2) select the soft threshold power of 8 to construct an adjacency matrix; (3) transform the adjacency matrix into a topological overlap matrix (TOM); (4) form a hierarchical clustering tree by hierarchical clustering on basis of the TOM dissimilarity; (5) obtain the modules from the branches of the hierarchical clustering tree with the minModuleSize =30; and (6) cluster the modules to build a co-expression network by the average-linkage hierarchical clustering method according to the value of module eigengene (ME), an index indicating the overall expression of a module [22]. Gene functional annotation on Kyoto Encyclopedia of Genes and Genomes (KEGG) pathways was conducted by employing the *clusterProfiler*, *org.Hs.eg.db*, and *topGo* packages with default parameters.

### 2.4. Protein-Protein Interaction (PPI) Network and Cross-Talking Pathways of IGF1

Module-trait relationships were assessed by the correlations between intramodular connectivity and genetic phenotype, as measured by module membership (MM) and gene significance (GS), respectively [23]. Scatterplots of the association between MM for module genes and GS for IGF1 expression were drawn using the *verboseScatterplot* function. Through the STRING (Search Tool for the Retrieval of Interacting Genes, http://www.stringdb.org/) online database, protein-protein interaction (PPI) network was established to investigate the physical or functional interaction between proteins that were encoded by the DEGs under consideration [24, 25]. The *cytoscape* software (Version 3.8.0, http://www.cytoscape.org/) was utilized to analyze and visualize the PPI network and the cross-talking pathways of IGF1 [26].

### 2.5. Diagnostic Performance of the Area Under the Curve (AUC)

AUC analysis is performed by comparing the receiver operating curve (ROC) of each subgroup in the cohort, providing the value of AUC, sensitivity, and specificity, as well as the best accuracy value at an optimal cut-off to estimate the positivity of a diagnosis or biomarker for AD [27]. The *pROC* function was used to detect the differentiation of IGF1 between AD cases and non-dementia controls. An AUC value of 100% represented a complete prediction, relative to a random selection with an AUC value of 50%.

### 2.6. Pathway Signature Genes and Gene Set Enrichment Analysis (GSEA)

Pearson's correlation coefficient (PCC) analysis was used to quantitatively evaluate the association between genes [28]. In each cross-talking pathway, the six genes with the top-rank PCC values were defined as the pathway signature genes, the expression of which were most strongly correlated with other genes of the pathway [29]. If IGF1 was significantly associated with a set of all pathway signature genes (*p* <0.05), this pathway could be served to be mediated or regulated by IGF1. The entire predefined differential expression gene lists were subjected to GSEA analysis to gain a complete biological process (BP) of gene ontology (GO) terms using the *GSEABase*, *enrichplot*, and *ClusterProfiler* packages [30]. The number and type for permutations were set to 1000 and phenotype, respectively. The *ggplot2* function was adopted to visualize the top 10 representative BPs enriched by GSEA analysis. All data were expressed as mean ± standard deviation (SD). Unpaired Student's *t*-test, one-way analysis of variance (ANOVA), or two-way ANOVA with Bonferroni's correction were used to assess the differences between groups. GraphPad Prism software (La Jolla, CA, USA) was utilized to perform statistical analysis. A two-tailed value of *p* less than 0.05 was considered to indicate statistical significance.

## 3. Results

### 3.1. Differentially Expressed Gene Identification

**Figure 1:**
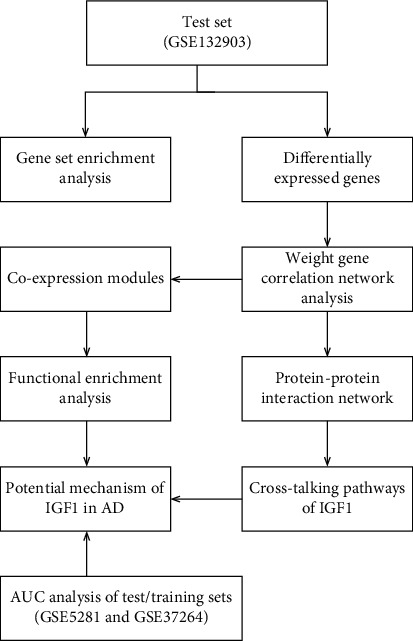
The flowchart for study design. AD: Alzheimer's disease; AUC: area under the curve.

**Figure 2:**
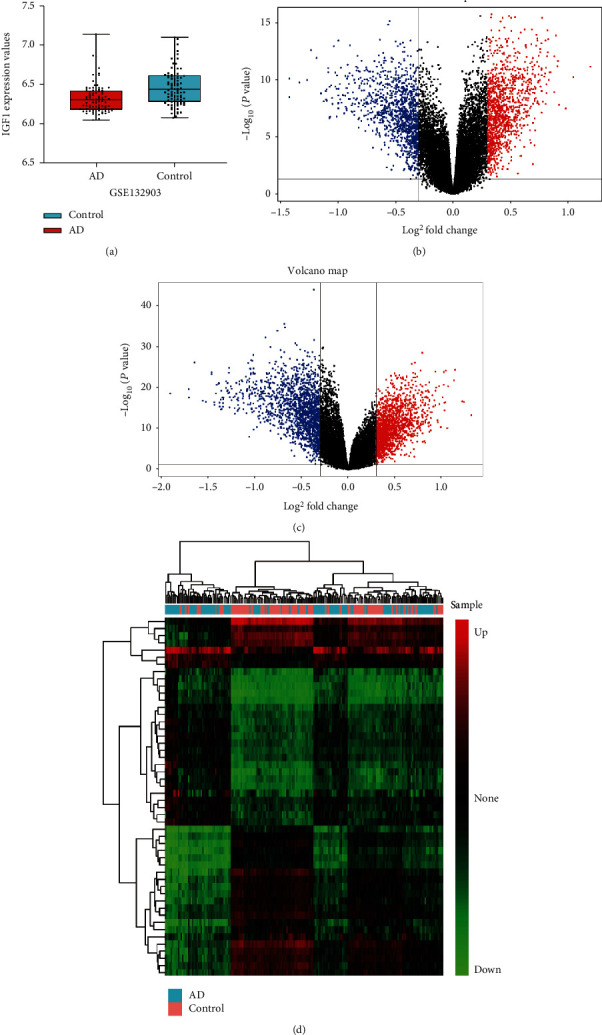
Differential expression analysis. (a) IGF1 expression between AD and non-dementia controls in GSE132903. Volcano plots of DEGs in AD/control (b) and IGF1-low/high (c) groups: blue and red represent down-regulated and up-regulated, respectively. (d) Clustering heatmap of the top 50 down- and up-regulated DEGs between AD and non-dementia controls: the gradual change of color from green to red indicates that the gene expression changes from down-regulation to up-regulation. AD: Alzheimer's disease; DEGs: differentially expressed genes.

The procedure for this study was followed by steps outlined in the flowchart (Figure 1). In test set of GSE132903, the expression level of IGF1 was significantly down-regulated in temporal lobes of 97 AD patients (5.38 ± 0.54) compared with 98 non-dementia controls (5.67 ± 0.45; *p* < 0.0001) (Figure 2(a)). Differential expression analysis on 19,245 background genes was conducted after eliminating invalid and repeated genes. As shown in volcano plots, 5,591 DEGs were selected in AD versus non-dementia controls, of which 2,805 were up-regulated and 2,786 were down-regulated (Figure 2(b)); meanwhile, 6,742 DEGs were identified in IGF1-low versus high group, of which 3,366 were up-regulated and 3,376 were down-regulated (Figure 2(c)). Ultimately, 4,424 DEGs (2,076 up-regulated and 2,348 down-regulated) were overlapped between AD/control and IGF1-low/high cohorts, which were subsequently extracted for two-dimensional hierarchical clustering analysis. The clustering results of the top 50 up- and down-regulated DEGs were presented in the heatmap (Figure 2(d)).

### 3.2. Gene Co-Expression Modules and Functional Enrichment Analysis

**Figure 3:**
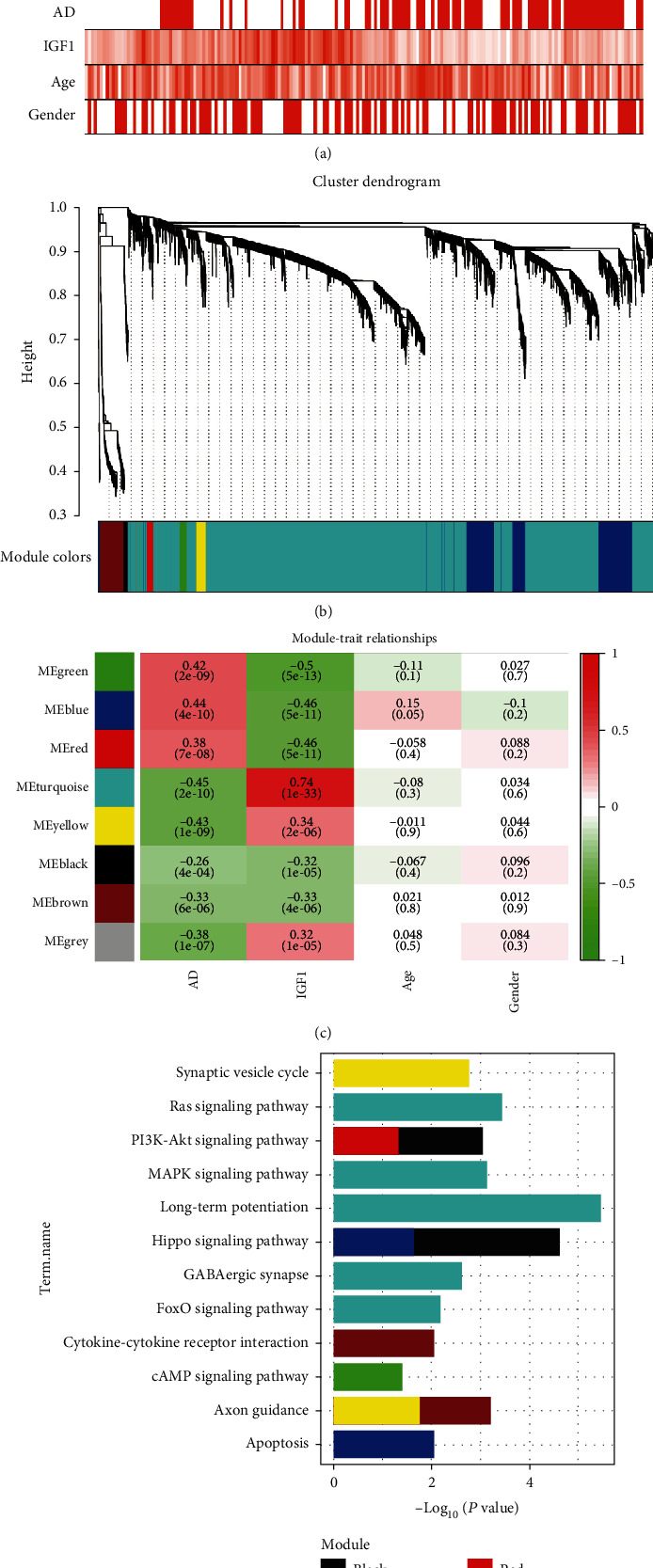
Weighted gene correlation network analysis. (a) Sample dendrogram and trait heatmap. (b) Cluster dendrogram of all DEGs with assigned module colors: non-clustering genes are displayed in gray. (c) Module-trait associations: the gradual change of color from green to red represents the changing correlation between ME and trait from negative to positive. (d) KEGG pathways enriched by module genes. AD: Alzheimer's disease; KEGG: Kyoto Encyclopedia of Genes and Genomes; ME: module eigengene.

After removing outliers above the cut-off line (height =60), 185 out of 195 samples were included for hierarchical clustering (Figure 3(a)). The DEGs with highly relevant expression were integrated into seven co-expression modules, while the remaining non-co-expressed DEGs were grouped into the gray module (Figure 3(b)). Heatmap of module-trait relationships (Figure 3(c)) exhibited that the turquoise module was most negatively associated with AD (correlation coefficient = -0.45, *p* =2e-10) and most positively related to IGF1 expression (correlation coefficient =0.74, *p* =1e-33). Co-expression modules were functionally annotated by KEGG pathway enrichment analysis (Figure 3(d)). In the turquoise module, DEGs were enriched in GABAergic synapse, long-term potentiation, mitogen-activated protein kinase (MAPK), Ras, and forkhead box O (FoxO) signaling pathways; in the black module, DEGs were involved in hippo and phosphoinositol-3-Kinase (PI3K)-Akt signaling pathways; in the blue module, genes participated in apoptosis and hippo signaling pathways; in the brown module, DEGs were implicated in cytokine-cytokine receptor interaction and axon guidance; and in the yellow, green, and red modules, DEGs were responsible for synaptic vesicle cycle, axon guidance, cAMP, and PI3K-Akt signaling pathways.

### 3.3. PPI Network and the AUC Analysis

**Figure 4:**
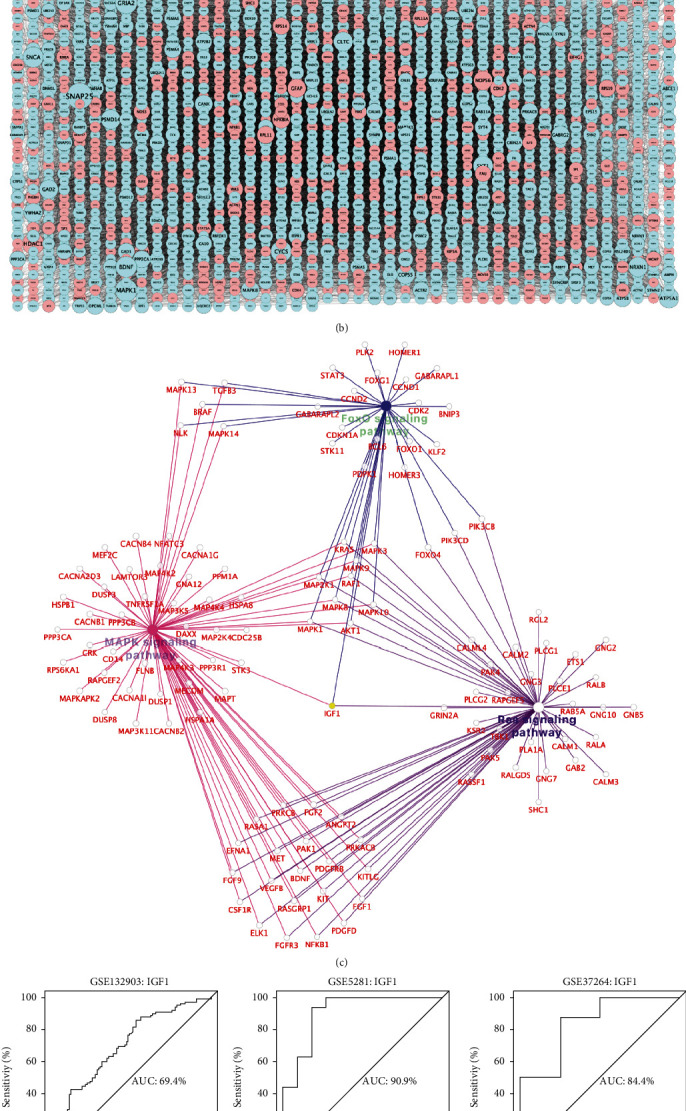
PPI network and AUC analysis. (a) Scatterplots of the relationship between MM (*x*-axis) and GS (*y*-axis). (b) PPI network based on turquoise module genes: node size reflects the degree of gene connectivity; down-regulated IGF1 is marked in yellow; blue and red represent down-regulated and up-regulated, respectively. (c) Cross-talking pathways of IGF1: down-regulated IGF1 is marked in yellow. (d) The predictive performance of IGF1 in AD measured by AUC analysis. (e) IGF1 expression between AD and non-dementia controls in training sets. AD: Alzheimer's disease: AUC: area under the curve; GS: gene significance; MM: module membership; PPI: protein-protein interaction.

Correlation analysis between MM for genes in each module and GS for IGF1 expression was conducted to detect the correlation coefficient of intramodular connectivity with genetic phenotypes. As shown in Figure 4(a), the turquoise module had the highest correlation coefficients between MM and GS (correlation coefficient =0.73, *p* <1e-200), which was consistent with the results of module-trait (IGF1) relationships. The MM and GS of each DEG in the co-expressed module are detailed in Supplementary Table 2. PPI network was constructed to display the interaction of DEGs in the turquoise module that were most closely correlated with IGF1 (Figure 4(b)). Further cross-talking pathways of IGF1 including MAPK, Ras, and FoxO signaling pathways were identified by functional enrichment analysis of KEGG (Figure 4(c)). According to the results of AUC analysis, low expression of IGF1 had a good diagnostic performance in AD prediction in test set (GSE132903: AUC =69.4%) and training sets (GSE5281: AUC =90.9%; GSE37264: AUC =84.4%) (Figure 4(d)). In comparison with non-dementia controls, down-regulation of IGF1 in AD was also observed in training sets (Figure 4(e)), including GSE5281 (AD: 4.76 ± 1.27 vs. controls: 6.63 ± 0.90; *p* < 0.001) and GSE37264 (AD: 5.04 ± 1.07 vs. controls: 5.99 ± 0.54; *p* = 0.04).

### 3.4. Validation of IGF1-Mediated Pathways and Biological Processes by GSEA

**Figure 5:**
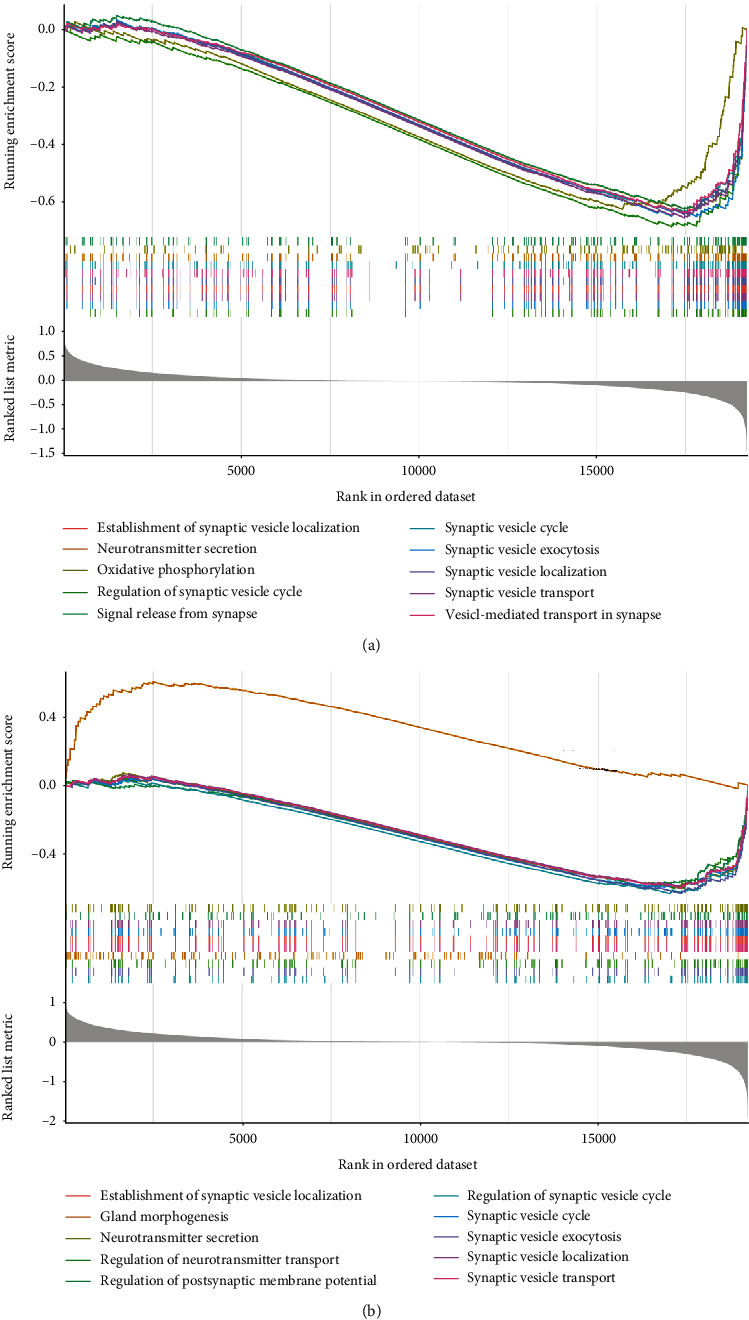
Gene set enrichment analysis. (a) Top 10 representative BPs enriched by GSEA in AD. (b) Top 10 representative BPs enriched by GSEA in IGF1-low cohort. AD: Alzheimer's disease; BP: biological process; GSEA: gene set enrichment analysis.

The signature genes in each cross-talking pathway are listed in Supplementary Table 3. As shown in Supplementary Figure 1, all pathway signature genes exhibited significantly positive or negative correlation with IGF1 expression (*p* < 0.05). Gene functional annotations of BPs in AD were related to neurotransmitter secretion, regulation of synaptic vesicle cycle, signal release from synapse, synaptic vesicle exocytosis, and synaptic vesicle transport (Figure 5(a)). Furthermore, the enriched BPs in IGF1-low cohort referred to neurotransmitter secretion, regulation of neurotransmitter transport, regulation of postsynaptic membrane potential, synaptic vesicle exocytosis, and synaptic vesicle transport (Figure 5(b)).

## 4. Discussion

In this study, we used publicly available RNA-seq data to screen for differentially expressed genes between AD and non-dementia cohorts. In line with the proteomic study [31], significant down-regulation of IGF1 expression in AD brains was observed in both test and training datasets. The GSEA analysis involving 19,245 background genes demonstrated that BPs of neurotransmitter secretion, synaptic vesicle exocytosis, and synaptic vesicle transport were associated with AD and low IGF1 expression. This was supported by *in vitro* evidence from PC12 cell lines that brief treatment with IGF1 activated intimal PI3K to enhance neurotransmitter secretion in a concentration-dependent manner [32], underlining the regulatory role of IGF1 in synaptic transmission of neurotransmitters. To decipher the signaling pathway of IGF1 in AD, we constructed a PPI network and co-expression modules of DEGs interacting with low IGF1 expression, thus to illuminate the genome-level pathogenesis of IGF1 underlying AD onset.

The results emerging from WGCNA showed that the turquoise module was overwhelmingly correlated with AD and IGF1 expression, in which DEGs were enriched in GABAergic synapse, long-term potentiation, MAPK, Ras, and FoxO signaling pathways. Under pathological stimulation, MAPK signaling is involved in a variety of intracellular events, such as proliferation, differentiation, survival, apoptosis, and transformation [33, 34]. As confirmed by astrocyte activation during the development of AD, multiple key stress-related responses are modulated by the MAPK cascades [35]. Additional evidence in support of this argument originates from *in vivo* and *in vitro* experiments with AD, wherein MAPKs subtypes, including p38 and c-Jun NH2 terminal kinase (JNK), are activated by A*β*-treated cultured astrocytes and cerebral cortex of rats [34]. Conversely, blockade of p38 signaling by the mitochondrial antioxidant SkQ1 alleviates phosphorylation of intermediate kinases and *α*B-crystallin, both of which have implications in senile plaques and tau protein inclusions [36]. Moreover, several lines of evidence have linked IGF1 to MAPKs by influencing cell proliferation and differentiation through extracellular signal related kinase 1 and 2 (ERK1/2) and p38 signaling pathways [37, 38]. For instance, IGF1 elicits the expression and translocation of intracellular androgen receptors, thereby enhancing p38 and ERK1/2 activities to increase the proliferation of C2C12 myoblasts [39]. Similar outcomes are also replicated in muscle satellite cells and skeletal muscle cells, i.e., EK1/2 signaling mediates the cell proliferation of IGF1 [40, 41], a role that can be completely suppressed by the ERK1/2 specific inhibitor PD98059 [37].

With except of the MAPK signaling pathway, Ras signaling has been linked to AD by regulating induction and post-translational modification of tau and amyloid-beta precursor protein (APP) [42, 43]. In frontal cortex of early AD, up-regulation of Ras expression acts on lysosomes and glycohydrolases, contributing to A*β* deposition and formation of paired helical filaments, which is suggestive of the progression of AD neurodegeneration [44, 45]. In this process, it is noteworthy that the Ras must be routed and anchored to the inner leaflet of plasma membrane for signal reception and transmission [46]. Dislodge of Ras from anchorage domains by the synthetic Ras inhibitor (e.g., FTS) enhances N-methyl-D-aspartate receptor (NMDAR)-dependent long-term potentiation (LTP) and blocks ERK-dependent LTP, hence improving synaptic plasticity and spatial memory [47, 48]. There is compelling evidence to support a relationship between IGF1 and Ras signaling pathway. Previous study has shown that IGF1 enhances Ras signaling through PI3K activation and thus to promote Lef/Tcf-dependent transcription, a process inhibited by dominant-negative mutants of Ha-Ras [49]. In addition, mice deficient in Rasgrf1, a guanine nucleotide-releasing factor of Ras, exhibits lower IGF1 levels responsible for learning and memory dysfunctions [50]. Consistently, our results provide evidence for the involvement of low IGF1 expression in Ras signaling pathway and that the up-regulation of IGF1 might be neuroprotective in AD.

In terms of FoxO signaling, transcription factors of the pathway are crucial regulators of cellular response to oxidative stress, a pathophysiological hallmark shared by insulin resistance, diabetic complications, and AD [51]. Mechanistically, short-term FoxO activation induced by oxidative stress involving Wingless proteins and beta-catenin pathway, which exerts antioxidant effects via regulating the transcription of antioxidant enzymes, manganese-superoxide dismutase, and catalase [52, 53]. Alternatively, long-term activation of FoxO due to an inability to cope with oxidative stress may predispose to hyperglycaemia and hyperinsulinaemia, which in turn fosters secondary oxidative stress related to the initiation of apoptosis [54, 55]. In the brain of established AD, oxidative stress-mediated FoxO activation is one of the important mechanisms underlying A*β*-induced cell death. Specifically, A*β* triggers FoxO phosphorylation in a p66Shc-dependent manner, promoting the generation of reactive oxygen species (ROS), lipid peroxides, and oxidized proteins, thus to the release of neurotoxic H_2_O_2_; intriguingly, ectopic expression of p66ShcS36A reduces FoxO phosphorylation, thereby preventing oxidative cell death against A*β* toxicity [56]. On the other hand, IGF1 encoding a paracrine or autocrine hormone is associated with FoxO transcription factors. In *Caenorhabditis elegans*, mutations in the IGF1 receptor homolog DAF-2 lead to metabolic, longevity, and developmental defects, which are alleviated by deletion of the FoxO homolog DAF-16 [57]. Additionally, FoxOs are transcriptional targets for fast induction of IGF1-mediated gluconeogenesis, which is reversed by insulin-stimulated nuclear rejection, implying a regulatory role of IGF1 in FoxO signaling pathway [58]. Likewise, these data are consistent with our findings that low expression of IGF1 is involved in the pathogenesis of AD through FoxO signaling pathway.

The scatterplot results between MM and GS confirmed the strongest correlation of DEGs with low IGF1 expression in the turquoise module. Based on these DEGs, a PPI network was constructed to identify the cross-talking pathways of IGF1, which supports the pluripotency of IGF1 in AD pathophysiology via MAPK, Ras, and FoxO signaling pathways. Of note, low expression of IGF1 is an important contributor to increased susceptibility of these relevant pathways during the development of AD, giving rise to subsequent impaired A*β* clearance and neuronal apoptosis [59–61]. According to AUC analysis of test set and validation of training sets, cohort subgroup comparisons revealed that low IGF1 could actually distinguish AD from non-dementia controls, indicating IGF1 to be a molecular target in predicting the onset of AD. As demonstrated by quantitative real-time polymerase chain reaction (qRT-PCR) experiment in advanced AD brains, IGF1 mRNA levels and overall expression of its receptors were apparently diminished with the increase of Braak stage [62]. Thenceforth, Fredue et al. found that insufficient expression or impaired function of IGF1 was dose-responsive to the severity of AD progression [63]. In view of this, IGF1 is envisioned as a potential candidate for the perspective of AD therapy that can be administered safely and systemically [31]. Additionally, PCC analysis demonstrated the significant correlation of IGF1 with all pathway signature genes, indicating that signature genes of each cross-talking pathway changed with the expression of IGF1. These data provided computational statistical evidence for the regulatory or mediated role of low IGF1 expression in MAPK, Ras, and FoxO signaling pathways. Future work should focus on the biological verification of the cross-talking pathways involved in the pathogenesis of low IGF1-mediated AD, thus facilitating the transition from a single target of IGF1 to multiline understanding of the holistic AD spectrum.

## 5. Conclusions

In summary, integrative genomic analysis is an attractive and effective approach to decipher complex mechanisms of the molecular signature that contribute to AD onset. Our findings reveal a pleiotropic role of low IGF1 expression in the pathogenesis of AD via MAPK, Ras, and FoxO signaling pathways, which may lead to a promising strategy in the prevention and treatment of AD by targeting IGF1.

## Figures and Tables

**Table 1:** Sample data in test and training sets. AD: Alzheimer's disease; GEO: gene expression omnibus.

GEO	Test/training set	Platform	AD	Controls
GSE132903	Test	GLP10558	97	98
GSE5281	Training	GPL570	16	11
GSE37264	Training	GPL5188	8	8

## Data Availability

The datasets referenced during the study are available in public repositories from the Gene Expression Omnibus database (https://www.ncbi.nlm.nih.gov/geo/) under accession numbers GSE132903, GSE5281, and GSE37264.

## Supplementary Materials

Supplementary 1 Supplementary Table 1: Sample phenotypic information. AD; Alzheimer's disease.

Supplementary 2 Supplementary Table 2: MM and GS for each differentially expressed gene in co-expression modules. MM: Module membership; GS: gene significance.

Supplementary 3 Supplementary Table 3: Identification of pathway representative genes by PCC analysis: pathway representative genes are highlighted in yellow. PCC: Pearson's correlation coefficient.

Supplementary 4 Supplementary Figure 1: Correlation between IGF1 and pathway signature genes. ∗*p* < 0.05.
