# Supplementary material for: Down-Regulation of Insulin Like Growth Factor 1 Involved in Alzheimer's Disease via MAPK, Ras, and FoxO Signaling Pathways
Source: Oxid Med Cell Longev. 2022 May 4;2022:8169981. doi: 10.1155/2022/8169981 (PMC9096571; doi:10.1155/2022/8169981)
Supplement: Supplementary 1 — Supplementary Table 1: Sample phenotypic information. AD; Alzheimer's disease. [file 8169981.f1.pdf]

		GSE132903		
Samples	Group	Age	Gender	IGF1
GSM238815	AD	75	Male	2.088222
GSM915119	AD	69	Male	2.529062
GSM238810	AD	81	Male	3.355774
GSM238824	AD	72	Male	4.543965
GSM333287(	AD	75	Male	4.587896
GSM333303:	AD	90	Male	4.710089
GSM238813	AD	78	Male	4.727213
GSM333300·	AD	68	Male	4.769507
GSM238812	AD	75	Male	4.978821
GSM238821	AD	79	Male	5.04476
GSM907825	AD	84	Male	5.081739
GSM907828	AD	88	Male	5.156479
GSM389611'	AD	93	Male	5.183066
GSM915113	AD	67	Male	5.194302
GSM333280.	AD	88	Male	5.211831
GSM389607(	AD	87	Male	5.230779
GSM389610:	AD	76	Male	5.247576
GSM389610(	AD	98	Male	5.266658
GSM389608:	AD	91	Male	5.272564
GSM389611(	AD	88	Male	5.276265
GSM389603:	AD	80	Male	5.278235
GSM389606:	AD	72	Male	5.288972
GSM389606.	AD	75	Male	5.292557
GSM389609:	AD	82	Male	5.296167
GSM389610'	AD	75	Male	5.298546
GSM389607:	AD	83	Male	5.305544
GSM389612:	AD	73	Male	5.328532
GSM389606:	AD	95	Male	5.334588
GSM389606	AD	90	Male	5.335299
GSM915122	AD	78	Male	5.339812
GSM333279(	AD	97	Male	5.341187
GSM389609:	AD	83	Male	5.344768
GSM389606·	AD	87	Male	5.35066
GSM389605(	AD	80	Male	5.351872
GSM389604·	AD	80	Male	5.35343
GSM333287	AD	69	Male	5.367728
GSM389609(	AD	71	Male	5.381176
GSM389608.	AD	84	Male	5.392352
GSM389605:	AD	92	Male	5.402426
GSM333285·	AD	71	Male	5.407012
GSM389609.	AD	74	Male	5.425445
GSM389611(	AD	84	Male	5.425616
GSM333277:	AD	88	Male	5.428426
GSM389610.	AD	84	Male	5.429481

GSM389609	AD	87	Male	5.445362
GSM389609	AD	85	Male	5.45423
GSM333289	AD	72	Male	5.470067
GSM389606	AD	90	Male	5.4818
GSM333302	AD	89	Male	5.482516
GSM389608	AD	79	Male	5.498655
GSM389605	AD	86	Male	5.50049
GSM333294	AD	76	Male	5.503188
GSM389613	AD	82	Male	5.51958
GSM389607	AD	81	Male	5.540259
GSM389612	AD	89	Male	5.540587
GSM238818	AD	68	Male	5.546245
GSM907833	AD	83	Male	5.549169
GSM389608	AD	86	Male	5.549384
GSM333272	AD	66	Male	5.553387
GSM333284	AD	80	Male	5.554635
GSM389612	AD	88	Male	5.55807
GSM333279	AD	89	Male	5.569688
GSM389607	AD	83	Male	5.576389
GSM389608	AD	88	Male	5.578573
GSM389607	AD	85	Male	5.608072
GSM389604	AD	78	Male	5.609977
GSM333281	AD	86	Male	5.637961
GSM389608	AD	97	Male	5.641984
GSM389612	AD	89	Male	5.656055
GSM389607	AD	94	Male	5.68608
GSM333292	AD	81	Male	5.720371
GSM333295	AD	66	Male	5.73566
GSM389604	AD	81	Male	5.739251
GSM389608	AD	92	Male	5.743714
GSM389608	AD	70	Male	5.754717
GSM333289	AD	105	Male	5.79017
GSM389604	AD	79	Male	5.802818
GSM907832	AD	89	Male	5.816599
GSM389613	AD	82	Male	5.821358
GSM915112	AD	83	Male	5.854882
GSM907834	AD	90	Male	5.876349
GSM333297	AD	79	Male	5.88412
GSM915115	AD	66	Male	5.922412
GSM389613	AD	85	Male	5.97718
GSM238816	AD	87	Male	6.003747
GSM238811	AD	72	Male	6.158009
GSM333290	AD	67	Male	6.194876
GSM389604	AD	76	Male	6.250443
GSM238823	AD	88	Male	6.302871
GSM119645	Control	79	Male	4.747987

GSM907846	Control	80	Male	4.997419
GSM333288	Control	80	Male	5.041523
GSM915123	Control	78	Male	5.132132
GSM389595	Control	82	Male	5.196735
GSM333276	Control	77	Male	5.198359
GSM389598	Control	89	Male	5.21044
GSM119651	Control	69	Male	5.226554
GSM907851	Control	74	Male	5.245159
GSM389597	Control	90	Male	5.248542
GSM389614	Control	76	Male	5.261397
GSM389613	Control	91	Male	5.268446
GSM389596	Control	85	Male	5.290109
GSM389598	Control	84	Male	5.30091
GSM333294	Control	78	Male	5.346265
GSM389601	Control	80	Male	5.350949
GSM389614	Control	90	Male	5.353737
GSM389596	Control	70	Male	5.355172
GSM389596	Control	89	Male	5.356386
GSM389598	Control	92	Male	5.36949
GSM389599	Control	82	Male	5.376324
GSM389602	Control	95	Male	5.386559
GSM389596	Control	77	Male	5.390743
GSM389598	Control	85	Male	5.39442
GSM389600	Control	80	Male	5.424277
GSM389599	Control	73	Male	5.439196
GSM333308	Control	65	Male	5.442037
GSM389595	Control	86	Male	5.455844
GSM389597	Control	83	Male	5.456151
GSM389597	Control	85	Male	5.478196
GSM333299	Control	67	Male	5.487295
GSM389600	Control	74	Male	5.491094
GSM389599	Control	91	Male	5.506551
GSM389595	Control	78	Male	5.510566
GSM389600	Control	86	Male	5.51823
GSM389613	Control	79	Male	5.521
GSM333284	Control	40	Male	5.521925
GSM333299	Control	51	Male	5.533259
GSM389601	Control	92	Male	5.556504
GSM389599	Control	100	Male	5.55807
GSM907848	Control	89	Male	5.564179
GSM389599	Control	75	Male	5.599277
GSM389613	Control	81	Male	5.600849
GSM389597	Control	94	Male	5.617434
GSM389614	Control	81	Male	5.619081
GSM389597	Control	84	Male	5.625953
GSM389601	Control	89	Male	5.631671

GSM907843	Control	82	Male	5.657509
GSM333282	Control	73	Male	5.66287
GSM907842	Control	89	Male	5.680909
GSM389600	Control	87	Male	5.705936
GSM389599	Control	82	Male	5.719809
GSM389595	Control	91	Male	5.738116
GSM907839	Control	77	Male	5.745219
GSM389601	Control	90	Male	5.773462
GSM389598	Control	88	Male	5.816828
GSM389595	Control	87	Male	5.823916
GSM389596	Control	79	Male	5.877588
GSM389614	Control	83	Male	5.879315
GSM333296	Control	41	Male	5.884721
GSM389611	Control	75	Male	5.934081
GSM389600	Control	76	Male	5.939197
GSM907845	Control	83	Male	5.941229
GSM333282	Control	78	Male	5.964473
GSM333275	Control	81	Male	5.967424
GSM915118	Control	66	Male	5.978752
GSM389601	Control	84	Male	5.986249
GSM389601	Control	80	Male	5.996439
GSM333303	Control	66	Male	6.032206
GSM907840	Control	55	Male	6.042739
GSM333281	Control	95	Male	6.057302
GSM389601	Control	87	Male	6.113476
GSM389613	Control	72	Male	6.121825
GSM389601	Control	71	Male	6.170927
GSM389614	Control	83	Male	6.186004
GSM915117	Control	87	Male	6.187262
GSM119647	Control	79	Male	6.225791
GSM333283	Control	86	Male	6.258351
GSM915110	Control	85	Male	6.292442
GSM119641	Control	85	Male	6.515928
GSM915111	Control	77	Male	6.648112
GSM119652	Control	78	Male	6.947981
GSM119642	Control	80	Male	6.951905
GSM119646	Control	83	Male	7.209129
GSM119644	Control	63	Male	7.294782
GSM238809	AD	73	Female	2.281488
GSM238817	AD	90	Female	4.083967
GSM238819	AD	81	Female	4.587107
GSM333298	AD	90	Female	4.59522
GSM333273	AD	72	Female	4.779341
GSM915124	AD	67	Female	4.944092
GSM333290	AD	79	Female	5.070144
GSM333304	AD	63	Female	5.084425

GSM238825	AD	80	Female	5.120605
GSM389605	AD	86	Female	5.165991
GSM389606	AD	88	Female	5.183895
GSM389607	AD	95	Female	5.237262
GSM389612	AD	91	Female	5.244036
GSM907826	AD	88	Female	5.244159
GSM389607	AD	81	Female	5.247647
GSM389613	AD	85	Female	5.249325
GSM915114	AD	88	Female	5.265662
GSM389605	AD	82	Female	5.266292
GSM389605	AD	90	Female	5.268314
GSM389608	AD	95	Female	5.278076
GSM389604	AD	87	Female	5.279665
GSM389612	AD	90	Female	5.285296
GSM389610	AD	77	Female	5.295168
GSM389609	AD	73	Female	5.299009
GSM389603	AD	87	Female	5.301971
GSM389605	AD	92	Female	5.303821
GSM389607	AD	85	Female	5.307637
GSM389606	AD	88	Female	5.309045
GSM333299	AD	83	Female	5.311449
GSM389604	AD	81	Female	5.322332
GSM389603	AD	92	Female	5.331337
GSM389603	AD	77	Female	5.344802
GSM389610	AD	70	Female	5.34593
GSM389606	AD	95	Female	5.351191
GSM333288	AD	96	Female	5.360326
GSM333292	AD	84	Female	5.361146
GSM907830	AD	95	Female	5.371029
GSM389604	AD	91	Female	5.385882
GSM389610	AD	87	Female	5.390149
GSM389604	AD	86	Female	5.397069
GSM389611	AD	87	Female	5.401249
GSM333282	AD	83	Female	5.402744
GSM389610	AD	96	Female	5.426692
GSM389612	AD	97	Female	5.428267
GSM907829	AD	91	Female	5.428499
GSM389609	AD	85	Female	5.431017
GSM389610	AD	78	Female	5.4375
GSM389603	AD	84	Female	5.448913
GSM907827	AD	95	Female	5.448959
GSM389609	AD	78	Female	5.456596
GSM389609	AD	90	Female	5.46138
GSM389603	AD	91	Female	5.465248
GSM333307	AD	98	Female	5.473826
GSM389605	AD	82	Female	5.480759

GSM238820	AD	85	Female	5.487421
GSM389611	AD	77	Female	5.502639
GSM389608	AD	82	Female	5.506775
GSM389603	AD	97	Female	5.527215
GSM389606	AD	86	Female	5.527837
GSM389611	AD	75	Female	5.537248
GSM389603	AD	87	Female	5.540804
GSM333305	AD	83	Female	5.544081
GSM333291	AD	87	Female	5.564428
GSM389604	AD	91	Female	5.565806
GSM389605	AD	89	Female	5.566867
GSM389607	AD	85	Female	5.568021
GSM333290	AD	92	Female	5.57408
GSM389610	AD	94	Female	5.574995
GSM389612	AD	78	Female	5.575396
GSM333287	AD	82	Female	5.611226
GSM915121	AD	63	Female	5.623752
GSM333281	AD	85	Female	5.661613
GSM333299	AD	95	Female	5.667416
GSM333268	AD	84	Female	5.718577
GSM333289	AD	81	Female	5.719943
GSM389605	AD	87	Female	5.731082
GSM333294	AD	80	Female	5.749578
GSM333273	AD	93	Female	5.805477
GSM333278	AD	90	Female	5.809771
GSM333293	AD	71	Female	5.810477
GSM238822	AD	82	Female	5.859118
GSM907831	AD	95	Female	5.881529
GSM333281	AD	85	Female	5.89153
GSM333294	AD	79	Female	5.927605
GSM333303	AD	88	Female	5.940106
GSM389612	AD	91	Female	5.963142
GSM333272	AD	96	Female	6.147121
GSM333308	Control	73	Female	4.728931
GSM333275	Control	87	Female	5.027353
GSM915120	Control	81	Female	5.161312
GSM333305	Control	88	Female	5.18314
GSM333307	Control	80	Female	5.203212
GSM907853	Control	54	Female	5.244089
GSM389600	Control	84	Female	5.25007
GSM389597	Control	96	Female	5.255134
GSM333270	Control	92	Female	5.302558
GSM389595	Control	88	Female	5.303655
GSM907849	Control	65	Female	5.318409
GSM389602	Control	95	Female	5.321216
GSM389595	Control	90	Female	5.357053

GSM389598	Control	98	Female	5.371884
GSM389602	Control	86	Female	5.389371
GSM907852	Control	86	Female	5.392819
GSM389602	Control	87	Female	5.395708
GSM389595	Control	87	Female	5.398213
GSM907847	Control	75	Female	5.405709
GSM907837	Control	80	Female	5.409919
GSM389597	Control	73	Female	5.419477
GSM907835	Control	77	Female	5.421619
GSM389598	Control	78	Female	5.425858
GSM389596	Control	95	Female	5.43002
GSM389598	Control	87	Female	5.433598
GSM389597	Control	90	Female	5.456332
GSM389596	Control	88	Female	5.464559
GSM389599	Control	77	Female	5.467816
GSM389596	Control	82	Female	5.476838
GSM333280	Control	91	Female	5.48707
GSM389613	Control	82	Female	5.489244
GSM389598	Control	83	Female	5.51254
GSM389596	Control	102	Female	5.517773
GSM389614	Control	70	Female	5.537213
GSM389600	Control	75	Female	5.54259
GSM907836	Control	87	Female	5.549829
GSM907850	Control	71	Female	5.563049
GSM333269	Control	51	Female	5.569764
GSM389597	Control	85	Female	5.571261
GSM389599	Control	87	Female	5.573422
GSM907841	Control	72	Female	5.588709
GSM389611	Control	77	Female	5.601345
GSM389596	Control	77	Female	5.626503
GSM389601	Control	86	Female	5.638664
GSM389599	Control	90	Female	5.651991
GSM389611	Control	81	Female	5.661472
GSM389603	Control	89	Female	5.673416
GSM333301	Control	43	Female	5.67851
GSM389595	Control	92	Female	5.688623
GSM915125	Control	88	Female	5.688682
GSM389597	Control	87	Female	5.693511
GSM389602	Control	85	Female	5.694735
GSM389613	Control	81	Female	5.703345
GSM389600	Control	91	Female	5.715282
GSM389598	Control	86	Female	5.719233
GSM389600	Control	91	Female	5.723455
GSM389601	Control	80	Female	5.728555
GSM907838	Control	84	Female	5.734129
GSM389599	Control	96	Female	5.738733

GSM907844	Control	78	Female	5.739669
GSM389611	Control	92	Female	5.748496
GSM389602	Control	83	Female	5.757999
GSM389603	Control	91	Female	5.81113
GSM333274	Control	52	Female	5.813548
GSM389602	Control	82	Female	5.816285
GSM389600	Control	94	Female	5.902158
GSM389612	Control	78	Female	5.939197
GSM389602	Control	91	Female	6.021094
GSM333269	Control	82	Female	6.025974
GSM389602	Control	85	Female	6.038977
GSM389602	Control	95	Female	6.210436
GSM915116	Control	79	Female	6.566612
GSM119650	Control	73	Female	6.835601
GSM119648	Control	88	Female	7.318032
GSM119643	Control	102	Female	7.612644

GSE5281

Samples	Group	Age	Gender	IGF1
GSM119641	Control	85	Male	6.515928
GSM119642	Control	80	Male	6.951905
GSM119643	Control	102	Female	7.612644
GSM119644	Control	63	Male	7.294782
GSM119645	Control	79	Male	4.747987
GSM119646	Control	83	Male	7.209129
GSM119647	Control	79	Male	6.225791
GSM119648	Control	88	Female	7.318032
GSM119650	Control	73	Female	6.835601
GSM119651	Control	69	Male	5.226554
GSM119652	Control	78	Male	6.947981
GSM238809	AD	73	Female	2.281488
GSM238810	AD	81	Male	3.355774
GSM238811	AD	72	Male	6.158009
GSM238812	AD	75	Male	4.978821
GSM238813	AD	78	Male	4.727213
GSM238815	AD	75	Male	2.088222
GSM238816	AD	87	Male	6.003747
GSM238817	AD	90	Female	4.083967
GSM238818	AD	68	Male	5.546245
GSM238819	AD	81	Female	4.587107
GSM238820	AD	85	Female	5.487421
GSM238821	AD	79	Male	5.04476
GSM238822	AD	82	Female	5.859118
GSM238823	AD	88	Male	6.302871
GSM238824	AD	72	Male	4.543965
GSM238825	AD	80	Female	5.120605

GSE37264

Samples	Group	Age	Gender	IGF1
GSM915110	Control	85	Male	6.292442
GSM915111	Control	77	Male	6.648112
GSM915112	AD	83	Male	5.978752
GSM915113	AD	67	Male	2.529062
GSM915114	AD	88	Female	5.161312
GSM915115	AD	66	Male	5.623752
GSM915116	Control	79	Female	5.854882
GSM915117	Control	87	Male	5.194302
GSM915118	Control	66	Male	5.265662
GSM915119	AD	69	Male	5.339812
GSM915120	Control	81	Female	5.922412
GSM915121	AD	63	Female	5.132132
GSM915122	AD	78	Male	4.944092
GSM915123	Control	78	Male	6.566612
GSM915124	AD	67	Female	5.688682
GSM915125	Control	88	Female	6.187262
