# Supplementary material for: Down-Regulation of Insulin Like Growth Factor 1 Involved in Alzheimer's Disease via MAPK, Ras, and FoxO Signaling Pathways
Source: Oxid Med Cell Longev. 2022 May 4;2022:8169981. doi: 10.1155/2022/8169981 (PMC9096571; doi:10.1155/2022/8169981)
Supplement: Supplementary 2 — Supplementary Table 2: MM and GS for each differentially expressed gene in co-expression modules. MM: Module membership; GS: gene significance. [file 8169981.f2.pdf]

Gene.symbc	moduleColc	GS.IGF1	p.GS.IGF1	MMgreen	p.MMgreen	MMblue
ZNF385A	black	-0.3218359	7.92E-06	0.109599	0.13752231	-0.3351149
TRIM28	black	-0.3419534	1.90E-06	-0.0194323	0.79290385	-0.3992022
TMEM160	black	-0.2214295	0.00245328	-0.1221374	0.09768222	-0.5157082
PTPN23	black	-0.3262208	5.85E-06	-0.0463926	0.53061412	-0.366121
PTMS	black	-0.275284	0.00014918	-0.1350798	0.06676653	-0.3985998
PRRC2A	black	-0.3240806	6.79E-06	-0.106067	0.15073329	-0.3168355
PODXL2	black	-0.2117478	0.00381043	-0.3341715	3.34E-06	-0.3562637
PNPLA2	black	-0.3193788	9.37E-06	-0.0820789	0.26669664	-0.373827
PLXNB3	black	-0.1974821	0.00705094	-0.0502861	0.49665723	-0.3726262
PLPPR2	black	-0.2026039	0.00567874	-0.1620892	0.02750284	-0.4382963
NFIX	black	-0.3622431	4.05E-07	-0.0838808	0.25630338	-0.1674904
MDK	black	-0.2471318	0.00069554	0.06006468	0.41669739	-0.2656544
KMT2A	black	-0.245366	0.00076176	-0.0241636	0.74406086	-0.5218541
JOSD2	black	-0.2896927	6.34E-05	-0.0682716	0.35580983	-0.3065513
INO80E	black	-0.2991463	3.53E-05	-0.0521018	0.4812236	-0.3302633
HCN2	black	-0.1681722	0.02212545	-0.1435418	0.05126524	-0.4834317
HBA1	black	-0.2916046	5.64E-05	0.1641627	0.02555615	-0.2007596
GHDC	black	-0.2464332	0.00072108	-0.0406589	0.58266264	-0.4568088
EPHX1	black	-0.4358334	5.62E-10	0.23802922	0.00110385	-0.1887531
DDN	black	-0.1725196	0.01886181	-0.1850934	0.0116586	-0.1069413
CST3	black	-0.3727531	1.74E-07	0.19235023	0.00871454	-0.3545915
CSNK2B	black	-0.411129	6.13E-09	-0.0990945	0.17959568	-0.2145337
COPE	black	-0.2246549	0.00210982	-0.0383088	0.60465744	-0.4861452
CLPTM1	black	-0.2467429	0.00070965	-0.1277153	0.08319465	-0.3904221
CACNA1A	black	-0.2337055	0.00136644	-0.2389932	0.00105201	-0.3415404
CA11	black	-0.222951	0.00228539	-0.2104137	0.00404292	-0.4215128
BSG	black	-0.3163465	1.15E-05	-0.0137238	0.85290961	-0.370064
BANF1	black	-0.235248	0.00126683	-0.0070702	0.92390636	-0.5117212
ATP1B2	black	-0.3937872	2.93E-08	0.29969202	3.41E-05	-0.3278468
ATN1	black	-0.3086273	1.92E-05	0.07408828	0.31621921	-0.3802037
ARPC4	black	-0.2298592	0.00164681	-0.2591488	0.00036809	-0.3629786
ABCA2	black	-0.3104208	1.71E-05	-0.0783083	0.28936453	-0.3459015
ZSWIM1	blue	-0.2637987	0.00028539	-0.0157769	0.83121177	0.82863729
ZRSR2	blue	-0.4733744	1.01E-11	0.22604672	0.00197561	0.89111682
ZRANB1	blue	-0.3901698	4.02E-08	0.19771258	0.00698335	0.74308535
ZNF827	blue	-0.4877712	1.90E-12	0.17456214	0.01747847	0.87803798
ZNF788	blue	-0.2754628	0.00014765	0.39053053	3.89E-08	0.75190069
ZNF787	blue	-0.538838	2.53E-15	0.34224419	1.86E-06	0.90392719
ZNF786	blue	-0.4561909	6.77E-11	0.38926945	4.34E-08	0.91598022
ZNF771	blue	-0.4940272	8.93E-13	0.40034452	1.64E-08	0.90098233
ZNF766	blue	-0.2879879	7.04E-05	0.2735324	0.000165	0.82241865
ZNF69	blue	0.06703485	0.36461148	0.01449579	0.84473697	0.02790688
ZNF672	blue	-0.5403051	2.06E-15	0.38423841	6.68E-08	0.88069609
ZNF669	blue	-0.3350704	3.13E-06	0.1801385	0.01414154	0.80251893
ZNF652	blue	-0.3468202	1.32E-06	0.36646367	2.89E-07	0.84927136

ZNF598	blue	-0.3986513	1.91E-08	0.15298323	0.03762222	0.82458409
ZNF562	blue	-0.2946901	4.66E-05	0.13955727	0.05814534	0.8864563
ZNF557	blue	-0.2102558	0.00407126	0.16663649	0.02338941	0.74682003
ZNF526	blue	-0.3929883	3.14E-08	0.20310576	0.00555808	0.92090432
ZNF493	blue	-0.3136024	1.38E-05	0.16476473	0.02501358	0.83211587
ZNF483	blue	0.1255615	0.08856956	-0.0592592	0.42298455	0.03339688
ZNF462	blue	-0.5054037	2.18E-13	0.4770696	6.63E-12	0.86344624
ZNF320	blue	-0.3297731	4.56E-06	0.1682313	0.02207801	0.7857548
ZNF223	blue	-0.3530379	8.27E-07	0.32403827	6.81E-06	0.84994374
ZNF184	blue	0.52064747	3.04E-14	-0.5363412	3.60E-15	-0.8238557
ZNF160	blue	-0.5394325	2.33E-15	0.42036148	2.57E-09	0.89276557
ZNF148	blue	-0.4041215	1.17E-08	0.23980497	0.0010101	0.81098006
ZNF14	blue	0.13532808	0.06626258	0.01335448	0.85682482	0.26076092
ZHX3	blue	-0.4528819	9.64E-11	0.56052681	1.07E-16	0.8718307
ZHX2	blue	-0.233211	0.00139986	0.46586373	2.35E-11	0.77684201
ZFP14	blue	-0.2414901	0.00092795	0.14314158	0.05192425	0.72258201
ZDHHC8	blue	-0.5343141	4.77E-15	0.40431032	1.15E-08	0.90846869
ZDHHC1	blue	-0.2028869	0.00561042	0.12193596	0.09824075	0.87791957
ZCWPW1	blue	-0.2869345	7.50E-05	0.12541157	0.08895381	0.80003558
ZCCHC6	blue	-0.454842	7.82E-11	0.31552863	1.22E-05	0.90884567
ZC3H3	blue	-0.3922446	3.35E-08	0.17657293	0.01620367	0.91753466
ZC2HC1C	blue	-0.268126	0.00022427	0.41361015	4.86E-09	0.82721724
ZBTB48	blue	-0.3251691	6.30E-06	0.08824163	0.23231664	0.86676974
ZBTB46	blue	-0.4713119	1.28E-11	0.33615534	2.90E-06	0.86324712
ZBTB45	blue	-0.1342136	0.06854925	0.12317152	0.09485507	0.76401468
ZBTB42	blue	-0.4439177	2.47E-10	0.46742869	1.98E-11	0.84883153
XAF1	blue	-0.5040336	2.59E-13	0.41906967	2.90E-09	0.80934003
WWC1	blue	-0.3967899	2.25E-08	0.56160141	9.09E-17	0.81645263
WTIP	blue	-0.2579967	0.00039177	0.4331957	7.33E-10	0.77838514
WHAMM	blue	-0.403517	1.23E-08	0.20535437	0.00504512	0.85310028
WFS1	blue	-0.4465431	1.88E-10	0.36621446	2.95E-07	0.85234205
WDR83OS	blue	-0.3501097	1.03E-06	0.27638699	0.00013996	0.90709406
WBP11	blue	0.35624935	6.46E-07	-0.29615	4.26E-05	-0.8619369
VGLL4	blue	-0.3937655	2.94E-08	0.38907164	4.42E-08	0.88406929
VEZT	blue	-0.3417792	1.92E-06	0.09079448	0.2190311	0.74639284
VAV2	blue	-0.3177903	1.04E-05	-0.0271009	0.71423047	0.8336624
VAMP2	blue	0.19518245	0.00775785	-0.4997251	4.44E-13	-0.7871137
USP22	blue	0.36423657	3.46E-07	-0.379348	1.01E-07	-0.7788854
UPP1	blue	-0.2837636	9.07E-05	0.06744483	0.36167906	0.78251548
UNC119	blue	-0.2496061	0.00061165	0.40483857	1.09E-08	0.69546931
UGGT1	blue	-0.3099404	1.76E-05	0.23349739	0.00138041	0.73266482
UCP3	blue	-0.4343037	6.56E-10	0.25910611	0.00036895	0.91965025
UBXN2A	blue	-0.5716838	1.91E-17	0.51890774	3.83E-14	0.92170287
UBC	blue	0.34100099	2.04E-06	-0.3555601	6.81E-07	-0.7003194
UBA7	blue	-0.3970975	2.19E-08	0.44053552	3.49E-10	0.81341025
U2AF2	blue	-0.3697248	2.23E-07	0.4519362	1.07E-10	0.81663304

TYW3	blue	-0.2828141	9.60E-05	0.05148671	0.48642238	0.79487564
TXLNA	blue	-0.3893658	4.31E-08	0.29215514	5.46E-05	0.95763976
TUSC1	blue	0.33723444	2.68E-06	-0.4182936	3.13E-09	-0.8100819
TUG1	blue	-0.5431413	1.38E-15	0.44133308	3.22E-10	0.84338849
TUBA1C	blue	0.35706597	6.06E-07	-0.3545751	7.35E-07	-0.9244715
TSSC4	blue	-0.5116795	9.81E-14	0.21171008	0.00381683	0.87729606
TSPAN7	blue	0.45214622	1.04E-10	-0.4042002	1.16E-08	-0.8771528
TSPAN4	blue	-0.4427237	2.79E-10	0.51447372	6.84E-14	0.83815634
TSPAN33	blue	-0.354554	7.36E-07	0.2899105	6.26E-05	0.877272
TSPAN31	blue	-0.3051266	2.41E-05	0.38859307	4.60E-08	0.84336788
TRPT1	blue	-0.3163692	1.15E-05	0.3492838	1.10E-06	0.79452135
TRPM4	blue	-0.323289	7.17E-06	0.29913123	3.53E-05	0.76668079
TRIM25	blue	-0.4415791	3.14E-10	0.46908736	1.64E-11	0.75030531
TRAP1	blue	0.31233735	1.50E-05	-0.4400684	3.66E-10	-0.7551267
TRAK2	blue	-0.4444566	2.33E-10	0.35202182	8.93E-07	0.75726939
TRAF3IP1	blue	-0.2356408	0.00124255	0.00986246	0.89400513	0.75916816
TRABD	blue	-0.5382697	2.75E-15	0.49236173	1.09E-12	0.94761982
TPPP	blue	0.27279169	0.00017215	-0.4310262	9.09E-10	-0.7912012
TPM3	blue	-0.4590265	4.98E-11	0.12005556	0.10357637	0.71136732
TPI1	blue	0.20404691	0.00533796	-0.5188875	3.84E-14	-0.6452754
TPCN2	blue	-0.216883	0.00302377	0.180433	0.01398197	0.78727405
TP53I13	blue	-0.3639343	3.54E-07	0.40483855	1.09E-08	0.94403066
TOB2	blue	-0.5592678	1.29E-16	0.48784259	1.88E-12	0.80577917
TNFSF14	blue	-0.423156	1.96E-09	0.39178596	3.49E-08	0.90599104
TNFAIP8L1	blue	-0.1669508	0.02312577	0.09154533	0.21522861	0.57240191
TMX2	blue	0.24342825	0.00084108	-0.2145877	0.00335516	-0.594185
TMLHE	blue	-0.3550099	7.11E-07	0.51438258	6.92E-14	0.68824918
TMEM91	blue	-0.3303036	4.40E-06	0.16877316	0.02164713	0.90768874
TMEM79	blue	-0.4001261	1.67E-08	0.30534219	2.38E-05	0.81406255
TMEM38B	blue	-0.2860596	7.90E-05	0.26442275	0.00027571	0.69234847
TMEM267	blue	-0.307422	2.08E-05	0.18973835	0.00968802	0.67127298
TMEM216	blue	-0.5094174	1.31E-13	0.61284527	1.84E-20	0.89543834
TMEM191A	blue	-0.3985762	1.92E-08	0.23737383	0.00114042	0.81107982
TMEM189	blue	-0.4622241	3.51E-11	0.42511283	1.62E-09	0.83869357
TMEM175	blue	-0.2352374	0.00126749	-0.0565333	0.44466722	0.77246819
TMEM156	blue	-0.4233405	1.93E-09	0.27599727	0.00014315	0.87675379
TMEM150A	blue	-0.4709358	1.33E-11	0.62273421	2.97E-21	0.84906987
TMEM115	blue	-0.2927244	5.27E-05	0.22142352	0.00245396	0.9034083
TMEM106A	blue	-0.2290344	0.00171339	0.20824071	0.00444906	0.73691
TMED1	blue	-0.3978982	2.04E-08	0.51974842	3.43E-14	0.79484187
TM9SF4	blue	0.17641657	0.0162998	-0.3667108	2.84E-07	-0.6822442
TICAM2	blue	-0.2993539	3.48E-05	0.45539821	7.37E-11	0.72585121
TFAP2C	blue	-0.2588267	0.00037458	0.61012669	3.01E-20	0.6875625
TERF2	blue	0.18212746	0.01309413	-0.2390388	0.00104962	-0.8506618
TDP1	blue	0.07256501	0.32629899	-0.0069368	0.92533774	0.09913539
TCL1B	blue	-0.3866592	5.44E-08	0.28940453	6.46E-05	0.80813311

TCEA1	blue	-0.4269737	1.35E-09	0.38221937	7.93E-08	0.82690662
TCAP	blue	-0.5056631	2.11E-13	0.40960986	7.06E-09	0.80218491
TCAF2	blue	-0.2383053	0.00108877	0.26419306	0.00027924	0.74506159
TBR1	blue	0.49816813	5.38E-13	-0.559705	1.21E-16	-0.9022915
TBC1D32	blue	-0.2635386	0.00028952	0.14080222	0.05591836	0.79682111
TAF13	blue	-0.5612142	9.64E-17	0.28671625	7.60E-05	0.79427019
TAF1	blue	-0.3437619	1.66E-06	0.23528394	0.00126459	0.68054634
SYNJ2BP	blue	-0.5031633	2.89E-13	0.37827404	1.10E-07	0.96124584
SYDE2	blue	-0.3851543	6.18E-08	0.22608377	0.00197214	0.84552152
SYAP1	blue	-0.311682	1.57E-05	0.28780849	7.11E-05	0.85536431
SUSD2	blue	-0.3760593	1.33E-07	0.40100825	1.54E-08	0.72567603
SUCLG1	blue	-0.315248	1.24E-05	0.05050101	0.49481657	0.76257471
STRIP2	blue	-0.3288553	4.87E-06	0.28768689	7.17E-05	0.82384111
STK36	blue	-0.2649141	0.0002683	0.18011007	0.01415703	0.820666
STK32B	blue	-0.2295729	0.00166965	0.45500829	7.68E-11	0.69958681
STARD8	blue	-0.5056555	2.11E-13	0.4831016	3.29E-12	0.86792952
SS18	blue	-0.3266502	5.68E-06	0.4423877	2.89E-10	0.76892728
SPOUT1	blue	-0.5201859	3.23E-14	0.487013	2.07E-12	0.88547
SPOP	blue	-0.5217836	2.62E-14	0.27395579	0.00016104	0.80920211
SPNS1	blue	-0.4343977	6.50E-10	0.1537908	0.036613	0.87048366
SPG20	blue	-0.3427299	1.79E-06	0.56860288	3.10E-17	0.74537515
SPECC1L	blue	-0.4907081	1.33E-12	0.50123475	3.68E-13	0.85768456
SPDYE8P	blue	-0.5510835	4.36E-16	0.41414313	4.63E-09	0.79688344
SPCS1	blue	0.37676623	1.25E-07	-0.2095682	0.00419681	-0.8658393
SPC24	blue	-0.4051397	1.06E-08	0.25451506	0.00047217	0.93131192
SNX29P2	blue	-0.3761721	1.31E-07	0.28334504	9.30E-05	0.88217626
SNURF	blue	0.44328217	2.63E-10	-0.4022143	1.39E-08	-0.8864448
SNTB2	blue	-0.3382745	2.48E-06	0.08022109	0.27770925	0.68914134
SNORD48	blue	-0.2407269	0.00096436	0.26816583	0.00022376	0.28073644
SNORA58	blue	-0.3143664	1.31E-05	0.09922176	0.17903475	0.83048904
SNORA14A	blue	-0.2115005	0.0038526	0.0554389	0.45354599	0.81172691
SNHG9	blue	-0.5059139	2.05E-13	0.42880606	1.13E-09	0.83466788
SNAPC1	blue	-0.242812	0.00086785	0.20967047	0.00417792	0.81693176
SMCR5	blue	-0.2931695	5.12E-05	0.13300879	0.07109329	0.82741877
SLC8B1	blue	-0.5011234	3.73E-13	0.46329011	3.13E-11	0.86689652
SLC6A16	blue	-0.3633285	3.71E-07	0.36001966	4.82E-07	0.78471616
SLC5A3	blue	-0.5333467	5.45E-15	0.47420273	9.21E-12	0.76307548
SLC44A2	blue	-0.5360754	3.73E-15	0.54791782	6.92E-16	0.81101388
SLC35E2B	blue	0.34036213	2.13E-06	-0.2442682	0.00080581	-0.8158127
SLC30A4	blue	-0.2143902	0.00338517	-0.0397746	0.59089559	0.80371784
SLC27A3	blue	-0.2321333	0.00147529	0.01288355	0.8618224	0.69846279
SLC26A6	blue	-0.4322162	8.08E-10	0.35310441	8.23E-07	0.85124502
SLC25A39	blue	-0.5744142	1.24E-17	0.42559176	1.55E-09	0.91373997
SLC25A34	blue	-0.3909856	3.74E-08	0.24201603	0.00090359	0.88578165
SLC25A10	blue	-0.2846547	8.60E-05	0.07169976	0.33211567	0.81040322
SLC19A3	blue	-0.505359	2.20E-13	0.45183804	1.08E-10	0.77055871

SIX5	blue	-0.6001338	1.75E-19	0.53495104	4.36E-15	0.85904409
SIPA1	blue	-0.4160624	3.86E-09	0.57305868	1.54E-17	0.8765065
SIL1	blue	-0.2794092	0.00011734	0.01076218	0.88440015	0.82277257
SIGLEC6	blue	-0.360521	4.63E-07	0.16469314	0.02507757	0.79582988
SGK3	blue	-0.4681764	1.82E-11	0.2879583	7.05E-05	0.84993241
SGF29	blue	-0.3279015	5.21E-06	0.10750967	0.14522585	0.86431473
SFMBT2	blue	-0.5368977	3.33E-15	0.44059532	3.47E-10	0.93450456
SF3A3	blue	-0.2014705	0.00595993	0.00022125	0.99761523	0.86234762
SETDB2	blue	-0.1834047	0.01245797	0.08189714	0.26776063	0.82780039
SERTAD3	blue	-0.4146101	4.43E-09	0.54311956	1.38E-15	0.81384414
SERBP1	blue	-0.2221099	0.00237686	0.30990899	1.76E-05	0.85182458
SEMA4C	blue	-0.3359686	2.94E-06	0.08061692	0.27533746	0.80474103
SEC61A1	blue	-0.4866959	2.15E-12	0.46503781	2.58E-11	0.87943871
SDHAP1	blue	-0.4865509	2.19E-12	0.39705105	2.20E-08	0.90258001
SDHAF2	blue	-0.3555093	6.84E-07	0.08823212	0.23236719	0.77089397
SCUBE2	blue	-0.37585	1.35E-07	0.22632043	0.00195015	0.84538284
SCRIB	blue	-0.4932726	9.79E-13	0.51866285	3.95E-14	0.9515728
SCN1B	blue	0.16496101	0.02483884	-0.3872385	5.17E-08	-0.6916887
SCARNA9	blue	-0.1937832	0.00821818	0.15373525	0.03668167	0.52910359
SCAPER	blue	-0.1894822	0.00978848	-0.1311081	0.07526224	0.53433554
SCAP	blue	-0.4524393	1.01E-10	0.1699377	0.02074558	0.83495238
SC5D	blue	0.46649188	2.19E-11	-0.3415092	1.96E-06	-0.872784
RRAGD	blue	-0.2528052	0.00051701	0.00529567	0.94296699	0.53523706
RRAGA	blue	0.47133274	1.28E-11	-0.4854433	2.50E-12	-0.9335608
RPS6KB2	blue	-0.4750223	8.39E-12	0.25576377	0.00044173	0.90774631
RPPH1	blue	-0.4909018	1.30E-12	0.4150394	4.25E-09	0.82803953
RPN2	blue	-0.435079	6.07E-10	0.41808354	3.19E-09	0.8603895
RPL18A	blue	-0.3845972	6.48E-08	0.50528543	2.22E-13	0.75528557
RPL15	blue	0.46869602	1.72E-11	-0.350629	9.93E-07	-0.9003274
RPH3A	blue	-0.1969403	0.00721212	-0.1564436	0.03345734	0.22847467
RP9	blue	-0.2329402	0.00141847	0.09124546	0.21674152	0.85170375
RNY4	blue	-0.3927995	3.20E-08	0.32031747	8.79E-06	0.81630396
RNF216	blue	-0.4145561	4.45E-09	0.36363118	3.63E-07	0.92476179
RNF213	blue	-0.3431103	1.74E-06	0.24679604	0.00070771	0.89795083
RN7SK	blue	-0.6158741	1.06E-20	0.56372383	6.58E-17	0.76199825
RINL	blue	-0.3369095	2.74E-06	0.33023158	4.42E-06	0.7762704
RHBDF1	blue	-0.5726452	1.65E-17	0.71716938	1.63E-30	0.78932811
RFX4	blue	-0.4267082	1.39E-09	0.2004049	0.00623557	0.86730001
RER1	blue	-0.2567406	0.00041919	0.15517357	0.03493805	0.7840208
RELL2	blue	0.25295563	0.00051291	-0.4183265	3.12E-09	-0.7313731
RDH5	blue	-0.3649504	3.26E-07	0.38702159	5.27E-08	0.76259933
RBM6	blue	-0.3349785	3.15E-06	0.2168177	0.00303277	0.86468211
RAX2	blue	-0.5003213	4.12E-13	0.41576266	3.97E-09	0.93971636
RASSF6	blue	-0.3194457	9.33E-06	0.1565433	0.03334334	0.80458188
RASAL3	blue	-0.4565615	6.50E-11	0.49890818	4.91E-13	0.84242673
RAP1GDS1	blue	0.3833844	7.18E-08	-0.4640363	2.88E-11	-0.8682991

RAMP1	blue	-0.5314843	7.05E-15	0.56022986	1.12E-16	0.82364624
RALGPS2	blue	-0.1834053	0.01245768	0.07557262	0.30659415	0.70073179
RAD51	blue	-0.3268658	5.60E-06	0.2273936	0.00185318	0.85070168
RAB2A	blue	0.58447975	2.45E-18	-0.6538345	6.14E-24	-0.8026434
RAB11FIP3	blue	-0.3540316	7.66E-07	0.18085187	0.01375773	0.84996658
PXN	blue	-0.4288645	1.13E-09	0.47389663	9.54E-12	0.82342819
PXMP4	blue	-0.3179092	1.04E-05	0.17058538	0.02025837	0.8419122
PTPRD	blue	-0.1859339	0.01127788	-0.0437017	0.55474803	0.39116974
PTDSS1	blue	0.46122338	3.92E-11	-0.4686396	1.73E-11	-0.8994652
PSMC4	blue	-0.4492437	1.42E-10	0.18912803	0.0099289	0.67083029
PRR14	blue	-0.4519837	1.06E-10	0.37070797	2.06E-07	0.95477644
PROM2	blue	-0.2606948	0.00033841	0.17141857	0.01964618	0.8227744
PRKAR1B	blue	0.35976291	4.92E-07	-0.6120055	2.14E-20	-0.7549104
PRDM11	blue	-0.3253598	6.21E-06	0.28256099	9.74E-05	0.62810132
PPP4C	blue	-0.4726618	1.10E-11	0.27098396	0.00019084	0.85387516
PPP2R5C	blue	-0.4475212	1.70E-10	0.3112519	1.61E-05	0.91092461
PPP2R3A	blue	-0.4563266	6.67E-11	0.32989586	4.52E-06	0.89683299
PPP1R16A	blue	-0.3386238	2.42E-06	0.5177653	4.45E-14	0.88731605
PPM1F	blue	-0.213429	0.00353463	0.16809848	0.02218475	0.87623953
PPIAL4G	blue	-0.2116762	0.00382259	0.05605283	0.44855301	0.81954252
PPIAL4A	blue	0.36037911	4.68E-07	-0.2654469	0.00026048	-0.9457245
PPFIA1	blue	-0.5014208	3.60E-13	0.60791632	4.46E-20	0.73897479
PPA2	blue	-0.2461107	0.00073315	0.27048235	0.00019634	0.81748921
PP7080	blue	-0.2535727	0.00049642	0.17964699	0.01441141	0.85459782
POTEF	blue	0.19648849	0.00734901	-0.4267208	1.39E-09	-0.6291972
POMGNT1	blue	-0.3708272	2.04E-07	0.17893184	0.01481212	0.85016853
POLR1B	blue	-0.3502754	1.02E-06	0.10114581	0.1707109	0.91433122
POGLUT1	blue	-0.3325867	3.74E-06	0.05791275	0.43361703	0.78729949
PLXND1	blue	-0.3491664	1.11E-06	0.24154759	0.00092526	0.84651114
PLSCR3	blue	-0.3769346	1.23E-07	0.3971137	2.19E-08	0.76933247
PLOD1	blue	-0.4765902	7.01E-12	0.5398286	2.20E-15	0.89062201
PLIN5	blue	-0.2750461	0.00015124	0.22727304	0.00186385	0.71753638
PLEKHO1	blue	-0.3918797	3.46E-08	0.2664652	0.00024612	0.89286522
PLEKHH3	blue	-0.4710606	1.32E-11	0.36979685	2.21E-07	0.88863171
PLA2G2D	blue	-0.3662336	2.95E-07	0.30384345	2.62E-05	0.88716884
PKNOX1	blue	-0.241531	0.00092603	0.22318789	0.00226021	0.81697867
PKDCC	blue	-0.2156046	0.00320451	-0.1304865	0.07666785	0.65525547
PITRM1	blue	0.33045166	4.35E-06	-0.3501906	1.03E-06	-0.735579
PISD	blue	0.37827555	1.10E-07	-0.632517	4.58E-22	-0.7770949
PIP4K2B	blue	0.41494256	4.29E-09	-0.4056346	1.02E-08	-0.6565519
PIK3R2	blue	-0.1762957	0.01637444	0.20040027	0.00623679	0.71222726
PIGX	blue	-0.2653795	0.00026146	0.20145637	0.00596351	0.82002254
PIGU	blue	-0.3175875	1.06E-05	0.08702288	0.23885541	0.87007337
PIGQ	blue	-0.3594468	5.04E-07	0.1082523	0.14245103	0.83507547
PICK1	blue	-0.2416115	0.00092227	0.0143836	0.84592368	0.79268525
PIAS4	blue	-0.5530422	3.27E-16	0.37370496	1.61E-07	0.86931864

PI4KB	blue	-0.2754731	0.00014756	-0.0035064	0.96221854	0.86642671
PI4KAP2	blue	0.37372281	1.61E-07	-0.5008382	3.87E-13	-0.876632
PI4KAP1	blue	0.29815817	3.76E-05	-0.4838899	3.00E-12	-0.8110142
PHF21A	blue	-0.5841895	2.57E-18	0.65829657	2.38E-24	0.84641629
PGAM4	blue	0.40327274	1.26E-08	-0.300095	3.32E-05	-0.9130772
PGAM1	blue	0.3233357	7.15E-06	-0.2678014	0.00022839	-0.9216243
PFDN1	blue	0.16010092	0.02948726	-0.3034479	2.68E-05	-0.8207413
PEX11B	blue	0.46677747	2.13E-11	-0.6674963	3.21E-25	-0.874123
PDE9A	blue	-0.5417197	1.69E-15	0.4174232	3.40E-09	0.88624821
PDCD4	blue	-0.4402809	3.58E-10	0.35447768	7.40E-07	0.91541863
PCYT2	blue	-0.1389843	0.05919451	-0.1402243	0.05694318	0.75587599
PCYOX1	blue	-0.2575427	0.00040148	0.10981996	0.13672625	0.75656465
PCSK4	blue	-0.387964	4.86E-08	0.07259095	0.32612558	0.84872317
PCDHB9	blue	-0.2707232	0.00019368	0.1968786	0.00723067	0.84253243
PCDHB19P	blue	-0.3531142	8.22E-07	0.20768184	0.00455927	0.9180441
PCDHA1	blue	-0.2289678	0.00171887	0.05087632	0.49161146	0.79853026
PCDH7	blue	0.17127801	0.01974832	-0.393728	2.95E-08	-0.8117574
PBX2	blue	-0.2311723	0.00154568	-0.0057876	0.93767963	0.80637932
PBX1	blue	-0.3368049	2.76E-06	-0.0136668	0.85351393	0.67478003
PAXIP1-AS1	blue	-0.3732338	1.67E-07	0.27208543	0.00017924	0.79665681
PATE2	blue	-0.2856017	8.13E-05	0.22821465	0.00178198	0.8337034
PARP10	blue	-0.4483696	1.55E-10	0.36819098	2.52E-07	0.95033904
PAMR1	blue	-0.3493335	1.10E-06	0.31461717	1.29E-05	0.56221464
PAGR1	blue	-0.4462951	1.93E-10	0.61782803	7.40E-21	0.77825886
P3H4	blue	-0.3335585	3.49E-06	0.1172031	0.11210322	0.84495348
P3H2	blue	-0.3799971	9.56E-08	0.46570256	2.39E-11	0.84397958
P2RY11	blue	-0.4212146	2.37E-09	0.22855272	0.00175339	0.88196633
P2RY1	blue	-0.1888488	0.01004085	0.35435003	7.48E-07	0.76922508
P2RX6	blue	-0.1701393	0.02059287	-0.1739985	0.01785111	0.626149
OSBPL8	blue	-0.29172	5.60E-05	0.08956192	0.22537643	0.56337469
OSBPL2	blue	-0.5089837	1.39E-13	0.51854534	4.01E-14	0.89418587
ORAOV1	blue	-0.1527099	0.03796908	-0.0252327	0.73315626	0.80229331
OPA3	blue	-0.40948	7.14E-09	0.23849275	0.00107864	0.90714715
NUBPL	blue	-0.2644347	0.00027553	0.26950463	0.00020751	0.77897475
NT5C	blue	-0.3229641	7.33E-06	0.28889248	6.66E-05	0.72210012
NR1H3	blue	-0.4204128	2.56E-09	0.36633835	2.92E-07	0.91647158
NPRL3	blue	-0.23109	0.00155185	0.17037093	0.02041858	0.81475763
NOTCH1	blue	-0.4669454	2.09E-11	0.65279957	7.63E-24	0.88403631
NOS1AP	blue	-0.1918517	0.00889336	-0.1616505	0.02793057	0.73265824
NOP14	blue	-0.3892635	4.35E-08	0.20898965	0.00430512	0.91604031
NOL8	blue	-0.4660718	2.30E-11	0.39637788	2.33E-08	0.87782192
NME1	blue	0.5160912	5.54E-14	-0.6613458	1.23E-24	-0.8485406
NHP2	blue	0.23523747	0.00126748	-0.230657	0.00158467	-0.8460417
NFATC2IP	blue	-0.3597842	4.91E-07	0.08466491	0.25186936	0.85942246
NENF	blue	-0.3262176	5.86E-06	0.27770357	0.00012965	0.85165984
NEK8	blue	-0.2881525	6.97E-05	0.09330409	0.20650698	0.89672332

NEK6	blue	-0.5051374	2.26E-13	0.42954455	1.05E-09	0.82244237
NDUFV2	blue	0.41595693	3.90E-09	-0.5188466	3.86E-14	-0.820983
NDUFV1	blue	0.11616579	0.11533677	-0.5441777	1.19E-15	-0.6999049
NCAM1	blue	-0.3388969	2.37E-06	0.02926101	0.6925625	0.60422241
NAT9	blue	-0.3475154	1.26E-06	0.22338699	0.00223925	0.84544904
MYL12B	blue	0.43877683	4.18E-10	-0.445456	2.10E-10	-0.886107
MXD4	blue	-0.588087	1.35E-18	0.51931844	3.63E-14	0.88670892
MUC1	blue	-0.4873715	1.99E-12	0.44961386	1.36E-10	0.80058684
MTFP1	blue	-0.4397898	3.77E-10	0.29648178	4.17E-05	0.80985378
MTERF1	blue	-0.3325273	3.76E-06	0.19271874	0.00858442	0.81587576
MST1	blue	-0.3303593	4.38E-06	0.18932372	0.0098511	0.86073943
MSRB2	blue	-0.4679379	1.87E-11	0.3654804	3.13E-07	0.84766512
MSL3	blue	-0.4886791	1.70E-12	0.16173109	0.02785156	0.83934792
MRPL35	blue	-0.3661992	2.96E-07	0.15124428	0.03987529	0.71675964
MRGBP	blue	-0.3251055	6.33E-06	0.13453513	0.06788306	0.85732064
MOB3C	blue	-0.5593783	1.27E-16	0.57127572	2.04E-17	0.89797204
MLXIPL	blue	-0.3719589	1.86E-07	0.06047026	0.41355232	0.83359098
MLLT6	blue	-0.6139849	1.50E-20	0.54400913	1.22E-15	0.82262801
MKNK2	blue	-0.6344131	3.17E-22	0.56211975	8.40E-17	0.76846948
MIR2116	blue	-0.2518778	0.00054295	0.19417955	0.00808538	0.85885731
MICB	blue	-0.2762557	0.00014103	0.27251202	0.00017493	0.70407409
MFSD10	blue	-0.4474091	1.72E-10	0.29672791	4.11E-05	0.91491403
METTL2B	blue	-0.2537848	0.00049086	0.06106029	0.40900187	0.8449613
MDM2	blue	-0.2640042	0.00028217	0.13603993	0.06483487	0.55071379
MBOAT2	blue	-0.3165043	1.14E-05	0.45631117	6.68E-11	0.66506724
MAX	blue	-0.4444559	2.33E-10	0.28690043	7.51E-05	0.7632317
MATK	blue	-0.2046243	0.00520682	0.01970108	0.79010576	0.77465113
MAPKAPK3	blue	-0.5111217	1.05E-13	0.50715607	1.75E-13	0.83668254
MAP3K6	blue	-0.5752839	1.08E-17	0.43838501	4.35E-10	0.84998789
MAP2K3	blue	-0.2646682	0.00027199	0.25463512	0.00046916	0.87224658
MAP1B	blue	0.46192111	3.63E-11	-0.4385366	4.28E-10	-0.8084992
MAGT1	blue	0.10643293	0.14932173	-0.1139127	0.12261044	0.21762056
MAGED1	blue	0.57900985	5.97E-18	-0.5836849	2.79E-18	-0.8540645
MAEA	blue	0.40887685	7.55E-09	-0.3600051	4.82E-07	-0.9001648
LZIC	blue	-0.2990529	3.55E-05	0.38260066	7.68E-08	0.74976246
LYL1	blue	-0.4498722	1.33E-10	0.4870207	2.07E-12	0.86810213
LSM14B	blue	-0.1364635	0.06399737	-0.0457809	0.53605385	0.51936867
LSM14A	blue	-0.4008226	1.57E-08	0.42115695	2.38E-09	0.77077497
LSM11	blue	-0.5089465	1.39E-13	0.2644294	0.00027561	0.81759392
LSAMP	blue	-0.284831	8.51E-05	0.09482385	0.19917798	0.50259237
LRRFIP1	blue	0.0530816	0.47300367	-0.0521378	0.48091959	0.02688961
LRRC14	blue	-0.4138982	4.74E-09	0.41081806	6.31E-09	0.87157587
LRP5	blue	-0.5190618	3.75E-14	0.602481	1.16E-19	0.87290734
LRP3	blue	-0.32352	7.06E-06	0.19994819	0.00635715	0.85482903
LRCH4	blue	-0.5758341	9.93E-18	0.55847044	1.46E-16	0.82379268
LOC441455	blue	-0.3505116	1.00E-06	0.03616277	0.62505912	0.70854197

LOC148413	blue	-0.187914	0.01042381	0.05928758	0.42276233	0.65650864
LOC1001302	blue	-0.3053026	2.38E-05	0.36869995	2.42E-07	0.76271886
LMNB2	blue	-0.3340531	3.37E-06	0.22941451	0.00168241	0.8430919
LLPH	blue	-0.4998148	4.39E-13	0.36655747	2.87E-07	0.94543306
LINC01567	blue	-0.424427	1.74E-09	0.18262186	0.01284458	0.80529391
LINC00997	blue	-0.233986	0.00134781	0.22714244	0.00187547	0.8764259
LINC00294	blue	0.3979127	2.04E-08	-0.2939454	4.88E-05	-0.8633653
LIMS2	blue	-0.3997246	1.73E-08	0.27021675	0.00019932	0.81920735
LILRB1	blue	-0.4445564	2.31E-10	0.36131767	4.35E-07	0.88446694
LETMD1	blue	0.55158315	4.06E-16	-0.5089044	1.40E-13	-0.8568612
LEMD2	blue	-0.2482269	0.00065718	0.21419332	0.00341531	0.87543946
LDOC1	blue	0.17974683	0.01435623	-0.381032	8.76E-08	-0.8728875
LCAT	blue	-0.4303888	9.68E-10	0.36366389	3.62E-07	0.8589801
LAT	blue	-0.2434374	0.00084069	0.00176877	0.98093645	0.8137809
LAS1L	blue	-0.3671103	2.75E-07	0.03526348	0.63369502	0.80268005
KRTCAP2	blue	-0.5008838	3.84E-13	0.31672346	1.12E-05	0.85768938
KLHL8	blue	-0.2797967	0.0001147	0.04834143	0.51347254	0.87234438
KIAA0408	blue	0.18439624	0.01198299	-0.247746	0.00067378	0.16163538
KDM6A	blue	-0.1955006	0.00765643	0.19835337	0.00679848	0.54681034
KCTD7	blue	-0.3902015	4.01E-08	0.1529136	0.03771033	0.83572902
KCTD5	blue	-0.5102616	1.18E-13	0.37744884	1.18E-07	0.84084508
KCTD11	blue	-0.4277261	1.26E-09	0.42898591	1.11E-09	0.8281098
KCNMA1	blue	-0.4411205	3.29E-10	0.56979126	2.58E-17	0.80985429
KCNK12	blue	-0.4664882	2.19E-11	0.39204361	3.41E-08	0.82173964
KCNC2	blue	0.4419784	3.01E-10	-0.5541756	2.77E-16	-0.8321868
KAT8	blue	-0.3232793	7.18E-06	0.0829949	0.26137787	0.72744955
KAT2A	blue	-0.3322529	3.83E-06	0.19084496	0.00926441	0.77070715
JAKMIP3	blue	-0.3854748	6.01E-08	0.12538909	0.08901155	0.83795114
JAG1	blue	-0.3257517	6.05E-06	0.42296135	2.00E-09	0.73156821
ITSN1	blue	-0.5485647	6.30E-16	0.69500396	5.10E-28	0.85675468
ITPR3	blue	-0.5320564	6.51E-15	0.62425859	2.23E-21	0.79862744
IRAK1BP1	blue	-0.254334	0.00047674	0.15223362	0.03857992	0.54909379
IP6K1	blue	-0.2230307	0.0022769	-0.0498956	0.50000963	0.76230861
INTS9	blue	-0.3477638	1.23E-06	0.30025569	3.29E-05	0.89120283
INTS1	blue	-0.3363625	2.85E-06	0.05944811	0.42150527	0.884493
INIP	blue	-0.4114315	5.96E-09	0.34564079	1.44E-06	0.81493524
ILVBL	blue	-0.3570094	6.09E-07	0.21468073	0.00334113	0.86030346
ILF2	blue	0.24987765	0.00060303	0.0661699	0.37084637	-0.7976771
IL10	blue	-0.3616721	4.23E-07	0.31061168	1.68E-05	0.81700373
IKZF4	blue	-0.3315403	4.03E-06	0.0713384	0.33456455	0.76871582
IKZF2	blue	-0.326883	5.59E-06	0.20270624	0.00565395	0.87102867
IGDCC3	blue	-0.4799933	4.73E-12	0.27198036	0.00018032	0.9050037
IFT122	blue	-0.2426428	0.00087534	0.01348037	0.85548983	0.84318291
IFNAR2	blue	-0.4948514	8.08E-13	0.33100709	4.18E-06	0.89287284
IBA57	blue	-0.4365578	5.23E-10	0.18746484	0.01061239	0.83437085
HYAL1	blue	-0.4729465	1.06E-11	0.49758533	5.78E-13	0.78414175

HTATSF1P2	blue	-0.4312896	8.86E-10	0.18623895	0.01114241	0.75783626
HSCB	blue	-0.2955495	4.42E-05	0.34021589	2.16E-06	0.87770156
HNRNPU	blue	0.11044995	0.1344759	-0.0620376	0.4015301	0.06588005
HMGN5	blue	-0.364437	3.40E-07	0.60276279	1.11E-19	0.75598761
HEY2	blue	-0.5022304	3.25E-13	0.65633006	3.62E-24	0.78777297
HES6	blue	-0.3150711	1.25E-05	0.50756792	1.66E-13	0.71469983
HERC1	blue	0.46798336	1.86E-11	-0.5615674	9.13E-17	-0.8841301
HEPH	blue	-0.4473748	1.72E-10	0.60347306	9.78E-20	0.80175387
HCG4	blue	-0.4570073	6.20E-11	0.38992936	4.10E-08	0.79845136
HAUS2	blue	-0.4447531	2.26E-10	0.36333376	3.71E-07	0.89057897
HACD4	blue	-0.2827657	9.63E-05	0.29107556	5.83E-05	0.8013411
GSTTP2	blue	-0.2352672	0.00126563	0.17075737	0.02013066	0.75517147
GSK3B	blue	-0.3560914	6.54E-07	-0.0335082	0.65069347	0.65743504
GSDMD	blue	-0.5566269	1.92E-16	0.59441984	4.67E-19	0.86628466
GSDMB	blue	-0.3557036	6.74E-07	0.28244831	9.81E-05	0.76925853
GRK2	blue	-0.2900959	6.19E-05	0.17469986	0.01738846	0.85329389
GRAP	blue	-0.4980849	5.44E-13	0.48285739	3.39E-12	0.79244548
GRAMD1A	blue	-0.3717798	1.88E-07	0.33900758	2.36E-06	0.83720192
GPX8	blue	-0.3791846	1.02E-07	0.23218735	0.00147142	0.90214742
GPT	blue	-0.3683206	2.49E-07	0.31033798	1.72E-05	0.88904272
GPS1	blue	-0.2497224	0.00060795	-0.0850189	0.24988502	0.75914186
GPRC5C	blue	-0.3326797	3.71E-06	0.50271451	3.06E-13	0.83919851
GOLGA6L6	blue	-0.3459244	1.41E-06	0.32826505	5.07E-06	0.88000423
GNL3L	blue	-0.4993761	4.63E-13	0.22961601	0.00166619	0.832423
GNAS	blue	0.32590389	5.98E-06	-0.1460334	0.0473169	-0.8706023
GMEB2	blue	-0.225764	0.00200223	0.12743351	0.08388257	0.85078734
GLO1	blue	0.32370599	6.97E-06	-0.2045779	0.00521723	-0.8495272
GLIS3	blue	-0.4538822	8.66E-11	0.64709168	2.49E-23	0.75026359
GGA3	blue	-0.297828	3.83E-05	0.06397846	0.38693463	0.63026186
GGA2	blue	-0.2585957	0.00037929	0.22014997	0.00260305	0.86762931
GCN1	blue	-0.4631791	3.16E-11	0.44920947	1.42E-10	0.80114221
GCLM	blue	-0.1781823	0.01524252	0.03842797	0.60353329	0.70263989
GCC1	blue	-0.3600661	4.80E-07	0.50467294	2.39E-13	0.76519344
GALNT3	blue	-0.1932259	0.00840813	0.11195804	0.12920438	0.75906119
GAK	blue	-0.3304887	4.34E-06	-0.0014014	0.9848951	0.81132086
GADD45G	blue	-0.3811379	8.68E-08	0.4338585	6.86E-10	0.75220444
FZR1	blue	-0.2959046	4.32E-05	0.01420114	0.84785435	0.84223992
FUT8-AS1	blue	-0.242335	0.00088911	0.03399125	0.64599711	0.70108083
FUK	blue	-0.4113808	5.99E-09	0.26934211	0.00020942	0.8858543
FTO	blue	0.341195	2.01E-06	-0.4229731	2.00E-09	-0.8636613
FTCD	blue	-0.2551679	0.00045602	-0.0382504	0.60520856	0.70395538
FOXP1-IT1	blue	-0.4468692	1.82E-10	0.38355016	7.08E-08	0.93928995
FOXJ2	blue	-0.326498	5.74E-06	0.04898381	0.50788557	0.8173587
FNTB	blue	-0.3232478	7.19E-06	0.34665803	1.34E-06	0.67783864
FLAD1	blue	-0.4290298	1.11E-09	0.13500212	0.06692484	0.85939871
FKRP	blue	-0.2751	0.00015078	-0.0531639	0.47231646	0.46406965

FKBP1A	blue	0.26102876	0.00033229	-0.4465909	1.87E-10	-0.8219429
FKBP14	blue	0.12894629	0.08024228	-0.0313542	0.67180157	0.08685688
FICD	blue	-0.3171727	1.09E-05	0.07390653	0.31741106	0.7310481
FHOD3	blue	0.52254781	2.36E-14	-0.5982639	2.42E-19	-0.8272854
FGD5	blue	-0.4164883	3.71E-09	0.52419216	1.90E-14	0.7432081
FGD3	blue	-0.28395	8.97E-05	0.49267062	1.05E-12	0.74351523
FER	blue	-0.3919681	3.44E-08	0.19325772	0.0083972	0.7942309
FCF1	blue	-0.2494291	0.00061732	0.15945084	0.03016204	0.68298942
FBXW4	blue	-0.5304742	8.09E-15	0.45754015	5.85E-11	0.86707166
FBXO5	blue	-0.2807084	0.00010871	0.29028434	6.12E-05	0.63359541
FBXL19-AS1	blue	-0.3334419	3.52E-06	0.36229469	4.03E-07	0.79248612
FBL	blue	-0.406791	9.14E-09	0.42332012	1.93E-09	0.80288742
FARP1	blue	-0.4828356	3.40E-12	0.48963258	1.52E-12	0.82332191
FANCE	blue	-0.340559	2.10E-06	0.39753585	2.11E-08	0.88953
FAM53C	blue	-0.3725549	1.77E-07	0.32461261	6.55E-06	0.7727322
FAM234B	blue	0.46613221	2.28E-11	-0.5788009	6.17E-18	-0.9172965
FAM167A	blue	-0.38959	4.22E-08	0.50719414	1.74E-13	0.78271796
FAM13C	blue	-0.1917769	0.00892048	-0.1344861	0.06798438	0.45201455
FAM13A	blue	-0.2873383	7.32E-05	0.1542359	0.0360666	0.74343081
FAM129B	blue	-0.5298071	8.87E-15	0.62127413	3.91E-21	0.88154922
FAM104B	blue	-0.2789593	0.00012048	0.16544447	0.02441289	0.86458289
FAAP100	blue	-0.2026289	0.00567269	-0.0792559	0.28355	0.66874772
EZR	blue	-0.4988822	4.93E-13	0.58864616	1.23E-18	0.87193384
EVI5	blue	-0.4818674	3.80E-12	0.30827568	1.96E-05	0.88902085
ERI3	blue	-0.2150619	0.00328414	0.03502443	0.63599901	0.79941091
ERCC6L2	blue	-0.2566309	0.00042166	0.10823009	0.14253341	0.80937994
ERCC5	blue	-0.5636287	6.67E-17	0.32813442	5.12E-06	0.85962017
ERC2-IT1	blue	-0.358508	5.42E-07	0.21366008	0.00349816	0.90535734
ENO3	blue	-0.3857997	5.85E-08	0.26714367	0.00023696	0.87754244
ENKD1	blue	-0.3358921	2.95E-06	0.31140312	1.60E-05	0.92577787
ENAH	blue	-0.4778705	6.05E-12	0.50324585	2.86E-13	0.83581997
EML3	blue	-0.5023301	3.21E-13	0.41462916	4.42E-09	0.87398503
EIF4H	blue	0.27422251	0.00015859	-0.1828915	0.01271026	-0.9208027
EIF4G3	blue	-0.2220811	0.00238006	-0.0537335	0.46757744	0.57390051
EIF3B	blue	-0.406439	9.44E-09	0.18575832	0.01135649	0.91253331
EIF2AK4	blue	-0.4017831	1.44E-08	0.29010757	6.19E-05	0.91956613
EID2B	blue	0.15005226	0.04148445	-0.0647709	0.38106919	-0.0003905
EHD1	blue	-0.3659589	3.01E-07	0.41206314	5.62E-09	0.86137049
EGLN2	blue	-0.2737512	0.00016294	0.12733893	0.08411449	0.88353498
EFEMP2	blue	-0.4890671	1.62E-12	0.53123685	7.29E-15	0.88263478
ECHDC2	blue	-0.4990268	4.84E-13	0.5022621	3.24E-13	0.80765342
DYX1C1	blue	-0.3316645	3.99E-06	0.24760547	0.0006787	0.86651817
DYRK2	blue	-0.2124252	0.00369704	0.0764291	0.30112889	0.7236938
DYNC1H1	blue	0.3471755	1.29E-06	-0.6580789	2.49E-24	-0.7115647
DTWD2	blue	-0.3201693	8.88E-06	0.30081731	3.17E-05	0.81484967
DSEL	blue	-0.4278841	1.24E-09	0.19762156	0.00700998	0.90696702

DPP7	blue	-0.4681737	1.82E-11	0.15914294	0.03048619	0.87837464
DOPEY2	blue	-0.3102296	1.73E-05	0.10139115	0.16967054	0.8644176
DOC2A	blue	0.3218273	7.93E-06	-0.5577436	1.63E-16	-0.7793104
DNAJB12	blue	-0.3974453	2.12E-08	0.22470208	0.00210514	0.89516614
DNAAF5	blue	-0.5754408	1.06E-17	0.46268811	3.34E-11	0.82897569
DLC1	blue	-0.5616984	8.95E-17	0.37743153	1.18E-07	0.89685759
DISC1	blue	-0.4605824	4.21E-11	0.32429955	6.69E-06	0.86685383
DIO3OS	blue	-0.3822772	7.89E-08	0.41138896	5.98E-09	0.81452687
DHX37	blue	-0.1846694	0.01185497	-0.004575	0.95071775	0.81129658
DHDDS	blue	0.40206665	1.40E-08	-0.593286	5.66E-19	-0.6946184
DFFA	blue	-0.4692492	1.61E-11	0.41058019	6.45E-09	0.88358877
DENR	blue	-0.4629272	3.25E-11	0.36938859	2.29E-07	0.92178728
DENND4C	blue	-0.4602818	4.35E-11	0.35310179	8.23E-07	0.92083541
DDX27	blue	-0.576177	9.40E-18	0.38143647	8.47E-08	0.90368278
DDX24	blue	0.29754551	3.90E-05	-0.6603715	1.52E-24	-0.7934625
DCLRE1C	blue	-0.4577238	5.73E-11	0.37056997	2.08E-07	0.91519382
DCAF11	blue	-0.343292	1.72E-06	0.26218165	0.00031196	0.92321284
DBT	blue	-0.4633849	3.09E-11	0.35437555	7.46E-07	0.86980236
CUEDC1	blue	-0.5171414	4.83E-14	0.34348873	1.70E-06	0.83281807
CTSB	blue	-0.4134593	4.93E-09	0.36425531	3.45E-07	0.90350863
CTNS	blue	-0.3976474	2.09E-08	0.41328346	5.02E-09	0.84727249
CSTB	blue	-0.2776132	0.00013033	0.45727375	6.02E-11	-0.4653586
CSRNP1	blue	-0.3849217	6.31E-08	0.36833067	2.49E-07	0.82859821
CSPG4	blue	-0.5099036	1.23E-13	0.43761255	4.70E-10	0.85545599
CSNK2A1	blue	0.41229534	5.50E-09	-0.3070747	2.12E-05	-0.9357481
CSNK1G2	blue	-0.4546247	8.00E-11	0.43860825	4.25E-10	0.87364297
CSNK1E	blue	-0.3351238	3.12E-06	0.18600983	0.01124401	0.85373951
CSF2RA	blue	-0.3439291	1.64E-06	0.35346375	8.00E-07	0.86839728
CRCP	blue	-0.4033303	1.25E-08	0.30718477	2.11E-05	0.73261066
CPSF4	blue	-0.3037406	2.63E-05	0.15281081	0.0378407	0.84813972
COX6B1	blue	0.48834143	1.77E-12	-0.4721242	1.17E-11	-0.7910622
COX19	blue	-0.5331491	5.60E-15	0.42010693	2.63E-09	0.94716923
COL11A1	blue	-0.2664379	0.00024649	0.26527667	0.00026296	0.4846755
COASY	blue	-0.3043076	2.54E-05	0.23318116	0.0014019	0.87955493
CMTR1	blue	-0.2991945	3.52E-05	-0.115532	0.11734796	0.73397998
CLK3	blue	-0.5700698	2.47E-17	0.40177774	1.44E-08	0.90931599
CLCN7	blue	-0.5545377	2.62E-16	0.37625817	1.30E-07	0.90708582
CIB1	blue	-0.3972554	2.16E-08	0.39239141	3.31E-08	0.8196404
CIAO1	blue	-0.3832339	7.28E-08	0.15216237	0.03867201	0.86393955
CHST12	blue	-0.3712857	1.96E-07	0.26611133	0.00025102	0.86675732
CHMP4A	blue	-0.355607	6.79E-07	0.4061524	9.69E-09	0.83641029
CFLAR	blue	-0.5753524	1.07E-17	0.58384103	2.72E-18	0.86252253
CETN3	blue	-0.3847138	6.42E-08	0.19898494	0.00662054	0.88086081
CEP290	blue	-0.4043242	1.14E-08	0.25883405	0.00037443	0.83045748
CEP164	blue	-0.3324075	3.79E-06	0.06691414	0.36547772	0.86004205
CEP131	blue	-0.2425985	0.00087731	0.10383651	0.1595574	0.87747956

CENPB	blue	-0.4914529	1.22E-12	0.40234761	1.37E-08	0.91956366
CEBPZ	blue	-0.3110598	1.64E-05	0.32369016	6.98E-06	0.86471558
CDKN2AIP	blue	-0.3305839	4.31E-06	0.27082937	0.00019252	0.809684
CDKL3	blue	-0.322996	7.32E-06	0.37154139	1.92E-07	0.62677105
CDK11A	blue	-0.5250124	1.70E-14	0.25375123	0.00049174	0.83612082
CDK10	blue	-0.4038644	1.19E-08	0.19144327	0.00904228	0.88803464
CDC42BPA	blue	-0.3275636	5.33E-06	0.12845553	0.081409	0.48981503
CD68	blue	-0.4257531	1.53E-09	0.40000671	1.69E-08	0.88528562
CCNT1	blue	-0.4225512	2.08E-09	0.51640103	5.32E-14	0.83115356
CCND3	blue	-0.4991573	4.76E-13	0.42896355	1.11E-09	0.88393696
CCM2	blue	-0.2956778	4.39E-05	-0.0123124	0.86789111	0.73088808
CCL5	blue	-0.3020019	2.94E-05	0.25508352	0.00045808	0.82710756
CCDC94	blue	-0.3189864	9.62E-06	0.13850323	0.0600875	0.8551118
CCDC93	blue	-0.4315352	8.64E-10	0.31272688	1.46E-05	0.91090553
CCDC151	blue	-0.5856452	2.03E-18	0.49218956	1.12E-12	0.72429
CCDC137	blue	-0.3685418	2.45E-07	0.1116298	0.13033796	0.74984771
CCDC125	blue	-0.3176294	1.06E-05	0.35956429	4.99E-07	0.80384099
CCDC115	blue	0.44700135	1.79E-10	-0.4894796	1.55E-12	-0.7222928
CASP2	blue	-0.4560327	6.88E-11	0.48488875	2.67E-12	0.84644202
CAPS	blue	-0.4484305	1.54E-10	0.48707142	2.06E-12	0.82627804
CADM4	blue	-0.4794577	5.03E-12	0.34687099	1.32E-06	0.78534714
C9orf64	blue	-0.3803955	9.24E-08	0.19000478	0.0095845	0.88589235
C8orf37	blue	-0.2764124	0.00013975	0.31620469	1.16E-05	0.75260495
C5orf22	blue	0.50731476	1.71E-13	-0.438549	4.27E-10	-0.8975766
C4orf3	blue	-0.4827686	3.42E-12	0.33709269	2.71E-06	0.89139512
C2CD2	blue	-0.4556234	7.19E-11	0.56318143	7.14E-17	0.83582935
C21orf58	blue	-0.4947114	8.22E-13	0.32526482	6.26E-06	0.79778966
C20orf27	blue	-0.1531993	0.03734992	0.09664886	0.19062834	0.82127507
C11orf63	blue	-0.2014998	0.00595251	0.07818604	0.29012041	0.65159737
C11orf49	blue	0.24751091	0.00068203	-0.3044035	2.52E-05	-0.8319364
BST2	blue	-0.5244417	1.83E-14	0.44826511	1.57E-10	0.81371872
BRSK2	blue	-0.3369891	2.73E-06	0.1111501	0.13200838	0.83348222
BRPF1	blue	-0.5324181	6.20E-15	0.43739128	4.81E-10	0.86970993
BRD2	blue	-0.3065089	2.20E-05	0.11093923	0.13274786	0.77776173
BRAT1	blue	-0.2865295	7.68E-05	0.26874627	0.00021658	0.91084971
BOP1	blue	-0.288283	6.91E-05	-0.0085485	0.90806002	0.86590405
BNIPL	blue	-0.3726903	1.75E-07	0.45698444	6.21E-11	0.6232325
BMP8B	blue	-0.3240766	6.79E-06	0.13467285	0.06759931	0.76127062
BMP7	blue	-0.3814307	8.47E-08	0.47833754	5.73E-12	0.7388716
BLZF1	blue	-0.2600617	0.00035029	0.27306336	0.0001695	0.76977787
BIRC3	blue	-0.2561444	0.00043281	0.27379305	0.00016255	0.71350491
BEX3	blue	0.50036212	4.10E-13	-0.5519692	3.83E-16	-0.8989986
BCL2L12	blue	-0.522985	2.23E-14	0.54905525	5.87E-16	0.82297565
BCAS4	blue	-0.2309909	0.0015593	-0.1539014	0.03647662	0.69072868
B4GALT4	blue	-0.4113137	6.03E-09	0.40720481	8.80E-09	0.81115984
AXL	blue	-0.5126454	8.66E-14	0.59761683	2.70E-19	0.83946454

ATP5H	blue	0.56678843	4.11E-17	-0.4662335	2.26E-11	-0.8390072
ATP2B4	blue	0.28524632	8.30E-05	-0.4132884	5.01E-09	-0.7205256
ATP13A1	blue	-0.3108372	1.66E-05	0.15808101	0.03162707	0.83242176
ATG12	blue	-0.471783	1.21E-11	0.18980234	0.00966306	0.91357258
ATG10	blue	-0.3989186	1.86E-08	0.14303416	0.05210232	0.8256692
ATF6B	blue	-0.3887392	4.55E-08	0.19619139	0.00744029	0.89222863
ASUN	blue	-0.5194339	3.57E-14	0.36656422	2.87E-07	0.90754003
ASIC1	blue	-0.1255764	0.08853135	0.0093209	0.89979415	0.76472304
ASCC2	blue	-0.3840329	6.80E-08	0.38342391	7.16E-08	0.89797258
ASAP3	blue	-0.4814034	4.01E-12	0.45012031	1.29E-10	0.84133974
ARPP21	blue	-0.506894	1.81E-13	0.18997006	0.00959793	0.74135919
ARPC3	blue	0.17788685	0.01541513	0.09928639	0.17875037	-0.8108891
ARL2	blue	-0.2763885	0.00013995	-0.0421164	0.56921019	0.84341642
ARID4B	blue	-0.5558045	2.17E-16	0.41367996	4.83E-09	0.83560992
ARHGEF9	blue	0.48011297	4.67E-12	-0.5665457	4.26E-17	-0.8092976
ARHGEF18	blue	-0.2718296	0.00018187	0.18330136	0.01250841	0.80506918
ARHGAP17	blue	-0.6032419	1.02E-19	0.56480062	5.58E-17	0.84322395
ARF3	blue	0.377353	1.19E-07	-0.6439425	4.74E-23	-0.7738777
ARCN1	blue	0.21466735	0.00334314	0.04067709	0.58249372	-0.8327607
ARAP3	blue	-0.4372778	4.86E-10	0.48702727	2.07E-12	0.7773149
ARAP2	blue	-0.306533	2.20E-05	0.19577027	0.0075714	0.61203062
APMAP	blue	0.44724117	1.75E-10	-0.5016591	3.49E-13	-0.7922018
API5	blue	0.36237806	4.00E-07	-0.1604502	0.0291301	-0.8971478
APEX1	blue	0.16608592	0.02385745	-0.1296399	0.0786163	-0.8449133
ANXA2R	blue	-0.4405514	3.49E-10	0.51813611	4.24E-14	0.78174272
ANKRD35	blue	-0.5671575	3.88E-17	0.52704973	1.29E-14	0.80454332
ANKRD13D	blue	-0.4731255	1.04E-11	0.31075605	1.67E-05	0.90810977
AMT	blue	-0.2776832	0.0001298	0.17385957	0.01794401	0.7729169
ALPP	blue	-0.3037798	2.63E-05	0.26205503	0.00031414	0.81718212
ALDOA	blue	0.16416833	0.02555103	-0.3450857	1.51E-06	-0.8314481
ALDH4A1	blue	-0.4865092	2.20E-12	0.52191786	2.57E-14	0.81647373
AKIRIN1	blue	-0.3661438	2.97E-07	0.23210169	0.00147756	0.58493715
AK1	blue	-0.3403354	2.14E-06	0.33406247	3.37E-06	0.88937595
AJAP1	blue	-0.1763693	0.01632896	-0.0414854	0.57501582	0.52336548
AIRE	blue	-0.3676007	2.64E-07	0.36805659	2.55E-07	0.82663178
AHR	blue	-0.4520953	1.05E-10	0.35108313	9.60E-07	0.88975164
AGO2	blue	-0.5492342	5.72E-16	0.38823526	4.75E-08	0.92372292
ADH4	blue	-0.2948687	4.61E-05	0.1076918	0.14454154	0.82906122
ADAM22	blue	-0.3569855	6.10E-07	0.24504445	0.00077443	0.79624449
ADAM17	blue	-0.3447461	1.54E-06	0.18326082	0.01252825	0.91065978
ACOT9	blue	-0.3035849	2.66E-05	0.2518641	0.00054334	0.85110085
ACBD7	blue	-0.4012048	1.52E-08	0.50506641	2.28E-13	0.74351317
ACACB	blue	-0.5091141	1.36E-13	0.56969158	2.62E-17	0.86895238
ABTB1	blue	-0.2552643	0.00045368	0.07833767	0.28918308	0.81679339
ABL1	blue	-0.4159584	3.90E-09	0.37539999	1.40E-07	0.94581551
ABHD8	blue	-0.2954221	4.46E-05	0.11546902	0.11754931	0.78450721

ABHD17C	blue	-0.2296191	0.00166594	0.17913818	0.01469551	0.83804605
ABCB7	blue	-0.4861527	2.30E-12	0.40125618	1.51E-08	0.89781464
AADAT	blue	-0.2962687	4.23E-05	0.06203325	0.40156312	0.6987991
ZNF576	brown	-0.4100661	6.76E-09	0.05642904	0.44550877	0.05565914
XCR1	brown	-0.2467305	0.00071011	-0.1150069	0.11903487	-0.2801903
WHRN	brown	-0.3445621	1.57E-06	0.01896295	0.79779724	-0.3764086
WASH7P	brown	-0.4578638	5.65E-11	0.08396388	0.25583126	-0.0042067
VWA7	brown	-0.2890383	6.60E-05	-0.0778541	0.29217909	-0.2114265
VPS52	brown	-0.3769846	1.23E-07	-0.1048761	0.15539775	-0.3300878
VNN1	brown	-0.2809071	0.00010745	-0.0443143	0.54920754	-0.2545042
VN1R2	brown	-0.2087633	0.00434817	-0.1560835	0.03387164	-0.285128
ULK3	brown	-0.29078	5.94E-05	-0.1925055	0.00865951	-0.3780939
TTC7B	brown	-0.165599	0.0242781	-0.2217235	0.00242	-0.3626929
TRIML1	brown	-0.4335864	7.05E-10	0.07160084	0.33278489	-0.0184726
TNXB	brown	-0.2711245	0.00018932	-0.1198331	0.10422242	-0.1929866
TNFRSF9	brown	-0.2985315	3.67E-05	-0.0714161	0.33403714	-0.1563382
TNFRSF10C	brown	-0.4003287	1.64E-08	0.03034973	0.68173438	-0.0042005
TMEM80	brown	-0.4440138	2.44E-10	0.05434337	0.46253253	-0.1532827
TMEM198	brown	-0.2890116	6.61E-05	-0.1555721	0.03446756	-0.1640469
TLCD1	brown	-0.2960992	4.27E-05	0.09873817	0.18117315	0.01389061
THEG	brown	-0.353004	8.29E-07	-0.0505305	0.49456453	-0.0767159
TBX6	brown	-0.2875587	7.22E-05	-0.0690012	0.35068095	-0.2619331
TBC1D10B	brown	-0.2788727	0.00012109	-0.2593712	0.00036368	-0.3412793
SYT5	brown	-0.2033029	0.0055113	-0.1748105	0.01731646	-0.3746831
SUPT6H	brown	-0.2874984	7.25E-05	-0.1084675	0.14165433	-0.3730304
SULT2B1	brown	-0.2827141	9.65E-05	-0.0930358	0.20782067	-0.2576638
STAC2	brown	-0.194047	0.00812958	-0.2585382	0.00038047	-0.4665999
SSX5	brown	-0.3997392	1.73E-08	0.12720185	0.08445154	0.02984214
SRC	brown	-0.2902947	6.11E-05	-0.289272	6.51E-05	-0.1579088
SPACA4	brown	-0.3079905	2.00E-05	-0.0690118	0.3506065	-0.2447291
SP6	brown	-0.3148389	1.27E-05	-0.0208178	0.77850794	-0.1937148
SNX32	brown	-0.286182	7.85E-05	-0.1203976	0.10258918	-0.2985589
SMU1	brown	-0.4111131	6.14E-09	0.09176419	0.21412916	-0.0275702
SMG6	brown	-0.42021	2.61E-09	0.09175782	0.21416112	0.04336438
SLCO1B1	brown	-0.3512675	9.46E-07	-0.0093051	0.89996321	-0.0101595
SLC35G6	brown	-0.4117189	5.80E-09	-0.0114199	0.87738955	-0.0331938
SLC2A11	brown	-0.2330078	0.00141381	-0.1857027	0.01138148	-0.2754249
SLC23A1	brown	-0.3774168	1.18E-07	0.05192091	0.48274914	-0.0526298
SLC16A2	brown	-0.3903889	3.94E-08	0.0835842	0.25799488	-0.1529431
SLC13A3	brown	-0.3398858	2.21E-06	-0.0108689	0.88326216	-0.2754625
SIT1	brown	-0.2960769	4.28E-05	-0.0659505	0.37243841	-0.2844906
SIPA1L3	brown	-0.4401431	3.63E-10	0.03811784	0.60646085	-0.0457295
SIGLEC5	brown	-0.3557936	6.69E-07	0.00320065	0.9655113	-0.1268536
SIGIRR	brown	-0.3697869	2.21E-07	0.02164156	0.76998368	-0.0695474
SH3BGRL3	brown	-0.3967407	2.26E-08	0.00819414	0.91185521	-0.253215
SGCA	brown	-0.3497009	1.07E-06	-0.0188372	0.79911002	-0.1774351

SERPINB7	brown	-0.2561349	0.00043303	-0.1434028	0.05149334	-0.3334384
SEMA4G	brown	-0.3180207	1.03E-05	-0.0557214	0.45124454	-0.198084
SCAF1	brown	-0.1664277	0.02356592	-0.2043772	0.00526257	-0.4517149
RWDD2B	brown	-0.1860222	0.0112385	-0.1663279	0.02365078	-0.3631256
RTP4	brown	-0.3110174	1.64E-05	-0.0159656	0.82922382	-0.2028434
RPS6KL1	brown	-0.3287888	4.89E-06	-0.2233979	0.00223811	-0.1226705
RNF5	brown	-0.3765438	1.27E-07	0.08351968	0.25836382	-0.2406973
RGS6	brown	-0.1498511	0.04176129	-0.2395893	0.00102108	-0.3876546
REG3G	brown	-0.2277068	0.00182571	-0.1248464	0.09041432	-0.2811979
RCAN3	brown	-0.2331493	0.00140408	-0.0799135	0.27956175	-0.3186211
RASSF5	brown	-0.3878886	4.89E-08	0.01164055	0.87503937	0.01490918
RAMP2	brown	-0.4467869	1.83E-10	0.08659476	0.2411826	-0.0265008
PSORS1C1	brown	-0.2940062	4.87E-05	-0.1213437	0.09989723	-0.2427003
PSENEN	brown	-0.4611241	3.96E-11	0.1111596	0.13197512	0.00410873
PRTN3	brown	-0.3637976	3.58E-07	0.0550156	0.45700669	-0.0424382
PRPF8	brown	-0.3174893	1.07E-05	-0.2112083	0.00390296	-0.3706391
PRDM9	brown	-0.2972993	3.96E-05	-0.1382186	0.06062107	0.02313046
PPIL2	brown	-0.2924662	5.35E-05	-0.1268344	0.08536033	-0.2130986
PM20D1	brown	-0.3511286	9.56E-07	-0.0006655	0.99282692	-0.0228831
PLP2	brown	-0.3693094	2.30E-07	0.06673022	0.36679994	-0.1737997
PGLYRP4	brown	-0.3818904	8.15E-08	-0.0337499	0.64834192	-0.1049238
PCGF3	brown	-0.3140112	1.34E-05	-0.0739758	0.31695674	-0.2234632
PAX7	brown	-0.257007	0.00041323	-0.0924496	0.21071224	-0.3040226
PADI1	brown	-0.2522633	0.00053203	-0.0775244	0.29423402	-0.2675457
OR8G2	brown	-0.3732285	1.67E-07	0.00190638	0.97945371	-0.0971145
OR52N2	brown	-0.400938	1.55E-08	0.06128305	0.40729159	0.02149517
OR52E6	brown	-0.3421037	1.88E-06	0.0507459	0.49272398	-0.0567215
OR52D1	brown	-0.2573582	0.00040549	-0.0574616	0.43721357	-0.2481002
OR51D1	brown	-0.3487483	1.14E-06	0.04631265	0.5313237	-0.0978591
OR4F21	brown	-0.2477433	0.00067387	-0.105003	0.15489545	-0.30455
OR2W5	brown	-0.3886651	4.58E-08	0.00418567	0.95490717	-0.1188989
OR2T6	brown	-0.2325065	0.00144876	-0.1234128	0.09420492	-0.2616746
OR2T12	brown	-0.2574975	0.00040246	-0.1179063	0.10995181	-0.296184
OR2T11	brown	-0.3626749	3.91E-07	0.02465615	0.73903043	0.01483262
OR2A42	brown	-0.3166348	1.13E-05	-0.0519247	0.48271698	-0.0368045
OLFM2	brown	-0.2487461	0.00063969	-0.036288	0.62386074	-0.315417
ODF4	brown	-0.2679938	0.00022594	-0.1397486	0.05779843	-0.284455
OAS1	brown	-0.3103064	1.72E-05	-0.0335932	0.64986638	-0.2560144
NRG3	brown	-0.1799985	0.01421798	-0.2441069	0.00081247	-0.1736231
NR1D1	brown	-0.2487035	0.0006411	-0.1966609	0.00729651	-0.3073089
NHLH1	brown	-0.3158181	1.19E-05	-0.0325673	0.65988096	-0.2323179
NEDD4	brown	-0.364748	3.32E-07	0.10541823	0.15326109	-0.2079044
MYOZ1	brown	-0.3402805	2.15E-06	-0.0192933	0.79435269	-0.1442758
MUC6	brown	-0.2948667	4.61E-05	-0.0902193	0.2219764	-0.2783333
MS4A15	brown	-0.2456169	0.00075201	-0.1121089	0.12868581	-0.2327589
MIR877	brown	-0.3479878	1.21E-06	-0.0348338	0.63783907	-0.1438512

MIR1267	brown	-0.3951165	2.61E-08	0.02184043	0.7679299	-0.0555344
MIR1224	brown	-0.3358511	2.96E-06	-0.0563457	0.44618227	-0.2369355
MGAT5B	brown	-0.2013937	0.00597943	-0.1688256	0.02160577	-0.3557348
MESDC2	brown	-0.2285654	0.00175233	-0.1354101	0.06609684	-0.2409504
MAPK15	brown	-0.2957824	4.36E-05	-0.0464147	0.53041848	-0.1938381
MAGEA12	brown	-0.3237769	6.93E-06	-0.0293987	0.6911894	-0.1341103
LYPD3	brown	-0.4520789	1.05E-10	0.07862827	0.28739235	-0.049118
LY9	brown	-0.3741335	1.55E-07	0.02172667	0.76910457	-0.0339571
LUZP2	brown	-0.3989666	1.85E-08	-0.0393045	0.59529334	0.05327575
LRRC71	brown	-0.2769207	0.00013569	-0.0809424	0.27339743	-0.2574046
LRRC46	brown	-0.355842	6.67E-07	0.0056349	0.93932041	-0.0322425
LRP2BP	brown	-0.2381823	0.00109546	-0.0952931	0.19695369	-0.2686759
LOC650293	brown	-0.3259292	5.97E-06	-0.005	0.9461469	-0.1141382
LOC284933	brown	-0.2737136	0.0001633	-0.0664351	0.36892761	-0.1864261
LOC202181	brown	-0.2819899	0.00010078	-0.1581522	0.0315495	-0.3355465
LOC1019271	brown	-0.3873367	5.13E-08	0.00759171	0.91831213	-0.0858445
LOC1001283	brown	-0.2388235	0.00106097	-0.1041347	0.15835597	-0.2981056
LGALS12	brown	-0.2967502	4.10E-05	-0.0764673	0.30088697	-0.2547099
LENG8	brown	-0.2491737	0.0006256	-0.1705261	0.02030253	-0.3349108
KRTAP8-1	brown	-0.2662289	0.00024939	-0.0677934	0.35919759	-0.2582503
KRTAP5-10	brown	-0.253601	0.00049567	-0.0967399	0.19020901	-0.2756672
KRTAP13-1	brown	-0.2070611	0.00468455	-0.150387	0.0410271	-0.3214957
KRT84	brown	-0.2395913	0.00102098	-0.0821854	0.26607417	-0.2672803
KLK10	brown	-0.3404626	2.12E-06	-0.0058769	0.93671927	-0.1456487
KDR	brown	-0.2914784	5.69E-05	0.01079209	0.88408118	-0.1993298
KCND3	brown	-0.2658809	0.00025427	-0.1302313	0.07725101	-0.2892762
ITGA7	brown	-0.4814169	4.01E-12	0.11427576	0.12141496	-0.0242155
IRX6	brown	-0.262973	0.00029868	-0.1474409	0.04520098	-0.2623855
IRF2BP1	brown	-0.2583144	0.0003851	-0.1917722	0.00892217	-0.3773132
IRF2	brown	-0.2586676	0.00037782	0.01167115	0.87471356	-0.4087724
IL17C	brown	-0.2362525	0.00120558	-0.1359267	0.06506025	-0.2632143
IGF2BP1	brown	-0.35952	5.01E-07	-0.0002799	0.99698255	-0.1064441
IFNL2	brown	-0.2554854	0.00044835	-0.115194	0.11843163	-0.2932128
HSFY1P1	brown	-0.4080684	8.13E-09	0.07625786	0.30221643	0.07106251
HOXC10	brown	-0.3161065	1.17E-05	-0.0297796	0.68739662	-0.1651361
HOXB1	brown	-0.2811674	0.00010581	-0.0706301	0.33939765	-0.2382895
HOXA7	brown	-0.2591909	0.00036726	-0.0662227	0.37046376	-0.2582096
HOXA6	brown	-0.2764679	0.0001393	-0.1288735	0.08041441	-0.2874612
HCN4	brown	-0.2640012	0.00028221	-0.069244	0.34898455	-0.3119252
HAAO	brown	-0.271415	0.00018622	-0.1043839	0.15735695	-0.2664621
GUCA1A	brown	-0.2361062	0.00121433	-0.0968621	0.18964717	-0.0413726
GP6	brown	-0.3686793	2.42E-07	-0.1050342	0.15477235	-0.0886586
GNAT1	brown	-0.305431	2.36E-05	-0.0681228	0.35686198	-0.2426331
GDPD4	brown	-0.3664537	2.90E-07	0.06676779	0.36652963	-0.0416837
GALE	brown	-0.2650498	0.00026629	-0.109281	0.13867411	-0.2682183
FXYD2	brown	-0.2809264	0.00010732	-0.060947	0.40987332	-0.2377124

FOSL1	brown	-0.3230811	7.28E-06	-0.0847444	0.25142305	-0.220297
FIBCD1	brown	-0.4255186	1.56E-09	-0.0086667	0.9067943	-0.0204396
FGFBP3	brown	-0.2744279	0.00015673	-0.0861701	0.24350666	-0.2707041
FGF23	brown	-0.4075667	8.52E-09	0.03042501	0.68098801	-0.0026452
FFAR3	brown	-0.3568671	6.16E-07	0.00863817	0.90709966	-0.0465791
FAM83F	brown	-0.4484479	1.54E-10	0.01943364	0.79289024	0.03764096
FAM219A	brown	-0.3342604	3.32E-06	-0.17654	0.01622387	-0.2015042
FAM178B	brown	-0.3268952	5.59E-06	-0.2259513	0.00198455	-0.258412
FAM155B	brown	-0.259035	0.00037037	-0.1030649	0.16269861	-0.25802
FAM110A	brown	-0.4164665	3.72E-09	0.05000983	0.49902796	0.02008599
EPYC	brown	-0.2549859	0.00046047	-0.061129	0.40847376	-0.2434379
EPHA8	brown	-0.4406722	3.44E-10	0.05628531	0.44667041	-0.0673059
EIF4EBP2	brown	-0.4692909	1.60E-11	0.22591701	0.00198778	-0.007071
EFNA4	brown	-0.426191	1.46E-09	0.00038791	0.9958188	-0.0100777
DUOX2	brown	-0.2501969	0.00059305	-0.1167163	0.11361185	-0.3065463
DPYSL5	brown	-0.3693851	2.29E-07	0.01666066	0.82190989	-0.0945913
DPCR1	brown	-0.28653	7.68E-05	-0.0689076	0.35133596	-0.2750997
DNA2	brown	-0.4665706	2.17E-11	0.0893659	0.22639746	0.02936195
DEFB123	brown	-0.2874336	7.28E-05	-0.1069274	0.14742989	-0.1737092
DCHS2	brown	-0.2474807	0.0006831	-0.1123008	0.12802875	-0.3097088
CXCL17	brown	-0.2121355	0.00374516	-0.1639459	0.02575398	-0.3526835
CTSW	brown	-0.2492658	0.0006226	-0.1075511	0.14506995	-0.27698
COA3	brown	-0.2727481	0.00017258	-0.1631017	0.02653697	-0.2385491
CNKSR1	brown	-0.4087412	7.64E-09	0.02630941	0.72222798	-0.0653714
CLN6	brown	-0.2510218	0.00056795	-0.1050274	0.15479918	-0.3199977
CLCN1	brown	-0.2309328	0.00156369	-0.1338057	0.06940204	-0.3214521
CHRNE	brown	-0.2736504	0.00016389	-0.0670203	0.36471574	-0.2818945
CHRNA2	brown	-0.3434451	1.70E-06	0.01422623	0.84758881	-0.0284914
CHPF	brown	-0.366612	2.86E-07	-0.1647694	0.0250094	-0.1324985
CEACAM22F	brown	-0.2468416	0.00070605	-0.1145746	0.12043783	-0.3165483
CEACAM19	brown	-0.2482574	0.00065614	-0.1080692	0.14313131	-0.2691523
CD4	brown	-0.3474418	1.26E-06	0.11868993	0.10759257	-0.2858719
CD3EAP	brown	-0.2889497	6.64E-05	-0.0881264	0.23292952	-0.2367821
CD1C	brown	-0.2588079	0.00037496	-0.1016441	0.16860295	-0.2831684
CCL8	brown	-0.3284467	5.01E-06	-0.0232771	0.75314192	-0.1101854
CAMKMT	brown	-0.4341774	6.64E-10	0.37708428	1.22E-07	0.03226586
C3orf35	brown	-0.2562346	0.00043073	-0.0895933	0.22521334	-0.2656363
C3orf20	brown	-0.2795837	0.00011615	-0.0536828	0.46799812	-0.2327618
C20orf96	brown	-0.2472069	0.00069284	-0.0832253	0.2600518	-0.4081698
BCAM	brown	-0.2695855	0.00020657	-0.0907132	0.21944582	-0.2855402
BACE2-IT1	brown	-0.1883892	0.01022757	-0.1263788	0.08649813	-0.3038971
ATP1A3	brown	-0.1551498	0.03496626	-0.2890357	6.60E-05	-0.4147683
ASH1L	brown	-0.2918041	5.57E-05	-0.0754746	0.30722346	-0.2454091
ARMC6	brown	-0.3469336	1.31E-06	-0.169305	0.02123125	-0.1668384
ARHGEF40	brown	-0.3684616	2.46E-07	0.09154858	0.21521224	-0.2722973
AQP7	brown	-0.266523	0.00024533	-0.1132028	0.12497453	-0.3230428

AOC2	brown	-0.2686311	0.00021799	-0.0836321	0.25772142	-0.2556785
ANKRD53	brown	-0.295936	4.32E-05	-0.092452	0.21070021	-0.2340853
ALPPL2	brown	-0.2677552	0.00022898	-0.0363759	0.62301994	-0.2346679
ALPK3	brown	-0.2869701	7.48E-05	-0.0910789	0.21758491	-0.265
ALPI	brown	-0.2769598	0.00013538	-0.0759974	0.30387529	-0.2501882
AGRN	brown	-0.421979	2.20E-09	-0.0476487	0.51953303	-0.126065
ADAMTS17	brown	-0.2711109	0.00018946	-0.0751316	0.30943389	-0.2812339
ADAM32	brown	-0.2055345	0.00500593	-0.1787879	0.01489396	-0.2797062
ACTRT1	brown	-0.485036	2.62E-12	0.10973013	0.13704945	0.00189781
ACPT	brown	-0.4004092	1.63E-08	0.01181692	0.8731618	-0.1270057
ACE2	brown	-0.2627937	0.00030165	-0.1121023	0.12870846	-0.2310276
ZIC2	green	-0.5487759	6.11E-16	0.82747401	9.61E-48	0.2795893
ZC3H12C	green	-0.396051	2.40E-08	0.82516322	2.90E-47	0.47918969
ZAK	green	-0.3248073	6.46E-06	0.85282715	1.59E-53	0.28671848
YAP1	green	-0.3642398	3.45E-07	0.87367944	3.67E-59	0.3959342
WDR54	green	0.51604016	5.58E-14	-0.9131661	3.02E-73	-0.4200018
TPD52L1	green	-0.4911113	1.27E-12	0.81227325	1.03E-44	0.07842067
TP53BP2	green	-0.5402478	2.08E-15	0.8335785	4.79E-49	0.42380464
TM4SF18	green	-0.3283167	5.06E-06	0.68796154	2.84E-27	0.14990802
TCF12	green	-0.3188756	9.70E-06	0.86487068	1.15E-56	0.29049839
SWAP70	green	-0.2978556	3.83E-05	0.85164741	3.12E-53	0.04318863
SPAG9	green	-0.4469497	1.80E-10	0.87723458	3.19E-60	0.33869888
SOX9	green	-0.4960029	7.02E-13	0.85244348	1.98E-53	0.29389591
SNTB1	green	-0.26773	0.00022931	0.81342376	6.23E-45	0.30832786
SASH1	green	-0.381762	8.24E-08	0.88281572	5.89E-62	0.13055594
RPS6	green	-0.3242227	6.72E-06	0.77488978	2.65E-38	0.10388622
RIN2	green	-0.506303	1.95E-13	0.91040873	4.63E-72	0.44326219
RHOBTB3	green	-0.4896212	1.52E-12	0.82182642	1.39E-46	0.25774298
RFTN2	green	-0.4948854	8.04E-13	0.90344526	3.15E-69	0.38696993
RANBP3L	green	-0.3533376	8.08E-07	0.82428043	4.40E-47	0.34442936
QKI	green	-0.4405931	3.47E-10	0.77805322	8.50E-39	0.07183466
PTPRB	green	-0.3067777	2.16E-05	0.74103027	1.76E-33	0.25506616
PRRX1	green	-0.3934215	3.03E-08	0.8389405	3.11E-50	0.32313362
PRCP	green	-0.4518236	1.08E-10	0.82327245	7.07E-47	0.30217777
PLSCR4	green	-0.3367699	2.77E-06	0.81116735	1.68E-44	0.11337605
PFKFB3	green	-0.2355497	0.00124814	0.44864371	1.51E-10	-0.08997
PELI3	green	0.28085799	0.00010776	-0.8203053	2.81E-46	-0.3195356
PDK4	green	-0.4084524	7.85E-09	0.77721781	1.15E-38	0.16234044
PALLD	green	-0.5068038	1.83E-13	0.8809438	2.30E-61	0.48426833
NPL	green	-0.3077929	2.03E-05	0.7955556	1.11E-41	0.13659819
MSRB3	green	-0.4147383	4.38E-09	0.88456324	1.62E-62	0.46005054
MAPRE1	green	-0.4643667	2.78E-11	0.9253089	5.49E-79	0.36319669
LRP4	green	-0.5827294	3.27E-18	0.85981023	2.61E-55	0.25308751
LRFN2	green	0.51097028	1.07E-13	-0.8710749	2.10E-58	-0.4929534
LIN7B	green	0.49862371	5.09E-13	-0.8844533	1.76E-62	-0.5281781
LEF1	green	-0.4545799	8.04E-11	0.86541622	8.15E-57	0.42030375

KDELC2	green	-0.3296525	4.60E-06	0.85486996	4.88E-54	0.28834413
KAT2B	green	-0.4353803	5.89E-10	0.90692807	1.29E-70	0.28463349
ITGB5	green	-0.553869	2.90E-16	0.89229688	4.10E-65	0.65193163
ITGB1	green	-0.5792033	5.79E-18	0.88292856	5.42E-62	0.53688133
IDH1	green	-0.2924946	5.34E-05	0.81256166	9.11E-45	0.17775898
GNG11	green	-0.3839305	6.86E-08	0.81684202	1.36E-45	0.15937688
GEM	green	-0.2821123	0.00010006	0.60598939	6.28E-20	0.04261611
FMNL2	green	-0.3431487	1.74E-06	0.74762923	2.32E-34	0.1764024
FAM149B1	green	-0.3262209	5.85E-06	0.8247336	3.56E-47	0.32315381
EMX2	green	-0.5198941	3.36E-14	0.87827475	1.54E-60	0.30371727
ELF1	green	-0.4191609	2.88E-09	0.90478101	9.38E-70	0.39534331
DCN	green	-0.4992542	4.71E-13	0.83570723	1.64E-49	0.39772372
CPQ	green	-0.434689	6.31E-10	0.91408434	1.19E-73	0.26322895
CDK2AP1	green	-0.4553551	7.40E-11	0.82198743	1.29E-46	0.28165507
CBFB	green	-0.399825	1.72E-08	0.85141773	3.55E-53	0.16833134
BBOX1	green	-0.4017356	1.45E-08	0.81807218	7.79E-46	0.29240359
BAMBI	green	-0.369222	2.32E-07	0.85218639	2.29E-53	0.21894381
ARHGEF6	green	-0.4953466	7.60E-13	0.90660448	1.74E-70	0.53507552
ANTXR2	green	-0.4570779	6.15E-11	0.8334287	5.17E-49	0.55254263
ANTXR1	green	-0.3961043	2.39E-08	0.85816334	7.01E-55	0.47461133
AMOT	green	-0.3558725	6.65E-07	0.83458201	2.89E-49	0.21506993
ADGRV1	green	-0.4631417	3.18E-11	0.77469032	2.84E-38	0.2834032
ACTL6A	green	-0.4126249	5.33E-09	0.88144974	1.59E-61	0.29802501
ZNF394	grey	0.09463702	0.20006867	-0.0231146	0.7548104	-0.0692406
MOXD1	grey	0.16722485	0.02289801	-0.0622061	0.40024994	-0.2108274
MFF	grey	0.22176713	0.00241509	-0.0081891	0.91190915	-0.1425652
FXR1	grey	-0.2290201	0.00171456	0.51396167	7.31E-14	0.26819089
F3	grey	0.11077683	0.13331951	0.16186928	0.02771653	-0.0658911
DIO2	grey	-0.0956942	0.19506677	0.09434816	0.20145141	0.0822654
AMY1C	grey	0.25375251	0.0004917	-0.2422668	0.00089219	-0.188086
ZDHHC9	red	-0.2146807	0.00334113	0.18000553	0.01421411	0.46268185
TMEM98	red	-0.3712015	1.97E-07	0.27105905	0.00019002	0.38015936
TJAP1	red	-0.5080725	1.56E-13	0.38170183	8.28E-08	0.74092464
THBS2	red	-0.2185781	0.00279835	0.24509766	0.00077232	0.24798967
SUN2	red	-0.4556244	7.19E-11	0.39104333	3.72E-08	0.61931562
SOX2-OT	red	-0.3843058	6.64E-08	0.44303372	2.70E-10	0.44105703
SLC5A11	red	-0.3043347	2.53E-05	0.07638211	0.30142706	0.29918174
SLC45A3	red	-0.240104	0.00099505	0.0293395	0.69177979	0.0686874
SLC44A1	red	-0.3908618	3.78E-08	0.48389985	3.00E-12	0.09393773
SLAIN1	red	-0.1891036	0.00993867	0.33747708	2.63E-06	0.31832983
SGK1	red	-0.2875616	7.22E-05	0.47916652	5.21E-12	0.03882627
SEMA6A	red	-0.4050195	1.07E-08	0.32931564	4.71E-06	0.52153842
SEMA3B	red	-0.3518059	9.08E-07	0.17465759	0.01741604	0.47661677
S1PR5	red	-0.2934908	5.02E-05	0.01399585	0.85002768	0.29793267
RTKN	red	-0.5329952	5.72E-15	0.41885993	2.96E-09	0.66253699
RNF130	red	-0.5564371	1.98E-16	0.70064659	1.24E-28	0.45465338

RHOU	red	-0.4520828	1.05E-10	0.49363731	9.36E-13	0.58032682
RASGRP3	red	-0.3070517	2.13E-05	0.36235405	4.01E-07	0.48748547
QDPR	red	-0.2517139	0.00054766	0.29880271	3.61E-05	-0.0448886
PPP1R14A	red	-0.2522222	0.00053318	0.0023279	0.97491204	0.36391605
PIEZO2	red	-0.4146858	4.40E-09	0.20192338	0.00584611	0.56172948
PHLDB1	red	-0.5781676	6.84E-18	0.27810952	0.00012661	0.6167083
P2RX7	red	-0.4123516	5.47E-09	0.37436966	1.52E-07	0.12883497
NKX6-2	red	-0.4123849	5.45E-09	0.15094851	0.04026957	0.31186769
NIPAL3	red	-0.3648319	3.30E-07	-0.0670793	0.36429306	0.42816278
NINJ2	red	-0.2230813	0.00227152	0.08933105	0.22657934	0.16807582
NDE1	red	-0.5093164	1.33E-13	0.39329073	3.06E-08	0.79426545
LPAR1	red	-0.5147072	6.63E-14	0.46693261	2.09E-11	0.47991458
KCNJ2	red	-0.379263	1.02E-07	0.19776255	0.00696878	0.4670245
ICK	red	-0.2591279	0.00036851	0.47940024	5.07E-12	0.36267933
HHIP	red	-0.2062571	0.00485143	0.07311484	0.32263701	0.35984459
GLTP	red	-0.4568276	6.32E-11	0.59408855	4.94E-19	0.54070464
FRYL	red	-0.4106433	6.41E-09	0.34282766	1.78E-06	0.64422921
FOXN2	red	-0.3307246	4.27E-06	0.6075717	4.75E-20	0.48730275
FAM222A	red	-0.4614485	3.83E-11	0.26711598	0.00023733	0.37572915
FAM107B	red	-0.496152	6.89E-13	0.3592633	5.11E-07	0.69881101
ERMN	red	-0.3232979	7.17E-06	0.2924587	5.35E-05	0.30882749
CYB5R2	red	-0.2333159	0.00139271	0.08751413	0.23620445	0.46212269
CNTN2	red	-0.3402223	2.16E-06	0.1102275	0.13526724	0.28918947
CNP	red	-0.3552577	6.97E-07	0.17688394	0.01601391	0.27855043
CLDND1	red	-0.3795684	9.90E-08	0.42977876	1.03E-09	0.15326016
CLCA4	red	-0.2979089	3.81E-05	0.39255775	3.26E-08	0.10885367
CAPN3	red	-0.3443795	1.59E-06	0.1016816	0.16844499	0.15896864
ANLN	red	-0.4327745	7.64E-10	0.54617143	8.91E-16	0.32106847
AMER2	red	-0.5578481	1.60E-16	0.3438431	1.65E-06	0.41988918
ADIPOR2	red	-0.6220374	3.39E-21	0.6056163	6.71E-20	0.5655662
ZYG11B	turquoise	0.52223588	2.46E-14	-0.5827289	3.27E-18	-0.4031274
ZWILCH	turquoise	0.77271874	5.71E-38	-0.5017156	3.47E-13	-0.645883
ZUFSP	turquoise	0.6673383	3.32E-25	-0.3476556	1.24E-06	-0.5564807
ZSWIM8	turquoise	-0.68106	1.46E-26	0.39691661	2.22E-08	0.4281857
ZSWIM7	turquoise	-0.2353833	0.00125841	0.18524828	0.01158762	0.59299035
ZSWIM6	turquoise	-0.3087404	1.90E-05	0.71118148	8.12E-30	0.40171017
ZSWIM4	turquoise	-0.4107937	6.32E-09	0.09203811	0.2127588	0.66925429
ZSCAN32	turquoise	-0.4367042	5.15E-10	0.09699919	0.18901826	0.73299452
ZSCAN18	turquoise	0.3244702	6.61E-06	-0.2833039	9.32E-05	-0.8323853
ZRANB2	turquoise	0.6366223	2.05E-22	-0.4552029	7.52E-11	-0.6626864
ZP3	turquoise	-0.3847494	6.40E-08	0.20689514	0.00471858	0.68677466
ZNHIT6	turquoise	-0.4431686	2.66E-10	0.68159765	1.29E-26	0.48738168
ZNHIT3	turquoise	0.53760815	3.01E-15	-0.2329738	0.00141615	-0.8398651
ZNFX1	turquoise	-0.4541504	8.42E-11	0.35171401	9.15E-07	0.2897031
ZNF91	turquoise	0.31014841	1.74E-05	-0.256127	0.00043322	-0.575774
ZNF830	turquoise	-0.3791587	1.02E-07	0.19502484	0.00780852	0.63193451

ZNF83	turquoise	0.0877221	0.23508841	0.34794372	1.22E-06	-0.3862806
ZNF768	turquoise	-0.49501	7.92E-13	0.27012326	0.00020038	0.43574021
ZNF716	turquoise	-0.391093	3.71E-08	0.18461581	0.01187998	0.79229293
ZNF711	turquoise	0.81030973	2.43E-44	-0.3919227	3.45E-08	-0.5460249
ZNF688	turquoise	-0.4679972	1.86E-11	0.1944972	0.00798033	0.79103586
ZNF681	turquoise	0.6728389	9.69E-26	-0.3446285	1.56E-06	-0.5712325
ZNF679	turquoise	0.50800616	1.57E-13	-0.1036106	0.16047244	-0.6797494
ZNF667	turquoise	0.64079926	8.93E-23	-0.4643273	2.79E-11	-0.8017937
ZNF664	turquoise	-0.2884797	6.83E-05	0.59039565	9.20E-19	0.32238638
ZNF653	turquoise	-0.3144947	1.30E-05	-0.0474932	0.52089828	0.75436613
ZNF641	turquoise	-0.4323816	7.95E-10	0.33454617	3.25E-06	0.431951
ZNF627	turquoise	0.43613774	5.45E-10	-0.1337867	0.06944199	-0.1432598
ZNF621	turquoise	-0.6412292	8.20E-23	0.6769782	3.77E-26	0.79178537
ZNF618	turquoise	-0.3399306	2.20E-06	0.33649934	2.82E-06	0.8096567
ZNF609	turquoise	-0.6057617	6.54E-20	0.48000007	4.73E-12	0.4859475
ZNF597	turquoise	0.73707235	5.74E-33	-0.5030109	2.95E-13	-0.6427457
ZNF581	turquoise	-0.5816247	3.91E-18	0.27122354	0.00018826	0.50575931
ZNF565	turquoise	-0.4908093	1.32E-12	0.41367862	4.83E-09	0.56257609
ZNF512	turquoise	0.39672216	2.26E-08	-0.0110484	0.88134832	-0.8168885
ZNF511	turquoise	-0.3341218	3.35E-06	0.130552	0.07651877	0.76030889
ZNF480	turquoise	-0.3756809	1.37E-07	0.71536855	2.65E-30	0.67207672
ZNF467	turquoise	-0.3587065	5.34E-07	0.505594	2.13E-13	0.68190515
ZNF444	turquoise	-0.6067586	5.48E-20	0.10957278	0.13761704	0.3643467
ZNF397	turquoise	-0.4382015	4.43E-10	0.39561403	2.50E-08	0.47479751
ZNF395	turquoise	-0.570049	2.47E-17	0.75717906	1.11E-35	0.29726279
ZNF385B	turquoise	0.59327845	5.66E-19	-0.6257869	1.67E-21	-0.6240114
ZNF366	turquoise	-0.5286137	1.04E-14	0.63863846	1.38E-22	0.27268572
ZNF365	turquoise	0.63011872	7.29E-22	-0.7412837	1.63E-33	-0.6754226
ZNF331	turquoise	0.67505832	5.85E-26	-0.4380518	4.50E-10	-0.5396845
ZNF329	turquoise	0.46744493	1.97E-11	-0.2879349	7.06E-05	-0.7482977
ZNF326	turquoise	-0.5146258	6.70E-14	0.46893718	1.67E-11	0.41455215
ZNF319	turquoise	-0.4190617	2.91E-09	0.63789835	1.59E-22	0.42885834
ZNF296	turquoise	-0.4551388	7.57E-11	0.07643532	0.30108946	0.85041494
ZNF280B	turquoise	0.43119972	8.94E-10	-0.7124098	5.86E-30	-0.5340845
ZNF25	turquoise	0.73064011	3.77E-32	-0.7049599	4.13E-29	-0.6856576
ZNF234	turquoise	-0.3950154	2.63E-08	0.20067019	0.0061659	0.71613548
ZNF233	turquoise	0.79131788	5.87E-41	-0.6109197	2.61E-20	-0.5543642
ZNF226	turquoise	0.59684363	3.09E-19	-0.3371314	2.70E-06	-0.5285538
ZNF219	turquoise	-0.6072112	5.06E-20	0.41698303	3.54E-09	0.51295804
ZNF215	turquoise	0.7638951	1.19E-36	-0.6565773	3.44E-24	-0.5296438
ZNF205	turquoise	-0.4103422	6.59E-09	0.08714946	0.23817038	0.7720592
ZNF189	turquoise	0.40849712	7.82E-09	-0.3038331	2.62E-05	-0.6027493
ZNF10	turquoise	0.50043153	4.07E-13	-0.1828496	0.01273101	-0.6672958
ZMYND15	turquoise	-0.2879083	7.07E-05	0.28733846	7.32E-05	0.70450309
ZMYM4	turquoise	0.66098507	1.33E-24	-0.4490577	1.44E-10	-0.7592477
ZMIZ2	turquoise	-0.4133971	4.96E-09	0.24678749	0.00070802	0.74855316

ZIC3	turquoise	-0.3498102	1.06E-06	0.72024276	7.04E-31	0.50231679
ZFYVE19	turquoise	-0.5954887	3.89E-19	0.22926831	0.00169426	0.54545518
ZFPM2	turquoise	0.50851306	1.47E-13	-0.2955445	4.42E-05	-0.7568754
ZFP36	turquoise	-0.3157151	1.20E-05	0.4533548	9.16E-11	0.15280154
ZFHX2	turquoise	-0.5172322	4.77E-14	0.19912937	0.00658044	0.57975701
ZFAND5	turquoise	0.5635599	6.74E-17	-0.3952605	2.58E-08	-0.7802006
ZFAND1	turquoise	0.56320116	7.12E-17	-0.2788758	0.00012107	-0.7975503
ZEB2	turquoise	-0.4760921	7.42E-12	0.52262316	2.34E-14	0.39159975
ZDHHC8P1	turquoise	-0.3634035	3.69E-07	0.2973707	3.95E-05	0.68847881
ZDHHC23	turquoise	0.66595261	4.51E-25	-0.5796374	5.40E-18	-0.6257891
ZDHHC21	turquoise	0.69876119	2.00E-28	-0.3626089	3.93E-07	-0.7183885
ZDHHC17	turquoise	0.69731672	2.87E-28	-0.4001325	1.67E-08	-0.7206915
ZDHHC13	turquoise	0.72702692	1.06E-31	-0.3799288	9.61E-08	-0.3816135
ZCCHC24	turquoise	-0.7343242	1.29E-32	0.61122071	2.47E-20	0.70820213
ZCCHC17	turquoise	0.47236583	1.13E-11	-0.5995241	1.95E-19	-0.5024773
ZCCHC14	turquoise	-0.3218203	7.93E-06	0.44030489	3.57E-10	0.66232936
ZCCHC12	turquoise	0.65567447	4.16E-24	-0.6787954	2.47E-26	-0.7873201
ZC3H15	turquoise	0.60781466	4.54E-20	-0.2603377	0.00034506	-0.8338386
ZC3H12B	turquoise	0.19655876	0.00732757	-0.5680942	3.35E-17	-0.5991153
ZC3H10	turquoise	-0.4026164	1.34E-08	0.55227882	3.66E-16	0.62591437
ZBTB21	turquoise	0.51302561	8.25E-14	-0.2873058	7.33E-05	-0.4432987
ZBTB20	turquoise	-0.7108445	8.88E-30	0.73950177	2.78E-33	0.72021868
ZBTB17	turquoise	-0.43858	4.26E-10	0.11970125	0.10460685	0.48090498
ZBTB16	turquoise	-0.6225347	3.09E-21	0.17704949	0.01591369	0.43362425
ZBTB11	turquoise	0.71115536	8.18E-30	-0.3773214	1.19E-07	-0.2782121
ZBED9	turquoise	0.73313542	1.83E-32	-0.5489674	5.95E-16	-0.6518169
ZBED1	turquoise	-0.4443496	2.36E-10	0.31393482	1.35E-05	0.52877738
YY1	turquoise	-0.3035303	2.67E-05	0.53198026	6.58E-15	0.44251932
YWHAZ	turquoise	0.60954566	3.34E-20	-0.5714355	1.99E-17	-0.9210513
YWHAH	turquoise	0.64839424	1.91E-23	-0.691633	1.17E-27	-0.696962
YWHAG	turquoise	0.71237377	5.92E-30	-0.6113718	2.40E-20	-0.8007873
YWHAB	turquoise	0.70336642	6.21E-29	-0.6673594	3.31E-25	-0.7377614
YTHDF2	turquoise	0.69920191	1.79E-28	-0.4527786	9.74E-11	-0.7241122
YTHDC2	turquoise	0.63748077	1.73E-22	-0.403179	1.27E-08	-0.7964136
YPEL5	turquoise	0.62690352	1.35E-21	-0.4999985	4.29E-13	-0.8889277
YPEL2	turquoise	0.45937064	4.80E-11	-0.175747	0.01671715	-0.7154255
YIPF6	turquoise	-0.3620631	4.10E-07	0.77387279	3.80E-38	0.34570351
YIPF5	turquoise	0.65768428	2.71E-24	-0.3032401	2.72E-05	-0.1706242
YIPF4	turquoise	0.41270111	5.30E-09	0.19197798	0.00884776	-0.5199957
YIF1B	turquoise	-0.2517916	0.00054542	-0.0655302	0.37549934	0.68494063
YEATS4	turquoise	0.63252959	4.57E-22	-0.230366	0.00160708	-0.773858
YEATS2	turquoise	0.41906353	2.91E-09	-0.3270238	5.54E-06	-0.7786418
YBX1	turquoise	-0.6665642	3.94E-25	0.74584632	4.03E-34	0.54106505
YARS2	turquoise	0.59951553	1.95E-19	-0.3439111	1.64E-06	-0.8286776
YARS	turquoise	0.54254026	1.50E-15	-0.690934	1.38E-27	-0.2017956
YAE1D1	turquoise	0.35745778	5.88E-07	0.00319469	0.96557548	-0.6843756

XYLT2	turquoise	-0.3189818	9.63E-06	-0.0138333	0.85174981	0.79201528
XPC	turquoise	-0.4862421	2.27E-12	0.37410586	1.56E-07	0.23768583
XKR8	turquoise	-0.6512307	1.06E-23	0.56097067	1.00E-16	0.5462578
XKR4	turquoise	0.57204985	1.81E-17	-0.6159939	1.04E-20	-0.7103946
XK	turquoise	0.69293983	8.48E-28	-0.6894104	2.00E-27	-0.7865941
WWC3	turquoise	-0.7034683	6.05E-29	0.64847518	1.88E-23	0.66214463
WSCD1	turquoise	-0.5733468	1.47E-17	0.69160267	1.18E-27	0.68698347
WSB1	turquoise	-0.3155884	1.21E-05	0.1601612	0.02942534	0.42158314
WRNIP1	turquoise	0.41856549	3.05E-09	-0.223433	0.00223442	-0.5036168
WRB	turquoise	0.52548413	1.59E-14	-0.119154	0.10621434	-0.8537769
WNT10B	turquoise	0.43883101	4.15E-10	-0.8029175	5.58E-43	-0.2359141
WDR83	turquoise	-0.3995801	1.76E-08	0.09787757	0.18502511	0.77715213
WDR7	turquoise	0.62331792	2.66E-21	-0.679027	2.35E-26	-0.7982906
WDR61	turquoise	0.50578111	2.08E-13	-0.3404438	2.12E-06	-0.825534
WDR6	turquoise	-0.6889856	2.22E-27	0.3573754	5.92E-07	0.63651322
WDR59	turquoise	-0.5365057	3.51E-15	0.56139828	9.37E-17	0.22287558
WDR5	turquoise	-0.3778136	1.15E-07	-0.2458019	0.00074489	0.41430955
WDR47	turquoise	0.59070106	8.74E-19	-0.4127935	5.25E-09	-0.9087099
WDR45	turquoise	-0.6015372	1.37E-19	0.47076465	1.36E-11	0.6244028
WDR37	turquoise	0.62548709	1.77E-21	-0.4306917	9.40E-10	-0.6856877
WDR20	turquoise	-0.470202	1.45E-11	0.59020443	9.50E-19	0.60968514
WBP4	turquoise	0.5358527	3.85E-15	-0.1940614	0.00812477	-0.8026872
WBP1	turquoise	-0.4620116	3.60E-11	0.4882878	1.78E-12	0.73416632
WASL	turquoise	0.64946113	1.53E-23	-0.5250503	1.69E-14	-0.8024811
WASH3P	turquoise	-0.6014651	1.39E-19	0.24105876	0.00094837	0.82292341
WASH1	turquoise	-0.5725627	1.67E-17	0.3337208	3.45E-06	0.86215629
WASF2	turquoise	-0.6402059	1.01E-22	0.43519231	6.00E-10	0.48130654
WASF1	turquoise	0.72550805	1.62E-31	-0.6419428	7.10E-23	-0.8063444
WAS	turquoise	-0.5016641	3.49E-13	0.5489132	5.99E-16	0.46120472
VWA8	turquoise	0.64994549	1.38E-23	-0.1023875	0.16549386	-0.4309138
VTA1	turquoise	0.73924585	3.00E-33	-0.5894001	1.09E-18	-0.684933
VSTM2B	turquoise	0.35972686	4.93E-07	-0.5447963	1.09E-15	-0.7077339
VSTM2A	turquoise	0.31169794	1.57E-05	-0.408131	8.09E-09	0.11999779
VSNL1	turquoise	0.62606125	1.59E-21	-0.6843748	6.69E-27	-0.6939159
VRK3	turquoise	-0.4266764	1.39E-09	0.12839279	0.08155914	0.68136576
VRK2	turquoise	-0.37731	1.20E-07	0.37761612	1.17E-07	0.60380362
VRK1	turquoise	0.69585278	4.13E-28	-0.642379	6.50E-23	-0.5692127
VPS53	turquoise	0.4348567	6.20E-10	-0.7258899	1.46E-31	-0.478234
VPS51	turquoise	-0.292774	5.25E-05	-0.0563071	0.44649391	0.71597491
VPS50	turquoise	0.79944682	2.32E-42	-0.4732006	1.03E-11	-0.663665
VPS4B	turquoise	0.44895123	1.46E-10	-0.0871916	0.23794238	-0.7212714
VPS37B	turquoise	-0.5610888	9.82E-17	0.12818854	0.08204945	0.7113152
VPS36	turquoise	0.71067359	9.29E-30	-0.4352038	5.99E-10	-0.5443842
VPS35	turquoise	0.6600059	1.65E-24	-0.4576865	5.76E-11	-0.8112019
VPS33B	turquoise	0.53433119	4.76E-15	-0.7264811	1.23E-31	-0.4972475
VPS29	turquoise	0.49182445	1.17E-12	-0.0468768	0.5263284	-0.8274652

VPS13A	turquoise	0.5061487	1.99E-13	-0.5812338	4.17E-18	-0.3824494
VNN2	turquoise	-0.3449973	1.52E-06	0.65306732	7.21E-24	0.27872432
VLDLR	turquoise	0.66265112	9.30E-25	-0.4320462	8.21E-10	-0.7868568
VKORC1L1	turquoise	0.4250767	1.63E-09	-0.3103478	1.71E-05	-0.7603468
VKORC1	turquoise	-0.6704639	1.65E-25	0.25787766	0.0003943	0.71837745
VIP	turquoise	0.74874787	1.64E-34	-0.5940527	4.97E-19	-0.4559208
VGF	turquoise	0.52384025	1.99E-14	-0.721514	4.96E-31	-0.4479243
VEZF1	turquoise	-0.5703452	2.36E-17	0.63430024	3.24E-22	0.71042187
VEGFB	turquoise	-0.5305439	8.02E-15	0.67397723	7.48E-26	0.35300232
VDAC1	turquoise	0.52601828	1.48E-14	-0.3700777	2.16E-07	-0.9229685
VCPKMT	turquoise	0.72009845	7.32E-31	-0.5241147	1.92E-14	-0.6173076
VCL	turquoise	-0.3768576	1.24E-07	0.63500629	2.82E-22	0.53538935
VCAN	turquoise	-0.447237	1.75E-10	0.80556104	1.85E-43	0.62892914
VBP1	turquoise	0.49660396	6.52E-13	-0.0766591	0.2996724	-0.7466709
VAT1L	turquoise	0.65555382	4.27E-24	-0.5175377	4.58E-14	-0.7002124
VAT1	turquoise	-0.7071935	2.31E-29	0.49287606	1.03E-12	0.44820279
VASP	turquoise	-0.4881817	1.80E-12	0.39789869	2.04E-08	0.69360515
VASN	turquoise	-0.6003561	1.68E-19	0.29643872	4.18E-05	0.58848749
VASH1	turquoise	-0.6075099	4.80E-20	0.35643564	6.37E-07	0.6857966
VAPA	turquoise	0.7582773	7.72E-36	-0.6058806	6.40E-20	-0.4417981
VANGL2	turquoise	-0.5314461	7.08E-15	0.5746132	1.21E-17	0.39966376
VAMP7	turquoise	0.56474891	5.62E-17	-0.1361359	0.06464442	-0.7446225
VAMP5	turquoise	-0.610615	2.75E-20	0.71496985	2.96E-30	0.41422951
VAMP3	turquoise	-0.6533126	6.85E-24	0.76270538	1.78E-36	0.40278179
VAMP1	turquoise	0.47980124	4.84E-12	-0.6426244	6.19E-23	-0.5759919
UXT	turquoise	-0.5789587	6.02E-18	0.75970384	4.83E-36	0.37046825
UVSSA	turquoise	-0.2974982	3.91E-05	0.14136861	0.05492876	0.69267209
USP46	turquoise	-0.2290181	0.00171473	0.44990035	1.32E-10	0.35974393
USP21	turquoise	-0.4557739	7.08E-11	0.25522512	0.00045463	0.26217287
USP15	turquoise	0.63835551	1.45E-22	-0.3565948	6.29E-07	-0.6938664
USP10	turquoise	0.37625884	1.30E-07	-0.1181564	0.10919434	-0.8258844
USO1	turquoise	0.56236208	8.09E-17	-0.0626229	0.39709411	-0.7036993
USF2	turquoise	-0.5572929	1.74E-16	0.24557439	0.00075365	0.13509249
UQCRQ	turquoise	0.36475795	3.32E-07	0.10219364	0.1663005	-0.6750832
UQCRHL	turquoise	0.57387403	1.36E-17	-0.4346186	6.35E-10	-0.5631734
UQCRH	turquoise	0.58585689	1.96E-18	-0.3783752	1.09E-07	-0.8643463
UQCRFS1	turquoise	0.41436181	4.53E-09	-0.1234142	0.09420099	-0.8629821
UQCRC2	turquoise	0.63938419	1.19E-22	-0.3335073	3.50E-06	-0.8404347
UQCRBP1	turquoise	0.4723469	1.14E-11	-0.0642286	0.38507712	-0.2959654
UQCC1	turquoise	0.64362975	5.05E-23	-0.4466583	1.86E-10	-0.3720108
UPK3BL	turquoise	-0.282528	9.76E-05	0.23574189	0.00123636	0.73459724
UPF3A	turquoise	-0.3452698	1.49E-06	-0.0006243	0.99327078	0.68696867
UPF1	turquoise	-0.645604	3.38E-23	0.25830522	0.0003853	0.42239137
UNG	turquoise	-0.5159244	5.66E-14	0.8085419	5.21E-44	0.48755081
UNC93B1	turquoise	-0.429623	1.04E-09	0.42716935	1.33E-09	0.74175196
UNC79	turquoise	0.528008	1.13E-14	-0.6828542	9.59E-27	-0.5155272

UNC50	turquoise	0.30202189	2.94E-05	0.12834946	0.08166297	-0.7588443
UNC45A	turquoise	-0.3868075	5.37E-08	0.24426368	0.00080599	0.7080932
UNC119B	turquoise	-0.3649463	3.27E-07	0.61679866	8.95E-21	0.38005489
UMAD1	turquoise	0.54500471	1.05E-15	-0.2120359	0.00376183	-0.7934232
UIMC1	turquoise	-0.4357134	5.69E-10	0.5613276	9.47E-17	0.72730401
UHRF1BP1L	turquoise	0.61185087	2.20E-20	-0.3800073	9.55E-08	-0.800855
UHRF1	turquoise	-0.5577833	1.62E-16	0.68211416	1.14E-26	0.47625888
UHMK1	turquoise	0.55960231	1.23E-16	-0.299694	3.41E-05	-0.6542444
UGP2	turquoise	0.43393808	6.80E-10	-0.0211423	0.77514671	-0.8113266
UFM1	turquoise	0.47269555	1.09E-11	0.02304147	0.75556116	-0.6811672
UCHL5	turquoise	0.65657275	3.44E-24	-0.464539	2.72E-11	-0.8281458
UCHL1	turquoise	0.57932517	5.67E-18	-0.6102024	2.97E-20	-0.8756569
UBXN1	turquoise	-0.6264535	1.47E-21	0.20313015	0.00555227	0.58771866
UBR3	turquoise	0.56171992	8.93E-17	-0.345401	1.47E-06	-0.7002436
UBQLN4	turquoise	-0.5119425	9.49E-14	0.18681646	0.01088988	0.85161964
UBQLN2	turquoise	0.64093714	8.69E-23	-0.6991469	1.81E-28	-0.6877162
UBQLN1	turquoise	0.66983284	1.91E-25	-0.6058997	6.38E-20	-0.7566039
UBLCP1	turquoise	0.77884979	6.36E-39	-0.5589468	1.36E-16	-0.418831
UBL5	turquoise	0.43843287	4.33E-10	-0.0556955	0.4514554	-0.3459128
UBL3	turquoise	0.55019704	4.97E-16	-0.1834818	0.01242046	-0.6683382
UBE4A	turquoise	0.625113	1.90E-21	-0.1248799	0.09032727	-0.2925703
UBE3C	turquoise	0.46851362	1.75E-11	-0.0699713	0.34393301	-0.6159439
UBE2V2	turquoise	0.71764011	1.43E-30	-0.5914948	7.65E-19	-0.7124253
UBE2T	turquoise	0.69087939	1.40E-27	-0.6012265	1.45E-19	-0.7526864
UBE2N	turquoise	0.75322315	3.97E-35	-0.7106962	9.23E-30	-0.6777819
UBE2M	turquoise	-0.4556768	7.15E-11	0.16808021	0.02219947	0.79512176
UBE2L6	turquoise	-0.58016	4.96E-18	0.51929226	3.64E-14	0.64194718
UBE2K	turquoise	0.56970485	2.61E-17	-0.309576	1.80E-05	-0.8741552
UBE2E3	turquoise	0.4791464	5.22E-12	-0.2253003	0.00204659	-0.8978825
UBE2E2	turquoise	0.56781724	3.50E-17	-0.723305	3.01E-31	-0.6255728
UBE2D2	turquoise	0.73198081	2.56E-32	-0.6872834	3.35E-27	-0.5011236
UBB	turquoise	0.36510742	3.22E-07	0.0128058	0.86264807	-0.7521457
UBASH3B	turquoise	0.66302962	8.56E-25	-0.7415267	1.51E-33	-0.1444978
UBA6	turquoise	0.58684485	1.66E-18	-0.1102843	0.13506498	-0.1263965
UBA5	turquoise	0.60957029	3.32E-20	-0.2416671	0.00091968	-0.7935257
TYRP1	turquoise	0.62473204	2.04E-21	-0.4224311	2.11E-09	-0.4469573
TYMP	turquoise	-0.3297845	4.56E-06	0.07736726	0.29521675	0.39899621
TXNL1	turquoise	0.66921777	2.19E-25	-0.2562213	0.00043103	-0.5620576
TXNIP	turquoise	-0.5016482	3.50E-13	0.69882164	1.97E-28	0.54023242
TXNDC9	turquoise	0.78254093	1.64E-39	-0.4368057	5.10E-10	-0.4773856
TXNDC16	turquoise	0.49757196	5.79E-13	-0.3525134	8.61E-07	-0.8016456
TXN2	turquoise	-0.3501887	1.03E-06	0.06194609	0.40222613	0.77435561
TXN	turquoise	0.4001226	1.67E-08	-0.0908924	0.21853229	-0.8533354
TUSC3	turquoise	0.64326263	5.44E-23	-0.4786588	5.52E-12	-0.8824189
TUSC2	turquoise	0.43545523	5.84E-10	-0.7300449	4.47E-32	-0.650429
TUFT1	turquoise	0.64016825	1.01E-22	-0.6112492	2.46E-20	-0.4770013

TUBGCP6	turquoise	-0.4577462	5.72E-11	-0.0696848	0.34591733	0.42398429
TUBGCP5	turquoise	0.64085168	8.84E-23	-0.4617879	3.69E-11	-0.5254261
TUBG1	turquoise	0.30598619	2.28E-05	-0.5094992	1.30E-13	-0.096342
TUBE1	turquoise	0.58003347	5.06E-18	-0.127985	0.08254037	-0.5820247
TUBB6	turquoise	-0.363774	3.58E-07	0.62207785	3.36E-21	0.20601458
TUBB4B	turquoise	0.23229758	0.00146356	-0.5836215	2.82E-18	-0.6931093
TUBB3	turquoise	0.52522814	1.65E-14	-0.7908996	6.90E-41	-0.5639533
TUBB2A	turquoise	0.57880414	6.17E-18	-0.7083316	1.72E-29	-0.708827
TUBB	turquoise	0.4318493	8.38E-10	-0.3554329	6.88E-07	-0.3025755
TUBA4A	turquoise	0.60302216	1.06E-19	-0.7940523	2.01E-41	-0.286514
TUBA1B	turquoise	0.26072048	0.00033793	-0.5467131	8.24E-16	-0.5975683
TUBA1A	turquoise	0.5051725	2.25E-13	-0.6163608	9.70E-21	-0.7997411
TTYH1	turquoise	-0.6054433	6.92E-20	0.37286726	1.72E-07	0.44631294
TTLL7	turquoise	0.38471657	6.42E-08	-0.1577918	0.03194398	-0.7733189
TTC9	turquoise	0.66633896	4.14E-25	-0.5796962	5.34E-18	-0.7877026
TTC38	turquoise	-0.6810825	1.45E-26	0.61820715	6.90E-21	0.73599253
TTC37	turquoise	0.48316927	3.27E-12	-0.1812721	0.01353594	-0.7078805
TTC33	turquoise	0.76254419	1.88E-36	-0.2934981	5.02E-05	-0.6438289
TTC32	turquoise	-0.472613	1.10E-11	0.39282401	3.19E-08	0.61463106
TTC31	turquoise	-0.4989023	4.91E-13	0.55878277	1.39E-16	0.3655448
TTC19	turquoise	0.62185326	3.51E-21	-0.4319864	8.26E-10	-0.7887492
TTC17	turquoise	-0.3825796	7.69E-08	0.07759232	0.29381003	0.51395945
TTC14	turquoise	-0.3097563	1.78E-05	0.09269022	0.20952173	0.5477646
TSTD1	turquoise	0.57641911	9.05E-18	-0.3474198	1.27E-06	-0.7407133
TST	turquoise	-0.3607653	4.54E-07	0.36501342	3.25E-07	0.70002523
TSPYL5	turquoise	0.76019433	4.11E-36	-0.6451815	3.68E-23	-0.7293485
TSPYL1	turquoise	0.64998759	1.37E-23	-0.567393	3.74E-17	-0.8706221
TSPO	turquoise	-0.5866898	1.70E-18	0.64391966	4.76E-23	0.80100291
TSPAN9	turquoise	-0.3515309	9.27E-07	0.19044435	0.00941583	0.78092725
TSPAN18	turquoise	-0.3262511	5.84E-06	0.21260288	0.00366782	0.84649178
TSPAN17	turquoise	-0.3572496	5.98E-07	0.12624138	0.08684368	0.79878223
TSPAN13	turquoise	0.64752107	2.28E-23	-0.5401023	2.12E-15	-0.8678045
TSPAN1	turquoise	0.63963465	1.13E-22	-0.5417642	1.68E-15	-0.5352771
TSNAX	turquoise	0.59845977	2.34E-19	-0.28262	9.71E-05	-0.7642266
TSG101	turquoise	0.31446725	1.30E-05	0.04927564	0.50535783	-0.7986935
TSEN2	turquoise	0.55543101	2.30E-16	-0.1548634	0.03530798	-0.6258912
TSC22D2	turquoise	0.29787257	3.82E-05	-0.1232523	0.0946371	-0.2657502
TRPC1	turquoise	0.56357846	6.72E-17	-0.6205504	4.47E-21	-0.1810785
TRO	turquoise	0.55308084	3.25E-16	-0.6114121	2.39E-20	-0.4263304
TRNT1	turquoise	0.60201319	1.26E-19	-0.3129565	1.44E-05	-0.7464744
TRMT61B	turquoise	0.70868463	1.57E-29	-0.3790217	1.04E-07	-0.6905358
TRMT61A	turquoise	-0.511974	9.45E-14	-0.0355839	0.63061196	0.56408138
TRMT10C	turquoise	0.77908292	5.85E-39	-0.4480001	1.61E-10	-0.7399728
TRMT1	turquoise	-0.4737781	9.67E-12	0.09948903	0.17786088	0.87521646
TRIQK	turquoise	0.51626506	5.41E-14	-0.2848942	8.48E-05	-0.8076229
TRIP6	turquoise	-0.661423	1.21E-24	0.64945732	1.53E-23	0.51903044

TRIP13	turquoise	0.62522267	1.86E-21	-0.6578749	2.61E-24	-0.4492696
TRIP10	turquoise	-0.5765404	8.87E-18	0.55993199	1.17E-16	0.40128994
TRIOBP	turquoise	-0.6939068	6.69E-28	0.46596778	2.33E-11	0.47244984
TRIM9	turquoise	0.59432088	4.75E-19	-0.6013909	1.41E-19	-0.7169934
TRIM8	turquoise	-0.3673819	2.69E-07	0.4137039	4.82E-09	0.20325261
TRIM59	turquoise	-0.3275442	5.34E-06	0.06109997	0.40869694	0.62067503
TRIM56	turquoise	-0.6633889	7.92E-25	0.66087508	1.37E-24	0.74025774
TRIM51	turquoise	0.30469932	2.48E-05	-0.6187084	6.29E-21	-0.544784
TRIM48	turquoise	0.3143873	1.31E-05	-0.5949538	4.26E-19	-0.6140976
TRIM47	turquoise	-0.5842835	2.53E-18	0.36401208	3.52E-07	0.12657845
TRIM41	turquoise	-0.6803035	1.74E-26	0.27434618	0.00015747	0.46862223
TRIM39	turquoise	-0.4909684	1.29E-12	0.354367	7.47E-07	0.7453742
TRIM37	turquoise	0.66833352	2.66E-25	-0.5876297	1.46E-18	-0.8374747
TRIM36	turquoise	0.74844679	1.80E-34	-0.7019922	8.83E-29	-0.4916406
TRIM33	turquoise	0.58054544	4.66E-18	-0.4852269	2.56E-12	-0.6872729
TRIM23	turquoise	0.48304564	3.31E-12	-0.2231492	0.00226431	-0.7869504
TRIM2	turquoise	0.58749083	1.49E-18	-0.3336963	3.45E-06	-0.4184378
TRIL	turquoise	-0.3523463	8.72E-07	0.40312235	1.28E-08	0.45763418
TRIB2	turquoise	-0.4123742	5.46E-09	0.3393332	2.30E-06	0.74847739
TRAPPC6B	turquoise	0.50460855	2.41E-13	-0.6755328	5.25E-26	-0.2287709
TRAPPC4	turquoise	0.43948494	3.89E-10	-0.2687538	0.00021649	-0.6144417
TRAPPC13	turquoise	0.62377448	2.44E-21	-0.3298387	4.54E-06	-0.8163302
TRAPPC11	turquoise	0.68704918	3.54E-27	-0.2812669	0.00010519	-0.5697774
TRAF1	turquoise	-0.493508	9.51E-13	0.33018839	4.43E-06	0.71232309
TPST2	turquoise	-0.3908852	3.78E-08	0.45707726	6.15E-11	0.64373914
TPRG1L	turquoise	0.52462413	1.79E-14	-0.6817866	1.23E-26	-0.0855629
TPPP3	turquoise	-0.3714436	1.94E-07	0.15987024	0.02972521	0.32316402
TPM2	turquoise	-0.6683305	2.66E-25	0.60896142	3.70E-20	0.64346936
TPM1	turquoise	0.63275145	4.38E-22	-0.7280364	7.94E-32	-0.456095
TPGS2	turquoise	0.33213597	3.86E-06	-0.2576362	0.00039947	-0.7909717
TPD52L2	turquoise	-0.5123757	8.97E-14	0.29735561	3.95E-05	0.83588166
TP53INP1	turquoise	-0.4220363	2.19E-09	0.73219264	2.40E-32	0.73699399
TP53BP1	turquoise	0.34678262	1.33E-06	-0.1332143	0.07065394	-0.8124782
TOX3	turquoise	0.66044858	1.50E-24	-0.3796708	9.82E-08	-0.6725119
TOX2	turquoise	0.50063782	3.96E-13	-0.6917878	1.12E-27	-0.2362427
TOX	turquoise	0.67057113	1.62E-25	-0.6872766	3.35E-27	-0.3324993
TOR1A	turquoise	0.57409487	1.31E-17	-0.4280303	1.22E-09	-0.866517
TOPBP1	turquoise	0.70417807	5.05E-29	-0.4690223	1.65E-11	-0.4513124
TOP3B	turquoise	-0.6040409	8.86E-20	0.4163753	3.75E-09	0.56586352
TOP2B	turquoise	0.37892988	1.04E-07	0.00063924	0.99310979	-0.7865949
TOP1MT	turquoise	-0.3855699	5.97E-08	0.11116237	0.13196545	0.69290663
TOMM70	turquoise	0.68392488	7.44E-27	-0.7320889	2.48E-32	-0.7343797
TOMM5	turquoise	0.49587386	7.13E-13	-0.1008482	0.17197921	-0.6429706
TOMM34	turquoise	0.30890525	1.88E-05	-0.6091085	3.61E-20	0.01383402
TOMM20	turquoise	0.66267585	9.25E-25	-0.7279769	8.07E-32	-0.7503084
TOB1	turquoise	-0.6021871	1.23E-19	0.61188336	2.19E-20	0.52473015

TNS3	turquoise	-0.5666915	4.17E-17	0.78791074	2.18E-40	0.58077958
TNS1	turquoise	-0.6475281	2.28E-23	0.65594599	3.93E-24	0.69527866
TNRC6C	turquoise	-0.4790079	5.30E-12	0.49693452	6.26E-13	0.69558856
TNRC6B	turquoise	-0.5558972	2.14E-16	0.21886344	0.00276194	0.75400014
TNPO3	turquoise	-0.3891244	4.40E-08	-0.0639686	0.38700788	0.70588464
TNK2	turquoise	-0.6743104	6.93E-26	0.23595575	0.00122338	0.36784441
TNIP1	turquoise	-0.4228748	2.02E-09	0.31775947	1.05E-05	0.82046797
TNFSF12	turquoise	-0.4877579	1.90E-12	0.50883136	1.41E-13	0.77635666
TNFRSF21	turquoise	0.51152534	1.00E-13	-0.7235044	2.85E-31	-0.5707983
TNFRSF1B	turquoise	-0.4680191	1.85E-11	0.54512215	1.04E-15	0.69329185
TNFRSF1A	turquoise	-0.6662231	4.25E-25	0.58748481	1.49E-18	0.52467022
TNFRSF10B	turquoise	-0.5237313	2.02E-14	0.67612996	4.58E-26	0.77544242
TMX4	turquoise	0.49778368	5.64E-13	-0.386718	5.41E-08	-0.5802371
TMX3	turquoise	0.65363746	6.40E-24	-0.350963	9.68E-07	-0.8442828
TMTC1	turquoise	0.35665841	6.26E-07	-0.0130207	0.86036619	-0.7469898
TMSB10	turquoise	0.55295648	3.31E-16	-0.5625371	7.88E-17	-0.6248108
TMPRSS5	turquoise	-0.4850369	2.62E-12	0.36616481	2.96E-07	0.50574229
TMOD2	turquoise	0.57507256	1.12E-17	-0.1886593	0.01011747	-0.6935731
TMOD1	turquoise	0.59646038	3.30E-19	-0.476799	6.84E-12	-0.6238394
TMIGD3	turquoise	-0.3491297	1.11E-06	0.45209548	1.05E-10	0.24881865
TMEM97	turquoise	0.58921585	1.12E-18	-0.6156914	1.10E-20	-0.3027634
TMEM60	turquoise	0.58978029	1.02E-18	-0.2053542	0.00504517	-0.5887768
TMEM59L	turquoise	0.25450723	0.00047237	-0.7713301	9.30E-38	-0.1922197
TMEM59	turquoise	0.34755108	1.25E-06	-0.0001397	0.99849467	-0.6375327
TMEM56	turquoise	0.41593779	3.91E-09	-0.0126789	0.8639956	-0.5742247
TMEM55A	turquoise	0.51059011	1.13E-13	-0.1957119	0.00758974	-0.7816613
TMEM47	turquoise	-0.2054734	0.00501919	0.6311782	5.94E-22	0.60888629
TMEM35A	turquoise	0.69532083	4.72E-28	-0.5425174	1.51E-15	-0.7904192
TMEM30A	turquoise	0.46643667	2.21E-11	-0.4573356	5.98E-11	-0.150758
TMEM259	turquoise	-0.6096267	3.29E-20	0.027879	0.70639768	0.60394727
TMEM246	turquoise	0.68775682	2.99E-27	-0.654262	5.61E-24	-0.7083212
TMEM245	turquoise	0.54008339	2.13E-15	-0.2658833	0.00025423	-0.7289492
TMEM237	turquoise	0.61887074	6.11E-21	-0.2757779	0.00014498	-0.7149107
TMEM230	turquoise	0.40686341	9.08E-09	0.06826892	0.35582909	-0.7482699
TMEM219	turquoise	-0.7006657	1.24E-28	0.41763476	3.33E-09	0.64505112
TMEM209	turquoise	0.42754876	1.28E-09	-0.013531	0.85495327	-0.6300326
TMEM207	turquoise	-0.4834283	3.17E-12	0.30364361	2.65E-05	0.72643138
TMEM204	turquoise	-0.5077514	1.62E-13	0.64013657	1.02E-22	0.46355513
TMEM200A	turquoise	0.76232743	2.02E-36	-0.5696624	2.63E-17	-0.4838388
TMEM19	turquoise	0.46573634	2.39E-11	0.03764785	0.61090931	-0.7466293
TMEM183B	turquoise	0.45324957	9.27E-11	-0.2651928	0.00026419	-0.6271039
TMEM183A	turquoise	0.47114028	1.30E-11	-0.2428112	0.00086788	-0.6025498
TMEM179B	turquoise	-0.7096469	1.22E-29	0.52450127	1.82E-14	0.45449734
TMEM178A	turquoise	0.56811622	3.34E-17	-0.7222194	4.08E-31	-0.478607
TMEM177	turquoise	0.45501716	7.67E-11	-0.6286222	9.73E-22	-0.5929267
TMEM176A	turquoise	-0.5793895	5.62E-18	0.49931005	4.67E-13	0.64192515

TMEM17	turquoise	0.7174405	1.51E-30	-0.4326991	7.70E-10	-0.7284333
TMEM169	turquoise	0.68081396	1.55E-26	-0.6601756	1.59E-24	-0.3131343
TMEM163	turquoise	0.43324662	7.29E-10	-0.4692541	1.61E-11	-0.7579996
TMEM158	turquoise	0.37380009	1.60E-07	-0.5241219	1.91E-14	-0.2703831
TMEM155	turquoise	0.69859098	2.09E-28	-0.6816707	1.27E-26	-0.7419047
TMEM14B	turquoise	0.55694628	1.83E-16	-0.1568616	0.0329817	-0.6994203
TMEM14A	turquoise	0.61910867	5.84E-21	-0.3311979	4.13E-06	-0.8900426
TMEM132D	turquoise	0.59891472	2.16E-19	-0.8017633	8.98E-43	-0.5452304
TMEM132B	turquoise	0.42829099	1.19E-09	-0.1154973	0.11745897	-0.52861
TMEM130	turquoise	0.47157904	1.24E-11	-0.8711596	1.98E-58	-0.4974259
TMEM129	turquoise	-0.4127936	5.25E-09	0.17799457	0.01535199	0.67918029
TMEM128	turquoise	0.62246599	3.13E-21	-0.1919704	0.00885049	-0.3038681
TMEM126B	turquoise	0.44334621	2.62E-10	0.11015674	0.13551969	-0.6666553
TMEM126A	turquoise	0.44008694	3.66E-10	-0.0395084	0.59338415	-0.7693025
TMEM120A	turquoise	-0.6081424	4.29E-20	0.27270851	0.00017297	0.74353833
TMEM119	turquoise	-0.4977803	5.64E-13	0.56604455	4.61E-17	0.14504096
TMEM106B	turquoise	0.46110543	3.97E-11	-0.0244157	0.74148448	-0.7774448
TMEFF2	turquoise	0.50587454	2.06E-13	-0.6013434	1.42E-19	-0.4954266
TMEFF1	turquoise	0.77432166	3.24E-38	-0.4553094	7.44E-11	-0.5872138
TMED7	turquoise	0.28329322	9.33E-05	0.21853302	0.00280415	-0.7568952
TMED5	turquoise	0.16916037	0.02134368	0.38157573	8.37E-08	-0.5959472
TMED2	turquoise	0.52935827	9.43E-15	0.00557565	0.93995725	-0.7200638
TMCC3	turquoise	-0.4501967	1.28E-10	0.42594567	1.50E-09	0.55581489
TMCC2	turquoise	-0.4632051	3.16E-11	0.07909216	0.28454925	0.32015534
TMC6	turquoise	-0.5468343	8.10E-16	0.27140669	0.0001863	0.66622589
TMBIM1	turquoise	-0.6182334	6.87E-21	0.61381248	1.54E-20	0.55226381
TM9SF2	turquoise	0.53673245	3.40E-15	-0.0784599	0.28842907	-0.7967204
TM2D3	turquoise	0.67865851	2.55E-26	-0.7019861	8.84E-29	-0.7649203
TM2D2	turquoise	0.57988034	5.19E-18	-0.4513343	1.14E-10	-0.8559762
TM2D1	turquoise	0.5203973	3.14E-14	-0.1729172	0.01858534	-0.3193647
TLR5	turquoise	-0.4160419	3.87E-09	0.65544241	4.37E-24	0.55043001
TLN2	turquoise	0.45859274	5.22E-11	-0.884664	1.50E-62	-0.3702911
TLL2	turquoise	0.66131873	1.24E-24	-0.6369606	1.92E-22	-0.746951
TLE2	turquoise	-0.5527103	3.44E-16	0.2027033	0.00565466	0.77398372
TLE1	turquoise	-0.4341228	6.68E-10	0.54942707	5.56E-16	0.70858451
TJP2	turquoise	-0.4058471	9.97E-09	0.68036998	1.72E-26	0.08248037
TJP1	turquoise	-0.5177333	4.47E-14	0.83831136	4.31E-50	0.38490595
TIPRL	turquoise	0.59393282	5.07E-19	-0.510873	1.09E-13	-0.1116953
TIMP1	turquoise	-0.580116	4.99E-18	0.41281012	5.24E-09	0.45921679
TIMM9	turquoise	0.47803297	5.94E-12	-0.1594502	0.03016266	-0.72344
TIMM8A	turquoise	0.65419528	5.69E-24	-0.320405	8.74E-06	-0.2189478
TIMM23	turquoise	0.55000165	5.11E-16	-0.5065142	1.90E-13	-0.8167938
TIMM22	turquoise	-0.2961525	4.26E-05	0.07154303	0.33317644	0.78792878
TIMM10B	turquoise	-0.329755	4.57E-06	-0.0432309	0.5590245	0.68321686
TIAM2	turquoise	0.41506558	4.24E-09	-0.6223591	3.19E-21	-0.4130307
TIAL1	turquoise	-0.5141233	7.16E-14	0.50813586	1.54E-13	0.25003242

TIA1	turquoise	0.30621006	2.25E-05	0.12525276	0.08936229	-0.6525708
THYN1	turquoise	0.55469517	2.56E-16	-0.4118883	5.71E-09	-0.9057389
THUMPD1	turquoise	0.75589537	1.68E-35	-0.2645636	0.00027357	-0.4772633
THOP1	turquoise	-0.3816457	8.32E-08	-0.267288	0.00023506	0.59169589
THOC7	turquoise	0.50945691	1.30E-13	-0.2299137	0.0016425	-0.8581317
THOC3	turquoise	0.55634211	2.01E-16	-0.6277808	1.14E-21	-0.5728962
THOC2	turquoise	-0.4263843	1.43E-09	0.30452012	2.50E-05	0.81781759
THNSL1	turquoise	0.72549235	1.63E-31	-0.5830903	3.08E-18	-0.7380062
THEMIS	turquoise	0.61068449	2.72E-20	-0.4715143	1.25E-11	-0.6101474
THAP10	turquoise	0.56612734	4.55E-17	-0.4251848	1.61E-09	-0.652318
TGIF1	turquoise	-0.5150356	6.36E-14	0.79901061	2.77E-42	0.3612406
TGFBI	turquoise	-0.2333024	0.00139362	0.55651652	1.95E-16	0.05038889
TGFB3	turquoise	-0.668628	2.49E-25	0.46604752	2.30E-11	0.58934425
TGFB1I1	turquoise	-0.5294438	9.32E-15	0.56822816	3.28E-17	0.74450201
TFPI	turquoise	-0.4234809	1.90E-09	0.63159093	5.49E-22	0.53784835
TFG	turquoise	0.30526147	2.39E-05	-0.040487	0.58425844	-0.4225024
TFB2M	turquoise	0.68316321	8.91E-27	-0.299928	3.36E-05	-0.7543706
TEX30	turquoise	0.63663574	2.05E-22	-0.1003117	0.17428325	-0.4974463
TESK1	turquoise	-0.462382	3.45E-11	0.06054468	0.4129767	0.19389044
TERF2IP	turquoise	0.42165438	2.27E-09	-0.6569477	3.18E-24	-0.6549805
TERF1	turquoise	0.37736132	1.19E-07	-0.5835124	2.87E-18	-0.2080726
TEAD4	turquoise	-0.4388957	4.13E-10	0.48596185	2.35E-12	0.62984944
TEAD2	turquoise	-0.6386353	1.38E-22	0.73182599	2.67E-32	0.59531364
TDRD9	turquoise	0.57173339	1.90E-17	-0.4810487	4.18E-12	-0.5108113
TDO2	turquoise	0.64502539	3.80E-23	-0.4992621	4.70E-13	-0.6080056
TCTEX1D2	turquoise	0.65384163	6.13E-24	-0.5004364	4.06E-13	-0.7972449
TCP1	turquoise	0.43959386	3.84E-10	-0.0191905	0.79542371	-0.5540379
TCIRG1	turquoise	-0.6209395	4.16E-21	0.54992105	5.17E-16	0.47198711
TCF7L2	turquoise	-0.4878821	1.87E-12	0.37237388	1.79E-07	0.23326106
TCF3	turquoise	-0.6769042	3.83E-26	0.61856088	6.47E-21	0.537675
TCERG1L	turquoise	0.4813494	4.04E-12	-0.7071149	2.36E-29	-0.1408886
TCERG1	turquoise	0.76535089	7.29E-37	-0.4821738	3.67E-12	-0.3820432
TCEB1	turquoise	0.40678133	9.15E-09	0.01092413	0.88267305	-0.7187028
TCEAL7	turquoise	0.53428722	4.79E-15	-0.4242147	1.77E-09	-0.9245805
TCEAL6	turquoise	0.67225615	1.11E-25	-0.593869	5.12E-19	-0.8652745
TCEAL3	turquoise	0.60662774	5.61E-20	-0.5281434	1.11E-14	-0.5175282
TCEAL2	turquoise	0.61480623	1.29E-20	-0.7947775	1.51E-41	-0.636393
TCEAL1	turquoise	0.524741	1.76E-14	-0.2867741	7.57E-05	-0.9199025
TBX3	turquoise	-0.4969992	6.21E-13	0.69104151	1.35E-27	0.61407387
TBX2	turquoise	-0.6356404	2.49E-22	0.57277477	1.61E-17	0.64862551
TBPL1	turquoise	0.55158862	4.05E-16	-0.4204662	2.54E-09	-0.7384849
TBP	turquoise	0.47088078	1.34E-11	-0.2429026	0.00086386	-0.7666476
TBL1X	turquoise	-0.6649538	5.62E-25	0.64211408	6.86E-23	0.83267196
TBK1	turquoise	0.61926179	5.68E-21	-0.3954008	2.54E-08	-0.5813507
TBCC	turquoise	0.50194395	3.37E-13	-0.7462206	3.59E-34	-0.6242668
TBCA	turquoise	0.44312503	2.68E-10	-0.082694	0.26311671	-0.6237504

TBC1D9B	turquoise	-0.3996293	1.75E-08	0.65097294	1.12E-23	0.23056339
TBC1D9	turquoise	0.76855801	2.43E-37	-0.6246128	2.09E-21	-0.7750084
TBC1D7	turquoise	0.62120904	3.95E-21	-0.7541058	2.99E-35	-0.4424167
TBC1D2B	turquoise	-0.5821048	3.62E-18	0.69340889	7.56E-28	0.49817214
TBC1D20	turquoise	-0.4462434	1.94E-10	0.29016293	6.16E-05	0.74473067
TBC1D16	turquoise	-0.3539036	7.74E-07	0.36106786	4.44E-07	0.63056042
TBC1D14	turquoise	-0.662909	8.79E-25	0.56486865	5.52E-17	0.48759618
TBC1D13	turquoise	-0.3626036	3.93E-07	-0.1078728	0.14386382	0.70060115
TAZ	turquoise	-0.5948147	4.36E-19	0.15232351	0.038464	0.36075311
TAX1BP3	turquoise	-0.637651	1.67E-22	0.56584647	4.75E-17	0.53662326
TAX1BP1	turquoise	0.53228536	6.31E-15	-0.0834579	0.25871749	-0.3873387
TATDN1	turquoise	0.60092872	1.53E-19	-0.2389108	0.00105635	-0.3971564
TARBP1	turquoise	0.6945369	5.73E-28	-0.6485473	1.85E-23	-0.5312383
TAP1	turquoise	-0.5717511	1.89E-17	0.62724063	1.27E-21	0.56459432
TANC2	turquoise	0.59587826	3.64E-19	-0.6368511	1.96E-22	-0.6542438
TAMM41	turquoise	0.62503772	1.93E-21	-0.4529968	9.52E-11	-0.6453084
TAGLN3	turquoise	0.58397332	2.67E-18	-0.6490949	1.65E-23	-0.8735609
TAGLN	turquoise	-0.4939384	9.03E-13	0.50310036	2.92E-13	0.6350488
TAF9B	turquoise	0.49122793	1.25E-12	-0.1127091	0.1266392	-0.7587834
TAF6L	turquoise	-0.4648496	2.63E-11	0.00603882	0.93498	0.73829805
TAF4	turquoise	-0.4003134	1.64E-08	0.69855912	2.10E-28	0.36654906
TAF2	turquoise	0.46907448	1.64E-11	-0.0747678	0.31178866	-0.8427523
TAF1C	turquoise	-0.4006641	1.59E-08	0.36348759	3.67E-07	0.60217202
TADA1	turquoise	0.71050868	9.70E-30	-0.3993804	1.79E-08	-0.7866847
TACC2	turquoise	0.52516275	1.66E-14	-0.283707	9.10E-05	-0.7480061
TAC1	turquoise	0.76238749	1.98E-36	-0.6356515	2.48E-22	-0.6577296
SZRD1	turquoise	-0.6792658	2.22E-26	0.76519721	7.68E-37	0.69190878
SYTL4	turquoise	-0.5741321	1.30E-17	0.75168796	6.47E-35	0.63770401
SYT7	turquoise	-0.329778	4.56E-06	-0.0339228	0.64666197	0.70885482
SYT4	turquoise	0.61686555	8.84E-21	-0.7788066	6.47E-39	-0.5019488
SYT17	turquoise	-0.3577635	5.74E-07	0.04795812	0.51682143	0.65331605
SYT13	turquoise	0.68919207	2.11E-27	-0.7795986	4.85E-39	-0.5788693
SYT1	turquoise	0.66030969	1.54E-24	-0.6557666	4.08E-24	-0.8207084
SYNPR	turquoise	0.51994535	3.34E-14	-0.5802363	4.90E-18	-0.5875618
SYNJ1	turquoise	0.70423837	4.97E-29	-0.6766963	4.02E-26	-0.5261269
SYNGR1	turquoise	0.32850964	4.99E-06	-0.7397522	2.58E-33	-0.2536045
SYNCRIP	turquoise	0.54672113	8.23E-16	-0.2526958	0.00052001	-0.5760867
SYN2	turquoise	0.65176877	9.47E-24	-0.8200584	3.14E-46	-0.576366
SYMPK	turquoise	-0.44249	2.86E-10	0.05291974	0.47435622	0.60799577
SYK	turquoise	-0.2491835	0.00062528	0.63610044	2.27E-22	0.26168582
SYDE1	turquoise	-0.6188348	6.15E-21	0.6971259	3.01E-28	0.67180773
SYBU	turquoise	0.60177971	1.32E-19	-0.5936047	5.36E-19	-0.7910691
SVOP	turquoise	0.58575963	1.99E-18	-0.8814714	1.57E-61	-0.5378877
SVIL	turquoise	-0.4407152	3.43E-10	0.74963296	1.24E-34	0.45511084
SV2B	turquoise	0.58267616	3.30E-18	-0.7794194	5.17E-39	-0.5842758
SUV39H2	turquoise	-0.4494161	1.39E-10	0.29384313	4.92E-05	0.78670069

SUSD1	turquoise	0.65025547	1.30E-23	-0.5114708	1.01E-13	-0.8050849
SURF6	turquoise	-0.2934709	5.03E-05	-0.0602476	0.41527695	0.63170774
SURF1	turquoise	-0.4093659	7.22E-09	0.28596444	7.95E-05	0.24766392
SUN1	turquoise	0.37322529	1.67E-07	0.07987489	0.27979488	-0.7400373
SUMO3	turquoise	0.57748867	7.62E-18	-0.6740575	7.35E-26	-0.7172643
SUMO2	turquoise	0.31026944	1.72E-05	0.10586128	0.1515315	-0.7512517
SUMO1P3	turquoise	0.67576176	4.98E-26	-0.2800845	0.00011278	-0.3244937
SULF1	turquoise	0.27976978	0.00011488	-0.3153255	1.23E-05	-0.5636167
SUCLA2	turquoise	0.61586357	1.06E-20	-0.3383791	2.47E-06	-0.89012
SUB1	turquoise	0.67346611	8.40E-26	-0.4006487	1.60E-08	-0.7078002
STXBP5L	turquoise	0.75536446	1.99E-35	-0.6420219	6.99E-23	-0.7029987
STXBP5	turquoise	0.74007437	2.34E-33	-0.5813027	4.12E-18	-0.5720575
STX7	turquoise	0.46548719	2.45E-11	-0.0384033	0.60376578	-0.7578425
STX6	turquoise	0.59313723	5.80E-19	-0.5965355	3.25E-19	-0.6581581
STX5	turquoise	-0.560281	1.11E-16	0.04989503	0.50001493	0.69176659
STX12	turquoise	0.67455035	6.57E-26	-0.5067691	1.84E-13	-0.6530556
STRBP	turquoise	0.6368376	1.97E-22	-0.3624658	3.98E-07	-0.574243
STRAP	turquoise	0.49381693	9.16E-13	-0.0078462	0.9155838	-0.7647229
STRA13	turquoise	-0.4619183	3.63E-11	0.48791652	1.86E-12	0.73039618
STMN4	turquoise	0.37355055	1.63E-07	-0.77239	6.41E-38	-0.3231424
STMN3	turquoise	0.59541866	3.94E-19	-0.6438039	4.87E-23	-0.3432216
STMN2	turquoise	0.58078435	4.48E-18	-0.7817072	2.23E-39	-0.6252514
STK40	turquoise	-0.5652756	5.18E-17	0.54939865	5.58E-16	0.69749085
STK39	turquoise	0.49108734	1.27E-12	-0.5113464	1.02E-13	-0.6903901
STK38	turquoise	-0.6107401	2.69E-20	0.68236834	1.07E-26	0.74123328
STK3	turquoise	-0.439824	3.75E-10	0.72242268	3.85E-31	0.52006108
STK26	turquoise	-0.3506794	9.89E-07	0.28316102	9.40E-05	0.73220727
STK24	turquoise	0.48548222	2.49E-12	-0.1999313	0.00636168	-0.5314647
STK19	turquoise	-0.4088213	7.59E-09	0.14574956	0.0477535	0.63505962
STK11	turquoise	-0.5034151	2.80E-13	0.14127353	0.05509386	0.61063206
STH	turquoise	-0.4723413	1.14E-11	0.16149883	0.02807976	0.5281264
STEAP3	turquoise	-0.2888664	6.67E-05	0.53362298	5.25E-15	0.42299316
STEAP2	turquoise	0.67382289	7.75E-26	-0.5876548	1.45E-18	-0.7662623
STAU2	turquoise	0.659878	1.70E-24	-0.4621955	3.53E-11	-0.7323078
STAT5A	turquoise	-0.5615811	9.12E-17	0.55427506	2.73E-16	0.68735553
STAT4	turquoise	0.66776025	3.02E-25	-0.746407	3.39E-34	-0.7408211
STAT3	turquoise	-0.5495409	5.47E-16	0.66456495	6.12E-25	0.15453908
STAT2	turquoise	-0.3429223	1.77E-06	0.18423208	0.01206051	0.786078
STAT1	turquoise	0.32118149	8.29E-06	-0.038037	0.60722457	-0.3478833
STARD3NL	turquoise	0.35516728	7.02E-07	0.04924056	0.50566136	-0.7950734
STARD3	turquoise	-0.6554621	4.35E-24	0.3707596	2.05E-07	0.71259032
STARD10	turquoise	-0.4739468	9.48E-12	0.12579371	0.08797703	0.03932811
STAMBPL1	turquoise	0.62395845	2.36E-21	-0.5265609	1.38E-14	-0.8581469
STAM	turquoise	0.65797523	2.55E-24	-0.4460592	1.98E-10	-0.8505175
STAB1	turquoise	-0.4643644	2.78E-11	0.38936243	4.31E-08	0.30008568
ST8SIA5	turquoise	0.41544101	4.10E-09	-0.7100802	1.09E-29	-0.0199103

ST8SIA3	turquoise	0.53302138	5.70E-15	-0.7306884	3.71E-32	-0.3751703
ST6GALNAC	turquoise	0.72547072	1.64E-31	-0.7231609	3.14E-31	-0.6507609
ST5	turquoise	-0.6528234	7.59E-24	0.495973	7.04E-13	0.41095122
ST3GAL5	turquoise	0.4819329	3.77E-12	-0.5469921	7.92E-16	-0.7470136
SSX2IP	turquoise	0.70994487	1.13E-29	-0.6496904	1.46E-23	-0.5136925
SSTR1	turquoise	0.82334006	6.85E-47	-0.4524705	1.01E-10	-0.4950309
SST	turquoise	0.55761105	1.66E-16	-0.6352483	2.69E-22	-0.7095888
SSPO	turquoise	-0.2998289	3.38E-05	0.17975997	0.01434898	0.76453837
SSH3	turquoise	-0.4904709	1.37E-12	0.34387638	1.65E-06	0.7794043
SSFA2	turquoise	-0.3444142	1.58E-06	0.69395788	6.60E-28	0.07938112
SSBP1	turquoise	0.42727082	1.32E-09	-0.0666681	0.36724704	-0.77216
SSB	turquoise	0.46388322	2.93E-11	-0.0896791	0.22476785	-0.831377
SS18L1	turquoise	0.61465928	1.32E-20	-0.6172564	8.23E-21	-0.3706732
SRSF8	turquoise	0.5810323	4.31E-18	-0.3025405	2.84E-05	-0.7875891
SRSF7	turquoise	0.63277077	4.36E-22	-0.5411403	1.83E-15	-0.5881742
SRSF3	turquoise	0.59922908	2.05E-19	-0.3307224	4.27E-06	-0.8389881
SRSF2	turquoise	0.38078619	8.94E-08	-0.112016	0.12900503	-0.7701223
SRSF12	turquoise	0.67329263	8.74E-26	-0.540227	2.08E-15	-0.7405975
SRRM4	turquoise	0.40391293	1.19E-08	-0.7802727	3.79E-39	-0.3578199
SRRM2	turquoise	-0.5482134	6.63E-16	0.31430377	1.32E-05	0.58021966
SRPK2	turquoise	0.80159374	9.63E-43	-0.4388312	4.15E-10	-0.684654
SRPK1	turquoise	0.57364679	1.41E-17	-0.3454392	1.47E-06	-0.5331983
SRP9	turquoise	0.29823576	3.74E-05	0.13287391	0.07138281	-0.6744163
SRP72	turquoise	0.70312666	6.61E-29	-0.5532901	3.15E-16	-0.5209106
SRP54	turquoise	0.4131427	5.08E-09	-0.0082582	0.91116887	-0.8115224
SRGAP3	turquoise	0.49998603	4.30E-13	-0.6924286	9.62E-28	-0.2977354
SRGAP1	turquoise	-0.568606	3.10E-17	0.61365893	1.59E-20	0.72135329
SREBF1	turquoise	-0.324117	6.77E-06	0.18571498	0.01137597	0.5095708
SRD5A1	turquoise	0.64190484	7.15E-23	-0.747333	2.54E-34	-0.6191491
SQLE	turquoise	0.46256575	3.38E-11	-0.1842263	0.01206326	-0.7959908
SPTSSB	turquoise	0.73787254	4.53E-33	-0.4880963	1.82E-12	-0.5894325
SPTLC1	turquoise	0.28013972	0.00011241	0.26335138	0.00029252	-0.6373657
SPTBN1	turquoise	-0.5173552	4.69E-14	0.31722674	1.08E-05	0.70622582
SPRYD7	turquoise	0.69699734	3.11E-28	-0.7423235	1.19E-33	-0.6944266
SPR	turquoise	-0.5385868	2.63E-15	0.52749332	1.21E-14	0.42952169
SPP1	turquoise	-0.3035412	2.67E-05	0.41183809	5.74E-09	-0.1657787
SPOCK1	turquoise	0.257372	0.00040519	-0.5106201	1.12E-13	-0.6068203
SPNS2	turquoise	-0.4307006	9.39E-10	0.07564818	0.30610935	0.60840434
SPINT2	turquoise	0.62160388	3.67E-21	-0.8200498	3.16E-46	-0.3746232
SPIDR	turquoise	0.4214365	2.32E-09	-0.4528952	9.62E-11	-0.652182
SPHKAP	turquoise	0.56098317	9.98E-17	-0.6016883	1.34E-19	-0.8269851
SPHK2	turquoise	-0.5132616	8.00E-14	0.22059244	0.00255034	0.7088763
SPEN	turquoise	-0.6075688	4.75E-20	0.48550349	2.48E-12	0.57915755
SPATS2L	turquoise	0.27569534	0.00014568	0.03076746	0.67759681	-0.7530371
SPATA13	turquoise	-0.3342901	3.31E-06	0.45044666	1.25E-10	0.36271373
SPAST	turquoise	0.55996706	1.16E-16	-0.1337351	0.06955071	-0.7259535

SPARCL1	turquoise	0.2786622	0.00012259	0.16410667	0.02560715	-0.7343675
SPAG16	turquoise	-0.3250113	6.37E-06	0.43613441	5.46E-10	0.60665096
SPACA6	turquoise	-0.4711574	1.30E-11	0.35408208	7.63E-07	0.78804624
SP110	turquoise	-0.4072583	8.76E-09	0.6334645	3.81E-22	0.49049615
SP100	turquoise	-0.3798215	9.70E-08	0.48922053	1.59E-12	0.42845594
SP1	turquoise	-0.6289778	9.08E-22	0.44812794	1.59E-10	0.76702594
SOX8	turquoise	-0.594235	4.82E-19	0.35290493	8.35E-07	0.68459052
SOX4	turquoise	-0.3122581	1.51E-05	0.49114096	1.27E-12	0.60973907
SOX18	turquoise	-0.3513437	9.41E-07	0.42043928	2.55E-09	0.53372642
SOX13	turquoise	-0.6794606	2.12E-26	0.70558568	3.51E-29	0.7036711
SOWAHC	turquoise	-0.4360371	5.51E-10	0.73244818	2.23E-32	0.63491566
SOWAHA	turquoise	0.38896172	4.46E-08	-0.180427	0.01398521	-0.6177768
SORBS2	turquoise	0.66271901	9.16E-25	-0.6537612	6.23E-24	-0.5390578
SORBS1	turquoise	-0.5733398	1.48E-17	0.58431232	2.52E-18	0.80238181
SOCS5	turquoise	0.53659064	3.47E-15	-0.0609216	0.41006853	-0.7047492
SNX6	turquoise	-0.5272202	1.26E-14	0.33630315	2.87E-06	0.83499964
SNX5	turquoise	-0.3447848	1.54E-06	0.78076711	3.16E-39	0.43799104
SNX4	turquoise	0.68021654	1.78E-26	-0.2913068	5.75E-05	-0.609038
SNX30	turquoise	0.52201027	2.54E-14	-0.2094652	0.00421592	-0.7702859
SNX3	turquoise	0.26688906	0.00024036	0.24861517	0.00064406	-0.6711918
SNX22	turquoise	-0.5189956	3.78E-14	0.29473476	4.65E-05	0.74383066
SNX14	turquoise	0.7887252	1.60E-40	-0.457079	6.15E-11	-0.4853265
SNX10	turquoise	0.75217377	5.54E-35	-0.646557	2.78E-23	-0.7503865
SNU13	turquoise	0.48102098	4.20E-12	-0.8172682	1.12E-45	-0.1526939
SNTG1	turquoise	0.70342207	6.13E-29	-0.5950385	4.20E-19	-0.6851228
SNRPN	turquoise	0.54742629	7.44E-16	-0.7759037	1.84E-38	-0.6343768
SNRPB2	turquoise	0.61496186	1.25E-20	-0.1786915	0.01494892	-0.6435684
SNRPA1	turquoise	0.55865621	1.42E-16	-0.5270793	1.28E-14	-0.7019457
SNRNP70	turquoise	-0.3567167	6.23E-07	0.04795032	0.51688969	0.68307817
SNRNP27	turquoise	0.38113263	8.69E-08	-0.0823591	0.26506197	-0.8026612
SNORD3A	turquoise	-0.2230916	0.00227041	0.05688426	0.44184078	0.71903494
SNORD114-	turquoise	0.60591124	6.37E-20	-0.7010111	1.13E-28	-0.297678
SNORD104	turquoise	-0.5184365	4.07E-14	0.35249275	8.62E-07	0.83935374
SNORA8	turquoise	0.60005702	1.77E-19	-0.4588024	5.10E-11	-0.5203503
SNORA18	turquoise	0.50787661	1.60E-13	-0.5003604	4.10E-13	-0.7189305
SNHG5	turquoise	-0.4392139	4.00E-10	0.6002046	1.73E-19	0.5880903
SNCA	turquoise	0.62056914	4.46E-21	-0.6135046	1.63E-20	-0.8596396
SNAR-A1	turquoise	-0.4137467	4.80E-09	0.28793265	7.06E-05	0.47051112
SNAP91	turquoise	0.64433727	4.37E-23	-0.70641	2.84E-29	-0.4145564
SNAP25	turquoise	0.55567706	2.21E-16	-0.629621	8.03E-22	-0.6664045
SMYD3	turquoise	0.68367013	7.91E-27	-0.6650834	5.46E-25	-0.7227108
SMYD2	turquoise	0.60936584	3.45E-20	-0.5827562	3.25E-18	-0.7964233
SMS	turquoise	0.39840441	1.95E-08	-0.0421095	0.56927396	-0.779529
SMPX	turquoise	0.71090922	8.73E-30	-0.4996454	4.48E-13	-0.5638193
SMPDL3A	turquoise	0.48265199	3.47E-12	-0.0133203	0.85718753	-0.7779604
SMOX	turquoise	-0.7459763	3.87E-34	0.61236327	2.01E-20	0.632314

SMOC1	turquoise	-0.6393411	1.20E-22	0.54216201	1.58E-15	0.6147185
SMO	turquoise	-0.6309166	6.25E-22	0.67822164	2.83E-26	0.69303391
SMIM14	turquoise	0.61333096	1.69E-20	-0.3144347	1.31E-05	-0.8111136
SMIM13	turquoise	0.67691163	3.82E-26	-0.4870958	2.05E-12	-0.742554
SMG5	turquoise	-0.6720522	1.16E-25	0.37212871	1.83E-07	0.5977896
SMG1	turquoise	0.31562564	1.21E-05	0.14767316	0.04485946	-0.6688354
SMARCD3	turquoise	-0.4861854	2.29E-12	0.20547691	0.00501843	0.76092748
SMARCD1	turquoise	-0.4070142	8.96E-09	0.05114258	0.48934426	0.66614314
SMARCC1	turquoise	-0.6249621	1.95E-21	0.67094229	1.49E-25	0.62004992
SMARCAD1	turquoise	0.5918418	7.22E-19	-0.0935308	0.20540134	-0.6128522
SMARCA5	turquoise	-0.3196717	9.19E-06	0.59778632	2.63E-19	0.56183026
SMARCA2	turquoise	0.63586546	2.38E-22	-0.4134983	4.92E-09	-0.2517042
SMAP2	turquoise	0.5523687	3.61E-16	-0.6996309	1.61E-28	-0.1302632
SMAP1	turquoise	0.62430713	2.21E-21	-0.4349296	6.16E-10	-0.7090235
SMAD6	turquoise	-0.464222	2.82E-11	0.63081157	6.38E-22	0.42722069
SLX4IP	turquoise	-0.5151076	6.30E-14	0.2415315	0.00092601	0.77113025
SLITRK5	turquoise	0.69415499	6.29E-28	-0.7934784	2.52E-41	-0.5812386
SLITRK4	turquoise	0.69479057	5.38E-28	-0.7542983	2.81E-35	-0.6969935
SLITRK3	turquoise	0.58188344	3.75E-18	-0.6779352	3.02E-26	-0.4384721
SLITRK1	turquoise	0.54754065	7.31E-16	-0.63491	2.87E-22	-0.8253933
SLIT3	turquoise	0.70876292	1.54E-29	-0.7059245	3.22E-29	-0.6815511
SLIRP	turquoise	0.42942699	1.06E-09	-0.1221918	0.09753203	-0.7639618
SLF2	turquoise	-0.3710817	1.99E-07	0.15832466	0.03136215	0.69375557
SLF1	turquoise	0.61774409	7.52E-21	-0.5251243	1.67E-14	-0.3889169
SLCO4A1	turquoise	-0.6225547	3.07E-21	0.62007365	4.89E-21	0.32175049
SLCO2B1	turquoise	-0.5127621	8.53E-14	0.76481616	8.74E-37	0.43219991
SLCO1C1	turquoise	0.27951539	0.00011661	0.11307361	0.1254084	-0.4627138
SLC9A9	turquoise	-0.4286725	1.15E-09	0.79029092	8.74E-41	0.39466927
SLC9A7	turquoise	0.56764509	3.60E-17	-0.633675	3.66E-22	-0.4243964
SLC9A6	turquoise	0.69634946	3.65E-28	-0.7384764	3.78E-33	-0.6634862
SLC9A3R1	turquoise	-0.6838836	7.52E-27	0.47926938	5.15E-12	0.70678219
SLC8A3	turquoise	0.58934058	1.10E-18	-0.6924732	9.51E-28	-0.3673978
SLC8A2	turquoise	0.3948421	2.67E-08	-0.7712599	9.53E-38	-0.4652458
SLC8A1	turquoise	0.65176373	9.48E-24	-0.7347873	1.13E-32	-0.5563152
SLC7A5	turquoise	-0.6855271	5.09E-27	0.45656176	6.50E-11	0.72106092
SLC7A2	turquoise	-0.5728176	1.60E-17	0.66687305	3.68E-25	0.62049593
SLC7A14	turquoise	0.5760564	9.58E-18	-0.712984	5.03E-30	-0.2188489
SLC6A9	turquoise	-0.6058524	6.44E-20	0.45741193	5.93E-11	0.46285628
SLC6A8	turquoise	-0.6110464	2.55E-20	0.13810759	0.06083014	0.42821022
SLC6A15	turquoise	0.64480955	3.97E-23	-0.6739319	7.56E-26	-0.7262124
SLC6A12	turquoise	-0.6828278	9.64E-27	0.61796672	7.22E-21	0.56569367
SLC6A10P	turquoise	-0.621514	3.74E-21	0.19241118	0.0086929	0.75455726
SLC6A1	turquoise	0.19932755	0.00652576	0.15996326	0.02962906	-0.6527976
SLC52A3	turquoise	-0.5942593	4.80E-19	0.57141041	2.00E-17	0.46686786
SLC52A2	turquoise	-0.6130346	1.78E-20	0.2617262	0.00031985	0.59035668
SLC4A1AP	turquoise	0.70687078	2.52E-29	-0.591943	7.09E-19	-0.7204074

SLC4A11	turquoise	-0.5110297	1.07E-13	0.43943688	3.91E-10	0.46972746
SLC48A1	turquoise	-0.225705	0.00200782	0.23504926	0.00127928	0.73407139
SLC3A2	turquoise	-0.7300789	4.43E-32	0.60702204	5.23E-20	0.40604764
SLC39A11	turquoise	-0.4755915	7.86E-12	0.63948373	1.16E-22	0.51029862
SLC39A10	turquoise	0.75693963	1.20E-35	-0.6223047	3.22E-21	-0.7287141
SLC39A1	turquoise	-0.5904	9.19E-19	0.65284857	7.55E-24	0.57366851
SLC38A7	turquoise	-0.2629163	0.00029962	-0.2690922	0.0002124	0.56784816
SLC38A3	turquoise	-0.4313786	8.78E-10	0.22204367	0.00238421	0.49874091
SLC35G2	turquoise	0.36156691	4.27E-07	0.15083628	0.04042005	-0.6106173
SLC35E3	turquoise	0.53027183	8.32E-15	-0.2568564	0.00041659	-0.4932396
SLC35B4	turquoise	0.58861285	1.24E-18	-0.6335255	3.77E-22	-0.4704952
SLC35B1	turquoise	0.38944956	4.28E-08	-0.5873097	1.54E-18	-0.7045725
SLC35A5	turquoise	0.39333443	3.05E-08	-0.0264618	0.72068551	-0.4343265
SLC35A2	turquoise	0.33954524	2.26E-06	-0.7449044	5.39E-34	-0.4493992
SLC35A1	turquoise	0.52984111	8.82E-15	-0.2824028	9.83E-05	-0.6303772
SLC30A9	turquoise	0.66508845	5.46E-25	-0.5500234	5.10E-16	-0.6876951
SLC30A3	turquoise	0.40128206	1.51E-08	-0.8232235	7.24E-47	-0.3272485
SLC2A4RG	turquoise	-0.5324849	6.14E-15	0.60596277	6.31E-20	0.68885561
SLC2A1	turquoise	-0.4822653	3.63E-12	0.68482484	6.01E-27	0.41078867
SLC29A4	turquoise	-0.4143362	4.54E-09	0.20038289	0.00624138	0.69426666
SLC27A1	turquoise	-0.6009013	1.53E-19	0.21878471	0.00277195	0.65700487
SLC26A8	turquoise	0.3881105	4.80E-08	-0.7201262	7.27E-31	-0.505397
SLC26A4	turquoise	0.71508449	2.87E-30	-0.5434817	1.31E-15	-0.2349844
SLC25A5	turquoise	0.3189946	9.62E-06	0.05994196	0.41765174	-0.8025479
SLC25A46	turquoise	0.64377028	4.91E-23	-0.3480963	1.20E-06	-0.8132009
SLC25A44	turquoise	0.66867307	2.47E-25	-0.6782462	2.81E-26	-0.6517197
SLC25A42	turquoise	-0.3561505	6.51E-07	0.13396684	0.0690642	0.70998435
SLC25A40	turquoise	0.63721325	1.82E-22	-0.4863752	2.24E-12	-0.7190633
SLC25A4	turquoise	0.51556551	5.93E-14	-0.2709532	0.00019117	-0.9042359
SLC25A37	turquoise	-0.5040308	2.59E-13	0.16846483	0.02189141	0.66342987
SLC25A3	turquoise	0.23304904	0.00141096	-0.0909641	0.21816811	-0.7317238
SLC25A29	turquoise	-0.4962698	6.79E-13	0.15322034	0.03732349	0.80164535
SLC25A28	turquoise	-0.6872405	3.38E-27	0.34970618	1.07E-06	0.54705648
SLC25A26	turquoise	0.42703774	1.35E-09	-0.3793747	1.01E-07	-0.4445987
SLC25A20	turquoise	-0.4998333	4.38E-13	0.75135299	7.20E-35	0.63762836
SLC25A18	turquoise	-0.6699799	1.84E-25	0.58569312	2.01E-18	0.77168783
SLC25A12	turquoise	0.63585962	2.38E-22	-0.6462785	2.94E-23	-0.6772932
SLC22A1	turquoise	-0.3749131	1.46E-07	0.19646894	0.00735499	0.81075088
SLC20A1	turquoise	0.40687994	9.07E-09	-0.1988353	0.00666232	-0.7460296
SLC1A7	turquoise	-0.4754501	7.99E-12	0.46357752	3.03E-11	0.48374487
SLC1A4	turquoise	-0.3523203	8.73E-07	0.2202381	0.00259247	0.70125605
SLC1A1	turquoise	0.41418284	4.61E-09	-0.093809	0.204051	-0.7039987
SLC19A2	turquoise	-0.5239098	1.97E-14	0.25416465	0.00048106	0.73714303
SLC18B1	turquoise	-0.375855	1.35E-07	0.5580304	1.56E-16	0.6919433
SLC17A7	turquoise	0.29184912	5.56E-05	-0.7017434	9.40E-29	-0.3047864
SLC17A6	turquoise	0.59349798	5.46E-19	-0.7373981	5.21E-33	-0.4504217

SLC16A9	turquoise	-0.5174406	4.64E-14	0.69831774	2.23E-28	0.67308411
SLC16A3	turquoise	-0.3393538	2.30E-06	0.29970848	3.41E-05	0.447741
SLC16A14	turquoise	0.56112693	9.77E-17	-0.4209036	2.44E-09	-0.9037033
SLC15A3	turquoise	-0.5564209	1.98E-16	0.70331852	6.29E-29	0.72825818
SLC13A5	turquoise	-0.1758261	0.01666733	0.2245203	0.00212323	0.54081166
SLC12A9	turquoise	-0.489082	1.62E-12	0.33318603	3.58E-06	0.88003513
SLC12A5	turquoise	0.56900626	2.91E-17	-0.7184765	1.14E-30	-0.600362
SLC11A1	turquoise	-0.346654	1.34E-06	0.42771474	1.26E-09	0.34280907
SLBP	turquoise	0.70408878	5.16E-29	-0.3974863	2.12E-08	-0.4622485
SLA	turquoise	-0.207277	0.00464064	0.52776612	1.17E-14	0.06162855
SKP1	turquoise	0.62026132	4.72E-21	-0.3921263	3.39E-08	-0.8427551
SKAP2	turquoise	0.58446683	2.46E-18	-0.4037826	1.20E-08	-0.8038227
SKA2	turquoise	-0.2944721	4.73E-05	0.58462459	2.40E-18	0.56730984
SIRT2	turquoise	-0.3761365	1.32E-07	0.28746375	7.26E-05	0.20064897
SIMC1	turquoise	0.56935507	2.76E-17	-0.3210639	8.35E-06	-0.8171238
SIK1	turquoise	-0.3310887	4.16E-06	0.50254854	3.12E-13	0.26282799
SIAH1	turquoise	0.41494793	4.29E-09	-0.1699242	0.02075587	-0.6956165
SHTN1	turquoise	-0.4457513	2.04E-10	0.31454089	1.30E-05	0.27978991
SHROOM1	turquoise	-0.4709066	1.34E-11	0.27211567	0.00017893	0.50666205
SHPK	turquoise	-0.2425129	0.00088113	0.29931563	3.49E-05	0.62645045
SHMT2	turquoise	-0.5290688	9.80E-15	0.30175093	2.99E-05	0.67898191
SHD	turquoise	0.21659584	0.00306354	-0.3521649	8.84E-07	0.24156326
SHC1	turquoise	-0.6014262	1.40E-19	0.37652397	1.28E-07	0.53288698
SHANK2	turquoise	0.63390867	3.49E-22	-0.6768	3.92E-26	-0.2888555
SH3TC1	turquoise	-0.4979896	5.50E-13	0.51212301	9.27E-14	0.47056926
SH3PXD2A	turquoise	-0.4890992	1.62E-12	0.31430903	1.32E-05	0.73759991
SH3KBP1	turquoise	0.20625352	0.00485218	-0.245943	0.00073951	-0.5459431
SH3GL2	turquoise	0.61294174	1.81E-20	-0.6995469	1.64E-28	-0.632217
SH3BP4	turquoise	-0.6232863	2.68E-21	0.65194242	9.13E-24	0.3659125
SH3BGRL2	turquoise	0.54820247	6.64E-16	-0.6481458	2.01E-23	-0.1280481
SH3BGRL	turquoise	0.22220453	0.00236641	0.34560769	1.45E-06	-0.6404214
SH2D5	turquoise	0.47090837	1.34E-11	-0.8526729	1.74E-53	-0.351928
SGTB	turquoise	0.66008205	1.62E-24	-0.3511628	9.54E-07	-0.5772135
SGSM1	turquoise	0.44238349	2.89E-10	-0.7778122	9.27E-39	-0.3171177
SGK223	turquoise	0.31245839	1.49E-05	-0.494518	8.41E-13	0.13983821
SGIP1	turquoise	0.62237107	3.18E-21	-0.3938595	2.91E-08	-0.7931028
SFXN5	turquoise	-0.6609359	1.35E-24	0.42641698	1.43E-09	0.64467333
SFXN4	turquoise	-0.3196842	9.18E-06	-0.0046341	0.95008169	0.7234435
SFXN1	turquoise	0.51447167	6.84E-14	-0.1212799	0.10007715	-0.6792721
SF3B2	turquoise	-0.4791816	5.20E-12	0.05652229	0.44475599	0.30741991
SEZ6L2	turquoise	0.41365894	4.84E-09	-0.8221595	1.19E-46	-0.2068555
SETX	turquoise	-0.5506412	4.66E-16	0.39481072	2.68E-08	0.72146517
SETD2	turquoise	-0.407929	8.24E-09	0.36653024	2.88E-07	0.48869685
SERTM1	turquoise	0.70361863	5.83E-29	-0.6277013	1.16E-21	-0.649086
SERTAD1	turquoise	-0.6482342	1.97E-23	0.45470321	7.94E-11	0.5155405
SERPINI1	turquoise	0.62505286	1.92E-21	-0.5230639	2.20E-14	-0.8921089

SERPING1	turquoise	-0.5569407	1.83E-16	0.50999398	1.22E-13	0.2381587
SERPINF1	turquoise	0.48809653	1.82E-12	-0.5211418	2.85E-14	-0.3097302
SERPINB6	turquoise	-0.6941029	6.37E-28	0.62880635	9.39E-22	0.79133164
SERPINA3	turquoise	-0.3488054	1.14E-06	0.25785239	0.00039483	0.16553997
SERP2	turquoise	0.49774273	5.67E-13	-0.3324943	3.76E-06	-0.4999418
SERINC3	turquoise	0.34149202	1.96E-06	-0.3089897	1.87E-05	-0.6711841
SERINC1	turquoise	0.48943289	1.55E-12	-0.1593356	0.03028298	-0.8643799
SERF2	turquoise	-0.5981093	2.48E-19	0.45919165	4.89E-11	0.19084561
SERAC1	turquoise	0.67488013	6.09E-26	-0.6902718	1.63E-27	-0.5244453
4-Sep	turquoise	-0.4609125	4.06E-11	0.25662448	0.00042181	0.5863603
3-Sep	turquoise	0.55222808	3.69E-16	-0.5201806	3.24E-14	-0.3889587
2-Sep	turquoise	-0.489786	1.49E-12	0.64812974	2.01E-23	0.62293063
15-Sep	turquoise	0.29654968	4.15E-05	0.26069911	0.00033833	-0.7071866
SEMA6C	turquoise	-0.3016745	3.01E-05	0.0044686	0.95186268	0.69198754
SEMA5B	turquoise	0.32665671	5.68E-06	-0.7402752	2.20E-33	-0.1418947
SEMA4F	turquoise	0.51107909	1.06E-13	-0.7502279	1.03E-34	-0.6571026
SELT	turquoise	0.62597922	1.61E-21	-0.2509865	0.00056901	-0.6585029
SELENBP1	turquoise	-0.6148638	1.28E-20	0.62527267	1.84E-21	0.36856079
SEL1L	turquoise	0.73980733	2.54E-33	-0.573088	1.53E-17	-0.4476254
SEH1L	turquoise	0.56182875	8.78E-17	-0.6630459	8.53E-25	-0.8215404
SEC63	turquoise	-0.3169374	1.11E-05	0.18246393	0.01292384	0.77968228
SEC62	turquoise	0.50171153	3.47E-13	-0.3583894	5.47E-07	-0.5156669
SEC61G	turquoise	0.61468736	1.32E-20	-0.382002	8.07E-08	-0.6897588
SEC61A2	turquoise	0.58181282	3.79E-18	-0.6868504	3.71E-27	-0.6213475
SEC24D	turquoise	0.64599976	3.12E-23	-0.1592089	0.03041649	-0.6036218
SEC24C	turquoise	-0.4511786	1.15E-10	-0.106696	0.14831313	0.67081961
SEC24B	turquoise	0.51292884	8.35E-14	0.16911449	0.02137944	-0.5698515
SEC23B	turquoise	0.24548081	0.00075728	0.19981397	0.00639328	-0.6640555
SEC14L2	turquoise	-0.4250475	1.63E-09	0.38723551	5.17E-08	0.36253403
SEC14L1	turquoise	-0.4371524	4.92E-10	0.7505531	9.27E-35	0.56250129
SEC11C	turquoise	0.17786863	0.01542583	0.28213835	9.99E-05	-0.6174017
SEC11A	turquoise	-0.3891126	4.40E-08	0.83997094	1.82E-50	0.18601928
SDHD	turquoise	0.27315791	0.00016858	0.26646954	0.00024606	-0.5871898
SDHB	turquoise	0.35513343	7.04E-07	-0.1783309	0.01515631	-0.8552609
SDHAP2	turquoise	-0.5057443	2.09E-13	0.37374753	1.60E-07	0.80428053
SDHAF3	turquoise	0.56271275	7.67E-17	-0.140372	0.05667985	-0.5157842
SDHAF1	turquoise	0.28212402	1.00E-04	-0.6370631	1.88E-22	-0.5308612
SDHA	turquoise	0.39648733	2.31E-08	-0.3347688	3.20E-06	-0.7798271
SDF2L1	turquoise	-0.4638104	2.95E-11	0.24250961	0.00088127	0.68637643
SDCCAG8	turquoise	-0.4208578	2.45E-09	0.32163791	8.03E-06	0.65228626
SDCCAG3	turquoise	-0.5841102	2.61E-18	0.3790874	1.03E-07	0.76700462
SDC2	turquoise	-0.3646363	3.35E-07	0.06394463	0.3871862	0.74297247
SDAD1	turquoise	0.52893911	9.98E-15	-0.6270507	1.31E-21	-0.5186877
SCYL2	turquoise	0.44228981	2.92E-10	0.09085512	0.21872223	-0.6465521
SCPEP1	turquoise	0.36015678	4.77E-07	-0.0804683	0.27622614	-0.716481
SCOC	turquoise	0.66217894	1.03E-24	-0.3993325	1.79E-08	-0.860084

SCO1	turquoise	0.46470884	2.67E-11	-0.0395898	0.59262287	-0.7072204
SCN3B	turquoise	0.52335169	2.12E-14	-0.5930379	5.90E-19	-0.8112006
SCN2B	turquoise	0.61301811	1.78E-20	-0.694994	5.11E-28	-0.8106011
SCIN	turquoise	-0.2971838	3.99E-05	0.50773869	1.62E-13	0.10466001
SCHIP1	turquoise	0.57868283	6.29E-18	-0.4642781	2.80E-11	-0.8448499
SCG5	turquoise	0.67170271	1.25E-25	-0.7910138	6.60E-41	-0.6523717
SCG3	turquoise	0.40884045	7.58E-09	-0.1295425	0.07884312	-0.5002003
SCG2	turquoise	0.71177386	6.94E-30	-0.7079266	1.91E-29	-0.7113884
SCFD1	turquoise	0.59720619	2.90E-19	-0.2210122	0.00250124	-0.7440308
SCARB2	turquoise	0.3289557	4.83E-06	-0.1542525	0.03604634	-0.5049497
SCARA3	turquoise	-0.268571	0.00021872	0.61153828	2.33E-20	0.33675334
SCAMP3	turquoise	-0.5346526	4.55E-15	0.01291234	0.86151679	0.68403573
SCAMP1	turquoise	0.60336414	9.97E-20	-0.7086044	1.60E-29	-0.3371932
SATB2-AS1	turquoise	0.68526825	5.41E-27	-0.7030088	6.81E-29	-0.5032718
SATB2	turquoise	0.64074499	9.03E-23	-0.5404426	2.02E-15	-0.6548918
SATB1	turquoise	0.39514054	2.60E-08	-0.4380038	4.52E-10	-0.7395098
SAT1	turquoise	-0.4062922	9.57E-09	0.71689156	1.76E-30	0.16203152
SART1	turquoise	-0.450746	1.21E-10	0.31932716	9.40E-06	0.69269547
SARS	turquoise	0.28734144	7.32E-05	-0.7094913	1.27E-29	-0.4118909
SARAF	turquoise	0.61700972	8.61E-21	-0.3710304	2.00E-07	-0.8577716
SAPCD2	turquoise	-0.4384331	4.33E-10	0.35211485	8.87E-07	0.54232392
SAP18	turquoise	-0.4326051	7.77E-10	0.53919548	2.41E-15	0.52996106
SALL2	turquoise	-0.5633776	6.93E-17	0.69323091	7.90E-28	0.33232312
SAFB2	turquoise	-0.6228487	2.91E-21	0.33517421	3.11E-06	0.33372515
SACS	turquoise	0.73045135	3.98E-32	-0.5309389	7.59E-15	-0.4182229
SACM1L	turquoise	0.57914862	5.84E-18	-0.3276268	5.31E-06	-0.1354208
SAC3D1	turquoise	-0.4221885	2.15E-09	-0.2182236	0.0028442	0.57634211
S1PR3	turquoise	-0.3883444	4.70E-08	0.75289298	4.41E-35	0.13494461
S100PBP	turquoise	-0.6180689	7.08E-21	0.68539117	5.26E-27	0.66344736
S100A6	turquoise	-0.4522382	1.03E-10	0.51294438	8.34E-14	0.41474336
S100A4	turquoise	-0.3211938	8.28E-06	0.60804575	4.36E-20	0.26529667
S100A13	turquoise	-0.542524	1.50E-15	0.4362231	5.41E-10	0.72529266
S100A10	turquoise	-0.398237	1.98E-08	0.65355172	6.51E-24	0.15974881
RYR2	turquoise	0.18276388	0.01277367	-0.6917678	1.13E-27	-0.177567
RYBP	turquoise	-0.2338271	0.00135833	0.07270325	0.32537575	0.57029591
RXRA	turquoise	-0.6885763	2.45E-27	0.71855791	1.12E-30	0.70928742
RXFP1	turquoise	0.70172358	9.45E-29	-0.5185146	4.03E-14	-0.698093
RWDD2A	turquoise	0.68538597	5.26E-27	-0.5941115	4.92E-19	-0.6692126
RUNX1T1	turquoise	0.36890969	2.38E-07	-0.4328236	7.60E-10	-0.4311785
RUNDC1	turquoise	0.67634068	4.36E-26	-0.7440051	7.10E-34	-0.6499295
RUFY3	turquoise	0.64618074	3.00E-23	-0.5679137	3.45E-17	-0.8393754
RTN4IP1	turquoise	0.74094647	1.80E-33	-0.6583608	2.35E-24	-0.6154171
RTN4	turquoise	0.60463007	7.99E-20	-0.4815077	3.97E-12	-0.8982098
RTN3	turquoise	0.54869203	6.19E-16	-0.8196555	3.78E-46	-0.5034297
RTN1	turquoise	0.61338187	1.67E-20	-0.6367138	2.01E-22	-0.8402736
RTCA	turquoise	0.65107279	1.09E-23	-0.2705306	0.00019581	-0.7771532

RSRC1	turquoise	0.52655559	1.38E-14	-0.1411739	0.05526738	-0.748786
RSPO2	turquoise	0.71869698	1.08E-30	-0.6253184	1.83E-21	-0.756531
RSL24D1	turquoise	0.72005817	7.40E-31	-0.6093109	3.48E-20	-0.4570344
RRBP1	turquoise	-0.7014786	1.01E-28	0.49306524	1.00E-12	0.74730483
RRAGC	turquoise	-0.4345781	6.38E-10	0.31533882	1.23E-05	0.7261629
RRAGB	turquoise	0.65097525	1.12E-23	-0.3613827	4.33E-07	-0.7041445
RPSA	turquoise	-0.5466782	8.29E-16	0.5698606	2.55E-17	0.29939382
RPS9	turquoise	-0.4971037	6.13E-13	0.68536569	5.29E-27	0.20957464
RPS6KC1	turquoise	0.64684385	2.62E-23	-0.5317997	6.75E-15	-0.7267003
RPS6KA1	turquoise	-0.5060238	2.02E-13	0.38058458	9.10E-08	0.73276217
RPS5	turquoise	-0.4090153	7.45E-09	0.5413684	1.77E-15	-0.0275076
RPS3	turquoise	-0.5951601	4.11E-19	0.70974028	1.19E-29	0.1737205
RPS19	turquoise	-0.6126333	1.91E-20	0.606809	5.43E-20	0.49655725
RPS16	turquoise	-0.4653995	2.48E-11	0.60800114	4.40E-20	0.69024846
RPS14	turquoise	-0.4946099	8.32E-13	0.41054936	6.47E-09	0.73800423
RPRML	turquoise	0.38918367	4.38E-08	-0.7764729	1.50E-38	-0.2211237
RPRM	turquoise	0.19289393	0.00852316	-0.2735592	0.00016475	0.1892386
RPRD2	turquoise	0.61462683	1.33E-20	-0.5198807	3.37E-14	-0.2327108
RPRD1B	turquoise	0.58772111	1.44E-18	-0.039205	0.5962261	-0.6265164
RPP40	turquoise	0.55517483	2.39E-16	-0.7103111	1.02E-29	-0.2734626
RPL4	turquoise	-0.4559359	6.95E-11	0.42393321	1.82E-09	0.27601843
RPL23AP7	turquoise	-0.4828612	3.39E-12	0.46424142	2.81E-11	0.7366482
RPL23AP32	turquoise	-0.3968254	2.24E-08	0.14882096	0.04320355	0.71477301
RPL14	turquoise	-0.367127	2.74E-07	0.45643202	6.59E-11	0.68829363
RPL13P5	turquoise	-0.4220356	2.19E-09	0.32346783	7.08E-06	0.86410959
RPL13AP3	turquoise	-0.64429	4.42E-23	0.46262347	3.36E-11	0.294929
RPL13A	turquoise	-0.5822105	3.56E-18	0.58214084	3.60E-18	0.23003942
RPL13	turquoise	-0.3280811	5.14E-06	0.16859486	0.0217881	0.71465254
RPL11	turquoise	-0.6050636	7.40E-20	0.69437768	5.96E-28	0.37023905
RPF1	turquoise	0.63844641	1.43E-22	-0.0819304	0.26756563	-0.468782
RPE	turquoise	0.48818537	1.80E-12	-0.1506199	0.04071154	-0.7638565
RPAP3	turquoise	0.55024752	4.93E-16	-0.2412746	0.0009381	-0.8005868
RPA3	turquoise	0.60453979	8.11E-20	-0.3998245	1.72E-08	-0.7836805
ROM1	turquoise	-0.749809	1.17E-34	0.53110884	7.42E-15	0.71946908
ROBO3	turquoise	-0.6693095	2.14E-25	0.6006211	1.61E-19	0.49539058
ROBO2	turquoise	0.6595894	1.80E-24	-0.7146395	3.23E-30	-0.6669742
ROBO1	turquoise	0.58299092	3.13E-18	-0.134046	0.06889877	-0.1325094
RNY1	turquoise	-0.5527593	3.41E-16	0.37227152	1.81E-07	0.81610162
RNVU1-7	turquoise	-0.2611473	0.00033014	0.30521917	2.39E-05	0.44946662
RNU6-1	turquoise	-0.5750037	1.13E-17	0.45325341	9.26E-11	0.819275
RNU4-2	turquoise	-0.3065721	2.19E-05	0.3252693	6.25E-06	0.62354283
RNPEPL1	turquoise	-0.5115803	9.94E-14	0.20839172	0.00441969	0.73680677
RNFT2	turquoise	0.52648986	1.39E-14	-0.8228791	8.51E-47	-0.3648853
RNF41	turquoise	0.49904111	4.83E-13	-0.8006455	1.42E-42	-0.6950196
RNF4	turquoise	0.53056521	7.99E-15	-0.0638583	0.38782883	-0.5727845
RNF38	turquoise	0.64824484	1.97E-23	-0.2645055	0.00027445	-0.5903137

RNF34	turquoise	0.60999669	3.08E-20	-0.4851772	2.58E-12	-0.3454315
RNF219	turquoise	0.66571861	4.75E-25	-0.7319823	2.55E-32	-0.4275865
RNF175	turquoise	0.69382414	6.83E-28	-0.7913686	5.75E-41	-0.5630789
RNF170	turquoise	0.55276877	3.41E-16	-0.1715768	0.01953171	-0.4885693
RNF144A	turquoise	-0.466903	2.10E-11	0.37325412	1.67E-07	0.48391161
RNF14	turquoise	0.66239112	9.84E-25	-0.4259212	1.50E-09	-0.629881
RNF135	turquoise	-0.508642	1.45E-13	0.81244658	9.58E-45	0.65429834
RNF114	turquoise	-0.5684159	3.19E-17	0.80854961	5.19E-44	0.64967695
RNF103	turquoise	0.39151938	3.57E-08	0.12341469	0.09419978	-0.6781203
RND2	turquoise	-0.707501	2.14E-29	0.39053826	3.89E-08	0.62027853
RNASET2	turquoise	-0.4700184	1.48E-11	0.55406452	2.81E-16	0.60775759
RNASEH2C	turquoise	-0.5052217	2.23E-13	0.19308865	0.00845553	0.34372061
RNASEH2B	turquoise	0.59366079	5.31E-19	-0.5013763	3.62E-13	-0.0321381
RMI2	turquoise	0.49809957	5.43E-13	-0.6441916	4.51E-23	-0.561611
RIT2	turquoise	0.63701731	1.90E-22	-0.7170126	1.70E-30	-0.4449225
RIPPLY2	turquoise	0.63595187	2.34E-22	-0.6234704	2.59E-21	-0.6505258
RIPK1	turquoise	-0.5760119	9.65E-18	0.4869965	2.08E-12	0.71585118
RIMS4	turquoise	-0.4636527	3.00E-11	-0.0492842	0.50528389	0.35816999
RIMS3	turquoise	0.4562075	6.75E-11	-0.6381078	1.53E-22	-0.3518422
RIMKLB	turquoise	-0.1722202	0.0190724	0.32327024	7.18E-06	0.66948059
RIMKLA	turquoise	0.66553954	4.94E-25	-0.7238283	2.60E-31	-0.4231394
RIMBP2	turquoise	0.7061918	3.00E-29	-0.7143282	3.51E-30	-0.7132618
RIC3	turquoise	0.62098416	4.12E-21	-0.3754582	1.39E-07	-0.7355368
RHPN2	turquoise	-0.333541	3.49E-06	0.68354707	8.14E-27	0.51979337
RHPN1	turquoise	-0.3616455	4.24E-07	-0.2501454	0.00059465	0.42323143
RHOQ	turquoise	-0.5719481	1.84E-17	0.70462918	4.49E-29	0.50772
RHOJ	turquoise	-0.6217884	3.55E-21	0.77431726	3.24E-38	0.68050602
RHOG	turquoise	-0.6652531	5.26E-25	0.38207415	8.03E-08	0.14354896
RHOC	turquoise	-0.6822051	1.12E-26	0.71438614	3.46E-30	0.53433934
RHNO1	turquoise	0.4576009	5.81E-11	-0.2926908	5.28E-05	-0.410423
RHBDF2	turquoise	-0.5684538	3.17E-17	0.71916543	9.46E-31	0.5624144
RHBDD1	turquoise	-0.5200469	3.29E-14	0.73120318	3.20E-32	0.3881701
RGS7BP	turquoise	0.61673866	9.05E-21	-0.6163964	9.64E-21	-0.73392
RGS7	turquoise	0.67419075	7.13E-26	-0.6502895	1.29E-23	-0.8382766
RGS4	turquoise	0.67436996	6.84E-26	-0.6529265	7.43E-24	-0.8052022
RGS2	turquoise	0.41191566	5.70E-09	-0.3924895	3.28E-08	-0.1820865
RGS11	turquoise	-0.4637786	2.96E-11	0.09968228	0.17701572	0.7531991
RGR	turquoise	-0.2921841	5.45E-05	0.48272959	3.44E-12	0.27168226
RGMA	turquoise	-0.6555907	4.24E-24	0.45663228	6.45E-11	0.47055782
RGL3	turquoise	-0.4830277	3.32E-12	0.37008652	2.16E-07	0.75990759
RGL2	turquoise	-0.5557825	2.18E-16	0.74288724	9.99E-34	0.43473981
RFXANK	turquoise	-0.5631724	7.15E-17	0.42789475	1.24E-09	0.52381883
RFX3	turquoise	0.60751604	4.79E-20	-0.1513644	0.03971609	-0.6506781
RFX2	turquoise	-0.4881137	1.82E-12	0.65653971	3.46E-24	0.5210439
RFWD2	turquoise	0.55960749	1.23E-16	-0.1765764	0.01620153	-0.7315699
RFTN1	turquoise	0.55019303	4.97E-16	-0.7066934	2.63E-29	-0.2490601

RFPL2	turquoise	0.55652883	1.95E-16	-0.4221165	2.17E-09	-0.3280292
REPS2	turquoise	0.70809736	1.83E-29	-0.6468252	2.63E-23	-0.7928398
REPS1	turquoise	0.62107221	4.06E-21	-0.2596637	0.00035795	-0.596901
REPIN1	turquoise	-0.4777444	6.14E-12	0.66160817	1.17E-24	0.63640283
RENBP	turquoise	-0.3716925	1.90E-07	0.0689076	0.35133626	0.11396518
REEP5	turquoise	0.71838716	1.17E-30	-0.7095262	1.26E-29	-0.6181423
REEP1	turquoise	0.64199535	7.03E-23	-0.8581861	6.91E-55	-0.5625874
REC8	turquoise	-0.3067083	2.17E-05	-0.1911243	0.00916009	0.43946174
RDX	turquoise	-0.4043123	1.15E-08	0.81311486	7.14E-45	0.12092828
RDH13	turquoise	-0.3651886	3.20E-07	-0.0687495	0.3524448	0.74926472
RCOR3	turquoise	0.61338562	1.67E-20	-0.4007763	1.58E-08	-0.5135624
RCN2	turquoise	0.5070352	1.78E-13	-0.0710576	0.33647509	-0.811696
RCAN2	turquoise	0.65828485	2.39E-24	-0.7761449	1.69E-38	-0.6734318
RBSN	turquoise	0.64767061	2.21E-23	-0.4658793	2.35E-11	-0.4223625
RBPJ	turquoise	-0.4300861	9.98E-10	0.55942729	1.26E-16	0.68456022
RBP4	turquoise	0.55241651	3.59E-16	-0.626804	1.38E-21	-0.7651953
RBMS2	turquoise	-0.3200679	8.94E-06	0.5355286	4.03E-15	0.59289551
RBM4B	turquoise	-0.6396597	1.12E-22	0.53370959	5.18E-15	0.46307996
RBM39	turquoise	-0.6335484	3.75E-22	0.4091879	7.34E-09	0.80381913
RBM3	turquoise	0.52577963	1.53E-14	-0.3656362	3.09E-07	-0.4891867
RBM26	turquoise	0.41144865	5.95E-09	0.11517159	0.11850376	-0.7249885
RBM24	turquoise	0.71260112	5.57E-30	-0.5222806	2.45E-14	-0.7456194
RBM17	turquoise	-0.5414469	1.75E-15	0.79115122	6.26E-41	0.6951723
RBM11	turquoise	0.7507354	8.75E-35	-0.5810028	4.33E-18	-0.7487792
RBM10	turquoise	-0.3260532	5.92E-06	0.06501088	0.37930329	0.79721858
RBFOX1	turquoise	0.4931179	9.97E-13	-0.7816895	2.25E-39	-0.6506527
RBBP7	turquoise	0.54940594	5.58E-16	-0.1590824	0.03055032	-0.7615329
RBBP5	turquoise	0.61188053	2.19E-20	-0.3324932	3.76E-06	-0.6963595
RASSF7	turquoise	-0.3556421	6.77E-07	-0.0155976	0.83310127	0.75204222
RASSF4	turquoise	-0.4262121	1.46E-09	0.31643663	1.14E-05	0.77867586
RASSF2	turquoise	-0.6682239	2.73E-25	0.55090555	4.48E-16	0.42534513
RASSF1	turquoise	-0.5474753	7.38E-16	0.4941058	8.84E-13	0.61848486
RASL12	turquoise	-0.54411	1.20E-15	0.62121362	3.95E-21	0.67530775
RASL11B	turquoise	0.61694318	8.72E-21	-0.7334436	1.67E-32	-0.6359852
RASGRP1	turquoise	0.69746677	2.77E-28	-0.6204292	4.57E-21	-0.4627163
RASD1	turquoise	0.21461031	0.00335175	-0.211707	0.00381735	-0.6014939
RASA1	turquoise	0.67670205	4.01E-26	-0.4901295	1.43E-12	-0.7471076
RARRES3	turquoise	-0.5926838	6.26E-19	0.55990542	1.17E-16	0.41437197
RARRES2	turquoise	-0.6144117	1.39E-20	0.47812235	5.88E-12	0.56243279
RAPH1	turquoise	0.62114382	4.00E-21	-0.3553764	6.91E-07	-0.618818
RAPGEF5	turquoise	0.45195039	1.06E-10	-0.3287892	4.89E-06	-0.615832
RAPGEF3	turquoise	-0.6501663	1.32E-23	0.46276992	3.31E-11	0.84877132
RAPGEF2	turquoise	0.65973852	1.75E-24	-0.4151181	4.22E-09	-0.7411269
RAPGEF1	turquoise	-0.5132294	8.03E-14	0.48690414	2.10E-12	0.73018593
RAP2A	turquoise	0.58475408	2.35E-18	-0.2948945	4.61E-05	-0.7337472
RAP1GAP2	turquoise	0.43375336	6.93E-10	-0.5781783	6.82E-18	-0.6393868

RANBP9	turquoise	0.74145357	1.54E-33	-0.3611108	4.42E-07	-0.4014042
RANBP6	turquoise	0.61972541	5.21E-21	-0.2365473	0.00118813	-0.7393677
RANBP2	turquoise	0.72902603	5.98E-32	-0.3228874	7.37E-06	-0.6374413
RANBP1	turquoise	0.62966446	7.96E-22	-0.4570432	6.17E-11	-0.4813426
RAMP3	turquoise	-0.4353108	5.93E-10	0.15984257	0.02975386	0.69111596
RALYL	turquoise	0.56717883	3.87E-17	-0.7139137	3.92E-30	-0.5455471
RALY	turquoise	-0.6356251	2.50E-22	0.20141262	0.00597461	0.41063845
RALGDS	turquoise	-0.5908947	8.46E-19	0.35898152	5.23E-07	0.62991984
RALGAPA1	turquoise	0.58803105	1.36E-18	-0.1539406	0.03642834	-0.5573877
RALB	turquoise	0.42304816	1.98E-09	-0.0285699	0.69946903	-0.7748306
RALA	turquoise	-0.3903797	3.95E-08	0.51688433	4.99E-14	0.6301878
RAI14	turquoise	-0.6781872	2.85E-26	0.69490299	5.23E-28	0.62220688
RAF1	turquoise	-0.4031062	1.28E-08	0.58851792	1.26E-18	0.66435091
RAD54L2	turquoise	-0.4533273	9.19E-11	0.16710653	0.02299611	0.84547529
RAD51C	turquoise	0.64228138	6.63E-23	-0.4519282	1.07E-10	-0.7502152
RAD23B	turquoise	0.61022292	2.95E-20	-0.4622329	3.51E-11	-0.7221436
RABGGTB	turquoise	0.43514855	6.03E-10	-0.0066902	0.92798431	-0.3322466
RABGEF1	turquoise	0.64677104	2.66E-23	-0.4434095	2.60E-10	-0.0649204
RABGAP1	turquoise	-0.4052219	1.06E-08	0.02829655	0.70220724	0.63215488
RABEPK	turquoise	0.4585169	5.26E-11	-0.4286407	1.15E-09	-0.0464181
RAB8A	turquoise	-0.5414898	1.74E-15	0.65768341	2.71E-24	0.58304752
RAB6B	turquoise	0.50093024	3.82E-13	-0.7526212	4.81E-35	-0.3582528
RAB6A	turquoise	0.60162193	1.35E-19	-0.4111452	6.12E-09	-0.9011705
RAB5A	turquoise	0.34652107	1.35E-06	0.12060932	0.10198189	-0.7749362
RAB40C	turquoise	0.41417385	4.61E-09	-0.7331695	1.81E-32	-0.0669739
RAB3IP	turquoise	-0.3588173	5.29E-07	0.29838326	3.70E-05	0.43078247
RAB3GAP2	turquoise	0.55278873	3.40E-16	-0.4175363	3.36E-09	-0.8165877
RAB39B	turquoise	0.80314061	5.08E-43	-0.4039814	1.18E-08	-0.5637476
RAB34	turquoise	-0.5005505	4.01E-13	0.51414595	7.13E-14	0.55425093
RAB31	turquoise	-0.6059268	6.35E-20	0.81965501	3.78E-46	0.67860217
RAB24	turquoise	-0.3200915	8.93E-06	-0.2771295	0.00013405	0.47962249
RAB22A	turquoise	0.73538377	9.46E-33	-0.5027397	3.05E-13	-0.6649699
RAB18	turquoise	0.57793095	7.10E-18	-0.1000525	0.17540493	-0.6559365
RAB15	turquoise	0.53497231	4.35E-15	-0.6953525	4.68E-28	-0.5500086
RAB13	turquoise	-0.5815434	3.96E-18	0.46837152	1.78E-11	0.84165274
RAB11FIP4	turquoise	-0.4134476	4.94E-09	-0.027741	0.7077841	0.67810216
RAB11FIP2	turquoise	0.52379486	2.00E-14	-0.1887729	0.01007149	-0.8263156
RAB11A	turquoise	0.6956647	4.33E-28	-0.5996123	1.92E-19	-0.5431894
R3HDM1	turquoise	0.70516726	3.91E-29	-0.6368289	1.97E-22	-0.7392985
QRICH1	turquoise	0.57993491	5.14E-18	-0.2551193	0.0004572	-0.662393
QPRT	turquoise	-0.3865065	5.51E-08	0.29592472	4.32E-05	0.73184002
QPCT	turquoise	0.54318429	1.37E-15	-0.4583488	5.36E-11	-0.8621874
QARS	turquoise	-0.3203145	8.79E-06	0.06047202	0.41353867	0.76885766
PYROXD2	turquoise	-0.5847893	2.33E-18	0.51699123	4.92E-14	0.74525257
PYGM	turquoise	-0.5375501	3.04E-15	0.47257681	1.11E-11	0.36992649
PYGL	turquoise	-0.4862781	2.26E-12	0.66819366	2.75E-25	0.25977244

PXYLP1	turquoise	0.5215758	2.69E-14	-0.3272314	5.46E-06	-0.8245912
PWP1	turquoise	0.60827966	4.18E-20	-0.4165066	3.70E-09	-0.6844259
PUM2	turquoise	0.58744726	1.50E-18	-0.3387627	2.40E-06	-0.7406446
PTTG1IP	turquoise	-0.5656049	4.93E-17	0.80470006	2.65E-43	0.59644
PTTG1	turquoise	0.57669581	8.65E-18	-0.6671215	3.48E-25	-0.1677091
PTS	turquoise	0.56476573	5.61E-17	-0.3171807	1.09E-05	-0.8508849
PTRH2	turquoise	0.5996609	1.90E-19	-0.4746665	8.74E-12	-0.6440821
PTRF	turquoise	-0.5770304	8.20E-18	0.58193724	3.72E-18	0.80166011
PTPRT	turquoise	0.55245959	3.57E-16	-0.5813369	4.10E-18	-0.7223829
PTPRO	turquoise	0.65428002	5.59E-24	-0.7733978	4.49E-38	-0.2843049
PTPRN2	turquoise	0.27196205	0.0001805	-0.2977287	3.86E-05	-0.8415031
PTPRK	turquoise	0.44985736	1.33E-10	-0.2548415	0.00046403	-0.7490588
PTPRE	turquoise	0.39602122	2.41E-08	-0.2194049	0.00269403	-0.6850608
PTPRA	turquoise	-0.2948334	4.62E-05	0.03954343	0.5930562	0.72698975
PTPN4	turquoise	0.62803108	1.09E-21	-0.6962252	3.77E-28	-0.3763287
PTPN3	turquoise	0.63938915	1.18E-22	-0.710517	9.68E-30	-0.5793154
PTOV1	turquoise	-0.5373676	3.12E-15	0.00800279	0.91390555	0.25654877
PTH2R	turquoise	0.607108	5.15E-20	-0.7564384	1.41E-35	-0.517429
PTH1R	turquoise	-0.612355	2.01E-20	0.60301231	1.06E-19	0.85921738
PTGR1	turquoise	0.48075006	4.33E-12	-0.0857142	0.24601897	-0.7307702
PTGFRN	turquoise	0.60306271	1.05E-19	-0.6037076	9.39E-20	-0.4890408
PTGES3	turquoise	0.38292585	7.47E-08	0.12540809	0.08896275	-0.2868428
PTDSS2	turquoise	-0.6609271	1.35E-24	0.21334941	0.00354727	0.6835928
PTBP1	turquoise	-0.626285	1.52E-21	0.62140637	3.81E-21	0.84951782
PTAR1	turquoise	-0.5154213	6.04E-14	0.78766852	2.39E-40	0.70006864
PSMG4	turquoise	0.55252731	3.53E-16	-0.6519896	9.04E-24	-0.6783329
PSMG1	turquoise	0.69894147	1.91E-28	-0.5367583	3.39E-15	-0.7763473
PSMD7	turquoise	0.48194209	3.77E-12	-0.1269701	0.08502377	-0.6897365
PSMD6	turquoise	0.44110681	3.29E-10	-0.0754025	0.30768722	-0.8247356
PSMD14	turquoise	0.55173368	3.97E-16	-0.2607788	0.00033686	-0.8816134
PSMD12	turquoise	0.5050189	2.29E-13	-0.1360259	0.06486277	-0.7496363
PSMD10	turquoise	0.43772265	4.65E-10	-0.0762433	0.30230894	-0.8504468
PSMD1	turquoise	0.52014874	3.25E-14	-0.5635405	6.76E-17	-0.7301219
PSMC6	turquoise	0.52792962	1.14E-14	-0.1617134	0.02786893	-0.7785979
PSMC2	turquoise	0.51629487	5.39E-14	-0.1373158	0.06233908	-0.841805
PSMC1	turquoise	0.50596753	2.03E-13	-0.757433	1.02E-35	-0.1402552
PSMB8	turquoise	-0.4853158	2.54E-12	0.56313899	7.19E-17	0.62359737
PSMB5	turquoise	0.39415003	2.84E-08	-0.3084116	1.95E-05	-0.723136
PSMB4	turquoise	0.39583828	2.45E-08	-0.5061545	1.99E-13	-0.3443835
PSMB2	turquoise	0.2905602	6.02E-05	-0.5648138	5.56E-17	-0.7207503
PSMB10	turquoise	-0.4321381	8.14E-10	0.2374773	0.00113457	0.81030507
PSMA6	turquoise	0.70934761	1.32E-29	-0.3491988	1.11E-06	-0.613107
PSMA5	turquoise	0.56368188	6.62E-17	-0.4049002	1.09E-08	-0.8062481
PSMA4	turquoise	0.71841026	1.16E-30	-0.5927082	6.24E-19	-0.6330957
PSMA3	turquoise	0.47751186	6.30E-12	-0.0924061	0.21092749	-0.8320447
PSMA1	turquoise	0.63014155	7.26E-22	-0.288997	6.62E-05	-0.8447621

PSD2	turquoise	-0.4878187	1.88E-12	0.69282578	8.72E-28	0.5414796
PRUNE2	turquoise	0.46810779	1.83E-11	-0.3679683	2.56E-07	-0.836902
PRSS53	turquoise	-0.3292052	4.75E-06	-0.0147161	0.84240812	0.68394183
PRSS42	turquoise	-0.3636951	3.61E-07	0.15641963	0.03348474	0.74752604
PRSS3	turquoise	0.33758622	2.61E-06	-0.6558378	4.02E-24	-0.524929
PRRT2	turquoise	-0.3797833	9.73E-08	0.07788115	0.29201125	0.74703431
PRRG3	turquoise	0.5294265	9.34E-15	-0.6703252	1.71E-25	-0.1718299
PRRG2	turquoise	-0.3798684	9.66E-08	0.34766445	1.24E-06	0.8393062
PRRC2C	turquoise	0.21052677	0.00402273	0.04372245	0.55455955	-0.6534598
PRPS2	turquoise	0.69779105	2.55E-28	-0.6081335	4.29E-20	-0.5280662
PRPS1	turquoise	0.65250297	8.12E-24	-0.573963	1.34E-17	-0.8545206
PRPH2	turquoise	0.53794401	2.87E-15	-0.6481699	2.00E-23	-0.496674
PRPF4	turquoise	0.52728509	1.25E-14	-0.4919029	1.15E-12	-0.3688738
PRPF38A	turquoise	-0.368472	2.46E-07	0.53835175	2.71E-15	0.7189378
PRPF31	turquoise	-0.4400512	3.67E-10	0.03664196	0.62047808	0.45466497
PRPF18	turquoise	0.71967956	8.22E-31	-0.1551482	0.03496817	-0.436569
PROSER1	turquoise	0.35151172	9.29E-07	0.06088373	0.41036043	-0.7857877
PROSC	turquoise	0.4508464	1.20E-10	-0.0584939	0.42900952	-0.6921731
PRNP	turquoise	0.6203109	4.67E-21	-0.542405	1.53E-15	-0.7136646
PRMT8	turquoise	0.57117738	2.07E-17	-0.8077053	7.44E-44	-0.1932338
PRMT6	turquoise	0.66712341	3.48E-25	-0.3706149	2.07E-07	-0.7145285
PRMT2	turquoise	-0.5587347	1.40E-16	0.41211802	5.59E-09	0.85491185
PRKX	turquoise	-0.521027	2.89E-14	0.53730275	3.14E-15	0.78168525
PRKRA	turquoise	0.75523109	2.08E-35	-0.403502	1.23E-08	-0.4207346
PRKDC	turquoise	0.699724	1.57E-28	-0.2562311	0.00043081	-0.6547303
PRKCI	turquoise	0.74676077	3.04E-34	-0.5520395	3.79E-16	-0.2639303
PRKCH	turquoise	-0.4977496	5.66E-13	0.8132007	6.88E-45	0.41806806
PRKCE	turquoise	0.71455245	3.31E-30	-0.6793766	2.16E-26	-0.6251938
PRKCB	turquoise	0.65953044	1.83E-24	-0.6230805	2.78E-21	-0.789607
PRKAR1A	turquoise	0.46872575	1.71E-11	-0.1399558	0.05742466	-0.8731957
PRKACB	turquoise	0.59129943	7.91E-19	-0.3975317	2.11E-08	-0.7695634
PRICKLE2	turquoise	0.46101555	4.01E-11	-0.5021203	3.30E-13	-0.4821304
PREX1	turquoise	-0.6827775	9.76E-27	0.5241193	1.91E-14	0.43375165
PREPL	turquoise	0.70202726	8.75E-29	-0.6871665	3.44E-27	-0.6602873
PREP	turquoise	0.66028871	1.55E-24	-0.6386707	1.37E-22	-0.6910442
PRDX6	turquoise	-0.4509181	1.19E-10	0.77062786	1.19E-37	0.51772974
PRDX3	turquoise	0.29576328	4.36E-05	0.14460503	0.04954813	-0.7724983
PRDM8	turquoise	0.30389532	2.61E-05	-0.4640336	2.88E-11	-0.515257
PRDM2	turquoise	0.4906807	1.34E-12	-0.8059323	1.58E-43	-0.1471632
PRDM16	turquoise	-0.4381992	4.43E-10	0.76246634	1.93E-36	0.47368192
PRDM10	turquoise	0.60118347	1.46E-19	-0.2319105	0.00149134	-0.6788673
PRC1	turquoise	0.59872809	2.23E-19	-0.7415854	1.48E-33	-0.5934911
PRAM1	turquoise	-0.4921088	1.13E-12	0.18429302	0.01203167	0.44562437
PPT1	turquoise	0.53564411	3.96E-15	0.00178236	0.98079005	-0.6249875
PPP6R3	turquoise	-0.3484422	1.17E-06	0.10489104	0.15533848	0.79643603
PPP6R2	turquoise	-0.5096131	1.28E-13	0.41095703	6.23E-09	0.76032432

PPP6R1	turquoise	-0.555142	2.40E-16	0.03874833	0.60051585	0.48974571
PPP4R4	turquoise	0.68282167	9.66E-27	-0.4771059	6.61E-12	-0.6998582
PPP4R1	turquoise	-0.4139881	4.70E-09	0.64125353	8.16E-23	0.66183936
PPP3R1	turquoise	0.55765691	1.65E-16	-0.670731	1.56E-25	-0.5409971
PPP3CB	turquoise	0.67945454	2.12E-26	-0.7666609	4.67E-37	-0.6930292
PPP3CA	turquoise	0.71867405	1.08E-30	-0.5439919	1.22E-15	-0.6652339
PPP2R2C	turquoise	0.48665903	2.16E-12	-0.5552639	2.35E-16	-0.3088752
PPP2R2B	turquoise	0.66657653	3.93E-25	-0.5302678	8.32E-15	-0.7366306
PPP2CA	turquoise	0.60736515	4.92E-20	-0.588829	1.20E-18	-0.5839599
PPP1R3F	turquoise	0.3021324	2.92E-05	-0.6531714	7.06E-24	-0.3925556
PPP1R3C	turquoise	-0.4200011	2.66E-09	0.64757631	2.26E-23	0.43955186
PPP1R26	turquoise	-0.2602558	0.0003466	0.16805302	0.02222139	0.76143364
PPP1R2	turquoise	0.70311081	6.63E-29	-0.5559177	2.14E-16	-0.7210848
PPP1R1A	turquoise	-0.4931429	9.94E-13	0.19135459	0.0090749	0.66883112
PPP1R16B	turquoise	-0.4211472	2.38E-09	0.35277571	8.44E-07	0.40971901
PPP1R14C	turquoise	0.6925777	9.27E-28	-0.732365	2.29E-32	-0.4680498
PPP1R14B	turquoise	-0.7156931	2.43E-30	0.54115777	1.83E-15	0.62960737
PPP1R12A	turquoise	0.61390092	1.52E-20	-0.1533155	0.03720421	-0.6361369
PPM1M	turquoise	-0.3361157	2.90E-06	0.05351221	0.46941566	0.7424247
PPM1L	turquoise	0.62761842	1.18E-21	-0.656215	3.71E-24	-0.434415
PPM1H	turquoise	0.65195179	9.11E-24	-0.8142656	4.29E-45	-0.4658632
PPM1A	turquoise	0.51712683	4.84E-14	-0.2123061	0.00371677	-0.7144663
PPL	turquoise	0.66371853	7.37E-25	-0.657702	2.70E-24	-0.6830779
PPIL1	turquoise	0.42747552	1.29E-09	-0.0756208	0.30628506	-0.7910544
PPIA	turquoise	0.68623467	4.30E-27	-0.3703527	2.12E-07	-0.6571944
PPFIBP2	turquoise	-0.5852666	2.16E-18	0.5689123	2.95E-17	0.73967243
PPEF1	turquoise	0.68803086	2.80E-27	-0.7381335	4.19E-33	-0.4201565
PPCS	turquoise	0.42093261	2.43E-09	-0.1390899	0.05899996	-0.2676556
PPARA	turquoise	-0.4188531	2.97E-09	0.80148166	1.01E-42	0.53636913
PPA1	turquoise	0.36376361	3.59E-07	-0.0420299	0.57000441	-0.837406
POU3F2	turquoise	-0.3757333	1.36E-07	0.74410738	6.88E-34	0.52448087
POU2F1	turquoise	-0.5191593	3.70E-14	0.20779258	0.00453724	0.89808804
POPDC3	turquoise	0.53986291	2.19E-15	-0.6349842	2.83E-22	-0.1821206
POP7	turquoise	0.46744049	1.97E-11	-0.7102235	1.05E-29	-0.7048826
POMT2	turquoise	-0.6651974	5.33E-25	0.40744142	8.61E-09	0.57500258
POMP	turquoise	0.35859567	5.38E-07	0.20827551	0.00444228	-0.3989167
POMGNT2	turquoise	0.33599458	2.93E-06	-0.2232097	0.00225791	-0.826984
POLR3H	turquoise	-0.50376	2.68E-13	0.6068756	5.37E-20	0.78969055
POLR3C	turquoise	0.4747271	8.68E-12	-0.6187259	6.27E-21	-0.2362085
POLR3B	turquoise	0.64558984	3.39E-23	-0.5377372	2.96E-15	-0.3463319
POLR2L	turquoise	-0.4626022	3.37E-11	0.47384249	9.60E-12	0.5700209
POLR2J4	turquoise	-0.3426776	1.80E-06	0.30825306	1.97E-05	0.53064662
POLR2B	turquoise	0.48716253	2.04E-12	-0.1559334	0.03404563	-0.8940661
POLN	turquoise	-0.4685638	1.74E-11	0.16385021	0.02584173	0.65200677
POLDIP3	turquoise	-0.6809304	1.51E-26	0.60354686	9.66E-20	0.4206925
POLD2	turquoise	-0.5333187	5.47E-15	0.1402594	0.05688054	0.59407477

POLA1	turquoise	-0.3633965	3.69E-07	0.60230939	1.20E-19	0.6977202
POGK	turquoise	-0.5314436	7.09E-15	0.60372329	9.36E-20	0.47278974
PODN	turquoise	-0.5204807	3.11E-14	0.46851398	1.75E-11	0.76398482
PNPLA8	turquoise	0.23170779	0.00150609	0.32974316	4.57E-06	-0.3605067
PNPLA7	turquoise	-0.471898	1.20E-11	0.03325147	0.65319522	0.63478056
PNP	turquoise	-0.3053397	2.38E-05	0.59401584	5.00E-19	0.28231642
PNN	turquoise	-0.3965224	2.30E-08	0.59185515	7.20E-19	0.18994313
PNMAL1	turquoise	0.7286201	6.72E-32	-0.7606373	3.55E-36	-0.539045
PNMA3	turquoise	0.35545246	6.87E-07	-0.7270028	1.06E-31	-0.1263706
PNMA2	turquoise	0.69039935	1.58E-27	-0.6519442	9.13E-24	-0.7776489
PNMA1	turquoise	0.6191291	5.82E-21	-0.3458315	1.42E-06	-0.8866499
PNKP	turquoise	-0.2758281	0.00014456	-0.2896017	6.38E-05	0.5601521
PMVK	turquoise	-0.5282842	1.09E-14	0.40172011	1.45E-08	0.53377848
PMS1	turquoise	0.7048889	4.20E-29	-0.2646885	0.00027168	-0.316508
PMPCB	turquoise	0.74442761	6.24E-34	-0.4809873	4.21E-12	-0.5880212
PMPCA	turquoise	-0.4840801	2.93E-12	0.05795787	0.43325829	0.81168198
PMP22	turquoise	-0.4045357	1.12E-08	0.59387206	5.12E-19	0.40337073
PMP2	turquoise	-0.2986949	3.63E-05	0.49944365	4.60E-13	0.26580477
PML	turquoise	-0.3637828	3.58E-07	0.10142231	0.16953875	0.7959158
PMEPA1	turquoise	-0.3077384	2.03E-05	0.12670079	0.0856928	0.61157378
PLXNB2	turquoise	-0.7007663	1.21E-28	0.52220451	2.47E-14	0.39999941
PLXNB1	turquoise	-0.7172792	1.58E-30	0.59448661	4.61E-19	0.75378309
PLXNA3	turquoise	-0.5964778	3.29E-19	0.21648257	0.00307936	0.61620035
PLXNA1	turquoise	-0.2944972	4.72E-05	-0.1675294	0.02264719	0.36577436
PLS3	turquoise	0.3697675	2.22E-07	0.16479601	0.02498566	-0.6825469
PLPPR5	turquoise	0.60667279	5.57E-20	-0.7059059	3.23E-29	-0.1730503
PLPPR4	turquoise	0.70849466	1.65E-29	-0.6728967	9.56E-26	-0.6482783
PLPP6	turquoise	0.6661988	4.27E-25	-0.4731937	1.03E-11	-0.6392465
PLOD2	turquoise	-0.3596863	4.95E-07	0.77447	3.07E-38	0.29608206
PLK2	turquoise	0.67381363	7.76E-26	-0.8001764	1.72E-42	-0.5423498
PLIN3	turquoise	-0.5609854	9.98E-17	0.66716063	3.45E-25	0.53361541
PLIN2	turquoise	-0.4417105	3.10E-10	0.73446681	1.24E-32	0.17910706
PLEKHO2	turquoise	-0.6019811	1.27E-19	0.64467232	4.09E-23	0.71331654
PLEKHM2	turquoise	-0.7117027	7.07E-30	0.59332837	5.62E-19	0.59604429
PLEKHB1	turquoise	-0.7322171	2.39E-32	0.46502483	2.58E-11	0.21900359
PLEKHA5	turquoise	0.7602502	4.03E-36	-0.450353	1.26E-10	-0.3187777
PLEKHA4	turquoise	-0.47811	5.88E-12	0.4127799	5.26E-09	0.67454287
PLCXD3	turquoise	0.69889711	1.93E-28	-0.7590883	5.91E-36	-0.5143806
PLCL2	turquoise	0.62933707	8.48E-22	-0.8045584	2.81E-43	-0.5002929
PLCH1	turquoise	0.68114237	1.43E-26	-0.4813254	4.05E-12	-0.4162067
PLCG2	turquoise	-0.262583	0.00030516	0.71367566	4.18E-30	0.27053813
PLCG1	turquoise	-0.5190283	3.77E-14	0.68530702	5.36E-27	0.66618433
PLCE1	turquoise	-0.4392926	3.96E-10	0.74786428	2.16E-34	0.55143553
PLCD3	turquoise	-0.5022818	3.23E-13	0.41880374	2.98E-09	0.60784382
PLCB1	turquoise	0.54191862	1.64E-15	-0.6367926	1.98E-22	-0.3984955
PLAC9	turquoise	-0.4039815	1.18E-08	0.45668806	6.41E-11	0.40716566

PLA1A	turquoise	-0.5174287	4.65E-14	0.59898243	2.14E-19	0.32917412
PKP4	turquoise	-0.5355953	3.99E-15	0.32202348	7.82E-06	0.46151824
PKN2	turquoise	-0.389622	4.21E-08	0.48603799	2.33E-12	0.57834177
PKIA	turquoise	0.49277396	1.04E-12	-0.2802471	0.00011171	-0.4385582
PJA2	turquoise	0.53582191	3.87E-15	-0.5130825	8.19E-14	-0.5996945
PJA1	turquoise	0.55338717	3.11E-16	-0.4422012	2.94E-10	-0.9311192
PITHD1	turquoise	0.62817884	1.06E-21	-0.7050669	4.01E-29	-0.7970323
PIP4K2C	turquoise	0.61933156	5.61E-21	-0.6463866	2.88E-23	-0.6313889
PIP4K2A	turquoise	-0.5143131	6.98E-14	0.24942793	0.00061736	0.66617311
PIK3CD	turquoise	-0.3265855	5.71E-06	0.17183422	0.01934681	0.72753439
PIK3CB	turquoise	0.60267166	1.13E-19	-0.3666973	2.84E-07	-0.8680698
PIGZ	turquoise	0.40412266	1.17E-08	-0.6396497	1.12E-22	-0.6693789
PIGY	turquoise	0.19618183	0.00744325	0.27473997	0.00015394	-0.6918567
PIGP	turquoise	0.38128234	8.58E-08	0.02856131	0.69955479	-0.6760793
PIGK	turquoise	0.40972752	6.98E-09	0.02725769	0.71264935	-0.6998956
PIGH	turquoise	0.62310578	2.77E-21	-0.068602	0.35348133	-0.3106874
PIGB	turquoise	0.47033158	1.43E-11	-0.1531477	0.03741478	-0.7156122
PIEZO1	turquoise	-0.6690693	2.26E-25	0.57161313	1.94E-17	0.84319213
PIDD1	turquoise	-0.5647551	5.62E-17	0.15000599	0.041548	0.42581347
PID1	turquoise	0.40297629	1.29E-08	-0.0741637	0.31572573	-0.8201903
PI16	turquoise	-0.4939793	8.98E-13	0.42330536	1.93E-09	0.66324295
PHYHD1	turquoise	-0.4535964	8.93E-11	0.45169588	1.09E-10	0.28969593
PHYH	turquoise	0.48689318	2.10E-12	-0.0394344	0.59407651	-0.3355453
PHTF1	turquoise	0.61704832	8.55E-21	-0.4177509	3.29E-09	-0.56525
PHRF1	turquoise	-0.4421735	2.95E-10	0.31196446	1.54E-05	0.69891839
PHLPP2	turquoise	0.60432989	8.42E-20	-0.2961332	4.26E-05	-0.8181057
PHLPP1	turquoise	-0.4691064	1.64E-11	0.5249788	1.71E-14	0.32221026
PHKG1	turquoise	-0.3834565	7.14E-08	0.44718702	1.76E-10	0.29749524
PHKB	turquoise	0.34553091	1.46E-06	0.16953326	0.02105494	-0.6815548
PHKA2	turquoise	-0.476051	7.46E-12	0.269418	0.00020853	0.88931923
PHGDH	turquoise	-0.5831991	3.03E-18	0.77894799	6.14E-39	0.6181205
PHF24	turquoise	0.47092841	1.33E-11	-0.7855662	5.30E-40	-0.6004147
PHF21B	turquoise	-0.5631113	7.22E-17	0.66084896	1.37E-24	0.63234837
PHF2	turquoise	-0.5899293	9.95E-19	0.61870637	6.30E-21	0.6810214
PHF19	turquoise	-0.6541236	5.78E-24	0.55317288	3.21E-16	0.77307886
PHF14	turquoise	0.68839594	2.56E-27	-0.6265132	1.46E-21	-0.8110876
PHF11	turquoise	-0.3478723	1.22E-06	0.60752066	4.79E-20	0.46353267
PHB	turquoise	-0.5329229	5.78E-15	0.77665676	1.41E-38	0.66824034
PHACTR4	turquoise	-0.3828614	7.51E-08	0.70610873	3.07E-29	0.59649861
PHACTR3	turquoise	-0.5855386	2.06E-18	0.6237812	2.44E-21	0.34624361
PGRMC1	turquoise	0.65728086	2.96E-24	-0.5583346	1.49E-16	-0.8115909
PGM5	turquoise	-0.378692	1.07E-07	0.67351498	8.31E-26	0.52138366
PGGHG	turquoise	-0.5449383	1.06E-15	0.45929077	4.84E-11	0.48999274
PGBD1	turquoise	0.64763898	2.23E-23	-0.465823	2.36E-11	-0.5461874
PGAP1	turquoise	0.64227544	6.64E-23	-0.4368603	5.07E-10	-0.7398347
PFN2	turquoise	0.61263679	1.91E-20	-0.6123189	2.03E-20	-0.9010355

PFN1	turquoise	-0.4058923	9.93E-09	0.27395836	0.00016102	0.67984093
PFKM	turquoise	0.64270444	6.09E-23	-0.2491217	0.0006273	-0.6075444
PFKFB4	turquoise	-0.27733	0.0001325	0.27091445	0.00019159	0.8343012
PFDN5	turquoise	-0.4241207	1.79E-09	0.38146669	8.45E-08	0.64332806
PFDN4	turquoise	0.7248743	1.94E-31	-0.6007852	1.56E-19	-0.4664759
PEX7	turquoise	0.56056142	1.06E-16	-0.2553801	0.00045088	-0.6270537
PEX6	turquoise	-0.3404105	2.13E-06	0.16088575	0.02868981	0.28593587
PEX19	turquoise	0.53515379	4.24E-15	-0.4891231	1.61E-12	-0.3854776
PEG10	turquoise	0.53284276	5.84E-15	-0.5295189	9.22E-15	-0.7504381
PDS5B	turquoise	0.69507772	5.01E-28	-0.6580909	2.49E-24	-0.4658244
PDPN	turquoise	-0.2787008	0.00012231	0.37524639	1.42E-07	0.57679099
PDPK1	turquoise	0.57800242	7.02E-18	-0.4068045	9.13E-09	-0.7035542
PDP1	turquoise	0.74402806	7.05E-34	-0.6277641	1.15E-21	-0.6971671
PDK3	turquoise	0.6661384	4.33E-25	-0.7934489	2.55E-41	-0.5299378
PDIK1L	turquoise	0.75115476	7.66E-35	-0.294697	4.66E-05	-0.6069146
PDHX	turquoise	0.55444489	2.66E-16	-0.2950404	4.56E-05	-0.8722838
PDHB	turquoise	0.51408187	7.19E-14	-0.3328995	3.66E-06	-0.7867502
PDGFRB	turquoise	-0.6697455	1.94E-25	0.79124371	6.04E-41	0.69508953
PDGFD	turquoise	0.74482556	5.52E-34	-0.5737438	1.38E-17	-0.6197347
PDE5A	turquoise	-0.4631269	3.18E-11	0.68315983	8.92E-27	0.73335948
PDE4DIP	turquoise	-0.2005051	0.00620918	-0.1014715	0.16933069	0.58131553
PDE4D	turquoise	0.61847813	6.57E-21	-0.2206055	0.0025488	-0.7606366
PDE1A	turquoise	0.55561392	2.24E-16	-0.687972	2.84E-27	-0.2520049
PDE12	turquoise	0.43388017	6.84E-10	-0.0632717	0.39221215	-0.6364475
PDDC1	turquoise	-0.5196703	3.46E-14	0.2120471	0.00375995	0.80809206
PDCD6IP	turquoise	0.57406382	1.32E-17	-0.341555	1.95E-06	-0.7623823
PDCD6	turquoise	0.51908882	3.74E-14	-0.2278523	0.00181309	-0.8582858
PDCD2L	turquoise	0.52725273	1.25E-14	-0.5057398	2.09E-13	-0.7770147
PDCD10	turquoise	0.52452541	1.81E-14	0.01045459	0.88768207	-0.6842661
PCYOX1L	turquoise	0.69025463	1.63E-27	-0.7794886	5.04E-39	-0.5060703
PCSK7	turquoise	-0.5784281	6.56E-18	-0.0664181	0.36905025	0.50866874
PCSK2	turquoise	0.84385336	2.32E-51	-0.5681109	3.35E-17	-0.5923004
PCSK1	turquoise	0.70677357	2.58E-29	-0.7119023	6.71E-30	-0.6646216
PCP4	turquoise	0.39940192	1.78E-08	-0.6123009	2.03E-20	-0.2102786
PCNP	turquoise	0.45502478	7.67E-11	0.03472054	0.63893311	-0.7413945
PCMT1	turquoise	0.73011732	4.38E-32	-0.6922135	1.01E-27	-0.6561582
PCLO	turquoise	0.64153943	7.70E-23	-0.6137139	1.57E-20	-0.7639001
PCF11	turquoise	-0.3584628	5.44E-07	0.64416231	4.53E-23	0.61541786
PCED1A	turquoise	-0.53027	8.32E-15	0.3467522	1.33E-06	0.55105164
PCDHB4	turquoise	0.19573418	0.00758273	0.04943993	0.50393775	-0.7021771
PCDHB10	turquoise	0.69523376	4.82E-28	-0.3456731	1.44E-06	-0.592362
PCDHA3	turquoise	0.58744476	1.50E-18	-0.4790219	5.29E-12	-0.8647834
PCDH8	turquoise	0.72860912	6.74E-32	-0.6799324	1.90E-26	-0.7217408
PCDH20	turquoise	0.663574	7.60E-25	-0.5494462	5.54E-16	-0.7149324
PCDH19	turquoise	0.69837564	2.20E-28	-0.7484928	1.77E-34	-0.4904759
PCDH18	turquoise	-0.1823609	0.01297577	0.65751223	2.82E-24	0.35581835

PCDH10	turquoise	0.33595219	2.94E-06	-0.0712338	0.33527546	-0.7977705
PCBP1	turquoise	0.24066759	0.00096725	0.17691514	0.01599498	-0.6895976
PC	turquoise	-0.712728	5.39E-30	0.45573653	7.10E-11	0.31349872
PBXIP1	turquoise	-0.6298654	7.66E-22	0.54413306	1.19E-15	0.41174949
PAXIP1-AS2	turquoise	-0.5176728	4.50E-14	0.60950317	3.36E-20	0.80478888
PARVG	turquoise	-0.5178835	4.38E-14	0.53995215	2.17E-15	0.28398881
PARP9	turquoise	-0.4587095	5.15E-11	0.80933185	3.71E-44	0.20933515
PARP4	turquoise	-0.3542573	7.53E-07	0.85089941	4.77E-53	0.1251958
PARP14	turquoise	-0.4521784	1.04E-10	0.56600271	4.63E-17	0.72157406
PARP12	turquoise	-0.4875101	1.96E-12	0.38313281	7.34E-08	0.6657925
PARM1	turquoise	0.64425247	4.45E-23	-0.7523339	5.27E-35	-0.665324
PARG	turquoise	0.72167755	4.74E-31	-0.6845166	6.47E-27	-0.6032879
PAQR8	turquoise	-0.5129033	8.38E-14	0.60557847	6.76E-20	0.73825785
PAQR6	turquoise	-0.6367904	1.98E-22	0.40107209	1.54E-08	0.5248709
PAPSS1	turquoise	0.63399129	3.44E-22	-0.2749911	0.00015173	-0.2741359
PAPOLA	turquoise	-0.2421285	0.00089846	0.58862787	1.24E-18	0.63792267
PAPLN	turquoise	-0.3464036	1.36E-06	0.56438129	5.95E-17	0.59484146
PAPD4	turquoise	0.54215186	1.59E-15	0.02094696	0.77716956	-0.6649401
PAOX	turquoise	-0.5470043	7.90E-16	0.43363635	7.01E-10	0.6392357
PAN2	turquoise	-0.4709007	1.34E-11	0.50102998	3.78E-13	0.51444345
PAM	turquoise	0.72935325	5.45E-32	-0.6426503	6.16E-23	-0.6348736
PALM	turquoise	-0.627559	1.19E-21	0.38284817	7.52E-08	0.72381828
PALD1	turquoise	-0.5384187	2.69E-15	0.62369202	2.48E-21	0.64437795
PAK5	turquoise	0.68894156	2.24E-27	-0.6111883	2.48E-20	-0.7418316
PAK4	turquoise	-0.7349642	1.07E-32	0.38342523	7.16E-08	0.45875902
PAK1	turquoise	0.66011416	1.61E-24	-0.7766581	1.41E-38	-0.7577993
PAIP2	turquoise	0.47143517	1.26E-11	-0.0891267	0.22764761	-0.8904155
PAIP1	turquoise	0.65700563	3.14E-24	-0.5916468	7.46E-19	-0.6425769
PAFAH1B1	turquoise	0.64157452	7.65E-23	-0.4809902	4.21E-12	-0.8519815
PAF1	turquoise	-0.4259964	1.49E-09	-0.0365514	0.62134258	0.6901324
PACSIN3	turquoise	-0.5815172	3.98E-18	0.31230501	1.51E-05	0.67412002
PACSIN2	turquoise	-0.518831	3.87E-14	0.51891666	3.82E-14	0.67762246
PACSIN1	turquoise	0.49236791	1.09E-12	-0.6638064	7.23E-25	-0.2611205
PABPN1	turquoise	-0.3827914	7.55E-08	-0.0192696	0.7945999	0.47022807
PABPC1L2B	turquoise	0.59259889	6.35E-19	-0.7236376	2.75E-31	-0.6727157
PABPC1L2A	turquoise	0.51651992	5.24E-14	-0.7733418	4.58E-38	-0.5817626
PABPC1	turquoise	-0.3991969	1.82E-08	0.49408482	8.87E-13	0.0342208
P3H1	turquoise	-0.5428057	1.44E-15	0.1730788	0.01847396	0.81105586
OXR1	turquoise	0.66824013	2.72E-25	-0.461604	3.76E-11	-0.7908079
OXCT1	turquoise	0.65204668	8.93E-24	-0.3876023	5.01E-08	-0.7362245
OTUD6B	turquoise	0.62276308	2.96E-21	-0.048466	0.51238642	-0.3776696
OTOS	turquoise	-0.4201366	2.62E-09	0.40483313	1.09E-08	0.32424226
OTOGL	turquoise	-0.4335373	7.08E-10	0.25875099	0.00037612	0.80541602
OSTF1	turquoise	0.53632586	3.60E-15	-0.3249902	6.38E-06	-0.7292536
OSTC	turquoise	0.27363456	0.00016404	0.34308294	1.75E-06	-0.4368955
OSBPL7	turquoise	-0.5768111	8.50E-18	0.12122624	0.10022841	0.38705548

OSBPL3	turquoise	0.65059036	1.21E-23	-0.4763084	7.24E-12	-0.5811583
OSBPL10	turquoise	0.70069036	1.23E-28	-0.6452867	3.61E-23	-0.7472163
OSBP2	turquoise	-0.2567422	0.00041915	-0.1831704	0.01257258	0.38432678
ORMDL3	turquoise	-0.5422675	1.56E-15	0.31161642	1.58E-05	0.68121559
ORC5	turquoise	0.57713059	8.07E-18	-0.744302	6.49E-34	-0.5693159
OR2L13	turquoise	0.55203044	3.80E-16	-0.5185898	3.99E-14	-0.533074
OPTN	turquoise	0.66685784	3.69E-25	-0.5182556	4.17E-14	-0.638587
OPRK1	turquoise	0.61071208	2.71E-20	-0.5269353	1.31E-14	-0.4940292
OPN3	turquoise	0.6351549	2.74E-22	-0.5918721	7.18E-19	-0.8169337
OPLAH	turquoise	-0.3480392	1.21E-06	0.15594274	0.03403478	0.58196976
OPCML	turquoise	0.6504888	1.24E-23	-0.7090512	1.42E-29	-0.6145166
OPA1	turquoise	0.66831212	2.68E-25	-0.6007221	1.58E-19	-0.8432476
OMG	turquoise	0.40504668	1.07E-08	-0.1132772	0.12472515	-0.8181731
OLMALINC	turquoise	-0.5498063	5.26E-16	0.48214788	3.68E-12	0.32188032
OLIG2	turquoise	-0.5899812	9.86E-19	0.4725757	1.11E-11	0.53788913
OLIG1	turquoise	-0.6217755	3.56E-21	0.65326764	6.92E-24	0.46875668
OLFM4	turquoise	0.31976812	9.13E-06	-0.2655886	0.00025844	-0.1415618
OLFM3	turquoise	0.66886198	2.37E-25	-0.6155668	1.12E-20	-0.7568701
OLFM1	turquoise	0.4938003	9.18E-13	-0.6306725	6.55E-22	-0.6227475
OLA1	turquoise	0.71241557	5.85E-30	-0.6394255	1.18E-22	-0.6493219
OGFOD1	turquoise	0.48278395	3.42E-12	-0.722556	3.71E-31	-0.3224264
OCRL	turquoise	0.60528404	7.12E-20	-0.7186451	1.09E-30	-0.5735642
OCIAD2	turquoise	0.50104207	3.77E-13	-0.3760446	1.33E-07	-0.4939352
OCA2	turquoise	0.60058377	1.62E-19	-0.5248706	1.73E-14	-0.7006397
OAZ1	turquoise	0.50914335	1.36E-13	-0.5209391	2.93E-14	-0.616845
OAT	turquoise	0.50975387	1.26E-13	-0.2173284	0.002963	-0.8756181
OAS2	turquoise	-0.3081207	1.98E-05	0.35776832	5.74E-07	0.53307778
NXPH1	turquoise	0.65273032	7.74E-24	-0.3214011	8.16E-06	-0.6871681
NXNL1	turquoise	-0.2950691	4.56E-05	0.29878397	3.61E-05	0.69488622
NXN	turquoise	-0.5833603	2.95E-18	0.58402333	2.64E-18	0.74447604
NWD2	turquoise	0.74879894	1.61E-34	-0.6050046	7.48E-20	-0.6140702
NUPR1	turquoise	-0.6643266	6.45E-25	0.72836277	7.23E-32	0.50549324
NUPL2	turquoise	0.48965359	1.51E-12	-0.0697129	0.34572233	-0.5416474
NUP54	turquoise	0.56622573	4.48E-17	-0.0610994	0.40870098	-0.445828
NUP50	turquoise	0.3296535	4.60E-06	-0.2567332	0.00041935	-0.4656957
NUP188	turquoise	-0.5452149	1.02E-15	0.52486605	1.73E-14	0.72756921
NUP153	turquoise	0.41109502	6.15E-09	-0.02303	0.75567947	-0.8478048
NUP133	turquoise	0.46074861	4.13E-11	-0.0508184	0.49210548	-0.5450861
NUMA1	turquoise	-0.6148424	1.28E-20	0.15003028	0.04151462	0.54104939
NUDT9	turquoise	0.31559091	1.21E-05	0.10482491	0.15560056	-0.6481515
NUDT7	turquoise	0.41304111	5.13E-09	-0.0437607	0.55421271	-0.3316591
NUDT3	turquoise	-0.6055742	6.76E-20	0.25855499	0.00038013	0.60861962
NUDT21	turquoise	0.70975955	1.18E-29	-0.3878467	4.91E-08	-0.8305424
NUDT2	turquoise	0.2920806	5.48E-05	-0.3781541	1.11E-07	-0.4510307
NUDT16	turquoise	-0.6147144	1.31E-20	0.35847598	5.44E-07	0.59622801
NUDT15	turquoise	-0.3813592	8.52E-08	0.69282214	8.73E-28	0.64834343

NUDT11	turquoise	0.67029704	1.72E-25	-0.7115269	7.41E-30	-0.6931384
NUDT10	turquoise	0.73736645	5.26E-33	-0.5633541	6.96E-17	-0.3842116
NUDT1	turquoise	-0.388444	4.66E-08	0.39778978	2.06E-08	0.65899568
NUAK1	turquoise	0.63020575	7.17E-22	-0.7417338	1.42E-33	-0.4734613
NTPCR	turquoise	0.32540931	6.19E-06	-0.3717218	1.89E-07	-0.7327119
NTNG1	turquoise	0.68512423	5.60E-27	-0.6017848	1.31E-19	-0.6426721
NTM	turquoise	0.37339379	1.65E-07	-0.3932951	3.06E-08	-0.0287516
NT5C3A	turquoise	0.40694873	9.01E-09	-0.2634368	0.00029115	-0.5359751
NT5C2	turquoise	-0.3320987	3.87E-06	0.6513034	1.04E-23	0.59372475
NSUN5	turquoise	-0.6208063	4.26E-21	0.18318579	0.01256503	0.75589946
NSRP1	turquoise	-0.2940306	4.86E-05	0.5620518	8.49E-17	0.51763305
NSMF	turquoise	-0.39427	2.81E-08	0.26615933	0.00025035	0.80445822
NSL1	turquoise	-0.4272196	1.32E-09	0.45976622	4.60E-11	0.51124446
NSG1	turquoise	0.67277689	9.82E-26	-0.7223751	3.90E-31	-0.6575212
NSF	turquoise	0.66586776	4.60E-25	-0.7631447	1.54E-36	-0.7113639
NSA2	turquoise	0.19708314	0.0071693	0.20804556	0.00448727	-0.5932735
NRXN3	turquoise	0.85318358	1.30E-53	-0.68137	1.36E-26	-0.4905913
NRXN1	turquoise	0.69725467	2.92E-28	-0.6130872	1.76E-20	-0.7202241
NRP1	turquoise	0.17202412	0.01921137	0.32372965	6.96E-06	-0.4772227
NRN1	turquoise	0.69026453	1.63E-27	-0.7121407	6.30E-30	-0.5984136
NRIP1	turquoise	0.63046705	6.82E-22	-0.341395	1.98E-06	-0.5119657
NREP	turquoise	0.54138053	1.77E-15	-0.6364309	2.13E-22	-0.6128281
NRDE2	turquoise	-0.4771086	6.60E-12	0.20479015	0.00516968	0.89167641
NRCAM	turquoise	0.70173334	9.43E-29	-0.3349196	3.17E-06	-0.3987204
NR3C2	turquoise	0.57159421	1.94E-17	-0.192965	0.00849843	-0.415474
NR2F1	turquoise	-0.6309016	6.27E-22	0.49028481	1.40E-12	0.72614179
NR2E1	turquoise	-0.4523685	1.02E-10	0.7273523	9.64E-32	0.43308632
NR1D2	turquoise	0.51338612	7.87E-14	-0.1298579	0.07811073	-0.6123772
NPY5R	turquoise	-0.4018774	1.43E-08	0.19756129	0.00702765	0.7784381
NPY	turquoise	0.42294405	2.00E-09	-0.4845227	2.79E-12	-0.6310218
NPTX2	turquoise	0.53180322	6.74E-15	-0.6352987	2.66E-22	-0.6822302
NPTX1	turquoise	0.44600319	1.99E-10	-0.6917108	1.15E-27	-0.2874658
NPTN	turquoise	0.65303947	7.25E-24	-0.7029874	6.85E-29	-0.6442438
NPIPB3	turquoise	-0.6493889	1.55E-23	0.24413863	0.00081116	0.61429193
NPHP3	turquoise	0.18681693	0.01088967	0.1055972	0.15256063	-0.5030203
NPFF	turquoise	-0.4394086	3.92E-10	0.31219421	1.52E-05	0.78358407
NPEPL1	turquoise	-0.5103197	1.17E-13	0.27610422	0.00014227	0.79788401
NPAS3	turquoise	-0.4479194	1.63E-10	0.6760361	4.67E-26	0.3020001
NOVA1	turquoise	0.60813723	4.29E-20	-0.2610488	0.00033193	-0.7154338
NOV	turquoise	0.65905544	2.02E-24	-0.3773438	1.19E-07	-0.6392829
NOTCH4	turquoise	-0.6685945	2.51E-25	0.49776522	5.65E-13	0.69541017
NOTCH2NL	turquoise	-0.3060452	2.27E-05	0.52420978	1.89E-14	0.58027208
NOSTRIN	turquoise	-0.3890843	4.41E-08	0.56595977	4.67E-17	0.50008997
NOS3	turquoise	-0.5013798	3.61E-13	0.59200697	7.02E-19	0.70572713
NOP56	turquoise	-0.5307962	7.74E-15	0.12497804	0.09007244	0.72317464
NOP2	turquoise	-0.3903618	3.95E-08	0.13034345	0.07699433	0.75106294

NOMO2	turquoise	0.44674785	1.84E-10	-0.7310713	3.32E-32	-0.5174355
NOL4	turquoise	0.67379787	7.79E-26	-0.7058792	3.25E-29	-0.5279615
NOL12	turquoise	-0.6469885	2.55E-23	0.41037326	6.58E-09	0.74169505
NOA1	turquoise	0.43409477	6.70E-10	-0.1388268	0.05948565	-0.8203357
NNT	turquoise	0.36344404	3.68E-07	0.0464907	0.52974462	-0.7859629
NNAT	turquoise	0.50252673	3.13E-13	-0.689508	1.96E-27	-0.5148054
NMNAT2	turquoise	0.6574622	2.85E-24	-0.8093561	3.67E-44	-0.6350852
NME5	turquoise	0.64821832	1.98E-23	-0.6879549	2.85E-27	-0.4016266
NME4	turquoise	-0.6803467	1.73E-26	0.40881591	7.59E-09	0.46212547
NME3	turquoise	-0.6657482	4.72E-25	0.37943906	1.00E-07	0.79090539
NMD3	turquoise	0.45477869	7.87E-11	0.18233856	0.01298706	-0.4971453
NLK	turquoise	0.75278788	4.56E-35	-0.7422808	1.20E-33	-0.4008221
NLGN4X	turquoise	0.43946052	3.90E-10	-0.4524249	1.01E-10	-0.0314483
NLGN1	turquoise	0.55101572	4.41E-16	-0.3966855	2.27E-08	-0.2763799
NKX2-2	turquoise	-0.6122505	2.05E-20	0.64743719	2.32E-23	0.56443638
NKRF	turquoise	0.62010953	4.85E-21	-0.6204149	4.59E-21	-0.4986748
NKIRAS1	turquoise	0.723485	2.87E-31	-0.6675496	3.17E-25	-0.6109793
NKD2	turquoise	0.32757847	5.32E-06	-0.6210708	4.06E-21	0.0796143
NKAIN2	turquoise	0.55200201	3.81E-16	-0.5678637	3.48E-17	-0.2872324
NIPSNAP3B	turquoise	0.5996527	1.90E-19	-0.6830488	9.16E-27	-0.2532136
NIPSNAP1	turquoise	0.46733181	2.00E-11	-0.7504206	9.66E-35	-0.2850947
NIPBL	turquoise	-0.4084497	7.85E-09	0.64444585	4.28E-23	0.7990574
NINJ1	turquoise	-0.5742926	1.27E-17	0.70369465	5.71E-29	0.58905537
NIFK	turquoise	0.48587522	2.37E-12	-0.0633104	0.3919218	-0.5516229
NIF3L1	turquoise	0.68673279	3.82E-27	-0.5039857	2.61E-13	-0.7896713
NHLRC3	turquoise	0.5665849	4.24E-17	-0.226464	0.00193691	-0.6829056
NGDN	turquoise	0.42280222	2.03E-09	-0.1640843	0.02562751	-0.7327501
NGB	turquoise	-0.1903421	0.00945484	-0.0649342	0.37986706	0.49767582
NFYC-AS1	turquoise	-0.3360825	2.91E-06	0.36281335	3.87E-07	0.72945551
NFU1	turquoise	0.4613999	3.85E-11	-0.2368661	0.00116951	-0.8782871
NFS1	turquoise	0.75756207	9.76E-36	-0.5873735	1.52E-18	-0.4756441
NFKBIZ	turquoise	-0.3798484	9.68E-08	0.60226027	1.21E-19	0.50176764
NFKBIA	turquoise	-0.6869418	3.63E-27	0.6358986	2.37E-22	0.67383044
NFKB1	turquoise	-0.5337604	5.15E-15	0.71895978	1.00E-30	0.69073306
NFIC	turquoise	-0.6094899	3.37E-20	0.42632131	1.44E-09	0.82085287
NFIB	turquoise	-0.5714104	2.00E-17	0.70415122	5.08E-29	0.4686647
NFIA	turquoise	-0.5431121	1.38E-15	0.855604	3.18E-54	0.27346667
NFATC3	turquoise	-0.5446696	1.11E-15	0.33672037	2.78E-06	0.81832955
NEUROD6	turquoise	0.63381546	3.56E-22	-0.6733033	8.72E-26	-0.7045674
NEURL1	turquoise	-0.1868231	0.010887	-0.1175751	0.11096094	0.72871347
NEU4	turquoise	-0.3413802	1.98E-06	0.20480376	0.00516664	0.77849975
NETO2	turquoise	0.7386986	3.54E-33	-0.6207313	4.32E-21	-0.61824
NET1	turquoise	-0.2853783	8.24E-05	0.46387333	2.93E-11	0.49803865
NES	turquoise	-0.6621395	1.04E-24	0.50603444	2.02E-13	0.50791716
NELL2	turquoise	0.62923361	8.65E-22	-0.6021073	1.24E-19	-0.8066377
NELL1	turquoise	0.68023596	1.77E-26	-0.658806	2.13E-24	-0.8166428

NELFB	turquoise	-0.4181069	3.18E-09	-0.1187042	0.10755001	0.54761126
NEK7	turquoise	-0.4798846	4.79E-12	0.74032448	2.17E-33	0.50771636
NEK2	turquoise	0.78006083	4.09E-39	-0.6062582	5.99E-20	-0.3762225
NEGR1	turquoise	0.80108461	1.19E-42	-0.6329982	4.17E-22	-0.6539701
NEFM	turquoise	0.59582342	3.67E-19	-0.6143969	1.39E-20	-0.8234592
NEFH	turquoise	0.53802573	2.84E-15	-0.6336632	3.67E-22	-0.4409129
NEDD4L	turquoise	0.48990173	1.47E-12	-0.7172717	1.59E-30	-0.2329379
NECTIN3	turquoise	-0.1450631	0.04882322	-0.0721757	0.32890794	0.64597334
NECAP2	turquoise	-0.6735631	8.22E-26	0.77933938	5.33E-39	0.57812745
NECAP1	turquoise	0.68011898	1.82E-26	-0.6952403	4.81E-28	-0.7919178
NECAB1	turquoise	0.5910673	8.22E-19	-0.5943248	4.74E-19	-0.7937183
NDUFS7	turquoise	-0.61524	1.19E-20	0.14247906	0.05303062	0.68735365
NDUFS5	turquoise	0.54294221	1.42E-15	-0.3546299	7.32E-07	-0.6325851
NDUFS4	turquoise	0.44041496	3.53E-10	-0.0694389	0.34762631	-0.7614036
NDUFC2	turquoise	0.49748547	5.85E-13	-0.4786152	5.55E-12	0.11430322
NDUFC1	turquoise	0.37903472	1.04E-07	-0.1130283	0.12556084	-0.6548425
NDUFB6	turquoise	0.51085238	1.09E-13	-0.2762899	0.00014075	-0.7766346
NDUFB5	turquoise	0.53308191	5.65E-15	-0.10139	0.16967555	-0.7164284
NDUFB3	turquoise	0.29120359	5.78E-05	0.1027888	0.16383354	-0.7852588
NDUFAF5	turquoise	0.66803641	2.84E-25	-0.7163073	2.06E-30	-0.6050436
NDUFAF4	turquoise	0.71817284	1.24E-30	-0.246629	0.00071383	-0.4593651
NDUFAF1	turquoise	0.46654662	2.18E-11	-0.1694388	0.02112778	-0.6139877
NDUFAB1	turquoise	0.71296143	5.06E-30	-0.6217846	3.55E-21	-0.7769014
NDUFA4	turquoise	0.54597518	9.17E-16	-0.1986576	0.00671223	-0.8150486
NDUFA12	turquoise	0.33301333	3.63E-06	0.10755336	0.14506148	-0.6862345
NDUFA1	turquoise	0.33842574	2.46E-06	-0.042593	0.56484334	-0.6873442
NDRG4	turquoise	0.58920451	1.12E-18	-0.6864789	4.06E-27	-0.8025387
NDRG3	turquoise	0.55936267	1.28E-16	-0.7712101	9.69E-38	-0.6638274
NDRG1	turquoise	-0.6808452	1.54E-26	0.50761077	1.65E-13	0.53242213
NDN	turquoise	0.67008153	1.80E-25	-0.7282321	7.51E-32	-0.5464468
NDFIP2	turquoise	0.59797617	2.54E-19	-0.3560004	6.59E-07	-0.8624542
NDFIP1	turquoise	0.72680031	1.13E-31	-0.6886075	2.43E-27	-0.7454466
NDEL1	turquoise	0.62727827	1.26E-21	-0.6034528	9.82E-20	-0.5566143
NCSTN	turquoise	-0.5150457	6.35E-14	0.19766749	0.00699653	0.61310234
NCOA7	turquoise	0.60232539	1.20E-19	-0.5545282	2.63E-16	-0.8364237
NCKAP1L	turquoise	-0.4157983	3.96E-09	0.4600018	4.48E-11	0.55488713
NCKAP1	turquoise	0.477062	6.64E-12	-0.1330265	0.07105526	-0.8747061
NCEH1	turquoise	0.71642532	2.00E-30	-0.6616065	1.17E-24	-0.3328213
NCBP1	turquoise	-0.4686729	1.72E-11	0.66422921	6.59E-25	0.65567114
NCAPD2	turquoise	-0.383771	6.95E-08	0.27083678	0.00019244	0.54751178
NCAM2	turquoise	0.58947416	1.07E-18	-0.3374552	2.64E-06	-0.7543296
NCALD	turquoise	0.62098072	4.13E-21	-0.814978	3.13E-45	-0.5426099
NBPF22P	turquoise	-0.2027934	0.0056329	-0.0120015	0.87119714	0.755479
NBPF14	turquoise	-0.2135891	0.00350932	0.27080213	0.00019282	0.60559828
NBPF10	turquoise	-0.5934563	5.49E-19	0.38512544	6.20E-08	0.54825621
NBN	turquoise	0.43409384	6.70E-10	-0.0801186	0.27832554	-0.8600955

NBEAL2	turquoise	-0.4635682	3.03E-11	0.28864461	6.76E-05	0.86334135
NBEA	turquoise	0.68513694	5.58E-27	-0.5377011	2.97E-15	-0.8417422
NAV3	turquoise	0.64112967	8.36E-23	-0.375453	1.39E-07	-0.8579525
NAV2	turquoise	-0.3062205	2.24E-05	0.39816273	1.99E-08	0.60350731
NAT14	turquoise	-0.4275529	1.28E-09	-0.1757831	0.01669443	0.45592726
NASP	turquoise	-0.3182669	1.01E-05	0.32755994	5.33E-06	0.63671011
NAPRT	turquoise	-0.2428262	0.00086723	0.06520731	0.37786177	0.76591421
NAPEPLD	turquoise	0.68970003	1.87E-27	-0.3753923	1.40E-07	-0.58183
NAPB	turquoise	0.64239113	6.49E-23	-0.780445	3.56E-39	-0.5968614
NAP1L5	turquoise	0.5413194	1.78E-15	-0.6626089	9.38E-25	-0.8291992
NAP1L3	turquoise	0.6528915	7.48E-24	-0.6124771	1.97E-20	-0.8105717
NAP1L2	turquoise	0.68768308	3.04E-27	-0.5710277	2.12E-17	-0.8294087
NAGK	turquoise	-0.5075697	1.66E-13	0.15208762	0.03876882	0.64272363
NAE1	turquoise	0.40879357	7.61E-09	-0.1357433	0.06542674	-0.5977854
NADSYN1	turquoise	-0.4285908	1.16E-09	0.18292432	0.01269397	0.56164452
NACC2	turquoise	-0.6163582	9.70E-21	0.82779015	8.25E-48	0.56940324
NACAP1	turquoise	0.18482764	0.01178135	0.21916212	0.00272429	-0.3188331
NAA60	turquoise	-0.5013405	3.63E-13	0.11619356	0.11524925	0.46049021
NAA20	turquoise	0.66792127	2.92E-25	-0.4815129	3.96E-12	-0.522176
NAA10	turquoise	-0.2926411	5.29E-05	-0.2219172	0.00239828	0.67155158
N4BP2L1	turquoise	0.61030624	2.91E-20	-0.2166998	0.00304909	-0.7093282
N4BP1	turquoise	-0.2534513	0.00049962	-0.2698463	0.00020354	0.52867016
MZT1	turquoise	0.71462544	3.24E-30	-0.543047	1.40E-15	-0.7686565
MZF1	turquoise	-0.4492614	1.41E-10	0.26673073	0.0002425	0.72761925
MYT1L	turquoise	0.61972373	5.21E-21	-0.645445	3.49E-23	-0.816591
MYRIP	turquoise	0.35713893	6.03E-07	-0.2095014	0.00420919	-0.6007809
MYOT	turquoise	-0.5399592	2.16E-15	0.62603471	1.59E-21	0.56001659
MYOM1	turquoise	-0.551773	3.94E-16	0.51392216	7.34E-14	0.74668841
MYOF	turquoise	-0.3734941	1.64E-07	0.75080851	8.55E-35	0.14883866
MYO9B	turquoise	-0.6314375	5.65E-22	0.30137294	3.06E-05	0.69321284
MYO18A	turquoise	-0.2279369	0.00180578	-0.1559041	0.03407968	0.56157112
MYO10	turquoise	-0.4089509	7.50E-09	0.68933213	2.04E-27	0.68112338
MYL3	turquoise	-0.5133861	7.87E-14	0.32325507	7.19E-06	0.58444791
MYH9	turquoise	-0.4690865	1.64E-11	0.66225873	1.01E-24	-0.0585722
MYH11	turquoise	-0.2747813	0.00015357	0.53238243	6.23E-15	0.34698334
MYH10	turquoise	0.59578566	3.70E-19	-0.6177825	7.47E-21	-0.5377859
MYCBP2	turquoise	0.55952872	1.24E-16	-0.68881	2.32E-27	-0.5017456
MYBPH	turquoise	-0.2666893	0.00024306	0.31149487	1.59E-05	0.38205225
MYBPC1	turquoise	-0.4819808	3.75E-12	0.73696609	5.93E-33	0.69680609
MYADM	turquoise	0.52828053	1.09E-14	-0.597917	2.57E-19	-0.6387161
MVB12B	turquoise	-0.6261353	1.56E-21	0.32844745	5.01E-06	0.60161007
MVB12A	turquoise	-0.4240571	1.80E-09	0.31629229	1.15E-05	0.79719635
MUTYH	turquoise	-0.3812417	8.61E-08	0.21157841	0.00383926	0.71762652
MUM1L1	turquoise	0.65079339	1.16E-23	-0.5526251	3.48E-16	-0.7207339
MTX3	turquoise	0.60917682	3.56E-20	-0.2082661	0.00444411	-0.7336429
MTX2	turquoise	0.70057114	1.27E-28	-0.5938268	5.16E-19	-0.7921879

MTX1	turquoise	-0.3651858	3.20E-07	-0.0075559	0.918696	0.78882342
MTRF1L	turquoise	-0.4270873	1.34E-09	0.66345232	7.81E-25	0.56898368
MTPN	turquoise	0.65844393	2.31E-24	-0.5202441	3.21E-14	-0.5421657
MTMR9	turquoise	0.66245379	9.71E-25	-0.7206927	6.22E-31	-0.3595954
MTMR7	turquoise	0.70770324	2.03E-29	-0.7585912	6.96E-36	-0.5628357
MTMR6	turquoise	0.70561374	3.49E-29	-0.6437532	4.92E-23	-0.2998266
MTMR4	turquoise	0.64848933	1.87E-23	-0.5506263	4.67E-16	-0.5216102
MTIF3	turquoise	-0.3562489	6.46E-07	0.14094329	0.05567052	0.75371806
MTHFD2	turquoise	-0.2022863	0.00575632	0.34073767	2.08E-06	0.33739038
MTHFD1L	turquoise	0.55059097	4.69E-16	-0.4160716	3.86E-09	-0.6177386
MTERF3	turquoise	0.40679813	9.14E-09	-0.2734109	0.00016616	-0.7424604
MTCH1	turquoise	0.63720199	1.83E-22	-0.5303036	8.28E-15	-0.7737477
MTA3	turquoise	-0.4841385	2.91E-12	0.7175219	1.48E-30	0.54318547
MT3	turquoise	-0.6581436	2.46E-24	0.51418877	7.09E-14	0.72730688
MT2A	turquoise	-0.7198405	7.86E-31	0.71711427	1.66E-30	0.41983662
MT1X	turquoise	-0.5311887	7.34E-15	0.59212206	6.88E-19	0.3914337
MT1M	turquoise	-0.4523334	1.02E-10	0.72871409	6.54E-32	0.36978472
MT1H	turquoise	-0.4097223	6.98E-09	0.55349813	3.06E-16	0.51181521
MT1G	turquoise	-0.4130523	5.12E-09	0.53018025	8.42E-15	0.47989098
MT1F	turquoise	-0.4533016	9.22E-11	0.56017318	1.13E-16	0.53064849
MT1E	turquoise	-0.4952728	7.67E-13	0.66162166	1.16E-24	0.54995042
MT1A	turquoise	-0.5991769	2.07E-19	0.68880611	2.32E-27	0.47854433
MSRB1	turquoise	-0.5771084	8.10E-18	0.46877812	1.70E-11	0.71594023
MSRA	turquoise	0.39782338	2.05E-08	-0.4938108	9.17E-13	-0.634332
MSN	turquoise	-0.626197	1.55E-21	0.71984873	7.84E-31	0.27877298
MSMO1	turquoise	0.52543151	1.60E-14	-0.3742023	1.55E-07	-0.4218527
MSH2	turquoise	0.78256985	1.62E-39	-0.4419562	3.02E-10	-0.6089885
MS4A14	turquoise	-0.3485185	1.16E-06	0.66364289	7.49E-25	0.38916994
MRVI1	turquoise	-0.5129484	8.33E-14	0.50634253	1.94E-13	0.36945572
MRPS36	turquoise	0.62613454	1.56E-21	-0.4312024	8.93E-10	-0.29714
MRPS30	turquoise	0.60021175	1.73E-19	-0.4983946	5.23E-13	-0.8737767
MRPS28	turquoise	0.3676391	2.63E-07	0.10114701	0.17070581	-0.6036181
MRPS27	turquoise	0.5218326	2.60E-14	-0.3630401	3.80E-07	-0.8414455
MRPS23	turquoise	0.62143193	3.79E-21	-0.3861686	5.67E-08	-0.7008308
MRPS22	turquoise	0.42289125	2.01E-09	-0.0448892	0.54403213	-0.70868
MRPS21	turquoise	0.42083436	2.45E-09	-0.1763042	0.01636922	-0.6365615
MRPL52	turquoise	-0.2925874	5.31E-05	0.30395308	2.60E-05	0.72665887
MRPL50	turquoise	0.48837582	1.76E-12	-0.0443877	0.54854527	-0.6394512
MRPL47	turquoise	0.59663659	3.20E-19	-0.2071677	0.00466283	-0.1948926
MRPL45	turquoise	0.69566028	4.34E-28	-0.3476183	1.25E-06	-0.6481259
MRPL40	turquoise	0.29772605	3.86E-05	-0.1087716	0.14053468	-0.5248745
MRPL39	turquoise	0.38513836	6.19E-08	0.05010868	0.4981789	-0.769389
MRPL32	turquoise	0.63717377	1.84E-22	-0.4138118	4.77E-09	-0.708111
MRPL3	turquoise	0.51693247	4.96E-14	-0.2063087	0.00484056	-0.8995613
MRPL21	turquoise	0.32021244	8.85E-06	-0.5566333	1.92E-16	-0.5980396
MRPL15	turquoise	0.65010233	1.34E-23	-0.43309	7.40E-10	-0.444976

MRPL1	turquoise	0.79497105	1.40E-41	-0.5920349	6.99E-19	-0.4945133
MRAP2	turquoise	0.60411228	8.75E-20	-0.5899849	9.85E-19	-0.7600689
MPZL2	turquoise	-0.3412655	2.00E-06	0.4957565	7.23E-13	0.28704882
MPST	turquoise	-0.5538614	2.90E-16	0.34113995	2.02E-06	0.8568902
MPRIP	turquoise	-0.4578275	5.67E-11	0.01677402	0.82071849	0.55894533
MPPED2	turquoise	0.75040495	9.71E-35	-0.36002	4.82E-07	-0.4357224
MPPED1	turquoise	0.56750414	3.68E-17	-0.6804262	1.69E-26	-0.5835705
MPP1	turquoise	0.43371405	6.96E-10	-0.7205522	6.46E-31	-0.7090728
MPHOSPH1(	turquoise	-0.3612493	4.38E-07	0.10657943	0.14875937	0.56197224
MPC2	turquoise	0.38426767	6.67E-08	-0.170575	0.02026609	-0.815213
MPC1	turquoise	0.31789725	1.04E-05	0.19450436	0.00797798	-0.5609906
MOV10	turquoise	-0.6720412	1.16E-25	0.45922364	4.88E-11	0.65645759
MOSPD2	turquoise	-0.4692062	1.62E-11	0.13239436	0.07241994	0.54437312
MORN4	turquoise	0.42760382	1.27E-09	-0.7522791	5.36E-35	-0.4209391
MORF4L2	turquoise	0.5435913	1.29E-15	-0.2926612	5.29E-05	-0.9218043
MOBP	turquoise	-0.463419	3.08E-11	0.31636757	1.15E-05	0.56902241
MOB4	turquoise	0.69461852	5.61E-28	-0.5082925	1.51E-13	-0.8416491
MOB3A	turquoise	-0.550906	4.48E-16	0.38226109	7.90E-08	0.78052315
MOB2	turquoise	-0.6738715	7.66E-26	0.16259834	0.02701343	0.58739425
MOAP1	turquoise	0.65560678	4.22E-24	-0.5872698	1.55E-18	-0.8764391
MNS1	turquoise	0.38988571	4.12E-08	-0.2123965	0.00370179	-0.3347237
MNAT1	turquoise	0.64453221	4.20E-23	-0.1422952	0.05334109	-0.4383666
MMD	turquoise	0.5728717	1.59E-17	-0.2774634	0.00013147	-0.7687002
MMADHC	turquoise	0.46078553	4.11E-11	0.03492267	0.63698092	-0.8033851
MMAB	turquoise	-0.4634351	3.08E-11	-0.0893961	0.22623988	0.58550977
MLST8	turquoise	-0.4904477	1.38E-12	0.09414548	0.20242576	0.4052033
MLKL	turquoise	-0.5080055	1.57E-13	0.58375848	2.76E-18	0.64648875
MLIP	turquoise	0.65137549	1.03E-23	-0.6910294	1.35E-27	-0.7850424
MLEC	turquoise	-0.5432128	1.36E-15	0.32805519	5.15E-06	0.06234438
MKRN2	turquoise	0.2878465	7.10E-05	0.01069073	0.8851624	-0.6571824
MKL2	turquoise	0.71668494	1.86E-30	-0.674547	6.57E-26	-0.6907882
MKKS	turquoise	0.6452285	3.65E-23	-0.4883923	1.76E-12	-0.3549445
MIR503HG	turquoise	-0.2884927	6.82E-05	0.34074942	2.07E-06	0.69090044
MIR30C2	turquoise	-0.4258027	1.52E-09	0.53532539	4.14E-15	0.74682657
MIR300	turquoise	-0.3895012	4.26E-08	0.19792805	0.0069207	0.77431528
MIR124-2H(	turquoise	0.52395902	1.96E-14	-0.5212173	2.82E-14	-0.4285427
MIR1185-1	turquoise	-0.5599948	1.16E-16	0.36447546	3.39E-07	0.78387838
MIR100HG	turquoise	-0.4636152	3.02E-11	0.33893839	2.37E-06	0.52187329
MIER2	turquoise	-0.5102523	1.18E-13	-0.0311421	0.67389467	0.50579717
MIDN	turquoise	-0.4023301	1.37E-08	0.25293586	0.00051345	0.82479028
MID1IP1	turquoise	-0.530118	8.50E-15	0.54578774	9.42E-16	0.60468839
MICU3	turquoise	0.7676192	3.36E-37	-0.4996115	4.50E-13	-0.4190983
MICALL2	turquoise	-0.6333148	3.92E-22	0.35498805	7.12E-07	0.4389867
MICAL1	turquoise	-0.5221593	2.49E-14	-0.0072438	0.92204351	0.55872086
MGST3	turquoise	0.49482307	8.11E-13	-0.0806872	0.27491786	-0.7659039
MGLL	turquoise	-0.6458095	3.24E-23	0.49683834	6.34E-13	0.37022239

MGEA5	turquoise	0.45392959	8.62E-11	-0.2136624	0.00349781	-0.1963911
MGAT2	turquoise	0.45603484	6.88E-11	0.0198778	0.78826723	-0.6741474
MGAT1	turquoise	-0.7390209	3.21E-33	0.55887672	1.37E-16	0.65279808
MFSD6	turquoise	0.63360736	3.71E-22	-0.752426	5.12E-35	-0.5220253
MFSD4A	turquoise	0.71933773	9.02E-31	-0.5556484	2.22E-16	-0.6504427
MFNG	turquoise	-0.4902619	1.41E-12	0.71849157	1.14E-30	0.58680857
MFN2	turquoise	-0.2692117	0.00021097	-0.1333477	0.07037007	0.49077672
MFGE8	turquoise	-0.5719788	1.83E-17	0.58609382	1.88E-18	0.77524675
MFAP3L	turquoise	-0.5589816	1.35E-16	0.50196791	3.36E-13	0.81655577
MEX3A	turquoise	-0.4840777	2.93E-12	0.25471806	0.00046709	0.76459455
METTL9	turquoise	0.55119521	4.29E-16	-0.2975945	3.89E-05	-0.7743165
METTL7B	turquoise	-0.5610945	9.81E-17	0.59763657	2.69E-19	0.5019959
METTL16	turquoise	-0.3824087	7.80E-08	0.09996811	0.17577102	0.78306462
METAP1	turquoise	0.50015986	4.21E-13	-0.3240942	6.78E-06	-0.8953381
MET	turquoise	0.62397997	2.35E-21	-0.6907397	1.45E-27	-0.3654353
MEPE	turquoise	0.60464541	7.96E-20	-0.6920695	1.05E-27	-0.4084248
MEPCE	turquoise	-0.3923441	3.33E-08	0.09729673	0.18765863	0.77117041
MEGF10	turquoise	-0.3811566	8.67E-08	0.70618542	3.01E-29	0.39323445
MEF2C	turquoise	0.55437207	2.69E-16	-0.6103335	2.90E-20	-0.4933972
MED7	turquoise	0.65048155	1.24E-23	-0.274236	0.00015847	-0.6382132
MED6	turquoise	0.54462779	1.11E-15	-0.719887	7.76E-31	-0.2772942
MED31	turquoise	0.48850812	1.74E-12	-0.3700351	2.17E-07	-0.672854
MED27	turquoise	0.5363149	3.61E-15	-0.3660573	2.99E-07	-0.781379
MED22	turquoise	-0.3161384	1.17E-05	0.18064256	0.01386939	0.84018019
MED16	turquoise	-0.5372817	3.15E-15	0.1810947	0.01362918	0.7447348
MED10	turquoise	0.57236688	1.72E-17	-0.6152922	1.18E-20	-0.5822403
MECOM	turquoise	-0.5753124	1.08E-17	0.72003222	7.46E-31	0.64716809
MEAF6	turquoise	0.68383196	7.61E-27	-0.5739972	1.33E-17	-0.7531992
ME3	turquoise	0.61951174	5.42E-21	-0.7376818	4.79E-33	-0.5968408
ME2	turquoise	0.26680731	0.00024146	0.31601769	1.18E-05	-0.5014764
ME1	turquoise	0.441242	3.25E-10	-0.0214889	0.77156102	-0.7412315
MDP1	turquoise	0.527825	1.16E-14	-0.6355326	2.54E-22	-0.3854037
MDH2	turquoise	0.58935118	1.10E-18	-0.4607979	4.11E-11	-0.7883106
MDGA2	turquoise	0.56680283	4.10E-17	-0.4189738	2.93E-09	-0.3816321
MDGA1	turquoise	-0.4980935	5.43E-13	0.3577325	5.76E-07	0.57131967
MCTP1	turquoise	0.45084227	1.20E-10	-0.5359242	3.81E-15	-0.4251656
MCRS1	turquoise	-0.3912562	3.66E-08	0.00796266	0.91433562	0.78066021
MCRIP1	turquoise	-0.5762879	9.24E-18	0.07427151	0.3150205	0.29564006
MCM7	turquoise	-0.6338806	3.51E-22	0.54355214	1.30E-15	0.70406272
MCM6	turquoise	0.4744096	9.00E-12	-0.3156752	1.20E-05	-0.2443746
MCM4	turquoise	0.58710921	1.59E-18	-0.7011176	1.10E-28	-0.3465897
MCL1	turquoise	-0.5333957	5.41E-15	0.67366824	8.03E-26	0.76835496
MCFD2	turquoise	0.42734965	1.31E-09	0.01494856	0.83995139	-0.5982791
MCF2L	turquoise	-0.5929849	5.95E-19	0.21570474	0.00319	0.40877111
MCF2	turquoise	0.69698138	3.12E-28	-0.7128336	5.24E-30	-0.4382173
MCEE	turquoise	0.49625622	6.80E-13	0.00361621	0.96103678	-0.5838163

MBTPS2	turquoise	0.68682696	3.73E-27	-0.4498475	1.33E-10	-0.8126466
MBP	turquoise	0.63299271	4.18E-22	-0.6403125	9.85E-23	-0.6030478
MBNL2	turquoise	0.5874271	1.51E-18	-0.3448178	1.54E-06	-0.6052941
MBNL1	turquoise	0.48921782	1.59E-12	-0.0270906	0.71433413	-0.4409171
MBLAC2	turquoise	0.74859801	1.71E-34	-0.5643777	5.95E-17	-0.7478556
MBIP	turquoise	0.56384457	6.46E-17	-0.0243515	0.7421403	-0.6899915
MB21D2	turquoise	0.7314402	2.99E-32	-0.6616523	1.16E-24	-0.4782179
MAVS	turquoise	-0.4219022	2.21E-09	0.54724248	7.64E-16	0.3832502
MATR3	turquoise	0.65155157	9.90E-24	-0.6054269	6.94E-20	-0.6977941
MAT2B	turquoise	0.68122055	1.41E-26	-0.3846416	6.46E-08	-0.7439111
MAT2A	turquoise	-0.5157465	5.79E-14	0.34965545	1.07E-06	0.69573215
MARS2	turquoise	0.41447794	4.48E-09	-0.1455618	0.0480441	-0.797178
8-Mar	turquoise	-0.2234078	0.00223707	0.23599735	0.00122087	0.62853314
7-Mar	turquoise	0.27696992	0.0001353	0.21299133	0.00360464	-0.5616467
5-Mar	turquoise	0.51630572	5.39E-14	-0.1272328	0.08437529	-0.5837954
4-Mar	turquoise	0.27667311	0.00013765	-0.6879479	2.85E-27	0.02597696
11-Mar	turquoise	0.66367328	7.44E-25	-0.7459674	3.88E-34	-0.5753175
2-Mar	turquoise	0.37301418	1.70E-07	-0.0164088	0.82455861	-0.6099681
MAPT	turquoise	0.32335614	7.14E-06	-0.6782606	2.80E-26	-0.5970398
MAPKAPK2	turquoise	-0.6831035	9.04E-27	0.54001339	2.15E-15	0.72465912
MAPK9	turquoise	0.60769879	4.64E-20	-0.4885748	1.72E-12	-0.888394
MAPK8	turquoise	0.62608793	1.58E-21	-0.7251195	1.81E-31	-0.4451992
MAPK6	turquoise	0.67625563	4.45E-26	-0.5102641	1.18E-13	-0.8034083
MAPK3	turquoise	-0.3211987	8.28E-06	0.12816661	0.08210224	0.71339141
MAPK14	turquoise	-0.360136	4.77E-07	0.20303442	0.00557509	0.79909416
MAPK13	turquoise	0.51406477	7.21E-14	-0.6763892	4.31E-26	-0.467279
MAPK10	turquoise	0.47898709	5.32E-12	-0.4227109	2.05E-09	-0.8657116
MAPK1	turquoise	0.63833546	1.46E-22	-0.7854833	5.47E-40	-0.3495138
MAP4K5	turquoise	-0.3362115	2.88E-06	0.32270496	7.47E-06	0.60499051
MAP4K4	turquoise	-0.632062	5.01E-22	0.64943994	1.54E-23	0.42201081
MAP4K3	turquoise	0.53439113	4.72E-15	-0.1315192	0.07434409	-0.8075192
MAP4K2	turquoise	-0.4504116	1.25E-10	0.27697758	0.00013524	0.80936555
MAP4	turquoise	0.70433449	4.85E-29	-0.7687464	2.28E-37	-0.6623473
MAP3K9	turquoise	0.5395775	2.28E-15	-0.779436	5.14E-39	-0.5628927
MAP3K5	turquoise	-0.4564002	6.61E-11	0.62945238	8.29E-22	0.5153803
MAP3K11	turquoise	-0.6562879	3.65E-24	0.30198106	2.95E-05	0.61673977
MAP3K10	turquoise	-0.498506	5.16E-13	0.23038906	0.0016053	0.58109466
MAP2K4	turquoise	0.63138501	5.71E-22	-0.6143196	1.41E-20	-0.8251893
MAP2K1	turquoise	0.63077886	6.42E-22	-0.55497	2.46E-16	-0.8777754
MAP2	turquoise	0.41702402	3.53E-09	-0.4076194	8.48E-09	-0.3663893
MAP1LC3B	turquoise	0.27290187	0.00017107	-0.0456645	0.53709189	-0.7924361
MAP10	turquoise	0.71823718	1.22E-30	-0.5555551	2.25E-16	-0.6846714
MANF	turquoise	-0.1560637	0.03389451	0.20599317	0.00490735	0.4933734
MAN1C1	turquoise	0.40253829	1.35E-08	-0.3185495	9.91E-06	-0.7630988
MAN1A1	turquoise	0.70931804	1.33E-29	-0.2742751	0.00015811	-0.6254191
MALSU1	turquoise	0.4060908	9.75E-09	-0.1605393	0.0290395	-0.9043334

MAL2	turquoise	0.74375237	7.67E-34	-0.6503904	1.26E-23	-0.7731813
MAK16	turquoise	0.7561187	1.56E-35	-0.4665018	2.19E-11	-0.421917
MAGI2	turquoise	0.56658023	4.24E-17	-0.1025139	0.16496967	-0.530798
MAGEH1	turquoise	0.70746264	2.16E-29	-0.4583091	5.38E-11	-0.776507
MAGEE1	turquoise	0.64556864	3.40E-23	-0.7488041	1.61E-34	-0.6332772
MAFG-AS1	turquoise	-0.3520334	8.93E-07	0.1000165	0.17556097	0.72881009
MAEL	turquoise	0.73525744	9.81E-33	-0.5504428	4.79E-16	-0.5983309
MAD2L2	turquoise	-0.6560977	3.80E-24	0.30851202	1.93E-05	0.32270841
MAD2L1BP	turquoise	0.66510416	5.44E-25	-0.4464372	1.90E-10	-0.6505203
MAD2L1	turquoise	0.734035	1.40E-32	-0.4832341	3.24E-12	-0.7366148
MACF1	turquoise	-0.4613566	3.86E-11	0.26339089	0.00029189	0.35310585
LZTS2	turquoise	-0.6550179	4.78E-24	0.16819632	0.02210608	0.54624097
LZTR1	turquoise	-0.7025285	7.70E-29	0.38471385	6.42E-08	0.59282585
LZTFL1	turquoise	0.70439759	4.77E-29	-0.5853303	2.13E-18	-0.7091374
LYRM7	turquoise	0.44081569	3.39E-10	-0.4222118	2.15E-09	-0.5880172
LYPLAL1	turquoise	0.31107088	1.63E-05	0.1390698	0.05903701	-0.467699
LYPD1	turquoise	-0.2949159	4.60E-05	0.42640329	1.43E-09	0.72180249
LYAR	turquoise	-0.3813734	8.51E-08	0.71100768	8.50E-30	0.41902569
LY6G5C	turquoise	-0.5001065	4.23E-13	0.49236203	1.09E-12	0.55082651
LTN1	turquoise	0.30876857	1.90E-05	-0.1748622	0.01728287	-0.8096711
LTBR	turquoise	-0.6056728	6.64E-20	0.66539866	5.10E-25	0.36852716
LSS	turquoise	-0.6669244	3.64E-25	0.40365784	1.22E-08	0.2919119
LSM3	turquoise	0.29528983	4.49E-05	0.02388608	0.74689986	-0.7859037
LRRTM3	turquoise	0.72905069	5.94E-32	-0.592386	6.58E-19	-0.77628
LRRTM2	turquoise	0.65171158	9.58E-24	-0.3906986	3.84E-08	-0.4131518
LRRTM1	turquoise	0.65479437	5.01E-24	-0.6533062	6.86E-24	-0.7167773
LRRN3	turquoise	0.59276556	6.18E-19	-0.4625777	3.38E-11	-0.5625231
LRRN2	turquoise	-0.2356816	0.00124004	0.00620599	0.93318427	0.75912968
LRRCC1	turquoise	-0.4880518	1.83E-12	0.57371735	1.39E-17	0.55243924
LRRC8B	turquoise	0.71176472	6.96E-30	-0.7849202	6.76E-40	-0.6511127
LRRC8A	turquoise	-0.5485462	6.32E-16	0.74678703	3.01E-34	0.51353981
LRRC73	turquoise	0.4815095	3.97E-12	-0.7562064	1.52E-35	-0.4485998
LRRC7	turquoise	0.57003109	2.48E-17	-0.5707878	2.20E-17	-0.3324862
LRRC4C	turquoise	0.60400577	8.91E-20	-0.5151663	6.25E-14	-0.6608135
LRRC49	turquoise	0.6631652	8.31E-25	-0.6830075	9.25E-27	-0.615644
LRRC40	turquoise	0.64652593	2.80E-23	-0.3276468	5.30E-06	-0.763653
LRRC32	turquoise	-0.5398719	2.19E-15	0.56029304	1.11E-16	0.74318409
LRRC25	turquoise	-0.3555944	6.79E-07	0.33045231	4.35E-06	0.51818788
LRRC2	turquoise	0.62321748	2.71E-21	-0.6470809	2.50E-23	-0.4365552
LRRC1	turquoise	-0.4721883	1.16E-11	0.67128868	1.37E-25	0.45280406
LRPPRC	turquoise	0.59921972	2.05E-19	-0.7497447	1.20E-34	-0.5580257
LRPAP1	turquoise	0.28677127	7.57E-05	-0.2649266	0.00026812	-0.7696852
LRP11	turquoise	0.63247167	4.63E-22	-0.401013	1.54E-08	-0.8957948
LRP10	turquoise	-0.7237528	2.66E-31	0.71881701	1.04E-30	0.47371197
LRMP	turquoise	0.59864601	2.26E-19	-0.3692493	2.31E-07	-0.640238
LRFN5	turquoise	0.69071319	1.46E-27	-0.7239368	2.53E-31	-0.6682894

LRFN4	turquoise	-0.5398724	2.19E-15	-0.0209922	0.77670059	0.46543917
LRCH1	turquoise	0.75406924	3.02E-35	-0.6361468	2.25E-22	-0.6049515
LPP	turquoise	-0.5791601	5.83E-18	0.84929086	1.18E-52	0.65619701
LPAR6	turquoise	-0.3076356	2.05E-05	0.73753115	5.01E-33	0.28507728
LPAR5	turquoise	-0.2557578	0.00044187	0.65447089	5.37E-24	0.22008452
LPAR4	turquoise	-0.252739	0.00051882	0.2570143	0.00041307	0.69795118
LONRF2	turquoise	0.55525508	2.36E-16	-0.4812273	4.10E-12	-0.6088888
LONRF1	turquoise	0.2997099	3.40E-05	0.07237279	0.32758549	-0.6327483
LONP2	turquoise	0.47852972	5.61E-12	-0.4952504	7.69E-13	-0.7197915
LOC729970	turquoise	-0.4107769	6.33E-09	0.48008101	4.68E-12	0.55099746
LOC729860	turquoise	0.64462462	4.13E-23	-0.5850268	2.24E-18	-0.5887789
LOC728554	turquoise	0.46336968	3.10E-11	-0.6557305	4.11E-24	-0.2941137
LOC440040	turquoise	0.57814175	6.86E-18	-0.6490838	1.65E-23	-0.2985602
LOC285147	turquoise	0.62558581	1.74E-21	-0.5722674	1.75E-17	-0.7938374
LOC283713	turquoise	0.54306607	1.39E-15	-0.6015244	1.38E-19	-0.5951378
LOC146880	turquoise	0.48492541	2.66E-12	-0.1947938	0.00788333	-0.834326
LOC105372[illegible]	turquoise	-0.3982506	1.98E-08	0.22959029	0.00166825	0.75114459
LOC102467[illegible]	turquoise	-0.381509	8.42E-08	0.20421971	0.0052984	0.80986671
LOC100129[illegible]	turquoise	-0.5625568	7.86E-17	0.66944058	2.08E-25	0.49494524
LOC100128[illegible]	turquoise	0.56946773	2.71E-17	-0.5949285	4.28E-19	-0.5333544
LNX1	turquoise	0.71062042	9.42E-30	-0.6678691	2.95E-25	-0.6207273
LMTK2	turquoise	0.67591833	4.80E-26	-0.6357748	2.42E-22	-0.4689698
LMO4	turquoise	0.47140503	1.27E-11	-0.6166543	9.19E-21	-0.2695101
LMO3	turquoise	0.57220156	1.77E-17	-0.5341063	4.91E-15	-0.5837785
LMBRD2	turquoise	0.66143577	1.21E-24	-0.6298979	7.61E-22	-0.7544917
LMBRD1	turquoise	0.31354783	1.39E-05	0.18547393	0.01148486	-0.7675483
LMBR1	turquoise	0.64413422	4.56E-23	-0.3526065	8.55E-07	-0.8382638
LLGL1	turquoise	-0.4703888	1.42E-11	0.36041349	4.67E-07	0.79726104
LINC00930	turquoise	-0.3112258	1.62E-05	0.25478459	0.00046544	0.55097722
LINC00672	turquoise	0.73559392	8.89E-33	-0.6344934	3.12E-22	-0.73293
LINC00623	turquoise	-0.4253346	1.59E-09	0.3732645	1.67E-07	0.46033385
LINC00493	turquoise	0.44531092	2.14E-10	0.05717156	0.43953467	-0.7015044
LINC00461	turquoise	0.30212779	2.92E-05	0.1006505	0.17282572	-0.3695764
LINC00403	turquoise	0.45073965	1.21E-10	-0.0936228	0.20495413	-0.5227489
LINC00320	turquoise	-0.368124	2.53E-07	0.37822922	1.11E-07	0.65288143
LINC00173	turquoise	-0.4230902	1.98E-09	0.24705096	0.00069845	0.7894105
LINC00092	turquoise	-0.2787838	0.00012172	0.14960379	0.04210382	0.69152905
LIME1	turquoise	-0.5353561	4.13E-15	0.29174917	5.59E-05	0.86947806
LILRA2	turquoise	-0.2805451	0.00010976	0.68281566	9.67E-27	0.35799714
LHX6	turquoise	0.52524738	1.65E-14	-0.7572063	1.10E-35	-0.2508991
LHPP	turquoise	-0.500189	4.19E-13	0.3973806	2.14E-08	0.77696967
LGMN	turquoise	0.55971195	1.21E-16	-0.5191362	3.71E-14	-0.802516
LGI4	turquoise	-0.6267495	1.39E-21	0.33966289	2.25E-06	0.35016756
LGALSL	turquoise	0.72920634	5.68E-32	-0.4522236	1.03E-10	-0.6834235
LGALS8	turquoise	0.64149322	7.77E-23	-0.7178971	1.34E-30	-0.3847242
LFNG	turquoise	-0.4643879	2.77E-11	0.29244058	5.36E-05	0.80387409

LDOC1L	turquoise	0.26729357	0.00023498	-0.0514962	0.48634178	-0.8356401
LDLRAP1	turquoise	-0.5179206	4.36E-14	0.36355095	3.65E-07	0.45311753
LDHB	turquoise	0.33827692	2.48E-06	0.1935367	0.00830172	-0.6224046
LDHA	turquoise	0.42808349	1.22E-09	-0.2832841	9.33E-05	-0.8699483
LDB2	turquoise	0.63542498	2.60E-22	-0.7693417	1.86E-37	-0.6637413
LCP1	turquoise	-0.3821706	7.96E-08	0.68459539	6.35E-27	0.20953657
LCNL1	turquoise	-0.481177	4.12E-12	0.06831565	0.35549914	0.19388759
LCMT2	turquoise	0.40795663	8.22E-09	-0.0422173	0.56828489	-0.7571115
LCMT1	turquoise	0.36501226	3.25E-07	-0.8503415	6.53E-53	-0.07028
LCLAT1	turquoise	0.81159093	1.39E-44	-0.4395305	3.87E-10	-0.3895139
LBHD1	turquoise	-0.539711	2.24E-15	0.28417601	8.85E-05	0.7178682
LARGE1	turquoise	0.59298973	5.95E-19	-0.7292521	5.61E-32	-0.4780869
LAPTM5	turquoise	-0.3954398	2.53E-08	0.54493514	1.07E-15	0.42964187
LAPTM4B	turquoise	0.34724347	1.28E-06	-0.0735202	0.31995406	-0.7862153
LANCL2	turquoise	0.65295344	7.39E-24	-0.5697962	2.57E-17	-0.6392758
LANCL1	turquoise	0.6500144	1.36E-23	-0.5418735	1.65E-15	-0.696942
LAMTOR4	turquoise	-0.6070225	5.23E-20	0.18382721	0.01225358	0.52870186
LAMTOR3	turquoise	0.42725365	1.32E-09	0.02487475	0.73680145	-0.7454558
LAMP5	turquoise	0.59890328	2.17E-19	-0.7314061	3.02E-32	-0.4122368
LAMP1	turquoise	-0.4186748	3.02E-09	0.33664527	2.80E-06	0.49705739
LAMA5	turquoise	-0.6561055	3.80E-24	0.6232718	2.69E-21	0.74356262
LACTB2	turquoise	0.49740744	5.91E-13	-0.2519946	0.00053962	-0.5451907
LACC1	turquoise	-0.3263013	5.82E-06	0.07690858	0.29809761	0.65028018
KYAT3	turquoise	0.54298419	1.41E-15	-0.308494	1.94E-05	-0.5062946
KTN1	turquoise	-0.2413352	0.00093523	0.69145185	1.22E-27	0.17846702
KTI12	turquoise	0.52604452	1.48E-14	-0.5899276	9.95E-19	-0.6671842
KSR2	turquoise	-0.3172732	1.08E-05	0.03575623	0.62895689	0.80096002
KRT222	turquoise	0.66256131	9.48E-25	-0.785913	4.65E-40	-0.6601951
KRCC1	turquoise	-0.6001254	1.75E-19	0.4850924	2.60E-12	0.79310609
KRAS	turquoise	0.39388886	2.91E-08	-0.4691907	1.62E-11	-0.6910176
KPNB1	turquoise	0.33797221	2.54E-06	0.04105533	0.57898899	-0.7165236
KPNA3	turquoise	0.58134749	4.09E-18	-0.124861	0.09037633	-0.7773506
KPNA1	turquoise	0.52937427	9.40E-15	-0.3762028	1.31E-07	-0.8408052
KMT5C	turquoise	-0.3843896	6.60E-08	0.13173312	0.07387004	0.69660282
KMT2E	turquoise	-0.5130237	8.25E-14	0.22455447	0.00211982	0.74162523
KLKB1	turquoise	-0.3976079	2.09E-08	0.27361283	0.00016424	0.83554507
KLK7	turquoise	0.57333749	1.48E-17	-0.5979022	2.57E-19	-0.3083427
KLHL9	turquoise	0.57607426	9.56E-18	-0.2048197	0.00516308	-0.6633802
KLHL7	turquoise	0.51022291	1.18E-13	-0.0765438	0.300402	-0.8255194
KLHL5	turquoise	-0.5574156	1.71E-16	0.76142085	2.73E-36	0.26902814
KLHL42	turquoise	0.64149163	7.77E-23	-0.4758174	7.66E-12	-0.7554175
KLHL4	turquoise	0.51470766	6.63E-14	-0.6365413	2.08E-22	-0.6642257
KLHL29	turquoise	0.46023785	4.37E-11	-0.4583932	5.33E-11	-0.2172647
KLHL28	turquoise	0.25416734	0.00048099	-0.2246179	0.0021135	-0.4602668
KLHL12	turquoise	0.6484438	1.89E-23	-0.3629118	3.84E-07	-0.7373236
KLHDC9	turquoise	0.50018082	4.20E-13	-0.516862	5.01E-14	-0.8050017

KLHDC8B	turquoise	-0.4372366	4.88E-10	0.37725846	1.20E-07	0.6861071
KLHDC2	turquoise	0.54405007	1.21E-15	-0.1638288	0.02586136	-0.6084538
KLF9	turquoise	0.34562224	1.45E-06	0.02395433	0.74620132	-0.5544936
KLF6	turquoise	-0.5170927	4.86E-14	0.55126584	4.25E-16	0.58482952
KLF2	turquoise	-0.4668921	2.10E-11	0.63275575	4.38E-22	0.6508126
KLF15	turquoise	-0.7083197	1.72E-29	0.73839405	3.87E-33	0.62855035
KLC1	turquoise	0.58345703	2.90E-18	-0.8398586	1.93E-50	-0.4855111
KITLG	turquoise	0.77908255	5.85E-39	-0.4240512	1.80E-09	-0.6534953
KIT	turquoise	0.76134703	2.80E-36	-0.6638794	7.11E-25	-0.4050098
KIFAP3	turquoise	0.54920568	5.74E-16	-0.6975687	2.70E-28	-0.6939535
KIF5B	turquoise	-0.503624	2.73E-13	0.71429829	3.54E-30	0.20190239
KIF5A	turquoise	-0.5550794	2.42E-16	0.45564249	7.18E-11	0.48647867
KIF3C	turquoise	0.39766596	2.08E-08	-0.393357	3.04E-08	-0.7222334
KIF3A	turquoise	0.65194616	9.12E-24	-0.4896564	1.51E-12	-0.5869322
KIF22	turquoise	-0.2861121	7.88E-05	-0.1426609	0.05272509	0.61488395
KIF21A	turquoise	0.68364001	7.96E-27	-0.5195015	3.54E-14	-0.6258397
KIF1C	turquoise	-0.6642616	6.54E-25	0.54378605	1.26E-15	0.67946552
KIF1BP	turquoise	0.49665435	6.48E-13	-0.2897469	6.32E-05	-0.9606569
KIF19	turquoise	-0.3899605	4.09E-08	0.52790647	1.15E-14	0.16971633
KIF13B	turquoise	-0.6252249	1.86E-21	0.48254918	3.51E-12	0.35581958
KIDINS220	turquoise	0.39829763	1.97E-08	-0.0601858	0.41575629	-0.7665478
KIAA2022	turquoise	0.81672643	1.43E-45	-0.552796	3.39E-16	-0.5380927
KIAA1958	turquoise	-0.6201138	4.85E-21	0.52841389	1.07E-14	0.74814661
KIAA1755	turquoise	-0.5237279	2.02E-14	0.41205333	5.63E-09	0.64585649
KIAA1683	turquoise	-0.2542853	0.00047798	0.08224059	0.26575241	0.78806495
KIAA1549L	turquoise	0.57563348	1.03E-17	-0.7134044	4.50E-30	-0.3488289
KIAA1468	turquoise	0.63843898	1.43E-22	-0.4523908	1.02E-10	-0.7728835
KIAA1217	turquoise	0.68957556	1.93E-27	-0.6087626	3.84E-20	-0.354426
KIAA1211L	turquoise	0.57553305	1.04E-17	-0.6861653	4.37E-27	-0.7033468
KIAA0556	turquoise	-0.4290598	1.10E-09	0.65363178	6.41E-24	0.48640215
KIAA0513	turquoise	0.53433122	4.76E-15	-0.7156405	2.47E-30	-0.7022961
KIAA0430	turquoise	0.5722308	1.76E-17	-0.3062267	2.24E-05	-0.5401138
KIAA0368	turquoise	0.62125786	3.92E-21	-0.4810986	4.16E-12	-0.7314096
KIAA0355	turquoise	-0.4801612	4.64E-12	0.55827163	1.50E-16	0.77695325
KIAA0319	turquoise	0.58347485	2.89E-18	-0.5559512	2.13E-16	-0.2205983
KIAA0232	turquoise	0.73025122	4.21E-32	-0.6038345	9.18E-20	-0.4685125
KHSRP	turquoise	-0.6055775	6.76E-20	0.30991918	1.76E-05	0.84051864
KHNYN	turquoise	-0.7283856	7.19E-32	0.69028093	1.62E-27	0.56026672
KHDRBS3	turquoise	0.2206853	0.00253941	-0.4900222	1.45E-12	-0.4257354
KHDRBS1	turquoise	0.46742629	1.98E-11	-0.1933887	0.00835226	-0.8824599
KDSR	turquoise	-0.4105707	6.46E-09	0.58906463	1.15E-18	0.55931585
KDM2A	turquoise	-0.3756987	1.37E-07	0.24520724	0.00076799	0.76828418
KCTD8	turquoise	0.68373443	7.79E-27	-0.6733328	8.66E-26	-0.6239894
KCTD16	turquoise	0.69154936	1.19E-27	-0.6123733	2.01E-20	-0.7433031
KCTD15	turquoise	-0.4293929	1.07E-09	0.52519927	1.66E-14	0.66737491
KCNV1	turquoise	0.67243155	1.06E-25	-0.6369222	1.93E-22	-0.7597526

KCNT2	turquoise	0.62175316	3.57E-21	-0.3492412	1.10E-06	-0.4932361
KCNS2	turquoise	0.64540814	3.52E-23	-0.7886062	1.67E-40	-0.4041139
KCNQ5	turquoise	0.59640045	3.33E-19	-0.5515766	4.06E-16	-0.795898
KCNQ3	turquoise	0.59258186	6.37E-19	-0.5772091	7.97E-18	-0.6371679
KCNQ2	turquoise	0.2486426	0.00064314	-0.616912	8.77E-21	-0.2505844
KCNN3	turquoise	-0.6017004	1.33E-19	0.43575949	5.67E-10	0.77473961
KCNN2	turquoise	0.59392507	5.08E-19	-0.4255439	1.56E-09	-0.4550219
KCNK1	turquoise	0.73453317	1.21E-32	-0.5949849	4.24E-19	-0.6758333
KCNJ8	turquoise	-0.3937334	2.95E-08	0.66405815	6.84E-25	0.50065119
KCNJ4	turquoise	0.33819811	2.50E-06	-0.6062695	5.98E-20	-0.1645008
KCNJ16	turquoise	-0.345919	1.42E-06	0.72171163	4.69E-31	0.49994306
KCNJ10	turquoise	-0.4050716	1.07E-08	0.56244426	7.99E-17	0.68034153
KCNIP4	turquoise	0.6619824	1.08E-24	-0.8026809	6.15E-43	-0.6019607
KCNH3	turquoise	-0.3236409	7.00E-06	-0.1259153	0.08766793	0.5687207
KCNH1	turquoise	0.65968131	1.77E-24	-0.6797752	1.97E-26	-0.7359581
KCNG3	turquoise	0.7453465	4.70E-34	-0.6906085	1.50E-27	-0.5729314
KCNE4	turquoise	-0.3243811	6.65E-06	0.40043703	1.63E-08	0.46682837
KCND2	turquoise	0.76279075	1.73E-36	-0.6427149	6.08E-23	-0.4651509
KCNB2	turquoise	0.58103182	4.31E-18	-0.7971256	5.92E-42	-0.4662484
KCNB1	turquoise	0.24571329	0.00074829	-0.619025	5.94E-21	-0.3633251
KCNAB1	turquoise	0.69697844	3.12E-28	-0.7475252	2.40E-34	-0.5183504
KCNA4	turquoise	0.45303726	9.48E-11	-0.552502	3.54E-16	-0.7348423
KBTBD7	turquoise	0.69444108	5.86E-28	-0.2910983	5.82E-05	-0.2804894
KBTBD6	turquoise	0.78464263	7.50E-40	-0.5116073	9.90E-14	-0.6977863
KBTBD11	turquoise	0.46758015	1.94E-11	-0.1904399	0.00941752	-0.4257289
KAZN	turquoise	-0.5199435	3.34E-14	0.1263341	0.08661042	0.65447314
KATNAL1	turquoise	0.57266085	1.64E-17	-0.6613285	1.24E-24	-0.3891583
KAT14	turquoise	0.51352436	7.73E-14	-0.4106538	6.41E-09	-0.6106034
KANK3	turquoise	-0.4877029	1.91E-12	0.46962498	1.55E-11	0.24710656
KANK2	turquoise	-0.520082	3.28E-14	0.80460249	2.76E-43	0.39688055
KANK1	turquoise	-0.5454458	9.90E-16	0.8403241	1.51E-50	0.13206372
KALRN	turquoise	0.64541206	3.52E-23	-0.6261554	1.56E-21	-0.6603956
JPH2	turquoise	-0.4825352	3.52E-12	0.22286729	0.00229435	0.68205013
JMJD8	turquoise	-0.5509307	4.46E-16	0.18768652	0.01051895	0.80105181
JKAMP	turquoise	0.55709632	1.79E-16	-0.0562854	0.44666945	-0.7578649
JDP2	turquoise	-0.3480165	1.21E-06	0.34164945	1.94E-06	0.85250647
JAZF1	turquoise	0.72892406	6.16E-32	-0.5501026	5.04E-16	-0.8242633
JAKMIP1	turquoise	0.68776982	2.98E-27	-0.6142116	1.44E-20	-0.7250091
JAK1	turquoise	0.22773241	0.00182349	0.25141778	0.00055626	-0.676439
JAGN1	turquoise	-0.4574467	5.91E-11	0.24045479	0.00097766	0.73552492
JAG2	turquoise	-0.387027	5.27E-08	-0.1583564	0.03132774	0.3616553
JADE3	turquoise	-0.4857631	2.41E-12	0.66393372	7.03E-25	0.63117736
JADE1	turquoise	0.59881918	2.20E-19	-0.7859868	4.52E-40	-0.2467654
IZUMO4	turquoise	-0.5310755	7.45E-15	0.25518063	0.00045571	0.63720895
ITPRIPL2	turquoise	-0.5315721	6.96E-15	0.48093801	4.24E-12	0.7741728
ITPRIP	turquoise	-0.5970475	2.98E-19	0.64360646	5.07E-23	0.74186813

ITPR1	turquoise	0.59236137	6.61E-19	-0.3896566	4.20E-08	-0.8192978
ITPKB	turquoise	-0.7596702	4.88E-36	0.63050267	6.77E-22	0.58204854
ITM2B	turquoise	0.2134441	0.00353224	0.15792822	0.03179417	-0.8317249
ITGB8	turquoise	-0.3164257	1.14E-05	0.47627515	7.27E-12	0.57086864
ITGB2	turquoise	-0.3629999	3.81E-07	0.64716909	2.45E-23	0.022518
ITGB1BP1	turquoise	0.31805641	1.03E-05	-0.3095847	1.80E-05	-0.6077382
ITGAX	turquoise	-0.3497721	1.06E-06	0.45723008	6.05E-11	0.35167585
ITGA8	turquoise	-0.3783813	1.09E-07	0.324039	6.81E-06	0.819013
ITGA5	turquoise	-0.6055348	6.81E-20	0.53341523	5.40E-15	0.62462731
ITGA10	turquoise	-0.5363207	3.61E-15	0.7271874	1.01E-31	0.67791483
ITFG2	turquoise	0.26535149	0.00026187	-0.4390783	4.05E-10	-0.5345487
ITFG1	turquoise	0.62366637	2.49E-21	-0.3990673	1.84E-08	-0.9279886
ISYNA1	turquoise	-0.5942549	4.80E-19	0.41825588	3.14E-09	0.55125202
ISOC1	turquoise	0.44353137	2.57E-10	0.04309772	0.56023669	-0.6540813
ISG20	turquoise	-0.3744401	1.52E-07	0.45003583	1.30E-10	0.5744634
ISCU	turquoise	0.53607049	3.73E-15	-0.4369532	5.02E-10	-0.9208494
ISCA1	turquoise	0.65039183	1.26E-23	-0.2926037	5.31E-05	-0.8348045
IRF7	turquoise	-0.6011358	1.47E-19	0.5346448	4.55E-15	0.74437942
IRF5	turquoise	-0.2208911	0.00251531	0.17478262	0.01733456	0.32238993
IRF1	turquoise	-0.24647	0.00071971	0.31180726	1.56E-05	0.53783454
IQGAP1	turquoise	-0.3672359	2.72E-07	0.74713257	2.71E-34	0.23723906
IQCB1	turquoise	0.47740422	6.38E-12	-0.1338915	0.06922206	-0.6952302
IPW	turquoise	0.60121666	1.45E-19	-0.4194982	2.79E-09	-0.3541318
IPO5	turquoise	0.67689136	3.84E-26	-0.4617516	3.70E-11	-0.7687161
INTU	turquoise	0.12792058	0.08269631	0.26672106	0.00024263	-0.2601669
INSM2	turquoise	0.50665339	1.86E-13	-0.751339	7.23E-35	-0.1900844
INSIG2	turquoise	0.71290122	5.14E-30	-0.4458512	2.02E-10	-0.6580326
INPPL1	turquoise	-0.6855942	5.01E-27	0.54922216	5.73E-16	0.84332598
INPP5K	turquoise	-0.5763865	9.09E-18	0.14171343	0.05433342	0.59506721
INPP5D	turquoise	-0.5095575	1.29E-13	0.68666375	3.88E-27	0.64100396
INPP4B	turquoise	0.74549782	4.49E-34	-0.542326	1.55E-15	-0.5698382
INHBB	turquoise	-0.5972295	2.89E-19	0.60953197	3.34E-20	0.71069321
INF2	turquoise	-0.4366508	5.18E-10	0.1825104	0.01290047	0.78401407
INA	turquoise	0.57712748	8.08E-18	-0.7387121	3.52E-33	-0.7268378
IMPDH1	turquoise	-0.7251843	1.78E-31	0.32061545	8.61E-06	0.4857908
IMPAD1	turquoise	0.6246152	2.09E-21	-0.2530482	0.00051041	-0.6584928
IMPA1	turquoise	0.50869425	1.44E-13	-0.2621823	0.00031195	-0.5235771
IMP3	turquoise	0.53382754	5.10E-15	-0.4310403	9.08E-10	-0.8063933
ILK	turquoise	-0.6347502	2.96E-22	0.65808515	2.49E-24	0.47911421
ILF3	turquoise	-0.5017827	3.44E-13	0.02392893	0.74646126	0.5588441
IL4R	turquoise	-0.5572454	1.75E-16	0.40300634	1.29E-08	0.729413
IL32	turquoise	-0.34595	1.41E-06	0.1369625	0.06302212	0.48907993
IL18BP	turquoise	-0.3204068	8.74E-06	0.17730646	0.01575922	0.43998612
IL17RB	turquoise	-0.479874	4.80E-12	0.80574811	1.71E-43	0.37109047
IL17D	turquoise	-0.4176672	3.32E-09	0.70535999	3.72E-29	0.60083105
IL13RA2	turquoise	0.76032582	3.93E-36	-0.5259237	1.50E-14	-0.5486801

IL10RB	turquoise	-0.4151289	4.22E-09	0.3316232	4.00E-06	0.81150733
IKZF3	turquoise	-0.4087043	7.67E-09	0.23636792	0.00119872	0.75458009
IGSF3	turquoise	0.7174667	1.50E-30	-0.7407141	1.93E-33	-0.4789131
IGFBP5	turquoise	-0.2835055	9.21E-05	0.28515239	8.35E-05	0.01160028
IGF2R	turquoise	-0.5661599	4.52E-17	0.59464747	4.49E-19	0.68405712
IGF2BP2	turquoise	-0.5044568	2.46E-13	0.4204396	2.55E-09	0.76112311
IGF1	turquoise	1	0	-0.4994094	4.62E-13	-0.4582529
IFT52	turquoise	0.61143522	2.38E-20	-0.2664795	0.00024592	-0.5939556
IFNAR1	turquoise	-0.4073882	8.66E-09	0.36002867	4.81E-07	0.74458961
IFITM3	turquoise	-0.6557629	4.08E-24	0.58470193	2.37E-18	0.16860703
IFITM2	turquoise	-0.701762	9.36E-29	0.63251847	4.58E-22	0.27931611
IFITM1	turquoise	-0.5698846	2.54E-17	0.58047734	4.71E-18	0.52292977
IFIT1	turquoise	0.49554819	7.42E-13	-0.410846	6.29E-09	-0.7190668
IFI35	turquoise	-0.4390362	4.07E-10	0.31629777	1.15E-05	0.65683956
IFI27L2	turquoise	-0.6548511	4.95E-24	0.50437849	2.48E-13	0.69625657
IFI27	turquoise	-0.4649269	2.61E-11	0.60617288	6.08E-20	0.21590553
IER5	turquoise	0.41022249	6.67E-09	-0.419526	2.78E-09	-0.6179125
IER3IP1	turquoise	0.74526448	4.82E-34	-0.2339791	0.00134826	-0.5409808
IER3	turquoise	-0.4063467	9.52E-09	0.37596744	1.34E-07	0.43691054
IDS	turquoise	0.31356857	1.39E-05	-0.2511531	0.00056405	-0.4677501
IDI1	turquoise	0.58358224	2.84E-18	-0.2045563	0.00522211	-0.4572324
IDH3A	turquoise	0.38316197	7.32E-08	-0.6739995	7.44E-26	-0.6439928
ID3	turquoise	-0.5551587	2.39E-16	0.69932412	1.74E-28	0.15464504
ID2	turquoise	0.56982035	2.56E-17	-0.1345084	0.06793817	-0.7073068
ID1	turquoise	-0.3594814	5.03E-07	0.52497876	1.71E-14	0.40558641
ICE1	turquoise	0.68241185	1.06E-26	-0.2744289	0.00015672	-0.5519859
ICAM2	turquoise	-0.5540974	2.80E-16	0.58079095	4.48E-18	0.40238857
ICA1L	turquoise	0.64498881	3.83E-23	-0.4870711	2.06E-12	-0.7426296
ICA1	turquoise	0.56399418	6.31E-17	-0.8225878	9.75E-47	-0.4688827
IBTK	turquoise	0.55557965	2.25E-16	-0.2834557	9.24E-05	-0.3363951
IARS	turquoise	0.51868588	3.94E-14	-0.2744064	0.00015692	-0.9197413
HVCN1	turquoise	-0.495039	7.89E-13	0.6067927	5.45E-20	0.42436939
HTR4	turquoise	0.45023609	1.28E-10	-0.6930036	8.35E-28	-0.1988563
HTR2C	turquoise	0.66176665	1.13E-24	-0.4127088	5.29E-09	-0.5744259
HSPH1	turquoise	0.42697479	1.35E-09	-0.2591752	0.00036757	-0.5361163
HSPB3	turquoise	0.63550213	2.56E-22	-0.7688675	2.19E-37	-0.4636766
HSPB2	turquoise	-0.5880029	1.37E-18	0.55534248	2.33E-16	0.64211516
HSPB11	turquoise	0.61377785	1.55E-20	-0.2541733	0.00048084	-0.708748
HSPB1	turquoise	-0.6417876	7.33E-23	0.5565204	1.95E-16	0.50675737
HSPA8	turquoise	0.2596095	0.000359	0.14916835	0.0427126	-0.6767535
HSPA7	turquoise	-0.2194083	0.0026936	0.31916185	9.51E-06	0.40291819
HSPA1A	turquoise	-0.2900814	6.20E-05	0.47083769	1.35E-11	0.35667116
HSPA13	turquoise	0.59838941	2.37E-19	-0.3575451	5.84E-07	-0.0672068
HSPA12B	turquoise	-0.491368	1.23E-12	0.44576218	2.04E-10	0.60073133
HSPA12A	turquoise	0.63051762	6.75E-22	-0.7041302	5.11E-29	-0.4230977
HSP90AB1	turquoise	0.25753943	0.00040155	-0.4224646	2.10E-09	-0.6613266

HSP90AA1	turquoise	0.32047348	8.70E-06	0.03540253	0.63235645	-0.4864389
HSF2	turquoise	0.66894195	2.33E-25	-0.3649025	3.28E-07	-0.7379744
HSD17B12	turquoise	0.15123002	0.03989422	0.30936335	1.83E-05	-0.4622111
HSD17B11	turquoise	0.56325677	7.06E-17	-0.001898	0.97954451	-0.5592953
HS6ST2	turquoise	0.76980589	1.58E-37	-0.5518138	3.92E-16	-0.6995252
HS3ST2	turquoise	0.4594997	4.73E-11	-0.6713787	1.35E-25	-0.4492401
HS1BP3	turquoise	-0.5723178	1.73E-17	0.5696697	2.62E-17	0.3324874
HPRT1	turquoise	0.69596454	4.02E-28	-0.6711338	1.42E-25	-0.8269382
HPF1	turquoise	0.64622896	2.97E-23	-0.2538051	0.00049033	-0.7071652
HPCAL4	turquoise	0.55855274	1.44E-16	-0.7073599	2.22E-29	-0.4974969
HPCAL1	turquoise	0.28898161	6.62E-05	-0.6296232	8.02E-22	-0.2336409
HP1BP3	turquoise	-0.5329705	5.74E-15	0.43866396	4.22E-10	0.53511529
HOXB6	turquoise	-0.575358	1.07E-17	0.3608563	4.51E-07	0.61395251
HOPX	turquoise	0.51677989	5.06E-14	-0.2324493	0.0014528	-0.8307452
HOMER3	turquoise	-0.5008204	3.87E-13	0.33617662	2.89E-06	0.75350994
HOMER1	turquoise	0.77744621	1.06E-38	-0.587721	1.44E-18	-0.6415299
HNRNPUL2	turquoise	-0.396685	2.27E-08	0.05882062	0.42643133	0.72572164
HNRNPR	turquoise	0.32951921	4.65E-06	0.08553001	0.24703895	-0.7731927
HNRNPK	turquoise	0.57062423	2.26E-17	-0.1879783	0.01039708	-0.8573025
HNRNPH2	turquoise	0.73208183	2.48E-32	-0.2293631	0.00168657	-0.6027528
HNRNPDL	turquoise	0.35953272	5.01E-07	-0.0626592	0.39681994	-0.6696422
HNRNPD	turquoise	0.3819363	8.12E-08	-0.0357323	0.62918675	-0.3825352
HNRNPC	turquoise	0.60168435	1.34E-19	-0.3805539	9.12E-08	-0.4684986
HNRNPA0	turquoise	0.54493911	1.06E-15	-0.1296047	0.07869828	-0.8260967
HN1	turquoise	0.68554766	5.06E-27	-0.6092559	3.51E-20	-0.8527127
HMP19	turquoise	0.61701528	8.60E-21	-0.8042495	3.20E-43	-0.5479196
HMOX1	turquoise	-0.3241331	6.77E-06	0.43639119	5.32E-10	0.34856601
HMGN4	turquoise	0.50070651	3.93E-13	-0.0672138	0.36332973	-0.6815102
HMGCS1	turquoise	0.57976753	5.28E-18	-0.622043	3.38E-21	-0.3023593
HMGCR	turquoise	0.54906146	5.86E-16	-0.7131334	4.83E-30	-0.2677988
HMGCLL1	turquoise	0.71261286	5.55E-30	-0.5226017	2.35E-14	-0.8025724
HMBOX1	turquoise	-0.4901621	1.42E-12	0.55112377	4.34E-16	0.508274
HLTF	turquoise	0.52416337	1.90E-14	-0.1095324	0.13776303	-0.6822491
HLF	turquoise	0.66134978	1.23E-24	-0.5256578	1.56E-14	-0.7355974
HLA-E	turquoise	-0.5939003	5.10E-19	0.66727369	3.37E-25	0.21784557
HLA-DRB6	turquoise	-0.2630547	0.00029734	0.39705967	2.20E-08	-0.1238483
HLA-DMB	turquoise	-0.2865896	7.66E-05	0.56681505	4.09E-17	0.38776305
HLA-DMA	turquoise	-0.4781236	5.87E-12	0.43376967	6.92E-10	0.64784214
HK1	turquoise	0.47454736	8.86E-12	-0.7360832	7.69E-33	-0.630314
HIVEP1	turquoise	0.5944932	4.61E-19	-0.2537413	0.000492	-0.8176872
HIST2H2BE	turquoise	-0.4133509	4.98E-09	0.65479181	5.02E-24	0.23496747
HIST2H2AC	turquoise	-0.3993778	1.79E-08	0.33574542	2.98E-06	0.71844132
HIST1H2BK	turquoise	-0.21487	0.00331271	0.497404	5.91E-13	0.50890966
HIST1H2BD	turquoise	-0.2795408	0.00011644	0.58827221	1.31E-18	0.27583223
HIPK2	turquoise	-0.7196378	8.31E-31	0.60309518	1.05E-19	0.42090929
HINT1	turquoise	0.48731271	2.00E-12	-0.2747327	0.000154	-0.9371304

HIKESHI	turquoise	0.5201146	3.26E-14	-0.1035662	0.16065259	-0.7076176
HIGD2A	turquoise	-0.5484409	6.42E-16	0.3797403	9.76E-08	0.30106285
HIGD1A	turquoise	0.48891312	1.65E-12	-0.1216827	0.09894648	-0.8234383
HIF3A	turquoise	-0.6736907	7.98E-26	0.44835088	1.56E-10	0.41092033
HHATL	turquoise	-0.252811	0.00051685	0.02825678	0.702606	0.68085766
HEYL	turquoise	-0.5435275	1.30E-15	0.67650391	4.20E-26	0.61441072
HEY1	turquoise	0.47681334	6.83E-12	-0.4478305	1.64E-10	-0.2976638
HEXB	turquoise	0.43193375	8.31E-10	-0.1424553	0.05307058	-0.7394247
HES4	turquoise	-0.2267481	0.00191096	0.17076794	0.02012283	0.72158748
HERC6	turquoise	0.32446168	6.61E-06	-0.4028497	1.31E-08	-0.7082726
HERC5	turquoise	-0.5087914	1.42E-13	0.6129743	1.80E-20	0.73277052
HERC3	turquoise	0.46448497	2.74E-11	-0.217879	0.00288941	-0.897006
HERC2P2	turquoise	-0.31629	1.15E-05	0.45310741	9.41E-11	0.50795797
HEPACAM	turquoise	-0.6085965	3.95E-20	0.7002006	1.39E-28	0.52447125
HENMT1	turquoise	0.70900633	1.44E-29	-0.6599057	1.69E-24	-0.7628208
HELZ2	turquoise	-0.555162	2.39E-16	0.59476816	4.40E-19	0.63048607
HECTD4	turquoise	0.44498219	2.21E-10	-0.4808665	4.27E-12	-0.8512607
HECTD1	turquoise	0.53204638	6.52E-15	-0.1272005	0.08445484	-0.8356875
HEATR5B	turquoise	0.60391543	9.05E-20	-0.3095364	1.81E-05	-0.8236877
HDGFRP3	turquoise	0.63533356	2.64E-22	-0.1586173	0.03104642	-0.5441464
HDAC1	turquoise	-0.7154113	2.62E-30	0.69985133	1.52E-28	0.73647115
HCP5	turquoise	-0.4390652	4.06E-10	0.58616345	1.86E-18	0.41326335
HCN1	turquoise	0.60913669	3.59E-20	-0.6723842	1.07E-25	-0.7641345
HCCS	turquoise	0.63082805	6.36E-22	-0.5285781	1.05E-14	-0.8127633
HBP1	turquoise	-0.4723647	1.13E-11	0.74809526	2.01E-34	0.70279704
HBEGF	turquoise	-0.4672336	2.02E-11	0.46140694	3.84E-11	0.36845419
HAVCR2	turquoise	-0.4093498	7.23E-09	0.58475683	2.34E-18	0.53906729
HAUS7	turquoise	-0.4701516	1.46E-11	0.09219615	0.21197102	0.84686238
HAUS4	turquoise	-0.6455299	3.43E-23	0.48774624	1.90E-12	0.51961828
HAGHL	turquoise	-0.3444267	1.58E-06	0.04267915	0.56405594	0.71669654
HADHA	turquoise	-0.368464	2.46E-07	0.15597861	0.03399314	0.81481334
HACL1	turquoise	0.61332035	1.69E-20	-0.5425036	1.51E-15	-0.4090338
HACD3	turquoise	0.48398448	2.97E-12	-0.0665852	0.36784456	-0.7837374
H3F3B	turquoise	-0.3770688	1.22E-07	0.26412995	0.00028021	0.54821617
H2AFZ	turquoise	0.46905065	1.65E-11	-0.0972915	0.18768248	-0.7782265
H2AFY	turquoise	0.55311639	3.24E-16	-0.5128078	8.48E-14	-0.4653407
H2AFJ	turquoise	-0.4238071	1.84E-09	0.44091392	3.36E-10	0.54260487
H1F0	turquoise	-0.3711888	1.98E-07	0.13921	0.05877942	0.08815641
GYPC	turquoise	-0.509711	1.26E-13	0.62506197	1.92E-21	0.49138766
GUSBP2	turquoise	-0.5295851	9.14E-15	0.2093871	0.00423044	0.3331347
GUSB	turquoise	-0.5861197	1.87E-18	0.22705727	0.00188308	0.56792531
GUF1	turquoise	0.66876968	2.42E-25	-0.3497577	1.06E-06	-0.520337
GUCY1B3	turquoise	0.4517364	1.09E-10	-0.7077854	1.98E-29	-0.1290684
GUCD1	turquoise	-0.6656524	4.82E-25	0.61402395	1.49E-20	0.81949482
GTF2H1	turquoise	0.807329	8.74E-44	-0.4346999	6.30E-10	-0.3612295
GTF2A2	turquoise	0.23004092	0.00163246	0.22796621	0.00180325	-0.6693478

GSTP1	turquoise	-0.5719564	1.83E-17	0.54177642	1.67E-15	0.73705533
GSTM3	turquoise	-0.3342773	3.31E-06	0.36169527	4.22E-07	0.64820859
GSTM2	turquoise	-0.4150107	4.27E-09	0.33519562	3.10E-06	0.6656743
GSTM1	turquoise	-0.4148968	4.31E-09	0.31896592	9.64E-06	0.67589145
GSPT2	turquoise	0.72209852	4.21E-31	-0.5423523	1.54E-15	-0.7082135
GSN	turquoise	-0.5727105	1.63E-17	0.65680197	3.28E-24	0.06005297
GSKIP	turquoise	0.5676903	3.57E-17	-0.4765149	7.07E-12	-0.8467626
GRWD1	turquoise	-0.3141452	1.33E-05	-0.0864603	0.24191703	0.72213443
GRPEL2	turquoise	0.64082494	8.89E-23	-0.3247145	6.50E-06	-0.6843224
GRP	turquoise	0.59596309	3.59E-19	-0.5323351	6.27E-15	-0.3711233
GRM8	turquoise	0.62596321	1.62E-21	-0.6220044	3.41E-21	-0.517144
GRK5	turquoise	-0.2293947	0.00168401	0.23373571	0.00136442	0.55781925
GRIN3A	turquoise	0.73203605	2.51E-32	-0.6891138	2.15E-27	-0.277913
GRIN2C	turquoise	-0.5975152	2.75E-19	0.49818451	5.37E-13	0.78499766
GRIN2A	turquoise	0.79135255	5.79E-41	-0.6746331	6.44E-26	-0.3406336
GRIA4	turquoise	0.68034772	1.72E-26	-0.669585	2.01E-25	-0.6172453
GRIA2	turquoise	0.50694115	1.80E-13	-0.5601484	1.13E-16	-0.6587311
GREM2	turquoise	0.64978299	1.43E-23	-0.6658859	4.58E-25	-0.4666487
GRB14	turquoise	0.59665811	3.19E-19	-0.4913742	1.23E-12	-0.4509538
GPT2	turquoise	-0.5355484	4.02E-15	0.63643654	2.13E-22	0.76439258
GPRC5B	turquoise	-0.5114189	1.01E-13	0.54327837	1.35E-15	0.68179622
GPRASP2	turquoise	0.66831808	2.67E-25	-0.7219369	4.41E-31	-0.7786593
GPRASP1	turquoise	0.8308054	1.90E-48	-0.5978467	2.60E-19	-0.5157252
GPR88	turquoise	0.71845116	1.15E-30	-0.7103645	1.01E-29	-0.6178666
GPR4	turquoise	-0.495769	7.22E-13	0.61803955	7.12E-21	0.55647231
GPR37L1	turquoise	-0.6102212	2.96E-20	0.58695437	1.63E-18	0.79788536
GPR22	turquoise	0.70222406	8.32E-29	-0.5857479	1.99E-18	-0.7456104
GPR19	turquoise	0.51765893	4.51E-14	-0.2298122	0.00165054	-0.6499065
GPR137C	turquoise	0.70173732	9.42E-29	-0.5677199	3.55E-17	-0.7241597
GPR137	turquoise	-0.6168988	8.79E-21	0.30695963	2.14E-05	0.70351066
GPN1	turquoise	0.43936484	3.93E-10	-0.291837	5.56E-05	-0.6341774
GPM6A	turquoise	0.49130879	1.24E-12	-0.310783	1.67E-05	-0.7958452
GPIHBP1	turquoise	-0.3408734	2.06E-06	0.10946774	0.13799691	0.5767048
GPER1	turquoise	-0.5154287	6.04E-14	0.66986073	1.89E-25	0.74514198
GPC2	turquoise	-0.3301211	4.45E-06	0.3395419	2.27E-06	0.72302057
GPATCH2	turquoise	0.73598721	7.92E-33	-0.6130671	1.77E-20	-0.4939938
GPAM	turquoise	-0.3319076	3.92E-06	0.67868502	2.54E-26	0.54082344
GOT2	turquoise	0.52780589	1.16E-14	-0.7515869	6.68E-35	-0.731925
GOT1	turquoise	0.65863152	2.22E-24	-0.6965859	3.44E-28	-0.8150272
GOPC	turquoise	0.70949152	1.27E-29	-0.3832257	7.28E-08	-0.6833645
GOLT1B	turquoise	0.56874232	3.03E-17	-0.2490744	0.00062884	-0.7763335
GOLPH3L	turquoise	0.35057442	9.97E-07	-0.0962368	0.19253486	-0.6444581
GOLIM4	turquoise	-0.5712159	2.06E-17	0.62919593	8.71E-22	0.57152301
GOLGA8B	turquoise	0.51512346	6.28E-14	-0.549714	5.33E-16	-0.4863359
GNPNAT1	turquoise	-0.3036754	2.64E-05	0.06665836	0.3673174	0.7443688
GNPDA2	turquoise	0.54139311	1.77E-15	-0.0322841	0.66265586	-0.6523318

GNL3	turquoise	0.60495728	7.54E-20	-0.5799669	5.12E-18	-0.573277
GNL1	turquoise	-0.3981812	1.99E-08	0.19946383	0.0064884	0.83001485
GNG7	turquoise	-0.7199934	7.54E-31	0.41385132	4.76E-09	0.40947518
GNG3	turquoise	0.54786651	6.98E-16	-0.7597582	4.74E-36	-0.1557485
GNG2	turquoise	0.6360305	2.30E-22	-0.4996344	4.49E-13	-0.8775472
GNG10	turquoise	0.33240706	3.79E-06	-0.0177624	0.81034831	-0.7938927
GNB5	turquoise	0.60813995	4.29E-20	-0.5237257	2.02E-14	-0.5644879
GNAI1	turquoise	0.53971203	2.24E-15	-0.4025421	1.35E-08	-0.5611706
GNA12	turquoise	-0.5982663	2.42E-19	0.77954377	4.94E-39	0.72743165
GNA11	turquoise	-0.5945746	4.55E-19	0.20736155	0.00462354	0.57666254
GMPR2	turquoise	0.5186506	3.96E-14	-0.1476928	0.04483064	-0.7469356
GMPR	turquoise	-0.4730826	1.05E-11	0.56789972	3.46E-17	0.5570894
GLTSCR2	turquoise	-0.4596052	4.68E-11	0.44320669	2.65E-10	0.65888117
GLT8D2	turquoise	0.57827992	6.71E-18	-0.4621374	3.55E-11	-0.704359
GLS2	turquoise	0.60661749	5.62E-20	-0.7366307	6.54E-33	-0.6310819
GLS	turquoise	0.60090043	1.53E-19	-0.7172335	1.60E-30	-0.5081912
GLRX5	turquoise	0.43763821	4.69E-10	-0.0541561	0.46407826	-0.4927748
GLRX3	turquoise	0.3801016	9.47E-08	-0.0175459	0.81261773	-0.8306951
GLRX2	turquoise	0.62124405	3.93E-21	-0.4141757	4.61E-09	-0.4217404
GLRX	turquoise	0.47859773	5.56E-12	-0.319005	9.61E-06	-0.8228451
GLRB	turquoise	0.62165704	3.64E-21	-0.5170345	4.90E-14	-0.8907506
GLRA2	turquoise	0.74087928	1.84E-33	-0.6141156	1.46E-20	-0.6658371
GLMN	turquoise	0.74231437	1.19E-33	-0.6712125	1.40E-25	-0.4818565
GLIPR2	turquoise	-0.3131416	1.42E-05	0.19704733	0.00718002	0.65248459
GLI3	turquoise	-0.5416458	1.70E-15	0.64356365	5.12E-23	0.49139992
GLG1	turquoise	-0.2909244	5.88E-05	0.25305277	0.00051028	0.81561158
GLCE	turquoise	0.68454534	6.43E-27	-0.5169591	4.94E-14	-0.6323491
GJC2	turquoise	-0.4881979	1.80E-12	0.01925967	0.794703	0.49770451
GJA4	turquoise	-0.5653804	5.10E-17	0.7373064	5.36E-33	0.42877592
GIMAP8	turquoise	-0.5072029	1.74E-13	0.72674839	1.14E-31	0.58019545
GIMAP7	turquoise	-0.5567603	1.88E-16	0.67833678	2.75E-26	0.55546335
GIMAP5	turquoise	-0.6800148	1.86E-26	0.49302466	1.01E-12	0.47425047
GIMAP4	turquoise	-0.4964295	6.66E-13	0.74516648	4.97E-34	0.35320264
GGNBP2	turquoise	0.53849908	2.66E-15	-0.285461	8.19E-05	-0.7523905
GGCT	turquoise	0.64632252	2.92E-23	-0.4782007	5.82E-12	-0.8009505
GFRA2	turquoise	0.24500317	0.00077607	-0.6275455	1.20E-21	0.15129486
GFPT1	turquoise	0.66760844	3.13E-25	-0.5191171	3.72E-14	-0.4056757
GFM1	turquoise	0.45666894	6.43E-11	-0.1855728	0.01144008	-0.4305341
GFAP	turquoise	-0.6977918	2.55E-28	0.50205821	3.32E-13	0.42793987
GEMIN2	turquoise	0.7177388	1.40E-30	-0.3577636	5.74E-07	-0.662032
GDPD3	turquoise	-0.5337202	5.18E-15	0.38030885	9.31E-08	0.67914531
GDI2	turquoise	0.53910191	2.44E-15	-0.073089	0.32280854	-0.5572701
GDF11	turquoise	-0.5766967	8.65E-18	0.50823166	1.53E-13	0.83535811
GDF10	turquoise	0.32806972	5.14E-06	-0.240495	0.00097569	-0.6755532
GDAP1	turquoise	0.60796148	4.43E-20	-0.6126075	1.92E-20	-0.5333288
GDA	turquoise	0.55509217	2.42E-16	-0.6567575	3.31E-24	-0.3229802

GCNT1	turquoise	0.63727444	1.80E-22	-0.6083242	4.15E-20	-0.3639503
GCA	turquoise	0.37088196	2.03E-07	-0.0505911	0.49404626	-0.6889624
GBP2	turquoise	-0.4907302	1.33E-12	0.48341587	3.17E-12	0.32065103
GBGT1	turquoise	-0.4293037	1.08E-09	0.60199818	1.27E-19	0.57582959
GBE1	turquoise	0.43736309	4.82E-10	0.10107965	0.17099228	-0.6969658
GBAS	turquoise	0.34797195	1.21E-06	0.0386003	0.6019093	-0.3981331
GATS	turquoise	-0.4922074	1.11E-12	0.43842698	4.33E-10	0.74687596
GATB	turquoise	0.50430606	2.51E-13	-0.520191	3.23E-14	-0.0506413
GATAD2A	turquoise	-0.68942	2.00E-27	0.6407266	9.06E-23	0.71720535
GAS6	turquoise	-0.6272364	1.27E-21	0.23127923	0.0015377	0.41313397
GAS2L3	turquoise	-0.4273352	1.31E-09	0.32693052	5.57E-06	0.81229683
GAS2	turquoise	0.72700794	1.06E-31	-0.4682987	1.79E-11	-0.4576101
GARS	turquoise	0.56962701	2.64E-17	-0.6734629	8.41E-26	-0.7313379
GAMT	turquoise	-0.5273308	1.24E-14	-0.0393407	0.5949541	0.59539693
GALNTL5	turquoise	0.68306424	9.12E-27	-0.4613092	3.88E-11	-0.4249996
GALNT15	turquoise	-0.3828018	7.55E-08	0.49792108	5.55E-13	0.23072382
GALNT11	turquoise	0.32551972	6.15E-06	-0.5936323	5.33E-19	-0.3978174
GAL3ST4	turquoise	-0.349323	1.10E-06	0.62679712	1.38E-21	0.69583135
GAD2	turquoise	0.74276757	1.04E-33	-0.822243	1.14E-46	-0.536896
GAD1	turquoise	0.74662691	3.17E-34	-0.7379147	4.47E-33	-0.7015483
GABRG2	turquoise	0.68245306	1.05E-26	-0.7192993	9.12E-31	-0.6338072
GABRD	turquoise	0.3922097	3.36E-08	-0.8515736	3.25E-53	-0.2795071
GABRA5	turquoise	0.60583382	6.46E-20	-0.4837496	3.05E-12	-0.854725
GABRA2	turquoise	0.41185234	5.73E-09	-0.0927461	0.20924622	-0.6562045
GABRA1	turquoise	0.76758762	3.40E-37	-0.7031799	6.52E-29	-0.7103382
GABBR2	turquoise	0.60294087	1.07E-19	-0.7001745	1.40E-28	-0.6666482
GABARAPL[illegible]	turquoise	0.38121491	8.63E-08	-0.0333138	0.65258723	-0.8751755
GABARAPL[illegible]	turquoise	0.61163978	2.29E-20	-0.7090878	1.41E-29	-0.8504598
GAB2	turquoise	-0.5655305	4.98E-17	0.39733193	2.14E-08	0.48567168
G3BP2	turquoise	0.51602861	5.58E-14	-0.3991888	1.82E-08	-0.6971536
FZD9	turquoise	-0.5316904	6.85E-15	0.56593184	4.69E-17	0.77033477
FZD4	turquoise	-0.5907407	8.68E-19	0.57619592	9.37E-18	0.6998871
FZD3	turquoise	0.57329596	1.49E-17	-0.4035485	1.23E-08	-0.7824537
FYN	turquoise	-0.5920226	7.00E-19	0.69202127	1.06E-27	-0.0167852
FYCO1	turquoise	-0.5708664	2.18E-17	0.78258201	1.62E-39	0.75622836
FXYD7	turquoise	-0.1700457	0.02066362	-0.4464344	1.90E-10	0.21108726
FXYD6	turquoise	0.51585061	5.72E-14	-0.8287646	5.15E-48	-0.5331673
FXYD5	turquoise	-0.5960532	3.53E-19	0.49621251	6.84E-13	0.77139383
FUT9	turquoise	0.35027361	1.02E-06	0.15644687	0.03345355	-0.5794494
FUNDC1	turquoise	0.57183304	1.87E-17	-0.2062618	0.00485043	-0.6519105
FUCA1	turquoise	0.58193864	3.72E-18	-0.2987019	3.63E-05	-0.4922565
FTL	turquoise	-0.5413859	1.77E-15	0.62178588	3.55E-21	-0.0524624
FSTL5	turquoise	0.72712003	1.03E-31	-0.7224009	3.87E-31	-0.4936502
FSTL3	turquoise	-0.5939701	5.04E-19	0.242336	0.00088907	0.45086655
FSTL1	turquoise	-0.4957631	7.23E-13	0.72932113	5.50E-32	0.27711475
FSD1	turquoise	-0.3374253	2.64E-06	-0.1812964	0.0135232	0.53007354

FRZB	turquoise	-0.2005151	0.00620655	0.58934884	1.10E-18	0.36871308
FRRS1L	turquoise	0.48903568	1.63E-12	-0.5189489	3.81E-14	-0.5704412
FRMPD4	turquoise	0.55582284	2.17E-16	-0.5753325	1.08E-17	-0.6721704
FRMPD2	turquoise	0.50560223	2.13E-13	-0.7031174	6.62E-29	-0.2889747
FRMD8	turquoise	-0.3198509	9.08E-06	0.24506697	0.00077353	0.75426031
FRMD4A	turquoise	0.49849909	5.16E-13	-0.0366807	0.62010828	-0.5906766
FRG1	turquoise	0.56773039	3.55E-17	-0.5065671	1.88E-13	-0.0603328
FRAT2	turquoise	-0.3476816	1.24E-06	0.54789353	6.95E-16	0.16155456
FRAS1	turquoise	0.45579394	7.06E-11	-0.5264929	1.39E-14	-0.3230155
FOXRED2	turquoise	-0.5224703	2.39E-14	0.16752874	0.02264769	0.8026421
FOXQ1	turquoise	-0.2982122	3.74E-05	0.52745895	1.22E-14	0.56992246
FOXO4	turquoise	-0.7297353	4.88E-32	0.59966716	1.90E-19	0.71305793
FOXO1	turquoise	-0.5025017	3.14E-13	0.82162497	1.53E-46	0.68605074
FOXN3	turquoise	-0.481801	3.83E-12	0.80746756	8.24E-44	0.4707493
FOXJ3	turquoise	0.63930488	1.20E-22	-0.6946301	5.60E-28	-0.4947579
FOXJ1	turquoise	-0.4523034	1.02E-10	0.57483933	1.16E-17	0.58047392
FOXG1	turquoise	0.4826576	3.47E-12	-0.1229595	0.09542927	-0.4817822
FOXF1	turquoise	-0.4521441	1.04E-10	0.62001629	4.94E-21	0.51930651
FOXD1	turquoise	-0.4721846	1.16E-11	0.79122637	6.08E-41	0.52245693
FOXC1	turquoise	-0.679025	2.35E-26	0.65713514	3.05E-24	0.79160891
FOSL2	turquoise	-0.3540498	7.65E-07	0.20794379	0.00450731	0.77811397
FOPNL	turquoise	0.46714646	2.04E-11	-0.1071387	0.14662739	-0.685127
FNBP4	turquoise	0.32159872	8.05E-06	-0.0780164	0.29117146	-0.6223983
FN3KRP	turquoise	0.34563671	1.45E-06	-0.4482313	1.57E-10	-0.2157545
FMO6P	turquoise	-0.527313	1.24E-14	0.28208156	0.00010024	0.7117905
FMO3	turquoise	-0.3391829	2.33E-06	0.72372129	2.68E-31	0.26067924
FMN2	turquoise	0.3753384	1.41E-07	0.14439437	0.0498845	-0.6697944
FLVCR2	turquoise	-0.4982051	5.36E-13	0.38689976	5.33E-08	0.68084883
FLRT3	turquoise	0.6785338	2.63E-26	-0.672026	1.16E-25	-0.4413532
FLRT2	turquoise	0.26705825	0.0002381	0.07498608	0.31037421	-0.505456
FLNB	turquoise	-0.5338203	5.11E-15	0.37537229	1.40E-07	0.39371708
FKBP5	turquoise	-0.5986192	2.27E-19	0.65401524	5.91E-24	0.29557312
FKBP1B	turquoise	0.62066148	4.38E-21	-0.6639404	7.02E-25	-0.7529214
FIP1L1	turquoise	-0.5544895	2.64E-16	0.20884055	0.00433344	0.2751558
FILIP1	turquoise	0.47565027	7.81E-12	-0.3950021	2.63E-08	-0.6500871
FIG4	turquoise	0.47407817	9.34E-12	-0.6428252	5.94E-23	-0.4661048
FHOD1	turquoise	-0.5177574	4.45E-14	0.37119699	1.98E-07	0.67336657
FHL2	turquoise	0.66264051	9.32E-25	-0.6297797	7.79E-22	-0.5674008
FHL1	turquoise	0.39028773	3.98E-08	-0.1528437	0.03779894	-0.8052142
FH	turquoise	0.72088323	5.90E-31	-0.5408794	1.90E-15	-0.3812269
FGR	turquoise	-0.5519112	3.87E-16	0.6552327	4.57E-24	0.75810705
FGL1	turquoise	-0.5781754	6.83E-18	0.26820917	0.00022322	0.69553454
FGFRL1	turquoise	-0.6896738	1.88E-27	0.62247796	3.12E-21	0.34279428
FGFR3	turquoise	-0.3979774	2.02E-08	0.37089643	2.02E-07	0.70021665
FGFR1OP2	turquoise	0.6658242	4.64E-25	-0.3923805	3.32E-08	-0.5324201
FGF9	turquoise	0.46429942	2.80E-11	-0.7577805	9.08E-36	-0.1540634

FGF2	turquoise	-0.3979522	2.03E-08	0.7879163	2.17E-40	0.35677009
FGF14	turquoise	0.66282141	8.96E-25	-0.5466257	8.35E-16	-0.6838585
FGF12	turquoise	0.64893093	1.71E-23	-0.702367	8.02E-29	-0.7332209
FGF1	turquoise	-0.5283707	1.08E-14	0.53209346	6.48E-15	0.21067858
FGD2	turquoise	-0.3929895	3.14E-08	0.61023265	2.95E-20	0.4610194
FEZ2	turquoise	0.56057305	1.06E-16	-0.4602203	4.37E-11	-0.6620469
FES	turquoise	-0.5890474	1.15E-18	0.46921411	1.62E-11	0.61395847
FERMT3	turquoise	-0.4161995	3.81E-09	0.4705469	1.39E-11	0.4089736
FEN1	turquoise	0.53558696	3.99E-15	-0.8411682	9.69E-51	-0.3710645
FCRLB	turquoise	0.67592984	4.79E-26	-0.5022856	3.23E-13	-0.6967982
FCN3	turquoise	-0.2870077	7.47E-05	0.41077896	6.33E-09	0.39727787
FCGRT	turquoise	-0.6058484	6.44E-20	0.70103219	1.13E-28	0.22393673
FCGR3B	turquoise	-0.5375305	3.05E-15	0.41776511	3.29E-09	0.77776875
FCGBP	turquoise	-0.4335468	7.07E-10	0.52910371	9.76E-15	0.29977029
FBXW7	turquoise	0.6097445	3.22E-20	-0.6514749	1.01E-23	-0.631423
FBXO41	turquoise	-0.4794769	5.02E-12	0.18647867	0.01103696	0.74674311
FBXO34	turquoise	0.67775462	3.15E-26	-0.5508687	4.50E-16	-0.8050291
FBXO33	turquoise	0.60318113	1.03E-19	-0.4820935	3.70E-12	-0.5270648
FBXO3	turquoise	0.50465107	2.40E-13	-0.0819322	0.26755506	-0.7952544
FBXO28	turquoise	0.60538537	6.99E-20	-0.2857616	8.05E-05	-0.5057184
FBXO21	turquoise	0.43242493	7.91E-10	-0.1393693	0.05848792	-0.6347402
FBXO2	turquoise	-0.5563041	2.02E-16	0.35040569	1.01E-06	0.11531878
FBXO11	turquoise	0.63258366	4.53E-22	-0.2832511	9.35E-05	-0.6047129
FBXL7	turquoise	-0.5282339	1.10E-14	0.6397037	1.11E-22	0.77360234
FBXL5	turquoise	0.32207899	7.79E-06	0.17706306	0.0159055	-0.7144852
FBXL2	turquoise	0.64942044	1.54E-23	-0.7196061	8.38E-31	-0.6722287
FBXL18	turquoise	-0.2033379	0.00550304	-0.0974102	0.18714195	0.55464233
FBXL14	turquoise	0.57139609	2.00E-17	-0.5517043	3.98E-16	-0.6704572
FBRS	turquoise	-0.6027593	1.11E-19	0.24082783	0.00095947	0.23849314
FBLN1	turquoise	-0.5650052	5.40E-17	0.50666313	1.86E-13	0.33480185
FAXC	turquoise	0.56576081	4.81E-17	-0.6867463	3.80E-27	-0.8195868
FAU	turquoise	-0.5600808	1.14E-16	0.57380286	1.37E-17	0.61175266
FAT1	turquoise	-0.3579875	5.65E-07	0.78661942	3.56E-40	0.54187233
FASTKD3	turquoise	0.5358377	3.86E-15	-0.2535135	0.00049798	-0.8189262
FASTKD2	turquoise	0.58853269	1.26E-18	-0.2610087	0.00033266	-0.8250245
FASTK	turquoise	-0.2528001	0.00051715	0.13226182	0.07270872	0.79811246
FAR2	turquoise	0.67615127	4.55E-26	-0.6063469	5.90E-20	-0.7168723
FANCL	turquoise	0.66124313	1.26E-24	-0.4066439	9.27E-09	-0.7072079
FANCB	turquoise	-0.4072353	8.78E-09	0.29681167	4.09E-05	0.77232266
FAM92A1	turquoise	0.61647005	9.51E-21	-0.3053335	2.38E-05	-0.8228518
FAM89A	turquoise	-0.4810865	4.17E-12	0.68703115	3.55E-27	0.08478249
FAM84B	turquoise	-0.4014397	1.49E-08	0.71685039	1.78E-30	0.22877526
FAM81A	turquoise	0.53151472	7.02E-15	-0.7077334	2.01E-29	-0.2295636
FAM72B	turquoise	-0.3611284	4.42E-07	0.21353846	0.00351731	0.77955061
FAM69C	turquoise	-0.2130291	0.00359855	0.05899682	0.42504473	0.46267145
FAM69A	turquoise	0.53295349	5.76E-15	-0.4283225	1.19E-09	-0.4424605

FAM65C	turquoise	-0.5671464	3.88E-17	0.70328925	6.34E-29	0.64881361
FAM53B	turquoise	-0.6175407	7.81E-21	0.1499903	0.04156956	0.40392469
FAM50B	turquoise	0.36333273	3.71E-07	-0.1742969	0.01765299	-0.8677385
FAM49B	turquoise	0.73347164	1.66E-32	-0.3824719	7.76E-08	-0.6281483
FAM46A	turquoise	-0.3544028	7.45E-07	0.61800216	7.17E-21	0.61904982
FAM43B	turquoise	-0.4200637	2.64E-09	-0.0561027	0.44814878	0.45471715
FAM3C	turquoise	0.59908655	2.10E-19	-0.420989	2.42E-09	-0.8228361
FAM3A	turquoise	-0.6489228	1.71E-23	0.17227349	0.01903474	0.57037284
FAM229B	turquoise	-0.4940724	8.88E-13	0.55747169	1.69E-16	0.80701771
FAM220A	turquoise	0.57165665	1.92E-17	-0.4050853	1.07E-08	-0.9040605
FAM217B	turquoise	0.51344546	7.81E-14	-0.4976946	5.70E-13	-0.5774233
FAM216A	turquoise	0.62495378	1.96E-21	-0.6669451	3.62E-25	-0.7692336
FAM215A	turquoise	-0.4601081	4.43E-11	0.24524957	0.00076632	0.71638557
FAM214B	turquoise	-0.4987214	5.03E-13	0.1216416	0.09906132	0.69539227
FAM20C	turquoise	-0.449193	1.42E-10	0.119322	0.10571876	0.27594126
FAM20B	turquoise	0.58359888	2.83E-18	-0.6876072	3.10E-27	-0.3907571
FAM206A	turquoise	0.36599207	3.00E-07	-0.0979637	0.18463679	-0.7309353
FAM200B	turquoise	0.55419315	2.76E-16	-0.5105702	1.13E-13	-0.5483441
FAM19A2	turquoise	0.73433402	1.29E-32	-0.6037928	9.25E-20	-0.7193118
FAM196A	turquoise	0.62499689	1.94E-21	-0.3438607	1.65E-06	-0.2097579
FAM189A2	turquoise	-0.5184344	4.07E-14	0.62881434	9.37E-22	0.28546829
FAM188A	turquoise	0.65178395	9.44E-24	-0.6257958	1.67E-21	-0.5565689
FAM181B	turquoise	-0.5598487	1.18E-16	0.60383765	9.18E-20	0.38372942
FAM179B	turquoise	0.61187706	2.19E-20	-0.4287847	1.13E-09	-0.128021
FAM179A	turquoise	-0.2214645	0.0024493	0.40037275	1.64E-08	0.59587592
FAM175B	turquoise	0.67450526	6.63E-26	-0.4504892	1.24E-10	-0.4083065
FAM174A	turquoise	0.60988331	3.14E-20	-0.5523713	3.61E-16	-0.8207378
FAM171B	turquoise	0.28842389	6.85E-05	0.11490358	0.11936896	-0.2591367
FAM162A	turquoise	0.55392958	2.87E-16	-0.2634855	0.00029037	-0.0416157
FAM135B	turquoise	0.63345726	3.82E-22	-0.573147	1.52E-17	-0.6855371
FAM134B	turquoise	0.59354334	5.41E-19	-0.6546642	5.15E-24	-0.5668846
FAM133CP	turquoise	-0.3569438	6.12E-07	0.07512267	0.30949123	0.78341356
FAM133B	turquoise	-0.293752	4.94E-05	0.12020698	0.10313842	0.86982085
FAM131A	turquoise	0.55196008	3.84E-16	-0.6926723	9.06E-28	-0.2415012
FAM126B	turquoise	0.49783003	5.61E-13	-0.3631423	3.77E-07	-0.7105721
FAM122B	turquoise	0.43584042	5.62E-10	-0.2178631	0.00289151	-0.6990318
FAM110D	turquoise	-0.5620278	8.52E-17	0.52405431	1.93E-14	0.53003455
FAM107A	turquoise	-0.7389778	3.25E-33	0.71608732	2.19E-30	0.52250008
FAM102B	turquoise	0.72182216	4.55E-31	-0.6319217	5.15E-22	-0.7013498
FAM102A	turquoise	-0.3516793	9.17E-07	0.2345274	0.0013125	0.81663529
FAHD1	turquoise	0.62129204	3.89E-21	-0.579611	5.42E-18	-0.3410802
FAH	turquoise	-0.4380171	4.51E-10	0.48977337	1.49E-12	0.60182541
FABP3	turquoise	0.7735041	4.33E-38	-0.740757	1.91E-33	-0.5067211
F8	turquoise	0.48434845	2.84E-12	-0.34359	1.68E-06	-0.7355339
EXTL2	turquoise	0.61265931	1.90E-20	-0.479709	4.89E-12	-0.8367715
EXOSC9	turquoise	0.63158417	5.49E-22	-0.6428203	5.95E-23	-0.3112867

EXOSC3	turquoise	0.54082919	1.91E-15	-0.2395877	0.00102116	-0.5763052
EXOC6	turquoise	0.71447127	3.38E-30	-0.7590592	5.97E-36	-0.5598829
EXOC1	turquoise	0.5423606	1.54E-15	-0.3042455	2.55E-05	-0.6705433
EVI5L	turquoise	-0.5970155	3.00E-19	0.16681675	0.0232379	0.59016933
ETV6	turquoise	-0.4969813	6.22E-13	0.29560302	4.41E-05	0.7880337
ETS1	turquoise	-0.4439477	2.46E-10	0.77683717	1.32E-38	0.50140188
ESRRG	turquoise	0.55972196	1.21E-16	-0.5276232	1.19E-14	-0.4161193
ESD	turquoise	0.35980064	4.90E-07	0.0728576	0.32434692	-0.7737855
ESAM	turquoise	-0.6633722	7.95E-25	0.70932176	1.33E-29	0.38632595
ERO1A	turquoise	0.66358827	7.58E-25	-0.365224	3.19E-07	-0.4868831
ERLEC1	turquoise	0.68827495	2.64E-27	-0.6863347	4.20E-27	-0.7706672
ERICH3	turquoise	0.64176652	7.36E-23	-0.6815732	1.30E-26	-0.8205513
ERICH1	turquoise	0.56098286	9.98E-17	-0.059431	0.42163916	-0.6055797
ERH	turquoise	0.34225018	1.86E-06	-0.1071245	0.14668098	-0.4729951
ERCC1	turquoise	-0.3781224	1.12E-07	0.07945256	0.28235344	0.67617801
ERC2	turquoise	0.66798158	2.88E-25	-0.7164311	1.99E-30	-0.5477672
ERC1	turquoise	-0.4900684	1.44E-12	0.30465511	2.48E-05	0.64366436
EPT1	turquoise	0.68121608	1.41E-26	-0.6545065	5.33E-24	-0.4555043
EPS15	turquoise	0.6773187	3.48E-26	-0.5416937	1.69E-15	-0.6352293
EPOR	turquoise	-0.4938031	9.18E-13	0.70381556	5.54E-29	0.40946571
EPN2	turquoise	-0.6613015	1.25E-24	0.40806072	8.14E-09	0.62773536
EPM2AIP1	turquoise	0.54622382	8.85E-16	-0.7327581	2.04E-32	-0.6631727
EPM2A	turquoise	-0.3565367	6.32E-07	0.51076254	1.10E-13	0.6282118
EPHX4	turquoise	0.6950312	5.07E-28	-0.6303158	7.02E-22	-0.7145772
EPHX2	turquoise	-0.3343363	3.30E-06	0.53523392	4.20E-15	0.47012111
EPHA5	turquoise	0.63005583	7.38E-22	-0.775353	2.24E-38	-0.4457514
EPHA4	turquoise	0.68957754	1.92E-27	-0.5900228	9.79E-19	-0.7931212
EPHA3	turquoise	0.66857274	2.52E-25	-0.4187202	3.00E-09	-0.5800488
EPCAM	turquoise	0.60735064	4.94E-20	-0.4735137	9.96E-12	-0.5644154
EPB41L4B	turquoise	0.46795637	1.86E-11	-0.768796	2.24E-37	-0.3172362
EPB41L3	turquoise	0.6196771	5.26E-21	-0.5285552	1.05E-14	-0.8869429
EPB41L2	turquoise	-0.464708	2.67E-11	0.68467223	6.24E-27	0.21809544
ENY2	turquoise	0.36901214	2.36E-07	-0.0043208	0.95345315	-0.757527
ENTPD3	turquoise	0.73548888	9.17E-33	-0.6377893	1.63E-22	-0.7504978
ENTPD2	turquoise	-0.4468692	1.82E-10	0.2873903	7.30E-05	0.53892335
ENPP5	turquoise	0.49623469	6.82E-13	-0.3587903	5.30E-07	-0.5285824
ENPP4	turquoise	0.3990851	1.83E-08	0.00304588	0.96717812	-0.7962527
ENOPH1	turquoise	0.5292014	9.63E-15	-0.1864452	0.01105163	-0.8119339
ENHO	turquoise	-0.6495515	1.50E-23	0.54304495	1.40E-15	0.54177516
ENC1	turquoise	0.62749342	1.21E-21	-0.7183929	1.17E-30	-0.6319818
EMX2OS	turquoise	-0.6771803	3.59E-26	0.81855871	6.24E-46	0.49163855
EML1	turquoise	0.44867266	1.50E-10	-0.2996604	3.42E-05	-0.5830951
EMILIN2	turquoise	-0.3355111	3.03E-06	0.63616447	2.24E-22	0.56535582
EMC7	turquoise	0.33309243	3.61E-06	0.05258606	0.47715117	-0.7623706
EMC4	turquoise	0.44528355	2.14E-10	-0.1061412	0.15044619	-0.8441471
EMC1	turquoise	0.67085898	1.51E-25	-0.5961904	3.45E-19	-0.2936047

ELOVL4	turquoise	0.62042304	4.58E-21	-0.7969288	6.41E-42	-0.4791616
ELMOD1	turquoise	0.68097883	1.49E-26	-0.5985362	2.31E-19	-0.8179522
ELMO1	turquoise	0.4729391	1.06E-11	-0.6537328	6.27E-24	-0.5300334
ELK1	turquoise	-0.3074973	2.07E-05	0.14087143	0.05579665	0.6108478
ELAVL4	turquoise	0.52567137	1.55E-14	-0.7146165	3.25E-30	-0.7486867
ELAVL3	turquoise	-0.4386371	4.24E-10	0.18385854	0.01223855	0.87650629
ELAVL2	turquoise	0.65480294	5.00E-24	-0.5375934	3.02E-15	-0.7509456
EIF4G2	turquoise	0.28783902	7.10E-05	0.22729009	0.00186233	-0.7293862
EIF4G1	turquoise	-0.6686867	2.46E-25	0.4240248	1.80E-09	0.59292029
EIF4EBP3	turquoise	-0.4771997	6.53E-12	0.30055185	3.23E-05	0.70859256
EIF4E3	turquoise	0.56784604	3.49E-17	-0.3999404	1.70E-08	-0.808546
EIF4A2	turquoise	0.63711721	1.86E-22	-0.5269766	1.30E-14	-0.7473022
EIF3M	turquoise	0.57652367	8.90E-18	-0.2574214	0.00040412	-0.0656051
EIF3J	turquoise	0.37144573	1.94E-07	0.11056907	0.1340536	-0.6845311
EIF2S1	turquoise	0.6525701	8.01E-24	-0.4914837	1.21E-12	-0.0168468
EIF2D	turquoise	-0.4650985	2.56E-11	0.40560239	1.02E-08	0.70704107
EIF2B4	turquoise	-0.4930738	1.00E-12	0.40775418	8.37E-09	0.48787314
EIF2B3	turquoise	0.62118572	3.97E-21	-0.7232992	3.02E-31	-0.1353292
EIF1B	turquoise	0.5207987	2.98E-14	-0.2741764	0.00015901	-0.9155095
EIF1AX	turquoise	0.73500582	1.06E-32	-0.436021	5.52E-10	-0.3932539
EID2	turquoise	0.58010381	5.00E-18	-0.451811	1.08E-10	-0.9171573
EID1	turquoise	0.60601993	6.25E-20	-0.3474065	1.27E-06	-0.5938123
EHD2	turquoise	-0.5227749	2.29E-14	0.37447585	1.51E-07	0.80385248
EHBP1L1	turquoise	-0.5449767	1.06E-15	0.14918338	0.04269147	0.21123419
EGR3	turquoise	0.70998306	1.11E-29	-0.6656228	4.85E-25	-0.5808256
EGR1	turquoise	0.46465003	2.69E-11	-0.5587955	1.39E-16	-0.383729
EFR3B	turquoise	0.35773079	5.76E-07	-0.4782454	5.79E-12	-0.6321174
EFNB3	turquoise	0.55388528	2.89E-16	-0.7062834	2.93E-29	-0.1343799
EFNA1	turquoise	-0.4522536	1.03E-10	0.37266505	1.75E-07	0.54122633
EFHD1	turquoise	-0.6012865	1.43E-19	0.62936391	8.43E-22	0.1238945
EFCAB7	turquoise	0.61936866	5.57E-21	-0.2412403	0.00093973	-0.7666456
EFCAB14	turquoise	-0.5545014	2.64E-16	0.87890435	9.86E-61	0.39302506
EEF2K	turquoise	-0.3816004	8.35E-08	0.3907833	3.81E-08	0.69207688
EEF1E1	turquoise	0.63580651	2.41E-22	-0.355437	6.88E-07	-0.886582
EEF1D	turquoise	-0.6427853	5.99E-23	0.34835664	1.18E-06	0.37609926
EDN1	turquoise	-0.3796033	9.88E-08	0.11728451	0.11185246	0.49881699
ECHDC1	turquoise	0.64725763	2.41E-23	-0.0588008	0.42658742	-0.3395665
ECE2	turquoise	-0.1429444	0.05225148	0.20122946	0.00602131	0.67434913
ECD	turquoise	0.51078137	1.10E-13	-0.0989402	0.18027765	-0.7527171
EBNA1BP2	turquoise	0.62870211	9.58E-22	-0.5175406	4.58E-14	-0.8329013
EBI3	turquoise	-0.3906078	3.87E-08	0.60594469	6.33E-20	0.22378313
EARS2	turquoise	0.49341251	9.62E-13	-0.2781398	0.00012639	-0.6575411
EAPP	turquoise	0.71064477	9.36E-30	-0.2645178	0.00027426	-0.5609626
EAF1	turquoise	0.76528367	7.46E-37	-0.4905596	1.36E-12	-0.4938631
DYRK1A	turquoise	0.64542334	3.51E-23	-0.2496003	0.00061183	-0.804285
DYNLT3	turquoise	0.52613641	1.46E-14	-0.3251991	6.28E-06	-0.9183788

DYNLL1	turquoise	0.48857535	1.72E-12	-0.2715475	0.00018482	-0.8963558
DYNC2LI1	turquoise	0.60458371	8.05E-20	-0.2583661	0.00038403	-0.7957153
DYNC1I1	turquoise	0.66372568	7.36E-25	-0.6578137	2.64E-24	-0.856761
DUSP8	turquoise	-0.4570494	6.17E-11	0.32145333	8.14E-06	0.26514365
DUSP3	turquoise	-0.1997414	0.00641291	-0.0419492	0.57074578	0.65726256
DUSP28	turquoise	0.45580522	7.05E-11	-0.5119457	9.48E-14	-0.6411294
DUSP23	turquoise	-0.5236364	2.04E-14	0.47475681	8.65E-12	0.64473633
DUSP18	turquoise	-0.4932444	9.82E-13	0.45810649	5.50E-11	0.53454823
DUSP14	turquoise	0.36430081	3.44E-07	-0.5469595	7.96E-16	-0.4235685
DUSP11	turquoise	0.52988665	8.77E-15	-0.1424404	0.0530958	-0.6023876
DUSP1	turquoise	-0.361936	4.14E-07	0.37315718	1.68E-07	0.15574605
DUS4L	turquoise	0.60382223	9.20E-20	-0.2850749	8.39E-05	-0.463813
DTX1	turquoise	-0.3877641	4.94E-08	0.1836323	0.0123475	0.7503297
DTNA	turquoise	-0.3896213	4.21E-08	0.60472568	7.85E-20	0.35192496
DTD1	turquoise	0.38867635	4.57E-08	-0.6239779	2.35E-21	-0.4591127
DSTN	turquoise	0.64775755	2.17E-23	-0.5746294	1.20E-17	-0.7104575
DSE	turquoise	-0.3110667	1.63E-05	0.79747781	5.14E-42	0.07519156
DRG2	turquoise	-0.5247822	1.75E-14	0.08246195	0.26446352	0.64578409
DRG1	turquoise	0.50485538	2.34E-13	-0.4156443	4.02E-09	-0.8702904
DRD3	turquoise	-0.2712974	0.00018747	-0.1323967	0.07241475	0.6553484
DRAM1	turquoise	-0.4389538	4.10E-10	0.77534203	2.25E-38	0.35400682
DPYSL3	turquoise	-0.4788955	5.37E-12	0.64466048	4.10E-23	0.49949638
DPYSL2	turquoise	0.49218256	1.12E-12	-0.2131165	0.00358448	-0.8087486
DPY30	turquoise	0.4155253	4.06E-09	-0.0021826	0.97647757	-0.8159546
DPY19L1	turquoise	0.55947662	1.25E-16	-0.2993094	3.49E-05	-0.7705881
DPP9	turquoise	-0.641172	8.29E-23	0.24133397	0.00093529	0.40008141
DPP8	turquoise	0.49331827	9.73E-13	-0.2551063	0.00045752	-0.8866795
DPP10	turquoise	0.70499346	4.09E-29	-0.7432313	9.00E-34	-0.6388242
DPM1	turquoise	0.55823094	1.51E-16	-0.1626959	0.02692048	-0.8493256
DPF2	turquoise	-0.6924789	9.50E-28	0.49630262	6.77E-13	0.50437963
DOK6	turquoise	0.63479936	2.94E-22	-0.4992612	4.70E-13	-0.686862
DOHH	turquoise	-0.4038817	1.19E-08	-0.1116277	0.13034526	0.56411129
DOCK6	turquoise	-0.684174	7.02E-27	0.64709684	2.49E-23	0.5300935
DOCK3	turquoise	0.55572843	2.20E-16	-0.5591967	1.31E-16	-0.8862722
DOCK1	turquoise	-0.6589086	2.09E-24	0.74793701	2.11E-34	0.49241543
DNM3	turquoise	0.64087096	8.81E-23	-0.6788563	2.44E-26	-0.7881114
DNM1L	turquoise	0.64134696	8.00E-23	-0.568803	3.00E-17	-0.7963222
DNHD1	turquoise	-0.559906	1.17E-16	0.13740002	0.06217708	0.46767503
DNER	turquoise	0.39728575	2.15E-08	0.05382934	0.46678286	-0.7986534
DNASE1L1	turquoise	0.2312434	0.00154037	0.06830451	0.35557779	-0.6801711
DNALI1	turquoise	-0.3782548	1.11E-07	0.61887194	6.11E-21	0.7314185
DNAL4	turquoise	0.34662911	1.34E-06	-0.639991	1.05E-22	-0.1920394
DNAJC7	turquoise	0.55676049	1.88E-16	-0.5593944	1.27E-16	-0.3752415
DNAJC27	turquoise	0.59974322	1.87E-19	-0.7023944	7.97E-29	-0.4627975
DNAJC25	turquoise	0.73144318	2.99E-32	-0.4263043	1.45E-09	-0.4831857
DNAJC19	turquoise	0.69116841	1.31E-27	-0.3176105	1.06E-05	-0.7085818

DNAJC12	turquoise	0.62765029	1.17E-21	-0.4964721	6.63E-13	-0.793314
DNAJC10	turquoise	0.6025273	1.15E-19	-0.2881279	6.98E-05	-0.6308226
DNAJB9	turquoise	0.55796684	1.57E-16	-0.3092677	1.84E-05	-0.571945
DNAJB6	turquoise	0.49979797	4.40E-13	-0.1394145	0.0584054	-0.8174494
DNAJB2	turquoise	-0.6729767	9.39E-26	0.23772797	0.00112052	0.65174327
DNAJA4	turquoise	0.40308752	1.28E-08	-0.1263663	0.08652946	-0.6151218
DNAJA3	turquoise	0.59716851	2.92E-19	-0.5326089	6.04E-15	-0.2595675
DNAJA2	turquoise	0.61732055	8.13E-21	-0.5442584	1.17E-15	-0.6047011
DNAAF2	turquoise	0.61622638	9.94E-21	-0.127024	0.08489058	-0.73987
DMXL2	turquoise	0.63719308	1.83E-22	-0.4645267	2.73E-11	-0.8509978
DMWD	turquoise	-0.7018714	9.10E-29	0.27213242	0.00017876	0.27705865
DLX1	turquoise	0.74077901	1.89E-33	-0.7255401	1.61E-31	-0.5806735
DLK2	turquoise	0.52996362	8.68E-15	-0.7011994	1.08E-28	-0.0387569
DLGAP2	turquoise	0.49513306	7.80E-13	-0.7399271	2.45E-33	-0.3260192
DLG3	turquoise	0.65361656	6.43E-24	-0.8033738	4.61E-43	-0.2824143
DLG2	turquoise	0.37476102	1.48E-07	-0.6142617	1.42E-20	-0.3056285
DLD	turquoise	0.58947718	1.07E-18	-0.1571707	0.03263373	-0.7870414
DKK3	turquoise	0.58380894	2.74E-18	-0.3599357	4.85E-07	-0.698352
DIS3L2	turquoise	-0.441227	3.25E-10	-0.0097281	0.89544075	0.77176475
DIRAS2	turquoise	0.59189866	7.15E-19	-0.6069221	5.33E-20	-0.7632
DIP2C	turquoise	-0.3165938	1.13E-05	-0.0203603	0.78325374	0.51248725
DIEXF	turquoise	0.66356495	7.62E-25	-0.6015452	1.37E-19	-0.537936
DIAPH3	turquoise	-0.2381359	0.001098	0.53717689	3.20E-15	0.6207688
DIAPH2	turquoise	-0.2362556	0.00120539	0.08892423	0.22870998	0.73746833
DHX36	turquoise	0.61493839	1.26E-20	-0.3881687	4.78E-08	-0.7997296
DHX34	turquoise	-0.6215874	3.68E-21	0.24986857	0.00060332	0.77379888
DHX29	turquoise	0.52475255	1.76E-14	-0.2367284	0.00117752	-0.8482052
DHRS7	turquoise	0.30049977	3.24E-05	0.26853461	0.00021917	-0.6506738
DHRS4	turquoise	-0.5925695	6.38E-19	0.4191858	2.87E-09	0.63332148
DHRS3	turquoise	-0.6079322	4.45E-20	0.70271592	7.34E-29	0.5639466
DHCR24	turquoise	0.56483339	5.55E-17	-0.7774187	1.07E-38	-0.3549598
DGKI	turquoise	0.71075893	9.08E-30	-0.586214	1.84E-18	-0.6628272
DGKD	turquoise	-0.3857539	5.87E-08	0.09270962	0.209426	0.64112159
DGKB	turquoise	0.66764657	3.10E-25	-0.7454966	4.49E-34	-0.4053162
DGCR8	turquoise	-0.4207252	2.48E-09	0.06577672	0.37370217	0.5010687
DGCR6L	turquoise	-0.670545	1.62E-25	0.11600968	0.11582962	0.50454935
DGCR6	turquoise	-0.5383026	2.73E-15	0.1470363	0.04580092	0.67205511
DGCR11	turquoise	-0.514484	6.83E-14	0.26394558	0.00028308	0.75461541
DGAT1	turquoise	-0.4350131	6.11E-10	0.14041525	0.0566029	0.82933908
DFNA5	turquoise	0.58886882	1.19E-18	-0.3163504	1.15E-05	-0.4814577
DERL1	turquoise	0.68004342	1.85E-26	-0.2747639	0.00015372	-0.665523
DENND5A	turquoise	-0.4813337	4.05E-12	0.71822219	1.22E-30	0.47294068
DEF8	turquoise	-0.4584813	5.28E-11	0.19538689	0.00769254	0.469316
DEF6	turquoise	-0.3802767	9.33E-08	0.30987196	1.77E-05	0.76196261
DDX56	turquoise	-0.4480241	1.61E-10	0.06176743	0.40358741	0.28478033
DDX39A	turquoise	-0.5631118	7.22E-17	0.30948402	1.81E-05	0.66976692

DDX25	turquoise	0.68224948	1.11E-26	-0.6016772	1.34E-19	-0.7091351
DDX10	turquoise	0.67810119	2.91E-26	-0.8173935	1.06E-45	-0.5984418
DDX1	turquoise	0.62673455	1.40E-21	-0.4211188	2.39E-09	-0.8753922
DDR2	turquoise	-0.4994256	4.61E-13	0.54766071	7.19E-16	0.49226848
DDR1	turquoise	-0.6944909	5.79E-28	0.59766858	2.68E-19	0.18660915
DDIT4L	turquoise	-0.529765	8.92E-15	0.68575667	4.82E-27	0.62076822
DDIT4	turquoise	-0.6133848	1.67E-20	0.60858884	3.96E-20	0.61800029
DDAH2	turquoise	-0.6227106	2.99E-21	0.55111087	4.35E-16	0.62671315
DCXR	turquoise	-0.7241758	2.36E-31	0.36914222	2.33E-07	0.74736225
DCUN1D4	turquoise	0.58823912	1.32E-18	-0.5297774	8.90E-15	-0.1645677
DCTPP1	turquoise	0.42404548	1.80E-09	-0.4019747	1.42E-08	-0.6107017
DCTN3	turquoise	0.3216158	8.05E-06	-0.2668212	0.00024127	-0.8418123
DCTN2	turquoise	0.45900194	4.99E-11	-0.7948092	1.49E-41	-0.418204
DCP2	turquoise	0.69202174	1.06E-27	-0.4337039	6.96E-10	-0.5566407
DCLK1	turquoise	0.67499284	5.94E-26	-0.6519113	9.19E-24	-0.8144047
DCK	turquoise	0.53249453	6.13E-15	-0.2166614	0.00305442	-0.8729064
DCAKD	turquoise	-0.362365	4.01E-07	0.21826055	0.00283939	0.72533703
DCAF6	turquoise	0.67675247	3.97E-26	-0.5024085	3.18E-13	-0.8310359
DCAF4	turquoise	0.63570116	2.46E-22	-0.611402	2.39E-20	-0.7292611
DCAF16	turquoise	0.63470359	2.99E-22	-0.4479771	1.62E-10	-0.5862449
DCAF12	turquoise	-0.41926	2.85E-09	0.78617401	4.21E-40	0.58076057
DBN1	turquoise	-0.358457	5.44E-07	0.16156881	0.02801084	0.7856168
DBI	turquoise	-0.5647449	5.62E-17	0.76437995	1.01E-36	0.38683936
DAZAP2	turquoise	0.21602748	0.00314366	0.17794648	0.01538015	-0.6260956
DAXX	turquoise	-0.528324	1.09E-14	0.19195839	0.00885482	0.25349815
DAW1	turquoise	0.65297473	7.35E-24	-0.5415718	1.72E-15	-0.5892241
DAPK1	turquoise	0.58912817	1.14E-18	-0.5139754	7.29E-14	-0.7386576
DANCR	turquoise	-0.4511794	1.15E-10	0.65660882	3.41E-24	0.48171977
DAG1	turquoise	-0.6430904	5.63E-23	0.57570401	1.01E-17	0.45314222
DACH2	turquoise	0.68610657	4.43E-27	-0.6754934	5.29E-26	-0.7771681
DAB2	turquoise	-0.3468568	1.32E-06	0.39946846	1.77E-08	0.72893955
DAAM2	turquoise	-0.627506	1.20E-21	0.48910278	1.62E-12	0.40894161
CYTH4	turquoise	-0.4379324	4.55E-10	0.42092002	2.43E-09	0.26836789
CYTH2	turquoise	-0.6097648	3.21E-20	0.3711902	1.98E-07	0.52569226
CYTH1	turquoise	-0.4297541	1.03E-09	0.11954	0.10507847	0.4538777
CYP4X1	turquoise	0.66566519	4.81E-25	-0.7179053	1.33E-30	-0.6206084
CYP4A11	turquoise	0.37204993	1.84E-07	-0.7337345	1.53E-32	-0.2088297
CYP2E1	turquoise	0.5344078	4.71E-15	-0.6690935	2.25E-25	-0.3822416
CYP2C8	turquoise	0.34665812	1.34E-06	-0.6426966	6.10E-23	-0.5897441
CYP27A1	turquoise	-0.6122364	2.06E-20	0.46535043	2.49E-11	0.43505165
CYP26B1	turquoise	0.60676025	5.48E-20	-0.5925388	6.42E-19	-0.7426932
CYCS	turquoise	0.58238803	3.45E-18	-0.2352833	0.00126462	-0.5144967
CYB5D2	turquoise	0.32808147	5.14E-06	-0.2910609	5.83E-05	-0.7110635
CXXC1	turquoise	-0.6260565	1.59E-21	0.32711851	5.50E-06	0.73784168
CXorf57	turquoise	0.52115936	2.84E-14	-0.1240953	0.09238487	-0.6391693
CXorf40B	turquoise	0.61569005	1.10E-20	-0.2209906	0.00250374	-0.6081873

CXorf40A	turquoise	0.53424981	4.81E-15	-0.2820972	0.00010015	-0.6357425
CXCL16	turquoise	-0.4391849	4.01E-10	0.73518877	1.00E-32	0.55398432
CXADR	turquoise	0.60119476	1.46E-19	-0.4963297	6.74E-13	-0.7525618
CUX2	turquoise	0.47785321	6.06E-12	-0.490482	1.37E-12	-0.3433627
CUX1	turquoise	-0.4766967	6.92E-12	0.53440173	4.71E-15	0.55221281
CUL5	turquoise	0.59716913	2.92E-19	-0.1548381	0.03533827	-0.5157965
CUL2	turquoise	0.66488426	5.71E-25	-0.446732	1.84E-10	-0.7820606
CTXN2	turquoise	0.40824406	8.00E-09	-0.6164634	9.52E-21	-0.5499836
CTSH	turquoise	-0.6036891	9.42E-20	0.61765515	7.64E-21	0.41596045
CTPS1	turquoise	0.53132433	7.20E-15	-0.3359702	2.93E-06	-0.6471942
CTNNA3	turquoise	-0.3644913	3.39E-07	0.19730476	0.00710334	0.5527929
CTIF	turquoise	-0.5158117	5.74E-14	-0.0512545	0.48839325	0.18247185
CTH	turquoise	0.39896611	1.85E-08	-0.0610772	0.4088716	-0.3094913
CTDSPL	turquoise	-0.5982427	2.43E-19	0.58059977	4.62E-18	0.80024443
CTDSP2	turquoise	-0.6020327	1.26E-19	0.79820403	3.84E-42	0.71514418
CTDSP1	turquoise	-0.6261764	1.55E-21	0.54202474	1.61E-15	0.66205877
CSTF3	turquoise	0.43189972	8.34E-10	-0.4316984	8.50E-10	-0.2370354
CSTF2T	turquoise	0.56248117	7.95E-17	-0.0486756	0.51056244	-0.5861487
CSRP2	turquoise	-0.4689321	1.67E-11	0.7330327	1.88E-32	0.38137881
CSRP1	turquoise	-0.7186853	1.08E-30	0.52684891	1.33E-14	0.28815903
CSRNP2	turquoise	0.45143898	1.12E-10	-0.2871947	7.38E-05	-0.898972
CSNK1G3	turquoise	0.54930045	5.66E-16	-0.1906046	0.00935502	-0.8394427
CSNK1A1	turquoise	0.34069353	2.08E-06	0.09920305	0.17911713	-0.6871422
CSMD3	turquoise	0.64782692	2.14E-23	-0.5506647	4.64E-16	-0.5497688
CSK	turquoise	-0.672006	1.17E-25	0.44076911	3.41E-10	0.39809969
CSF3R	turquoise	-0.3686762	2.42E-07	0.64499719	3.83E-23	0.46868244
CSF1R	turquoise	-0.48706	2.06E-12	0.69299353	8.37E-28	0.53391925
CSE1L	turquoise	0.59856686	2.30E-19	-0.533307	5.48E-15	-0.6300513
CSDC2	turquoise	-0.2081916	0.00445865	-0.005093	0.94514644	0.60163114
CSAD	turquoise	0.31868581	9.82E-06	-0.0133437	0.8569391	-0.7366858
CRYZL1	turquoise	0.49320948	9.86E-13	-0.1551828	0.03492709	-0.5803798
CRYM	turquoise	0.68608275	4.46E-27	-0.8114325	1.49E-44	-0.6958628
CRYAB	turquoise	-0.5675902	3.63E-17	0.53410709	4.91E-15	0.10113455
CRTC3	turquoise	-0.6726944	1.00E-25	0.38530198	6.10E-08	0.73973377
CRTAP	turquoise	-0.3611945	4.39E-07	0.30612245	2.26E-05	0.77666644
CROCC	turquoise	-0.4283223	1.19E-09	-0.0344375	0.6416706	0.74401648
CRK	turquoise	-0.4212512	2.36E-09	0.58741878	1.51E-18	0.25667345
CRISPLD2	turquoise	-0.4349202	6.17E-10	0.56187377	8.72E-17	0.32551701
CRIP1	turquoise	-0.4903026	1.40E-12	0.59807159	2.50E-19	0.53514746
CRHBP	turquoise	0.57715135	8.05E-18	-0.5254969	1.59E-14	-0.6942035
CRH	turquoise	0.657982	2.55E-24	-0.607136	5.13E-20	-0.4204405
CREG2	turquoise	0.60552453	6.82E-20	-0.729559	5.14E-32	-0.6533118
CRBN	turquoise	0.5438786	1.24E-15	-0.3331749	3.59E-06	-0.6872812
CRACR2B	turquoise	-0.5540755	2.81E-16	0.34166036	1.94E-06	0.56493721
CPSF1	turquoise	-0.5148128	6.54E-14	0.30878038	1.90E-05	0.66374491
CPNE8	turquoise	0.48651703	2.20E-12	-0.0986789	0.18143664	-0.2447752

CPNE4	turquoise	0.62120054	3.96E-21	-0.6257216	1.69E-21	-0.695852
CPM	turquoise	-0.533792	5.13E-15	0.27828059	0.00012536	0.66918757
CPEB3	turquoise	-0.3427783	1.79E-06	0.23092791	0.00156406	0.62253037
CPEB2	turquoise	0.3241082	6.78E-06	-0.0854237	0.24762901	-0.5527253
CPE	turquoise	0.2166312	0.00305862	0.27451374	0.00015596	-0.5985993
COX7B	turquoise	0.66271829	9.16E-25	-0.186554	0.01100401	-0.5639437
COX7A2L	turquoise	0.65634024	3.61E-24	-0.7045053	4.64E-29	-0.6623544
COX7A2	turquoise	0.47584272	7.64E-12	-0.034673	0.63939252	-0.7337384
COX6C	turquoise	0.5589674	1.35E-16	-0.3119877	1.54E-05	-0.779792
COX5A	turquoise	0.59176338	7.31E-19	-0.5246559	1.78E-14	-0.802094
COX10	turquoise	0.42230202	2.13E-09	-0.4397016	3.80E-10	-0.285989
CORT	turquoise	0.59411619	4.91E-19	-0.6200353	4.92E-21	-0.6238404
COQ10B	turquoise	0.66085913	1.37E-24	-0.3851146	6.20E-08	-0.5529467
COPS8	turquoise	0.65756015	2.79E-24	-0.403716	1.21E-08	-0.6847062
COPS5	turquoise	0.54379427	1.25E-15	-0.1291609	0.07973625	-0.7833808
COPS4	turquoise	0.65620717	3.72E-24	-0.3385468	2.44E-06	-0.8340047
COPS3	turquoise	0.67127782	1.38E-25	-0.5981845	2.45E-19	-0.6844653
COPS2	turquoise	0.69175674	1.13E-27	-0.142529	0.05294656	-0.4823151
COPG2IT1	turquoise	0.51985172	3.38E-14	-0.7351658	1.01E-32	-0.4799308
COPG2	turquoise	0.32916298	4.76E-06	-0.2574571	0.00040334	-0.0990926
COPG1	turquoise	0.40961591	7.05E-09	-0.6265743	1.44E-21	-0.7308873
COPA	turquoise	0.40924488	7.30E-09	-0.3023773	2.87E-05	-0.7591862
COMTD1	turquoise	-0.4168261	3.59E-09	-0.0625635	0.39754289	0.63055419
COMMD3	turquoise	0.38756542	5.03E-08	0.04515311	0.54166477	-0.6292201
COMMD10	turquoise	0.5582306	1.51E-16	-0.0550838	0.45644779	-0.6508416
COLGALT2	turquoise	-0.3238432	6.90E-06	0.14565366	0.04790177	0.33925597
COLGALT1	turquoise	-0.6605576	1.46E-24	0.44532893	2.13E-10	0.65995404
COLEC12	turquoise	-0.255215	0.00045487	0.7293586	5.44E-32	-0.0401169
COL9A1	turquoise	-0.3575371	5.85E-07	0.61207739	2.12E-20	0.28594084
COL6A1	turquoise	-0.6419084	7.15E-23	0.25318782	0.00050665	0.45092432
COL5A2	turquoise	0.55522568	2.37E-16	-0.5991983	2.06E-19	-0.3654227
COL24A1	turquoise	0.61574801	1.09E-20	-0.5381121	2.81E-15	-0.6372234
COL20A1	turquoise	-0.5664531	4.32E-17	0.27961016	0.00011597	0.50876373
COL1A2	turquoise	-0.4207345	2.48E-09	0.70978776	1.17E-29	0.09116323
COL18A1	turquoise	-0.5132319	8.03E-14	0.45730625	6.00E-11	0.75407218
COL16A1	turquoise	-0.3326416	3.72E-06	0.16682597	0.02323018	0.78774686
COCH	turquoise	0.63255814	4.55E-22	-0.3769949	1.23E-07	-0.5537277
COBLL1	turquoise	-0.3074838	2.07E-05	0.60710782	5.15E-20	0.52951123
COBL	turquoise	-0.3459471	1.41E-06	0.4825195	3.52E-12	0.1455471
COA1	turquoise	0.42638553	1.43E-09	-0.2247091	0.00210444	-0.8000986
CNTN6	turquoise	0.79854456	3.34E-42	-0.5890696	1.15E-18	-0.5175737
CNTN3	turquoise	0.64236815	6.52E-23	-0.5004965	4.03E-13	-0.7426708
CNTN1	turquoise	0.41276237	5.27E-09	-0.1278226	0.08293381	-0.8056749
CNRIP1	turquoise	0.67790907	3.04E-26	-0.6735874	8.17E-26	-0.6293102
CNPY4	turquoise	-0.4310556	9.06E-10	0.53237122	6.24E-15	0.69825105
CNOT9	turquoise	0.64261598	6.20E-23	-0.3392815	2.31E-06	-0.76239

CNOT7	turquoise	0.60665644	5.58E-20	-0.2017514	0.00588911	-0.8046489
CNNM3	turquoise	-0.6063137	5.93E-20	0.38717789	5.20E-08	0.87219006
CNN3	turquoise	-0.356577	6.30E-07	0.68539163	5.26E-27	0.40597526
CNKSR3	turquoise	-0.5114978	1.00E-13	0.68932806	2.04E-27	0.48729804
CNIH3	turquoise	0.70193368	8.96E-29	-0.6646837	5.96E-25	-0.6150209
CNGB1	turquoise	-0.2242356	0.00215185	0.03202003	0.66524832	0.66357082
CNBP	turquoise	0.18627347	0.01112717	0.20624801	0.00485334	-0.7473939
CMTM3	turquoise	-0.5513712	4.18E-16	0.72927763	5.57E-32	0.62281071
CMSS1	turquoise	0.7560006	1.62E-35	-0.5889556	1.17E-18	-0.5895033
CMKLR1	turquoise	-0.3596533	4.96E-07	0.61003589	3.06E-20	0.47117519
CMC1	turquoise	0.47849001	5.63E-12	-0.2687506	0.00021652	-0.8417998
CMAS	turquoise	0.68841031	2.55E-27	-0.5663921	4.36E-17	-0.8042513
CLUL1	turquoise	0.65756697	2.78E-24	-0.4664283	2.21E-11	-0.5803283
CLTC	turquoise	0.77266621	5.82E-38	-0.3726831	1.75E-07	-0.6203798
CLTA	turquoise	0.41979172	2.71E-09	-0.6697833	1.93E-25	-0.6286609
CLRN1	turquoise	-0.5178998	4.37E-14	0.29138283	5.72E-05	0.77500866
CLOCK	turquoise	0.71602243	2.23E-30	-0.4858568	2.38E-12	-0.5705361
CLN8	turquoise	-0.3437568	1.66E-06	0.36163145	4.25E-07	0.53716808
CLIP4	turquoise	0.3385678	2.43E-06	0.06346199	0.39078679	-0.7532889
CLIP2	turquoise	-0.683348	8.53E-27	0.20359778	0.00544201	0.39043098
CLIP1	turquoise	0.5070505	1.77E-13	-0.0963967	0.19179357	-0.4772272
CLINT1	turquoise	0.40807994	8.12E-09	0.05040399	0.49564692	-0.7772626
CLIC1	turquoise	-0.5088936	1.40E-13	0.65598852	3.89E-24	0.28896892
CLEC2D	turquoise	0.31067432	1.68E-05	0.01861649	0.80141422	-0.3784495
CLEC2B	turquoise	-0.4591699	4.90E-11	0.67145299	1.32E-25	0.53012778
CLEC14A	turquoise	-0.3710517	2.00E-07	0.70211094	8.56E-29	0.45087034
CLDND2	turquoise	-0.288838	6.68E-05	0.06143467	0.40612995	0.73718667
CLDN5	turquoise	-0.6761155	4.59E-26	0.53083005	7.71E-15	0.70479327
CLDN15	turquoise	-0.6003863	1.68E-19	0.50549043	2.16E-13	0.58126169
CLCC1	turquoise	-0.4408275	3.39E-10	0.72880277	6.38E-32	0.5052565
CLASRP	turquoise	-0.3989383	1.86E-08	0.04671645	0.52774597	0.45475526
CLASP2	turquoise	0.38176189	8.24E-08	-0.063309	0.39193215	-0.857587
CKAP5	turquoise	0.41223173	5.53E-09	-0.3416283	1.94E-06	-0.7262348
CITED2	turquoise	0.65525267	4.55E-24	-0.6001949	1.73E-19	-0.7968353
CISD1	turquoise	0.61601077	1.03E-20	-0.8184994	6.41E-46	-0.5957266
CIRBP	turquoise	0.48202811	3.73E-12	-0.6514006	1.02E-23	-0.4233586
CIDECP	turquoise	0.26435601	0.00027673	0.03937081	0.59467221	-0.7812457
CIC	turquoise	-0.5624388	8.00E-17	0.12426002	0.09194975	0.1177346
CIB2	turquoise	-0.4872483	2.02E-12	0.25998794	0.00035169	0.75342878
CHSY3	turquoise	0.65878422	2.14E-24	-0.5891055	1.14E-18	-0.4454203
CHSY1	turquoise	-0.3093371	1.83E-05	0.60853844	3.99E-20	0.46004409
CHST6	turquoise	-0.5262319	1.44E-14	0.45894735	5.02E-11	0.72841673
CHST3	turquoise	-0.7872073	2.85E-40	0.46274445	3.32E-11	0.46002353
CHRNB1	turquoise	-0.4405022	3.50E-10	0.69207355	1.05E-27	0.55941066
CHRM3	turquoise	0.62146766	3.77E-21	-0.6811447	1.43E-26	-0.5772493
CHP1	turquoise	0.51661868	5.17E-14	-0.419843	2.70E-09	-0.8068214

CHN1	turquoise	0.58551639	2.07E-18	-0.6921366	1.03E-27	-0.7380937
CHMP5	turquoise	0.6573808	2.90E-24	-0.288797	6.70E-05	-0.6665429
CHMP4B	turquoise	0.3975934	2.10E-08	-0.5944422	4.65E-19	-0.5058146
CHMP2B	turquoise	0.54986762	5.21E-16	-0.0636074	0.3896999	-0.7212279
CHMP1B	turquoise	0.42170394	2.26E-09	-0.1313141	0.07480118	-0.8275466
CHMP1A	turquoise	-0.64148	7.79E-23	0.12918474	0.07968027	0.49496371
CHM	turquoise	0.72702975	1.06E-31	-0.6408511	8.84E-23	-0.4401072
CHL1	turquoise	0.61099477	2.57E-20	-0.414684	4.40E-09	-0.8229532
CHKB	turquoise	-0.4868614	2.11E-12	0.04661496	0.52864404	0.55711952
CHKA	turquoise	-0.4623439	3.47E-11	0.28040547	0.00011067	0.50591205
CHGB	turquoise	0.68806898	2.77E-27	-0.690539	1.52E-27	-0.8256797
CHGA	turquoise	0.32785351	5.22E-06	-0.5355995	3.99E-15	-0.4574122
CHDH	turquoise	-0.5582659	1.50E-16	0.68415309	7.05E-27	0.63477186
CHD8	turquoise	-0.5560525	2.09E-16	0.14389696	0.05068624	0.75313451
CHD7	turquoise	-0.5905359	8.99E-19	0.67772146	3.17E-26	0.75751421
CHCHD7	turquoise	0.47983152	4.82E-12	-0.4986278	5.08E-13	-0.3533534
CHCHD6	turquoise	0.50709852	1.76E-13	-0.7404104	2.12E-33	-0.6769135
CHCHD2	turquoise	0.50305435	2.93E-13	-0.2762095	0.00014141	-0.6107686
CHADL	turquoise	-0.3981748	1.99E-08	0.07751889	0.29426853	0.51098718
CGNL1	turquoise	-0.525804	1.53E-14	0.80397402	3.59E-43	0.68967931
CGN	turquoise	-0.355132	7.04E-07	0.28781682	7.11E-05	0.68943298
CGGBP1	turquoise	0.44290988	2.74E-10	0.07539846	0.30771339	-0.5965214
CFI	turquoise	-0.3371584	2.69E-06	0.62282291	2.92E-21	0.36750227
CFH	turquoise	-0.4267486	1.38E-09	0.54242371	1.53E-15	0.59689761
CFDP1	turquoise	0.41402823	4.68E-09	-0.3012361	3.09E-05	-0.2928497
CFB	turquoise	-0.548584	6.29E-16	0.46020074	4.38E-11	0.4375913
CFAP36	turquoise	0.63805272	1.55E-22	-0.32907	4.80E-06	-0.8274301
CFAP20	turquoise	0.57801647	7.00E-18	-0.5222989	2.44E-14	-0.679838
CETN2	turquoise	0.46569577	2.40E-11	-0.2514029	0.00055669	-0.3117702
CERS6	turquoise	0.57046983	2.32E-17	-0.7855487	5.34E-40	-0.4719131
CERS2	turquoise	-0.5674413	3.71E-17	0.77847525	7.29E-39	0.44240342
CERS1	turquoise	-0.6754772	5.31E-26	0.51165778	9.84E-14	0.70285003
CEPT1	turquoise	0.32001776	8.97E-06	0.26081834	0.00033613	-0.4147666
CEP68	turquoise	0.43333674	7.22E-10	-0.3067322	2.17E-05	-0.7194024
CEP41	turquoise	0.70188923	9.06E-29	-0.6219569	3.44E-21	-0.7927172
CEP295	turquoise	-0.3554519	6.87E-07	0.1814312	0.01345278	0.90286894
CEP104	turquoise	-0.4937887	9.19E-13	0.32444072	6.62E-06	0.6180212
CENPF	turquoise	0.59931911	2.02E-19	-0.5759907	9.69E-18	-0.3292348
CELF2	turquoise	0.62678548	1.38E-21	-0.53036	8.22E-15	-0.7689404
CEL	turquoise	-0.4491422	1.43E-10	0.26893098	0.00021434	0.23268599
CECR6	turquoise	0.47444169	8.96E-12	-0.8185051	6.40E-46	-0.4313059
CECR1	turquoise	-0.5002892	4.14E-13	0.42054379	2.52E-09	0.26897351
CEBPD	turquoise	-0.6274374	1.22E-21	0.58695833	1.63E-18	0.38071492
CEBPB	turquoise	-0.4646524	2.69E-11	0.59857492	2.29E-19	0.40448119
CDYL2	turquoise	0.59085175	8.52E-19	-0.46813	1.83E-11	-0.5395141
CDS2	turquoise	0.55237337	3.61E-16	-0.4371324	4.93E-10	-0.4490273

CDR2L	turquoise	-0.3781033	1.12E-07	0.26500471	0.00026696	0.75482732
CDR2	turquoise	0.61907103	5.88E-21	-0.6103856	2.87E-20	-0.2390738
CDO1	turquoise	0.65309041	7.18E-24	-0.2788415	0.00012131	-0.6471546
CDKN1A	turquoise	-0.5340838	4.92E-15	0.49129088	1.24E-12	0.41646563
CDK6	turquoise	-0.3284152	5.02E-06	0.41691839	3.56E-09	0.56348542
CDK5RAP2	turquoise	-0.4719106	1.19E-11	0.38086514	8.88E-08	0.76861281
CDK5R1	turquoise	0.56495406	5.45E-17	-0.7542114	2.89E-35	-0.4971966
CDK5	turquoise	0.38135444	8.53E-08	-0.6610863	1.31E-24	-0.6524683
CDK4	turquoise	-0.6454018	3.52E-23	0.52124817	2.81E-14	0.44939438
CDK20	turquoise	0.52410808	1.92E-14	-0.7748392	2.69E-38	-0.3623421
CDK2	turquoise	-0.5431896	1.37E-15	0.69433056	6.03E-28	0.44534399
CDK19	turquoise	-0.4222383	2.14E-09	0.22982306	0.00164968	0.83475732
CDK14	turquoise	0.61782403	7.41E-21	-0.6552796	4.52E-24	-0.7931256
CDIP1	turquoise	-0.6041656	8.66E-20	0.59496902	4.25E-19	0.67025289
CDH8	turquoise	0.65158958	9.83E-24	-0.757716	9.28E-36	-0.5166395
CDH23	turquoise	-0.4620658	3.58E-11	0.21961989	0.00266748	0.58298375
CDH22	turquoise	0.53624569	3.64E-15	-0.7612777	2.87E-36	-0.2560529
CDH20	turquoise	-0.5019002	3.39E-13	0.41822069	3.15E-09	0.74678751
CDH13	turquoise	0.74729536	2.57E-34	-0.6754449	5.35E-26	-0.7387114
CDH12	turquoise	0.69954503	1.64E-28	-0.6883984	2.56E-27	-0.618075
CDH10	turquoise	0.58737458	1.52E-18	-0.407183	8.82E-09	-0.610006
CDCA5	turquoise	0.61379574	1.55E-20	-0.8006925	1.39E-42	-0.3795536
CDC7	turquoise	0.61498313	1.25E-20	-0.526357	1.42E-14	-0.3950851
CDC42SE2	turquoise	0.78244087	1.70E-39	-0.5815408	3.96E-18	-0.4874038
CDC40	turquoise	0.68038987	1.71E-26	-0.5413904	1.77E-15	-0.7592673
CDC25B	turquoise	-0.5315907	6.94E-15	0.0924763	0.21057973	0.3940381
CDC123	turquoise	0.50680211	1.83E-13	-0.2790712	0.00011969	-0.7768345
CD99	turquoise	-0.5096719	1.27E-13	0.57597977	9.70E-18	0.16534525
CD84	turquoise	-0.2953056	4.49E-05	0.12181278	0.09858349	0.72419986
CD83	turquoise	0.53035775	8.22E-15	-0.6394172	1.18E-22	-0.6597668
CD82	turquoise	-0.6185067	6.53E-21	0.40054312	1.61E-08	0.80123015
CD79B	turquoise	-0.5624291	8.01E-17	0.37827556	1.10E-07	0.73497987
CD7	turquoise	-0.3688679	2.39E-07	0.37916601	1.02E-07	0.7123132
CD59	turquoise	-0.229165	0.00170269	0.5956365	3.79E-19	0.17326105
CD47	turquoise	0.67838094	2.72E-26	-0.657834	2.63E-24	-0.6494873
CD44	turquoise	-0.2340007	0.00134684	0.40319208	1.27E-08	0.12432887
CD34	turquoise	-0.3871233	5.22E-08	0.64119122	8.26E-23	0.41713241
CD2BP2	turquoise	0.35847836	5.43E-07	-0.4981734	5.38E-13	-0.2804486
CD24	turquoise	0.56548495	5.02E-17	-0.4950941	7.84E-13	-0.404103
CD151	turquoise	-0.6012815	1.43E-19	0.55941404	1.27E-16	0.86990637
CD14	turquoise	-0.3384998	2.44E-06	0.35994894	4.84E-07	0.17700451
CCT6P1	turquoise	0.58277629	3.24E-18	-0.2215169	0.00244334	-0.7782778
CCT2	turquoise	0.4623638	3.46E-11	-0.127081	0.08474968	-0.742243
CCSER2	turquoise	0.5764396	9.02E-18	-0.2269872	0.00188937	-0.8359212
CCS	turquoise	-0.3347651	3.20E-06	-0.0657169	0.37413817	0.7699382
CCPG1	turquoise	0.50287496	3.00E-13	-0.2838373	9.03E-05	-0.3321094

CCNY	turquoise	-0.3146875	1.29E-05	0.02511175	0.73438733	0.69029089
CCNT2	turquoise	0.56693974	4.01E-17	-0.1584098	0.03127003	-0.7481712
CCNH	turquoise	0.73104715	3.35E-32	-0.4213226	2.34E-09	-0.5759959
CCND2	turquoise	0.50667547	1.86E-13	-0.2727358	0.0001727	-0.5187365
CCND1	turquoise	-0.4646702	2.68E-11	0.34566933	1.44E-06	0.66593712
CCNC	turquoise	0.56936241	2.75E-17	-0.224207	0.00215475	-0.8349435
CCM2L	turquoise	-0.5066976	1.85E-13	0.42412522	1.79E-09	0.65840737
CCKBR	turquoise	0.53898543	2.48E-15	-0.7633063	1.45E-36	-0.7046292
CCK	turquoise	0.58244791	3.42E-18	-0.7343167	1.29E-32	-0.5709431
CCDC97	turquoise	-0.4710624	1.31E-11	0.53946	2.32E-15	0.24832242
CCDC85C	turquoise	-0.3820311	8.05E-08	0.41173961	5.79E-09	0.80039913
CCDC85A	turquoise	0.66112126	1.30E-24	-0.6468409	2.62E-23	-0.4305713
CCDC6	turquoise	0.55574973	2.19E-16	-0.6241427	2.28E-21	-0.4369195
CCDC34	turquoise	-0.2146779	0.00334155	-0.1354728	0.06597025	0.61058343
CCDC190	turquoise	-0.2515138	0.00055346	0.28603797	7.92E-05	0.68656578
CCDC110	turquoise	0.68908465	2.17E-27	-0.5120665	9.34E-14	-0.6883043
CCDC102A	turquoise	-0.4238768	1.83E-09	0.69742917	2.79E-28	0.71386627
CCAR2	turquoise	0.3666299	2.86E-07	-0.5934153	5.53E-19	-0.4635179
CBY1	turquoise	-0.5708019	2.20E-17	0.26430419	0.00027752	0.65563606
CBX7	turquoise	0.48570658	2.42E-12	-0.4353297	5.92E-10	-0.7213961
CBX1	turquoise	0.2489476	0.00063301	-0.2213188	0.00246592	-0.4629448
CBS	turquoise	-0.5047013	2.39E-13	0.63679813	1.98E-22	0.63060348
CBLN4	turquoise	0.69859656	2.08E-28	-0.6470685	2.50E-23	-0.7840121
CBLN2	turquoise	0.63539775	2.61E-22	-0.6562012	3.72E-24	-0.6669409
CAV1	turquoise	-0.3381296	2.51E-06	0.72285511	3.41E-31	0.15237258
CASZ1	turquoise	-0.4441814	2.40E-10	0.60902996	3.66E-20	0.68573962
CASD1	turquoise	0.62001062	4.94E-21	-0.329065	4.80E-06	-0.7277359
CASC3	turquoise	-0.6910874	1.33E-27	0.63259852	4.51E-22	0.27398696
CASC15	turquoise	0.48813332	1.82E-12	-0.7794537	5.11E-39	-0.2738403
CASC1	turquoise	0.69934991	1.72E-28	-0.5960658	3.53E-19	-0.4471667
CARTPT	turquoise	0.50787479	1.60E-13	-0.5305992	7.96E-15	-0.4489618
CARM1	turquoise	-0.6838168	7.64E-27	0.12264028	0.09629924	0.62403083
CARHSP1	turquoise	-0.4856572	2.44E-12	0.45414011	8.43E-11	0.68560122
CARD9	turquoise	-0.3739776	1.57E-07	0.41853803	3.06E-09	0.59532511
CARD8	turquoise	-0.2534266	0.00050028	0.59725409	2.88E-19	0.55666922
CAPRIN2	turquoise	0.52595519	1.50E-14	-0.4770005	6.69E-12	-0.7428293
CAPRIN1	turquoise	0.62754789	1.19E-21	-0.6805802	1.63E-26	-0.5290681
CAPG	turquoise	-0.4353639	5.90E-10	0.37164874	1.90E-07	0.5193762
CAP2	turquoise	0.67098582	1.47E-25	-0.646055	3.08E-23	-0.7128765
CANX	turquoise	0.60089073	1.54E-19	-0.4026766	1.33E-08	-0.421855
CAND1	turquoise	0.61271133	1.89E-20	-0.2368778	0.00116883	-0.7856209
CAMTA1	turquoise	0.60673621	5.50E-20	-0.7666861	4.63E-37	-0.5027978
CAMSAP1	turquoise	0.68088365	1.52E-26	-0.501858	3.41E-13	-0.5192267
CAMLG	turquoise	0.53196334	6.60E-15	-0.5205528	3.08E-14	-0.2576049
CAMKV	turquoise	0.48095918	4.23E-12	-0.7898392	1.04E-40	-0.2955845
CAMK4	turquoise	0.67166889	1.26E-25	-0.5546342	2.59E-16	-0.7378651

CAMK2G	turquoise	0.287487	7.25E-05	-0.004243	0.95428997	-0.6213796
CAMK2D	turquoise	0.55069918	4.62E-16	-0.6508686	1.14E-23	-0.3444744
CAMK2B	turquoise	-0.2725908	0.00017414	-0.2751134	0.00015066	0.40098329
CAMK1D	turquoise	0.56912415	2.86E-17	-0.5727008	1.63E-17	-0.8286484
CALY	turquoise	0.36017672	4.76E-07	-0.8337039	4.50E-49	-0.2404957
CALML4	turquoise	-0.4587286	5.14E-11	0.6414742	7.80E-23	0.49796784
CALM3	turquoise	0.54053273	2.00E-15	-0.7414459	1.55E-33	-0.7152275
CALM2	turquoise	0.57932634	5.67E-18	-0.4082795	7.98E-09	-0.8282771
CALM1	turquoise	0.65528926	4.52E-24	-0.6208469	4.23E-21	-0.785614
CALD1	turquoise	-0.5591066	1.33E-16	0.39413209	2.84E-08	0.88619282
CALB1	turquoise	0.81017177	2.58E-44	-0.5214219	2.74E-14	-0.5598131
CADPS2	turquoise	0.59539956	3.95E-19	-0.5553906	2.31E-16	-0.5920764
CADPS	turquoise	0.64445922	4.27E-23	-0.6666471	3.87E-25	-0.7901329
CADM2	turquoise	0.41549173	4.08E-09	-0.0735747	0.31959498	-0.7431956
CADM1	turquoise	-0.2643489	0.00027684	0.39774401	2.07E-08	0.38001422
CACNB4	turquoise	0.5062674	1.96E-13	-0.5291507	9.70E-15	-0.3831257
CACNB2	turquoise	0.61118265	2.49E-20	-0.8166012	1.51E-45	-0.5162084
CACNB1	turquoise	0.38922927	4.36E-08	-0.8331517	5.94E-49	-0.2219897
CACNA2D3	turquoise	0.68145179	1.33E-26	-0.7120868	6.39E-30	-0.5404844
CACNA1I	turquoise	-0.3238818	6.88E-06	0.03593323	0.62725856	0.67412711
CACNA1G	turquoise	0.51391206	7.35E-14	-0.4488604	1.47E-10	-0.3289558
CACFD1	turquoise	-0.4459071	2.01E-10	0.25618918	0.00043178	0.75570454
CABYR	turquoise	0.69534327	4.69E-28	-0.8118597	1.24E-44	-0.4821724
CAB39L	turquoise	-0.3767278	1.25E-07	0.47839435	5.69E-12	0.52464481
CAB39	turquoise	0.58747814	1.50E-18	-0.2702534	0.00019891	-0.802146
CA10	turquoise	0.5928433	6.09E-19	-0.7632063	1.50E-36	-0.4449128
C9orf72	turquoise	0.7423636	1.17E-33	-0.3672359	2.72E-07	-0.5515455
C9orf40	turquoise	0.53697869	3.29E-15	-0.386601	5.46E-08	-0.458705
C8orf82	turquoise	-0.5704092	2.34E-17	0.29434693	4.76E-05	0.62195012
C8orf59	turquoise	0.52396062	1.96E-14	0.02754419	0.70976418	-0.4084145
C8orf46	turquoise	0.5674501	3.71E-17	-0.3525284	8.60E-07	-0.7807682
C6orf120	turquoise	0.74074203	1.92E-33	-0.3029442	2.77E-05	-0.7408304
C5orf42	turquoise	0.70762441	2.07E-29	-0.29667	4.12E-05	-0.6133701
C5orf34	turquoise	0.66694936	3.62E-25	-0.5426896	1.47E-15	-0.6280059
C5orf24	turquoise	0.28792044	7.07E-05	-0.0889851	0.22839005	-0.7342976
C5	turquoise	-0.4429709	2.72E-10	0.36028649	4.72E-07	0.67371123
C4orf33	turquoise	0.62013968	4.83E-21	-0.431274	8.87E-10	-0.4983983
C3orf14	turquoise	0.65918341	1.97E-24	-0.4309695	9.14E-10	-0.8041794
C2orf82	turquoise	-0.3242802	6.70E-06	0.05410984	0.46446084	0.55218323
C2orf80	turquoise	0.72218286	4.12E-31	-0.6740889	7.29E-26	-0.6849296
C2orf47	turquoise	0.61717895	8.35E-21	-0.2264775	0.00193567	-0.7521119
C21orf2	turquoise	-0.5441108	1.20E-15	0.14763424	0.04491653	0.83130108
C20orf24	turquoise	0.27505604	0.00015116	0.09290193	0.20847846	-0.7377038
C1QTNF5	turquoise	-0.5226759	2.32E-14	0.38391383	6.87E-08	0.36026514
C1QTNF1	turquoise	-0.6233463	2.65E-21	0.59079651	8.60E-19	0.61901294
C1QL3	turquoise	0.63210463	4.97E-22	-0.691169	1.31E-27	-0.4584158

C1QBP	turquoise	0.38095817	8.82E-08	-0.1249019	0.09026994	-0.8722767
C1orf64	turquoise	-0.5706616	2.25E-17	0.64321034	5.50E-23	0.1704919
C1orf53	turquoise	0.41863183	3.03E-09	-0.1410175	0.05554044	-0.6675029
C1orf52	turquoise	0.51619993	5.46E-14	-0.3264566	5.76E-06	-0.551164
C1orf216	turquoise	0.34128642	1.99E-06	-0.753298	3.87E-35	-0.3807573
C1orf198	turquoise	-0.7624512	1.94E-36	0.61193036	2.17E-20	0.41325192
C1orf162	turquoise	-0.5134621	7.80E-14	0.63247203	4.62E-22	0.50844685
C1orf109	turquoise	0.54596694	9.18E-16	-0.3060246	2.27E-05	-0.7107494
C19orf54	turquoise	-0.5858045	1.97E-18	0.40508339	1.07E-08	0.72621864
C19orf25	turquoise	-0.6610321	1.32E-24	0.44438598	2.35E-10	0.61935708
C18orf32	turquoise	0.3955378	2.51E-08	0.05193031	0.48266976	-0.2216737
C17orf97	turquoise	0.4967411	6.41E-13	-0.6705405	1.63E-25	-0.2017165
C17orf58	turquoise	0.33309571	3.61E-06	-0.4337451	6.94E-10	-0.2447866
C17orf100	turquoise	0.5895761	1.06E-18	-0.5570547	1.80E-16	-0.563182
C16orf58	turquoise	0.44335009	2.62E-10	-0.6035983	9.57E-20	-0.0435875
C15orf52	turquoise	-0.5490994	5.83E-16	0.56220799	8.29E-17	0.84423989
C14orf93	turquoise	-0.6152672	1.18E-20	0.47863731	5.54E-12	0.75695963
C14orf2	turquoise	0.51086794	1.09E-13	-0.610753	2.69E-20	-0.7841352
C12orf76	turquoise	0.53997268	2.16E-15	-0.5216409	2.67E-14	-0.7183415
C12orf43	turquoise	0.53381652	5.11E-15	-0.4580173	5.56E-11	-0.6655183
C12orf4	turquoise	0.59064336	8.83E-19	-0.3705416	2.08E-07	-0.79357
C12orf29	turquoise	0.32865123	4.94E-06	0.1731204	0.01844538	-0.4272914
C11orf96	turquoise	-0.4473755	1.72E-10	0.38598867	5.76E-08	0.25227861
C11orf87	turquoise	0.69763837	2.65E-28	-0.7983081	3.68E-42	-0.4904551
C11orf74	turquoise	0.51244756	8.89E-14	-0.3556954	6.74E-07	-0.548716
C10orf88	turquoise	0.76409812	1.11E-36	-0.57686	8.43E-18	-0.4376662
C10orf54	turquoise	-0.5149978	6.39E-14	0.73330835	1.74E-32	0.58204228
C10orf35	turquoise	0.58024386	4.89E-18	-0.8613314	1.03E-55	-0.1820947
C10orf10	turquoise	-0.5846882	2.37E-18	0.4726668	1.10E-11	0.34301859
BZW2	turquoise	0.73836142	3.91E-33	-0.7271195	1.03E-31	-0.6493512
BTN3A1	turquoise	-0.3910315	3.73E-08	0.49085037	1.31E-12	0.67054793
BTN2A1	turquoise	0.50634628	1.94E-13	0.02256345	0.7604767	-0.6196761
BTF3L4	turquoise	0.56671737	4.15E-17	-0.2372077	0.00114986	-0.8263676
BTBD3	turquoise	0.69555015	4.46E-28	-0.6422478	6.68E-23	-0.6654113
BTBD2	turquoise	-0.4636042	3.02E-11	0.16228865	0.02731021	0.76561808
BTBD10	turquoise	0.67907532	2.32E-26	-0.5242626	1.88E-14	-0.8412058
BTBD1	turquoise	0.40608511	9.75E-09	0.05793966	0.43340305	-0.7332481
BSN	turquoise	0.44974984	1.34E-10	-0.6638422	7.17E-25	-0.3334811
BSDC1	turquoise	-0.5179669	4.33E-14	0.11504966	0.11889679	0.73606585
BSCL2	turquoise	0.37118598	1.98E-07	-0.8392598	2.63E-50	-0.2939301
BRWD1	turquoise	0.68294936	9.37E-27	-0.7895549	1.16E-40	-0.587467
BRSK1	turquoise	-0.5190638	3.75E-14	0.01623027	0.82643684	0.55798709
BRINP1	turquoise	0.60885585	3.77E-20	-0.604276	8.50E-20	-0.5779337
BRF2	turquoise	0.37860991	1.07E-07	-0.5835004	2.88E-18	-0.7491986
BRE	turquoise	0.42295045	2.00E-09	-0.7704053	1.28E-37	-0.661616
BRAF	turquoise	0.72151667	4.95E-31	-0.4830517	3.31E-12	-0.7330913

BPTF	turquoise	-0.4482171	1.58E-10	0.40356126	1.23E-08	0.38518264
BPGM	turquoise	0.56288923	7.47E-17	-0.0805013	0.27602896	-0.7250383
BORCS7	turquoise	0.56240947	8.04E-17	-0.145485	0.0481634	-0.8021544
BOLA3	turquoise	0.23714911	0.00115321	-0.0685985	0.35350607	-0.7727416
BOLA2	turquoise	-0.6295845	8.08E-22	0.34578365	1.43E-06	0.70084879
BOC	turquoise	-0.4675548	1.95E-11	0.67767168	3.21E-26	0.62307629
BNIP3L	turquoise	0.68475522	6.11E-27	-0.3078715	2.02E-05	-0.2868981
BNIP3	turquoise	0.57476061	1.18E-17	-0.3709697	2.01E-07	-0.729871
BMPR2	turquoise	0.6689647	2.31E-25	-0.3476376	1.24E-06	-0.7530005
BMPER	turquoise	0.68699394	3.59E-27	-0.5253101	1.63E-14	-0.820602
BMP1	turquoise	-0.5966419	3.19E-19	0.54682086	8.12E-16	0.35784752
BLVRA	turquoise	0.62008947	4.87E-21	-0.5297565	8.93E-15	-0.2096849
BIVM	turquoise	0.54305136	1.39E-15	-0.2505054	0.00058354	-0.5313999
BIN1	turquoise	-0.4904532	1.38E-12	-0.0051379	0.94466357	0.15331764
BHLHE40	turquoise	0.12409159	0.09239463	-0.0491405	0.50652735	-0.434907
BHLHE22	turquoise	0.60032284	1.69E-19	-0.6638604	7.14E-25	-0.4986766
BHLHB9	turquoise	0.72731992	9.73E-32	-0.523753	2.01E-14	-0.7142413
BGN	turquoise	-0.6383083	1.47E-22	0.66998165	1.84E-25	0.73568494
BEX5	turquoise	0.68979457	1.83E-27	-0.6984433	2.17E-28	-0.807267
BEX4	turquoise	0.58253909	3.37E-18	-0.4284334	1.17E-09	-0.950231
BEX2	turquoise	0.68911649	2.15E-27	-0.7301429	4.34E-32	-0.7714615
BEX1	turquoise	0.67986007	1.93E-26	-0.7394967	2.79E-33	-0.7461477
BEND7	turquoise	-0.3161062	1.17E-05	0.72640225	1.26E-31	0.58667721
BEND6	turquoise	0.60915302	3.58E-20	-0.4784029	5.69E-12	-0.7518582
BECN1	turquoise	0.4881079	1.82E-12	-0.3327207	3.70E-06	-0.7482358
BDNF	turquoise	0.61640617	9.62E-21	-0.651118	1.08E-23	-0.539433
BCLAF1	turquoise	0.54605751	9.06E-16	-0.2746161	0.00015504	-0.7275824
BCL6B	turquoise	-0.4527575	9.77E-11	0.54799524	6.85E-16	0.71994548
BCL6	turquoise	-0.5692631	2.80E-17	0.65035313	1.27E-23	0.65206137
BCL3	turquoise	-0.589204	1.12E-18	0.38878345	4.53E-08	0.21544771
BCL2	turquoise	-0.4493974	1.39E-10	0.76216488	2.13E-36	0.60946171
BCL11B	turquoise	0.6176992	7.58E-21	-0.638241	1.49E-22	-0.2994374
BCCIP	turquoise	0.75974438	4.76E-36	-0.5037904	2.67E-13	-0.5020328
BCAT2	turquoise	-0.6880311	2.80E-27	0.59459002	4.53E-19	0.4899981
BCAT1	turquoise	0.71329201	4.63E-30	-0.4066558	9.26E-09	-0.7297135
BCAS2	turquoise	0.79074168	7.34E-41	-0.3557137	6.73E-07	-0.5197623
BCAS1	turquoise	-0.6051077	7.34E-20	0.4320425	8.22E-10	0.4483266
BCAN	turquoise	-0.6970012	3.11E-28	0.54314989	1.38E-15	0.46977742
BBX	turquoise	-0.5393582	2.36E-15	0.70876501	1.53E-29	0.33774653
BBS7	turquoise	0.58149375	3.99E-18	-0.6926329	9.15E-28	-0.3849221
BBS4	turquoise	-0.3099855	1.76E-05	0.0810769	0.27259859	0.79848183
BBS10	turquoise	0.49940179	4.62E-13	0.11629163	0.11494065	-0.5682752
BBC3	turquoise	-0.5331109	5.63E-15	0.32110404	8.33E-06	0.78596762
BAZ2B	turquoise	-0.5794148	5.59E-18	0.70936733	1.31E-29	0.67699844
BAZ1A	turquoise	-0.5292907	9.51E-15	0.80352572	4.33E-43	0.54155241
BASP1	turquoise	0.5933376	5.61E-19	-0.7206325	6.32E-31	-0.7144879

BARD1	turquoise	-0.4500372	1.30E-10	0.3795168	9.95E-08	0.63491093
BAP1	turquoise	-0.4973052	5.98E-13	0.20327467	0.00551798	0.64243457
BANP	turquoise	-0.5195515	3.52E-14	0.36247326	3.97E-07	0.64107445
BAIAP2	turquoise	0.34067231	2.09E-06	-0.7306898	3.71E-32	-0.4730046
BAGE5	turquoise	-0.3897918	4.15E-08	0.39990778	1.70E-08	0.57951494
BAG5	turquoise	0.55985667	1.18E-16	-0.7975038	5.09E-42	-0.2705827
BAG3	turquoise	-0.460151	4.41E-11	0.69035348	1.59E-27	0.29090323
BACE2	turquoise	-0.4548424	7.82E-11	0.41401575	4.68E-09	0.61228653
B4GAT1	turquoise	0.52306598	2.20E-14	-0.8245226	3.93E-47	-0.3966061
B4GALT6	turquoise	0.77077649	1.13E-37	-0.585792	1.98E-18	-0.697522
B4GALT5	turquoise	0.65172969	9.54E-24	-0.5710609	2.11E-17	-0.7097475
B3GALT2	turquoise	0.61996422	4.99E-21	-0.4095184	7.12E-09	-0.341548
B3GALNT1	turquoise	0.71178175	6.93E-30	-0.6684965	2.57E-25	-0.5688066
AZIN1	turquoise	0.62058132	4.45E-21	-0.2651084	0.00026543	-0.8378518
AZGP1	turquoise	-0.4745052	8.90E-12	0.49855846	5.13E-13	0.38739305
AUP1	turquoise	-0.5577562	1.62E-16	0.3445199	1.57E-06	0.78151772
AUNIP	turquoise	0.65392953	6.02E-24	-0.7187194	1.07E-30	-0.2552014
AUH	turquoise	0.66408035	6.81E-25	-0.4671479	2.04E-11	-0.8470057
ATXN7L2	turquoise	-0.3977103	2.07E-08	-0.1548162	0.03536464	0.48214123
ATXN3	turquoise	-0.3584932	5.43E-07	0.05989472	0.4180195	0.85577408
ATXN1L	turquoise	-0.4765863	7.01E-12	0.67628458	4.42E-26	0.53400379
ATRNL1	turquoise	0.65193782	9.14E-24	-0.5729494	1.57E-17	-0.7442997
ATRN	turquoise	0.61039163	2.87E-20	-0.5361821	3.68E-15	-0.8824851
ATPIF1	turquoise	0.58239785	3.45E-18	-0.76489	8.53E-37	-0.3062434
ATPAF1	turquoise	0.51969883	3.45E-14	-0.7498339	1.16E-34	-0.2326665
ATP9A	turquoise	0.63804362	1.55E-22	-0.7677953	3.17E-37	-0.4011382
ATP6V1H	turquoise	0.65913679	1.99E-24	-0.5419183	1.64E-15	-0.8217522
ATP6V1G2	turquoise	0.57875427	6.22E-18	-0.6223028	3.22E-21	-0.8801181
ATP6V1E1	turquoise	0.61583631	1.07E-20	-0.8447819	1.40E-51	-0.5466229
ATP6V1D	turquoise	0.69034139	1.60E-27	-0.6831863	8.86E-27	-0.7542266
ATP6V1C1	turquoise	0.59002297	9.79E-19	-0.7136815	4.18E-30	-0.5477596
ATP6V1B2	turquoise	0.64564989	3.35E-23	-0.808132	6.20E-44	-0.6710534
ATP6V0E2	turquoise	0.30130029	3.08E-05	-0.5414943	1.74E-15	0.02457758
ATP6V0E1	turquoise	-0.5492685	5.69E-16	0.83819188	4.58E-50	0.59334
ATP6AP2	turquoise	0.59611298	3.50E-19	-0.3529954	8.30E-07	-0.9079882
ATP5O	turquoise	0.39522767	2.58E-08	-0.1379441	0.0611393	-0.9093902
ATP5L	turquoise	0.66192066	1.09E-24	-0.4853974	2.51E-12	-0.7605681
ATP5J	turquoise	0.35134716	9.41E-07	-0.0029219	0.9685133	-0.8062177
ATP5G3	turquoise	0.58713592	1.58E-18	-0.1712838	0.01974409	-0.6988447
ATP5F1	turquoise	0.41888991	2.96E-09	-0.058187	0.4314393	-0.8672114
ATP5B	turquoise	0.59351085	5.44E-19	-0.4775745	6.26E-12	-0.8561095
ATP5A1	turquoise	0.49429447	8.64E-13	-0.2324792	0.00145068	-0.9146974
ATP2C1	turquoise	0.73915654	3.08E-33	-0.5289988	9.90E-15	-0.5807343
ATP2B3	turquoise	0.66448174	6.23E-25	-0.6699853	1.84E-25	-0.8186206
ATP2B2	turquoise	0.58254041	3.37E-18	-0.7331232	1.83E-32	-0.5915228
ATP2B1	turquoise	0.66782282	2.98E-25	-0.6588117	2.13E-24	-0.2914731

ATP2A2	turquoise	0.6078595	4.51E-20	-0.4822455	3.64E-12	-0.8875522
ATP1B1	turquoise	0.62595163	1.62E-21	-0.7256099	1.58E-31	-0.694367
ATP1A1	turquoise	0.5382712	2.74E-15	-0.6719684	1.18E-25	-0.8309804
ATP10A	turquoise	-0.4502062	1.28E-10	0.58637422	1.80E-18	0.69185459
ATMIN	turquoise	0.41476201	4.37E-09	0.003618	0.96101751	-0.7758079
ATL1	turquoise	0.39180042	3.49E-08	-0.6388453	1.32E-22	-0.2314511
ATG2A	turquoise	-0.3583839	5.47E-07	-0.1008879	0.17180971	0.50361143
ATG16L2	turquoise	-0.4534604	9.06E-11	0.17796875	0.01536711	0.78739311
ATG101	turquoise	-0.3044571	2.51E-05	-0.1440024	0.05051541	0.5434978
ATCAY	turquoise	0.4206829	2.49E-09	-0.5207794	2.99E-14	-0.6848793
ATAD1	turquoise	0.71768554	1.42E-30	-0.2259523	0.00198447	-0.5695419
ASTN2	turquoise	-0.3951873	2.59E-08	0.03931129	0.5952298	0.69196311
ASTN1	turquoise	0.43442983	6.48E-10	-0.2727548	0.00017252	-0.8281327
ASRGL1	turquoise	-0.440997	3.33E-10	0.64060363	9.29E-23	0.40186442
ASPSCR1	turquoise	-0.5291973	9.63E-15	0.16275356	0.02686573	0.87536724
ASPHD2	turquoise	0.42354183	1.89E-09	-0.7013379	1.04E-28	-0.2660943
ASPH	turquoise	0.67807172	2.93E-26	-0.3656333	3.09E-07	-0.5745222
ASNSD1	turquoise	0.44791262	1.63E-10	-0.0889979	0.22832303	-0.8121031
ASNS	turquoise	0.53783643	2.92E-15	-0.6857031	4.88E-27	-0.5784323
ASL	turquoise	-0.3600936	4.79E-07	0.12952901	0.07887448	0.72847102
ASIC2	turquoise	0.39584472	2.45E-08	-0.8263983	1.61E-47	-0.2454979
ASH2L	turquoise	0.59054001	8.98E-19	-0.5725511	1.67E-17	-0.6919859
ASCL1	turquoise	-0.4198289	2.70E-09	0.79218921	4.18E-41	0.52990258
ASB6	turquoise	-0.679703	2.00E-26	0.40681709	9.12E-09	0.66062701
ARV1	turquoise	0.55370912	2.97E-16	-0.1993511	0.00651928	-0.7430084
ARSK	turquoise	0.63944606	1.17E-22	-0.3594869	5.02E-07	-0.6519971
ARSA	turquoise	-0.2832511	9.35E-05	0.14905127	0.04287753	0.80033075
ARRDC4	turquoise	-0.3130533	1.43E-05	0.50840257	1.49E-13	0.46517858
ARRDC2	turquoise	-0.5594968	1.25E-16	0.42440698	1.74E-09	0.78373703
ARPC5L	turquoise	0.58069961	4.54E-18	-0.7994865	2.28E-42	-0.280629
ARPC1A	turquoise	0.43102799	9.09E-10	-0.3109908	1.64E-05	-0.7361894
ARMCX5	turquoise	0.71996737	7.59E-31	-0.5580817	1.55E-16	-0.7538324
ARMCX3	turquoise	0.47635385	7.20E-12	-0.0817691	0.26851204	-0.7934529
ARMCX1	turquoise	0.46329684	3.12E-11	-0.0850469	0.24972871	-0.8849536
ARMC8	turquoise	0.61604061	1.03E-20	-0.3240533	6.80E-06	-0.7824934
ARMC10	turquoise	0.5086449	1.45E-13	-0.283213	9.37E-05	-0.9151589
ARL6IP5	turquoise	0.6762624	4.44E-26	-0.4300479	1.00E-09	-0.4837418
ARL6IP1	turquoise	0.57755643	7.54E-18	-0.3958879	2.44E-08	-0.8366311
ARL6	turquoise	0.78103933	2.86E-39	-0.5079973	1.57E-13	-0.6544011
ARL5A	turquoise	-0.2646637	0.00027205	0.21581399	0.00317424	0.68487007
ARL4A	turquoise	0.62942374	8.34E-22	-0.621618	3.66E-21	-0.3522547
ARL3	turquoise	0.34303312	1.75E-06	-0.5265826	1.37E-14	-0.6110421
ARL2BP	turquoise	0.58134696	4.09E-18	-0.3727991	1.73E-07	-0.6960129
ARL1	turquoise	0.66356355	7.62E-25	-0.4351929	6.00E-10	-0.8299761
ARID3B	turquoise	-0.5222129	2.47E-14	0.18395309	0.01219326	0.80575408
ARHGEF3	turquoise	0.62535332	1.81E-21	-0.7363855	7.04E-33	-0.5673836

ARHGEF10L	turquoise	-0.3628486	3.86E-07	0.15594634	0.0340306	0.68583797
ARHGEF10	turquoise	-0.6027028	1.12E-19	0.8355112	1.81E-49	0.53058687
ARHGDIB	turquoise	-0.2872124	7.37E-05	0.66133404	1.24E-24	0.38036923
ARHGAP9	turquoise	-0.3961071	2.39E-08	0.55506388	2.43E-16	0.37528644
ARHGAP44	turquoise	0.49879231	4.98E-13	-0.5594808	1.25E-16	-0.3532181
ARHGAP4	turquoise	-0.4432526	2.64E-10	0.29471156	4.66E-05	0.69125024
ARHGAP33	turquoise	-0.5352288	4.20E-15	0.1811117	0.01362022	0.5391878
ARHGAP32	turquoise	0.53517612	4.23E-15	-0.5565206	1.95E-16	-0.8584647
ARHGAP31	turquoise	-0.4823666	3.59E-12	0.76881378	2.23E-37	0.16157623
ARHGAP30	turquoise	-0.5333675	5.44E-15	0.63119038	5.93E-22	0.37236244
ARHGAP22	turquoise	-0.4504443	1.25E-10	0.2936154	4.99E-05	0.71409943
ARHGAP20	turquoise	0.68408085	7.17E-27	-0.5206736	3.03E-14	-0.8240614
ARHGAP19	turquoise	-0.3867219	5.41E-08	0.55082511	4.53E-16	0.58874319
ARHGAP10	turquoise	-0.3709804	2.01E-07	0.23559911	0.0012451	0.75356727
ARGLU1	turquoise	0.28010552	0.00011264	0.19122097	0.00912426	-0.5950274
ARFGEF3	turquoise	0.59341154	5.54E-19	-0.5131671	8.10E-14	-0.4693401
ARF1	turquoise	0.40409045	1.17E-08	-0.7044565	4.70E-29	-0.5323797
ARAP1	turquoise	-0.5832743	2.99E-18	0.40837213	7.91E-09	0.70014978
AQP11	turquoise	0.66942885	2.09E-25	-0.3482319	1.19E-06	-0.4943108
APRT	turquoise	-0.4920531	1.13E-12	0.21186818	0.00379005	0.63092198
APPBP2	turquoise	0.64118021	8.28E-23	-0.3020705	2.93E-05	-0.6145727
APP	turquoise	0.43530436	5.93E-10	-0.3873417	5.13E-08	-0.8936522
APOO	turquoise	0.50871755	1.43E-13	-0.3629572	3.82E-07	-0.8899172
APOLD1	turquoise	-0.456651	6.44E-11	0.66620792	4.26E-25	0.21687397
APOL3	turquoise	-0.6079025	4.47E-20	0.59578004	3.70E-19	0.45151075
APOC1	turquoise	-0.3712547	1.97E-07	0.46884192	1.69E-11	0.50673278
APOBEC3F	turquoise	-0.4300764	9.99E-10	-0.0079472	0.9145014	0.73601338
APLNR	turquoise	-0.334858	3.18E-06	0.2466663	0.00071246	0.49651871
APLN	turquoise	-0.2614499	0.00032473	0.28362152	9.15E-05	0.26528321
APBA2	turquoise	0.418114	3.18E-09	-0.7146009	3.26E-30	-0.6435157
AP5M1	turquoise	0.65742345	2.87E-24	-0.3214727	8.12E-06	-0.1488185
AP3S1	turquoise	0.47465384	8.75E-12	-0.129084	0.0799173	-0.8312343
AP3M2	turquoise	0.61526364	1.19E-20	-0.8227414	9.07E-47	-0.4219261
AP1S2	turquoise	0.74026845	2.21E-33	-0.5508581	4.51E-16	-0.4727751
AP1S1	turquoise	0.59451182	4.59E-19	-0.5196017	3.49E-14	-0.835307
ANXA7	turquoise	0.46574076	2.38E-11	-0.1763317	0.01635216	-0.6050047
ANXA6	turquoise	0.55558898	2.24E-16	-0.6947309	5.46E-28	-0.5245669
ANXA2	turquoise	-0.452687	9.84E-11	0.6458639	3.21E-23	0.37155274
ANP32B	turquoise	-0.5563682	2.00E-16	0.52849314	1.06E-14	0.7263233
ANO3	turquoise	0.61513623	1.21E-20	-0.7389095	3.32E-33	-0.4393653
ANKRD9	turquoise	-0.4637774	2.96E-11	0.20557289	0.00499761	0.50803397
ANKRD6	turquoise	0.61273772	1.88E-20	-0.522356	2.42E-14	-0.2835652
ANKRD50	turquoise	0.42581943	1.52E-09	-0.523418	2.10E-14	-0.5789413
ANKRD46	turquoise	0.68034054	1.73E-26	-0.5444026	1.15E-15	-0.7678296
ANKRD40	turquoise	-0.6396067	1.13E-22	0.50506867	2.28E-13	0.55760989
ANKRD34A	turquoise	0.45630832	6.68E-11	-0.7547008	2.47E-35	-0.4349845

ANKRD33	turquoise	-0.3261514	5.88E-06	0.10262315	0.1645174	0.6287959
ANKRD29	turquoise	0.60835123	4.13E-20	-0.279246	0.00011847	-0.4780677
ANKRD27	turquoise	0.43257576	7.79E-10	-0.131967	0.07335449	-0.5139493
ANKRD20A1	turquoise	-0.4347517	6.27E-10	0.13310808	0.07088078	0.67011346
ANKRD18A	turquoise	-0.3377906	2.57E-06	0.0821876	0.26606156	0.64679411
ANKRD13C	turquoise	0.59162039	7.49E-19	-0.1978884	0.00693218	-0.7935419
ANKRA2	turquoise	0.65042523	1.25E-23	-0.1866874	0.01094588	-0.570981
ANKMY2	turquoise	0.64236389	6.52E-23	-0.3939374	2.89E-08	-0.8213858
ANKLE2	turquoise	0.50149534	3.56E-13	-0.2061123	0.00488204	-0.7238139
ANKIB1	turquoise	-0.3506281	9.93E-07	0.68036995	1.72E-26	0.5485325
ANKFY1	turquoise	-0.4624594	3.42E-11	0.77763577	9.88E-39	0.3360859
ANKDD1A	turquoise	-0.1770955	0.01588594	0.13862047	0.05986887	0.54514369
ANK3	turquoise	0.59895441	2.15E-19	-0.4918134	1.17E-12	-0.2429742
ANGPTL4	turquoise	-0.5419375	1.63E-15	0.35802945	5.63E-07	0.47049419
ANGPT2	turquoise	-0.398712	1.90E-08	0.56446084	5.87E-17	0.37216297
ANAPC16	turquoise	-0.302019	2.94E-05	0.72568203	1.55E-31	0.60356022
ANAPC13	turquoise	0.59557186	3.84E-19	-0.3732793	1.67E-07	-0.7526785
ANAPC10	turquoise	0.49958626	4.52E-13	0.00032854	0.99645872	-0.7327508
AMY2A	turquoise	0.43573383	5.68E-10	-0.2980975	3.77E-05	-0.0508776
AMY1A	turquoise	0.48499536	2.63E-12	-0.3433246	1.72E-06	-0.0073054
AMPH	turquoise	0.71326065	4.67E-30	-0.7064596	2.80E-29	-0.6703158
AMACR	turquoise	0.60336844	9.97E-20	-0.3615395	4.28E-07	-0.7388559
ALPL	turquoise	-0.4272521	1.32E-09	0.49290838	1.02E-12	0.43247322
ALG6	turquoise	0.33203465	3.89E-06	0.09800682	0.1844428	-0.4339332
ALDH8A1	turquoise	-0.3913738	3.62E-08	0.09070865	0.21946881	0.84569174
ALDH5A1	turquoise	0.38691474	5.32E-08	0.03531389	0.6332096	-0.6445376
ALDH1L1	turquoise	-0.6689179	2.34E-25	0.55014153	5.01E-16	0.45180833
AKT1	turquoise	-0.7156104	2.49E-30	0.41234942	5.47E-09	0.73525784
AKR1C3	turquoise	-0.528035	1.13E-14	0.80137466	1.05E-42	0.63031614
AKNA	turquoise	-0.5248533	1.73E-14	0.41591518	3.92E-09	0.38409069
AKIRIN2	turquoise	0.4790944	5.25E-12	-0.1925553	0.00864191	-0.886364
AKAP8L	turquoise	-0.4067623	9.17E-09	-0.1099677	0.1361959	0.36912489
AKAP6	turquoise	0.63241014	4.68E-22	-0.5751819	1.10E-17	-0.7652831
AKAP12	turquoise	0.50823436	1.53E-13	-0.0379235	0.60829829	-0.1286356
AKAP11	turquoise	0.70560296	3.50E-29	-0.486471	2.21E-12	-0.8183503
AK5	turquoise	0.57565798	1.02E-17	-0.7185574	1.12E-30	-0.6246111
AK4	turquoise	-0.3443321	1.59E-06	0.50781337	1.61E-13	0.59168529
AK2	turquoise	-0.641104	8.40E-23	0.61975739	5.18E-21	0.48373585
AIMP2	turquoise	0.32036631	8.76E-06	-0.1583009	0.03138788	-0.2667118
AIG1	turquoise	0.38436169	6.61E-08	-0.2482844	0.00065522	-0.8447287
AIFM3	turquoise	-0.6594467	1.86E-24	0.47175662	1.22E-11	0.52232178
AIF1L	turquoise	-0.5917052	7.38E-19	0.43622943	5.40E-10	0.60151753
AHNAK	turquoise	-0.6469888	2.55E-23	0.76889827	2.16E-37	0.70559278
AHI1	turquoise	0.59622487	3.43E-19	-0.7708507	1.10E-37	-0.3400585
AGPAT4	turquoise	0.37524728	1.42E-07	-0.639762	1.10E-22	-0.3253008
AGK	turquoise	0.70735858	2.22E-29	-0.5955518	3.85E-19	-0.809546

AGGF1	turquoise	0.75987899	4.56E-36	-0.5540116	2.84E-16	-0.66785
AGFG2	turquoise	-0.5875546	1.48E-18	0.52860182	1.04E-14	0.43849519
AGFG1	turquoise	0.54517372	1.03E-15	-0.09147	0.21560809	-0.740349
AFTPH	turquoise	0.56002552	1.15E-16	-0.7172026	1.62E-30	-0.6533364
AFF3	turquoise	0.5456892	9.56E-16	-0.4882017	1.80E-12	-0.3397196
AFF2	turquoise	0.7002043	1.39E-28	-0.7362267	7.37E-33	-0.4777729
AEBP1	turquoise	-0.5091608	1.36E-13	0.56572007	4.84E-17	0.47884219
ADORA2A	turquoise	-0.5714451	1.99E-17	0.46726253	2.01E-11	0.60373372
ADO	turquoise	0.67158548	1.29E-25	-0.4622075	3.52E-11	-0.5292425
ADIRF	turquoise	-0.6353708	2.62E-22	0.50932951	1.33E-13	0.64887342
ADH1A	turquoise	-0.4259298	1.50E-09	0.65357959	6.48E-24	0.25912555
ADGRL3	turquoise	0.5529842	3.30E-16	-0.0009397	0.98987185	-0.4026194
ADGRL2	turquoise	0.45777699	5.70E-11	-0.4883473	1.77E-12	-0.2712852
ADGRG1	turquoise	-0.6036662	9.46E-20	0.64108748	8.43E-23	0.14872341
ADGRB3	turquoise	0.61797734	7.20E-21	-0.3389393	2.37E-06	-0.7948248
ADGRA2	turquoise	-0.5307578	7.78E-15	0.39161388	3.54E-08	0.58266966
ADCYAP1	turquoise	0.62678996	1.38E-21	-0.7668876	4.32E-37	-0.5038263
ADCY4	turquoise	-0.529825	8.84E-15	0.44810115	1.60E-10	0.73540486
ADCY1	turquoise	0.53356592	5.29E-15	-0.6239591	2.36E-21	-0.7867652
ADARB2	turquoise	-0.4033049	1.26E-08	0.57481873	1.17E-17	0.33229945
ADARB1	turquoise	0.60990429	3.13E-20	-0.6986563	2.05E-28	-0.3810635
ADAP2	turquoise	-0.3272517	5.45E-06	0.50863167	1.45E-13	0.59144806
ADAP1	turquoise	-0.3733578	1.66E-07	0.02883673	0.69679944	0.77203771
ADAMTSL2	turquoise	-0.3981833	1.99E-08	0.17447529	0.01753545	0.72763784
ADAMTS9	turquoise	-0.2742517	0.00015833	0.36630895	2.93E-07	0.22996633
ADAMTS8	turquoise	0.39452053	2.75E-08	-0.6454024	3.52E-23	-0.2940591
ADAMTS1	turquoise	-0.3544222	7.44E-07	0.58085037	4.43E-18	0.26054899
ADAM23	turquoise	0.60347212	9.79E-20	-0.802765	5.94E-43	-0.6363531
ADA	turquoise	-0.5030985	2.92E-13	0.27166201	0.00018362	0.39187252
ACYP2	turquoise	0.21428199	0.0034017	0.01244116	0.86652216	-0.8088669
ACYP1	turquoise	0.45252017	1.00E-10	-0.2669157	0.00024	-0.8305039
ACVR2A	turquoise	0.63182411	5.24E-22	-0.3617141	4.22E-07	-0.8146933
ACTR6	turquoise	0.63454625	3.08E-22	-0.3968607	2.24E-08	-0.6429137
ACTR3B	turquoise	0.57801276	7.01E-18	-0.5884245	1.28E-18	-0.8364102
ACTR3	turquoise	0.53823291	2.76E-15	-0.1915532	0.00900198	-0.0987294
ACTR2	turquoise	0.59027498	9.39E-19	-0.2382689	0.00109075	-0.7193605
ACTR10	turquoise	0.5746623	1.20E-17	-0.3806619	9.04E-08	-0.9183567
ACTN4	turquoise	-0.6242442	2.24E-21	0.14308618	0.05201602	0.33231179
ACTN2	turquoise	0.70874686	1.54E-29	-0.6665744	3.93E-25	-0.7282286
ACTN1	turquoise	-0.5605239	1.07E-16	0.23252639	0.00144736	0.72368601
ACTL6B	turquoise	0.39931407	1.80E-08	-0.8452377	1.10E-51	-0.2511149
ACTG1	turquoise	0.43693609	5.03E-10	-0.4392081	4.00E-10	-0.6809692
ACTB	turquoise	0.20838989	0.00442005	-0.4673442	2.00E-11	-0.7278217
ACTA2	turquoise	-0.321171	8.29E-06	0.59129226	7.92E-19	0.21237414
ACSS3	turquoise	-0.4428125	2.76E-10	0.68205857	1.16E-26	0.659383
ACSS2	turquoise	-0.3782298	1.11E-07	0.15430332	0.03598444	0.8181858

ACSS1	turquoise	-0.5483052	6.55E-16	0.52206474	2.52E-14	0.75933471
ACSL6	turquoise	0.34112871	2.02E-06	-0.2059759	0.00491104	-0.4401101
ACSL4	turquoise	0.6842322	6.92E-27	-0.4175868	3.34E-09	-0.8500447
ACSL3	turquoise	0.45779665	5.69E-11	-0.0916076	0.21491556	-0.808319
ACSF2	turquoise	-0.5377646	2.95E-15	0.4424847	2.86E-10	0.80583611
ACSBG1	turquoise	-0.5630465	7.29E-17	0.67874388	2.50E-26	0.52295747
ACP1	turquoise	0.51874304	3.91E-14	-0.2926774	5.28E-05	-0.9234608
ACOT7	turquoise	0.62692062	1.35E-21	-0.8013692	1.06E-42	-0.317414
ACOT11	turquoise	-0.32199	7.84E-06	0.4408824	3.37E-10	0.55662732
ACKR3	turquoise	-0.324136	6.76E-06	0.77424627	3.33E-38	0.44342841
ACER3	turquoise	-0.5696621	2.63E-17	0.3703137	2.12E-07	0.45335812
ACAT2	turquoise	0.50023476	4.17E-13	-0.3048875	2.45E-05	-0.8207372
ACADVL	turquoise	-0.7734902	4.35E-38	0.53526127	4.18E-15	0.48293023
ACAD8	turquoise	0.2756405	0.00014614	0.05597441	0.44918906	-0.7802412
ACAA1	turquoise	-0.6324401	4.65E-22	0.33915758	2.33E-06	0.27301552
ABRACL	turquoise	0.50491871	2.32E-13	-0.5057603	2.09E-13	-0.2360243
ABLIM1	turquoise	-0.5826222	3.32E-18	0.69790643	2.48E-28	0.47831331
ABI1	turquoise	0.46750887	1.96E-11	0.04878905	0.50957606	-0.5208069
ABCG1	turquoise	-0.441326	3.22E-10	0.472158	1.16E-11	0.51002138
ABCE1	turquoise	0.59343895	5.51E-19	-0.2968807	4.07E-05	-0.8597876
ABCC12	turquoise	0.64905329	1.66E-23	-0.6512719	1.05E-23	-0.3306575
ABCB10	turquoise	0.43249664	7.86E-10	0.13199293	0.07329748	-0.677609
ABCA7	turquoise	-0.3903907	3.94E-08	-0.0666599	0.36730613	0.54060244
ABCA5	turquoise	0.76356747	1.33E-36	-0.6840036	7.31E-27	-0.3971042
ABCA1	turquoise	-0.4125385	5.38E-09	0.7813633	2.54E-39	0.53083492
AATF	turquoise	-0.6564214	3.55E-24	0.35933951	5.08E-07	0.44024259
AASDHPPT	turquoise	0.70598246	3.17E-29	-0.454462	8.14E-11	-0.8057181
AACS	turquoise	0.35175909	9.12E-07	-0.6037688	9.29E-20	-0.5063543
ZNF22	yellow	-0.3812234	8.62E-08	0.76955301	1.73E-37	0.64222893
ZFP36L1	yellow	-0.2328661	0.00142361	0.74490833	5.38E-34	0.39561945
ZC3HAV1	yellow	-0.4014155	1.49E-08	0.76023788	4.05E-36	0.57536663
YJEFN3	yellow	0.21345742	0.00353012	-0.5335801	5.28E-15	-0.6768795
YES1	yellow	-0.3575785	5.83E-07	0.76474897	8.94E-37	0.64552253
VSTM2L	yellow	0.29176863	5.59E-05	-0.7465031	3.29E-34	-0.4919771
USP11	yellow	0.16522636	0.02460427	-0.714416	3.43E-30	-0.6058042
TRANK1	yellow	0.2447808	0.00078496	-0.6715691	1.29E-25	-0.4717888
TMEM151B	yellow	0.33405767	3.37E-06	-0.6398726	1.08E-22	-0.422314
THY1	yellow	0.19419829	0.00807914	-0.715062	2.88E-30	-0.37647
SYP	yellow	0.18305027	0.01263172	-0.5812555	4.15E-18	-0.7375464
SYNGR3	yellow	0.35224083	8.79E-07	-0.6542164	5.66E-24	-0.8139118
SYN1	yellow	0.22624719	0.00195693	-0.6403181	9.84E-23	-0.6036389
STXBP1	yellow	0.42182935	2.23E-09	-0.5737573	1.38E-17	-0.9227769
STX1A	yellow	0.19429825	0.00804598	-0.5836501	2.81E-18	-0.7210516
SNHG6	yellow	-0.4155572	4.05E-09	0.64055379	9.38E-23	0.66928378
SLC6A17	yellow	0.34247237	1.83E-06	-0.7168677	1.77E-30	-0.7146284
SCAMP5	yellow	0.19574559	0.00757915	-0.7401195	2.31E-33	-0.5204295

SCAF11	yellow	-0.4219843	2.20E-09	0.79678722	6.78E-42	0.67903853
RUNDC3A	yellow	0.24865419	0.00064275	-0.5726418	1.65E-17	-0.5396104
RNMT	yellow	0.35348891	7.99E-07	-0.6764202	4.28E-26	-0.7485502
RNF112	yellow	0.24873848	0.00063994	-0.7299597	4.58E-32	-0.4015478
PRRT1	yellow	0.2484597	0.00064928	-0.7115822	7.30E-30	-0.5925446
PPP2R5D	yellow	0.19538595	0.00769284	-0.6532131	7.00E-24	-0.5574569
PPME1	yellow	0.16647443	0.02352635	-0.8082837	5.81E-44	-0.3905017
PNMAL2	yellow	0.33003762	4.48E-06	-0.8080524	6.42E-44	-0.5461166
PNMA6A	yellow	0.36033646	4.70E-07	-0.8782049	1.62E-60	-0.4810332
PNKD	yellow	0.14180275	0.05418008	-0.6442051	4.49E-23	-0.6057408
PKNOX2	yellow	0.33837207	2.47E-06	-0.7072469	2.28E-29	-0.5834143
PAK6	yellow	0.25528235	0.00045324	-0.5260531	1.48E-14	-0.4541617
PAK2	yellow	-0.2931296	5.14E-05	0.65176593	9.47E-24	0.71899954
P4HTM	yellow	0.27310312	0.00016911	-0.7453685	4.67E-34	-0.4347495
OGDHL	yellow	0.27799737	0.00012744	-0.696341	3.66E-28	-0.5831879
NRGN	yellow	0.18716131	0.0107415	-0.656867	3.23E-24	-0.5865297
NPTXR	yellow	0.196461	0.00735742	-0.5685299	3.13E-17	-0.7000973
NPM2	yellow	0.29742801	3.93E-05	-0.6965592	3.47E-28	-0.5561478
NOMO1	yellow	0.27389908	0.00016157	-0.7043817	4.79E-29	-0.6111395
MICU1	yellow	0.22211662	0.00237612	-0.6251427	1.89E-21	-0.6580754
MICAL2	yellow	0.41569027	4.00E-09	-0.7145381	3.32E-30	-0.735348
MAST3	yellow	0.23153565	0.00151871	-0.7115541	7.36E-30	-0.3873021
LINGO1	yellow	0.28156107	0.00010337	-0.8199846	3.25E-46	-0.3491349
LAMC1	yellow	-0.4086915	7.68E-09	0.84846538	1.86E-52	0.55630894
KATNB1	yellow	0.2575019	0.00040237	-0.6996958	1.58E-28	-0.3842028
ICAM5	yellow	0.29679085	4.09E-05	-0.7114175	7.63E-30	-0.6695716
HSPB8	yellow	-0.3479823	1.21E-06	0.69106359	1.34E-27	0.57576435
GPI	yellow	0.16763442	0.0225612	-0.7116021	7.27E-30	-0.638453
GAS7	yellow	0.2657433	0.00025622	-0.6927448	8.90E-28	-0.6614337
FNDC3B	yellow	-0.2370362	0.00115969	0.67058177	1.61E-25	0.3137351
FERMT2	yellow	-0.3489377	1.13E-06	0.7906169	7.70E-41	0.48698155
FAM127A	yellow	0.32295141	7.34E-06	-0.7086031	1.60E-29	-0.633243
ENTPD6	yellow	0.21105185	0.00393016	-0.6458216	3.23E-23	-0.6595467
ENO2	yellow	0.40332514	1.25E-08	-0.8806436	2.85E-61	-0.5471385
EHD3	yellow	0.4560664	6.86E-11	-0.7220381	4.29E-31	-0.7058535
EFHD2	yellow	0.18951516	0.0097755	-0.6607344	1.41E-24	-0.2805656
DPP6	yellow	0.47285654	1.07E-11	-0.8513285	3.74E-53	-0.5917935
CRMP1	yellow	0.34185201	1.91E-06	-0.7488412	1.59E-34	-0.6147458
CPNE9	yellow	0.33454742	3.25E-06	-0.634517	3.10E-22	-0.6297291
CNTNAP1	yellow	0.23952911	0.00102417	-0.5928489	6.09E-19	-0.5376012
CLTB	yellow	0.41939193	2.82E-09	-0.6604559	1.50E-24	-0.7755677
CLSTN1	yellow	0.38635092	5.58E-08	-0.7086036	1.60E-29	-0.7168865
CKMT1B	yellow	0.45325329	9.26E-11	-0.7881268	2.01E-40	-0.5832173
CKMT1A	yellow	0.43104239	9.08E-10	-0.7772127	1.15E-38	-0.657622
CHST1	yellow	0.15367396	0.03675758	-0.5416605	1.70E-15	-0.6671956
CHRM1	yellow	0.34716914	1.29E-06	-0.7282834	7.40E-32	-0.7143558

CELSR2	yellow	0.23141762	0.00152743	-0.6324118	4.68E-22	-0.5375198
CDK5R2	yellow	0.3237789	6.93E-06	-0.7805484	3.42E-39	-0.3135111
CCDC184	yellow	0.47484598	8.56E-12	-0.7925255	3.67E-41	-0.5581325
CAMK1G	yellow	0.17660911	0.01618149	-0.6519947	9.03E-24	-0.7035107
CACNG3	yellow	0.35389647	7.74E-07	-0.7803408	3.69E-39	-0.4984169
C16orf45	yellow	0.32430007	6.69E-06	-0.7292155	5.67E-32	-0.7076236
ATXN7L3B	yellow	0.30581731	2.30E-05	-0.7659574	5.94E-37	-0.4788081
ARF5	yellow	0.19100239	0.0092055	-0.7253895	1.68E-31	-0.5131398
ABLIM2	yellow	0.43241208	7.92E-10	-0.7375209	5.03E-33	-0.6056453

p.MMblue	MMred	p.MMred	MMturquois	p.MMturquo	MMyellow	p.MMyellow
3.12E-06	-0.0536909	0.46793107	-0.1396804	0.05792192	0.26984584	0.00020355
1.82E-08	0.01419198	0.84795123	-0.0893968	0.22623632	0.42327345	1.94E-09
5.82E-14	-0.1625319	0.02707685	0.09351984	0.20545483	0.51915383	3.71E-14
2.97E-07	0.03733601	0.61386886	-0.0969342	0.18931597	0.443712	2.52E-10
1.92E-08	-0.1185342	0.10805827	-0.0304836	0.68040773	0.49554205	7.42E-13
1.11E-05	0.00922298	0.90084146	-0.1105571	0.13409599	0.46762202	1.93E-11
6.45E-07	-0.0237341	0.74845583	0.02305065	0.75546685	0.65221073	8.63E-24
1.59E-07	-0.0029455	0.96825896	-0.0655003	0.3757178	0.44197924	3.01E-10
1.76E-07	0.18218557	0.01306458	-0.0250198	0.73532377	0.35989052	4.87E-07
4.39E-10	-0.1381032	0.0608385	0.03790172	0.60850463	0.56298852	7.36E-17
0.02267911	0.12750574	0.08370577	-0.2248009	0.00209537	0.4045053	1.13E-08
0.00025749	0.0452842	0.54049075	-0.1050005	0.15490539	0.20418453	0.00530643
2.59E-14	-0.062808	0.39569782	0.06876073	0.35236624	0.48549794	2.48E-12
2.20E-05	0.09686778	0.18962103	-0.1231402	0.09493964	0.37803251	1.13E-07
4.41E-06	0.05506667	0.45658842	-0.1124258	0.12760204	0.39441239	2.77E-08
3.17E-12	-0.1485516	0.04358748	0.07806787	0.29085231	0.52874229	1.03E-14
0.00614258	0.08524352	0.24863144	-0.1243299	0.09176565	0.06298351	0.39437617
6.33E-11	-0.0073672	0.92072031	-0.0066693	0.92820946	0.4431373	2.67E-10
0.01007948	0.05646488	0.44521931	-0.2917852	5.58E-05	0.12387011	0.0929822
0.14737722	-0.3430434	1.75E-06	-0.0372414	0.61476832	0.40707313	8.91E-09
7.34E-07	0.00863702	0.90711192	-0.1194886	0.10522927	0.27832462	0.00012503
0.00336335	0.01607562	0.82806489	-0.1789841	0.01478248	0.37660418	1.27E-07
2.30E-12	-0.0174363	0.81376702	0.03362537	0.64955308	0.46432519	2.79E-11
3.93E-08	-0.0852309	0.24870173	-0.0261942	0.72339498	0.49142285	1.22E-12
1.96E-06	-0.0391542	0.59670233	0.00789387	0.91507287	0.6119968	2.15E-20
2.30E-09	-0.1900901	0.00955156	0.04221466	0.5683087	0.59724381	2.88E-19
2.17E-07	-0.0489885	0.50784498	-0.0911629	0.21715962	0.39883439	1.88E-08
9.76E-14	-0.074909	0.3108734	0.04515452	0.54165215	0.46088173	4.07E-11
5.23E-06	-0.0614116	0.40630689	-0.1599879	0.02960368	0.14453103	0.04966608
9.39E-08	-0.0928087	0.20893761	-0.0605617	0.41284506	0.35330472	8.10E-07
3.82E-07	-0.048748	0.5099325	-0.0270109	0.71513835	0.56036941	1.10E-16
1.42E-06	0.26503892	0.00026645	-0.0860437	0.24420146	0.41868678	3.01E-09
5.48E-48	0.29894751	3.57E-05	-0.5707082	2.23E-17	-0.3221683	7.75E-06
1.05E-64	0.46769553	1.92E-11	-0.758512	7.15E-36	-0.4887963	1.68E-12
9.41E-34	0.3511735	9.53E-07	-0.6545603	5.27E-24	-0.3907244	3.83E-08
1.82E-60	0.3855637	5.97E-08	-0.761554	2.61E-36	-0.426188	1.46E-09
6.05E-35	0.31744679	1.07E-05	-0.5855787	2.05E-18	-0.6684822	2.58E-25
2.04E-69	0.41457302	4.44E-09	-0.8638263	2.21E-56	-0.5668058	4.09E-17
1.69E-74	0.45179845	1.08E-10	-0.8024821	6.68E-43	-0.6414848	7.78E-23
2.81E-68	0.388727	4.55E-08	-0.7973947	5.32E-42	-0.5919562	7.08E-19
1.05E-46	0.42342049	1.91E-09	-0.70831	1.73E-29	-0.5954789	3.90E-19
0.70611764	0.065556	0.37531121	0.03512098	0.63506806	-0.0083229	0.91047548
2.75E-61	0.49499383	7.94E-13	-0.8275106	9.45E-48	-0.6332129	4.00E-22
6.58E-43	0.37894998	1.04E-07	-0.6567988	3.28E-24	-0.4644431	2.75E-11
1.19E-52	0.40492286	1.08E-08	-0.6981824	2.31E-28	-0.6130427	1.78E-20

3.82E-47	0.41965171	2.75E-09	-0.6618877	1.10E-24	-0.3722614	1.81E-07
3.90E-63	0.40740528	8.64E-09	-0.6399543	1.06E-22	-0.4744725	8.93E-12
2.98E-34	0.34028554	2.15E-06	-0.5662063	4.49E-17	-0.4719395	1.19E-11
8.46E-77	0.48566374	2.43E-12	-0.7431171	9.32E-34	-0.5265239	1.38E-14
9.94E-49	0.5429119	1.42E-15	-0.624183	2.26E-21	-0.4562486	6.72E-11
0.6517778	0.01233175	0.86768526	0.07623095	0.30238753	-0.0073327	0.92109017
2.80E-56	0.52055034	3.08E-14	-0.8495106	1.04E-52	-0.6469811	2.55E-23
4.94E-40	0.32604654	5.93E-06	-0.5877233	1.44E-18	-0.4727148	1.09E-11
8.17E-53	0.39613298	2.38E-08	-0.7149767	2.95E-30	-0.6023706	1.19E-19
5.38E-47	-0.5972719	2.87E-19	0.83308442	6.14E-49	0.65167996	9.64E-24
2.81E-65	0.46717537	2.03E-11	-0.808463	5.38E-44	-0.6288371	9.33E-22
1.82E-44	0.39638187	2.33E-08	-0.6872244	3.39E-27	-0.415364	4.13E-09
0.00033719	0.09910045	0.17956941	-0.0570797	0.44027116	-0.169675	0.02094608
1.27E-58	0.50426734	2.52E-13	-0.8022851	7.24E-43	-0.7218448	4.52E-31
1.32E-38	0.45680841	6.33E-11	-0.5834833	2.89E-18	-0.7681317	2.82E-37
3.68E-31	0.32261695	7.51E-06	-0.5056322	2.12E-13	-0.3612913	4.36E-07
3.00E-71	0.52522974	1.65E-14	-0.7981018	4.00E-42	-0.5826321	3.32E-18
1.97E-60	0.2787679	0.00012183	-0.5612703	9.56E-17	-0.4917758	1.17E-12
1.82E-42	0.55952624	1.24E-16	-0.5965129	3.27E-19	-0.4938809	9.09E-13
2.10E-71	0.41755236	3.36E-09	-0.7975101	5.07E-42	-0.5873062	1.54E-18
3.29E-75	0.41448815	4.48E-09	-0.7322157	2.39E-32	-0.4760498	7.46E-12
1.09E-47	0.27449124	0.00015616	-0.6279414	1.11E-21	-0.6943993	5.92E-28
3.45E-57	0.431711	8.49E-10	-0.665836	4.63E-25	-0.4252902	1.60E-09
3.17E-56	0.32180412	7.94E-06	-0.759898	4.53E-36	-0.5330751	5.66E-15
1.15E-36	0.19752183	0.00703925	-0.4928631	1.03E-12	-0.4726828	1.09E-11
1.52E-52	0.40149401	1.48E-08	-0.7873921	2.66E-40	-0.7329514	1.93E-32
3.69E-44	0.46011293	4.43E-11	-0.7994112	2.35E-42	-0.6002586	1.71E-19
1.62E-45	0.32108727	8.34E-06	-0.7368297	6.17E-33	-0.7373933	5.22E-33
7.53E-39	0.22899701	0.00171646	-0.575936	9.77E-18	-0.6842467	6.90E-27
1.36E-53	0.33925103	2.31E-06	-0.698948	1.91E-28	-0.4752468	8.18E-12
2.10E-53	0.31047984	1.70E-05	-0.7511652	7.64E-35	-0.5957711	3.71E-19
1.10E-70	0.40188011	1.43E-08	-0.7094396	1.29E-29	-0.6338864	3.51E-22
7.12E-56	-0.3736871	1.61E-07	0.65493621	4.87E-24	0.52837659	1.08E-14
2.34E-62	0.39484732	2.67E-08	-0.7145596	3.30E-30	-0.6267105	1.40E-21
3.40E-34	0.35511756	7.05E-07	-0.6025083	1.16E-19	-0.3282589	5.08E-06
4.60E-49	0.31347741	1.39E-05	-0.6125795	1.93E-20	-0.3324978	3.76E-06
2.95E-40	-0.3437216	1.67E-06	0.53198413	6.58E-15	0.84145794	8.31E-51
6.28E-39	-0.5405208	2.00E-15	0.65299143	7.33E-24	0.62173204	3.59E-21
1.66E-39	0.36131633	4.35E-07	-0.5534925	3.06E-16	-0.3653477	3.16E-07
4.55E-28	0.1531294	0.03743783	-0.497853	5.59E-13	-0.6206025	4.43E-21
2.10E-32	0.2690298	0.00021315	-0.5571555	1.78E-16	-0.4032839	1.26E-08
3.36E-76	0.38047268	9.18E-08	-0.7676296	3.35E-37	-0.5429516	1.41E-15
3.47E-77	0.48095791	4.23E-12	-0.8862501	4.56E-63	-0.7019758	8.86E-29
1.35E-28	-0.3011813	3.10E-05	0.62653801	1.45E-21	0.57783255	7.21E-18
6.27E-45	0.47651668	7.07E-12	-0.7738512	3.83E-38	-0.6756395	5.12E-26
1.49E-45	0.41297813	5.16E-09	-0.6503745	1.27E-23	-0.6601394	1.60E-24

1.45E-41	0.27383004	0.00016221	-0.5432717	1.35E-15	-0.326355	5.80E-06
7.06E-101	0.49751891	5.83E-13	-0.7619346	2.30E-36	-0.6094561	3.39E-20
2.68E-44	-0.3021817	2.91E-05	0.64388897	4.79E-23	0.70501155	4.07E-29
2.97E-51	0.55248065	3.55E-16	-0.7967952	6.76E-42	-0.6159194	1.05E-20
1.46E-78	-0.472092	1.17E-11	0.70741385	2.18E-29	0.67457681	6.53E-26
3.06E-60	0.43551033	5.81E-10	-0.8104879	2.25E-44	-0.492932	1.02E-12
3.38E-60	-0.5571791	1.77E-16	0.81983041	3.49E-46	0.70479899	4.30E-29
4.67E-50	0.4093388	7.24E-09	-0.8191808	4.70E-46	-0.7573168	1.06E-35
3.11E-60	0.37846306	1.09E-07	-0.7343183	1.29E-32	-0.6417721	7.35E-23
3.01E-51	0.46014335	4.41E-11	-0.6733657	8.60E-26	-0.7038835	5.44E-29
1.67E-41	0.4667888	2.12E-11	-0.6409577	8.65E-23	-0.6355754	2.52E-22
4.64E-37	0.30472031	2.47E-05	-0.6411815	8.27E-23	-0.6221823	3.30E-21
1.00E-34	0.33738733	2.65E-06	-0.7013264	1.05E-28	-0.6736917	7.98E-26
2.15E-35	-0.3337053	3.45E-06	0.64152067	7.73E-23	0.69011811	1.69E-27
1.07E-35	0.55545971	2.29E-16	-0.7210545	5.63E-31	-0.5503236	4.88E-16
5.76E-36	0.2614363	0.00032497	-0.5528475	3.37E-16	-0.3005222	3.23E-05
1.22E-92	0.56736791	3.75E-17	-0.8691142	7.60E-58	-0.7172223	1.61E-30
6.14E-41	-0.2144114	0.00338194	0.61014464	3.00E-20	0.71446527	3.38E-30
7.73E-30	0.23029965	0.00161223	-0.6802568	1.76E-26	-0.2504662	0.00058475
3.61E-23	-0.3914434	3.60E-08	0.49430017	8.64E-13	0.70389975	5.42E-29
2.78E-40	0.31957095	9.25E-06	-0.5597338	1.21E-16	-0.5298686	8.79E-15
4.45E-90	0.46953584	1.56E-11	-0.7453291	4.73E-34	-0.738815	3.42E-33
1.68E-43	0.62683936	1.37E-21	-0.8336263	4.68E-49	-0.628236	1.05E-21
3.08E-70	0.43470777	6.30E-10	-0.8047648	2.58E-43	-0.6545263	5.31E-24
1.71E-17	0.09168775	0.2145127	-0.4202598	2.59E-09	-0.2663866	0.0002472
4.86E-19	-0.125615	0.08843277	0.40998678	6.81E-09	0.41751495	3.37E-09
2.65E-27	0.311685	1.57E-05	-0.6369974	1.90E-22	-0.6702236	1.75E-25
6.29E-71	0.39618658	2.37E-08	-0.6874237	3.23E-27	-0.5062809	1.95E-13
4.70E-45	0.47694856	6.73E-12	-0.6752937	5.54E-26	-0.574676	1.19E-17
9.81E-28	0.38134909	8.53E-08	-0.5671679	3.87E-17	-0.4922967	1.10E-12
1.38E-25	0.25036912	0.00058773	-0.5520854	3.77E-16	-0.4098908	6.88E-09
3.17E-66	0.48309366	3.29E-12	-0.8253116	2.70E-47	-0.7800495	4.11E-39
1.74E-44	0.34194277	1.90E-06	-0.6694694	2.07E-25	-0.5036577	2.72E-13
3.53E-50	0.73588816	8.15E-33	-0.7736796	4.07E-38	-0.665519	4.96E-25
6.24E-38	0.37619503	1.31E-07	-0.5219306	2.56E-14	-0.2644273	0.00027564
4.46E-60	0.39328211	3.06E-08	-0.7511249	7.73E-35	-0.5713703	2.01E-17
1.33E-52	0.53536385	4.12E-15	-0.8205439	2.51E-46	-0.8145523	3.78E-45
3.26E-69	0.39306753	3.12E-08	-0.6931128	8.13E-28	-0.5780578	6.96E-18
6.02E-33	0.34346873	1.70E-06	-0.5445934	1.12E-15	-0.4933109	9.74E-13
1.47E-41	0.48532536	2.53E-12	-0.7533771	3.78E-35	-0.7666948	4.62E-37
1.11E-26	-0.2025434	0.00569345	0.52154642	2.70E-14	0.71069598	9.23E-30
1.47E-31	0.34331428	1.72E-06	-0.5738842	1.35E-17	-0.6159903	1.04E-20
3.13E-27	0.251256	0.00056101	-0.5665198	4.28E-17	-0.7124101	5.86E-30
5.45E-53	-0.3594143	5.05E-07	0.53219251	6.39E-15	0.63801578	1.56E-22
0.17941528	0.06859143	0.35355586	-0.0211967	0.77458368	-0.0609897	0.40954466
6.20E-44	0.57035723	2.36E-17	-0.6965641	3.46E-28	-0.5638024	6.50E-17

1.26E-47	0.36745126	2.67E-07	-0.7599326	4.48E-36	-0.5980724	2.50E-19
7.55E-43	0.33569932	2.99E-06	-0.7448542	5.47E-34	-0.5398258	2.20E-15
5.14E-34	0.34909587	1.12E-06	-0.5819638	3.70E-18	-0.528184	1.11E-14
8.85E-69	-0.5515389	4.08E-16	0.87396958	3.01E-59	0.74628282	3.52E-34
6.69E-42	0.3450612	1.51E-06	-0.5899412	9.93E-19	-0.4798059	4.84E-12
1.85E-41	0.42478887	1.68E-09	-0.8122522	1.04E-44	-0.3914145	3.61E-08
1.65E-26	0.30481758	2.46E-05	-0.6382968	1.47E-22	-0.3779171	1.14E-07
2.43E-104	0.49372957	9.26E-13	-0.8594707	3.20E-55	-0.6229629	2.85E-21
9.41E-52	0.31876106	9.77E-06	-0.6906288	1.49E-27	-0.4756177	7.84E-12
3.66E-54	0.42081759	2.46E-09	-0.6776985	3.19E-26	-0.5961339	3.48E-19
1.55E-31	0.3230683	7.28E-06	-0.680069	1.84E-26	-0.6348233	2.92E-22
1.86E-36	0.31993836	9.02E-06	-0.5838794	2.71E-18	-0.3093461	1.83E-05
5.42E-47	0.36105474	4.44E-07	-0.6712178	1.40E-25	-0.5731242	1.53E-17
2.38E-46	0.32904206	4.81E-06	-0.5642512	6.07E-17	-0.4296778	1.04E-09
1.62E-28	0.33828481	2.48E-06	-0.5820757	3.63E-18	-0.6709367	1.49E-25
1.64E-57	0.39115789	3.69E-08	-0.8263077	1.68E-47	-0.7137494	4.10E-30
2.14E-37	0.43362747	7.02E-10	-0.715932	2.28E-30	-0.6788682	2.43E-26
8.21E-63	0.43250188	7.85E-10	-0.8082632	5.87E-44	-0.7026116	7.54E-29
3.92E-44	0.45019127	1.28E-10	-0.7822425	1.83E-39	-0.4204388	2.55E-09
3.10E-58	0.47428207	9.13E-12	-0.778827	6.42E-39	-0.4573578	5.97E-11
4.66E-34	0.56562342	4.91E-17	-0.6638278	7.19E-25	-0.7666285	4.72E-37
9.32E-55	0.45643055	6.59E-11	-0.7736864	4.06E-38	-0.6635337	7.67E-25
6.53E-42	0.45412956	8.44E-11	-0.7642091	1.07E-36	-0.5610654	9.86E-17
6.23E-57	-0.446248	1.94E-10	0.69516378	4.90E-28	0.47252711	1.11E-11
3.39E-82	0.37959286	9.88E-08	-0.7706224	1.19E-37	-0.5844199	2.48E-18
9.40E-62	0.39569711	2.48E-08	-0.725754	1.52E-31	-0.5746469	1.20E-17
3.93E-63	-0.5281511	1.11E-14	0.78894809	1.47E-40	0.64077841	8.97E-23
2.14E-27	0.23385775	0.0013563	-0.530034	8.59E-15	-0.2824596	9.80E-05
0.00010853	0.09293233	0.20832895	-0.2993736	3.48E-05	-0.1729604	0.0185555
2.22E-48	0.27924114	0.0001185	-0.6435969	5.08E-23	-0.4347287	6.28E-10
1.31E-44	0.23778325	0.00111745	-0.5309981	7.53E-15	-0.3719344	1.86E-07
2.77E-49	0.5483658	6.49E-16	-0.8167471	1.42E-45	-0.6319439	5.12E-22
1.30E-45	0.3131701	1.42E-05	-0.5980487	2.51E-19	-0.5649359	5.46E-17
9.87E-48	0.40727209	8.75E-09	-0.6311662	5.96E-22	-0.4760598	7.45E-12
3.18E-57	0.56978005	2.58E-17	-0.8385619	3.78E-50	-0.6964702	3.55E-28
7.30E-40	0.20908414	0.00428726	-0.6719594	1.18E-25	-0.5566207	1.92E-16
1.57E-36	0.35952538	5.01E-07	-0.7732709	4.70E-38	-0.5341553	4.87E-15
1.79E-44	0.53079039	7.75E-15	-0.8337764	4.34E-49	-0.6888051	2.32E-27
2.16E-45	-0.401519	1.48E-08	0.68895531	2.24E-27	0.60124471	1.44E-19
4.00E-43	0.19733069	0.00709566	-0.545476	9.85E-16	-0.2941391	4.83E-05
2.15E-28	0.11986779	0.10412146	-0.4747934	8.61E-12	-0.3560627	6.55E-07
3.92E-53	0.51816211	4.22E-14	-0.7044985	4.65E-29	-0.6329248	4.23E-22
1.69E-73	0.49968942	4.46E-13	-0.8599033	2.46E-55	-0.6297812	7.78E-22
6.49E-63	0.36839187	2.48E-07	-0.6837283	7.80E-27	-0.5140022	7.27E-14
2.33E-44	0.2813157	0.00010488	-0.6292961	8.55E-22	-0.4308653	9.24E-10
1.22E-37	0.44433721	2.36E-10	-0.7763745	1.56E-38	-0.664962	5.61E-25

4.13E-55	0.40027288	1.65E-08	-0.8830319	5.02E-62	-0.692053	1.05E-27
5.29E-60	0.43999046	3.69E-10	-0.7800599	4.09E-39	-0.8236599	5.90E-47
8.94E-47	0.30774602	2.03E-05	-0.6200606	4.90E-21	-0.4003053	1.65E-08
9.94E-42	0.31296872	1.44E-05	-0.6007263	1.58E-19	-0.4541072	8.46E-11
8.22E-53	0.6021744	1.23E-19	-0.7913607	5.77E-41	-0.51838	4.10E-14
1.63E-56	0.43968251	3.81E-10	-0.6507577	1.17E-23	-0.4571551	6.10E-11
5.05E-84	0.44138277	3.20E-10	-0.8669752	3.02E-57	-0.6545642	5.26E-24
5.53E-56	0.3552648	6.97E-07	-0.556703	1.90E-16	-0.4139538	4.71E-09
8.21E-48	0.31955614	9.26E-06	-0.5439027	1.23E-15	-0.4276723	1.27E-09
5.18E-45	0.52885899	1.01E-14	-0.7572712	1.07E-35	-0.7462968	3.51E-34
2.82E-53	0.33546334	3.04E-06	-0.6047382	7.83E-20	-0.6437443	4.93E-23
2.61E-43	0.5032835	2.85E-13	-0.6171682	8.36E-21	-0.4332047	7.32E-10
6.75E-61	0.42511766	1.62E-09	-0.818136	7.56E-46	-0.6962817	3.72E-28
6.84E-69	0.45140228	1.13E-10	-0.807191	9.27E-44	-0.6205023	4.51E-21
1.08E-37	0.30468166	2.48E-05	-0.5885132	1.26E-18	-0.4042842	1.15E-08
1.01E-51	0.33132491	4.09E-06	-0.7117834	6.92E-30	-0.5110516	1.06E-13
1.12E-95	0.51340798	7.85E-14	-0.8588254	4.71E-55	-0.7656405	6.61E-37
1.15E-27	-0.4367187	5.14E-10	0.46847848	1.76E-11	0.67635752	4.34E-26
9.76E-15	0.21794071	0.00288127	-0.3717457	1.89E-07	-0.2907584	5.94E-05
4.75E-15	0.16520507	0.02462302	-0.3926283	3.24E-08	-0.0871954	0.23792216
2.40E-49	0.39290623	3.17E-08	-0.6989424	1.91E-28	-0.4394832	3.89E-10
6.71E-59	-0.4969843	6.22E-13	0.81425941	4.31E-45	0.53173153	6.81E-15
4.19E-15	0.33114076	4.14E-06	-0.3744477	1.51E-07	-0.1981398	0.0068596
1.79E-83	-0.4505016	1.24E-10	0.85504796	4.40E-54	0.72382542	2.61E-31
5.96E-71	0.49264138	1.06E-12	-0.8015553	9.79E-43	-0.5606644	1.05E-16
7.32E-48	0.3957911	2.46E-08	-0.752554	4.91E-35	-0.6180007	7.17E-21
1.83E-55	0.41344644	4.94E-09	-0.8185253	6.34E-46	-0.6714626	1.32E-25
2.04E-35	0.48304852	3.31E-12	-0.6904171	1.57E-27	-0.7012605	1.06E-28
4.98E-68	-0.5107087	1.11E-13	0.80418437	3.29E-43	0.58278873	3.24E-18
0.00175995	-0.0331288	0.65439184	-0.2028202	0.00562647	0.11767031	0.11067015
3.02E-53	0.37336029	1.66E-07	-0.5511362	4.33E-16	-0.4615595	3.78E-11
1.73E-45	0.41600111	3.89E-09	-0.7151286	2.83E-30	-0.5696727	2.62E-17
1.04E-78	0.41369024	4.83E-09	-0.7398523	2.50E-33	-0.6434711	5.21E-23
3.85E-67	0.43318131	7.34E-10	-0.7222281	4.07E-31	-0.5770344	8.20E-18
2.25E-36	0.42898142	1.11E-09	-0.7910623	6.48E-41	-0.6107397	2.69E-20
1.62E-38	0.27897887	0.00012034	-0.6293926	8.39E-22	-0.5291072	9.75E-15
1.27E-40	0.45718354	6.08E-11	-0.8163702	1.68E-45	-0.8464485	5.67E-52
2.45E-57	0.37485625	1.46E-07	-0.7648357	8.69E-37	-0.4821053	3.70E-12
9.46E-40	0.3097132	1.79E-05	-0.576907	8.37E-18	-0.5340847	4.92E-15
3.05E-32	-0.3534351	8.02E-07	0.5642488	6.07E-17	0.75153683	6.79E-35
1.84E-36	0.50121866	3.69E-13	-0.6439499	4.73E-23	-0.6621169	1.04E-24
1.29E-56	0.48855148	1.73E-12	-0.6779434	3.01E-26	-0.4729363	1.06E-11
3.26E-87	0.45663641	6.45E-11	-0.8459641	7.39E-52	-0.6461626	3.02E-23
2.79E-43	0.28910411	6.58E-05	-0.6219153	3.47E-21	-0.450652	1.22E-10
4.97E-51	0.43251847	7.84E-10	-0.8128004	8.20E-45	-0.7200026	7.52E-31
1.29E-57	-0.3592468	5.12E-07	0.7746487	2.88E-38	0.66639093	4.10E-25

5.94E-47	0.42918361	1.09E-09	-0.8331051	6.08E-49	-0.6769643	3.78E-26
1.22E-28	0.18691183	0.01084867	-0.4693454	1.60E-11	-0.314131	1.33E-05
5.33E-53	0.32935249	4.70E-06	-0.66218	1.03E-24	-0.5442155	1.18E-15
6.25E-43	-0.5225841	2.35E-14	0.8432374	3.23E-51	0.69186991	1.10E-27
8.06E-53	0.29522244	4.51E-05	-0.6679982	2.87E-25	-0.376281	1.30E-07
6.58E-47	0.41449139	4.48E-09	-0.7887854	1.56E-40	-0.7169348	1.74E-30
6.53E-51	0.36692709	2.79E-07	-0.6438625	4.82E-23	-0.4893199	1.58E-12
3.68E-08	0.36771065	2.62E-07	-0.2775409	0.00013088	-0.0545857	0.46053651
1.05E-67	-0.5287809	1.02E-14	0.83632109	1.20E-49	0.73875063	3.48E-33
1.52E-25	0.26907829	0.00021256	-0.6479769	2.08E-23	-0.2886018	6.78E-05
2.46E-98	0.52834703	1.08E-14	-0.7885725	1.69E-40	-0.6406223	9.26E-23
8.93E-47	0.52159976	2.68E-14	-0.5858114	1.97E-18	-0.4955394	7.43E-13
2.31E-35	-0.5149895	6.39E-14	0.69826718	2.26E-28	0.78146637	2.44E-39
1.07E-21	0.03411677	0.64477904	-0.5106477	1.12E-13	-0.420954	2.43E-09
8.70E-54	0.50368264	2.71E-13	-0.7731437	4.92E-38	-0.498754	5.00E-13
2.80E-72	0.34532429	1.48E-06	-0.8151591	2.89E-45	-0.5513007	4.23E-16
9.90E-67	0.45335465	9.16E-11	-0.7930721	2.96E-41	-0.6178532	7.37E-21
2.03E-63	0.33189824	3.93E-06	-0.7195281	8.57E-31	-0.7503364	9.92E-35
6.37E-60	0.22425346	0.00215004	-0.5791034	5.88E-18	-0.5132735	7.99E-14
3.98E-46	0.24830963	0.00065436	-0.5549499	2.47E-16	-0.4350897	6.06E-10
2.89E-91	-0.3732837	1.67E-07	0.73415674	1.36E-32	0.61601576	1.03E-20
3.26E-33	0.51965829	3.47E-14	-0.74005	2.36E-33	-0.7134007	4.50E-30
1.01E-45	0.39161774	3.54E-08	-0.6154134	1.15E-20	-0.5661914	4.50E-17
5.72E-54	0.46510379	2.56E-11	-0.6015498	1.37E-19	-0.5075997	1.65E-13
8.71E-22	-0.216131	0.00312894	0.42489612	1.66E-09	0.64057838	9.34E-23
7.20E-53	0.54389376	1.24E-15	-0.7112206	8.04E-30	-0.4465626	1.87E-10
9.26E-74	0.37175115	1.89E-07	-0.6988339	1.96E-28	-0.4286432	1.15E-09
2.75E-40	0.44319297	2.66E-10	-0.5838779	2.71E-18	-0.3120179	1.54E-05
5.48E-52	0.33392479	3.40E-06	-0.6765189	4.18E-26	-0.5535109	3.05E-16
1.86E-37	0.4701208	1.46E-11	-0.6771653	3.61E-26	-0.6613281	1.24E-24
1.55E-64	0.48323137	3.24E-12	-0.7977492	4.61E-42	-0.7596377	4.94E-36
1.48E-30	0.3767212	1.26E-07	-0.5747276	1.18E-17	-0.4754047	8.03E-12
2.59E-65	0.30322045	2.72E-05	-0.7199452	7.64E-31	-0.5545198	2.63E-16
7.36E-64	0.47197532	1.19E-11	-0.7915775	5.31E-41	-0.5869169	1.64E-18
2.27E-63	0.43777507	4.62E-10	-0.7391154	3.12E-33	-0.6111701	2.49E-20
1.28E-45	0.33687734	2.75E-06	-0.5490866	5.84E-16	-0.5104607	1.15E-13
4.55E-24	0.15167953	0.03930097	-0.384756	6.39E-08	-0.1320045	0.07327208
8.93E-33	-0.3629049	3.84E-07	0.62523588	1.85E-21	0.60720558	5.06E-20
1.20E-38	-0.4210617	2.40E-09	0.72282074	3.45E-31	0.85149302	3.41E-53
3.45E-24	-0.4201605	2.62E-09	0.66268972	9.22E-25	0.57451173	1.23E-17
6.15E-30	0.12943353	0.07909729	-0.4459185	2.00E-10	-0.4123911	5.45E-09
3.20E-46	0.31629774	1.15E-05	-0.603166	1.03E-19	-0.5339531	5.01E-15
4.06E-58	0.52603689	1.48E-14	-0.6551661	4.63E-24	-0.470339	1.43E-11
2.26E-49	0.42562414	1.55E-09	-0.6665248	3.98E-25	-0.4107615	6.34E-09
3.44E-41	0.4366015	5.21E-10	-0.503242	2.86E-13	-0.342908	1.77E-06
6.65E-58	0.52712836	1.28E-14	-0.7953865	1.19E-41	-0.5790806	5.90E-18

4.29E-57	0.39725324	2.16E-08	-0.6042617	8.52E-20	-0.3647811	3.31E-07
4.85E-60	-0.4252145	1.61E-09	0.74503469	5.18E-34	0.80589157	1.61E-43
1.79E-44	-0.4418366	3.06E-10	0.62787817	1.12E-21	0.74654914	3.24E-34
5.77E-52	0.52701662	1.30E-14	-0.8596688	2.84E-55	-0.7585145	7.14E-36
3.30E-73	-0.5527461	3.42E-16	0.72175611	4.63E-31	0.58043355	4.74E-18
3.79E-77	-0.5393459	2.36E-15	0.69276889	8.85E-28	0.59850217	2.32E-19
2.30E-46	-0.315441	1.22E-05	0.55331109	3.14E-16	0.66370773	7.39E-25
2.72E-59	-0.4938485	9.13E-13	0.84255896	4.63E-51	0.86097096	1.29E-55
4.56E-63	0.50840948	1.49E-13	-0.8773511	2.94E-60	-0.6186684	6.34E-21
3.03E-74	0.4470254	1.79E-10	-0.7950095	1.38E-41	-0.628009	1.09E-21
1.69E-35	0.28817168	6.96E-05	-0.4640597	2.87E-11	-0.3029351	2.77E-05
1.35E-35	0.28299244	9.50E-05	-0.5471788	7.71E-16	-0.4069175	9.04E-09
1.62E-52	0.3281896	5.10E-06	-0.6775119	3.33E-26	-0.3580076	5.64E-07
4.70E-51	0.37182842	1.88E-07	-0.6509383	1.13E-23	-0.5420585	1.61E-15
1.91E-75	0.38317512	7.31E-08	-0.7234656	2.88E-31	-0.5508233	4.53E-16
3.36E-42	0.28927852	6.51E-05	-0.527962	1.14E-14	-0.3357726	2.98E-06
1.30E-44	-0.3854817	6.01E-08	0.54999834	5.12E-16	0.79406541	2.00E-41
1.31E-43	0.30385353	2.61E-05	-0.5341777	4.86E-15	-0.3960141	2.41E-08
6.23E-26	0.33499952	3.15E-06	-0.5461735	8.91E-16	-0.2144544	0.00337538
7.15E-42	0.47843325	5.67E-12	-0.690733	1.45E-27	-0.5001319	4.22E-13
4.50E-49	0.34683957	1.32E-06	-0.6613925	1.22E-24	-0.4909357	1.30E-12
1.05E-94	0.47196692	1.19E-11	-0.8318872	1.11E-48	-0.6722534	1.11E-25
8.28E-17	0.24839195	0.00065157	-0.5925894	6.36E-19	-0.4463522	1.92E-10
7.89E-39	0.57600389	9.66E-18	-0.7237419	2.67E-31	-0.7532267	3.96E-35
1.28E-51	0.29212722	5.46E-05	-0.6818915	1.20E-26	-0.4067341	9.19E-09
2.17E-51	0.46222332	3.51E-11	-0.6874887	3.18E-27	-0.7047457	4.36E-29
1.10E-61	0.3608003	4.53E-07	-0.694932	5.19E-28	-0.5034555	2.79E-13
1.93E-37	0.28139314	0.00010441	-0.5916889	7.40E-19	-0.6129175	1.82E-20
1.56E-21	0.18156003	0.01338578	-0.4092826	7.27E-09	-0.095143	0.19766301
6.94E-17	0.20235717	0.00573893	-0.4545407	8.07E-11	-0.2081534	0.00446612
8.87E-66	0.51534742	6.10E-14	-0.8530909	1.37E-53	-0.6698776	1.89E-25
7.22E-43	0.32741806	5.38E-06	-0.5132992	7.96E-14	-0.39863	1.91E-08
1.05E-70	0.33051194	4.33E-06	-0.7391753	3.07E-33	-0.5349832	4.34E-15
6.08E-39	0.35170435	9.15E-07	-0.5956304	3.80E-19	-0.543219	1.36E-15
4.21E-31	0.51023712	1.18E-13	-0.66181	1.12E-24	-0.5659803	4.65E-17
1.01E-74	0.45354355	8.98E-11	-0.790945	6.78E-41	-0.6787238	2.52E-26
3.45E-45	0.25883215	0.00037447	-0.5365623	3.49E-15	-0.4636319	3.01E-11
2.39E-62	0.59788757	2.58E-19	-0.8251823	2.87E-47	-0.8505794	5.71E-53
2.10E-32	0.26179703	0.00031861	-0.4311497	8.98E-10	-0.1431207	0.05195889
1.59E-74	0.37249581	1.78E-07	-0.7473738	2.51E-34	-0.5143837	6.92E-14
2.11E-60	0.49756835	5.79E-13	-0.8077117	7.42E-44	-0.6563715	3.59E-24
1.79E-52	-0.5067948	1.83E-13	0.83503458	2.30E-49	0.83734461	7.09E-50
7.08E-52	-0.3334506	3.52E-06	0.56320958	7.11E-17	0.54316485	1.37E-15
3.29E-55	0.37619795	1.31E-07	-0.7025826	7.59E-29	-0.4078063	8.33E-09
3.10E-53	0.59798416	2.54E-19	-0.6683915	2.63E-25	-0.6254983	1.76E-21
1.09E-66	0.34673177	1.33E-06	-0.6539871	5.94E-24	-0.4900656	1.44E-12

1.04E-46	0.29945814	3.46E-05	-0.7784133	7.46E-39	-0.5955809	3.83E-19
2.05E-46	-0.4890146	1.63E-12	0.75881968	6.46E-36	0.73650283	6.80E-33
1.50E-28	-0.2070006	0.00469693	0.45828916	5.39E-11	0.7854451	5.55E-40
8.58E-20	0.29517994	4.52E-05	-0.5047963	2.36E-13	-0.1517843	0.03916382
9.78E-52	0.46203502	3.59E-11	-0.671454	1.32E-25	-0.4595449	4.71E-11
5.08E-63	-0.4798887	4.79E-12	0.77604874	1.75E-38	0.67113663	1.42E-25
3.22E-63	0.4186776	3.02E-09	-0.8664714	4.17E-57	-0.6378299	1.61E-22
1.46E-42	0.37606902	1.32E-07	-0.7660222	5.81E-37	-0.6487167	1.78E-23
2.96E-44	0.24378095	0.0008261	-0.7340447	1.40E-32	-0.5344445	4.68E-15
2.10E-45	0.29986356	3.37E-05	-0.6538625	6.10E-24	-0.424249	1.77E-09
1.48E-55	0.50853335	1.47E-13	-0.667179	3.44E-25	-0.4723203	1.14E-11
2.91E-52	0.31829942	1.01E-05	-0.7646286	9.32E-37	-0.5804272	4.75E-18
2.52E-50	0.32419367	6.74E-06	-0.738384	3.89E-33	-0.3540605	7.65E-07
1.82E-30	0.26117622	0.00032962	-0.6114601	2.37E-20	-0.3467671	1.33E-06
1.16E-54	0.43124229	8.90E-10	-0.6136104	1.60E-20	-0.4190578	2.91E-09
3.78E-67	0.48289816	3.37E-12	-0.8904655	1.76E-64	-0.7600226	4.35E-36
4.76E-49	0.36645984	2.89E-07	-0.6440932	4.60E-23	-0.3408881	2.05E-06
9.56E-47	0.43407068	6.71E-10	-0.854644	5.57E-54	-0.6103811	2.87E-20
2.51E-37	0.42109531	2.39E-09	-0.8447489	1.43E-51	-0.6122748	2.04E-20
4.63E-55	0.31297016	1.44E-05	-0.6420858	6.90E-23	-0.5622456	8.24E-17
5.18E-29	0.19864056	0.00671705	-0.5146938	6.64E-14	-0.5254612	1.60E-14
5.09E-74	0.4788193	5.42E-12	-0.75605	1.60E-35	-0.5540105	2.84E-16
1.27E-51	0.2641551	0.00027982	-0.605463	6.89E-20	-0.4015092	1.48E-08
4.61E-16	0.28394071	8.97E-05	-0.5005867	3.99E-13	-0.2549447	0.00046148
5.48E-25	0.46637338	2.22E-11	-0.5870913	1.60E-18	-0.6421136	6.86E-23
1.49E-36	0.35185727	9.05E-07	-0.664896	5.69E-25	-0.4759646	7.53E-12
2.88E-38	0.16665611	0.02337288	-0.4739126	9.52E-12	-0.291562	5.66E-05
9.96E-50	0.38074051	8.98E-08	-0.8295905	3.45E-48	-0.7249799	1.88E-31
7.97E-53	0.43534225	5.91E-10	-0.8437485	2.45E-51	-0.6383768	1.45E-22
9.62E-59	0.31940128	9.36E-06	-0.6263167	1.51E-21	-0.6086192	3.94E-20
5.30E-44	-0.5009643	3.81E-13	0.79837788	3.58E-42	0.60969125	3.25E-20
0.00292374	0.0404027	0.58504227	-0.0079519	0.91445131	-0.0352755	0.63357894
7.80E-54	-0.5246554	1.78E-14	0.88314869	4.61E-62	0.73236307	2.29E-32
5.74E-68	-0.4528017	9.72E-11	0.72528653	1.73E-31	0.67045493	1.66E-25
1.19E-34	0.36965068	2.24E-07	-0.5924638	6.50E-19	-0.6077846	4.57E-20
1.46E-57	0.45214109	1.04E-10	-0.8359398	1.45E-49	-0.7427478	1.04E-33
3.60E-14	0.1512268	0.03989849	-0.324017	6.82E-06	-0.1370289	0.06289327
1.13E-37	0.49425133	8.69E-13	-0.6809999	1.48E-26	-0.6214619	3.77E-21
9.67E-46	0.33024207	4.42E-06	-0.7369152	6.02E-33	-0.45149	1.12E-10
3.11E-13	0.19227218	0.00874233	-0.4018661	1.43E-08	-0.1650272	0.02478013
0.71636211	0.03361079	0.649695	0.0399516	0.58924319	0.01065804	0.88551107
1.50E-58	0.46119838	3.93E-11	-0.7435859	8.08E-34	-0.6611614	1.28E-24
6.18E-59	0.44678513	1.83E-10	-0.8305886	2.11E-48	-0.760566	3.63E-36
5.00E-54	0.22335708	0.00224239	-0.6510401	1.10E-23	-0.4641597	2.84E-11
5.54E-47	0.53487429	4.41E-15	-0.8243302	4.30E-47	-0.7093563	1.31E-29
1.63E-29	0.28393795	8.98E-05	-0.5590966	1.33E-16	-0.2248203	0.00209345

3.49E-24	0.41795855	3.23E-09	-0.5164999	5.25E-14	-0.4179009	3.25E-09
1.77E-36	0.33037146	4.38E-06	-0.6441746	4.52E-23	-0.6305572	6.70E-22
3.49E-51	0.37282439	1.73E-07	-0.6219497	3.44E-21	-0.4634444	3.07E-11
4.66E-91	0.42755124	1.28E-09	-0.8492885	1.18E-52	-0.6331946	4.02E-22
2.07E-43	0.3235059	7.07E-06	-0.6690473	2.27E-25	-0.4769021	6.76E-12
5.60E-60	0.30151493	3.04E-05	-0.6075856	4.73E-20	-0.5524605	3.57E-16
2.95E-56	-0.3734092	1.65E-07	0.756367	1.44E-35	0.62650215	1.46E-21
4.64E-46	0.41246272	5.41E-09	-0.7208706	5.92E-31	-0.5558466	2.16E-16
1.74E-62	0.44887897	1.47E-10	-0.7808334	3.08E-39	-0.6170514	8.54E-21
1.52E-54	-0.5096417	1.27E-13	0.83343782	5.14E-49	0.67417782	7.15E-26
1.11E-59	0.49747968	5.86E-13	-0.5878431	1.41E-18	-0.5882072	1.33E-18
6.26E-59	-0.3106644	1.68E-05	0.5976629	2.68E-19	0.7687313	2.29E-37
4.30E-55	0.46500289	2.59E-11	-0.8015051	9.99E-43	-0.5854576	2.09E-18
5.32E-45	0.31821299	1.01E-05	-0.5432387	1.36E-15	-0.3247032	6.50E-06
6.15E-43	0.4981104	5.42E-13	-0.6464902	2.82E-23	-0.3382258	2.49E-06
9.29E-55	0.48660835	2.18E-12	-0.772887	5.38E-38	-0.5864922	1.76E-18
9.01E-59	0.32765427	5.30E-06	-0.6338628	3.53E-22	-0.3942371	2.82E-08
0.02794541	-0.0479738	0.51668458	0.10050048	0.17347007	0.03407422	0.64519184
8.13E-16	0.42936533	1.07E-09	-0.3984655	1.94E-08	-0.4044829	1.13E-08
1.62E-49	0.40961437	7.05E-09	-0.6776236	3.24E-26	-0.4113569	6.00E-09
1.15E-50	0.51284108	8.45E-14	-0.7697181	1.63E-37	-0.6155696	1.12E-20
7.07E-48	0.44375955	2.51E-10	-0.7576156	9.59E-36	-0.6817861	1.23E-26
2.96E-44	0.29993886	3.36E-05	-0.7614925	2.67E-36	-0.682816	9.67E-27
1.45E-46	0.30836648	1.95E-05	-0.7102623	1.04E-29	-0.5159023	5.68E-14
9.60E-49	-0.5441123	1.20E-15	0.78141748	2.49E-39	0.69527691	4.77E-28
9.38E-32	0.32380276	6.92E-06	-0.5255517	1.58E-14	-0.3592065	5.13E-07
1.16E-37	0.46862689	1.73E-11	-0.5585191	1.45E-16	-0.393156	3.10E-08
5.19E-50	0.38246345	7.77E-08	-0.6777611	3.14E-26	-0.3962847	2.35E-08
2.88E-32	0.23070896	0.0015807	-0.632877	4.27E-22	-0.6701201	1.79E-25
1.62E-54	0.41800133	3.22E-09	-0.8475187	3.15E-52	-0.8039207	3.67E-43
3.23E-42	0.48196981	3.76E-12	-0.8320689	1.02E-48	-0.7872768	2.77E-40
5.84E-16	0.19855201	0.00674206	-0.4227577	2.04E-09	-0.2504776	0.0005844
2.03E-36	0.21011385	0.0040969	-0.504728	2.38E-13	-0.2859543	7.96E-05
9.81E-65	0.49872737	5.02E-13	-0.6858308	4.73E-27	-0.5773939	7.74E-18
1.71E-62	0.36873385	2.41E-07	-0.6913588	1.25E-27	-0.3775688	1.17E-07
3.19E-45	0.42180116	2.24E-09	-0.6967959	3.27E-28	-0.564429	5.90E-17
1.93E-55	0.58631998	1.81E-18	-0.6985033	2.13E-28	-0.5068322	1.82E-13
4.74E-42	-0.2332537	0.00139694	0.54783578	7.01E-16	0.25805555	0.00039053
1.26E-45	0.37249811	1.78E-07	-0.665781	4.69E-25	-0.5541188	2.79E-16
2.31E-37	0.45510075	7.60E-11	-0.6249077	1.97E-21	-0.267863	0.0002276
2.16E-58	0.27899481	0.00012023	-0.6840296	7.26E-27	-0.5323689	6.24E-15
7.65E-70	0.44397594	2.45E-10	-0.776091	1.72E-38	-0.5216533	2.66E-14
3.32E-51	0.22940559	0.00168313	-0.5559246	2.13E-16	-0.3773952	1.19E-07
2.58E-65	0.48884987	1.67E-12	-0.8230675	7.79E-47	-0.5808099	4.46E-18
3.22E-49	0.29919152	3.52E-05	-0.7288667	6.26E-32	-0.3991689	1.82E-08
9.05E-40	0.38842757	4.67E-08	-0.7576351	9.53E-36	-0.7157138	2.42E-30

8.92E-36	0.26890788	0.00021462	-0.6791416	2.28E-26	-0.4611749	3.94E-11
2.30E-60	0.40178315	1.44E-08	-0.6705199	1.63E-25	-0.676821	3.90E-26
0.37295037	0.03503204	0.63592565	0.04667934	0.52807424	-0.0146243	0.84337822
1.63E-35	0.36867596	2.42E-07	-0.6689549	2.32E-25	-0.7798357	4.44E-39
2.30E-40	0.56970973	2.61E-17	-0.7745784	2.96E-38	-0.7920742	4.37E-41
3.18E-30	0.40791523	8.25E-09	-0.6136459	1.59E-20	-0.6577037	2.70E-24
2.23E-62	-0.4719276	1.19E-11	0.87014411	3.87E-58	0.77607868	1.73E-38
9.02E-43	0.37212573	1.83E-07	-0.7580016	8.45E-36	-0.7454483	4.56E-34
3.47E-42	0.38651486	5.50E-08	-0.7703797	1.30E-37	-0.6335647	3.74E-22
1.61E-64	0.41527491	4.16E-09	-0.7920728	4.37E-41	-0.6173794	8.04E-21
1.07E-42	0.34670883	1.33E-06	-0.6335794	3.73E-22	-0.5697801	2.58E-17
2.12E-35	0.30568998	2.32E-05	-0.5506486	4.65E-16	-0.4780062	5.95E-12
2.86E-24	0.24901671	0.00063074	-0.5208726	2.95E-14	-0.1976013	0.00701591
4.70E-57	0.4931621	9.92E-13	-0.8771206	3.45E-60	-0.8020602	7.95E-43
1.91E-37	0.32329856	7.17E-06	-0.6564964	3.50E-24	-0.4888741	1.66E-12
1.22E-53	0.23645805	0.00119339	-0.606412	5.83E-20	-0.4380431	4.50E-10
3.78E-41	0.40015148	1.67E-08	-0.7980756	4.04E-42	-0.6880638	2.77E-27
7.63E-50	0.35629676	6.44E-07	-0.6961422	3.85E-28	-0.6293568	8.45E-22
1.01E-68	0.37150133	1.93E-07	-0.7267461	1.15E-31	-0.5738332	1.36E-17
5.35E-64	0.34330119	1.72E-06	-0.7329939	1.90E-32	-0.5906705	8.79E-19
5.81E-36	0.38397839	6.83E-08	-0.5665149	4.28E-17	-0.2328771	0.00142285
2.72E-50	0.39415805	2.84E-08	-0.7184746	1.14E-30	-0.795441	1.16E-41
4.51E-61	0.39718039	2.17E-08	-0.7248954	1.93E-31	-0.6152709	1.18E-20
8.54E-49	0.42665742	1.40E-09	-0.7555589	1.87E-35	-0.4433068	2.63E-10
2.87E-58	-0.3736265	1.62E-07	0.7091296	1.39E-29	0.54698909	7.92E-16
5.08E-53	0.34609212	1.40E-06	-0.5832042	3.02E-18	-0.4852878	2.54E-12
1.03E-52	-0.5129582	8.32E-14	0.69845632	2.16E-28	0.46282329	3.29E-11
1.02E-34	0.3119076	1.55E-05	-0.7292842	5.56E-32	-0.7757523	1.95E-38
7.10E-22	0.26150027	0.00032383	-0.5163739	5.34E-14	-0.2175991	0.00292661
1.99E-57	0.26582924	0.000255	-0.6278447	1.13E-21	-0.5142486	7.04E-14
1.16E-42	0.50554963	2.14E-13	-0.7309191	3.47E-32	-0.6594731	1.85E-24
7.48E-29	0.22461299	0.00211399	-0.4514269	1.12E-10	-0.3516933	9.16E-07
7.69E-37	0.45878281	5.11E-11	-0.6543199	5.54E-24	-0.771615	8.41E-38
5.97E-36	0.33572288	2.99E-06	-0.5275036	1.21E-14	-0.4403658	3.55E-10
1.57E-44	0.41339781	4.96E-09	-0.5835559	2.85E-18	-0.2665744	0.00024462
5.49E-35	0.2872677	7.35E-05	-0.6840576	7.21E-27	-0.612532	1.95E-20
5.49E-51	0.36536099	3.16E-07	-0.61447	1.37E-20	-0.3573974	5.91E-07
1.11E-28	0.36661386	2.86E-07	-0.464952	2.60E-11	-0.3338479	3.42E-06
6.15E-63	0.53981296	2.21E-15	-0.763534	1.35E-36	-0.544289	1.17E-15
2.45E-56	-0.3466755	1.34E-06	0.71728741	1.58E-30	0.70940363	1.30E-29
5.34E-29	0.24133482	0.00093525	-0.4286249	1.15E-09	-0.2503002	0.00058985
6.09E-87	0.53001612	8.62E-15	-0.7793641	5.28E-39	-0.6485722	1.84E-23
1.08E-45	0.39639312	2.33E-08	-0.6175183	7.84E-21	-0.3853405	6.08E-08
3.09E-26	0.62681937	1.37E-21	-0.5385293	2.65E-15	-0.5316553	6.88E-15
3.34E-55	0.50034991	4.11E-13	-0.7178321	1.36E-30	-0.4187661	2.99E-09
2.87E-11	0.2294401	0.00168034	-0.4311483	8.98E-10	-0.0552521	0.45507159

1.32E-46	-0.4593874	4.79E-11	0.59572858	3.73E-19	0.78134531	2.55E-39
0.23975588	0.03635137	0.62325434	0.06179893	0.40334722	-0.0339071	0.64681377
3.35E-32	0.35676201	6.21E-07	-0.5908988	8.46E-19	-0.3244435	6.62E-06
1.05E-47	-0.5734211	1.46E-17	0.84861152	1.72E-52	0.80019453	1.71E-42
9.06E-34	0.34978467	1.06E-06	-0.703435	6.11E-29	-0.7039101	5.41E-29
8.25E-34	0.33355522	3.49E-06	-0.6446643	4.09E-23	-0.7366612	6.49E-33
1.87E-41	0.39054609	3.89E-08	-0.687046	3.54E-27	-0.3903665	3.95E-08
9.28E-27	0.26943887	0.00020828	-0.5073548	1.71E-13	-0.4525194	1.00E-10
2.84E-57	0.61288428	1.83E-20	-0.8454884	9.58E-52	-0.6507127	1.18E-23
3.72E-22	0.29477016	4.64E-05	-0.487817	1.89E-12	-0.4554329	7.34E-11
3.72E-41	0.41638582	3.75E-09	-0.6383818	1.45E-22	-0.5601772	1.13E-16
5.65E-43	0.53656853	3.48E-15	-0.7509145	8.27E-35	-0.6663741	4.11E-25
6.91E-47	0.39436804	2.79E-08	-0.771529	8.67E-38	-0.6217522	3.57E-21
3.66E-64	0.50105482	3.76E-13	-0.6874487	3.22E-27	-0.7322687	2.35E-32
5.68E-38	0.25039209	0.00058702	-0.6068039	5.44E-20	-0.5677264	3.55E-17
4.23E-75	-0.511356	1.02E-13	0.85143939	3.51E-53	0.8310788	1.66E-48
1.54E-39	0.40125501	1.51E-08	-0.7270795	1.04E-31	-0.6833981	8.43E-27
1.06E-10	0.38803869	4.83E-08	-0.3632328	3.74E-07	-0.0270509	0.714734
8.47E-34	0.16583575	0.02407277	-0.5379073	2.89E-15	-0.3626952	3.90E-07
1.48E-61	0.40733437	8.70E-09	-0.8668752	3.22E-57	-0.823724	5.72E-47
1.38E-56	0.39896832	1.85E-08	-0.5987083	2.24E-19	-0.5584956	1.45E-16
2.43E-25	0.32110387	8.33E-06	-0.5102452	1.18E-13	-0.260935	0.000334
1.19E-58	0.45363248	8.90E-11	-0.8290788	4.42E-48	-0.7194595	8.73E-31
5.44E-64	0.40427383	1.15E-08	-0.7921767	4.20E-41	-0.5647366	5.63E-17
2.35E-42	0.28262103	9.71E-05	-0.5412879	1.79E-15	-0.4386234	4.24E-10
3.63E-44	0.31236048	1.50E-05	-0.619815	5.13E-21	-0.3882129	4.76E-08
2.92E-55	0.51055956	1.13E-13	-0.870306	3.48E-58	-0.5082243	1.53E-13
5.53E-70	0.32176228	7.96E-06	-0.6918721	1.10E-27	-0.4960421	6.98E-13
2.57E-60	0.3572949	5.96E-07	-0.7156266	2.48E-30	-0.5597774	1.20E-16
3.15E-79	0.41947018	2.80E-09	-0.7185866	1.11E-30	-0.6400108	1.05E-22
1.55E-49	0.49613531	6.90E-13	-0.7904484	8.22E-41	-0.6613485	1.23E-24
2.98E-59	0.57230796	1.74E-17	-0.8428213	4.03E-51	-0.6685787	2.52E-25
9.47E-77	-0.3623948	4.00E-07	0.68632505	4.21E-27	0.54265904	1.48E-15
1.35E-17	0.10659764	0.14868957	-0.4131299	5.09E-09	-0.1557944	0.03420742
5.69E-73	0.44040946	3.54E-10	-0.7145481	3.31E-30	-0.5190836	3.74E-14
3.69E-76	0.41925031	2.86E-09	-0.7828639	1.46E-39	-0.5968632	3.08E-19
0.99579105	0.0186417	0.80115094	0.08626187	0.24300308	0.06173389	0.40384325
1.01E-55	0.46752235	1.96E-11	-0.7201183	7.28E-31	-0.7185741	1.11E-30
3.47E-62	0.35388214	7.75E-07	-0.6143174	1.41E-20	-0.4642997	2.80E-11
6.72E-62	0.48756795	1.94E-12	-0.8500745	7.59E-53	-0.7355968	8.88E-33
7.61E-44	0.47678967	6.85E-12	-0.7883104	1.87E-40	-0.6449118	3.89E-23
4.05E-57	0.28141455	0.00010427	-0.7226176	3.65E-31	-0.5358283	3.86E-15
2.70E-31	0.3560461	6.56E-07	-0.5067495	1.84E-13	-0.3259463	5.97E-06
7.34E-30	-0.2381089	0.00109948	0.67083205	1.52E-25	0.8535036	1.08E-53
3.31E-45	0.36482627	3.30E-07	-0.6592941	1.92E-24	-0.5451906	1.03E-15
1.24E-70	0.45247179	1.01E-10	-0.7805713	3.39E-39	-0.4836202	3.10E-12

1.43E-60	0.38813278	4.79E-08	-0.7227325	3.53E-31	-0.47146	1.26E-11
1.53E-56	0.32252401	7.56E-06	-0.6683273	2.67E-25	-0.4557992	7.06E-11
5.38E-39	-0.4519071	1.07E-10	0.67386276	7.68E-26	0.83102933	1.70E-48
3.97E-66	0.41615021	3.83E-09	-0.7120666	6.42E-30	-0.5032909	2.85E-13
4.65E-48	0.4241806	1.78E-09	-0.8304104	2.31E-48	-0.5486169	6.26E-16
9.69E-67	0.62638427	1.49E-21	-0.877127	3.44E-60	-0.5829592	3.15E-18
3.27E-57	0.48410887	2.92E-12	-0.7809553	2.95E-39	-0.5551324	2.40E-16
3.82E-45	0.295133	4.54E-05	-0.6800346	1.86E-26	-0.6584235	2.32E-24
1.58E-44	0.33410529	3.36E-06	-0.5636201	6.68E-17	-0.4124505	5.42E-09
5.61E-28	-0.3828006	7.55E-08	0.65620116	3.72E-24	0.69150431	1.21E-27
3.33E-62	0.4852888	2.54E-12	-0.7897133	1.09E-40	-0.6173676	8.06E-21
3.15E-77	0.54407655	1.20E-15	-0.8096581	3.22E-44	-0.6141206	1.46E-20
9.13E-77	0.4537214	8.81E-11	-0.8320093	1.05E-48	-0.6311017	6.03E-22
2.54E-69	0.4560584	6.86E-11	-0.8931792	2.01E-65	-0.5705092	2.30E-17
2.54E-41	-0.3383275	2.47E-06	0.67933632	2.18E-26	0.85874612	4.94E-55
3.82E-74	0.43189038	8.34E-10	-0.7929907	3.05E-41	-0.6239837	2.35E-21
6.26E-78	0.37082042	2.04E-07	-0.7364885	6.83E-33	-0.5912328	7.99E-19
4.85E-58	0.4303317	9.74E-10	-0.7838262	1.02E-39	-0.5779121	7.12E-18
7.01E-49	0.68288422	9.52E-27	-0.8239775	5.08E-47	-0.5478862	6.96E-16
2.98E-69	0.36035037	4.69E-07	-0.754625	2.53E-35	-0.5976862	2.67E-19
3.61E-52	0.61164667	2.29E-20	-0.7768571	1.31E-38	-0.692148	1.03E-27
2.49E-11	-0.0718541	0.33107306	-0.0028775	0.96899206	-0.0080937	0.9129315
5.58E-48	0.3841311	6.74E-08	-0.6932291	7.90E-28	-0.6207773	4.29E-21
3.47E-54	0.44932127	1.40E-10	-0.8229723	8.14E-47	-0.6970967	3.03E-28
9.26E-85	-0.4966254	6.50E-13	0.77814924	8.21E-39	0.58363104	2.82E-18
3.76E-59	0.3278436	5.23E-06	-0.7611167	3.02E-36	-0.6037048	9.40E-20
9.41E-54	0.42000038	2.66E-09	-0.6394872	1.16E-22	-0.500107	4.23E-13
1.21E-57	0.42484593	1.67E-09	-0.7293382	5.47E-32	-0.6363274	2.17E-22
2.13E-32	0.32722917	5.46E-06	-0.659524	1.83E-24	-0.5030426	2.94E-13
2.23E-52	0.30782459	2.02E-05	-0.6307232	6.49E-22	-0.5078214	1.61E-13
6.48E-41	-0.4910228	1.28E-12	0.75850499	7.16E-36	0.62797669	1.10E-21
2.62E-92	0.5112829	1.03E-13	-0.8631427	3.38E-56	-0.6485339	1.85E-23
2.74E-12	0.24396682	0.0008183	-0.4022934	1.38E-08	-0.2685001	0.0002196
6.21E-61	0.35229423	8.75E-07	-0.6365296	2.09E-22	-0.5462101	8.87E-16
1.43E-32	0.29929935	3.49E-05	-0.5607774	1.03E-16	-0.1590383	0.03059708
1.33E-71	0.53437667	4.73E-15	-0.8471746	3.81E-52	-0.6259578	1.62E-21
1.11E-70	0.51125538	1.04E-13	-0.8375448	6.40E-50	-0.5585609	1.44E-16
3.81E-46	0.40937768	7.21E-09	-0.7378124	4.61E-33	-0.6793759	2.16E-26
2.06E-56	0.4589434	5.03E-11	-0.6768567	3.87E-26	-0.4148961	4.31E-09
3.47E-57	0.35114542	9.55E-07	-0.6755069	5.28E-26	-0.5377608	2.95E-15
1.14E-49	0.39430729	2.80E-08	-0.7313809	3.04E-32	-0.671525	1.30E-25
4.97E-56	0.54252769	1.50E-15	-0.8786913	1.15E-60	-0.7284718	7.01E-32
2.44E-61	0.44853057	1.53E-10	-0.7425786	1.10E-33	-0.5089499	1.39E-13
2.25E-48	0.40283023	1.31E-08	-0.709516	1.26E-29	-0.4755063	7.94E-12
2.26E-55	0.5116241	9.88E-14	-0.6910361	1.35E-27	-0.3718318	1.88E-07
2.69E-60	0.38658634	5.47E-08	-0.6157014	1.09E-20	-0.4958696	7.13E-13

3.70E-76	0.61596769	1.04E-20	-0.8532713	1.23E-53	-0.6768386	3.89E-26
1.27E-56	0.41617788	3.82E-09	-0.6825765	1.02E-26	-0.6501117	1.34E-23
3.19E-44	0.34564364	1.44E-06	-0.6522803	8.51E-24	-0.4970462	6.18E-13
1.39E-21	0.28446253	8.70E-05	-0.5419061	1.64E-15	-0.5151812	6.24E-14
1.33E-49	0.42124905	2.36E-09	-0.7775432	1.02E-38	-0.4358352	5.62E-10
1.17E-63	0.41041856	6.55E-09	-0.6606328	1.44E-24	-0.4555844	7.22E-11
1.48E-12	0.28680191	7.56E-05	-0.4810744	4.17E-12	-0.1691425	0.02135763
9.43E-63	0.43336235	7.21E-10	-0.799833	1.98E-42	-0.6569468	3.18E-24
1.60E-48	0.43442065	6.48E-10	-0.7504827	9.48E-35	-0.774634	2.90E-38
2.58E-62	0.41878771	2.98E-09	-0.799966	1.88E-42	-0.6708233	1.53E-25
3.51E-32	0.18483274	0.01177898	-0.5054497	2.17E-13	-0.214687	0.00334018
1.15E-47	0.40356003	1.23E-08	-0.6856053	5.00E-27	-0.5791403	5.85E-18
4.24E-54	0.51260122	8.71E-14	-0.6360464	2.30E-22	-0.4541257	8.44E-11
2.85E-72	0.46133922	3.87E-11	-0.7236555	2.73E-31	-0.5723535	1.72E-17
2.29E-31	0.41738829	3.41E-09	-0.7996387	2.14E-42	-0.51844	4.07E-14
1.16E-34	0.31730259	1.08E-05	-0.6183993	6.66E-21	-0.3415728	1.95E-06
3.80E-43	0.38751802	5.05E-08	-0.6707065	1.57E-25	-0.5844926	2.45E-18
3.99E-31	-0.3787403	1.06E-07	0.73119817	3.21E-32	0.57090103	2.17E-17
5.69E-52	0.58240247	3.45E-18	-0.7446634	5.80E-34	-0.7039954	5.29E-29
1.71E-47	0.5606148	1.06E-16	-0.7944247	1.74E-41	-0.6914923	1.21E-27
5.76E-40	0.53158104	6.95E-15	-0.7369497	5.95E-33	-0.5165596	5.21E-14
5.97E-63	0.35043035	1.01E-06	-0.667609	3.13E-25	-0.5385201	2.65E-15
4.83E-35	0.36582147	3.05E-07	-0.5837992	2.74E-18	-0.5583332	1.49E-16
5.29E-67	-0.5348371	4.43E-15	0.87209829	1.06E-58	0.61142329	2.38E-20
8.42E-65	0.44501759	2.20E-10	-0.7949049	1.44E-41	-0.5592909	1.29E-16
1.54E-49	0.3697765	2.22E-07	-0.7656133	6.67E-37	-0.7323027	2.33E-32
4.53E-42	0.49444711	8.48E-13	-0.7500206	1.10E-34	-0.4993247	4.66E-13
1.79E-46	0.17686584	0.01602489	-0.5263103	1.43E-14	-0.5119116	9.52E-14
9.81E-24	0.25470765	0.00046735	-0.4554169	7.35E-11	-0.3649723	3.26E-07
1.09E-48	-0.4233324	1.93E-09	0.59148372	7.66E-19	0.65049417	1.23E-23
5.47E-45	0.45796907	5.58E-11	-0.8133782	6.36E-45	-0.6615138	1.19E-24
5.03E-49	0.26870129	0.00021713	-0.6240187	2.33E-21	-0.3691657	2.33E-07
5.15E-58	0.53831134	2.73E-15	-0.8164606	1.61E-45	-0.5951221	4.14E-19
9.44E-39	0.30105639	3.13E-05	-0.5542008	2.76E-16	-0.4294238	1.06E-09
3.01E-72	0.45884816	5.08E-11	-0.6432783	5.42E-23	-0.6281298	1.07E-21
5.98E-57	0.2682246	0.00022303	-0.6051491	7.29E-20	-0.3345834	3.24E-06
2.71E-21	0.36582104	3.05E-07	-0.5486763	6.20E-16	-0.5555547	2.26E-16
2.87E-36	0.37020394	2.14E-07	-0.5945403	4.57E-19	-0.4429178	2.73E-10
3.36E-33	0.31902281	9.60E-06	-0.7002311	1.38E-28	-0.6376588	1.67E-22
1.60E-37	0.33677336	2.77E-06	-0.6006249	1.61E-19	-0.5184023	4.09E-14
4.38E-30	0.34425244	1.60E-06	-0.5576703	1.64E-16	-0.5164557	5.28E-14
1.57E-67	-0.4874404	1.97E-12	0.85870766	5.06E-55	0.72515192	1.80E-31
8.13E-47	0.58517998	2.19E-18	-0.8417824	7.00E-51	-0.7267056	1.16E-31
1.46E-27	0.07666938	0.29960732	-0.4489851	1.45E-10	-0.1587344	0.03092086
1.68E-44	0.38372257	6.98E-08	-0.7325987	2.14E-32	-0.6258459	1.65E-21
2.37E-50	0.40408684	1.17E-08	-0.8535946	1.02E-53	-0.7750507	2.50E-38

3.00E-50	-0.5642156	6.10E-17	0.84735349	3.45E-52	0.6309606	6.20E-22
6.51E-31	-0.4261482	1.47E-09	0.64029506	9.88E-23	0.68640682	4.13E-27
8.54E-49	0.39389823	2.90E-08	-0.5971947	2.91E-19	-0.4504842	1.24E-10
2.00E-73	0.44275838	2.78E-10	-0.7836271	1.10E-39	-0.442428	2.88E-10
2.28E-47	0.37384345	1.59E-07	-0.696373	3.63E-28	-0.4386258	4.24E-10
4.33E-65	0.36089322	4.50E-07	-0.7344074	1.26E-32	-0.5216155	2.67E-14
7.24E-71	0.48989295	1.47E-12	-0.8601718	2.09E-55	-0.5766218	8.76E-18
9.02E-37	0.29298002	5.19E-05	-0.4738067	9.64E-12	-0.3549365	7.15E-07
3.78E-67	0.50904423	1.38E-13	-0.7361545	7.53E-33	-0.7105455	9.61E-30
8.85E-51	0.4302214	9.84E-10	-0.7706417	1.18E-37	-0.6839369	7.42E-27
1.59E-33	0.2209416	0.00250944	-0.6848124	6.03E-27	-0.3393865	2.29E-06
1.89E-44	-0.3750333	1.44E-07	0.51707871	4.87E-14	0.27971874	0.00011523
2.93E-51	0.44003594	3.67E-10	-0.5920264	7.00E-19	-0.3343335	3.30E-06
1.72E-49	0.48437804	2.83E-12	-0.8401698	1.64E-50	-0.5637862	6.51E-17
3.76E-44	-0.5081073	1.55E-13	0.81487656	3.27E-45	0.76414003	1.10E-36
2.27E-43	0.49368214	9.31E-13	-0.5728527	1.59E-17	-0.4808217	4.30E-12
3.25E-51	0.71090965	8.73E-30	-0.8589389	4.40E-55	-0.7210127	5.69E-31
3.79E-38	-0.539996	2.15E-15	0.67441213	6.78E-26	0.85312753	1.34E-53
7.21E-49	-0.3164432	1.14E-05	0.56552189	4.99E-17	0.31734209	1.08E-05
1.11E-38	0.40441994	1.14E-08	-0.7193019	9.11E-31	-0.7482869	1.89E-34
2.13E-20	0.42039449	2.56E-09	-0.5196503	3.47E-14	-0.3421216	1.88E-06
4.16E-41	-0.4894603	1.55E-12	0.72814472	7.70E-32	0.66529644	5.21E-25
7.59E-67	-0.3391724	2.33E-06	0.73615822	7.53E-33	0.49061761	1.35E-12
1.31E-51	-0.2674897	0.00023242	0.5075799	1.66E-13	0.52588866	1.51E-14
2.21E-39	0.45513156	7.58E-11	-0.7220695	4.25E-31	-0.7351443	1.01E-32
2.83E-43	0.37174867	1.89E-07	-0.8365859	1.05E-49	-0.6803459	1.73E-26
4.23E-71	0.46768311	1.92E-11	-0.7780357	8.55E-39	-0.5338831	5.06E-15
5.32E-38	0.40033904	1.64E-08	-0.583699	2.79E-18	-0.4872533	2.02E-12
1.16E-45	0.41208146	5.61E-09	-0.6519571	9.10E-24	-0.5410229	1.86E-15
1.38E-48	-0.3368402	2.76E-06	0.53415367	4.87E-15	0.72471273	2.03E-31
1.60E-45	0.34083934	2.06E-06	-0.7893874	1.24E-40	-0.6926277	9.16E-28
2.28E-18	0.33691522	2.74E-06	-0.5530578	3.26E-16	-0.371619	1.91E-07
4.13E-64	0.40929698	7.26E-09	-0.7169168	1.75E-30	-0.5891198	1.14E-18
2.12E-14	0.082546	0.26397525	-0.2984067	3.70E-05	-0.1050053	0.15488627
1.44E-47	0.36693675	2.79E-07	-0.7305247	3.89E-32	-0.6146367	1.33E-20
3.08E-64	0.45189046	1.07E-10	-0.7769019	1.29E-38	-0.5962587	3.41E-19
3.49E-78	0.54008049	2.13E-15	-0.8407764	1.19E-50	-0.6260115	1.60E-21
4.46E-48	0.41310982	5.10E-09	-0.656891	3.21E-24	-0.4455899	2.07E-10
8.43E-42	0.32612003	5.90E-06	-0.637525	1.72E-22	-0.4365543	5.23E-10
3.63E-72	0.38320858	7.29E-08	-0.7041623	5.07E-29	-0.549654	5.38E-16
4.25E-53	0.28260465	9.72E-05	-0.669198	2.20E-25	-0.6038587	9.14E-20
8.26E-34	0.26482955	0.00026956	-0.6969266	3.16E-28	-0.6622232	1.02E-24
8.44E-58	0.51052273	1.14E-13	-0.8550914	4.29E-54	-0.7320104	2.53E-32
1.39E-45	0.26587555	0.00025434	-0.5721157	1.79E-17	-0.4303169	9.75E-10
2.49E-91	0.54010229	2.12E-15	-0.7874311	2.62E-40	-0.6963248	3.68E-28
7.89E-40	0.19326688	0.00839405	-0.5613612	9.42E-17	-0.328894	4.86E-06

4.94E-50	0.17510133	0.01712835	-0.6030863	1.05E-19	-0.4703553	1.42E-11
4.32E-67	0.46142486	3.84E-11	-0.8209612	2.07E-46	-0.6204173	4.58E-21
1.98E-28	0.3361672	2.89E-06	-0.5621499	8.36E-17	-0.2743286	0.00015763
0.4517513	-0.0050692	0.94540226	-0.3094651	1.82E-05	-0.0106119	0.88600289
0.00011208	-0.105286	0.15378023	0.00044533	0.99519989	0.27932977	0.00011789
1.29E-07	0.18635805	0.01108991	-0.0426879	0.56397561	0.30382825	2.62E-05
0.95468055	0.00808333	0.9130425	-0.3231129	7.26E-06	0.03087431	0.67653997
0.00386529	-0.1242225	0.09204863	-0.0742506	0.31515703	0.21412779	0.00342539
4.46E-06	0.03080092	0.67726578	-0.1042416	0.15792662	0.42653693	1.41E-09
0.00047244	-0.1323482	0.0725205	-0.0352959	0.63338301	0.20550113	0.00501317
8.36E-05	-0.13952	0.05821316	0.03433501	0.64266343	0.31758423	1.06E-05
1.12E-07	-0.0830115	0.26128226	0.01237966	0.86717596	0.48819562	1.80E-12
3.90E-07	-0.2181239	0.00285722	0.09949378	0.17784009	0.3977676	2.06E-08
0.80291756	-0.0207305	0.77941259	-0.2990811	3.54E-05	0.03500756	0.63616181
0.00849091	-0.1083365	0.14213893	-0.0599813	0.41734599	0.23599164	0.00122122
0.0335781	-0.1161823	0.11528489	-0.0919862	0.21301806	0.17134105	0.01970245
0.95474756	-0.006315	0.93201287	-0.2745784	0.00015538	0.05231305	0.47944456
0.0372453	0.03466824	0.63943861	-0.2360099	0.00122012	0.20017426	0.0062967
0.02566166	-0.11704	0.11260697	-0.1374667	0.06204902	0.30316235	2.73E-05
0.85114225	-0.0292238	0.69293388	-0.2404185	0.00097944	-0.0123867	0.86710094
0.29931343	-0.0388507	0.59955271	-0.1825093	0.01290103	0.14377666	0.05088174
0.00031624	-0.1111114	0.13214394	-0.0386935	0.60103228	0.25416777	0.00048098
1.99E-06	-0.0645385	0.3827837	-0.0394282	0.59413454	0.50921178	1.35E-13
1.49E-07	-0.1316858	0.0739747	0.05928581	0.42277622	0.38752431	5.05E-08
1.70E-07	-0.0761214	0.30308516	-0.0298977	0.68622288	0.37322758	1.67E-07
0.00039887	-0.0975286	0.18660399	-0.0398094	0.59057029	0.25113489	0.00056459
2.17E-11	-0.1774471	0.01567522	0.12197969	0.09811931	0.56503282	5.38E-17
0.68677485	-0.016741	0.82106555	-0.3319959	3.90E-06	-0.0609655	0.409731
0.03181547	-0.1359087	0.06509608	-0.0988087	0.18085994	0.52545801	1.60E-14
0.00078703	-0.0786405	0.28731708	-0.0673753	0.36217544	0.24245326	0.0008838
0.00824129	-0.0945041	0.20070425	-0.1057115	0.15211463	0.16591714	0.02400254
3.66E-05	-0.107374	0.14573708	-0.0347837	0.63832251	0.32475068	6.48E-06
0.70950207	-0.0511715	0.48909857	-0.2531343	0.00050809	-0.0112628	0.87906249
0.55781002	0.00902631	0.90294542	-0.3220447	7.81E-06	-0.029882	0.68637868
0.89083259	-0.0532675	0.47145295	-0.2200658	0.00261319	0.06171501	0.40398732
0.65375729	-0.0271215	0.71402221	-0.2646329	0.00027252	0.11522718	0.11832492
0.00014797	-0.1956042	0.00762367	0.02759352	0.70926778	0.32719309	5.47E-06
0.47678452	-0.017581	0.81224986	-0.2374333	0.00113706	0.06037665	0.41427697
0.03767294	-0.0581726	0.43155297	-0.2085416	0.00439071	0.08716279	0.2380983
0.00014765	0.00099045	0.98932443	-0.0889566	0.22853977	0.22200148	0.0023889
8.68E-05	-0.1159743	0.11594146	-0.039233	0.5959632	0.27418164	0.00015896
0.53651222	-0.0226133	0.75996326	-0.279533	0.00011649	0.08646294	0.24190234
0.08531283	-0.0549742	0.45734597	-0.1832892	0.01251438	0.1262604	0.0867958
0.34687139	-0.0484959	0.51212619	-0.2117278	0.00381382	0.07120259	0.33548789
0.00050592	-0.0285873	0.69929411	-0.1976842	0.00699165	0.29122487	5.78E-05
0.0156824	-0.0792429	0.28362928	-0.1395082	0.05823459	0.16127728	0.02829892

3.52E-06	-0.1328927	0.07134243	0.04309017	0.56030546	0.33261815	3.73E-06
0.00687566	-0.117432	0.11139934	-0.1151184	0.11867496	0.22124758	0.00247408
1.09E-10	-0.1964708	0.00735443	0.09393864	0.20342362	0.47472222	8.68E-12
3.77E-07	-0.1574703	0.0322995	0.08779179	0.23471526	0.34731126	1.28E-06
0.00562087	-0.0740284	0.31661163	-0.1056765	0.15225099	0.16335017	0.02630442
0.09621653	-0.0528264	0.47513748	-0.1351062	0.06671281	0.31727959	1.08E-05
0.0009658	-0.010031	0.89220448	-0.1868485	0.01087602	0.15829534	0.03139392
4.99E-08	-0.2343606	0.00132329	0.12283449	0.09576925	0.39546614	2.53E-08
0.00010562	-0.116151	0.11538347	0.00547694	0.9410183	0.28196592	0.00010093
9.87E-06	-0.0804917	0.27608631	0.0180346	0.80749855	0.2858854	7.99E-05
0.84036748	-0.0539333	0.46592115	-0.2954583	4.45E-05	0.08083553	0.27403351
0.72029093	0.02736517	0.7115665	-0.3329568	3.64E-06	0.05593517	0.44950758
0.00087279	-0.1031196	0.16247424	-0.0416039	0.57392327	0.27207469	0.00017935
0.95573521	0.01559785	0.83309905	-0.3462076	1.39E-06	0.0036175	0.96102289
0.56626034	-0.0384084	0.60371741	-0.2535009	0.00049831	0.02724305	0.71279692
2.07E-07	-0.075239	0.30874075	-0.0219123	0.76718807	0.51378074	7.48E-14
0.75464712	-0.0971342	0.18840024	-0.1469459	0.04593596	0.13338386	0.07029322
0.00358736	-0.1276538	0.08334425	-0.0468059	0.52695506	0.28244539	9.81E-05
0.75718904	-0.0573118	0.43841165	-0.2175845	0.00292856	0.07204494	0.32978729
0.01798414	-0.038363	0.60414571	-0.1488614	0.04314618	0.09548642	0.19604248
0.15520861	-0.0313399	0.6719421	-0.1951023	0.00778358	0.16028309	0.0293005
0.00223127	-0.0688145	0.35198916	-0.0789737	0.28527333	0.24087599	0.00095715
2.59E-05	-0.1229428	0.09547476	0.00176345	0.98099386	0.28347884	9.23E-05
0.00023169	-0.1281517	0.08213819	-0.0239587	0.74615691	0.24073743	0.00096385
0.18849061	-0.0530987	0.47286059	-0.2007605	0.00614233	0.11454778	0.12052518
0.7714966	-0.0208614	0.77805593	-0.296116	4.27E-05	0.00642755	0.93080472
0.44315055	-0.056666	0.44359746	-0.2181996	0.00284733	0.03823484	0.60535571
0.00066152	-0.107065	0.14690701	-0.0434236	0.55727179	0.21042878	0.00404022
0.18510846	-0.0655953	0.37502403	-0.1974702	0.00705443	0.06618966	0.37070321
2.50E-05	-0.1321124	0.07303548	0.01767018	0.81131491	0.28543194	8.21E-05
0.10697007	-0.054609	0.46034468	-0.1970659	0.00717446	0.12909513	0.07989109
0.00032076	-0.1359351	0.06504352	0.01230953	0.86792153	0.26667837	0.00024321
4.25E-05	-0.1239985	0.09264133	0.00320484	0.96546625	0.28281573	9.60E-05
0.84117634	-0.0310841	0.67446684	-0.2645974	0.00027306	0.02797171	0.70546648
0.61892754	-0.0846027	0.25221923	-0.1772459	0.01579551	0.10010528	0.17517603
1.22E-05	-0.160907	0.02866851	-0.063862	0.38780131	0.33586646	2.96E-06
8.70E-05	-0.1244846	0.09135923	-0.0009343	0.98992964	0.30730056	2.09E-05
0.00043584	-0.086242	0.24311186	-0.0565938	0.44417944	0.20947918	0.00421331
0.0181031	-0.2605707	0.00034071	0.08063109	0.27525284	0.33063498	4.29E-06
2.09E-05	-0.1245619	0.09115676	0.01675492	0.82091929	0.38196959	8.10E-08
0.00146212	-0.0834179	0.25894625	-0.0846334	0.25204624	0.20986872	0.00414152
0.00451509	-0.0580292	0.4326912	-0.121323	0.0999556	0.08571139	0.24603441
0.05007469	-0.0465552	0.52917286	-0.15071	0.04058996	0.13835662	0.06036182
0.00012497	-0.1081053	0.14299695	-0.0338271	0.64759152	0.26570255	0.0002568
0.00143106	-0.0932334	0.20685269	-0.030019	0.68501661	0.26454065	0.00027392
0.05076047	-0.0700053	0.34369808	-0.152025	0.03885002	0.15708569	0.03272916

0.45276746	-0.0557122	0.45131945	-0.2385834	0.00107377	0.08144148	0.27044097
0.0011655	-0.0745149	0.31343309	-0.098604	0.18176962	0.23626075	0.00120509
6.72E-07	-0.2426682	0.00087421	0.04917875	0.50619631	0.34850855	1.17E-06
0.00095356	-0.0964789	0.19141292	-0.0039781	0.95714096	0.26506142	0.00026612
0.00819965	-0.1022914	0.16589347	-0.1091965	0.1389814	0.18675892	0.01091481
0.06876438	-0.0913757	0.21608338	-0.1389933	0.05917802	0.15811615	0.03158874
0.50672231	-0.012263	0.86841665	-0.296375	4.20E-05	0.06461999	0.38218171
0.64632827	-0.0286156	0.69901174	-0.2430371	0.00085798	0.05096243	0.49087761
0.47138412	-0.0105546	0.88661474	-0.2955045	4.43E-05	0.07792808	0.29171966
0.00040448	-0.113496	0.12399396	-0.038482	0.60302365	0.24029273	0.00098566
0.66306481	-0.0433194	0.55821875	-0.2108408	0.00396714	0.07292677	0.32388656
0.00021744	-0.1510695	0.04010787	0.01796611	0.80821551	0.24213955	0.00089796
0.12186694	-0.0888308	0.2292012	-0.1595844	0.03002236	0.10076182	0.17234875
0.01106	-0.1053456	0.15354609	-0.0604878	0.41341679	0.2030113	0.00558061
3.03E-06	-0.0930082	0.2079561	0.01924808	0.79482381	0.37091344	2.02E-07
0.24529878	-0.0421606	0.56880436	-0.2121589	0.00374126	0.11053523	0.13417347
3.77E-05	-0.1088074	0.14040341	0.00671282	0.92774179	0.28529274	8.28E-05
0.00046729	-0.0709489	0.33721695	-0.0519891	0.48217365	0.2401326	0.00099362
3.17E-06	-0.0525972	0.47705799	-0.0296275	0.68890993	0.4283722	1.18E-09
0.00038644	-0.0867518	0.2403272	-0.0328563	0.6570534	0.22904539	0.00171248
0.00014592	-0.1110138	0.13248593	-0.0131305	0.85920064	0.26912332	0.00021203
8.11E-06	-0.1296639	0.07856053	0.06129523	0.4071982	0.31814458	1.02E-05
0.00023516	-0.131338	0.07474772	-0.0077424	0.91669617	0.23820637	0.00109415
0.04790939	-0.0645134	0.38296867	-0.1729357	0.01857257	0.13631766	0.06428474
0.00652514	-0.0682643	0.3558618	-0.1098736	0.13653361	0.13894059	0.05927525
6.51E-05	-0.1156329	0.11702597	-0.0023199	0.97499773	0.30115613	3.11E-05
0.74353009	0.01421586	0.84769854	-0.3313176	4.09E-06	0.03157718	0.6696041
0.00030849	-0.0938934	0.20364248	0.00076557	0.99174816	0.30976155	1.78E-05
1.20E-07	-0.1222601	0.0973434	0.00218907	0.97640775	0.48499299	2.63E-12
7.62E-09	-0.1180705	0.10945407	-0.0025194	0.97284868	0.27185011	0.00018166
0.00029474	-0.1142098	0.12163143	-0.0026048	0.97192871	0.28378479	9.06E-05
0.14927874	-0.0299157	0.68604336	-0.2011142	0.00605086	0.12446703	0.09140534
5.11E-05	-0.144181	0.05022712	0.00587461	0.93674438	0.28295625	9.52E-05
0.33644191	-0.0159282	0.82961724	-0.3243766	6.65E-06	-0.0392825	0.59549985
0.0246838	-0.075364	0.30793505	-0.1176436	0.11075182	0.15136195	0.03971933
0.00108962	-0.0864621	0.24190709	-0.047028	0.52499352	0.22086575	0.00251827
0.00038729	-0.1171021	0.11241498	-0.0405801	0.58339391	0.21621185	0.00311747
7.26E-05	-0.1016219	0.16869654	-0.0179312	0.80858061	0.31038571	1.71E-05
1.54E-05	-0.1024968	0.16504053	-0.0005014	0.99459519	0.2602989	0.00034579
0.00024616	-0.10962	0.13744645	-0.0282946	0.70222719	0.26811359	0.00022442
0.57605725	-0.0679466	0.35810978	-0.1282245	0.08196302	0.09839518	0.18270122
0.23010852	-0.0660015	0.37206752	-0.1623175	0.02728249	0.23303039	0.00141225
0.00087577	-0.0788407	0.2860882	-0.0605525	0.4129159	0.22458651	0.00211662
0.57318851	-0.0578148	0.4343964	-0.2521042	0.00053651	0.01085235	0.88343847
0.00022311	-0.1178959	0.10998345	-0.0123463	0.86753028	0.26230066	0.00030993
0.00112139	-0.0942555	0.20189637	-0.0510812	0.48986651	0.21173363	0.00381283

0.00258543	-0.0376732	0.61066918	-0.0882397	0.23232697	0.24991039	0.000602
0.78243074	0.03210263	0.6644371	-0.2774306	0.00013172	0.10668351	0.14836081
0.00019389	-0.0942261	0.20203771	-0.027729	0.70790508	0.26239741	0.00030829
0.97149396	-0.012925	0.8613819	-0.2681256	0.00022427	0.0470593	0.52471782
0.52896119	-0.0257124	0.7282802	-0.2166772	0.00305223	0.07619764	0.30259946
0.61097464	-0.0112543	0.87915357	-0.332025	3.89E-06	0.06547304	0.37591707
0.00595138	-0.0829532	0.26161848	-0.1491746	0.04270375	0.38999357	4.08E-08
0.00038308	0.13501797	0.06689251	-0.0784613	0.28842019	0.41474188	4.37E-09
0.00039128	-0.1196933	0.10463014	-0.0235351	0.75049543	0.25392676	0.00048718
0.78610286	-0.0174643	0.81347254	-0.3160529	1.17E-05	0.02819088	0.70326689
0.00084066	-0.1323169	0.07258868	-0.0366543	0.62035983	0.21285288	0.00362704
0.3626714	-0.0267222	0.71805254	-0.2753075	0.00014898	0.09000995	0.22305506
0.92389773	0.00512324	0.94482111	-0.3444309	1.58E-06	-0.0820448	0.26689626
0.8917061	-0.0116193	0.87526523	-0.2672312	0.0002358	0.09076204	0.21919645
2.20E-05	-0.143281	0.05169386	0.02015992	0.7853347	0.30034614	3.27E-05
0.20028686	-0.0500255	0.49889333	-0.2104648	0.00403378	0.09754337	0.18653705
0.00015078	-0.1168169	0.11329871	-0.0326335	0.65923313	0.25270474	0.00051977
0.69155596	-0.000238	0.99743478	-0.3262092	5.86E-06	0.0023778	0.97437441
0.01804504	-0.0930194	0.20790116	-0.0679134	0.35834555	0.21569039	0.00319207
1.79E-05	-0.1307804	0.07600063	0.02941318	0.69104531	0.29252939	5.33E-05
8.49E-07	-0.163612	0.02606127	0.06523517	0.37765757	0.33476851	3.20E-06
0.00013522	-0.0807417	0.27459241	-0.0114899	0.87664407	0.26036687	0.00034451
0.00107561	-0.0919833	0.2130324	-0.0345675	0.64041313	0.30761329	2.05E-05
0.37666027	-0.016269	0.82602952	-0.2529737	0.00051242	0.09374578	0.20435719
8.99E-06	-0.1159274	0.1160901	0.00240242	0.97410914	0.30613589	2.26E-05
8.14E-06	-0.1236769	0.09349732	0.04578221	0.53604195	0.31528866	1.23E-05
0.00010136	-0.1409077	0.05573291	-0.017076	0.81754653	0.24373072	0.00082822
0.70025488	-0.0325551	0.66000074	-0.2272484	0.00186603	0.04910169	0.50686376
0.07219376	-0.0134442	0.85587361	-0.2318906	0.00149279	0.36741502	2.68E-07
1.13E-05	-0.1348152	0.06730695	0.03453135	0.64076248	0.30276061	2.80E-05
0.00021168	-0.122264	0.09733256	-0.009952	0.89304886	0.27067555	0.00019421
7.99E-05	-0.0665193	0.36831979	-0.1708979	0.02002679	0.17559902	0.01681062
0.00117439	-0.0987471	0.18113348	-0.0530811	0.4730082	0.23786864	0.00111271
9.40E-05	-0.1064897	0.14910371	-0.0072111	0.92239406	0.26470722	0.0002714
0.1354175	-0.0846735	0.25182119	-0.1577879	0.03194832	0.12441528	0.09154119
0.66283512	0.0740694	0.31634288	-0.3356388	3.01E-06	-0.1216055	0.0991623
0.00025775	-0.1303907	0.07688645	-0.0202811	0.78407556	0.24402367	0.00081593
0.00143086	-0.1134671	0.12409013	-0.0559975	0.44900188	0.22383205	0.00219302
8.06E-09	-0.1144201	0.12094217	0.01630616	0.82563816	0.38348059	7.13E-08
8.16E-05	-0.1167764	0.11342458	-0.0221001	0.76525029	0.27317694	0.0001684
2.61E-05	-0.1417255	0.0543126	0.0569092	0.44164034	0.29165699	5.63E-05
4.36E-09	-0.2317492	0.00150307	0.09896563	0.18016501	0.50885524	1.41E-13
0.00076008	-0.0890443	0.22807962	-0.0539553	0.46573956	0.24574044	0.00074725
0.02321979	-0.0163804	0.82485695	-0.1157802	0.11655717	0.30575985	2.31E-05
0.00017709	-0.0036547	0.96062216	-0.1434817	0.0513638	0.19683139	0.0072449
7.29E-06	-0.1008194	0.17210257	0.00806356	0.91325432	0.32039409	8.75E-06

0.00044375	-0.0983956	0.1826995	-0.0161435	0.82735034	0.24622724	0.00072877
0.00134127	-0.0929353	0.20831459	-0.0690845	0.35009827	0.26071984	0.00033794
0.00130348	-0.118152	0.10920762	-0.0471907	0.52356003	0.18613924	0.01118652
0.00026703	-0.0977112	0.18577682	-0.0330624	0.65503962	0.26680885	0.00024144
0.00059332	-0.1127237	0.12658963	-0.0394197	0.5942142	0.23168625	0.00150766
0.08728886	0.02042069	0.78262672	-0.277509	0.00013112	0.28406334	8.91E-05
0.00010539	-0.0940665	0.20280623	-0.014078	0.84915794	0.26063077	0.00033959
0.00011531	-0.1379216	0.06118175	0.04911141	0.50677953	0.33311448	3.60E-06
0.97954601	0.0245617	0.73999417	-0.3566299	6.27E-07	0.03626077	0.62412108
0.0849357	-0.0495505	0.50298297	-0.211184	0.00390717	0.13731381	0.06234284
0.00155654	-0.1137691	0.12308584	-0.0443694	0.54870963	0.24972492	0.00060786
0.00011611	0.19754912	0.00703123	-0.5705497	2.29E-17	-0.5430092	1.40E-15
5.19E-12	0.37907326	1.03E-07	-0.5309934	7.54E-15	-0.7757856	1.92E-38
7.60E-05	0.17438034	0.01759792	-0.4295733	1.05E-09	-0.7294244	5.34E-32
2.43E-08	0.14321966	0.05179512	-0.5347702	4.48E-15	-0.7596844	4.86E-36
2.66E-09	-0.3445311	1.57E-06	0.66951019	2.05E-25	0.77215505	6.96E-38
0.28867084	0.06345888	0.39081003	-0.3678591	2.59E-07	-0.4711481	1.30E-11
1.84E-09	0.27914781	0.00011915	-0.6174255	7.98E-21	-0.6802291	1.77E-26
0.04168281	0.28813642	6.97E-05	-0.336045	2.92E-06	-0.5306274	7.92E-15
6.04E-05	0.43790301	4.56E-10	-0.4213714	2.33E-09	-0.7331848	1.80E-32
0.55940882	0.10282784	0.16367268	-0.2894021	6.46E-05	-0.6088271	3.79E-20
2.41E-06	0.43204543	8.22E-10	-0.5341094	4.90E-15	-0.7175788	1.46E-30
4.90E-05	0.26558949	0.00025842	-0.5396607	2.26E-15	-0.5999525	1.81E-19
1.96E-05	0.20781589	0.00453262	-0.4219909	2.20E-09	-0.715953	2.27E-30
0.0765098	0.18462205	0.01187706	-0.351042	9.63E-07	-0.607947	4.44E-20
0.15935661	0.29556734	4.42E-05	-0.3433928	1.71E-06	-0.5915116	7.63E-19
2.64E-10	0.39423705	2.82E-08	-0.6634013	7.90E-25	-0.7425212	1.12E-33
0.00039717	0.27985931	0.00011428	-0.4918484	1.16E-12	-0.602621	1.14E-19
5.29E-08	0.54578604	9.42E-16	-0.560273	1.11E-16	-0.7469064	2.90E-34
1.58E-06	0.09091861	0.21839918	-0.4051324	1.06E-08	-0.6778805	3.06E-26
0.33120449	0.44046859	3.52E-10	-0.3634929	3.67E-07	-0.4974095	5.91E-13
0.0004585	0.19639917	0.00737635	-0.385717	5.89E-08	-0.6266197	1.43E-21
7.25E-06	0.38439236	6.60E-08	-0.4996702	4.47E-13	-0.7302206	4.25E-32
2.91E-05	0.18536116	0.01153612	-0.5088671	1.41E-13	-0.6289943	9.06E-22
0.1243943	0.1312778	0.07488217	-0.3201104	8.92E-06	-0.5821041	3.62E-18
0.22326157	0.06029328	0.41492296	-0.1305617	0.07649676	-0.2040654	0.0053337
9.27E-06	-0.2688594	0.0002152	0.45869264	5.16E-11	0.78455274	7.76E-40
0.02726038	0.30450769	2.51E-05	-0.4002113	1.66E-08	-0.5817271	3.85E-18
2.87E-12	0.27279854	0.00017209	-0.641562	7.67E-23	-0.7832544	1.26E-39
0.06373292	0.1565829	0.03329817	-0.3495778	1.08E-06	-0.5657848	4.79E-17
4.46E-11	0.25385475	0.00048904	-0.5863331	1.81E-18	-0.7665839	4.79E-37
3.75E-07	0.32969156	4.59E-06	-0.5692499	2.80E-17	-0.7680232	2.93E-37
0.00050935	0.3858662	5.82E-08	-0.5607972	1.03E-16	-0.554531	2.63E-16
1.02E-12	-0.4035108	1.23E-08	0.63913857	1.25E-22	0.86564962	7.03E-57
1.11E-14	-0.3754252	1.40E-07	0.70241666	7.92E-29	0.82681627	1.32E-47
2.58E-09	0.31393612	1.35E-05	-0.6023099	1.20E-19	-0.733262	1.76E-32

6.89E-05	0.33500364	3.15E-06	-0.4624885	3.41E-11	-0.7466722	3.12E-34
8.61E-05	0.37773265	1.15E-07	-0.4895547	1.53E-12	-0.7161307	2.16E-30
9.15E-24	0.42891452	1.12E-09	-0.7837351	1.05E-39	-0.8563295	2.08E-54
3.33E-15	0.56299124	7.35E-17	-0.7584159	7.38E-36	-0.8095069	3.44E-44
0.01549037	0.23815358	0.00109703	-0.3584811	5.43E-07	-0.6330722	4.11E-22
0.03023963	0.30171783	3.00E-05	-0.4353299	5.92E-10	-0.6259278	1.63E-21
0.56463219	0.0737394	0.31850968	-0.2371632	0.00115241	-0.3630354	3.80E-07
0.01630854	0.61740561	8.00E-21	-0.3751693	1.43E-07	-0.5514428	4.14E-16
7.24E-06	0.44362871	2.54E-10	-0.4709065	1.34E-11	-0.7508207	8.52E-35
2.64E-05	0.19876217	0.00668283	-0.5557071	2.20E-16	-0.6183125	6.77E-21
2.56E-08	0.41747126	3.38E-09	-0.5563468	2.00E-16	-0.8085264	5.24E-44
2.07E-08	0.32464925	6.53E-06	-0.6258218	1.66E-21	-0.7113304	7.81E-30
0.0002945	0.49221743	1.11E-12	-0.4972188	6.05E-13	-0.7205538	6.46E-31
0.0001028	0.30204273	2.94E-05	-0.4929139	1.02E-12	-0.5797684	5.28E-18
0.02199791	0.39517709	2.59E-08	-0.3935054	3.00E-08	-0.6824269	1.06E-26
5.37E-05	0.26258678	0.0003051	-0.4886777	1.70E-12	-0.6543293	5.53E-24
0.00275177	0.38722398	5.18E-08	-0.4284687	1.17E-09	-0.7026349	7.49E-29
4.29E-15	0.40358797	1.22E-08	-0.6599732	1.66E-24	-0.81959	3.90E-46
3.52E-16	0.40765885	8.44E-09	-0.66559	4.89E-25	-0.8013125	1.08E-42
8.79E-12	0.36632491	2.93E-07	-0.5918171	7.25E-19	-0.8072205	9.15E-44
0.00328294	0.11643102	0.11450312	-0.4219416	2.21E-09	-0.6240545	2.32E-21
9.27E-05	0.22562919	0.00201503	-0.506065	2.01E-13	-0.543332	1.34E-15
3.79E-05	0.4993651	4.64E-13	-0.5057075	2.10E-13	-0.7395096	2.77E-33
0.34900828	0.03499766	0.63625727	0.12270335	0.09612687	0.07092279	0.33739523
0.00396949	-0.1053698	0.15345092	0.25651435	0.00042431	0.09784893	0.18515432
0.05288568	-0.097145	0.18835098	0.17435017	0.01761781	-0.0154248	0.83492421
0.00022345	0.21936659	0.00269878	-0.2513976	0.00055685	-0.471804	1.21E-11
0.37286982	-0.1438755	0.05072106	0.05022441	0.49718583	-0.1808343	0.01376709
0.26560774	-0.1943129	0.00804112	-0.1179329	0.10987114	-0.0533899	0.4704337
0.0103524	-0.215373	0.00323827	0.22750657	0.00184323	0.11925748	0.10590885
3.34E-11	0.82609074	1.87E-47	-0.405046	1.07E-08	-0.3980896	2.00E-08
9.43E-08	0.80857215	5.14E-44	-0.4858002	2.40E-12	-0.354669	7.30E-07
1.81E-33	0.86550993	7.68E-57	-0.7182593	1.21E-30	-0.557815	1.61E-16
0.00066532	0.760896	3.25E-36	-0.3034722	2.68E-05	-0.2759458	0.00014358
5.62E-21	0.86100229	1.26E-55	-0.6259974	1.60E-21	-0.5006271	3.97E-13
3.31E-10	0.84613574	6.73E-52	-0.5250948	1.68E-14	-0.5076555	1.64E-13
3.52E-05	0.7422949	1.20E-33	-0.3224642	7.59E-06	-0.1189075	0.1069446
0.35288124	0.80205026	7.98E-43	-0.2510905	0.00056591	0.03723743	0.6148057
0.20342801	0.83715143	7.83E-50	-0.3680357	2.55E-07	-0.3201888	8.87E-06
1.01E-05	0.81984991	3.46E-46	-0.3278195	5.24E-06	-0.4283736	1.18E-09
0.59978277	0.71009984	1.08E-29	-0.2677784	0.00022868	-0.3188236	9.73E-06
2.70E-14	0.86186764	7.43E-56	-0.5312359	7.29E-15	-0.3797019	9.79E-08
6.99E-12	0.78237461	1.75E-39	-0.5824173	3.44E-18	-0.2936667	4.97E-05
3.81E-05	0.84561604	8.93E-52	-0.3983525	1.96E-08	-0.1156534	0.11696067
9.53E-25	0.86255275	4.87E-56	-0.7103372	1.02E-29	-0.5776918	7.38E-18
7.98E-11	0.8192415	4.57E-46	-0.6992111	1.79E-28	-0.6186148	6.40E-21

4.83E-18	0.85746268	1.06E-54	-0.6570543	3.10E-24	-0.5904275	9.15E-19
1.96E-12	0.91352862	2.09E-73	-0.5255062	1.59E-14	-0.4841233	2.92E-12
0.54403784	0.61442459	1.38E-20	-0.1536322	0.03680934	-0.1230445	0.09519876
3.54E-07	0.76795987	2.99E-37	-0.4128264	5.23E-09	-0.1700521	0.02065878
8.91E-17	0.81033899	2.40E-44	-0.5699577	2.51E-17	-0.3315818	4.02E-06
9.10E-21	0.84563213	8.86E-52	-0.718044	1.29E-30	-0.4230844	1.98E-09
0.08050575	0.62733932	1.24E-21	-0.3172605	1.08E-05	-0.192784	0.00856154
1.55E-05	0.90760101	6.84E-71	-0.4925042	1.07E-12	-0.1771442	0.01585662
1.21E-09	0.74507852	5.11E-34	-0.4597535	4.60E-11	-0.0418845	0.57134072
0.02220301	0.85167005	3.08E-53	-0.2402071	0.00098991	-0.156787	0.03306619
1.85E-41	0.84804866	2.35E-52	-0.7867269	3.42E-40	-0.6020647	1.25E-19
4.77E-12	0.88204136	1.04E-61	-0.6434308	5.26E-23	-0.4836443	3.09E-12
2.07E-11	0.81063031	2.12E-44	-0.5045999	2.42E-13	-0.2941269	4.83E-05
3.91E-07	0.63014088	7.26E-22	-0.4087863	7.61E-09	-0.5098479	1.24E-13
4.88E-07	0.8049956	2.34E-43	-0.3363614	2.85E-06	-0.2204932	0.00256207
1.95E-15	0.9075782	6.99E-71	-0.6300031	7.46E-22	-0.606423	5.82E-20
4.47E-23	0.84532688	1.05E-51	-0.6258036	1.67E-21	-0.522659	2.33E-14
2.00E-12	0.78193433	2.05E-39	-0.5311797	7.35E-15	-0.6344214	3.16E-22
1.36E-07	0.82726345	1.06E-47	-0.5362944	3.62E-15	-0.2452124	0.00076779
1.97E-28	0.8841037	2.28E-62	-0.7418037	1.39E-33	-0.515554	5.94E-14
1.89E-05	0.84694297	4.32E-52	-0.4846826	2.73E-12	-0.3186003	9.88E-06
3.55E-11	0.8222961	1.12E-46	-0.3948034	2.68E-08	-0.3301857	4.43E-06
6.54E-05	0.89575055	2.44E-66	-0.3613544	4.34E-07	-0.136481	0.06396304
0.00012339	0.89466433	5.99E-66	-0.449763	1.34E-10	-0.2003672	0.00624553
0.03727352	0.87361245	3.84E-59	-0.3381339	2.51E-06	-0.336587	2.81E-06
0.14023372	0.75363086	3.48E-35	-0.3013828	3.06E-05	-0.2897792	6.31E-05
0.03067101	0.84589031	7.69E-52	-0.3386687	2.41E-06	-0.0362598	0.62413042
8.35E-06	0.85730178	1.17E-54	-0.5010617	3.76E-13	-0.4790025	5.31E-12
2.69E-09	0.8389277	3.13E-50	-0.6007497	1.57E-19	-0.290032	6.21E-05
4.96E-17	0.85940128	3.33E-55	-0.7422317	1.22E-33	-0.5943093	4.75E-19
1.28E-08	-0.4214287	2.32E-09	0.6993647	1.72E-28	0.55227424	3.66E-16
3.19E-23	-0.4798175	4.83E-12	0.85650115	1.88E-54	0.41300142	5.15E-09
1.96E-16	-0.3966086	2.29E-08	0.78949961	1.19E-40	0.30900751	1.87E-05
1.20E-09	0.45816972	5.47E-11	-0.6713034	1.37E-25	-0.1679137	0.022334
5.95E-19	0.37227117	1.81E-07	-0.4589339	5.03E-11	-0.3691555	2.33E-07
1.45E-08	0.48670978	2.15E-12	-0.4217999	2.24E-09	-0.6551063	4.69E-24
2.17E-25	0.2747521	0.00015383	-0.6411349	8.35E-23	-0.3145963	1.29E-05
1.90E-32	0.47001965	1.48E-11	-0.6198246	5.12E-21	-0.3180194	1.03E-05
8.70E-49	-0.2771438	0.00013394	0.69111769	1.32E-27	0.56738613	3.74E-17
9.23E-25	-0.3874503	5.08E-08	0.82800117	7.46E-48	0.50431217	2.50E-13
3.78E-27	0.33453585	3.25E-06	-0.6092072	3.54E-20	-0.4482712	1.57E-10
1.99E-12	0.38509114	6.21E-08	-0.591627	7.48E-19	-0.6925896	9.24E-28
1.92E-50	-0.497476	5.86E-13	0.84579753	8.09E-52	0.41329048	5.01E-09
6.34E-05	0.43277158	7.64E-10	-0.5601275	1.14E-16	-0.1983649	0.0067952
1.00E-17	-0.468981	1.66E-11	0.49952568	4.55E-13	0.49408607	8.87E-13
5.13E-22	0.38276581	7.57E-08	-0.6193932	5.54E-21	-0.3119085	1.55E-05

5.61E-08	0.08885632	0.22906698	0.23495436	0.00128526	-0.0658633	0.3730722
5.68E-10	0.31855318	9.91E-06	-0.6431601	5.55E-23	-0.2007303	0.00615022
4.01E-41	0.26454235	0.00027389	-0.6610475	1.32E-24	-0.4900944	1.44E-12
9.11E-16	-0.4495759	1.37E-10	0.78908585	1.39E-40	0.28703716	7.45E-05
6.55E-41	0.50657511	1.88E-13	-0.7832863	1.24E-39	-0.3555629	6.81E-07
2.06E-17	-0.3969985	2.21E-08	0.71875837	1.06E-30	0.36104305	4.45E-07
1.98E-26	-0.3998463	1.71E-08	0.65870859	2.18E-24	0.29165076	5.63E-05
8.87E-43	-0.4883459	1.77E-12	0.89734372	6.44E-67	0.57786617	7.18E-18
7.63E-06	0.25724011	0.00040808	-0.3936995	2.95E-08	-0.6221242	3.33E-21
2.75E-35	0.20769613	0.00455643	-0.5659572	4.67E-17	-0.2443488	0.00080249
8.29E-10	0.23846866	0.00107993	-0.5503984	4.83E-16	-0.2458659	0.00074245
0.05172879	-0.1283272	0.08171643	0.3631642	3.76E-07	-0.0706447	0.33929754
4.89E-41	0.62612082	1.57E-21	-0.9176423	2.93E-75	-0.7510555	7.91E-35
3.22E-44	0.42463927	1.70E-09	-0.6901474	1.68E-27	-0.5473749	7.49E-16
2.35E-12	0.46368738	2.99E-11	-0.685545	5.07E-27	-0.2987441	3.62E-05
6.04E-23	-0.4452653	2.15E-10	0.87196715	1.16E-58	0.45744645	5.91E-11
2.09E-13	0.45596928	6.93E-11	-0.7127521	5.35E-30	-0.2220222	0.00238659
7.84E-17	0.63944723	1.17E-22	-0.576185	9.39E-18	-0.3809404	8.83E-08
1.33E-45	-0.3627145	3.90E-07	0.66943299	2.08E-25	0.27695772	0.0001354
3.95E-36	0.43939165	3.92E-10	-0.583191	3.03E-18	-0.3341328	3.35E-06
1.15E-25	0.42696657	1.36E-09	-0.6745613	6.55E-26	-0.825767	2.18E-47
1.20E-26	0.20318663	0.00553885	-0.6297649	7.81E-22	-0.6633756	7.94E-25
3.43E-07	0.27122929	0.00018819	-0.6526466	7.88E-24	0.00595994	0.93582747
8.61E-12	0.70829154	1.74E-29	-0.5572119	1.76E-16	-0.3698354	2.21E-07
3.97E-05	0.41147166	5.94E-09	-0.5958096	3.68E-19	-0.5671235	3.90E-17
2.34E-21	-0.5189314	3.82E-14	0.85873018	4.99E-55	0.68192444	1.19E-26
0.0001732	0.26976787	0.00020445	-0.5011533	3.72E-13	-0.4882317	1.79E-12
5.38E-26	-0.4313594	8.79E-10	0.89201664	5.13E-65	0.76364884	1.30E-36
2.25E-15	-0.3732199	1.67E-07	0.74979979	1.18E-34	0.37261484	1.76E-07
1.88E-34	-0.4873325	2.00E-12	0.7456976	4.22E-34	0.42080901	2.46E-09
4.45E-09	0.48111678	4.15E-12	-0.6279528	1.11E-21	-0.4350141	6.11E-10
1.13E-09	0.32479279	6.46E-06	-0.5078394	1.60E-13	-0.5493135	5.65E-16
6.27E-53	0.35706566	6.06E-07	-0.7324793	2.21E-32	-0.3456312	1.45E-06
4.92E-15	-0.4768883	6.77E-12	0.72317854	3.12E-31	0.76661536	4.74E-37
4.93E-27	-0.5249789	1.71E-14	0.93878535	1.27E-86	0.65244878	8.21E-24
2.16E-30	0.53494364	4.37E-15	-0.6189699	6.00E-21	-0.40235	1.37E-08
2.69E-16	-0.4566378	6.45E-11	0.82068408	2.36E-46	0.53814549	2.79E-15
1.05E-14	-0.4537851	8.75E-11	0.6291458	8.80E-22	0.26451063	0.00027437
8.32E-14	0.22851209	0.0017568	-0.7283227	7.32E-32	-0.3020629	2.93E-05
9.07E-15	-0.4390344	4.07E-10	0.82617587	1.79E-47	0.54229172	1.55E-15
7.20E-38	0.3889793	4.45E-08	-0.6951184	4.96E-28	-0.3065114	2.20E-05
1.11E-19	-0.3028426	2.79E-05	0.68659733	3.94E-27	0.44789501	1.63E-10
3.35E-25	-0.2538414	0.00048939	0.68370473	7.84E-27	0.27870763	0.00012227
4.64E-29	0.3850307	6.25E-08	-0.6176457	7.66E-21	-0.5203618	3.16E-14
5.61E-36	-0.548208	6.64E-16	0.89271414	2.93E-65	0.57655442	8.85E-18
1.74E-34	0.3671042	2.75E-07	-0.6479145	2.10E-23	-0.3628684	3.85E-07

3.22E-13	0.3406317	2.09E-06	-0.5547539	2.54E-16	-0.7494817	1.30E-34
9.88E-16	0.53280464	5.87E-15	-0.7153192	2.69E-30	-0.2163953	0.0030916
1.22E-35	-0.4997951	4.40E-13	0.78485506	6.93E-40	0.453125	9.39E-11
0.03785248	0.16862611	0.02176333	-0.3384379	2.45E-06	-0.3385868	2.43E-06
5.29E-18	0.44140964	3.19E-10	-0.6133529	1.68E-20	-0.1574063	0.03237062
3.89E-39	-0.4651859	2.54E-11	0.86122769	1.10E-55	0.55406448	2.81E-16
4.99E-42	-0.5075185	1.67E-13	0.84816796	2.20E-52	0.37501838	1.44E-07
3.55E-08	0.69666256	3.38E-28	-0.6288862	9.25E-22	-0.5071538	1.75E-13
2.51E-27	0.28833313	6.89E-05	-0.5923495	6.62E-19	-0.441854	3.05E-10
1.67E-21	-0.5645096	5.83E-17	0.88137025	1.69E-61	0.58834459	1.30E-18
1.17E-30	-0.5196147	3.49E-14	0.87687186	4.11E-60	0.40976218	6.96E-09
6.22E-31	-0.3956653	2.49E-08	0.87701057	3.73E-60	0.459654	4.65E-11
8.34E-08	-0.3720518	1.84E-07	0.73409916	1.38E-32	0.24814027	0.00066014
1.78E-29	0.72509332	1.83E-31	-0.9303501	1.16E-81	-0.6104633	2.83E-20
3.15E-13	-0.3722076	1.82E-07	0.57621418	9.35E-18	0.57107373	2.11E-17
9.97E-25	0.27731731	0.0001326	-0.5323676	6.24E-15	-0.5026281	3.09E-13
2.73E-40	-0.5613016	9.51E-17	0.92308895	7.22E-78	0.75415114	2.95E-35
4.21E-49	-0.5273938	1.23E-14	0.87344217	4.31E-59	0.39211453	3.39E-08
2.09E-19	-0.2738775	0.00016177	0.57483615	1.16E-17	0.80968899	3.18E-44
1.63E-21	0.59195443	7.08E-19	-0.6939874	6.56E-28	-0.6446339	4.12E-23
2.63E-10	-0.405708	1.01E-08	0.66449601	6.21E-25	0.32170369	8.00E-06
7.08E-31	0.60346382	9.80E-20	-0.9153109	3.38E-74	-0.6881462	2.72E-27
4.26E-12	0.47180468	1.21E-11	-0.5783154	6.68E-18	-0.1154583	0.1175837
7.02E-10	0.32909448	4.79E-06	-0.5972162	2.90E-19	-0.0988366	0.1807364
0.00012586	-0.3246127	6.55E-06	0.60428113	8.49E-20	0.15580468	0.03419544
9.37E-24	-0.5243386	1.86E-14	0.88389552	2.66E-62	0.53488584	4.40E-15
1.02E-14	0.50105641	3.76E-13	-0.6669172	3.65E-25	-0.3102787	1.72E-05
2.85E-10	0.26804247	0.00022532	-0.4048805	1.09E-08	-0.4939746	8.99E-13
7.18E-77	-0.522152	2.49E-14	0.92303245	7.70E-78	0.7497053	1.21E-34
3.14E-28	-0.5224813	2.38E-14	0.89288202	2.56E-65	0.76041559	3.82E-36
1.34E-42	-0.5850826	2.22E-18	0.94747611	1.56E-92	0.67668877	4.02E-26
4.68E-33	-0.5054984	2.16E-13	0.93251468	7.13E-83	0.70897222	1.45E-29
2.40E-31	-0.525635	1.56E-14	0.8718033	1.29E-58	0.45597732	6.92E-11
7.88E-42	-0.4820149	3.74E-12	0.85701221	1.39E-54	0.51792783	4.35E-14
5.85E-64	-0.5744046	1.25E-17	0.94922899	7.56E-94	0.66972608	1.95E-25
2.61E-30	-0.1320212	0.07323539	0.74132937	1.60E-33	0.32422476	6.72E-06
1.44E-06	0.24558312	0.00075332	-0.4692119	1.62E-11	-0.6939746	6.58E-28
0.0202295	-0.3502233	1.02E-06	0.49237208	1.09E-12	0.0335577	0.6502116
3.32E-14	-0.326186	5.87E-06	0.5325937	6.05E-15	-0.0619947	0.40185636
5.85E-27	0.44678667	1.83E-10	-0.5418296	1.66E-15	-0.1911553	0.00914858
3.82E-38	-0.4575063	5.87E-11	0.80512323	2.22E-43	0.3270416	5.53E-06
6.86E-39	-0.3051008	2.41E-05	0.76122907	2.91E-36	0.5589934	1.35E-16
1.85E-15	0.50175025	3.45E-13	-0.8349384	2.42E-49	-0.6300162	7.44E-22
5.37E-48	-0.5310533	7.47E-15	0.8825425	7.19E-62	0.51804482	4.29E-14
0.00587802	-0.3623129	4.02E-07	0.55510722	2.41E-16	0.43921017	4.00E-10
6.69E-27	-0.2687523	0.0002165	0.58089848	4.40E-18	0.2181401	0.00285509

4.47E-41	0.3030066	2.76E-05	-0.6215391	3.72E-21	-0.3046044	2.49E-05
0.00112287	0.57086512	2.18E-17	-0.5425285	1.50E-15	-0.2477499	0.00067364
8.80E-16	0.51125679	1.04E-13	-0.8196279	3.83E-46	-0.4430392	2.70E-10
1.00E-29	-0.4613379	3.87E-11	0.82570421	2.24E-47	0.76609216	5.67E-37
3.60E-40	-0.5663	4.43E-17	0.9619381	4.81E-105	0.73632333	7.17E-33
1.04E-24	0.5914601	7.69E-19	-0.8760713	7.15E-60	-0.5730465	1.55E-17
3.59E-27	0.48000708	4.72E-12	-0.8042201	3.24E-43	-0.67873	2.51E-26
2.28E-09	0.59334938	5.60E-19	-0.4546971	7.94E-11	-0.2339937	0.0013473
2.73E-13	-0.372506	1.78E-07	0.59981633	1.85E-19	0.36592802	3.02E-07
9.21E-54	-0.5131695	8.10E-14	0.79302706	3.01E-41	0.3594087	5.05E-07
0.0012259	-0.3645869	3.36E-07	0.51757683	4.56E-14	0.69934618	1.73E-28
1.18E-38	0.36555021	3.11E-07	-0.6025149	1.16E-19	-0.3653211	3.17E-07
3.70E-42	-0.4932227	9.85E-13	0.93007954	1.63E-81	0.78673278	3.41E-40
2.43E-47	-0.4694083	1.58E-11	0.78959571	1.14E-40	0.48816303	1.81E-12
2.10E-22	0.53033303	8.25E-15	-0.8267796	1.34E-47	-0.2866651	7.62E-05
0.00229346	0.68249018	1.04E-26	-0.4708861	1.34E-11	-0.3478114	1.23E-06
4.56E-09	0.3066936	2.18E-05	-0.463458	3.07E-11	0.21222266	0.00373062
2.39E-71	-0.5440299	1.21E-15	0.9366599	2.61E-85	0.596181	3.46E-19
2.17E-21	0.45927462	4.85E-11	-0.7565162	1.37E-35	-0.4547185	7.92E-11
4.90E-27	-0.5409729	1.87E-15	0.8119899	1.17E-44	0.49621324	6.84E-13
3.25E-20	0.77780963	9.28E-39	-0.6605297	1.47E-24	-0.6262516	1.53E-21
6.13E-43	-0.4022098	1.39E-08	0.78790676	2.18E-40	0.35999477	4.83E-07
1.35E-32	0.41155403	5.89E-09	-0.7257297	1.53E-31	-0.6866189	3.92E-27
6.68E-43	-0.5786738	6.30E-18	0.93495562	2.74E-84	0.6305517	6.71E-22
8.33E-47	0.50540448	2.18E-13	-0.8084159	5.49E-44	-0.4098197	6.92E-09
6.22E-56	0.54214845	1.59E-15	-0.8048471	2.49E-43	-0.491744	1.18E-12
4.06E-12	0.48086535	4.27E-12	-0.8066291	1.18E-43	-0.3842447	6.68E-08
1.33E-43	-0.578422	6.56E-18	0.92701524	7.17E-80	0.6828789	9.53E-27
3.93E-11	0.29874299	3.62E-05	-0.6848351	6.00E-27	-0.5383699	2.71E-15
9.19E-10	-0.3650219	3.25E-07	0.61470628	1.31E-20	0.08196793	0.26734586
5.86E-27	-0.4586729	5.18E-11	0.90455001	1.16E-69	0.56211408	8.41E-17
2.01E-29	-0.325736	6.05E-06	0.68407933	7.18E-27	0.77896048	6.11E-39
0.10374383	-0.2209158	0.00251244	0.17700836	0.01593853	0.25491755	0.00046215
6.67E-28	-0.4946941	8.23E-13	0.89768521	4.82E-67	0.77071458	1.15E-37
1.36E-26	0.26549491	0.00025979	-0.6805604	1.64E-26	-0.3067776	2.16E-05
9.23E-20	0.75677728	1.26E-35	-0.6390136	1.28E-22	-0.5714049	2.00E-17
2.82E-17	-0.3489243	1.13E-06	0.80671571	1.13E-43	0.58475193	2.35E-18
5.80E-12	-0.4415615	3.14E-10	0.6696362	1.99E-25	0.770211	1.37E-37
2.25E-30	0.26894911	0.00021412	-0.5579509	1.58E-16	-0.2421247	0.00089864
7.45E-25	-0.5160579	5.56E-14	0.89193538	5.47E-65	0.4334076	7.17E-10
5.30E-31	-0.2540258	0.00048462	0.64784451	2.14E-23	0.25594586	0.00043744
7.84E-30	0.37183006	1.88E-07	-0.777528	1.03E-38	-0.2204306	0.00256951
1.15E-15	-0.4808805	4.27E-12	0.83030103	2.43E-48	0.39682436	2.24E-08
1.65E-44	-0.5103016	1.17E-13	0.9488246	1.53E-93	0.58379467	2.74E-18
6.02E-13	-0.2135264	0.00351922	0.66309195	8.45E-25	0.66348786	7.75E-25
9.65E-48	-0.4858075	2.39E-12	0.75727082	1.07E-35	0.25494523	0.00046147

7.78E-08	-0.3910966	3.71E-08	0.6904024	1.58E-27	0.57733275	7.82E-18
0.00012215	0.18101775	0.0136698	-0.4398697	3.74E-10	-0.5979952	2.53E-19
3.25E-40	-0.3456206	1.45E-06	0.86343358	2.82E-56	0.50102741	3.78E-13
3.90E-36	-0.5866149	1.73E-18	0.76175981	2.44E-36	0.4517317	1.09E-10
1.17E-30	0.55827429	1.50E-16	-0.8499835	7.99E-53	-0.3664456	2.90E-07
6.96E-11	-0.5727454	1.62E-17	0.72602439	1.40E-31	0.51726226	4.75E-14
1.63E-10	-0.4282032	1.20E-09	0.61971927	5.22E-21	0.70486326	4.23E-29
9.93E-30	0.80251721	6.58E-43	-0.8024575	6.75E-43	-0.697674	2.63E-28
8.29E-07	0.28086885	0.00010769	-0.6567712	3.30E-24	-0.5599208	1.17E-16
8.28E-78	-0.5683738	3.21E-17	0.87785198	2.07E-60	0.59602004	3.55E-19
8.15E-21	-0.4462953	1.93E-10	0.86166024	8.44E-56	0.44692264	1.81E-10
4.11E-15	0.24857886	0.00064527	-0.5572263	1.76E-16	-0.6820947	1.15E-26
9.17E-22	0.52146891	2.73E-14	-0.7162442	2.10E-30	-0.8253838	2.61E-47
3.12E-34	-0.4212666	2.35E-09	0.73706633	5.75E-33	0.25203586	0.00053845
1.39E-28	-0.5326968	5.96E-15	0.84028994	1.54E-50	0.56892276	2.95E-17
1.58E-10	0.55797918	1.57E-16	-0.7929338	3.12E-41	-0.3972911	2.15E-08
7.20E-28	0.39530441	2.57E-08	-0.7155153	2.55E-30	-0.5084552	1.48E-13
1.27E-18	0.28566541	8.09E-05	-0.7142183	3.62E-30	-0.3407406	2.08E-06
4.77E-27	0.5204045	3.14E-14	-0.8396928	2.10E-50	-0.4335871	7.05E-10
3.07E-10	-0.4248466	1.67E-09	0.78270193	1.55E-39	0.47710957	6.60E-12
1.74E-08	0.50985759	1.24E-13	-0.6733841	8.56E-26	-0.4666877	2.15E-11
5.88E-34	-0.3532364	8.14E-07	0.74870703	1.66E-34	0.23454314	0.00131149
4.59E-09	0.42558342	1.55E-09	-0.7416474	1.46E-33	-0.6152131	1.20E-20
1.32E-08	0.68678126	3.77E-27	-0.7361878	7.46E-33	-0.6122227	2.06E-20
9.68E-18	-0.3410302	2.03E-06	0.72630848	1.30E-31	0.70261368	7.53E-29
2.10E-07	0.50386676	2.65E-13	-0.6621423	1.04E-24	-0.6236467	2.50E-21
9.06E-28	0.41001863	6.79E-09	-0.5430111	1.40E-15	-0.3735797	1.63E-07
4.92E-07	0.07510794	0.3095864	-0.3628722	3.85E-07	-0.4988851	4.92E-13
0.00031211	0.36810185	2.54E-07	-0.4901849	1.42E-12	-0.0341983	0.64398792
6.76E-28	-0.4962746	6.79E-13	0.8599335	2.42E-55	0.42722251	1.32E-09
2.06E-47	-0.4220138	2.19E-09	0.69293932	8.48E-28	0.4500919	1.29E-10
5.71E-29	-0.3781253	1.12E-07	0.74391758	7.30E-34	0.14992661	0.0416572
0.06674069	0.16007272	0.02951626	-0.5325258	6.10E-15	0.01175728	0.87379658
5.81E-26	-0.3708851	2.03E-07	0.58581395	1.97E-18	0.14685779	0.04606775
7.15E-17	-0.5789039	6.07E-18	0.70543895	3.65E-29	0.43260234	7.77E-10
1.60E-56	-0.5531526	3.22E-16	0.87587665	8.18E-60	0.54796546	6.88E-16
3.74E-56	-0.4915408	1.21E-12	0.73295019	1.93E-32	0.37393856	1.58E-07
1.42E-50	-0.5482061	6.64E-16	0.86014163	2.13E-55	0.43171162	8.49E-10
4.31E-05	-0.3657671	3.06E-07	0.38125671	8.60E-08	-0.0155771	0.83331835
1.85E-07	-0.4089279	7.51E-09	0.74249452	1.13E-33	0.3221276	7.77E-06
1.19E-32	0.41666704	3.65E-09	-0.6290398	8.98E-22	-0.4931559	9.93E-13
3.61E-27	0.38299197	7.43E-08	-0.5985765	2.29E-19	-0.2072948	0.00463703
2.11E-09	0.38639139	5.56E-08	-0.6283377	1.03E-21	-0.1335055	0.07003537
1.95E-12	0.21913477	0.00272772	-0.651239	1.06E-23	-0.690139	1.68E-27
1.41E-33	0.42922355	1.09E-09	-0.7734036	4.48E-38	-0.5859239	1.93E-18
5.96E-14	-0.4199565	2.67E-09	0.76053664	3.67E-36	0.72851716	6.92E-32

6.41E-36	-0.226534	0.00193049	0.6127151	1.89E-20	0.14541081	0.04827894
1.83E-29	0.41815319	3.17E-09	-0.6892703	2.07E-27	-0.4192341	2.86E-09
9.51E-08	0.21017157	0.00408646	-0.5376212	3.01E-15	-0.5760722	9.56E-18
2.58E-41	-0.46318	3.16E-11	0.77270223	5.74E-38	0.37110722	1.99E-07
9.78E-32	0.43431118	6.55E-10	-0.6291276	8.83E-22	-0.7139247	3.91E-30
1.30E-42	-0.5581766	1.52E-16	0.84830277	2.04E-52	0.46353055	3.04E-11
7.28E-12	0.54854659	6.32E-16	-0.6344182	3.16E-22	-0.6014706	1.39E-19
5.63E-24	-0.5397016	2.24E-15	0.77706285	1.22E-38	0.41673287	3.63E-09
1.56E-44	-0.5545593	2.61E-16	0.72743671	9.42E-32	0.28207462	0.00010028
1.42E-26	-0.364171	3.47E-07	0.66394512	7.01E-25	0.11274858	0.12650539
6.95E-48	-0.5860345	1.90E-18	0.93867787	1.48E-86	0.54332208	1.34E-15
9.52E-60	-0.4881395	1.81E-12	0.91616252	1.40E-74	0.80697893	1.01E-43
1.44E-18	0.54567005	9.58E-16	-0.7735578	4.25E-38	-0.2203516	0.00257891
1.38E-28	-0.4211484	2.38E-09	0.8198999	3.38E-46	0.40537289	1.04E-08
3.17E-53	0.33782742	2.57E-06	-0.7810118	2.89E-39	-0.3577268	5.76E-07
3.02E-27	-0.4632294	3.15E-11	0.84573527	8.37E-52	0.75201335	5.83E-35
1.33E-35	-0.4758618	7.62E-12	0.91508293	4.28E-74	0.6045813	8.05E-20
2.97E-09	-0.4267048	1.39E-09	0.7635911	1.32E-36	0.34413702	1.62E-06
1.42E-06	-0.246788	0.000708	0.4934476	9.58E-13	0.03964986	0.59206088
2.66E-25	-0.2378263	0.00111506	0.76268771	1.79E-36	0.25777653	0.00039645
5.32E-05	-0.1813003	0.01352118	0.55555717	2.25E-16	-0.0119415	0.8718362
1.05E-20	-0.4455559	2.08E-10	0.6919869	1.07E-27	0.19397013	0.0081553
5.84E-30	-0.6199367	5.01E-21	0.8663031	4.64E-57	0.56408747	6.22E-17
4.71E-35	-0.5356296	3.97E-15	0.94568877	3.07E-91	0.66100499	1.33E-24
3.13E-26	-0.5679077	3.45E-17	0.92610627	2.13E-79	0.64797859	2.08E-23
1.32E-41	0.29184715	5.56E-05	-0.7208755	5.91E-31	-0.3684316	2.47E-07
7.09E-23	0.29844521	3.69E-05	-0.7457693	4.13E-34	-0.5920651	6.95E-19
2.66E-59	-0.5859597	1.92E-18	0.85931849	3.51E-55	0.50769241	1.63E-13
4.08E-67	-0.4982503	5.33E-13	0.78127684	2.62E-39	0.48636592	2.24E-12
1.74E-21	-0.5154113	6.05E-14	0.84060704	1.30E-50	0.76601613	5.82E-37
3.73E-13	-0.4694759	1.57E-11	0.82226705	1.13E-46	0.58034415	4.81E-18
5.59E-35	-0.345671	1.44E-06	0.67521005	5.65E-26	0.2304556	0.00160015
0.04971904	-0.3918588	3.47E-08	0.54212801	1.59E-15	0.44272586	2.79E-10
0.08645381	-0.117043	0.11259754	0.40495861	1.08E-08	-0.1131716	0.12507927
2.48E-41	-0.5119113	9.52E-14	0.87054586	2.97E-58	0.38842098	4.67E-08
1.80E-10	-0.4365757	5.22E-10	0.62838609	1.02E-21	0.31786539	1.04E-05
1.85E-08	0.23064282	0.00158576	-0.4704251	1.41E-11	-0.2210339	0.00249873
8.48E-17	-0.4376625	4.68E-10	0.70886662	1.49E-29	0.25010962	0.00059576
2.08E-15	0.48374506	3.05E-12	-0.6708183	1.53E-25	-0.7034623	6.06E-29
6.40E-12	-0.4144991	4.48E-09	0.75004375	1.09E-34	0.25927441	0.0003656
9.43E-43	-0.3882886	4.73E-08	0.81020253	2.55E-44	0.51641551	5.31E-14
3.20E-38	0.3221488	7.76E-06	-0.6564344	3.54E-24	-0.3678821	2.58E-07
1.19E-53	-0.4608196	4.10E-11	0.71546055	2.59E-30	0.35847984	5.43E-07
7.87E-62	-0.5494484	5.54E-16	0.93227136	9.80E-83	0.60778643	4.57E-20
1.25E-23	-0.4412538	3.24E-10	0.68512713	5.60E-27	0.84895782	1.42E-52
6.69E-12	-0.4153885	4.12E-09	0.7894904	1.19E-40	0.5440059	1.22E-15

1.81E-09	0.42801291	1.22E-09	-0.5271629	1.27E-14	0.13618691	0.06454326
1.61E-14	-0.2332904	0.00139444	0.71716388	1.63E-30	0.35341788	8.03E-07
0.19204684	-0.3091496	1.85E-05	0.38693603	5.31E-08	0.27001371	0.00020163
3.66E-18	-0.2512982	0.00055976	0.7020499	8.70E-29	0.18842188	0.01021419
0.0049028	0.21258024	0.00367153	-0.452113	1.05E-10	-0.5517314	3.97E-16
8.14E-28	-0.3323904	3.79E-06	0.53830509	2.73E-15	0.796197	8.59E-42
6.35E-17	-0.424569	1.71E-09	0.74258266	1.10E-33	0.78246999	1.69E-39
1.51E-29	-0.4728725	1.07E-11	0.84871812	1.62E-52	0.74296594	9.76E-34
2.84E-05	-0.2933393	5.07E-05	0.43328804	7.26E-10	0.24539223	0.00076073
7.69E-05	-0.3603463	4.70E-07	0.61859167	6.43E-21	0.58945439	1.08E-18
2.73E-19	-0.4334799	7.12E-10	0.56561859	4.92E-17	0.68343903	8.35E-27
2.06E-42	-0.4033451	1.25E-08	0.81335078	6.44E-45	0.7749334	2.61E-38
1.92E-10	0.40252042	1.35E-08	-0.6919549	1.08E-27	-0.2500672	0.00059709
4.62E-38	-0.0587673	0.42685116	0.69704349	3.07E-28	0.39604411	2.40E-08
2.36E-40	-0.5974791	2.77E-19	0.92619175	1.93E-79	0.66446742	6.25E-25
7.90E-33	0.51998332	3.32E-14	-0.9295537	3.16E-81	-0.6536009	6.45E-24
1.93E-29	-0.2516232	0.00055028	0.70790516	1.92E-29	0.32151657	8.10E-06
4.85E-23	-0.4713067	1.28E-11	0.83061908	2.08E-48	0.28305625	9.46E-05
1.33E-20	0.36415787	3.48E-07	-0.6916064	1.18E-27	-0.5333253	5.47E-15
3.11E-07	0.60269261	1.12E-19	-0.5949247	4.28E-19	-0.3719468	1.86E-07
1.58E-40	-0.4970631	6.16E-13	0.84431451	1.81E-51	0.50782259	1.61E-13
7.31E-14	0.36209579	4.09E-07	-0.5324584	6.16E-15	-0.1091245	0.13924368
7.08E-16	0.34095873	2.04E-06	-0.4859216	2.36E-12	-0.1456325	0.04793457
1.93E-33	-0.4896944	1.51E-12	0.77732599	1.11E-38	0.44947096	1.38E-10
1.45E-28	0.47593413	7.56E-12	-0.6840122	7.29E-27	-0.5615632	9.14E-17
5.46E-32	-0.5071496	1.75E-13	0.93853002	1.84E-86	0.62981726	7.73E-22
2.83E-58	-0.5654844	5.02E-17	0.96376664	5.78E-107	0.71132348	7.82E-30
1.23E-42	0.5304925	8.07E-15	-0.871129	2.02E-58	-0.7630984	1.56E-36
2.98E-39	0.3254572	6.17E-06	-0.6264355	1.48E-21	-0.4550561	7.64E-11
5.54E-52	0.3708667	2.03E-07	-0.7067108	2.62E-29	-0.5370529	3.26E-15
3.04E-42	0.17629177	0.01637689	-0.6183134	6.77E-21	-0.3890522	4.43E-08
1.77E-57	-0.5590382	1.34E-16	0.96800676	7.95E-112	0.68120724	1.41E-26
4.17E-15	-0.5367205	3.41E-15	0.78425292	8.68E-40	0.49538725	7.57E-13
1.07E-36	-0.473135	1.04E-11	0.80218979	7.53E-43	0.36200658	4.12E-07
3.15E-42	-0.2812546	0.00010526	0.61867647	6.33E-21	0.25348535	0.00049872
1.64E-21	-0.4305853	9.50E-10	0.74571603	4.20E-34	0.26817198	0.00022369
0.00025612	-0.373018	1.70E-07	0.42804749	1.22E-09	0.15005324	0.04148311
0.0136377	-0.1719351	0.01927477	0.56304862	7.29E-17	0.38282305	7.53E-08
1.44E-09	-0.2805556	0.0001097	0.71325774	4.68E-30	0.57498358	1.14E-17
3.32E-34	-0.5116342	9.87E-14	0.8333716	5.32E-49	0.45532301	7.43E-11
1.53E-27	-0.4080093	8.18E-09	0.84096014	1.08E-50	0.37929721	1.01E-07
6.23E-17	0.42505803	1.63E-09	-0.6775716	3.28E-26	-0.0638339	0.38801025
2.41E-33	-0.5649595	5.44E-17	0.89235736	3.90E-65	0.46392697	2.91E-11
1.29E-59	0.47250305	1.12E-11	-0.7552385	2.08E-35	-0.3607255	4.56E-07
7.71E-44	-0.5154945	5.99E-14	0.82635484	1.64E-47	0.45166242	1.10E-10
3.77E-14	0.52692778	1.31E-14	-0.8145399	3.80E-45	-0.5423365	1.54E-15

1.41E-10	-0.4126254	5.33E-09	0.74356951	8.12E-34		0.5793081	5.69E-18
1.51E-08	0.34677422	1.33E-06	-0.6603856	1.52E-24		-0.3921688	3.38E-08
1.12E-11	0.38779442	4.93E-08	-0.7774077	1.07E-38		-0.4271629	1.33E-09
1.71E-30	-0.5090237	1.38E-13	0.86178185	7.84E-56		0.69756721	2.70E-28
0.00552321	0.18311971	0.01259751	-0.3252717	6.25E-06		-0.2493045	0.00062135
4.37E-21	0.60133337	1.42E-19	-0.5663627	4.38E-17		-0.2683744	0.00022116
2.22E-33	0.60005691	1.77E-19	-0.9338493	1.22E-83		-0.7152566	2.74E-30
1.09E-15	-0.4433994	2.60E-10	0.5996168	1.91E-19		0.64696118	2.56E-23
1.47E-20	-0.422257	2.14E-09	0.63210031	4.97E-22		0.66973837	1.95E-25
0.08599808	0.29048464	6.04E-05	-0.5359475	3.80E-15		-0.1320094	0.07326139
1.73E-11	0.41018768	6.69E-09	-0.7481311	1.98E-34		-0.2480198	0.00066428
4.66E-34	0.5383765	2.70E-15	-0.7209388	5.81E-31		-0.5642062	6.11E-17
6.63E-50	-0.5760223	9.64E-18	0.95414187	8.56E-98		0.67384938	7.70E-26
1.19E-12	-0.5171349	4.83E-14	0.83802122	5.00E-50		0.58880853	1.20E-18
3.35E-27	-0.4197926	2.71E-09	0.83413819	3.62E-49		0.59235183	6.62E-19
3.14E-40	-0.4390187	4.08E-10	0.77348734	4.35E-38		0.4010326	1.54E-08
3.08E-09	-0.071192	0.33556021	0.61859323	6.43E-21		0.232027	0.00148293
5.79E-11	0.2550148	0.00045976	-0.512014	9.40E-14		-0.4260802	1.48E-09
1.78E-34	0.31872028	9.80E-06	-0.7265232	1.22E-31		-0.4939263	9.04E-13
0.00173517	-0.2634802	0.00029045	0.50880373	1.42E-13		0.50906441	1.37E-13
1.38E-20	-0.2485753	0.00064539	0.60053432	1.63E-19		0.27538111	0.00014835
1.71E-45	-0.4836545	3.08E-12	0.89158426	7.24E-65		0.44564214	2.06E-10
2.58E-17	-0.4650371	2.58E-11	0.74526391	4.83E-34		0.25363301	0.00049483
6.00E-30	0.62336645	2.64E-21	-0.7577135	9.28E-36		-0.5047646	2.37E-13
4.94E-23	0.24881455	0.00063741	-0.5761816	9.39E-18		-0.5528365	3.37E-16
0.24685639	-0.3852463	6.13E-08	0.50208822	3.31E-13		0.41719201	3.47E-09
7.23E-06	0.63181623	5.25E-22	-0.5120762	9.32E-14		-0.1574804	0.03228822
5.22E-23	0.48890608	1.66E-12	-0.8533494	1.18E-53		-0.5980876	2.49E-19
6.84E-11	-0.5075778	1.66E-13	0.77914535	5.72E-39		0.62722943	1.27E-21
6.71E-41	-0.3563936	6.39E-07	0.64293014	5.82E-23		0.52118462	2.83E-14
1.50E-49	0.46756513	1.95E-11	-0.7288084	6.37E-32		-0.4899513	1.46E-12
5.88E-33	0.54339969	1.33E-15	-0.7579051	8.72E-36		-0.8457038	8.52E-52
9.45E-45	-0.3405684	2.10E-06	0.67751602	3.33E-26		0.43622014	5.41E-10
1.04E-25	-0.4820247	3.73E-12	0.77891117	6.22E-39		0.44719927	1.75E-10
0.00120616	-0.3248636	6.43E-06	0.5783299	6.66E-18		0.56724794	3.82E-17
3.76E-06	-0.4319045	8.33E-10	0.71811526	1.26E-30		0.50424272	2.53E-13
4.05E-57	-0.4501728	1.28E-10	0.86525233	9.03E-57		0.60454401	8.11E-20
1.14E-10	-0.4077355	8.39E-09	0.74236884	1.17E-33		0.30343792	2.69E-05
4.74E-17	0.47063025	1.38E-11	-0.6946331	5.59E-28		-0.3311178	4.15E-06
3.59E-40	-0.317104	1.09E-05	0.65496149	4.84E-24		0.26117656	0.00032962
8.55E-28	0.5367611	3.39E-15	-0.6397189	1.11E-22		-0.3298624	4.54E-06
1.27E-32	-0.5204679	3.11E-14	0.91768731	2.79E-75		0.71325112	4.68E-30
5.77E-23	-0.4042072	1.16E-08	0.67458603	6.51E-26		0.20861903	0.00437582
0.85174169	-0.2869263	7.50E-05	0.3769913	1.23E-07		0.42499653	1.64E-09
1.00E-34	-0.5037831	2.68E-13	0.9231393	6.81E-78		0.7352415	9.86E-33
1.76E-14	0.3627007	3.90E-07	-0.7011562	1.09E-28		-0.5372843	3.15E-15

4.49E-18	0.37439128	1.52E-07	-0.753156	4.05E-35	-0.6615297	1.19E-24
4.77E-28	0.51179479	9.67E-14	-0.8557239	2.97E-54	-0.6783323	2.75E-26
4.41E-28	0.30092639	3.15E-05	-0.7192783	9.17E-31	-0.6239752	2.35E-21
3.09E-35	0.38173943	8.26E-08	-0.677213	3.57E-26	-0.3606221	4.60E-07
3.25E-29	0.29841458	3.70E-05	-0.5864102	1.79E-18	-0.1720047	0.01922516
2.59E-07	0.28545321	8.20E-05	-0.6216533	3.64E-21	-0.0265334	0.71996124
2.60E-46	0.48780045	1.89E-12	-0.7406222	1.99E-33	-0.540105	2.12E-15
1.57E-38	0.40273816	1.32E-08	-0.7869029	3.20E-40	-0.5767072	8.64E-18
2.20E-17	-0.4428864	2.74E-10	0.76786376	3.09E-37	0.73112835	3.27E-32
7.78E-28	0.38782526	4.92E-08	-0.7502178	1.03E-34	-0.7144745	3.38E-30
1.78E-14	0.34787991	1.22E-06	-0.7958781	9.75E-42	-0.5241075	1.92E-14
2.17E-38	0.37288445	1.72E-07	-0.8194744	4.11E-46	-0.797786	4.54E-42
4.90E-18	-0.3991488	1.82E-08	0.69833426	2.23E-28	0.4626605	3.35E-11
1.84E-51	-0.583454	2.90E-18	0.91192604	1.04E-72	0.4921818	1.12E-12
2.83E-34	-0.5454187	9.94E-16	0.63905671	1.27E-22	0.2374964	0.0011335
2.01E-21	-0.3706793	2.06E-07	0.72608662	1.38E-31	0.6218928	3.48E-21
2.09E-13	0.57969279	5.35E-18	-0.6255568	1.74E-21	-0.3947215	2.70E-08
7.26E-28	-0.61042	2.85E-20	0.79583794	9.91E-42	0.29333185	5.07E-05
2.41E-21	-0.6576934	2.71E-24	0.81713549	1.19E-45	0.45761748	5.80E-11
0.00063727	0.2758855	0.00014408	-0.4217357	2.25E-09	-0.3963533	2.34E-08
2.80E-05	-0.3641675	3.47E-07	0.62585916	1.65E-21	0.4464766	1.89E-10
1.21E-18	-0.3967788	2.25E-08	0.75744212	1.01E-35	0.26887179	0.00021505
0.00876105	-0.3004525	3.25E-05	0.39109164	3.71E-08	0.72185301	4.51E-31
1.71E-22	-0.2034656	0.00547297	0.59920411	2.06E-19	0.17830404	0.01517187
1.28E-17	-0.4418225	3.06E-10	0.63483907	2.91E-22	0.18095541	0.01370279
2.27E-39	-0.50544	2.17E-13	0.76672287	4.57E-37	0.38089644	8.86E-08
3.75E-20	0.15531107	0.03477511	-0.5404875	2.01E-15	-0.7271066	1.03E-31
8.32E-41	-0.5654945	5.01E-17	0.92670901	1.04E-79	0.59847301	2.33E-19
0.04052533	-0.2854656	8.19E-05	0.42904229	1.11E-09	0.19912748	0.00658096
9.00E-20	0.47270882	1.09E-11	-0.6903693	1.59E-27	-0.0916934	0.21448448
1.72E-29	-0.5040403	2.59E-13	0.91906243	6.38E-76	0.67505573	5.85E-26
6.12E-32	-0.4377901	4.62E-10	0.79039163	8.40E-41	0.46873633	1.71E-11
3.00E-30	-0.5079787	1.58E-13	0.83084596	1.86E-48	0.35234597	8.72E-07
1.90E-34	-0.3378959	2.55E-06	0.65547343	4.34E-24	0.1986056	0.00672691
3.78E-23	0.4791245	5.23E-12	-0.8918737	5.75E-65	-0.4031379	1.27E-08
7.42E-22	-0.2242447	0.00215093	0.57579278	1.00E-17	0.15857766	0.03108906
1.25E-31	0.28821356	6.94E-05	-0.7187865	1.05E-30	-0.4751699	8.25E-12
3.04E-11	0.41546155	4.09E-09	-0.6635444	7.65E-25	-0.6100481	3.05E-20
3.02E-12	-0.5068608	1.82E-13	0.76640234	5.10E-37	0.41579094	3.96E-09
3.16E-34	-0.4039055	1.19E-08	0.70437497	4.80E-29	0.2095536	0.00419951
1.30E-21	-0.5100325	1.21E-13	0.68954053	1.94E-27	0.44611019	1.96E-10
1.15E-19	-0.5024344	3.17E-13	0.66889584	2.35E-25	0.40851792	7.80E-09
8.11E-11	0.48911223	1.62E-12	-0.7814163	2.49E-39	-0.4487975	1.48E-10
5.56E-12	-0.4101126	6.74E-09	0.75713053	1.12E-35	0.72978473	4.82E-32
6.01E-19	-0.4298202	1.02E-09	0.63622629	2.22E-22	0.63877135	1.34E-22
7.13E-23	0.3571866	6.01E-07	-0.7935652	2.44E-41	-0.6023661	1.19E-19

7.09E-32	-0.4811617	4.13E-12	0.84638106	5.88E-52	0.44605351	1.98E-10
1.43E-05	-0.2749002	0.00015252	0.68720104	3.41E-27	0.48905989	1.63E-12
8.46E-36	-0.5470589	7.84E-16	0.74519567	4.93E-34	0.65924027	1.94E-24
0.00019745	-0.4715482	1.24E-11	0.49001977	1.45E-12	0.48709826	2.05E-12
1.35E-33	-0.5191305	3.72E-14	0.9409896	4.90E-88	0.7281023	7.79E-32
1.69E-28	-0.4403794	3.55E-10	0.72119518	5.41E-31	0.2467975	0.00070765
2.45E-64	-0.543617	1.29E-15	0.92323143	6.13E-78	0.50109453	3.75E-13
1.02E-15	-0.5259291	1.50E-14	0.80178295	8.91E-43	0.7821698	1.88E-39
1.04E-14	-0.5613571	9.43E-17	0.63130386	5.80E-22	0.2807349	0.00010854
5.89E-13	-0.4418071	3.07E-10	0.68674465	3.81E-27	0.87379472	3.39E-59
2.26E-26	0.3711331	1.99E-07	-0.6957757	4.21E-28	-0.3433291	1.72E-06
2.61E-05	-0.1795351	0.01447347	0.46993269	1.49E-11	0.07099685	0.33688971
3.86E-25	-0.3894063	4.29E-08	0.6628326	8.94E-25	0.06938975	0.34796823
1.88E-37	-0.520152	3.25E-14	0.70157408	9.82E-29	0.25872068	0.00037673
8.19E-34	0.47160365	1.24E-11	-0.8668274	3.32E-57	-0.3629108	3.84E-07
0.04885809	0.28519849	8.32E-05	-0.5410121	1.86E-15	-0.3242275	6.72E-06
1.06E-38	-0.3999676	1.70E-08	0.71391692	3.92E-30	0.27435703	0.00015737
7.53E-13	-0.0280175	0.70500687	0.68171092	1.25E-26	0.57607984	9.55E-18
1.56E-18	-0.3871875	5.20E-08	0.83923025	2.67E-50	0.37012688	2.15E-07
1.21E-35	-0.3052418	2.39E-05	0.58000471	5.08E-18	0.09940256	0.17824003
3.60E-19	-0.2497885	0.00060585	0.41552206	4.07E-09	-0.0477873	0.51831781
7.39E-31	-0.4099973	6.81E-09	0.72232719	3.95E-31	0.14585735	0.04758732
2.17E-16	0.79510528	1.33E-41	-0.6728139	9.74E-26	-0.5008067	3.88E-13
8.89E-06	0.36494741	3.27E-07	-0.5064137	1.92E-13	0.05724822	0.43892048
4.25E-25	0.81459981	3.70E-45	-0.7772967	1.12E-38	-0.4137831	4.79E-09
3.67E-16	0.39788847	2.04E-08	-0.7642478	1.06E-36	-0.6355138	2.55E-22
6.97E-42	-0.4455782	2.08E-10	0.75616033	1.54E-35	0.26490628	0.00026842
8.44E-37	-0.4440808	2.43E-10	0.92764199	3.35E-80	0.71345159	4.44E-30
2.56E-54	-0.5895242	1.06E-18	0.88354576	3.44E-62	0.65831103	2.37E-24
9.38E-06	-0.3851428	6.19E-08	0.53681465	3.37E-15	0.07953329	0.28186317
4.80E-16	0.30531479	2.38E-05	-0.6447787	4.00E-23	-0.6598134	1.72E-24
2.13E-07	-0.3939369	2.89E-08	0.59997285	1.80E-19	0.80453827	2.84E-43
2.86E-34	-0.4865952	2.18E-12	0.90760264	6.83E-71	0.62898054	9.08E-22
3.65E-38	0.38852126	4.63E-08	-0.7594438	5.26E-36	-0.3412561	2.00E-06
1.61E-29	0.23211108	0.00147689	-0.7000641	1.44E-28	-0.621685	3.62E-21
0.26435645	0.58167523	3.88E-18	-0.3644431	3.40E-07	-0.4718835	1.20E-11
6.31E-08	0.58708392	1.60E-18	-0.6412222	8.21E-23	-0.665673	4.80E-25
0.13011105	-0.3480479	1.21E-06	0.47649899	7.08E-12	0.25681181	0.00041759
4.88E-11	0.37850336	1.08E-07	-0.695755	4.24E-28	-0.4010799	1.53E-08
2.90E-31	-0.3481174	1.20E-06	0.66316151	8.32E-25	0.32732714	5.42E-06
0.00275126	-0.4075348	8.54E-09	0.58986292	1.01E-18	0.07607857	0.30335781
1.39E-45	-0.5237477	2.01E-14	0.87304302	5.64E-59	0.66471174	5.93E-25
2.16E-40	0.3042131	2.55E-05	-0.5677787	3.52E-17	-0.3734415	1.64E-07
8.80E-27	0.1816716	0.01332799	-0.5596656	1.22E-16	-0.2112641	0.00389329
5.14E-09	-0.3993605	1.79E-08	0.6478196	2.15E-23	0.68053359	1.65E-26
0.00059817	0.47611167	7.40E-12	-0.4827745	3.42E-12	-0.3382514	2.49E-06

8.00E-24	-0.1215002	0.09945745	0.55286546	3.36E-16	0.12118639	0.10034094
3.89E-70	-0.479952	4.75E-12	0.89944063	1.08E-67	0.60387507	9.12E-20
6.49E-12	-0.3836234	7.04E-08	0.73347905	1.65E-32	0.16038325	0.02919823
7.40E-19	0.25188422	0.00054277	-0.5225786	2.35E-14	0.10818822	0.14268885
7.14E-55	-0.5449649	1.06E-15	0.82515684	2.91E-47	0.43082724	9.27E-10
1.58E-17	-0.4369274	5.04E-10	0.77683179	1.32E-38	0.58856732	1.25E-18
8.74E-46	0.32493531	6.40E-06	-0.7851429	6.22E-40	-0.5276145	1.19E-14
4.35E-33	-0.5161609	5.49E-14	0.91343716	2.30E-73	0.59047391	9.08E-19
3.00E-20	-0.2723857	0.00017619	0.78757337	2.48E-40	0.47840371	5.69E-12
8.44E-24	-0.4604076	4.29E-11	0.81838797	6.75E-46	0.47536443	8.07E-12
4.38E-07	0.41242099	5.44E-09	-0.6097073	3.24E-20	-0.6268215	1.37E-21
0.49577619	0.1292566	0.07951153	-0.2568219	0.00041736	-0.3993267	1.80E-08
1.10E-18	0.68314008	8.96E-27	-0.7918377	4.79E-41	-0.5236261	2.05E-14
6.10E-34	0.4531466	9.37E-11	-0.8480206	2.39E-52	-0.667007	3.57E-25
2.91E-15	0.33471456	3.21E-06	-0.6516869	9.63E-24	-0.6898174	1.82E-27
2.09E-09	-0.1897308	0.00969096	0.40931361	7.25E-09	0.12197474	0.09813305
2.74E-35	-0.4722348	1.15E-11	0.88744254	1.84E-63	0.36632038	2.93E-07
5.88E-13	-0.2860836	7.89E-05	0.6782414	2.81E-26	0.08805562	0.23330637
0.00818205	0.2253661	0.00204024	-0.4213546	2.33E-09	0.17162769	0.01949506
4.82E-24	-0.3507001	9.88E-07	0.67663518	4.07E-26	0.76316498	1.53E-36
0.00448196	-0.2418737	0.00091013	0.38400624	6.82E-08	0.47998227	4.74E-12
7.68E-22	0.335619	3.01E-06	-0.6370737	1.88E-22	-0.6349138	2.87E-22
4.01E-19	0.36330255	3.72E-07	-0.7910495	6.51E-41	-0.6640819	6.80E-25
1.10E-13	-0.5235273	2.07E-14	0.74849994	1.77E-34	0.46604774	2.30E-11
4.39E-20	-0.4417348	3.09E-10	0.80322683	4.90E-43	0.46899734	1.66E-11
5.65E-42	-0.5940286	4.99E-19	0.91177988	1.20E-72	0.58465963	2.38E-18
2.82E-16	-0.3447656	1.54E-06	0.53537571	4.11E-15	0.10719656	0.14640799
1.18E-11	0.38975848	4.16E-08	-0.7864327	3.82E-40	-0.4544376	8.16E-11
0.00139644	0.29891536	3.58E-05	-0.5480464	6.80E-16	-0.1620104	0.02757921
2.98E-15	0.42752734	1.28E-09	-0.8332501	5.65E-49	-0.4813606	4.03E-12
0.05576641	-0.3978815	2.04E-08	0.5024452	3.16E-13	0.54492777	1.07E-15
8.05E-08	-0.3997609	1.73E-08	0.72615434	1.35E-31	0.27615344	0.00014187
1.07E-30	-0.5472327	7.65E-16	0.63702507	1.89E-22	0.19129964	0.00909517
1.29E-78	-0.4061196	9.72E-09	0.8698052	4.84E-58	0.60181809	1.31E-19
8.91E-57	-0.5916495	7.45E-19	0.96588878	2.55E-109	0.69242412	9.63E-28
4.59E-14	-0.4192121	2.87E-09	0.75370334	3.40E-35	0.40595879	9.87E-09
2.15E-22	-0.4751599	8.26E-12	0.85349366	1.08E-53	0.78399386	9.56E-40
2.55E-76	-0.5495544	5.46E-16	0.87243835	8.46E-59	0.54941286	5.57E-16
1.47E-20	0.30274006	2.81E-05	-0.690546	1.52E-27	-0.7162311	2.10E-30
1.82E-23	0.48785956	1.88E-12	-0.8524816	1.94E-53	-0.5671195	3.90E-17
3.77E-33	-0.481801	3.83E-12	0.8639312	2.07E-56	0.56622467	4.48E-17
4.69E-37	-0.2287281	0.00173873	0.75294758	4.33E-35	0.40722431	8.79E-09
7.54E-49	0.49276372	1.04E-12	-0.9161867	1.36E-74	-0.7489178	1.55E-34
4.09E-18	-0.3279725	5.18E-06	0.80189208	8.52E-43	0.43271741	7.68E-10
2.23E-21	-0.4091474	7.36E-09	0.75716264	1.11E-35	0.75320219	3.99E-35
2.46E-21	-0.4147474	4.37E-09	0.63526012	2.68E-22	0.19299444	0.00848819

0.00159185	0.34923895	1.10E-06	-0.4417455	3.08E-10	-0.4780913	5.90E-12
2.54E-38	-0.6355069	2.55E-22	0.9537082	1.98E-97	0.65468817	5.13E-24
2.88E-10	-0.3862005	5.65E-08	0.73967344	2.64E-33	0.59163446	7.47E-19
5.38E-13	0.46907722	1.64E-11	-0.7708875	1.09E-37	-0.5679789	3.41E-17
5.69E-34	0.53210611	6.47E-15	-0.7255316	1.61E-31	-0.4307342	9.36E-10
6.70E-22	0.27639509	0.00013989	-0.6392867	1.21E-22	-0.5080024	1.57E-13
1.94E-12	0.34344891	1.70E-06	-0.7667751	4.49E-37	-0.4044123	1.14E-08
1.26E-28	0.30281162	2.80E-05	-0.6082462	4.21E-20	-0.1092895	0.13864339
4.55E-07	0.54339567	1.33E-15	-0.629913	7.59E-22	-0.0136546	0.8536432
3.46E-15	0.58232131	3.49E-18	-0.8095514	3.37E-44	-0.5150419	6.35E-14
5.13E-08	-0.3421375	1.87E-06	0.58850901	1.26E-18	0.01711677	0.81711885
2.18E-08	-0.2510506	0.00056709	0.56359408	6.71E-17	0.07254318	0.32644489
7.29E-15	-0.3222893	7.68E-06	0.79423849	1.87E-41	0.5514337	4.15E-16
5.76E-17	0.40389349	1.19E-08	-0.7116978	7.08E-30	-0.6418275	7.27E-23
5.63E-24	-0.441575	3.14E-10	0.84421957	1.90E-51	0.71748529	1.50E-30
3.59E-23	-0.4254954	1.56E-09	0.8158006	2.17E-45	0.44363682	2.54E-10
3.98E-59	-0.5377472	2.95E-15	0.92235904	1.66E-77	0.81768782	9.27E-46
2.80E-22	0.48611243	2.31E-12	-0.7003351	1.34E-28	-0.6133835	1.67E-20
6.54E-36	-0.3948044	2.68E-08	0.71353944	4.34E-30	0.2593932	0.00036325
3.99E-33	0.43283747	7.59E-10	-0.7142187	3.62E-30	-0.176218	0.0164226
2.87E-07	0.38106969	8.73E-08	-0.4596695	4.64E-11	-0.5106751	1.12E-13
4.18E-51	-0.3871902	5.19E-08	0.76600929	5.83E-37	0.33635031	2.86E-06
1.23E-19	0.4738553	9.58E-12	-0.5529838	3.30E-16	-0.3883769	4.69E-08
3.47E-40	-0.4694047	1.58E-11	0.91722116	4.58E-75	0.44500747	2.20E-10
2.06E-34	-0.5314899	7.04E-15	0.77314219	4.92E-38	0.4541383	8.43E-11
2.69E-24	-0.4797863	4.85E-12	0.8308772	1.83E-48	0.57384136	1.36E-17
1.09E-27	0.46957821	1.55E-11	-0.8755434	1.03E-59	-0.7570734	1.14E-35
1.66E-22	0.4692921	1.60E-11	-0.7590019	6.09E-36	-0.746357	3.44E-34
1.50E-29	0.19935729	0.0065176	-0.5156274	5.88E-14	-0.1229518	0.09545014
3.37E-13	-0.4146572	4.41E-09	0.78747439	2.57E-40	0.75056475	9.23E-35
6.85E-24	0.22913723	0.00170495	-0.5625486	7.87E-17	-0.1905587	0.00937239
6.11E-18	-0.5068744	1.81E-13	0.86197752	6.95E-56	0.76001571	4.36E-36
2.33E-46	-0.5276317	1.19E-14	0.95451422	4.13E-98	0.75977168	4.72E-36
1.48E-18	-0.3295786	4.63E-06	0.76697929	4.19E-37	0.67076455	1.55E-25
1.46E-14	-0.4978266	5.61E-13	0.85434256	6.64E-54	0.59927064	2.03E-19
0.00049558	-0.4360414	5.51E-10	0.48773646	1.90E-12	0.68202015	1.17E-26
9.54E-18	-0.4193909	2.82E-09	0.65798361	2.55E-24	0.21993807	0.00262863
9.12E-18	-0.4858634	2.38E-12	0.82936281	3.85E-48	0.80240949	6.88E-43
4.40E-20	0.30488785	2.45E-05	-0.6634337	7.84E-25	-0.1523398	0.03844303
0.00032056	0.21327194	0.00355961	-0.4116328	5.85E-09	-0.5449015	1.07E-15
1.22E-25	0.46225616	3.50E-11	-0.8544908	6.09E-54	-0.7035236	5.97E-29
6.46E-41	-0.562278	8.20E-17	0.91090934	2.84E-72	0.7516987	6.45E-35
2.90E-15	-0.4864243	2.22E-12	0.78261525	1.60E-39	0.82644808	1.57E-47
7.60E-11	0.22473939	0.00210144	-0.6144357	1.38E-20	-0.7356524	8.74E-33
2.54E-18	-0.4167646	3.62E-09	0.82482632	3.40E-47	0.80384238	3.80E-43
3.45E-40	0.30303322	2.76E-05	-0.7094736	1.27E-29	-0.5063459	1.94E-13

2.26E-43	-0.5429656	1.41E-15	0.88651288	3.73E-63	0.59529116	4.02E-19
5.36E-22	0.30854198	1.93E-05	-0.5347593	4.48E-15	-0.173837	0.01795916
0.00067665	0.26520348	0.00026403	-0.4559482	6.94E-11	-0.17872	0.01493267
2.37E-33	-0.3185159	9.94E-06	0.66526975	5.24E-25	0.1984904	0.00675951
1.59E-30	-0.5744681	1.23E-17	0.83106571	1.67E-48	0.74760833	2.33E-34
7.43E-35	-0.3875495	5.04E-08	0.61643312	9.57E-21	0.16613568	0.02381482
6.60E-06	-0.4188466	2.97E-09	0.55539427	2.31E-16	0.09507915	0.19796552
6.68E-17	-0.2639505	0.00028301	0.46419705	2.83E-11	0.36928766	2.31E-07
2.31E-64	-0.5532187	3.19E-16	0.90265897	6.38E-69	0.48646035	2.21E-12
1.98E-29	-0.6580891	2.49E-24	0.83148052	1.36E-48	0.42283966	2.02E-09
6.83E-29	-0.5244216	1.84E-14	0.93848274	1.97E-86	0.62078812	4.28E-21
1.81E-17	-0.5102388	1.18E-13	0.87419086	2.59E-59	0.49136494	1.23E-12
8.90E-36	-0.4184311	3.09E-09	0.70760592	2.08E-29	0.22360031	0.00221698
2.45E-24	-0.4805034	4.46E-12	0.79076268	7.28E-41	0.63357083	3.73E-22
1.13E-27	0.43214976	8.13E-10	-0.7352398	9.87E-33	-0.1881444	0.01032824
7.23E-24	-0.3941941	2.83E-08	0.82010615	3.08E-46	0.43782353	4.60E-10
1.28E-17	-0.5311603	7.37E-15	0.79050619	8.04E-41	0.36647152	2.89E-07
9.02E-37	-0.5038721	2.65E-13	0.73608582	7.69E-33	0.18500672	0.01169853
4.04E-32	0.36391145	3.55E-07	-0.7049928	4.09E-29	-0.6260364	1.59E-21
7.24E-06	-0.0092706	0.90033193	0.5075938	1.65E-13	0.63200784	5.06E-22
1.73E-06	-0.4577518	5.72E-11	0.71093309	8.67E-30	0.53572971	3.92E-15
1.85E-21	-0.4893111	1.58E-12	0.8390678	2.91E-50	0.81265356	8.75E-45
2.75E-28	0.41136759	6.00E-09	-0.7817352	2.21E-39	-0.5805499	4.66E-18
1.58E-27	-0.2556101	0.00044537	0.75391921	3.17E-35	0.57113346	2.09E-17
1.65E-33	0.54213346	1.59E-15	-0.8305515	2.15E-48	-0.6616346	1.16E-24
3.29E-14	0.44506335	2.19E-10	-0.6243349	2.20E-21	-0.7397537	2.58E-33
2.39E-32	0.20085271	0.00611838	-0.6461291	3.04E-23	-0.5193699	3.60E-14
7.06E-15	-0.595387	3.96E-19	0.68214716	1.13E-26	0.31541621	1.22E-05
2.79E-22	0.25352072	0.00049779	-0.5964432	3.31E-19	-0.2877535	7.14E-05
2.75E-20	0.32430814	6.68E-06	-0.7041503	5.08E-29	-0.1890698	0.00995217
1.11E-14	0.50374412	2.69E-13	-0.571188	2.07E-17	-0.150627	0.04070186
1.99E-09	0.23334279	0.00139088	-0.4967276	6.42E-13	-0.5614324	9.32E-17
5.35E-37	-0.5933844	5.56E-19	0.94226099	7.08E-89	0.67417129	7.16E-26
2.32E-32	-0.575317	1.08E-17	0.91308912	3.26E-73	0.52603349	1.48E-14
3.29E-27	0.56374737	6.55E-17	-0.8383621	4.20E-50	-0.6607618	1.40E-24
1.87E-33	-0.5300586	8.57E-15	0.92559864	3.90E-79	0.76063863	3.54E-36
0.03569838	0.31159927	1.58E-05	-0.5524983	3.55E-16	-0.4170744	3.51E-09
4.37E-40	0.60515889	7.27E-20	-0.646045	3.09E-23	-0.4758423	7.64E-12
1.22E-06	-0.285335	8.26E-05	0.42172013	2.25E-09	0.02006974	0.78627173
1.34E-41	-0.412704	5.29E-09	0.65442926	5.42E-24	0.22329784	0.00224861
5.59E-30	0.46519744	2.53E-11	-0.862982	3.74E-56	-0.4069515	9.01E-09
0.59507215	0.3100689	1.75E-05	-0.3945213	2.75E-08	0.14324718	0.05174967
7.08E-55	-0.5354114	4.09E-15	0.93565956	1.05E-84	0.67045734	1.66E-25
5.91E-53	-0.4210007	2.42E-09	0.92891095	7.04E-81	0.56853151	3.13E-17
3.32E-05	0.22881429	0.00173156	-0.5954091	3.94E-19	-0.2938809	4.90E-05
0.78792884	-0.1960075	0.00749731	0.3740484	1.56E-07	0.46654412	2.18E-11

1.43E-07	-0.3510772	9.60E-07	0.67361927	8.12E-26	0.7181709	1.24E-30
1.17E-23	-0.6354417	2.59E-22	0.90579689	3.69E-70	0.71079749	8.99E-30
6.23E-09	0.3527944	8.42E-07	-0.7605531	3.65E-36	-0.3359543	2.94E-06
2.81E-34	-0.2759447	0.00014359	0.75438212	2.73E-35	0.71170257	7.07E-30
7.57E-14	-0.5324823	6.14E-15	0.85074825	5.19E-53	0.5599585	1.17E-16
7.90E-13	-0.557428	1.71E-16	0.79102431	6.58E-41	0.32816441	5.11E-06
1.24E-29	-0.3933861	3.04E-08	0.72792312	8.20E-32	0.72719387	1.01E-31
9.61E-37	0.29509815	4.55E-05	-0.6112885	2.44E-20	-0.4270899	1.34E-09
5.20E-39	0.68241851	1.06E-26	-0.7493499	1.35E-34	-0.5247693	1.75E-14
0.28278783	0.26346245	0.00029074	-0.330478	4.34E-06	-0.4694736	1.57E-11
6.95E-38	-0.4292405	1.08E-09	0.64390983	4.77E-23	0.25774804	0.00039706
1.43E-48	-0.503591	2.74E-13	0.77216837	6.93E-38	0.34896932	1.13E-06
2.06E-07	-0.359608	4.98E-07	0.73008301	4.42E-32	0.49792715	5.54E-13
2.46E-40	-0.4220939	2.17E-09	0.8181835	7.40E-46	0.38887415	4.49E-08
1.33E-18	-0.362453	3.98E-07	0.79550358	1.13E-41	0.50357732	2.75E-13
3.03E-50	-0.5031249	2.91E-13	0.83397725	3.92E-49	0.46798421	1.86E-11
1.42E-37	-0.2259868	0.00198122	0.65135148	1.03E-23	0.37018659	2.14E-07
2.00E-33	-0.6114076	2.39E-20	0.91524218	3.63E-74	0.58896379	1.17E-18
5.72E-07	-0.3461879	1.39E-06	0.59673684	3.14E-19	0.80747526	8.21E-44
4.91E-18	0.60633033	5.91E-20	-0.6914273	1.23E-27	-0.2828765	9.56E-05
6.26E-27	-0.4572662	6.03E-11	0.8713552	1.74E-58	0.40641522	9.46E-09
5.56E-15	-0.4492168	1.42E-10	0.78760736	2.45E-40	0.36204467	4.11E-07
6.77E-26	-0.3897614	4.16E-08	0.56748133	3.69E-17	0.11653264	0.11418498
2.94E-14	-0.451483	1.12E-10	0.75086324	8.40E-35	0.40093266	1.56E-08
1.44E-44	-0.3165812	1.13E-05	0.69929214	1.75E-28	0.21547354	0.00322357
3.86E-05	-0.386544	5.49E-08	0.61541292	1.15E-20	0.6355418	2.54E-22
5.18E-31	0.44533074	2.13E-10	-0.8200433	3.17E-46	-0.6217947	3.54E-21
1.29E-13	0.58885366	1.19E-18	-0.5817624	3.82E-18	-0.2858041	8.03E-05
5.80E-21	-0.4888432	1.67E-12	0.82923761	4.09E-48	0.73683765	6.16E-33
9.33E-42	-0.2297269	0.00165733	0.76085082	3.30E-36	0.36172265	4.22E-07
1.08E-18	-0.4920352	1.14E-12	0.83047676	2.23E-48	0.43491737	6.17E-10
1.77E-22	-0.220941	0.00250951	0.52824655	1.10E-14	0.02882328	0.69693382
2.97E-29	0.30194566	2.95E-05	-0.7225815	3.69E-31	-0.4082079	8.03E-09
5.88E-28	-0.5530197	3.28E-16	0.9160914	1.50E-74	0.7144048	3.44E-30
1.05E-09	0.29471422	4.66E-05	-0.5814054	4.05E-18	-0.4209399	2.43E-09
0.02412211	0.27999948	0.00011334	-0.1175039	0.11117891	-0.1489032	0.04308687
5.42E-20	-0.0191255	0.79610225	0.59422305	4.83E-19	0.65307712	7.20E-24
4.09E-20	0.54937543	5.60E-16	-0.5548298	2.51E-16	-0.1880542	0.01036558
1.49E-07	-0.4229679	2.00E-09	0.68570817	4.87E-27	0.66065409	1.43E-24
8.68E-24	-0.4852855	2.55E-12	0.75974744	4.76E-36	0.58101629	4.32E-18
1.22E-47	-0.4700474	1.47E-11	0.88599781	5.52E-63	0.70960904	1.23E-29
1.49E-29	0.31896996	9.64E-06	-0.6330781	4.11E-22	-0.3407357	2.08E-06
5.83E-18	0.53605063	3.74E-15	-0.7062317	2.97E-29	-0.3737922	1.60E-07
4.21E-35	-0.4041495	1.16E-08	0.60488387	7.64E-20	0.30457678	2.50E-05
3.90E-07	0.46445426	2.75E-11	-0.482918	3.36E-12	-0.4549608	7.72E-11
1.43E-31	-0.353307	8.10E-07	0.77358139	4.21E-38	0.26542509	0.0002608

1.27E-32	-0.4093354	7.24E-09	0.58903126	1.16E-18	0.19676822	0.00726398
5.59E-20	0.27190606	0.00018108	-0.6109171	2.61E-20	-0.5453684	1.00E-15
2.07E-40	0.45285945	9.66E-11	-0.762933	1.65E-36	-0.5229642	2.23E-14
1.37E-12	0.42619445	1.46E-09	-0.61775	7.51E-21	-0.668215	2.73E-25
1.17E-09	0.30435281	2.53E-05	-0.5811145	4.25E-18	-0.5246954	1.77E-14
4.12E-37	0.48730034	2.00E-12	-0.8408777	1.13E-50	-0.5787269	6.25E-18
6.36E-27	0.83501401	2.33E-49	-0.7631106	1.55E-36	-0.4768063	6.84E-12
3.22E-20	0.21287521	0.00362342	-0.5754834	1.05E-17	-0.6413815	7.95E-23
5.17E-15	0.33045888	4.35E-06	-0.5624076	8.04E-17	-0.5388828	2.52E-15
5.75E-29	0.45282592	9.69E-11	-0.8489835	1.40E-52	-0.7155339	2.54E-30
2.87E-22	0.55058031	4.70E-16	-0.6929561	8.45E-28	-0.8062422	1.39E-43
7.48E-21	-0.6054802	6.87E-20	0.6149514	1.26E-20	0.41639773	3.74E-09
2.46E-15	-0.5185031	4.04E-14	0.81474498	3.47E-45	0.62130794	3.88E-21
6.96E-43	0.48436575	2.84E-12	-0.8303345	2.39E-48	-0.6497029	1.45E-23
4.36E-29	-0.3956139	2.50E-08	0.73106908	3.33E-32	0.22133687	0.00246385
2.34E-49	0.61648536	9.48E-21	-0.8318733	1.12E-48	-0.579888	5.18E-18
4.52E-10	0.35361356	7.91E-07	-0.5557419	2.19E-16	-0.7452131	4.90E-34
3.65E-20	-0.3421921	1.87E-06	0.74703902	2.79E-34	0.26317687	0.00029535
1.34E-37	-0.2197435	0.00265233	0.78668111	3.48E-40	0.3396437	2.25E-06
1.40E-25	-0.3168406	1.11E-05	0.52810917	1.12E-14	0.04612493	0.53299109
7.49E-34	0.59365018	5.32E-19	-0.8117608	1.29E-44	-0.471529	1.25E-11
2.53E-12	-0.4481147	1.59E-10	0.78941373	1.23E-40	0.31231204	1.51E-05
9.77E-35	-0.6314652	5.62E-22	0.95607466	1.82E-99	0.65421217	5.67E-24
0.03798945	-0.2853029	8.27E-05	0.48366239	3.08E-12	0.52234166	2.43E-14
5.60E-27	-0.5186325	3.97E-14	0.89999633	6.65E-68	0.61466609	1.32E-20
3.19E-22	-0.462274	3.49E-11	0.81325732	6.71E-45	0.79788727	4.36E-42
5.11E-23	-0.324233	6.72E-06	0.7490832	1.47E-34	0.17510059	0.01712883
8.93E-29	-0.3374319	2.64E-06	0.77338369	4.52E-38	0.55238354	3.61E-16
9.09E-27	0.56746173	3.70E-17	-0.5549912	2.45E-16	-0.2064913	0.00480227
6.20E-43	-0.3984122	1.95E-08	0.71931427	9.08E-31	0.33609884	2.91E-06
9.80E-31	0.27832665	0.00012502	-0.5245112	1.82E-14	-0.3997523	1.73E-08
3.87E-05	-0.3897591	4.16E-08	0.65968608	1.77E-24	0.5258902	1.51E-14
2.51E-50	0.65586049	4.00E-24	-0.8089941	4.29E-44	-0.5490478	5.88E-16
3.16E-14	-0.315213	1.24E-05	0.74858988	1.72E-34	0.41280689	5.24E-09
1.01E-30	-0.5356208	3.98E-15	0.80419105	3.28E-43	0.56353802	6.77E-17
1.35E-18	0.5190924	3.74E-14	-0.6804556	1.68E-26	-0.7194723	8.70E-31
2.89E-55	-0.5452366	1.02E-15	0.92247436	1.45E-77	0.71574144	2.40E-30
1.40E-11	0.37459337	1.50E-07	-0.5454376	9.91E-16	-0.326664	5.68E-06
4.45E-09	-0.4408994	3.36E-10	0.76640329	5.10E-37	0.65059913	1.21E-23
4.08E-25	-0.4409211	3.36E-10	0.81917272	4.72E-46	0.76073406	3.43E-36
3.55E-31	-0.5549174	2.48E-16	0.88222538	9.07E-62	0.64845877	1.88E-23
7.85E-42	-0.5618988	8.69E-17	0.92435271	1.68E-78	0.69485142	5.30E-28
4.97E-39	-0.5676594	3.59E-17	0.68773052	3.00E-27	0.31915762	9.51E-06
6.48E-17	-0.4320628	8.20E-10	0.8269327	1.25E-47	0.46209628	3.56E-11
8.79E-39	-0.4116604	5.83E-09	0.74466032	5.81E-34	0.23458334	0.0013089
4.77E-22	0.43348591	7.12E-10	-0.8631727	3.32E-56	-0.5489216	5.99E-16

1.31E-20	0.50547357	2.16E-13	-0.8023239	7.13E-43	-0.5746272	1.20E-17
8.29E-28	0.46049381	4.25E-11	-0.870887	2.37E-58	-0.6793648	2.17E-26
1.71E-44	-0.5602589	1.11E-16	0.85218872	2.29E-53	0.41350973	4.91E-09
1.11E-33	-0.5019302	3.37E-13	0.91745246	3.59E-75	0.5765201	8.90E-18
2.62E-19	0.41375628	4.80E-09	-0.7745532	2.98E-38	-0.3752151	1.42E-07
2.38E-25	-0.2064817	0.00480429	0.5804333	4.74E-18	0.14985291	0.04175882
3.22E-36	0.3881925	4.77E-08	-0.755861	1.70E-35	-0.2933095	5.08E-05
4.33E-25	0.51932435	3.62E-14	-0.6016797	1.34E-19	-0.1841067	0.01212003
4.91E-21	0.67876071	2.49E-26	-0.8153043	2.71E-45	-0.6790037	2.36E-26
1.84E-20	-0.315836	1.19E-05	0.70708224	2.38E-29	0.19805282	0.00688464
8.78E-17	0.35972932	4.93E-07	-0.6480987	2.03E-23	-0.7486548	1.68E-34
0.00054794	-0.5235872	2.06E-14	0.55565974	2.22E-16	0.23420062	0.00133371
0.07717802	-0.3779029	1.14E-07	0.54108715	1.84E-15	0.45010664	1.29E-10
1.43E-29	-0.5059396	2.04E-13	0.8742246	2.53E-59	0.52459211	1.80E-14
1.32E-09	0.25806497	0.00039033	-0.5438494	1.24E-15	-0.6126954	1.89E-20
9.97E-38	0.3944027	2.78E-08	-0.7752213	2.35E-38	-0.397439	2.12E-08
4.16E-18	-0.510237	1.18E-13	0.86723616	2.56E-57	0.7496168	1.24E-34
3.11E-28	-0.560328	1.10E-16	0.91595067	1.74E-74	0.77543103	2.18E-38
4.31E-10	-0.4385943	4.25E-10	0.73285462	1.98E-32	0.6534093	6.71E-24
2.60E-47	-0.3932939	3.06E-08	0.87385732	3.25E-59	0.82210526	1.22E-46
1.30E-26	-0.4991506	4.77E-13	0.88450565	1.69E-62	0.6769193	3.82E-26
1.17E-36	-0.4545653	8.05E-11	0.6911221	1.32E-27	0.3121226	1.52E-05
6.94E-28	0.65225918	8.54E-24	-0.5943723	4.70E-19	-0.3528052	8.42E-07
4.48E-08	-0.3571496	6.03E-07	0.67414819	7.20E-26	0.42513256	1.62E-09
7.97E-06	0.28133701	0.00010475	-0.6329684	4.20E-22	-0.4524944	1.00E-10
8.09E-10	0.3573291	5.94E-07	-0.6754097	5.40E-26	-0.6688913	2.35E-25
3.33E-11	-0.4290658	1.10E-09	0.39528875	2.57E-08	0.09219069	0.21199819
2.71E-08	0.54060127	1.98E-15	-0.5850806	2.22E-18	-0.6941778	6.26E-28
1.74E-09	-0.5796616	5.37E-18	0.72021186	7.10E-31	0.60179965	1.31E-19
7.75E-25	-0.5228331	2.27E-14	0.91207536	8.98E-73	0.73920697	3.04E-33
2.57E-29	0.38627927	5.62E-08	-0.8612924	1.06E-55	-0.5365792	3.48E-15
2.68E-07	-0.2867979	7.56E-05	0.66218114	1.03E-24	0.64509389	3.75E-23
2.52E-11	-0.4271155	1.34E-09	0.59255041	6.40E-19	0.874566	2.01E-59
2.01E-16	-0.4458947	2.01E-10	0.83992835	1.86E-50	0.70247009	7.82E-29
5.62E-31	0.46582502	2.36E-11	-0.8536979	9.64E-54	-0.5030125	2.95E-13
4.52E-21	0.35563569	6.77E-07	-0.7442878	6.51E-34	-0.6309648	6.19E-22
0.00276379	-0.2530338	0.0005108	0.60613651	6.12E-20	0.50737736	1.70E-13
3.28E-11	0.73497075	1.07E-32	-0.6696214	2.00E-25	-0.39237	3.32E-08
1.20E-09	0.50534412	2.20E-13	-0.7145829	3.28E-30	-0.086389	0.24230694
1.33E-31	-0.452588	9.94E-11	0.91383919	1.53E-73	0.73215371	2.43E-32
4.86E-17	0.51532289	6.12E-14	-0.8323844	8.70E-49	-0.5088167	1.42E-13
2.58E-35	0.64209848	6.88E-23	-0.8242647	4.44E-47	-0.3313325	4.09E-06
7.63E-24	-0.2651473	0.00026485	0.4138787	4.74E-09	0.18957374	0.00975247
2.10E-11	0.36114083	4.41E-07	-0.7043725	4.80E-29	-0.5637079	6.59E-17
9.26E-19	0.48429501	2.86E-12	-0.7667457	4.54E-37	-0.3098389	1.77E-05
6.73E-31	-0.45281	9.71E-11	0.89153574	7.53E-65	0.5982349	2.43E-19

1.53E-11	0.32355943	7.04E-06	-0.6431053	5.62E-23	-0.3918034	3.49E-08
1.39E-32	0.52030636	3.18E-14	-0.5592128	1.30E-16	-0.5674215	3.72E-17
9.79E-09	0.38009977	9.47E-08	-0.7822809	1.81E-39	-0.3802084	9.39E-08
1.17E-13	0.38646911	5.53E-08	-0.6335223	3.77E-22	-0.5745083	1.23E-17
6.54E-32	-0.5983475	2.38E-19	0.94611837	1.51E-91	0.608331	4.15E-20
1.40E-17	0.41866578	3.02E-09	-0.7806669	3.28E-39	-0.6163157	9.78E-21
3.48E-17	0.32478958	6.47E-06	-0.4604728	4.26E-11	0.07580594	0.3050989
5.01E-13	0.44053014	3.49E-10	-0.6734205	8.49E-26	-0.2965446	4.16E-05
2.75E-20	-0.3505227	1.00E-06	0.53834754	2.72E-15	0.07466402	0.31246273
9.83E-13	-0.2220642	0.00238193	0.59078823	8.61E-19	0.22839747	0.00176647
1.40E-11	-0.4357608	5.67E-10	0.6969987	3.11E-28	0.5399092	2.18E-15
4.56E-29	-0.3860261	5.74E-08	0.6656351	4.84E-25	0.6470733	2.50E-23
6.54E-10	-0.0577419	0.43497669	0.50984486	1.24E-13	0.03354132	0.6503711
1.39E-10	-0.4614175	3.84E-11	0.56569688	4.86E-17	0.64890205	1.72E-23
6.94E-22	-0.3473844	1.27E-06	0.77230616	6.60E-38	0.36813207	2.53E-07
3.03E-27	-0.5029575	2.97E-13	0.84197995	6.30E-51	0.51574143	5.80E-14
5.45E-06	-0.4546597	7.97E-11	0.52184637	2.59E-14	0.72751195	9.22E-32
2.29E-27	0.39653378	2.30E-08	-0.7158108	2.36E-30	-0.6814263	1.34E-26
6.33E-09	0.33940912	2.29E-06	-0.5811705	4.21E-18	-0.6271997	1.28E-21
6.12E-28	0.07105602	0.33648613	-0.644529	4.21E-23	-0.328782	4.89E-06
3.14E-24	0.58863468	1.23E-18	-0.803795	3.87E-43	-0.2687444	0.0002166
2.19E-13	-0.3743275	1.53E-07	0.64349089	5.19E-23	0.79355302	2.45E-41
0.00128336	-0.325135	6.31E-06	0.63904252	1.27E-22	0.3267131	5.66E-06
6.50E-43	-0.4228291	2.03E-09	0.64374537	4.93E-23	0.24747334	0.00068336
6.88E-45	-0.4828243	3.40E-12	0.87473422	1.79E-59	0.44191733	3.03E-10
9.56E-24	-0.4601117	4.43E-11	0.86189943	7.29E-56	0.64521619	3.66E-23
1.11E-29	0.18204188	0.01313775	-0.5201115	3.26E-14	-0.3157668	1.20E-05
9.73E-31	-0.564597	5.75E-17	0.81076022	2.00E-44	0.49602364	7.00E-13
1.54E-69	-0.5220495	2.52E-14	0.86308525	3.51E-56	0.49556012	7.41E-13
7.85E-25	0.39978522	1.72E-08	-0.7204333	6.68E-31	-0.3105809	1.69E-05
2.75E-32	-0.4191393	2.89E-09	0.5561944	2.05E-16	0.28750261	7.25E-05
9.43E-43	0.51039584	1.16E-13	-0.7639579	1.17E-36	-0.343213	1.73E-06
7.85E-16	0.5730632	1.54E-17	-0.694665	5.55E-28	-0.326666	5.68E-06
2.30E-10	-0.3725129	1.77E-07	0.55704108	1.81E-16	0.3345879	3.24E-06
1.68E-22	0.34290465	1.77E-06	-0.7114866	7.49E-30	-0.7372573	5.43E-33
8.20E-38	0.46621169	2.26E-11	-0.9082003	3.88E-71	-0.5870585	1.60E-18
3.50E-26	-0.4734249	1.01E-11	0.88763089	1.59E-63	0.67152528	1.30E-25
2.01E-44	0.28718282	7.39E-05	-0.6813622	1.36E-26	-0.4756067	7.85E-12
3.81E-34	-0.4903209	1.40E-12	0.67189023	1.20E-25	0.4268168	1.38E-09
3.05E-12	0.40978084	6.95E-09	-0.5883948	1.28E-18	-0.4796775	4.91E-12
1.06E-28	0.24727996	0.00069023	-0.651241	1.06E-23	-0.392793	3.20E-08
5.28E-29	-0.54058	1.98E-15	0.67911291	2.30E-26	0.2684247	0.00022053
5.62E-33	0.2737586	0.00016288	-0.7598907	4.54E-36	-0.4290739	1.10E-09
1.08E-27	0.25903991	0.00037027	-0.6261953	1.55E-21	-0.6679782	2.88E-25
2.46E-05	-0.3845723	6.50E-08	0.46020764	4.38E-11	0.78412317	9.11E-40
1.25E-10	-0.5473395	7.53E-16	0.75201553	5.83E-35	0.69494918	5.17E-28

9.16E-26	0.49191032	1.15E-12	-0.7726064	5.94E-38	-0.7001951	1.39E-28
1.66E-10	0.21107068	0.00392688	-0.544303	1.17E-15	-0.3982861	1.97E-08
2.50E-69	-0.5507518	4.58E-16	0.8848263	1.33E-62	0.591242	7.98E-19
7.45E-32	0.43837861	4.35E-10	-0.8415214	8.04E-51	-0.7700015	1.48E-37
1.92E-15	0.05023076	0.49713137	-0.4050566	1.07E-08	-0.3864607	5.53E-08
4.41E-61	0.68959187	1.92E-27	-0.7946706	1.58E-41	-0.5917691	7.31E-19
1.68E-19	-0.4413662	3.21E-10	0.80665387	1.16E-43	0.8044208	2.98E-43
1.78E-06	0.25057451	0.00058144	-0.4816684	3.89E-12	-0.4465262	1.88E-10
3.50E-11	-0.4918869	1.16E-12	0.71072993	9.15E-30	0.27242522	0.0001758
0.40464737	0.06503214	0.37914708	-0.2798414	0.0001144	-0.4066524	9.26E-09
4.17E-51	-0.5305086	8.05E-15	0.91495241	4.90E-74	0.51203617	9.37E-14
3.83E-43	-0.5371794	3.20E-15	0.8355437	1.78E-49	0.49348005	9.54E-13
3.79E-17	0.31708948	1.09E-05	-0.5815632	3.95E-18	-0.694713	5.48E-28
0.00617145	0.65021844	1.31E-23	-0.4174081	3.40E-09	-0.1810316	0.01366246
1.20E-45	-0.5466542	8.31E-16	0.86993222	4.45E-58	0.53099378	7.54E-15
0.00030108	0.08147708	0.27023092	-0.409532	7.11E-09	-0.4544894	8.12E-11
4.38E-28	-0.407187	8.82E-09	0.71381818	4.03E-30	0.31010719	1.74E-05
0.00011475	0.77118525	9.78E-38	-0.4848096	2.69E-12	-0.3098079	1.78E-05
1.86E-13	0.71333072	4.59E-30	-0.6147617	1.30E-20	-0.3168558	1.11E-05
1.47E-21	0.25211605	0.00053617	-0.5093628	1.32E-13	-0.5523589	3.62E-16
2.37E-26	0.38599335	5.75E-08	-0.747812	2.19E-34	-0.3654603	3.13E-07
0.00092452	-0.1422702	0.05338344	0.12669367	0.08571053	0.09129254	0.21650349
5.81E-15	0.33300978	3.63E-06	-0.7382661	4.03E-33	-0.363802	3.58E-07
6.68E-05	-0.5200795	3.28E-14	0.66794619	2.90E-25	0.54847391	6.39E-16
1.39E-11	0.29653838	4.16E-05	-0.6943734	5.96E-28	-0.4809319	4.24E-12
4.91E-33	0.69767035	2.63E-28	-0.7338036	1.50E-32	-0.4656522	2.41E-11
9.21E-16	-0.2137133	0.0034898	0.45067918	1.22E-10	0.4833764	3.19E-12
4.86E-22	-0.4800024	4.73E-12	0.87650556	5.30E-60	0.74284487	1.01E-33
3.02E-07	0.71661231	1.90E-30	-0.6737873	7.81E-26	-0.5182358	4.18E-14
0.08238801	-0.3857322	5.88E-08	0.55345444	3.08E-16	0.38554061	5.98E-08
9.64E-23	-0.3548141	7.22E-07	0.44345106	2.59E-10	-0.0259602	0.72576594
9.00E-07	-0.4990907	4.80E-13	0.61904424	5.91E-21	0.77958193	4.87E-39
7.97E-18	-0.4682699	1.80E-11	0.75605526	1.59E-35	0.31217604	1.52E-05
1.09E-05	-0.3200831	8.93E-06	0.58589746	1.94E-18	0.75662633	1.32E-35
0.05763651	-0.3625433	3.95E-07	0.23116412	0.00154629	0.24749822	0.00068248
2.92E-41	-0.5685403	3.13E-17	0.89701527	8.49E-67	0.50185741	3.41E-13
4.09E-23	0.38760482	5.01E-08	-0.8447034	1.47E-51	-0.3878493	4.91E-08
2.90E-31	0.30847836	1.94E-05	-0.6030519	1.05E-19	-0.2761845	0.00014161
2.22E-26	-0.4768323	6.82E-12	0.73230199	2.33E-32	0.29116594	5.80E-05
2.08E-05	0.39255453	3.27E-08	-0.4546223	8.00E-11	0.00122679	0.98677717
0.00472673	-0.3027782	2.80E-05	0.49842535	5.21E-13	0.68527379	5.41E-27
5.02E-31	0.36176294	4.20E-07	-0.74974	1.20E-34	-0.5084311	1.49E-13
1.70E-12	0.57324985	1.50E-17	-0.5548056	2.52E-16	-0.3545934	7.34E-07
1.65E-23	-0.5254014	1.61E-14	0.81864698	6.00E-46	0.54552041	9.79E-16
5.95E-14	0.36938554	2.29E-07	-0.7180891	1.27E-30	-0.4332702	7.27E-10
4.76E-65	-0.5289941	9.91E-15	0.95748355	9.82E-101	0.6758391	4.89E-26

0.00109675	0.26769153	0.0002298	-0.6036573	9.47E-20	-0.3508407	9.77E-07
1.79E-05	-0.3719503	1.86E-07	0.52485133	1.74E-14	0.39564772	2.49E-08
5.84E-41	0.63588574	2.37E-22	-0.9404087	1.17E-87	-0.7001758	1.40E-28
0.0243295	0.26232471	0.00030952	-0.3212303	8.26E-06	-0.2445547	0.00079409
4.32E-13	-0.5275727	1.20E-14	0.68696266	3.61E-27	0.42542494	1.58E-09
1.41E-25	-0.3141538	1.33E-05	0.64204596	6.95E-23	0.49660528	6.52E-13
1.56E-56	-0.4691557	1.63E-11	0.79636203	8.04E-42	0.36942311	2.28E-07
0.00926417	0.3572877	5.96E-07	-0.5851217	2.21E-18	-0.1715286	0.01956652
1.83E-14	-0.373273	1.67E-07	0.80107407	1.19E-42	0.58114317	4.23E-18
1.80E-18	0.76904312	2.06E-37	-0.6259022	1.63E-21	-0.4234281	1.91E-09
4.46E-08	-0.4391412	4.02E-10	0.68438707	6.67E-27	0.56328015	7.04E-17
2.86E-21	0.51474649	6.60E-14	-0.6855584	5.05E-27	-0.6777411	3.16E-26
2.32E-29	-0.3029058	2.78E-05	0.53195703	6.60E-15	0.04446169	0.54787813
1.07E-27	0.27193524	0.00018078	-0.5411722	1.82E-15	-0.1375029	0.06197979
0.05402257	-0.2287752	0.00173481	0.36984994	2.20E-07	0.65638738	3.58E-24
3.07E-24	-0.5140276	7.24E-14	0.81019075	2.56E-44	0.80958464	3.33E-44
2.28E-24	-0.4522521	1.03E-10	0.81975151	3.62E-46	0.28067786	0.00010891
2.44E-07	0.29509582	4.55E-05	-0.6991279	1.82E-28	-0.4366486	5.18E-10
1.68E-10	-0.4450448	2.19E-10	0.79354155	2.46E-41	0.43692497	5.04E-10
1.59E-46	-0.5740744	1.31E-17	0.88634716	4.24E-63	0.76803406	2.92E-37
4.70E-39	0.26760093	0.00023097	-0.6702247	1.75E-25	-0.4656831	2.40E-11
5.85E-14	-0.4506434	1.22E-10	0.62611829	1.57E-21	0.38344785	7.15E-08
1.84E-27	-0.4765781	7.02E-12	0.75867313	6.78E-36	0.40370886	1.21E-08
3.85E-21	-0.3855954	5.95E-08	0.81748016	1.02E-45	0.68957952	1.92E-27
9.53E-20	-0.4448583	2.24E-10	0.73382314	1.49E-32	0.19224324	0.00875265
1.53E-25	0.45251633	1.00E-10	-0.6057864	6.51E-20	-0.0803653	0.27684353
2.55E-17	-0.3864508	5.53E-08	0.62276644	2.95E-21	-0.0472626	0.52292632
6.84E-25	-0.2364592	0.00119332	0.49343611	9.59E-13	0.02909836	0.69418559
3.95E-07	0.37276771	1.74E-07	-0.5799196	5.16E-18	-0.3332425	3.57E-06
7.93E-17	0.21981034	0.00264417	-0.6148901	1.27E-20	-0.7972463	5.64E-42
8.01E-21	-0.1264762	0.086254	0.37496806	1.45E-07	0.01695238	0.81884488
0.0112398	0.42587831	1.51E-09	-0.4719162	1.19E-11	-0.6784381	2.69E-26
1.57E-18	-0.3678123	2.60E-07	0.49397512	8.99E-13	-0.0686971	0.35281325
3.89E-54	-0.4405608	3.48E-10	0.73145813	2.97E-32	0.4362436	5.40E-10
3.16E-43	0.4678945	1.88E-11	-0.7595263	5.12E-36	-0.5019716	3.36E-13
5.76E-14	-0.4459529	2.00E-10	0.68747159	3.20E-27	0.07365969	0.31903456
7.67E-15	-0.1058319	0.15164562	0.47708228	6.62E-12	0.68646842	4.07E-27
4.46E-39	-0.3370096	2.72E-06	0.69533188	4.70E-28	0.5115427	9.99E-14
4.16E-27	0.25955886	0.00035999	-0.6883627	2.58E-27	-0.4210043	2.41E-09
8.50E-24	0.3141147	1.34E-05	-0.6399983	1.05E-22	-0.4445915	2.30E-10
4.15E-37	0.59993064	1.81E-19	-0.7854525	5.53E-40	-0.4836121	3.10E-12
9.74E-34	0.11674144	0.11353346	-0.5960212	3.55E-19	-0.2623785	0.00030861
3.94E-14	-0.3844299	6.57E-08	0.73356756	1.61E-32	0.62221903	3.27E-21
2.78E-23	-0.4424483	2.87E-10	0.65574343	4.10E-24	0.0579638	0.43321114
1.97E-30	-0.3403048	2.14E-06	0.62802323	1.09E-21	0.3065989	2.19E-05
2.21E-55	-0.5784753	6.51E-18	0.92530811	5.49E-79	0.50434174	2.50E-13

2.30E-29	-0.46904	1.65E-11	0.69248897	9.47E-28	0.14771278	0.04480143
1.65E-44	-0.5413467	1.78E-15	0.86877986	9.44E-58	0.7690212	2.07E-37
2.14E-44	-0.5543739	2.69E-16	0.91809839	1.80E-75	0.81965096	3.79E-46
0.15625549	0.16594512	0.02397843	-0.3277778	5.25E-06	-0.3784581	1.09E-07
1.35E-51	-0.3175479	1.06E-05	0.88694233	2.69E-63	0.59679826	3.11E-19
8.34E-24	-0.4489287	1.46E-10	0.89440455	7.42E-66	0.77126073	9.52E-38
4.18E-13	-0.4985736	5.12E-13	0.55147263	4.12E-16	0.26461672	0.00027276
7.69E-30	-0.5148406	6.52E-14	0.89896999	1.61E-67	0.71703572	1.69E-30
7.05E-34	-0.4789258	5.35E-12	0.81361488	5.73E-45	0.32746757	5.37E-06
2.31E-13	0.13569	0.06553353	0.51399298	7.28E-14	0.25270255	0.00051983
2.77E-06	0.12328807	0.09454052	-0.3804158	9.23E-08	-0.5731185	1.53E-17
7.25E-27	0.51635418	5.35E-14	-0.7470634	2.77E-34	-0.2092767	0.00425107
2.69E-06	-0.441088	3.30E-10	0.70441962	4.74E-29	0.57737192	7.77E-18
2.85E-13	-0.4164778	3.71E-09	0.79213586	4.27E-41	0.60525363	7.15E-20
4.91E-24	-0.4753574	8.07E-12	0.89433198	7.87E-66	0.61255491	1.94E-20
2.77E-33	-0.5070517	1.77E-13	0.72726987	9.87E-32	0.68365513	7.93E-27
0.02755874	0.04070061	0.58227551	-0.4091405	7.37E-09	-0.4912493	1.25E-12
9.01E-28	0.37473145	1.48E-07	-0.6114473	2.37E-20	-0.3926258	3.24E-08
5.71E-09	-0.2702801	0.00019861	0.46196417	3.62E-11	0.69319173	7.97E-28
8.85E-55	-0.547547	7.31E-16	0.89960921	9.30E-68	0.50642321	1.92E-13
1.55E-15	0.33255055	3.75E-06	-0.6325236	4.58E-22	-0.4108491	6.29E-09
8.68E-15	0.40544493	1.03E-08	-0.6577508	2.68E-24	-0.625069	1.91E-21
3.81E-06	0.30078165	3.18E-05	-0.6196162	5.32E-21	-0.5071512	1.75E-13
3.45E-06	0.30102134	3.13E-05	-0.6010129	1.50E-19	-0.0972261	0.18798066
3.15E-09	-0.4029992	1.29E-08	0.73220354	2.40E-32	0.40589863	9.92E-09
0.06607523	-0.3460012	1.41E-06	0.44403947	2.44E-10	0.12302584	0.09524938
9.16E-18	0.34064531	2.09E-06	-0.5702861	2.38E-17	0.03106185	0.67468668
0.06704226	0.20782862	0.00453009	-0.4281013	1.21E-09	-0.5771339	8.07E-18
7.82E-25	0.49019432	1.42E-12	-0.8259175	2.03E-47	-0.7319353	2.59E-32
4.37E-09	0.35407575	7.64E-07	-0.5792039	5.79E-18	-0.4895078	1.54E-12
0.00026267	0.30259347	2.83E-05	-0.3920663	3.41E-08	-0.5278846	1.15E-14
1.73E-31	0.41881962	2.97E-09	-0.8039385	3.65E-43	-0.5817694	3.82E-18
0.02985113	0.31802801	1.03E-05	-0.4239248	1.82E-09	-0.5040871	2.58E-13
0.01560391	-0.1630319	0.02660261	0.42187113	2.22E-09	0.63709812	1.87E-22
2.38E-17	0.45679453	6.34E-11	-0.4001041	1.67E-08	-0.2916365	5.63E-05
1.34E-29	0.4182764	3.13E-09	-0.8773107	3.02E-60	-0.6714373	1.33E-25
2.36E-28	-0.4987302	5.02E-13	0.85830145	6.45E-55	0.52080752	2.98E-14
2.19E-25	-0.4902559	1.41E-12	0.90448788	1.23E-69	0.60788116	4.49E-20
8.95E-10	-0.3722704	1.81E-07	0.57982141	5.24E-18	0.55473716	2.55E-16
1.39E-23	-0.5388384	2.53E-15	0.86985663	4.68E-58	0.73845481	3.80E-33
2.48E-50	-0.4044304	1.13E-08	0.94115066	3.85E-88	0.65305282	7.23E-24
1.15E-20	-0.435957	5.56E-10	0.85963943	2.89E-55	0.54314006	1.38E-15
3.09E-67	-0.4943798	8.55E-13	0.92482364	9.70E-79	0.64060286	9.29E-23
2.80E-13	-0.4111714	6.11E-09	0.77111008	1.00E-37	0.7808846	3.03E-39
1.55E-50	-0.482281	3.62E-12	0.94046499	1.08E-87	0.78494943	6.68E-40
1.18E-38	-0.5231524	2.18E-14	0.86407987	1.89E-56	0.35195569	8.98E-07

1.62E-34	-0.4213258	2.34E-09	0.7907188	7.40E-41	0.29057643	6.01E-05
1.37E-35	-0.5507364	4.59E-16	0.94091577	5.48E-88	0.62975192	7.83E-22
6.18E-11	-0.4224635	2.10E-09	0.76742749	3.59E-37	0.40219065	1.39E-08
2.57E-34	0.66393781	7.02E-25	-0.9317546	1.92E-82	-0.5599929	1.16E-16
1.35E-31	0.40150721	1.48E-08	-0.6674192	3.26E-25	-0.5111416	1.05E-13
5.09E-29	-0.4418806	3.04E-10	0.85550742	3.37E-54	0.39105423	3.72E-08
3.47E-05	0.3497729	1.06E-06	-0.5949441	4.27E-19	-0.3925155	3.28E-08
0.00419561	0.39478875	2.68E-08	-0.5281587	1.11E-14	-0.5242732	1.88E-14
1.16E-31	-0.4642521	2.81E-11	0.86104797	1.23E-55	0.57804071	6.98E-18
2.04E-32	0.52554155	1.58E-14	-0.795125	1.32E-41	-0.564724	5.64E-17
0.71013198	0.314009	1.35E-05	-0.3540846	7.63E-07	-0.2716609	0.00018363
0.01803742	0.39540356	2.54E-08	-0.5399917	2.15E-15	-0.4977713	5.65E-13
6.56E-13	0.48143254	4.00E-12	-0.7645727	9.49E-37	-0.5069128	1.80E-13
1.64E-27	0.52713766	1.27E-14	-0.6979394	2.46E-28	-0.7139154	3.92E-30
4.35E-33	0.45362394	8.91E-11	-0.7866579	3.51E-40	-0.5823232	3.49E-18
0.00248835	-0.2762546	0.00014104	0.4978594	5.59E-13	0.69592501	4.06E-28
0.00988487	0.14320972	0.05181154	0.10943704	0.13810806	0.13917471	0.05884418
0.00143442	-0.3416491	1.94E-06	0.59130324	7.90E-19	0.3297068	4.59E-06
1.45E-21	-0.2990637	3.55E-05	0.68810193	2.75E-27	0.12220317	0.09750054
0.00016567	-0.3731881	1.68E-07	0.61760885	7.71E-21	0.52183685	2.60E-14
0.00014298	0.41482242	4.34E-09	-0.4957161	7.27E-13	-0.3173976	1.07E-05
6.51E-33	0.33585833	2.96E-06	-0.7188923	1.02E-30	-0.5748672	1.16E-17
3.12E-30	0.52172168	2.64E-14	-0.6058327	6.46E-20	-0.3409786	2.04E-06
2.62E-27	0.37712668	1.21E-07	-0.698059	2.38E-28	-0.6516475	9.71E-24
1.85E-56	0.43031608	9.75E-10	-0.8133039	6.57E-45	-0.5757922	1.00E-17
4.60E-05	0.38073416	8.98E-08	-0.6458376	3.22E-23	-0.2639366	0.00028322
0.00163258	0.36252419	3.96E-07	-0.6147891	1.29E-20	-0.4402974	3.58E-10
3.22E-30	0.31760531	1.06E-05	-0.6560608	3.83E-24	-0.4549853	7.70E-11
2.13E-07	0.47650748	7.08E-12	-0.6492974	1.58E-23	-0.6032965	1.01E-19
1.70E-11	-0.4141204	4.64E-09	0.65495774	4.84E-24	0.05005184	0.49866701
1.21E-36	-0.4304108	9.66E-10	0.79289915	3.17E-41	0.34905276	1.12E-06
1.46E-42	-0.4722352	1.15E-11	0.81812966	7.59E-46	0.36663396	2.85E-07
1.07E-39	-0.4559581	6.94E-11	0.84376879	2.43E-51	0.45679511	6.34E-11
8.71E-31	0.48716371	2.04E-12	-0.9222281	1.92E-77	-0.5694079	2.73E-17
7.56E-13	0.54794949	6.89E-16	-0.786506	3.72E-40	-0.483753	3.05E-12
3.60E-25	-0.507092	1.76E-13	0.86163448	8.58E-56	0.72786389	8.34E-32
0.07217007	-0.0961503	0.19293672	0.36727133	2.71E-07	-0.0477688	0.51847912
1.89E-45	0.51453288	6.78E-14	-0.8410184	1.05E-50	-0.5616859	8.97E-17
1.38E-10	0.31857441	9.90E-06	-0.3832626	7.26E-08	-0.3405835	2.10E-06
4.50E-46	0.52777713	1.17E-14	-0.789941	1.00E-40	-0.516394	5.32E-14
2.55E-21	0.20891256	0.00431974	-0.5163403	5.36E-14	-0.5456633	9.59E-16
6.21E-33	0.457521	5.86E-11	-0.777601	1.00E-38	-0.3896094	4.22E-08
3.28E-07	-0.4564027	6.61E-11	0.62803606	1.09E-21	0.73937042	2.89E-33
5.08E-28	-0.487826	1.88E-12	0.78796108	2.14E-40	0.89826102	2.96E-67
1.61E-17	-0.4277509	1.26E-09	0.67425146	7.03E-26	0.16396438	0.02573708
9.33E-19	-0.4894747	1.55E-12	0.80534312	2.02E-43	0.29718973	3.99E-05

1.47E-06	-0.332897	3.66E-06	0.67939877	2.15E-26	0.35942355	5.05E-07
1.28E-09	-0.4523801	1.02E-10	0.74084024	1.86E-33	0.52890451	1.00E-14
7.26E-17	-0.5440693	1.21E-15	0.86554657	7.50E-57	0.73456381	1.20E-32
1.72E-12	-0.3406777	2.08E-06	0.62958735	8.08E-22	0.2202184	0.00259483
2.99E-12	0.58090237	4.40E-18	-0.4530901	9.43E-11	-0.3758223	1.35E-07
7.64E-22	-0.5191667	3.70E-14	0.79851942	3.38E-42	0.38222506	7.92E-08
5.57E-24	0.43559613	5.76E-10	-0.7894287	1.22E-40	-0.8242244	4.52E-47
1.46E-23	0.617731	7.54E-21	-0.7866385	3.54E-40	-0.8122726	1.03E-44
2.89E-26	-0.2950539	4.56E-05	0.63968562	1.12E-22	0.10097449	0.17144021
4.70E-21	0.32417862	6.74E-06	-0.8241728	4.63E-47	-0.3750937	1.44E-07
4.59E-20	0.3453433	1.48E-06	-0.6727915	9.79E-26	-0.6046624	7.94E-20
1.67E-06	0.41853588	3.06E-09	-0.5086941	1.44E-13	-0.0499565	0.49948649
0.66408924	-0.3066693	2.18E-05	0.38120066	8.64E-08	0.19893944	0.00663322
9.07E-17	-0.4627275	3.33E-11	0.67751155	3.33E-26	0.6292465	8.63E-22
2.22E-10	-0.4573053	6.00E-11	0.74487846	5.43E-34	0.64523188	3.65E-23
1.23E-23	-0.4683586	1.78E-11	0.89310867	2.13E-65	0.6536728	6.35E-24
2.33E-30	0.47311261	1.04E-11	-0.7888425	1.53E-40	-0.6067453	5.50E-20
5.57E-07	0.03280034	0.65760057	-0.5077536	1.62E-13	0.16726683	0.02286328
9.06E-07	-0.4535744	8.95E-11	0.64345957	5.23E-23	0.65143254	1.02E-23
2.06E-25	0.21776723	0.00290421	-0.5337302	5.17E-15	-0.5505126	4.75E-16
1.97E-09	-0.4777642	6.12E-12	0.76087495	3.28E-36	0.62699207	1.33E-21
4.67E-30	-0.5979093	2.57E-19	0.92267035	1.16E-77	0.76093145	3.22E-36
9.04E-33	-0.5019334	3.37E-13	0.88364464	3.20E-62	0.49325935	9.80E-13
3.41E-14	0.21054314	0.00401982	-0.5632587	7.06E-17	-0.6971892	2.96E-28
1.95E-09	0.14123756	0.05515644	-0.4561873	6.77E-11	0.19137314	0.00906807
1.63E-13	0.32678789	5.63E-06	-0.6629531	8.71E-25	-0.5812778	4.14E-18
1.66E-26	0.33667207	2.79E-06	-0.8124282	9.66E-45	-0.7378358	4.58E-33
0.0512535	0.55425723	2.73E-16	-0.6165198	9.42E-21	-0.1334568	0.07013841
4.75E-15	0.45277929	9.74E-11	-0.834568	2.92E-49	-0.6115159	2.34E-20
6.54E-09	-0.2634336	0.0002912	0.51374071	7.52E-14	0.23220867	0.0014699
8.03E-17	0.38826086	4.74E-08	-0.765825	6.21E-37	-0.6995429	1.64E-28
4.77E-08	0.5521351	3.74E-16	-0.6409142	8.73E-23	-0.6343436	3.21E-22
1.45E-32	-0.5528676	3.36E-16	0.88112781	2.01E-61	0.67894558	2.39E-26
4.39E-50	-0.6024769	1.16E-19	0.97122735	5.59E-116	0.76054379	3.66E-36
2.15E-43	-0.5576099	1.66E-16	0.9661924	1.14E-109	0.74383741	7.48E-34
0.013115	-0.3281025	5.13E-06	0.4262808	1.45E-09	0.28787146	7.09E-05
4.00E-35	0.48068671	4.36E-12	-0.6511806	1.07E-23	-0.257964	0.00039246
0.00018341	0.08012156	0.27830778	-0.3827307	7.59E-08	-0.3950445	2.62E-08
1.39E-11	0.35025143	1.02E-06	-0.7242165	2.34E-31	-0.3581116	5.59E-07
4.51E-36	0.43109649	9.03E-10	-0.7924916	3.71E-41	-0.5356077	3.98E-15
6.28E-10	0.46086949	4.08E-11	-0.7007513	1.21E-28	-0.5830943	3.08E-18
1.99E-14	0.52798927	1.14E-14	-0.7589467	6.20E-36	-0.3972474	2.16E-08
1.19E-23	-0.5205199	3.09E-14	0.74350096	8.29E-34	0.26629103	0.00024852
2.89E-14	0.38786244	4.90E-08	-0.6859338	4.62E-27	-0.5581473	1.53E-16
2.88E-32	-0.4590176	4.99E-11	0.78332733	1.23E-39	0.35524024	6.98E-07
0.00062931	-0.4532191	9.30E-11	0.5661712	4.52E-17	0.51034968	1.16E-13

5.16E-06	-0.3222092	7.72E-06	0.60528129	7.12E-20	0.38320125	7.30E-08
3.24E-41	-0.5073247	1.71E-13	0.96317717	2.46E-106	0.69987475	1.51E-28
3.06E-19	-0.4601912	4.39E-11	0.73867534	3.56E-33	0.30127069	3.08E-05
2.14E-22	0.38828599	4.73E-08	-0.6170099	8.61E-21	-0.6880226	2.80E-27
0.1224371	0.36864997	2.43E-07	-0.4404355	3.53E-10	0.09466189	0.19994992
6.99E-21	-0.456486	6.55E-11	0.89888104	1.74E-67	0.65927492	1.93E-24
7.82E-17	-0.490114	1.43E-12	0.82240042	1.06E-46	0.8146825	3.57E-45
3.90E-10	0.3347337	3.21E-06	-0.407917	8.25E-09	0.1022843	0.16592293
0.1010723	0.54200068	1.62E-15	-0.4133414	4.99E-09	-0.6047155	7.87E-20
1.39E-34	0.25222915	0.00053298	-0.634139	3.34E-22	-0.1873015	0.01068168
7.70E-14	-0.1860082	0.01124473	0.68966584	1.88E-27	0.35015239	1.03E-06
1.33E-44	-0.4775527	6.27E-12	0.77664697	1.41E-38	0.28355165	9.19E-05
8.47E-26	-0.4905937	1.35E-12	0.88853926	7.91E-64	0.80132211	1.08E-42
2.12E-09	-0.2858394	8.01E-05	0.64458436	4.16E-23	0.31127675	1.61E-05
6.40E-27	0.57601306	9.65E-18	-0.7207727	6.08E-31	-0.6596175	1.79E-24
7.69E-37	-0.6130337	1.78E-20	0.82103572	2.00E-46	0.73331641	1.73E-32
6.04E-19	0.25482374	0.00046447	-0.5504207	4.81E-16	-0.6849624	5.82E-27
3.20E-11	0.49820483	5.36E-13	-0.6788136	2.46E-26	-0.3811343	8.69E-08
3.83E-43	0.48264485	3.47E-12	-0.8274278	9.83E-48	-0.4961047	6.93E-13
1.60E-12	-0.3471537	1.29E-06	0.61606635	1.02E-20	0.28430566	8.78E-05
1.88E-31	-0.3185546	9.91E-06	0.63892652	1.30E-22	0.13642514	0.06407285
4.32E-34	-0.5415847	1.72E-15	0.92398488	2.58E-78	0.5708871	2.17E-17
4.89E-28	0.53396576	5.00E-15	-0.7512363	7.47E-35	-0.815556	2.42E-45
1.62E-34	-0.5462391	8.83E-16	0.93056609	8.81E-82	0.5797616	5.29E-18
5.71E-42	0.41990101	2.68E-09	-0.6309976	6.16E-22	-0.3170844	1.09E-05
1.19E-23	-0.5257399	1.54E-14	0.80523336	2.12E-43	0.87091791	2.33E-58
2.63E-36	-0.4618923	3.64E-11	0.7391846	3.06E-33	0.30685023	2.15E-05
3.64E-28	-0.4244172	1.74E-09	0.8166681	1.47E-45	0.43419088	6.63E-10
5.78E-35	0.23650083	0.00119086	-0.5545933	2.60E-16	-0.2331006	0.00140742
6.78E-39	0.48257956	3.50E-12	-0.7283117	7.34E-32	-0.5336451	5.23E-15
1.59E-09	0.74262547	1.08E-33	-0.759405	5.33E-36	-0.4413069	3.23E-10
6.56E-21	0.5194472	3.56E-14	-0.7741333	3.46E-38	-0.5200918	3.27E-14
5.52E-26	0.58117053	4.21E-18	-0.7592594	5.59E-36	-0.7349823	1.06E-32
2.33E-22	-0.4757122	7.75E-12	0.87760502	2.46E-60	0.73247944	2.21E-32
3.33E-11	-0.3370596	2.71E-06	0.77347001	4.38E-38	0.5218974	2.58E-14
1.38E-19	-0.3680282	2.55E-07	0.5662667	4.45E-17	0.45243818	1.01E-10
2.73E-34	-0.5096636	1.27E-13	0.93007299	1.65E-81	0.53147045	7.06E-15
4.53E-09	0.52443875	1.83E-14	-0.6987139	2.02E-28	-0.4546931	7.94E-11
8.01E-17	0.4386498	4.23E-10	-0.8067211	1.13E-43	-0.471039	1.32E-11
6.17E-21	-0.5232634	2.15E-14	0.71829446	1.20E-30	0.3685099	2.46E-07
1.07E-20	-0.0289598	0.6955697	0.71764257	1.43E-30	0.43256817	7.80E-10
1.57E-52	0.63623736	2.21E-22	-0.9176071	3.04E-75	-0.579404	5.60E-18
1.71E-33	-0.5673634	3.76E-17	0.89627653	1.58E-66	0.50552224	2.15E-13
4.29E-32	0.21985268	0.00263901	-0.7597004	4.83E-36	-0.5676831	3.57E-17
1.53E-32	-0.534257	4.81E-15	0.83632626	1.19E-49	0.40835517	7.92E-09
1.18E-22	-0.464404	2.76E-11	0.72029141	6.94E-31	0.78019534	3.90E-39

1.49E-08	-0.4811835	4.12E-12	0.69360158	7.21E-28	0.2400132	0.0009996
2.90E-33	-0.4980277	5.47E-13	0.82264006	9.51E-47	0.31567255	1.20E-05
1.74E-22	-0.4465032	1.89E-10	0.77707475	1.21E-38	0.34606953	1.40E-06
4.04E-12	-0.3241092	6.78E-06	0.69217557	1.02E-27	0.36521051	3.20E-07
1.32E-27	0.37487753	1.46E-07	-0.6630614	8.50E-25	-0.3720929	1.84E-07
9.75E-16	-0.4418174	3.06E-10	0.78683008	3.29E-40	0.77079899	1.12E-37
6.42E-09	0.56367617	6.62E-17	-0.7079942	1.88E-29	-0.116175	0.11530787
7.58E-22	0.57704516	8.18E-18	-0.714366	3.48E-30	-0.3996861	1.74E-08
1.72E-16	-0.3104651	1.70E-05	0.73691745	6.01E-33	0.20718763	0.00465877
2.70E-38	-0.5014425	3.59E-13	0.69855427	2.11E-28	0.26533869	0.00026205
7.20E-22	0.48273293	3.44E-12	-0.5796121	5.42E-18	-0.6301042	7.31E-22
3.28E-21	0.53341681	5.40E-15	-0.8313374	1.46E-48	-0.6831939	8.85E-27
6.42E-25	0.52769255	1.18E-14	-0.6053349	7.05E-20	-0.6380958	1.53E-22
9.65E-52	0.46938396	1.59E-11	-0.7286264	6.71E-32	-0.4687641	1.70E-11
1.03E-34	-0.5400013	2.15E-15	0.85650906	1.87E-54	0.4728176	1.08E-11
4.16E-31	-0.5455649	9.73E-16	0.81754973	9.87E-46	0.51080609	1.10E-13
3.83E-06	-0.2295471	0.00167172	0.48480656	2.69E-12	-0.0716792	0.33225457
0.3799683	-0.2373334	0.00114272	0.44356409	2.56E-10	0.13155986	0.07425385
4.92E-22	0.38594585	5.78E-08	-0.6006757	1.59E-19	-0.19595	0.00751519
0.53038854	-0.1266943	0.08570896	0.36633282	2.92E-07	0.14446665	0.04976888
3.10E-18	0.47504315	8.37E-12	-0.7729172	5.32E-38	-0.6302977	7.05E-22
5.53E-07	-0.4503848	1.26E-10	0.65352128	6.56E-24	0.72060697	6.37E-31
2.38E-68	-0.5522383	3.68E-16	0.8978276	4.27E-67	0.59375583	5.22E-19
2.60E-38	-0.4059047	9.92E-09	0.63753432	1.71E-22	0.14937243	0.04242636
0.365049	-0.2769771	0.00013525	0.45972858	4.61E-11	0.50466892	2.39E-13
9.31E-10	0.32054053	8.66E-06	-0.4762608	7.28E-12	-0.3709937	2.01E-07
1.52E-45	-0.5150083	6.38E-14	0.84896772	1.41E-52	0.59172612	7.36E-19
6.55E-17	-0.4300452	1.00E-09	0.79955505	2.22E-42	0.34181247	1.92E-06
2.74E-16	0.34548431	1.46E-06	-0.7141626	3.67E-30	-0.5342344	4.82E-15
2.59E-26	0.34287054	1.77E-06	-0.791595	5.27E-41	-0.7815209	2.39E-39
4.94E-12	0.21250253	0.0036843	-0.3634144	3.69E-07	0.15818982	0.03150852
5.60E-25	-0.5671356	3.89E-17	0.90918756	1.51E-71	0.51607042	5.55E-14
3.94E-24	-0.5209084	2.94E-14	0.73190726	2.61E-32	0.18230602	0.01300352
5.11E-16	-0.5014037	3.60E-13	0.74049425	2.06E-33	0.80937177	3.64E-44
7.50E-51	0.42073361	2.48E-09	-0.8895574	3.58E-64	-0.6715654	1.29E-25
2.91E-26	0.40561621	1.02E-08	-0.6542006	5.68E-24	-0.1465455	0.04653765
1.68E-47	-0.5517206	3.98E-16	0.83124724	1.53E-48	0.40469361	1.11E-08
1.37E-15	-0.4855116	2.48E-12	0.8297051	3.26E-48	0.4911326	1.27E-12
2.96E-33	-0.5786889	6.29E-18	0.93790619	4.49E-86	0.69668243	3.36E-28
9.84E-25	-0.3702151	2.14E-07	0.79520493	1.27E-41	0.28736635	7.31E-05
2.66E-32	0.3025501	2.84E-05	-0.6557548	4.09E-24	-0.5153157	6.13E-14
6.11E-56	-0.5915906	7.53E-19	0.87115688	1.99E-58	0.58020153	4.93E-18
2.20E-37	0.41250766	5.39E-09	-0.6592757	1.93E-24	-0.3707761	2.04E-07
4.84E-34	0.60883524	3.79E-20	-0.8275837	9.12E-48	-0.555533	2.26E-16
2.19E-07	0.27466612	0.00015459	-0.65332	6.84E-24	-0.2547444	0.00046644
0.00035584	0.15635902	0.03355423	-0.5404517	2.02E-15	-0.545965	9.18E-16

3.80E-47	-0.556966	1.83E-16	0.85652133	1.86E-54		0.51336043	7.90E-14
6.61E-27	-0.380386	9.25E-08	0.76814116	2.81E-37		0.41097158	6.22E-09
1.97E-33	-0.4945744	8.35E-13	0.82581576	2.13E-47		0.51737405	4.68E-14
3.31E-19	0.55910195	1.33E-16	-0.7468559	2.95E-34		-0.7642674	1.05E-36
0.02250029	-0.4156206	4.03E-09	0.56194484	8.63E-17		0.42723759	1.32E-09
4.81E-53	-0.5424254	1.52E-15	0.85177812	2.90E-53		0.4555537	7.24E-11
4.61E-23	-0.3979065	2.04E-08	0.80610683	1.47E-43		0.500472	4.05E-13
9.37E-43	0.66122425	1.27E-24	-0.8748641	1.64E-59		-0.7233339	2.99E-31
3.89E-31	-0.4861556	2.30E-12	0.86569182	6.84E-57		0.74658877	3.20E-34
8.78E-05	-0.5648236	5.56E-17	0.6562662	3.67E-24		0.55290029	3.34E-16
8.11E-51	-0.5460466	9.08E-16	0.65425451	5.62E-24		0.6128687	1.83E-20
1.48E-34	-0.2208598	0.00251897	0.75562613	1.83E-35		0.41789075	3.25E-09
5.69E-27	-0.4217663	2.24E-09	0.62809778	1.08E-21		0.48356676	3.12E-12
1.07E-31	0.34859028	1.16E-06	-0.6154123	1.15E-20		-0.242501	0.00088166
1.30E-07	-0.5308457	7.69E-15	0.70900623	1.44E-29		0.57365372	1.40E-17
5.68E-18	-0.5700183	2.49E-17	0.83543482	1.88E-49		0.66779651	3.00E-25
0.00042353	0.3348584	3.18E-06	-0.5059322	2.04E-13		0.18030083	0.01405339
4.65E-14	-0.5086315	1.45E-13	0.80161526	9.55E-43		0.69645642	3.56E-28
3.73E-55	0.45628006	6.70E-11	-0.8911489	1.02E-64		-0.7519225	6.01E-35
3.63E-32	-0.5104383	1.15E-13	0.69738373	2.82E-28		0.26426053	0.0002782
1.63E-12	-0.5910532	8.24E-19	0.66935426	2.12E-25		0.5964197	3.32E-19
7.54E-05	-0.195929	0.00752174	0.3145634	1.30E-05		-0.1375406	0.06190749
8.05E-27	0.53442079	4.70E-15	-0.8261472	1.82E-47		-0.235453	0.0012541
1.04E-52	0.55341261	3.10E-16	-0.8931479	2.06E-65		-0.7512184	7.51E-35
1.44E-28	0.36485631	3.29E-07	-0.750863	8.40E-35		-0.7959323	9.54E-42
2.75E-26	-0.3928386	3.19E-08	0.73414854	1.36E-32		0.71039176	1.00E-29
1.57E-38	-0.5714009	2.00E-17	0.90356688	2.82E-69		0.55347218	3.07E-16
1.85E-27	-0.4019893	1.41E-08	0.67164464	1.27E-25		0.21121921	0.00390106
3.55E-47	-0.4362433	5.40E-10	0.76520084	7.68E-37		0.3156155	1.21E-05
1.42E-61	-0.5548592	2.50E-16	0.83386801	4.15E-49		0.44046607	3.52E-10
1.24E-34	-0.5769073	8.37E-18	0.76378707	1.24E-36		0.29992378	3.36E-05
6.15E-53	-0.4110797	6.16E-09	0.75853443	7.10E-36		0.31915909	9.51E-06
4.37E-32	-0.475235	8.19E-12	0.77718492	1.16E-38		0.59244331	6.52E-19
6.98E-39	-0.4118229	5.75E-09	0.78112845	2.77E-39		0.28851825	6.81E-05
6.92E-51	-0.385743	5.88E-08	0.79900228	2.78E-42		0.33846725	2.45E-06
0.05688798	-0.2618025	0.00031852	0.47443963	8.96E-12		0.45891203	5.04E-11
2.53E-21	0.43250267	7.85E-10	-0.7341185	1.37E-32		-0.6752965	5.54E-26
3.16E-31	-0.243233	0.00084948	0.6475201	2.28E-23		0.42682814	1.37E-09
1.59E-06	-0.1713322	0.0197089	0.47022366	1.45E-11		0.39436853	2.79E-08
6.12E-31	-0.2060334	0.00489878	0.61546201	1.14E-20		0.70589525	3.24E-29
2.44E-44	0.36972859	2.22E-07	-0.7371621	5.59E-33		-0.4889598	1.64E-12
1.76E-20	-0.4474181	1.71E-10	0.78563106	5.17E-40		0.2710759	0.00018984
1.38E-43	-0.4362194	5.41E-10	0.83184893	1.13E-48		0.49175534	1.18E-12
4.10E-22	-0.3979687	2.03E-08	0.84048727	1.39E-50		0.5256022	1.57E-14
1.03E-48	-0.4233249	1.93E-09	0.7374392	5.15E-33		0.29241975	5.37E-05
1.42E-51	-0.5140546	7.22E-14	0.88302593	5.05E-62		0.41930943	2.84E-09

1.74E-15	0.30105001	3.13E-05	-0.6467033	2.70E-23	-0.5698867	2.54E-17
8.90E-50	-0.178431	0.0150985	0.81439272	4.06E-45	0.59457498	4.55E-19
7.41E-27	0.36392637	3.54E-07	-0.4839297	2.99E-12	-0.1375607	0.06186911
2.39E-34	0.57723911	7.93E-18	-0.632684	4.44E-22	-0.5031515	2.90E-13
1.72E-14	-0.4489074	1.47E-10	0.62734154	1.24E-21	0.80161929	9.53E-43
2.79E-34	0.28786394	7.09E-05	-0.6202184	4.76E-21	-0.3174595	1.07E-05
0.01934994	-0.3289417	4.84E-06	0.58003355	5.06E-18	0.50061868	3.97E-13
2.57E-50	0.35039397	1.01E-06	-0.6908375	1.42E-27	-0.5890735	1.15E-18
6.64E-24	-0.279736	0.00011511	0.48344586	3.16E-12	0.2918645	5.55E-05
1.12E-14	-0.4055807	1.02E-08	0.83674798	9.63E-50	0.50307901	2.92E-13
5.98E-54	-0.5278345	1.16E-14	0.89735554	6.37E-67	0.66462668	6.04E-25
6.46E-13	-0.3816372	8.33E-08	0.69642949	3.58E-28	0.60918279	3.56E-20
2.38E-07	-0.3056804	2.32E-05	0.60069291	1.59E-19	0.37165211	1.90E-07
1.01E-30	0.3844239	6.58E-08	-0.6568538	3.24E-24	-0.6893446	2.04E-27
7.97E-11	0.51692002	4.97E-14	-0.5463211	8.72E-16	-0.0721343	0.32918598
5.22E-10	-0.4471239	1.77E-10	0.67518771	5.68E-26	0.05550822	0.45298071
4.88E-40	-0.2377615	0.00111866	0.6314135	5.68E-22	0.2447587	0.00078584
1.02E-27	-0.454938	7.74E-11	0.70109975	1.11E-28	0.251968	0.00054037
4.19E-30	-0.494663	8.26E-13	0.85824156	6.69E-55	0.6418804	7.19E-23
0.00840543	-0.3793788	1.01E-07	0.57380157	1.37E-17	0.52006369	3.29E-14
3.33E-30	-0.4624345	3.43E-11	0.82172534	1.46E-46	0.42298442	2.00E-09
4.77E-54	0.6068123	5.43E-20	-0.8802096	3.89E-61	-0.5877679	1.43E-18
2.25E-39	0.49821603	5.35E-13	-0.7997697	2.03E-42	-0.6816949	1.26E-26
2.48E-09	-0.3486975	1.15E-06	0.68268395	9.98E-27	0.22020437	0.00259651
5.08E-24	-0.4324373	7.90E-10	0.74694539	2.87E-34	0.28770549	7.16E-05
0.00028332	-0.4467094	1.85E-10	0.64460911	4.14E-23	0.290608	6.00E-05
3.20E-09	0.33600068	2.93E-06	-0.6411119	8.39E-23	-0.7180805	1.27E-30
1.87E-21	-0.488911	1.65E-12	0.89037009	1.89E-64	0.66444961	6.28E-25
1.14E-40	-0.5540606	2.81E-16	0.94471208	1.50E-90	0.74957961	1.26E-34
5.09E-59	-0.4560083	6.90E-11	0.79691265	6.45E-42	0.35695005	6.12E-07
1.72E-37	-0.3859197	5.79E-08	0.83197208	1.07E-48	0.45775744	5.71E-11
3.69E-12	-0.4822211	3.65E-12	0.66289459	8.82E-25	0.56225619	8.23E-17
6.93E-10	0.66474535	5.88E-25	-0.7901667	9.17E-41	-0.3896642	4.20E-08
1.55E-24	-0.4745264	8.88E-12	0.89588244	2.19E-66	0.67919123	2.26E-26
1.35E-27	-0.5567662	1.88E-16	0.90007216	6.22E-68	0.66940035	2.10E-25
4.47E-14	0.34728752	1.28E-06	-0.6051412	7.30E-20	-0.7166592	1.87E-30
6.17E-38	-0.440007	3.69E-10	0.60785071	4.52E-20	0.16021093	0.02937435
6.17E-14	-0.4162858	3.78E-09	0.61314692	1.74E-20	0.67138789	1.34E-25
0.04561201	-0.2596763	0.0003577	0.52500708	1.70E-14	0.61828958	6.80E-21
9.77E-12	0.26111189	0.00033079	-0.59232	6.66E-19	-0.6334245	3.84E-22
2.43E-26	-0.4324102	7.92E-10	0.80925664	3.83E-44	0.33474189	3.21E-06
5.46E-19	-0.4484563	1.54E-10	0.82362997	5.98E-47	0.69294804	8.47E-28
2.07E-10	0.45099772	1.18E-10	-0.6321154	4.96E-22	-0.2371346	0.00115404
1.94E-21	-0.4283391	1.18E-09	0.67310585	9.12E-26	0.12986266	0.07809981
7.81E-42	0.5891776	1.13E-18	-0.620919	4.17E-21	-0.3759111	1.34E-07
3.93E-36	0.33089112	4.22E-06	-0.7450312	5.18E-34	-0.5137712	7.49E-14

1.50E-12	0.2068444	0.00472902	-0.6503406	1.27E-23	0.00721857	0.92231424
1.52E-28	-0.654891	4.91E-24	0.87444884	2.18E-59	0.51672196	5.10E-14
1.11E-24	0.52402907	1.94E-14	-0.7151162	2.84E-30	-0.6972749	2.90E-28
1.87E-15	-0.4016869	1.45E-08	0.78345774	1.17E-39	0.67585713	4.87E-26
8.30E-28	-0.5451065	1.04E-15	0.91333715	2.54E-73	0.77053868	1.23E-37
5.29E-25	-0.5411333	1.83E-15	0.83978663	2.00E-50	0.55959	1.23E-16
1.89E-05	-0.4737098	9.74E-12	0.58140082	4.06E-18	0.50891068	1.40E-13
6.54E-33	-0.4741403	9.28E-12	0.91873484	9.09E-76	0.58157352	3.94E-18
2.67E-18	-0.4696537	1.54E-11	0.82913108	4.31E-48	0.61353058	1.63E-20
3.26E-08	-0.3628208	3.87E-07	0.52796814	1.14E-14	0.7400548	2.36E-33
3.86E-10	0.12452952	0.0912415	-0.5489538	5.96E-16	-0.5539547	2.86E-16
2.72E-36	0.201427	0.00597096	-0.5795722	5.45E-18	-0.386272	5.62E-08
5.58E-31	-0.5086532	1.45E-13	0.86327063	3.12E-56	0.53824127	2.76E-15
2.38E-25	0.24553398	0.00075522	-0.6841757	7.02E-27	-0.2858266	8.02E-05
6.99E-09	0.20457957	0.00521687	-0.4575019	5.87E-11	-0.2029066	0.00560568
1.84E-11	-0.4409681	3.34E-10	0.79766861	4.76E-42	0.60378518	9.26E-20
8.05E-22	0.39034679	3.96E-08	-0.894928	4.83E-66	-0.477202	6.53E-12
2.26E-22	-0.506286	1.95E-13	0.74732502	2.55E-34	0.20984584	0.0041457
1.15E-33	0.29943775	3.46E-05	-0.5895603	1.06E-18	-0.3274517	5.37E-06
6.49E-10	-0.5331343	5.61E-15	0.77605698	1.74E-38	0.56720366	3.85E-17
2.35E-11	-0.3205657	8.64E-06	0.78176578	2.19E-39	0.68293941	9.39E-27
3.38E-30	-0.4312225	8.91E-10	0.74134949	1.59E-33	0.35743374	5.89E-07
9.09E-27	-0.4842348	2.88E-12	0.86661269	3.81E-57	0.7259726	1.43E-31
6.50E-41	-0.4262091	1.46E-09	0.68000283	1.87E-26	0.32644703	5.76E-06
3.01E-24	-0.4932599	9.80E-13	0.7899186	1.01E-40	0.36144758	4.31E-07
2.64E-33	0.78618389	4.20E-40	-0.8196195	3.85E-46	-0.6768705	3.86E-26
2.62E-09	-0.3746391	1.49E-07	0.7226494	3.62E-31	0.57143369	1.99E-17
0.00023026	-0.3457672	1.43E-06	0.37839197	1.09E-07	0.0114158	0.87743291
3.58E-15	0.35783715	5.71E-07	-0.6388007	1.33E-22	-0.7671571	3.94E-37
6.87E-50	-0.4034154	1.24E-08	0.70419992	5.02E-29	0.3030268	2.76E-05
1.82E-14	0.34905735	1.12E-06	-0.5716153	1.94E-17	-0.6946944	5.51E-28
3.43E-67	0.52527005	1.64E-14	-0.8417974	6.94E-51	-0.4643391	2.78E-11
0.01309763	-0.2469326	0.00070273	0.58694213	1.63E-18	0.4636915	2.99E-11
4.21E-29	-0.4121308	5.58E-09	0.74468692	5.76E-34	0.78478365	7.11E-40
1.13E-17	0.70450995	4.63E-29	-0.8079241	6.78E-44	-0.3536214	7.91E-07
1.86E-08	-0.3122426	1.51E-05	0.39313164	3.10E-08	-0.1536085	0.03683873
1.22E-47	-0.4336357	7.01E-10	0.70562132	3.48E-29	0.47836194	5.71E-12
1.10E-40	0.51537031	6.08E-14	-0.8089762	4.32E-44	-0.713593	4.28E-30
0.0012082	-0.1600632	0.02952601	0.41990657	2.68E-09	0.39523401	2.58E-08
1.37E-06	-0.2874253	7.28E-05	0.6597204	1.75E-24	0.38199206	8.08E-08
2.48E-17	0.41067757	6.39E-09	-0.6547692	5.04E-24	-0.5075332	1.67E-13
7.90E-15	0.31911937	9.54E-06	-0.5269355	1.31E-14	-0.3403325	2.14E-06
9.78E-66	-0.405769	1.00E-08	0.79512059	1.32E-41	0.38587191	5.81E-08
9.01E-24	0.30590569	2.29E-05	-0.6407312	9.06E-23	-0.1744424	0.01755709
2.49E-09	0.45207545	1.05E-10	-0.7261747	1.35E-31	-0.5038743	2.65E-13
4.95E-19	0.4853877	2.51E-12	-0.72567	1.55E-31	-0.2152488	0.0032565

2.60E-28	0.50817537	1.54E-13	-0.6214402	3.79E-21	-0.7418757	1.36E-33
1.08E-11	0.55902069	1.34E-16	-0.6313092	5.80E-22	-0.5312203	7.31E-15
1.16E-36	0.51557181	5.93E-14	-0.8092179	3.89E-44	-0.6321686	4.91E-22
4.64E-07	-0.3214983	8.11E-06	0.29025073	6.13E-05	-0.2206533	0.00254317
2.95E-22	0.34677472	1.33E-06	-0.6086301	3.93E-20	-0.217449	0.00294674
9.89E-05	0.21460167	0.00335306	-0.415823	3.95E-09	-0.5673346	3.77E-17
0.00960836	0.47025523	1.44E-11	-0.4896327	1.52E-12	-0.4528376	9.68E-11
2.46E-15	-0.4721339	1.16E-11	0.86386308	2.16E-56	0.65675145	3.31E-24
0.08651885	-0.2635547	0.00028926	0.41868222	3.01E-09	0.58614298	1.87E-18
9.84E-39	-0.5415324	1.73E-15	0.9550095	1.55E-98	0.72479344	1.99E-31
3.37E-63	-0.5706116	2.27E-17	0.91481632	5.63E-74	0.52127314	2.80E-14
1.13E-16	0.26072338	0.00033788	-0.4617673	3.69E-11	0.08701491	0.2388986
5.13E-15	0.36530624	3.17E-07	-0.6897948	1.83E-27	-0.4705885	1.39E-11
1.14E-05	-0.255604	0.00044552	0.58762793	1.46E-18	0.10343458	0.16118783
1.37E-18	-0.4914772	1.22E-12	0.86074624	1.48E-55	0.37318729	1.68E-07
1.34E-44	0.39923164	1.81E-08	-0.7478033	2.20E-34	-0.2714246	0.00018611
1.25E-08	0.72868002	6.61E-32	-0.5288903	1.00E-14	-0.6007425	1.58E-19
0.00025535	0.18169974	0.01331345	-0.411373	5.99E-09	-0.3883149	4.72E-08
9.61E-42	0.37354879	1.63E-07	-0.6544478	5.39E-24	-0.3992563	1.81E-08
2.32E-20	0.39188812	3.46E-08	-0.5495079	5.50E-16	-0.2606179	0.00033983
1.69E-08	0.43274749	7.66E-10	-0.7772117	1.15E-38	-0.3120155	1.54E-05
3.32E-35	0.66490917	5.68E-25	-0.9412353	3.38E-88	-0.5875858	1.47E-18
9.99E-21	0.58542354	2.10E-18	-0.752869	4.44E-35	-0.2715516	0.00018477
3.06E-07	0.10596146	0.15114246	-0.2828418	9.58E-05	0.19514625	0.00776946
1.03E-26	-0.193478	0.00832172	0.56999405	2.50E-17	0.0307906	0.67736791
0.01849353	-0.391935	3.45E-08	0.5938774	5.12E-19	0.5140501	7.22E-14
1.95E-23	-0.5760586	9.58E-18	0.89783526	4.25E-67	0.68423622	6.92E-27
1.22E-22	-0.3799024	9.63E-08	0.78130862	2.59E-39	0.44915735	1.43E-10
4.28E-05	0.17190299	0.01929767	-0.4334325	7.16E-10	-0.6237925	2.44E-21
1.54E-15	-0.4631374	3.18E-11	0.79036812	8.48E-41	0.72432435	2.27E-31
5.25E-15	0.71267313	5.47E-30	-0.7209037	5.87E-31	-0.6805618	1.64E-26
0.01471305	0.238692	0.00106796	-0.4566382	6.45E-11	-0.5614141	9.35E-17
4.60E-30	0.41423252	4.59E-09	-0.885519	7.91E-63	-0.6696848	1.97E-25
3.54E-19	0.36729002	2.71E-07	-0.8125593	9.12E-45	-0.4533312	9.19E-11
0.00274422	0.50526852	2.22E-13	-0.6826243	1.01E-26	-0.180548	0.01392007
9.76E-06	-0.3934369	3.02E-08	0.65872763	2.17E-24	0.21836126	0.00282632
6.58E-26	0.44221486	2.94E-10	-0.7677011	3.27E-37	-0.5465193	8.48E-16
6.92E-14	-0.4192899	2.84E-09	0.79967151	2.12E-42	0.67412266	7.24E-26
4.14E-13	-0.4996937	4.46E-13	0.77121428	9.68E-38	0.75631745	1.46E-35
3.81E-09	-0.4374241	4.79E-10	0.73132339	3.09E-32	0.40678341	9.15E-09
0.00019572	0.26692983	0.00023981	-0.434335	6.54E-10	-0.6534594	6.64E-24
4.29E-25	0.48704038	2.07E-12	-0.727593	9.01E-32	-0.6758367	4.89E-26
4.14E-16	0.37821562	1.11E-07	-0.6662343	4.24E-25	-0.7385463	3.70E-33
4.52E-20	0.45064849	1.22E-10	-0.7572668	1.07E-35	-0.4851944	2.57E-12
1.93E-08	-0.5464823	8.52E-16	0.65419701	5.69E-24	0.60325802	1.02E-19
8.84E-09	0.26032858	0.00034523	-0.6169426	8.72E-21	-0.4432941	2.63E-10

4.76E-06	0.28808064	7.00E-05	-0.5538689	2.90E-16	-0.5022318	3.25E-13
3.80E-11	0.5943912	4.69E-19	-0.601647	1.35E-19	-0.3766431	1.26E-07
6.65E-18	0.4285243	1.16E-09	-0.6346794	3.01E-22	-0.4985916	5.11E-13
4.27E-10	-0.481752	3.85E-12	0.61638257	9.66E-21	0.26041273	0.00034365
1.89E-19	-0.4159458	3.91E-09	0.75843194	7.34E-36	0.53244679	6.17E-15
4.35E-82	-0.4812822	4.07E-12	0.9022328	9.32E-69	0.6588911	2.10E-24
6.15E-42	-0.4403713	3.55E-10	0.89796476	3.80E-67	0.75718175	1.10E-35
5.71E-22	-0.4702697	1.44E-11	0.83964846	2.15E-50	0.6679248	2.92E-25
4.30E-25	0.73268871	2.08E-32	-0.7267552	1.14E-31	-0.4283689	1.18E-09
9.16E-32	0.30365608	2.65E-05	-0.6013357	1.42E-19	-0.388422	4.67E-08
1.50E-57	-0.5328947	5.80E-15	0.89615437	1.75E-66	0.52751049	1.21E-14
2.11E-25	-0.4139551	4.71E-09	0.6979844	2.43E-28	0.76021436	4.08E-36
1.11E-27	-0.2362414	0.00120624	0.48381127	3.03E-12	0.02812613	0.70391645
4.63E-26	-0.2784107	0.00012441	0.59647279	3.29E-19	0.17739787	0.01570458
1.50E-28	-0.1396323	0.05800909	0.65798011	2.55E-24	0.12639235	0.08646414
1.68E-05	-0.3077781	2.03E-05	0.56189404	8.69E-17	-0.0680125	0.35764285
2.49E-30	-0.3218669	7.91E-06	0.6923181	9.88E-28	0.33107505	4.16E-06
3.30E-51	0.49035958	1.39E-12	-0.9307699	6.80E-82	-0.6887595	2.35E-27
1.52E-09	0.57312661	1.53E-17	-0.6150731	1.23E-20	-0.0574817	0.43705266
2.96E-46	-0.5312354	7.29E-15	0.70797075	1.89E-29	0.35732986	5.94E-07
8.17E-25	0.65375776	6.24E-24	-0.7314056	3.02E-32	-0.5591462	1.32E-16
6.34E-05	0.31537557	1.23E-05	-0.5817924	3.81E-18	-0.2673507	0.00023423
3.03E-06	-0.4016966	1.45E-08	0.52621628	1.44E-14	-0.0253747	0.7317121
5.20E-17	-0.4219942	2.20E-09	0.80708741	9.68E-44	0.46358283	3.03E-11
1.92E-28	0.38317459	7.31E-08	-0.6171619	8.37E-21	-0.4530258	9.49E-11
7.67E-46	-0.5425466	1.50E-15	0.88177687	1.26E-61	0.46819933	1.81E-11
7.72E-06	0.71662467	1.89E-30	-0.5591627	1.31E-16	-0.358279	5.52E-07
3.91E-05	0.36688401	2.80E-07	-0.5367982	3.37E-15	-0.3251988	6.28E-06
1.30E-26	-0.2962649	4.23E-05	0.57824512	6.75E-18	0.09974754	0.17673095
4.32E-64	0.52324989	2.15E-14	-0.7789478	6.14E-39	-0.4879671	1.85E-12
7.02E-21	0.63515564	2.74E-22	-0.7454223	4.60E-34	-0.7480787	2.02E-34
1.67E-19	-0.4375402	4.73E-10	0.7363892	7.03E-33	0.89842704	2.57E-67
4.74E-22	0.3833681	7.19E-08	-0.7606656	3.51E-36	-0.6568623	3.23E-24
1.47E-26	0.52157874	2.69E-14	-0.8193784	4.29E-46	-0.6165074	9.44E-21
5.03E-38	0.70559882	3.50E-29	-0.8758023	8.61E-60	-0.6333801	3.87E-22
1.73E-44	-0.5791612	5.83E-18	0.93086144	6.05E-82	0.64158095	7.64E-23
3.04E-11	0.43489741	6.18E-10	-0.5332886	5.49E-15	-0.6373146	1.79E-22
2.72E-25	0.50501578	2.29E-13	-0.7387828	3.45E-33	-0.8376426	6.08E-50
3.27E-19	0.61935466	5.58E-21	-0.6381119	1.53E-22	-0.7345196	1.22E-32
1.38E-06	0.49431721	8.62E-13	-0.6010933	1.48E-19	-0.502527	3.13E-13
1.39E-44	-0.3954464	2.53E-08	0.91233465	6.94E-73	0.64167182	7.50E-23
2.76E-14	0.34298105	1.76E-06	-0.6263545	1.50E-21	-0.708914	1.48E-29
1.45E-12	0.41627927	3.79E-09	-0.7151065	2.85E-30	-0.4502839	1.27E-10
8.89E-16	-0.3062268	2.24E-05	0.79345815	2.54E-41	0.44025426	3.59E-10
2.52E-33	-0.5536516	2.99E-16	0.86766979	1.94E-57	0.51297077	8.31E-14
2.68E-68	-0.5883703	1.29E-18	0.92216535	2.06E-77	0.78434915	8.37E-40

1.94E-26	0.48273794	3.43E-12	-0.6932736	7.82E-28		-0.4666465	2.16E-11
4.77E-20	-0.5856065	2.04E-18	0.80623263	1.39E-43		0.26684808	0.00024091
3.33E-49	0.54355747	1.30E-15	-0.6342227	3.29E-22		-0.5822652	3.52E-18
5.37E-23	0.47155175	1.24E-11	-0.7029185	6.97E-29		-0.47916	5.21E-12
2.20E-11	-0.4913231	1.24E-12	0.78410811	9.16E-40		0.46997966	1.49E-11
1.31E-21	-0.4208344	2.45E-09	0.71800026	1.30E-30		0.27751402	0.00013109
7.96E-05	0.23608008	0.00121589	-0.3987801	1.89E-08		-0.0825182	0.26413668
6.01E-08	-0.4816774	3.89E-12	0.60105696	1.49E-19		0.39588648	2.44E-08
9.61E-35	-0.4050846	1.07E-08	0.74518738	4.94E-34		0.6910656	1.34E-27
2.36E-11	-0.5050873	2.27E-13	0.80872743	4.81E-44		0.52924052	9.58E-15
8.52E-18	0.27410098	0.0001597	-0.5304162	8.16E-15		-0.5726186	1.65E-17
5.92E-29	-0.642433	6.43E-23	0.83022607	2.52E-48		0.56481126	5.57E-17
2.98E-28	-0.5462071	8.87E-16	0.88824181	9.95E-64		0.62370657	2.48E-21
8.71E-15	-0.5197452	3.43E-14	0.83037664	2.35E-48		0.71934776	9.00E-31
5.33E-20	-0.411905	5.70E-09	0.83061193	2.09E-48		0.25842369	0.00038284
9.38E-59	-0.5710403	2.12E-17	0.87440321	2.24E-59		0.48556809	2.46E-12
3.39E-40	-0.4600178	4.47E-11	0.80282005	5.80E-43		0.45039286	1.25E-10
4.99E-28	0.51085227	1.09E-13	-0.8835717	3.38E-62		-0.7386267	3.61E-33
5.20E-21	-0.525005	1.70E-14	0.80105575	1.20E-42		0.51418875	7.09E-14
1.71E-32	0.40277388	1.32E-08	-0.7834327	1.18E-39		-0.7808784	3.03E-39
4.11E-18	0.07422896	0.31529861	-0.3879068	4.88E-08		-0.1026448	0.16442799
3.55E-36	-0.553207	3.19E-16	0.84101607	1.05E-50		0.33454829	3.25E-06
0.00053932	-0.1981502	0.00685662	0.59557247	3.84E-19		0.50170955	3.47E-13
2.12E-22	-0.410045	6.78E-09	0.68463344	6.29E-27		0.2378943	0.00111129
6.31E-44	0.49104966	1.28E-12	-0.8106811	2.07E-44		-0.369656	2.24E-07
1.98E-36	-0.3965422	2.30E-08	0.8564187	1.97E-54		0.5171176	4.84E-14
6.51E-55	-0.3725911	1.76E-07	0.80291366	5.58E-43		0.41870012	3.01E-09
1.24E-38	-0.4299611	1.01E-09	0.83922542	2.68E-50		0.62599655	1.61E-21
6.87E-27	-0.3981594	1.99E-08	0.73079885	3.60E-32		0.12078611	0.10147693
2.01E-13	-0.3923941	3.31E-08	0.72813526	7.72E-32		0.64050914	9.47E-23
1.44E-13	0.42848266	1.17E-09	-0.6243505	2.19E-21		0.04164098	0.57358201
6.68E-19	-0.4989627	4.88E-13	0.86146997	9.49E-56		0.47735465	6.42E-12
6.05E-25	-0.5166989	5.12E-14	0.89544299	3.15E-66		0.68849023	2.50E-27
0.00406717	-0.3763119	1.30E-07	0.45307108	9.45E-11		0.52066775	3.03E-14
1.57E-33	-0.4381188	4.47E-10	0.69303423	8.29E-28		0.15244902	0.03830265
3.76E-24	-0.5261117	1.46E-14	0.9275616	3.70E-80		0.62759622	1.18E-21
1.19E-36	-0.5050074	2.29E-13	0.93523434	1.88E-84		0.71895091	1.00E-30
1.15E-20	0.45866521	5.18E-11	-0.606319	5.93E-20		-0.6502846	1.29E-23
4.39E-16	0.60503535	7.44E-20	-0.7344656	1.24E-32		-0.3229546	7.34E-06
8.42E-29	-0.3528314	8.40E-07	0.50974019	1.26E-13		0.28025938	0.00011163
6.61E-19	-0.4393094	3.96E-10	0.77844322	7.38E-39		0.34242039	1.83E-06
1.21E-56	-0.5431772	1.37E-15	0.86077029	1.45E-55		0.63916601	1.24E-22
4.65E-31	-0.6355456	2.54E-22	0.89490989	4.90E-66		0.68833359	2.60E-27
2.99E-30	-0.5996033	1.92E-19	0.90069822	3.61E-68		0.59664907	3.19E-19
1.37E-12	-0.6054596	6.90E-20	0.76880522	2.24E-37		0.65729962	2.95E-24
6.68E-07	0.12137322	0.09981419	-0.3992878	1.80E-08		-0.6818004	1.23E-26

4.57E-42	-0.2863456	7.77E-05	0.66059572	1.45E-24	0.41156879	5.88E-09
1.92E-27	-0.2005675	0.00619277	0.52734533	1.24E-14	0.14147479	0.05474487
1.39E-05	0.34249687	1.82E-06	-0.698013	2.41E-28	-0.2161854	0.00312122
5.79E-09	0.32660783	5.70E-06	-0.7398055	2.54E-33	-0.4043832	1.14E-08
2.55E-43	0.43879908	4.17E-10	-0.8171588	1.18E-45	-0.7093073	1.33E-29
8.95E-05	0.27892968	0.00012069	-0.6037232	9.36E-20	-0.3731623	1.68E-07
0.00424014	0.30835847	1.95E-05	-0.490202	1.42E-12	-0.6143577	1.40E-20
0.08950916	0.39255559	3.26E-08	-0.3723891	1.79E-07	-0.6855307	5.09E-27
4.87E-31	0.42440759	1.74E-09	-0.7203424	6.85E-31	-0.7221743	4.13E-31
4.67E-25	0.55712042	1.79E-16	-0.7535109	3.62E-35	-0.530264	8.33E-15
5.18E-25	-0.5705626	2.28E-17	0.87234258	9.02E-59	0.75516458	2.13E-35
1.01E-19	-0.4881281	1.82E-12	0.84210565	5.90E-51	0.6171149	8.44E-21
4.04E-33	0.52639248	1.41E-14	-0.7780463	8.52E-39	-0.6795516	2.08E-26
1.73E-14	0.63316752	4.04E-22	-0.7752035	2.37E-38	-0.3734673	1.64E-07
0.00015938	-0.2925409	5.33E-05	0.53719532	3.19E-15	0.09165622	0.21467103
1.59E-22	0.32623095	5.85E-06	-0.4869991	2.08E-12	-0.6986143	2.07E-28
4.34E-19	0.32494842	6.39E-06	-0.5983106	2.40E-19	-0.6234661	2.59E-21
5.64E-25	-0.2963898	4.20E-05	0.67497505	5.96E-26	0.09655463	0.19106309
1.22E-22	0.70926353	1.35E-29	-0.738369	3.90E-33	-0.5182771	4.16E-14
6.86E-14	0.68690381	3.66E-27	-0.5744933	1.23E-17	-0.4550262	7.67E-11
2.89E-22	-0.5856743	2.02E-18	0.88544952	8.34E-63	0.60514715	7.29E-20
2.61E-31	0.26072011	0.00033794	-0.7974689	5.16E-42	-0.3962897	2.35E-08
4.34E-23	0.39647029	2.31E-08	-0.8214524	1.65E-46	-0.6527692	7.68E-24
1.38E-33	-0.5255442	1.58E-14	0.93656003	3.00E-85	0.6917191	1.14E-27
5.13E-11	0.37034884	2.12E-07	-0.7730083	5.16E-38	-0.2535944	0.00049585
9.03E-36	-0.5411173	1.84E-15	0.91678325	7.28E-75	0.81713805	1.19E-45
1.83E-64	-0.4248988	1.66E-09	0.78666965	3.49E-40	0.35160287	9.22E-07
6.25E-23	-0.5727952	1.61E-17	0.834258	3.41E-49	0.60644453	5.80E-20
2.58E-53	-0.4909547	1.29E-12	0.91321158	2.88E-73	0.57430802	1.27E-17
1.68E-27	0.5145053	6.81E-14	-0.6526361	7.90E-24	-0.2064829	0.00480404
7.24E-26	0.55621572	2.04E-16	-0.8254636	2.52E-47	-0.4035725	1.23E-08
3.25E-26	0.27567323	0.00014586	-0.7363949	7.02E-33	-0.615829	1.07E-20
0.00033063	-0.4818475	3.81E-12	0.58538214	2.12E-18	0.59498449	4.24E-19
1.44E-11	0.26759598	0.00023104	-0.505557	2.14E-13	0.04161354	0.57383483
9.96E-26	-0.510744	1.11E-13	0.86339657	2.89E-56	0.79179229	4.88E-41
3.82E-18	-0.4770293	6.66E-12	0.77674586	1.36E-38	0.80374969	3.94E-43
0.64377015	0.25715056	0.00041005	-0.3733454	1.66E-07	-0.2481188	0.00066088
1.76E-44	0.45792023	5.61E-11	-0.7954765	1.14E-41	-0.3956193	2.50E-08
7.15E-41	-0.5481105	6.73E-16	0.94186795	1.29E-88	0.54758095	7.27E-16
7.38E-33	-0.5485708	6.30E-16	0.87239239	8.72E-59	0.43612134	5.46E-10
1.16E-07	-0.3419239	1.90E-06	0.60211242	1.24E-19	-0.0521304	0.48098265
6.72E-06	0.32779015	5.25E-06	-0.5312276	7.30E-15	-0.3188878	9.69E-06
1.96E-43	0.30439939	2.52E-05	-0.7381403	4.18E-33	-0.5021639	3.28E-13
5.61E-32	-0.4538912	8.65E-11	0.77959467	4.85E-39	0.39812823	2.00E-08
5.05E-10	-0.2794655	0.00011696	0.3828426	7.52E-08	-0.2075865	0.00457833
5.25E-08	0.42884569	1.13E-09	-0.5919708	7.06E-19	0.03047559	0.68048679

4.22E-18	-0.3619686	4.13E-07	0.74726485	2.60E-34	0.55226337	3.67E-16
2.64E-34	-0.5630963	7.24E-17	0.94439636	2.49E-90	0.68549131	5.13E-27
6.63E-08	0.27926724	0.00011832	-0.3475293	1.25E-06	0.18105963	0.01364768
1.41E-26	0.33307275	3.61E-06	-0.7401356	2.30E-33	-0.4691991	1.62E-11
2.77E-17	-0.419056	2.91E-09	0.81267506	8.67E-45	0.73244238	2.24E-32
5.66E-15	-0.3068779	2.15E-05	0.70308335	6.68E-29	0.53117744	7.35E-15
1.39E-22	-0.5046428	2.40E-13	0.85377841	9.20E-54	0.52933826	9.45E-15
8.93E-13	-0.360944	4.48E-07	0.70625419	2.95E-29	0.51368221	7.58E-14
1.30E-45	-0.5720062	1.82E-17	0.9070785	1.12E-70	0.68165808	1.27E-26
3.70E-18	0.38512943	6.19E-08	-0.6176134	7.70E-21	-0.3470402	1.30E-06
1.36E-20	-0.5566707	1.91E-16	0.86395946	2.03E-56	0.74316348	9.19E-34
3.21E-51	-0.5619189	8.66E-17	0.94484	1.22E-90	0.68236964	1.07E-26
7.44E-46	-0.2392537	0.00103839	0.73777155	4.66E-33	0.3847119	6.42E-08
7.90E-06	0.63290118	4.25E-22	-0.5966426	3.19E-19	-0.3366725	2.79E-06
2.90E-15	0.71496224	2.96E-30	-0.7151279	2.83E-30	-0.4559927	6.91E-11
1.70E-11	0.63424815	3.27E-22	-0.7318063	2.69E-32	-0.5125405	8.78E-14
0.05459455	-0.3306281	4.30E-06	0.28826279	6.92E-05	0.1299181	0.07797168
1.22E-35	-0.5814366	4.03E-18	0.9322995	9.44E-83	0.68808646	2.76E-27
2.96E-21	-0.6468363	2.63E-23	0.72652802	1.22E-31	0.78808728	2.04E-40
1.57E-23	-0.5454883	9.84E-16	0.83514672	2.18E-49	0.57919507	5.79E-18
7.61E-06	-0.2561209	0.00043336	0.53493055	4.38E-15	0.5043977	2.48E-13
1.42E-17	-0.4814728	3.98E-12	0.79642007	7.86E-42	0.72807867	7.84E-32
9.03E-13	-0.5504238	4.81E-16	0.63890745	1.30E-22	0.3893984	4.29E-08
1.24E-28	-0.4735579	9.91E-12	0.8253136	2.70E-47	0.59248195	6.48E-19
8.87E-21	-0.5162807	5.40E-14	0.74363972	7.94E-34	0.56878	3.01E-17
9.77E-60	-0.4360288	5.52E-10	0.85530011	3.80E-54	0.44265113	2.81E-10
5.66E-15	0.3927258	3.22E-08	-0.5187008	3.93E-14	-0.5114222	1.01E-13
3.44E-27	-0.5005566	4.00E-13	0.76894879	2.13E-37	0.37822464	1.11E-07
5.25E-28	0.30932741	1.83E-05	-0.6193545	5.58E-21	-0.5419432	1.63E-15
6.15E-34	0.42267515	2.06E-09	-0.8442371	1.89E-51	-0.692907	8.55E-28
1.47E-20	-0.5205668	3.07E-14	0.89640023	1.42E-66	0.57280539	1.60E-17
2.16E-13	0.44367337	2.53E-10	-0.7770073	1.24E-38	-0.6328722	4.28E-22
1.70E-15	-0.2978532	3.83E-05	0.67063239	1.59E-25	0.19193295	0.008864
2.02E-10	-0.1340195	0.06895406	0.58980541	1.02E-18	0.0468237	0.52679776
2.40E-11	-0.3833371	7.21E-08	0.58514403	2.20E-18	0.3724985	1.78E-07
9.07E-32	0.56536674	5.11E-17	-0.8082982	5.78E-44	-0.5865898	1.73E-18
2.69E-52	-0.3477589	1.23E-06	0.71328068	4.65E-30	0.31561565	1.21E-05
1.04E-15	-0.1063574	0.14961219	0.61394265	1.51E-20	0.1131999	0.12498416
1.85E-15	0.63646742	2.11E-22	-0.7175511	1.47E-30	-0.1443736	0.04991777
2.00E-23	-0.3098759	1.77E-05	0.57963632	5.40E-18	0.11511403	0.11868918
3.99E-06	-0.1986092	0.00672591	0.44045295	3.52E-10	0.01932977	0.79397245
3.94E-20	0.26192564	0.00031637	-0.7202251	7.07E-31	-0.3026149	2.83E-05
2.16E-48	-0.5415567	1.73E-15	0.93789781	4.55E-86	0.46643579	2.21E-11
1.17E-10	-0.1778098	0.01546041	0.46538381	2.48E-11	0.33197591	3.91E-06
3.43E-19	0.59514687	4.12E-19	-0.7775811	1.01E-38	-0.4426162	2.82E-10
1.93E-23	0.5216853	2.65E-14	-0.6687199	2.44E-25	-0.8129538	7.67E-45

8.08E-28	-0.5966561	3.19E-19	0.91814199	1.72E-75	0.73280355	2.01E-32
6.70E-08	-0.3986033	1.92E-08	0.71441905	3.43E-30	0.38029157	9.32E-08
2.05E-24	0.63232911	4.75E-22	-0.6537578	6.24E-24	-0.5979948	2.53E-19
1.00E-11	-0.3696025	2.25E-07	0.7770484	1.22E-38	0.68923141	2.09E-27
2.07E-32	-0.4016942	1.45E-08	0.59476387	4.40E-19	0.53034921	8.23E-15
6.13E-23	-0.5669406	4.01E-17	0.89567278	2.61E-66	0.61717366	8.35E-21
0.69765039	-0.400871	1.56E-08	0.27567405	0.00014586	0.24571995	0.00074804
3.78E-15	-0.350694	9.88E-07	0.57048837	2.31E-17	0.36534717	3.16E-07
5.25E-19	0.3107475	1.67E-05	-0.5897586	1.02E-18	-0.7419174	1.34E-33
1.68E-35	0.52958756	9.14E-15	-0.8130794	7.25E-45	-0.2872564	7.35E-05
4.53E-14	0.39151369	3.58E-08	-0.5460521	9.07E-16	-0.7052227	3.86E-29
2.93E-43	0.23215009	0.00147409	-0.7219048	4.45E-31	-0.4643085	2.79E-11
1.04E-13	0.39772791	2.07E-08	-0.5895259	1.06E-18	-0.4143744	4.53E-09
2.81E-24	-0.5261786	1.45E-14	0.87711383	3.47E-60	0.72764325	8.88E-32
7.74E-30	-0.5262776	1.43E-14	0.92350131	4.50E-78	0.79558498	1.10E-41
5.67E-19	-0.1983463	0.00680048	0.41387998	4.74E-09	0.035095	0.63531848
1.35E-12	-0.4781749	5.84E-12	0.81054494	2.20E-44	0.49931311	4.67E-13
7.07E-31	-0.6315942	5.48E-22	0.92427693	1.84E-78	0.6519538	9.11E-24
6.52E-12	-0.3326851	3.71E-06	0.34595095	1.41E-06	-0.0646379	0.38204951
2.36E-19	-0.5773011	7.86E-18	0.81568155	2.29E-45	0.65477797	5.03E-24
9.46E-14	-0.4766883	6.93E-12	0.72934797	5.46E-32	0.3610369	4.45E-07
1.85E-20	-0.452894	9.62E-11	0.77980316	4.50E-39	0.73259059	2.14E-32
6.73E-65	0.54027739	2.07E-15	-0.7945293	1.67E-41	-0.4708424	1.35E-11
1.90E-08	-0.4533021	9.22E-11	0.71326388	4.67E-30	0.23330579	0.00139339
4.08E-09	-0.4798338	4.82E-12	0.64593347	3.16E-23	0.21593101	0.00315745
1.36E-31	0.34573385	1.43E-06	-0.8385249	3.86E-50	-0.4731218	1.04E-11
7.41E-10	0.12395976	0.09274401	-0.5688841	2.97E-17	-0.5916123	7.50E-19
2.00E-20	-0.4199978	2.66E-09	0.65908941	2.01E-24	0.22051773	0.00255917
7.39E-39	0.25179024	0.00054546	-0.6945863	5.66E-28	-0.4453348	2.13E-10
6.13E-22	-0.2162727	0.00310887	0.65699048	3.15E-24	0.5922086	6.78E-19
1.11E-26	-0.4162586	3.79E-09	0.77783687	9.19E-39	0.7136262	4.24E-30
7.26E-05	-0.3068986	2.15E-05	0.56698469	3.98E-17	0.67991096	1.91E-26
4.46E-23	-0.5253837	1.62E-14	0.88690789	2.77E-63	0.72519049	1.78E-31
1.42E-20	0.53735495	3.12E-15	-0.7263279	1.29E-31	-0.2289833	0.00171759
2.95E-13	0.04201123	0.57017605	0.36847994	2.46E-07	0.10839326	0.1419289
1.11E-39	0.42930048	1.08E-09	-0.7373467	5.29E-33	-0.4815782	3.93E-12
4.37E-42	0.44819342	1.58E-10	-0.7837141	1.06E-39	-0.4755726	7.88E-12
2.94E-05	0.25377311	0.00049117	-0.5307334	7.81E-15	-0.5236445	2.04E-14
2.61E-30	-0.2807316	0.00010856	0.78384425	1.01E-39	0.29744665	3.93E-05
1.21E-22	-0.5218436	2.59E-14	0.79900228	2.78E-42	0.4331793	7.34E-10
4.61E-28	0.53677424	3.39E-15	-0.8813371	1.73E-61	-0.5335313	5.31E-15
4.87E-18	0.18780701	0.01046847	-0.5879418	1.39E-18	-0.5926084	6.34E-19
4.24E-13	0.36604882	2.99E-07	-0.5858015	1.97E-18	-0.6494289	1.54E-23
3.38E-29	0.38013125	9.45E-08	-0.7430338	9.56E-34	-0.7340652	1.39E-32
3.12E-31	0.4043579	1.14E-08	-0.6975334	2.72E-28	-0.2359008	0.00122671
7.89E-35	0.37230101	1.81E-07	-0.5747792	1.17E-17	-0.3612292	4.38E-07

4.64E-14	-0.4406449	3.45E-10	0.65484025	4.97E-24	0.7804458	3.55E-39
1.14E-14	-0.6176531	7.65E-21	0.83026822	2.47E-48	0.66043475	1.50E-24
1.44E-33	0.56888977	2.96E-17	-0.7857118	5.02E-40	-0.4778179	6.09E-12
2.77E-46	-0.3969584	2.22E-08	0.75461205	2.54E-35	0.3566432	6.27E-07
4.56E-40	-0.5629902	7.36E-17	0.66737451	3.29E-25	0.25576976	0.00044158
6.55E-14	-0.4240207	1.81E-09	0.67037502	1.69E-25	0.75625615	1.49E-35
2.78E-22	-0.527412	1.23E-14	0.88146466	1.58E-61	0.79722815	5.68E-42
1.46E-08	-0.5023753	3.19E-13	0.73174653	2.74E-32	0.56380228	6.50E-17
3.55E-11	0.31334009	1.41E-05	-0.7809024	3.01E-39	-0.2621672	0.00031221
6.89E-41	0.62406729	2.31E-21	-0.8803021	3.64E-61	-0.4466611	1.86E-10
6.10E-13	-0.3332144	3.58E-06	0.53337042	5.43E-15	-0.0990919	0.17960708
1.57E-08	-0.4646639	2.69E-11	0.7612626	2.88E-36	0.54797531	6.87E-16
0.6708737	-0.4147616	4.37E-09	0.38902144	4.44E-08	0.30816065	1.98E-05
0.00014002	-0.3520029	8.95E-07	0.59694969	3.03E-19	0.31752457	1.06E-05
5.90E-17	0.76085424	3.30E-36	-0.7492539	1.40E-34	-0.5582668	1.50E-16
5.05E-13	-0.4333413	7.22E-10	0.79122653	6.08E-41	0.58982766	1.01E-18
2.58E-20	-0.5420932	1.60E-15	0.8931713	2.02E-65	0.59434481	4.73E-19
0.28137174	-0.1683401	0.0219909	0.24090636	0.00095568	0.36437006	3.42E-07
7.37E-05	0.03276049	0.65799031	0.55688866	1.85E-16	0.37899627	1.04E-07
0.00050596	-0.3349904	3.15E-06	0.58921208	1.12E-18	0.4640903	2.86E-11
8.38E-05	-0.3139897	1.35E-05	0.50339408	2.81E-13	0.64091749	8.72E-23
2.72E-42	0.56196917	8.59E-17	-0.6964358	3.58E-28	-0.7874942	2.55E-40
1.15E-18	0.45062143	1.22E-10	-0.7703422	1.31E-37	-0.6548344	4.97E-24
4.03E-16	-0.335873	2.96E-06	0.62697831	1.33E-21	0.09178978	0.21400086
1.11E-40	-0.4396964	3.80E-10	0.92343985	4.83E-78	0.53698054	3.29E-15
9.47E-27	-0.2444548	0.00079816	0.74499945	5.23E-34	0.329204	4.75E-06
2.04E-32	-0.0993351	0.17853613	0.67480446	6.20E-26	0.3145814	1.29E-05
5.72E-13	0.02152292	0.77120976	-0.3267768	5.63E-06	-0.0205827	0.78094585
5.29E-32	0.58433873	2.51E-18	-0.5878096	1.42E-18	-0.5969252	3.04E-19
1.53E-60	-0.3962489	2.36E-08	0.75019253	1.04E-34	0.45402696	8.53E-11
7.81E-12	-0.368627	2.43E-07	0.80155716	9.78E-43	0.4288541	1.13E-09
3.44E-13	0.25224975	0.00053241	-0.5600858	1.14E-16	-0.5946684	4.47E-19
7.74E-26	0.4894893	1.54E-12	-0.8228502	8.62E-47	-0.5990468	2.11E-19
1.45E-27	0.56559525	4.93E-17	-0.7511224	7.74E-35	-0.7684742	2.51E-37
2.18E-46	0.40732522	8.71E-09	-0.8584019	6.08E-55	-0.5519885	3.82E-16
1.72E-11	0.2927191	5.27E-05	-0.6510555	1.10E-23	-0.535202	4.21E-15
0.00016563	0.29861745	3.65E-05	-0.580964	4.35E-18	-0.5887429	1.21E-18
6.93E-46	0.42490826	1.66E-09	-0.7914142	5.65E-41	-0.5389873	2.48E-15
4.57E-29	-0.5428812	1.43E-15	0.85330539	1.21E-53	0.69759964	2.67E-28
6.54E-32	0.07077687	0.33839267	-0.477945	6.00E-12	-0.1822774	0.013018
7.23E-39	0.33804307	2.53E-06	-0.6549317	4.87E-24	-0.4854571	2.49E-12
6.86E-21	-0.6051499	7.29E-20	0.88131176	1.76E-61	0.61527907	1.18E-20
5.47E-13	0.46505359	2.57E-11	-0.5281131	1.12E-14	-0.5978026	2.62E-19
1.59E-13	0.49587835	7.13E-13	-0.8217873	1.41E-46	-0.4127984	5.25E-09
1.17E-43	-0.5025523	3.12E-13	0.94307654	2.00E-89	0.74690679	2.90E-34
1.49E-45	-0.602341	1.19E-19	0.97059638	3.96E-115	0.73336158	1.71E-32

7.24E-16	0.29201564	5.50E-05	-0.5749457	1.14E-17	0.00774018	0.91672038
1.63E-13	0.47054798	1.39E-11	-0.6660909	4.38E-25	-0.7202847	6.96E-31
1.31E-07	-0.519934	3.34E-14	0.71169611	7.09E-30	0.42397647	1.81E-09
5.97E-24	-0.5199716	3.33E-14	0.92117978	6.23E-77	0.5761336	9.47E-18
6.48E-47	-0.5399045	2.18E-15	0.93035278	1.15E-81	0.76208129	2.19E-36
3.36E-10	-0.4070991	8.89E-09	0.71088905	8.78E-30	0.6250576	1.92E-21
0.00141863	-0.4193317	2.83E-09	0.58707661	1.60E-18	0.61154638	2.33E-20
3.13E-23	0.05471828	0.4594462	-0.421052	2.40E-09	-0.1680747	0.02220389
6.88E-18	0.56977494	2.58E-17	-0.839488	2.34E-50	-0.7237247	2.68E-31
4.65E-41	-0.5367347	3.40E-15	0.95979399	6.57E-103	0.76423947	1.06E-36
2.29E-41	-0.4418658	3.05E-10	0.91071198	3.45E-72	0.73291644	1.95E-32
3.29E-27	0.54865361	6.22E-16	-0.730771	3.63E-32	-0.3176059	1.06E-05
4.52E-22	-0.4639846	2.90E-11	0.70624025	2.96E-29	0.45773174	5.73E-11
2.75E-36	-0.4327628	7.65E-10	0.66998919	1.84E-25	0.24617208	0.00073084
0.12132491	-0.3142445	1.32E-05	0.3331164	3.60E-06	0.08636711	0.24242649
4.96E-24	-0.4913404	1.24E-12	0.55727859	1.74E-16	0.29166566	5.62E-05
1.42E-38	-0.4957769	7.21E-13	0.81695991	1.29E-45	0.37263326	1.76E-07
1.99E-30	-0.5167317	5.09E-14	0.76856376	2.43E-37	0.22939636	0.00168387
5.95E-40	-0.4267395	1.39E-09	0.60727587	5.00E-20	0.21685405	0.00302776
7.42E-20	-0.4901639	1.42E-12	0.82605899	1.89E-47	0.66921223	2.19E-25
4.80E-11	-0.3869882	5.28E-08	0.67587843	4.85E-26	0.15311158	0.03746026
1.50E-20	-0.4260086	1.49E-09	0.68538267	5.27E-27	0.199858	0.00638141
1.29E-38	-0.6107107	2.71E-20	0.92057557	1.22E-76	0.626483	1.46E-21
3.03E-45	-0.5697162	2.61E-17	0.8215616	1.57E-46	0.41182804	5.74E-09
4.30E-27	-0.4219921	2.20E-09	0.60303499	1.06E-19	0.11455937	0.12048737
3.30E-27	-0.54403	1.21E-15	0.63313349	4.07E-22	0.26638063	0.00024728
6.52E-43	-0.5916807	7.41E-19	0.86642458	4.30E-57	0.82310112	7.67E-47
7.19E-25	-0.4462914	1.93E-10	0.8274402	9.77E-48	0.79838989	3.56E-42
6.19E-15	0.82296685	8.16E-47	-0.7553234	2.02E-35	-0.5034435	2.79E-13
8.57E-16	-0.3647157	3.33E-07	0.78564559	5.14E-40	0.65430548	5.56E-24
5.18E-56	-0.5430523	1.39E-15	0.92465176	1.19E-78	0.51190904	9.53E-14
4.56E-34	-0.6171567	8.38E-21	0.94657649	7.07E-92	0.68677669	3.78E-27
1.93E-16	-0.5114994	1.00E-13	0.83579583	1.57E-49	0.54143967	1.75E-15
1.76E-20	0.66008599	1.62E-24	-0.670158	1.77E-25	-0.2966196	4.14E-05
1.14E-49	-0.3995814	1.76E-08	0.91614886	1.42E-74	0.68901241	2.21E-27
2.49E-16	0.25843233	0.00038266	-0.6588917	2.10E-24	-0.5691117	2.86E-17
1.83E-59	-0.5394337	2.33E-15	0.80033792	1.61E-42	0.39295738	3.15E-08
3.68E-06	-0.5969468	3.03E-19	0.7017399	9.41E-29	0.48773955	1.90E-12
4.16E-24	0.34794237	1.22E-06	-0.6838217	7.63E-27	-0.7004445	1.31E-28
7.34E-16	0.55203779	3.79E-16	-0.5656238	4.91E-17	-0.3738929	1.58E-07
2.78E-35	-0.4243392	1.75E-09	0.84436238	1.76E-51	0.42919711	1.09E-09
1.49E-15	-0.4935751	9.43E-13	0.81299363	7.53E-45	0.77089925	1.08E-37
1.92E-35	0.26313656	0.00029601	-0.5451695	1.03E-15	-0.2892278	6.53E-05
6.73E-20	0.30046585	3.25E-05	-0.5301389	8.47E-15	-0.4221676	2.16E-09
6.59E-16	0.3635119	3.66E-07	-0.6828772	9.53E-27	-0.3115032	1.59E-05
2.19E-55	-0.4556412	7.18E-11	0.74741008	2.48E-34	0.34925973	1.10E-06

2.99E-56	0.42422608	1.77E-09	-0.7837718	1.04E-39	-0.5115387	9.99E-14
7.15E-51	-0.5784892	6.49E-18	0.96370281	6.77E-107	0.65060601	1.21E-23
7.95E-55	-0.4608791	4.07E-11	0.91295465	3.73E-73	0.55788995	1.59E-16
9.73E-20	0.28926326	6.51E-05	-0.5319411	6.62E-15	-0.4711158	1.31E-11
6.96E-11	0.23112136	0.00154949	-0.531273	7.25E-15	0.1669372	0.02313714
2.02E-22	0.77965861	4.74E-39	-0.5586228	1.43E-16	-0.527487	1.22E-14
6.02E-37	0.28945587	6.44E-05	-0.5651124	5.32E-17	-0.397679	2.08E-08
3.78E-18	-0.3652875	3.18E-07	0.81019706	2.55E-44	0.32304342	7.29E-06
3.08E-19	-0.4614518	3.82E-11	0.8590723	4.06E-55	0.77512435	2.43E-38
4.17E-48	-0.5939549	5.05E-19	0.8838738	2.70E-62	0.76127801	2.87E-36
2.17E-44	-0.5002507	4.16E-13	0.94780546	8.90E-93	0.73016589	4.32E-32
3.77E-48	-0.6024762	1.16E-19	0.96613666	1.32E-109	0.62606791	1.58E-21
6.07E-23	0.53409059	4.92E-15	-0.7384663	3.79E-33	-0.2537255	0.00049241
2.63E-19	-0.2143603	0.00338973	0.62700301	1.33E-21	0.19046523	0.00940789
9.03E-17	0.70140488	1.03E-28	-0.6468557	2.62E-23	-0.297833	3.83E-05
2.74E-17	0.44191929	3.03E-10	-0.7665246	4.89E-37	-0.6953228	4.71E-28
9.73E-06	-0.1747053	0.01738491	0.19599558	0.007501	-0.1890735	0.00995067
4.25E-11	0.29495738	4.59E-05	-0.6367413	2.00E-22	-0.0340793	0.6451428
2.48E-14	-0.4771365	6.58E-12	0.78823402	1.93E-40	0.4016745	1.45E-08
1.30E-25	0.33335347	3.54E-06	-0.4649114	2.61E-11	-0.0755258	0.30689488
1.32E-29	-0.4420481	2.99E-10	0.84135469	8.78E-51	0.30807542	1.99E-05
1.04E-14	0.22321383	0.00225747	-0.4088507	7.57E-09	0.12670266	0.08568815
2.35E-37	-0.6161724	1.00E-20	0.90871169	2.38E-71	0.58786414	1.40E-18
8.94E-32	0.50652977	1.89E-13	-0.651784	9.44E-24	-0.3478062	1.23E-06
1.52E-45	-0.5242965	1.87E-14	0.92877352	8.35E-81	0.80063535	1.43E-42
1.57E-19	-0.5366394	3.45E-15	0.61690754	8.77E-21	0.32636452	5.80E-06
1.16E-16	0.72652831	1.22E-31	-0.6711596	1.41E-25	-0.6692854	2.15E-25
3.11E-34	0.51113378	1.05E-13	-0.8336618	4.60E-49	-0.6119912	2.15E-20
0.04317843	0.17575436	0.0167125	-0.4176382	3.33E-09	-0.6060098	6.26E-20
7.93E-28	0.78288132	1.45E-39	-0.8442436	1.88E-51	-0.3776144	1.17E-07
9.13E-17	0.4745327	8.87E-12	-0.4562468	6.72E-11	-0.1213503	0.09987882
1.44E-26	0.47532266	8.10E-12	-0.7463195	3.48E-34	-0.7414362	1.55E-33
2.47E-18	0.42169009	2.26E-09	-0.7314159	3.01E-32	-0.4236465	1.87E-09
0.42839037	0.17529437	0.0170045	-0.3438294	1.65E-06	-0.2944598	4.73E-05
1.31E-06	0.25266267	0.00052092	-0.3870539	5.26E-08	-0.5505124	4.75E-16
2.94E-15	-0.5524484	3.57E-16	0.81975948	3.61E-46	0.65045449	1.24E-23
3.45E-13	-0.4564723	6.56E-11	0.71504377	2.90E-30	0.73187866	2.63E-32
8.04E-08	0.15520345	0.03490259	-0.3891651	4.38E-08	-0.3997269	1.73E-08
3.26E-28	0.43449274	6.44E-10	-0.7339069	1.46E-32	-0.7638199	1.22E-36
1.35E-22	-0.292463	5.35E-05	0.75728226	1.07E-35	0.67850051	2.65E-26
1.35E-19	0.77643109	1.53E-38	-0.8014282	1.03E-42	-0.3191011	9.55E-06
5.76E-42	0.48625882	2.27E-12	-0.7774812	1.05E-38	-0.5604889	1.08E-16
1.44E-30	0.70954913	1.25E-29	-0.6453332	3.57E-23	-0.5174648	4.63E-14
6.15E-31	-0.5115959	9.92E-14	0.91162194	1.41E-72	0.60411834	8.74E-20
1.58E-32	-0.5186772	3.95E-14	0.83326855	5.60E-49	0.30342705	2.69E-05
4.18E-41	-0.5342484	4.81E-15	0.95070066	5.49E-95	0.66849828	2.57E-25

1.54E-40	0.35276086	8.44E-07	-0.644201	4.50E-23	-0.2650189	0.00026675
2.92E-17	0.37315777	1.68E-07	-0.599357	2.00E-19	-0.6753329	5.49E-26
1.58E-15	-0.6150593	1.23E-20	0.77511323	2.44E-38	0.47864044	5.53E-12
4.98E-07	-0.3872817	5.15E-08	0.73840651	3.86E-33	0.56312577	7.21E-17
7.53E-17	-0.4637737	2.96E-11	0.85655252	1.82E-54	0.64902205	1.68E-23
3.38E-05	-0.3106085	1.68E-05	0.67496019	5.98E-26	0.39349788	3.01E-08
2.68E-14	-0.2884035	6.86E-05	0.7918345	4.80E-41	0.51685504	5.01E-14
3.38E-35	0.38144802	8.46E-08	-0.6113147	2.43E-20	-0.4153741	4.12E-09
2.65E-06	0.12191631	0.09829537	-0.3226305	7.50E-06	-0.4583234	5.37E-11
7.53E-21	-0.4775672	6.26E-12	0.70688271	2.51E-29	0.44545055	2.10E-10
1.14E-33	-0.3895497	4.24E-08	0.7347489	1.14E-32	0.43714114	4.93E-10
3.97E-38	-0.4707329	1.36E-11	0.89662304	1.18E-66	0.61372052	1.57E-20
1.37E-15	0.35522987	6.99E-07	-0.6376046	1.69E-22	-0.7336285	1.58E-32
9.77E-32	0.39857784	1.92E-08	-0.8339455	3.99E-49	-0.5235903	2.05E-14
2.70E-09	0.3762867	1.30E-07	-0.7232986	3.02E-31	-0.5306688	7.88E-15
3.60E-08	0.22735487	0.0018566	-0.5415819	1.72E-15	-0.5277563	1.17E-14
2.21E-07	0.26175703	0.00031931	-0.5219114	2.57E-14	-0.6194887	5.45E-21
9.64E-14	0.31904536	9.59E-06	-0.5665728	4.24E-17	-0.5591631	1.31E-16
4.79E-12	0.27567859	0.00014582	-0.53449	4.65E-15	-0.5010424	3.77E-13
7.90E-15	0.38093252	8.83E-08	-0.6012303	1.45E-19	-0.5403937	2.03E-15
5.15E-16	0.3420256	1.89E-06	-0.6208802	4.20E-21	-0.6289165	9.19E-22
5.60E-12	0.3498314	1.06E-06	-0.6670172	3.57E-25	-0.5774647	7.65E-18
2.27E-30	0.49544099	7.52E-13	-0.8506659	5.44E-53	-0.5811714	4.21E-18
3.22E-22	-0.5547179	2.55E-16	0.69073743	1.45E-27	0.60619462	6.06E-20
0.0001218	0.33055289	4.32E-06	-0.680971	1.49E-26	-0.5027832	3.03E-13
2.23E-09	-0.2553836	0.00045079	0.595305	4.01E-19	0.31090104	1.65E-05
3.69E-20	-0.4576364	5.79E-11	0.79566391	1.06E-41	0.3740239	1.57E-07
4.38E-08	0.30345844	2.68E-05	-0.5033753	2.82E-13	-0.5801996	4.93E-18
2.27E-07	0.26791407	0.00022695	-0.6280758	1.08E-21	-0.3783882	1.09E-07
4.00E-05	-0.5266292	1.37E-14	0.65502712	4.77E-24	0.24207256	0.00090101
3.44E-59	-0.5636181	6.68E-17	0.92886404	7.47E-81	0.61690174	8.78E-21
9.54E-20	-0.2768335	0.00013638	0.53283162	5.85E-15	0.0686087	0.35343443
8.37E-51	-0.5245609	1.80E-14	0.80303952	5.30E-43	0.51594213	5.65E-14
1.19E-28	-0.422248	2.14E-09	0.79463381	1.60E-41	0.40664074	9.27E-09
1.57E-29	-0.3569567	6.12E-07	0.67697658	3.77E-26	0.19393789	0.00816611
2.08E-22	-0.3723115	1.80E-07	0.63511756	2.76E-22	0.31395242	1.35E-05
1.17E-31	0.37571357	1.36E-07	-0.583675	2.80E-18	-0.503977	2.61E-13
1.17E-22	-0.398077	2.01E-08	0.67801985	2.96E-26	0.15169151	0.03928525
0.00785127	-0.3958448	2.45E-08	0.53874058	2.57E-15	-0.0078302	0.91575483
2.02E-23	-0.4223553	2.12E-09	0.8161749	1.83E-45	0.31864103	9.85E-06
1.73E-14	-0.2098677	0.0041417	0.46557207	2.43E-11	0.22071791	0.00253558
1.83E-37	-0.4010174	1.54E-08	0.65658037	3.43E-24	0.17554055	0.01684768
1.82E-29	-0.4245508	1.72E-09	0.85142035	3.55E-53	0.4694256	1.58E-11
9.69E-68	-0.4965755	6.54E-13	0.83467808	2.76E-49	0.43518133	6.01E-10
2.51E-19	-0.4034749	1.24E-08	0.55978657	1.20E-16	0.67552676	5.25E-26
2.21E-10	-0.5337159	5.18E-15	0.67902804	2.34E-26	0.29652733	4.16E-05

8.42E-13	-0.5166474	5.15E-14	0.78987958	1.02E-40	0.43125826	8.88E-10
4.28E-36	-0.4290987	1.10E-09	0.90542403	5.20E-70	0.68967242	1.88E-27
7.45E-05	0.17975552	0.01435144	-0.4175737	3.35E-09	-0.5205932	3.06E-14
1.49E-54	0.52290943	2.25E-14	-0.8682981	1.29E-57	-0.5398973	2.18E-15
1.36E-16	0.35441547	7.44E-07	-0.6152797	1.18E-20	-0.0893981	0.22622962
5.69E-10	-0.5285249	1.06E-14	0.72226401	4.03E-31	0.25173815	0.00054696
2.85E-18	-0.4654498	2.46E-11	0.80747603	8.21E-44	0.75968296	4.86E-36
1.42E-29	-0.4303847	9.69E-10	0.75831675	7.62E-36	0.83056193	2.14E-48
8.59E-17	0.34025018	2.15E-06	-0.4843245	2.85E-12	-0.2617993	0.00031857
2.82E-45	-0.5453579	1.00E-15	0.70618396	3.01E-29	0.38679219	5.37E-08
9.97E-17	-0.1584373	0.03124035	0.47320158	1.03E-11	0.0045138	0.9513763
3.52E-24	0.53601076	3.77E-15	-0.8795812	6.10E-61	-0.4798539	4.81E-12
1.15E-15	0.71748138	1.50E-30	-0.5943776	4.70E-19	-0.2432279	0.0008497
2.43E-09	-0.5014682	3.57E-13	0.58963567	1.04E-18	0.76704716	4.09E-37
3.10E-77	-0.5061931	1.98E-13	0.87044101	3.19E-58	0.52023938	3.21E-14
2.90E-17	0.74181218	1.39E-33	-0.6403416	9.79E-23	-0.4444244	2.34E-10
7.51E-51	-0.5872244	1.56E-18	0.94747936	1.55E-92	0.58154762	3.96E-18
3.45E-39	0.31411964	1.34E-05	-0.7868626	3.25E-40	-0.601164	1.46E-19
1.52E-18	0.43097334	9.14E-10	-0.7398089	2.54E-33	-0.1635004	0.02616465
5.55E-60	-0.5753743	1.07E-17	0.95271471	1.32E-96	0.6899892	1.74E-27
3.21E-06	-0.2049765	0.00512824	0.45997863	4.49E-11	0.20637979	0.00482562
4.35E-10	-0.3561025	6.53E-07	0.66672556	3.80E-25	0.10214036	0.16652269
2.32E-37	-0.5477869	7.06E-16	0.77335724	4.56E-38	0.431376	8.78E-10
4.59E-43	-0.4576584	5.78E-11	0.70432105	4.86E-29	0.18735116	0.01066058
2.07E-18	0.41272066	5.29E-09	-0.6052641	7.14E-20	-0.1346022	0.06774468
1.06E-08	0.32031491	8.79E-06	-0.5585228	1.45E-16	-0.0092292	0.90077542
2.82E-23	0.41187545	5.72E-09	-0.696624	3.41E-28	-0.6506922	1.18E-23
6.46E-40	-0.5769728	8.28E-18	0.9212663	5.65E-77	0.75525912	2.06E-35
0.39920148	0.25244098	0.00052706	-0.4534057	9.11E-11	0.0262168	0.72316576
3.02E-24	-0.394639	2.72E-08	0.61062309	2.75E-20	0.26381476	0.00028514
1.43E-27	-0.5947959	4.38E-19	0.8870885	2.41E-63	0.6845426	6.43E-27
7.14E-07	-0.4368437	5.08E-10	0.72114661	5.49E-31	0.36602334	3.00E-07
1.40E-27	0.25886626	0.00037378	-0.5461396	8.96E-16	-0.5630733	7.26E-17
2.98E-34	0.38748539	5.06E-08	-0.7224679	3.80E-31	-0.6406847	9.14E-23
3.25E-38	0.2444082	0.00080006	-0.6857587	4.82E-27	-0.4503909	1.25E-10
1.16E-09	-0.3895299	4.25E-08	0.71576091	2.39E-30	0.5713217	2.03E-17
9.98E-40	0.32611556	5.90E-06	-0.8189436	5.24E-46	-0.5181694	4.22E-14
2.58E-14	0.39008873	4.05E-08	-0.5886003	1.24E-18	-0.2782752	0.00012539
2.08E-13	0.33243774	3.78E-06	-0.643163	5.55E-23	0.01652926	0.82329138
3.46E-47	0.38699313	5.28E-08	-0.7328408	1.99E-32	-0.5465357	8.46E-16
7.90E-20	0.74492363	5.36E-34	-0.7057985	3.32E-29	-0.5737821	1.38E-17
2.90E-09	-0.4291859	1.09E-09	0.72653463	1.22E-31	0.36279838	3.87E-07
4.09E-10	0.42117912	2.37E-09	-0.7363129	7.19E-33	-0.2497786	0.00060616
1.40E-16	0.40987072	6.89E-09	-0.6200692	4.89E-21	-0.0230987	0.75497366
6.04E-37	-0.4716415	1.23E-11	0.7388767	3.35E-33	0.27584177	0.00014445
2.14E-07	0.32297753	7.33E-06	-0.7020635	8.67E-29	-0.2779234	0.000128

0.00737884	-0.2774679	0.00013144	0.46989152	1.50E-11		0.05700778	0.44084847
7.20E-26	-0.4108544	6.29E-09	0.63464628	3.03E-22		0.16725568	0.0228725
7.63E-24	0.50258883	3.11E-13	-0.9137288	1.71E-73		-0.5480387	6.80E-16
2.53E-14	-0.2670428	0.0002383	0.80373587	3.97E-43		0.65117468	1.07E-23
1.25E-23	-0.5411398	1.83E-15	0.88113735	2.00E-61		0.55197574	3.83E-16
1.67E-18	0.3820942	8.01E-08	-0.7375857	4.93E-33		-0.7568449	1.23E-35
1.32E-12	0.21699584	0.00300827	-0.4886932	1.70E-12		-0.0357788	0.62874005
2.33E-38	0.39791274	2.04E-08	-0.8171323	1.19E-45		-0.672486	1.05E-25
1.54E-45	0.51348418	7.77E-14	-0.8357309	1.62E-49		-0.6582623	2.40E-24
9.42E-37	0.27408786	0.00015982	-0.760528	3.68E-36		-0.4573647	5.96E-11
3.25E-38	-0.57797	7.06E-18	0.80613231	1.45E-43		0.49900134	4.85E-13
3.35E-13	0.43108337	9.04E-10	-0.7166818	1.86E-30		-0.5151664	6.25E-14
1.35E-39	0.43797934	4.53E-10	-0.6450889	3.75E-23		-0.3944018	2.78E-08
3.44E-66	-0.4823692	3.59E-12	0.83151232	1.34E-48		0.55514256	2.40E-16
3.14E-07	-0.516299	5.39E-14	0.62258609	3.06E-21		0.51635831	5.35E-14
7.87E-09	-0.3659056	3.03E-07	0.69265656	9.09E-28		0.56112285	9.77E-17
9.83E-38	0.32773845	5.27E-06	-0.6450877	3.76E-23		-0.3592125	5.13E-07
3.08E-08	0.57708174	8.14E-18	-0.4998388	4.38E-13		-0.5813258	4.11E-18
9.64E-13	-0.471903	1.20E-11	0.75772561	9.25E-36		0.63600904	2.31E-22
1.50E-22	-0.4339939	6.76E-10	0.76509943	7.94E-37		0.32075679	8.53E-06
0.00013277	-0.3356742	3.00E-06	0.58801173	1.37E-18		0.48764784	1.92E-12
9.65E-26	-0.5131246	8.14E-14	0.79715125	5.86E-42		0.48045246	4.49E-12
2.52E-39	-0.3808008	8.93E-08	0.79908563	2.69E-42		0.49630191	6.77E-13
1.63E-50	0.45494186	7.74E-11	-0.675248	5.60E-26		-0.5023151	3.22E-13
5.68E-34	0.43225717	8.04E-10	-0.783762	1.04E-39		-0.3178496	1.04E-05
3.54E-18	-0.4847708	2.70E-12	0.78389014	9.94E-40		0.58639767	1.79E-18
2.45E-23	0.44335645	2.61E-10	-0.7929724	3.08E-41		-0.733484	1.65E-32
4.00E-35	-0.5652174	5.23E-17	0.91688404	6.55E-75		0.59513218	4.13E-19
3.09E-19	-0.4186991	3.01E-09	0.79550571	1.13E-41		0.74307947	9.43E-34
3.57E-13	-0.3180368	1.03E-05	0.40007818	1.68E-08		-0.1230771	0.09511041
1.65E-33	-0.5971616	2.92E-19	0.67665382	4.06E-26		0.26552074	0.00025941
6.05E-08	-0.3573461	5.93E-07	0.56568896	4.86E-17		0.46553465	2.44E-11
1.87E-40	-0.5404022	2.03E-15	0.86929414	6.76E-58		0.50866543	1.44E-13
8.33E-08	-0.3683174	2.49E-07	0.67744563	3.38E-26		0.38306882	7.38E-08
2.03E-17	0.3215172	8.10E-06	-0.6513939	1.02E-23		-0.4023534	1.37E-08
1.62E-09	-0.3896968	4.19E-08	0.63878976	1.33E-22		0.55469352	2.56E-16
3.29E-39	0.36670876	2.84E-07	-0.6207018	4.35E-21		-0.2522468	0.00053249
4.40E-05	0.37215841	1.83E-07	-0.6154534	1.15E-20		0.05848633	0.42906919
5.20E-29	0.76405103	1.13E-36	-0.8324329	8.49E-49		-0.5921211	6.88E-19
0.00080144	-0.2284896	0.00175869	0.49666163	6.47E-13		0.10072905	0.17248907
1.35E-06	-0.3425011	1.82E-06	0.6860674	4.47E-27		0.52687395	1.32E-14
2.61E-37	0.41570554	4.00E-09	-0.8225414	9.96E-47		-0.7812217	2.67E-39
2.41E-19	-0.5289129	1.00E-14	0.58088828	4.41E-18		0.14756537	0.04501766
7.62E-09	0.33870969	2.41E-06	-0.647934	2.10E-23		-0.0435497	0.55612674
4.42E-10	-0.4796053	4.95E-12	0.81280131	8.20E-45		0.59386133	5.13E-19
2.74E-18	-0.2542508	0.00047886	0.64082612	8.89E-23		0.06791979	0.35830017

8.77E-45	-0.5008905	3.84E-13	0.90442089	1.30E-69	0.52226117	2.45E-14
1.05E-19	-0.4020104	1.41E-08	0.81896987	5.18E-46	0.59655162	3.24E-19
7.10E-20	-0.2470645	0.00069796	0.76393962	1.18E-36	0.35649255	6.34E-07
3.36E-10	-0.3754906	1.39E-07	0.59605726	3.53E-19	0.09701939	0.18892575
2.16E-34	-0.4975659	5.79E-13	0.93962059	3.75E-87	0.61956406	5.37E-21
1.74E-27	-0.3494526	1.09E-06	0.71903538	9.80E-31	0.19823427	0.00683251
5.81E-12	-0.4300873	9.97E-10	0.79672901	6.94E-42	0.53741735	3.09E-15
7.27E-08	0.40741685	8.63E-09	-0.5842095	2.56E-18	-0.4401331	3.64E-10
2.55E-28	-0.4547192	7.92E-11	0.88285646	5.72E-62	0.64574337	3.29E-23
7.31E-34	-0.5830598	3.10E-18	0.88451188	1.68E-62	0.44261485	2.82E-10
4.26E-28	0.72813067	7.73E-32	-0.7139238	3.91E-30	-0.4329074	7.54E-10
5.80E-42	-0.37451	1.51E-07	0.73466174	1.17E-32	0.37129186	1.96E-07
9.89E-22	0.3539419	7.72E-07	-0.5320121	6.55E-15	-0.4121379	5.58E-09
9.03E-17	-0.2612217	0.00032881	0.4809693	4.22E-12	0.02902428	0.6949254
2.74E-18	-0.4283675	1.18E-09	0.64980683	1.42E-23	0.18407407	0.01213554
0.72559634	-0.2865502	7.67E-05	0.22653472	0.00193042	0.57266383	1.64E-17
1.08E-17	-0.4714685	1.26E-11	0.84978039	8.95E-53	0.71558825	2.50E-30
3.09E-20	-0.4418533	3.05E-10	0.57224563	1.75E-17	0.21310854	0.00358577
2.98E-19	-0.2877231	7.15E-05	0.62266615	3.01E-21	0.83807062	4.88E-50
2.06E-31	0.4514331	1.12E-10	-0.9157678	2.11E-74	-0.5799822	5.10E-18
8.85E-64	-0.5334333	5.39E-15	0.92350317	4.49E-78	0.64990702	1.39E-23
2.16E-10	-0.410984	6.21E-09	0.76584296	6.17E-37	0.63934638	1.19E-22
4.55E-43	-0.5724891	1.69E-17	0.93538552	1.52E-84	0.58186903	3.76E-18
4.51E-30	0.11313368	0.12520649	-0.5703133	2.37E-17	-0.3534134	8.03E-07
2.68E-42	0.28414704	8.86E-05	-0.6802193	1.78E-26	-0.4676169	1.94E-11
2.01E-11	-0.4287023	1.14E-09	0.73156233	2.88E-32	0.64778156	2.16E-23
6.76E-57	-0.5972973	2.86E-19	0.84226145	5.43E-51	0.65568955	4.15E-24
1.08E-06	-0.4964557	6.64E-13	0.72375657	2.66E-31	0.61592597	1.05E-20
7.49E-20	0.7117127	7.06E-30	-0.5552278	2.37E-16	-0.4862673	2.27E-12
2.19E-09	0.74684625	2.96E-34	-0.7174047	1.53E-30	-0.5255123	1.59E-14
8.06E-44	-0.4424903	2.86E-10	0.8054456	1.94E-43	0.32549104	6.16E-06
3.65E-44	0.39923754	1.81E-08	-0.7143949	3.45E-30	-0.4964461	6.65E-13
9.93E-25	-0.5412133	1.81E-15	0.91132856	1.88E-72	0.77251478	6.14E-38
7.47E-17	-0.5351598	4.24E-15	0.75578978	1.74E-35	0.80411315	3.39E-43
6.08E-14	0.20921648	0.00426236	-0.5981361	2.47E-19	-0.5415866	1.72E-15
9.05E-21	0.68912504	2.15E-27	-0.8475266	3.14E-52	-0.3329261	3.65E-06
4.26E-18	0.20661402	0.0047767	-0.7115841	7.30E-30	-0.2311051	0.00155071
2.86E-47	-0.5057424	2.09E-13	0.93050624	9.51E-82	0.75626101	1.49E-35
2.19E-60	-0.5296109	9.11E-15	0.9472678	2.22E-92	0.69893592	1.91E-28
2.91E-07	-0.4983779	5.24E-13	0.57355574	1.43E-17	0.4451796	2.16E-10
3.80E-41	-0.3317993	3.95E-06	0.60712741	5.14E-20	0.34955947	1.08E-06
6.24E-27	-0.4656233	2.42E-11	0.84929422	1.18E-52	0.53438714	4.72E-15
9.67E-13	0.11279482	0.12634886	-0.3795929	9.88E-08	-0.3990835	1.83E-08
1.56E-36	-0.4684502	1.76E-11	0.72775768	8.59E-32	0.57177867	1.89E-17
1.79E-21	-0.5682043	3.30E-17	0.75929291	5.53E-36	0.27008373	0.00020083
1.41E-69	-0.4112253	6.08E-09	0.75903988	6.01E-36	0.42806723	1.22E-09

4.85E-38	-0.5994961	1.96E-19	0.96787244	1.16E-111	0.66025688	1.56E-24
2.21E-09	-0.414359	4.53E-09	0.7013707	1.03E-28	0.27930985	0.00011803
7.74E-15	-0.3806258	9.07E-08	0.63361289	3.70E-22	0.15974188	0.02985833
1.48E-38	-0.4380976	4.47E-10	0.90647386	1.97E-70	0.47682107	6.83E-12
3.95E-22	-0.5225996	2.35E-14	0.88835507	9.12E-64	0.75370349	3.40E-35
6.37E-32	0.27470219	0.00015427	-0.6086058	3.95E-20	-0.3136529	1.38E-05
2.39E-19	-0.4696283	1.55E-11	0.83561759	1.71E-49	0.49818985	5.37E-13
7.46E-06	0.44639282	1.91E-10	-0.6855093	5.11E-27	-0.1847069	0.01183749
1.23E-23	-0.3524925	8.62E-07	0.80933581	3.70E-44	0.43715284	4.92E-10
6.58E-33	-0.4949866	7.95E-13	0.90061079	3.89E-68	0.46047353	4.26E-11
8.23E-07	0.29359743	4.99E-05	-0.5213147	2.78E-14	-0.1751017	0.01712809
8.83E-16	0.65020843	1.31E-23	-0.7397675	2.57E-33	-0.2202187	0.0025948
6.11E-19	0.45824245	5.42E-11	-0.7788526	6.36E-39	-0.285158	8.34E-05
1.39E-29	-0.6247073	2.05E-21	0.92868733	9.29E-81	0.62740113	1.23E-21
1.37E-18	-0.2523036	0.00053089	0.69349148	7.41E-28	0.51178983	9.67E-14
1.92E-11	-0.184564	0.0119042	0.40130454	1.50E-08	-0.017407	0.81407332
4.57E-31	0.2578168	0.00039559	-0.6346028	3.05E-22	-0.587308	1.54E-18
2.92E-09	0.52151297	2.71E-14	-0.5158433	5.72E-14	-0.6975144	2.73E-28
4.53E-16	0.39501565	2.63E-08	-0.6634406	7.83E-25	-0.4265297	1.41E-09
3.20E-44	-0.3485825	1.16E-06	0.67603674	4.67E-26	0.44335507	2.61E-10
2.45E-07	0.35427509	7.52E-07	-0.7105929	9.49E-30	-0.494174	8.77E-13
5.54E-05	0.65428796	5.58E-24	-0.6456506	3.35E-23	-0.1755353	0.01685098
4.67E-40	-0.3439922	1.63E-06	0.56130362	9.51E-17	0.27083537	0.00019245
1.61E-38	-0.5576627	1.65E-16	0.93110127	4.45E-82	0.61760378	7.72E-21
5.08E-09	-0.2983921	3.70E-05	0.70247747	7.80E-29	0.35374499	7.83E-07
1.81E-30	-0.5114013	1.02E-13	0.89614568	1.76E-66	0.7464593	3.34E-34
7.90E-17	-0.4174055	3.40E-09	0.78936635	1.25E-40	0.55043181	4.80E-16
5.83E-36	0.18931494	0.00985457	-0.5012568	3.67E-13	-0.2460047	0.00073716
3.58E-16	0.53488644	4.40E-15	-0.7040872	5.17E-29	-0.600933	1.52E-19
1.09E-23	-0.4169179	3.56E-09	0.89464575	6.09E-66	0.72236999	3.91E-31
7.72E-14	0.29063808	5.99E-05	-0.6647429	5.89E-25	-0.6540233	5.90E-24
1.51E-10	-0.5259179	1.50E-14	0.61060991	2.76E-20	0.69835629	2.21E-28
3.77E-06	-0.4571465	6.10E-11	0.66206779	1.06E-24	0.47177998	1.21E-11
1.39E-24	-0.4134944	4.92E-09	0.8438489	2.32E-51	0.6198913	5.05E-21
1.11E-20	-0.4953644	7.59E-13	0.85806856	7.41E-55	0.660451	1.50E-24
1.29E-36	-0.4753037	8.12E-12	0.84657359	5.30E-52	0.39637413	2.33E-08
9.13E-34	0.41762434	3.33E-09	-0.7932949	2.71E-41	-0.7204254	6.69E-31
4.21E-14	0.24025435	0.00098756	-0.5489829	5.93E-16	-0.4830111	3.33E-12
5.23E-10	-0.5454012	9.96E-16	0.70739374	2.20E-29	0.57996753	5.12E-18
9.72E-11	0.72127279	5.30E-31	-0.5731619	1.52E-17	-0.6322413	4.84E-22
1.56E-16	-0.4295595	1.05E-09	0.78428025	8.59E-40	0.64336957	5.32E-23
1.65E-37	-0.3774372	1.18E-07	0.58201873	3.67E-18	0.48533867	2.53E-12
2.36E-66	-0.5016552	3.49E-13	0.93947125	4.67E-87	0.58005568	5.04E-18
9.74E-12	0.43781547	4.60E-10	-0.8272574	1.07E-47	-0.5791406	5.85E-18
1.00E-22	-0.4523877	1.02E-10	0.78825125	1.91E-40	0.42278818	2.03E-09
2.69E-25	-0.5398704	2.19E-15	0.91843788	1.25E-75	0.74374719	7.69E-34

2.47E-11	0.23251491	0.00144817	-0.5803352	4.82E-18	0.07066637	0.33914929
7.55E-20	-0.4665378	2.18E-11	0.87537686	1.15E-59	0.58422923	2.56E-18
3.72E-24	0.39887065	1.87E-08	-0.7959536	9.46E-42	-0.8170783	1.22E-45
8.39E-05	0.22550387	0.002027	-0.4450742	2.19E-10	-0.6567017	3.35E-24
0.00261092	0.16382148	0.02586813	-0.3800379	9.52E-08	-0.5373478	3.12E-15
2.45E-28	0.49124451	1.25E-12	-0.5893892	1.09E-18	-0.5032353	2.87E-13
3.75E-20	-0.5913521	7.84E-19	0.80376381	3.92E-43	0.55029191	4.90E-16
4.38E-22	-0.4317765	8.44E-10	0.58487879	2.30E-18	0.12453128	0.09123688
7.97E-31	-0.5011158	3.74E-13	0.74390922	7.31E-34	0.60894959	3.71E-20
4.42E-16	0.30899805	1.87E-05	-0.5974739	2.77E-19	-0.5579176	1.58E-16
1.21E-18	-0.5117929	9.67E-14	0.83319841	5.80E-49	0.56661742	4.22E-17
4.83E-05	-0.3682338	2.51E-07	0.60486655	7.66E-20	0.54386547	1.24E-15
3.66E-05	-0.3627113	3.90E-07	0.66543526	5.06E-25	0.5130374	8.24E-14
2.19E-41	-0.4853158	2.54E-12	0.94145067	2.44E-88	0.68216356	1.13E-26
4.13E-19	-0.3287169	4.92E-06	0.71801002	1.30E-30	0.69015004	1.68E-27
3.29E-49	-0.4413455	3.21E-10	0.78534052	5.77E-40	0.4273017	1.31E-09
7.69E-35	0.43396581	6.78E-10	-0.695499	4.51E-28	-0.4823082	3.61E-12
2.94E-44	0.31026679	1.72E-05	-0.6875876	3.11E-27	-0.4897268	1.50E-12
7.99E-13	0.28256272	9.74E-05	-0.7170214	1.70E-30	-0.5562013	2.05E-16
5.45E-15	-0.4433226	2.62E-10	0.77221685	6.81E-38	0.6764023	4.30E-26
4.33E-21	-0.5675685	3.64E-17	0.90097082	2.84E-68	0.66004465	1.64E-24
1.66E-11	-0.4772522	6.50E-12	0.74051525	2.05E-33	0.50353935	2.76E-13
0.00020745	-0.3942915	2.80E-08	0.5420212	1.62E-15	0.57216151	1.78E-17
2.75E-18	-0.4173769	3.41E-09	0.77189706	7.62E-38	0.5859379	1.93E-18
2.64E-35	-0.5526079	3.49E-16	0.92177903	3.18E-77	0.69233995	9.83E-28
3.45E-37	-0.2635214	0.00028979	0.59492153	4.29E-19	0.11972295	0.10454348
4.41E-50	-0.6038989	9.08E-20	0.91827556	1.49E-75	0.48033063	4.55E-12
5.61E-42	0.715992	2.24E-30	-0.734648	1.17E-32	-0.5604695	1.08E-16
4.43E-16	0.23643391	0.00119481	-0.411652	5.84E-09	-0.3201452	8.90E-06
1.94E-32	-0.4858862	2.37E-12	0.93306277	3.47E-83	0.63403146	3.41E-22
4.32E-11	0.33136968	4.08E-06	-0.5257114	1.55E-14	-0.3671192	2.75E-07
9.99E-29	-0.371674	1.90E-07	0.62684731	1.37E-21	0.15188393	0.03903367
2.25E-07	-0.2450258	0.00077516	0.37592207	1.34E-07	-0.0079659	0.91430131
2.30E-14	-0.5664459	4.33E-17	0.53819869	2.77E-15	0.24370375	0.00082935
7.50E-24	0.76998311	1.49E-37	-0.6176121	7.71E-21	-0.5974843	2.77E-19
1.23E-40	0.33128723	4.10E-06	-0.6943224	6.04E-28	-0.4899024	1.47E-12
1.20E-27	0.29752951	3.91E-05	-0.6026428	1.13E-19	-0.448823	1.48E-10
5.99E-58	0.54767162	7.18E-16	-0.8230637	7.80E-47	-0.4646344	2.70E-11
5.64E-07	0.26969595	0.00020528	-0.451509	1.11E-10	-0.6788551	2.44E-26
0.00057162	-0.2486167	0.000644	0.50263041	3.09E-13	0.61796667	7.22E-21
1.26E-38	0.79581321	1.00E-41	-0.7570436	1.16E-35	-0.6064255	5.82E-20
6.58E-43	-0.4164906	3.71E-09	0.80925474	3.83E-44	0.61375219	1.56E-20
1.03E-06	0.33540228	3.06E-06	-0.6856795	4.91E-27	-0.1657127	0.02417929
8.38E-27	-0.5139463	7.32E-14	0.85220311	2.27E-53	0.42805095	1.22E-09
6.41E-08	-0.4258874	1.51E-09	0.69821956	2.29E-28	0.54885246	6.05E-16
3.75E-43	0.40058188	1.60E-08	-0.7640491	1.13E-36	-0.4687792	1.70E-11

1.69E-49	-0.3985085	1.93E-08	0.60349572	9.75E-20	0.3728714	1.72E-07
9.40E-11	0.7910747	6.45E-41	-0.645738	3.29E-23	-0.3785894	1.07E-07
3.16E-21	-0.3960886	2.39E-08	0.49768646	5.71E-13	0.02787065	0.70648157
4.40E-58	-0.5072935	1.72E-13	0.74559774	4.35E-34	0.51298136	8.30E-14
7.33E-25	-0.5378799	2.90E-15	0.87272641	6.97E-59	0.80981244	3.01E-44
0.00420266	0.14921349	0.04264915	-0.4294515	1.06E-09	-0.5568334	1.86E-16
0.00818301	0.24510302	0.0007721	-0.4832086	3.25E-12	0.10223807	0.16611538
1.13E-35	-0.3518281	9.07E-07	0.62649392	1.46E-21	0.29728593	3.97E-05
0.34180301	-0.1925674	0.00863766	0.3785926	1.07E-07	0.59331563	5.63E-19
4.25E-08	-0.430006	1.01E-09	0.71416866	3.66E-30	0.24579912	0.000745
1.35E-30	0.3670269	2.77E-07	-0.7572577	1.08E-35	-0.446407	1.91E-10
5.90E-12	-0.5105368	1.14E-13	0.72318652	3.11E-31	0.72146021	5.03E-31
1.04E-09	0.27764755	0.00013007	-0.6224006	3.16E-21	-0.5396753	2.25E-15
4.15E-40	-0.3539731	7.70E-07	0.61264669	1.91E-20	0.33837855	2.47E-06
1.21E-22	-0.5175321	4.59E-14	0.85060247	5.64E-53	0.60546991	6.89E-20
3.15E-28	-0.1924402	0.00868262	0.84104339	1.03E-50	0.54579854	9.41E-16
1.03E-14	0.4949967	7.94E-13	-0.7280366	7.94E-32	-0.2165255	0.00307336
4.55E-34	-0.4728767	1.07E-11	0.70221318	8.34E-29	0.21406406	0.00343523
5.53E-09	-0.5789787	6.00E-18	0.69387562	6.74E-28	0.67152403	1.30E-25
6.17E-13	0.78235671	1.76E-39	-0.5814863	4.00E-18	-0.3926583	3.24E-08
8.13E-34	0.41319783	5.06E-09	-0.8328922	6.76E-49	-0.6492986	1.58E-23
1.03E-15	-0.1946342	0.00793541	0.68389934	7.49E-27	0.25475893	0.00046608
1.29E-23	0.67945175	2.12E-26	-0.5675952	3.62E-17	-0.3442646	1.60E-06
1.95E-13	-0.4551733	7.55E-11	0.64865197	1.81E-23	0.2774117	0.00013187
0.01507775	0.3527301	8.46E-07	-0.3180188	1.03E-05	-0.6103898	2.87E-20
3.44E-25	-0.4313339	8.82E-10	0.70924953	1.35E-29	0.64386911	4.81E-23
1.25E-42	0.24584205	0.00074336	-0.6170367	8.57E-21	-0.3269758	5.55E-06
1.58E-24	-0.585515	2.07E-18	0.88869696	7.00E-64	0.78103148	2.87E-39
2.92E-41	0.54840317	6.45E-16	-0.8871305	2.33E-63	-0.5916618	7.44E-19
1.36E-27	-0.5802456	4.89E-18	0.68639467	4.14E-27	0.52883293	1.01E-14
1.94E-30	-0.3615011	4.29E-07	0.59939486	1.99E-19	0.20589997	0.00492724
1.10E-38	-0.5423855	1.53E-15	0.81841802	6.65E-46	0.30547732	2.35E-05
1.17E-50	-0.5551888	2.38E-16	0.81846244	6.52E-46	0.56755957	3.64E-17
3.43E-28	0.33478822	3.19E-06	-0.6340392	3.41E-22	-0.3673408	2.70E-07
1.47E-33	0.57467087	1.19E-17	-0.7145413	3.32E-30	-0.354684	7.29E-07
1.78E-49	0.37945175	1.00E-07	-0.6960608	3.93E-28	-0.4825287	3.52E-12
1.95E-05	-0.4532393	9.28E-11	0.6390857	1.26E-22	0.44132314	3.22E-10
7.93E-25	-0.3291797	4.76E-06	0.76925264	1.92E-37	0.28003674	0.0001131
2.45E-47	-0.5245668	1.80E-14	0.79059202	7.78E-41	0.31022146	1.73E-05
0.00021317	0.39344497	3.02E-08	-0.5689023	2.96E-17	-0.5880797	1.35E-18
1.96E-35	-0.4814046	4.01E-12	0.8760577	7.22E-60	0.54803556	6.81E-16
6.59E-25	-0.2354462	0.00125452	0.7570529	1.15E-35	0.63171161	5.36E-22
0.00297162	-0.4401221	3.64E-10	0.52742995	1.23E-14	0.4362897	5.37E-10
4.35E-11	-0.2224273	0.00234197	0.46169503	3.72E-11	0.3588965	5.26E-07
5.33E-33	-0.5372004	3.19E-15	0.88723595	2.15E-63	0.43037472	9.70E-10
2.34E-43	-0.4949289	8.00E-13	0.8089402	4.39E-44	0.66561768	4.86E-25

4.43E-27	0.17811812	0.01527986	-0.7126224	5.54E-30	-0.5052379	2.23E-13
4.06E-20	-0.3505952	9.96E-07	0.75233121	5.27E-35	0.25348827	0.00049865
2.64E-16	-0.3732779	1.67E-07	0.5410412	1.86E-15	0.09843321	0.1825313
2.32E-18	0.44067112	3.44E-10	-0.728674	6.62E-32	-0.5715528	1.95E-17
1.16E-23	0.38908536	4.41E-08	-0.7156024	2.49E-30	-0.6603978	1.52E-24
9.86E-22	0.47590345	7.58E-12	-0.829389	3.80E-48	-0.6605578	1.46E-24
2.48E-12	-0.4052202	1.06E-08	0.77497973	2.56E-38	0.77437646	3.18E-38
6.59E-24	-0.5669551	4.00E-17	0.84814668	2.23E-52	0.40160696	1.46E-08
1.08E-08	-0.3928398	3.18E-08	0.67634806	4.35E-26	0.54437664	1.15E-15
6.61E-28	-0.4744041	9.00E-12	0.86227533	5.78E-56	0.7587611	6.59E-36
0.00585135	0.26500273	0.00026699	-0.4846886	2.73E-12	-0.4932086	9.86E-13
2.21E-12	0.14972929	0.04192971	-0.6181118	7.03E-21	-0.3927677	3.20E-08
4.06E-31	-0.4831071	3.29E-12	0.71987209	7.79E-31	0.66015317	1.60E-24
1.64E-18	-0.5006703	3.95E-13	0.78223267	1.84E-39	0.43728498	4.86E-10
1.27E-20	0.42034238	2.57E-09	-0.5557948	2.18E-16	-0.0643631	0.38408067
1.65E-21	-0.558481	1.46E-16	0.86619134	4.98E-57	0.49428188	8.66E-13
2.12E-26	0.58320957	3.02E-18	-0.8644907	1.46E-56	-0.6091045	3.61E-20
9.39E-104	-0.5230816	2.20E-14	0.84735416	3.45E-52	0.5643488	5.98E-17
0.02091442	0.60096008	1.52E-19	-0.3768355	1.24E-07	-0.3892969	4.33E-08
6.68E-07	0.75096924	8.13E-35	-0.711489	7.49E-30	-0.3386897	2.41E-06
4.85E-37	-0.1727381	0.01870944	0.64421948	4.48E-23	0.34425788	1.60E-06
2.81E-15	-0.5526853	3.45E-16	0.85359991	1.02E-53	0.46890205	1.68E-11
1.97E-34	0.49618626	6.86E-13	-0.8212162	1.84E-46	-0.6329257	4.23E-22
3.21E-23	0.6985726	2.10E-28	-0.7371439	5.62E-33	-0.4824449	3.55E-12
2.05E-40	0.37698892	1.23E-07	-0.5902626	9.41E-19	-0.3627583	3.88E-07
1.14E-06	-0.4222632	2.14E-09	0.69838154	2.20E-28	0.65816481	2.45E-24
5.39E-38	-0.4437655	2.51E-10	0.91916167	5.73E-76	0.56520863	5.24E-17
7.43E-07	-0.280588	0.00010949	0.71333356	4.58E-30	0.47379326	9.65E-12
6.25E-29	-0.587659	1.45E-18	0.86552994	7.58E-57	0.80442047	2.98E-43
2.23E-12	0.55228066	3.66E-16	-0.546774	8.17E-16	-0.5732019	1.51E-17
8.17E-29	-0.5620171	8.53E-17	0.82981377	3.09E-48	0.85308542	1.37E-53
2.12E-15	-0.3892197	4.36E-08	0.74036811	2.14E-33	0.3482911	1.18E-06
3.02E-32	-0.3608009	4.53E-07	0.80247406	6.70E-43	0.53606212	3.74E-15
1.26E-38	0.71971364	8.14E-31	-0.7838543	1.01E-39	-0.7138996	3.94E-30
0.00254965	-0.4162703	3.79E-09	0.61534967	1.17E-20	0.38741303	5.10E-08
1.75E-11	-0.4753875	8.04E-12	0.77428792	3.28E-38	0.50753392	1.67E-13
1.36E-50	0.43045845	9.62E-10	-0.8251454	2.93E-47	-0.4717473	1.22E-11
1.11E-16	0.51005735	1.21E-13	-0.8611692	1.14E-55	-0.599475	1.96E-19
1.53E-09	-0.3126514	1.47E-05	0.5193622	3.60E-14	0.66622537	4.25E-25
7.64E-62	-0.3740422	1.57E-07	0.75561172	1.84E-35	0.40749025	8.58E-09
1.28E-16	0.59448276	4.62E-19	-0.6926609	9.08E-28	-0.6489132	1.71E-23
2.68E-37	0.27908855	0.00011957	-0.6322672	4.81E-22	-0.4384007	4.34E-10
2.35E-21	-0.5081064	1.55E-13	0.8635722	2.59E-56	0.64484503	3.95E-23
8.80E-34	-0.5351229	4.26E-15	0.9395099	4.41E-87	0.67587478	4.85E-26
3.29E-25	0.29539275	4.46E-05	-0.7294716	5.27E-32	-0.6010762	1.49E-19
4.75E-36	-0.6292386	8.64E-22	0.93301174	3.71E-83	0.66201234	1.07E-24

9.83E-13	-0.4496056	1.36E-10	0.75332277	3.84E-35	0.33660701	2.80E-06
1.17E-08	-0.4687443	1.71E-11	0.75072754	8.77E-35	0.65461541	5.21E-24
9.68E-42	-0.5717884	1.88E-17	0.86000486	2.32E-55	0.62387024	2.40E-21
1.84E-22	-0.5710756	2.11E-17	0.84085851	1.14E-50	0.66745348	3.24E-25
0.00058113	-0.3663769	2.91E-07	0.4083839	7.90E-09	0.72220636	4.09E-31
2.79E-38	0.42421792	1.77E-09	-0.8619233	7.18E-56	-0.5131541	8.11E-14
7.67E-11	-0.4379344	4.55E-10	0.71571476	2.42E-30	0.44023819	3.60E-10
4.90E-26	-0.5265267	1.38E-14	0.89179184	6.14E-65	0.58586552	1.95E-18
3.96E-13	0.32332131	7.16E-06	-0.6028031	1.10E-19	-0.689357	2.03E-27
0.02525018	-0.4112471	6.06E-09	0.39430711	2.80E-08	0.55057231	4.70E-16
4.32E-13	0.15809954	0.03160685	-0.5359851	3.78E-15	-0.656947	3.18E-24
1.73E-26	0.41305214	5.13E-09	-0.7019971	8.82E-29	-0.6207796	4.28E-21
1.27E-19	-0.5674711	3.69E-17	0.86668201	3.65E-57	0.76037426	3.87E-36
3.04E-17	0.1452418	0.04854288	-0.4659827	2.32E-11	0.0446415	0.5462589
7.98E-33	-0.5518396	3.91E-16	0.92473627	1.07E-78	0.76349572	1.37E-36
1.57E-17	-0.5199302	3.34E-14	0.8304821	2.23E-48	0.57601703	9.64E-18
2.11E-11	0.24766654	0.00067656	-0.5074189	1.69E-13	-0.4317542	8.46E-10
2.55E-11	-0.5569151	1.84E-16	0.80870659	4.85E-44	0.51689548	4.99E-14
2.25E-11	-0.4722108	1.15E-11	0.74737667	2.51E-34	0.75258334	4.87E-35
3.71E-07	-0.3430936	1.75E-06	0.4995722	4.52E-13	0.74745479	2.45E-34
4.12E-14	-0.4324926	7.86E-10	0.82694845	1.24E-47	0.65327612	6.90E-24
1.11E-32	-0.5284376	1.07E-14	0.76289023	1.67E-36	0.6692188	2.19E-25
0.00011012	-0.2691357	0.00021188	0.55733708	1.73E-16	0.05776121	0.43482323
2.55E-28	-0.4825522	3.51E-12	0.9137156	1.73E-73	0.48327093	3.23E-12
1.53E-09	-0.4711955	1.30E-11	0.59824094	2.43E-19	0.22970476	0.00165909
5.37E-24	0.40273322	1.32E-08	-0.6655758	4.90E-25	-0.2139821	0.00344792
4.38E-08	-0.2444016	0.00080033	0.69398604	6.56E-28	0.50061921	3.97E-13
2.76E-20	-0.4441043	2.42E-10	0.7151621	2.81E-30	0.380114	9.46E-08
0.00069645	0.35997393	4.84E-07	-0.5861404	1.87E-18	-0.3347329	3.21E-06
2.23E-08	0.36741309	2.68E-07	-0.6273004	1.25E-21	-0.670908	1.50E-25
0.0731421	0.35917491	5.15E-07	-0.516586	5.19E-14	-0.5178136	4.42E-14
1.52E-24	-0.4931426	9.94E-13	0.84613914	6.72E-52	0.68031295	1.74E-26
1.16E-26	0.29977892	3.39E-05	-0.7181604	1.25E-30	-0.3350205	3.14E-06
1.20E-42	0.44714181	1.76E-10	-0.7803226	3.72E-39	-0.3635925	3.64E-07
8.84E-36	-0.3230001	7.32E-06	0.74159902	1.48E-33	0.22237617	0.00234755
1.91E-53	0.29360177	4.99E-05	-0.6924763	9.50E-28	-0.6153892	1.16E-20
4.44E-47	-0.5758176	9.96E-18	0.96215686	2.87E-105	0.62027399	4.71E-21
1.87E-31	-0.5215886	2.68E-14	0.90242257	7.87E-69	0.637841	1.61E-22
4.26E-26	-0.3582788	5.52E-07	0.48642689	2.22E-12	0.06949891	0.34720853
9.07E-33	0.24131283	0.00093629	-0.7331525	1.82E-32	-0.4355679	5.78E-10
4.24E-07	0.26037236	0.00034441	-0.4333111	7.24E-10	0.19431005	0.00804207
5.95E-22	0.57221058	1.76E-17	-0.6818334	1.22E-26	-0.7112685	7.94E-30
0.00070883	-0.3410367	2.03E-06	0.61883641	6.15E-21	0.51940011	3.59E-14
1.83E-22	0.36568444	3.08E-07	-0.7559164	1.67E-35	-0.3134893	1.39E-05
3.41E-38	0.39019426	4.01E-08	-0.7842617	8.65E-40	-0.6031517	1.04E-19
1.36E-33	0.43314312	7.36E-10	-0.8453102	1.06E-51	-0.7353471	9.56E-33

4.46E-46	-0.5978863	2.58E-19	0.90289568	5.16E-69	0.59468961	4.46E-19
3.65E-18	0.53785326	2.91E-15	-0.8798754	4.94E-61	-0.4973564	5.94E-13
1.21E-48	-0.3529905	8.30E-07	0.56838663	3.21E-17	0.25133351	0.00055873
2.18E-17	0.35453044	7.37E-07	-0.5181729	4.22E-14	-0.5757951	9.99E-18
0.76094461	0.16106696	0.02850833	-0.3115664	1.58E-05	-0.4304781	9.60E-10
4.61E-20	-0.323543	7.05E-06	0.5414359	1.76E-15	0.49320675	9.86E-13
9.17E-07	0.46078673	4.11E-11	-0.534106	4.91E-15	-0.4352262	5.98E-10
5.07E-46	0.29436921	4.76E-05	-0.6940351	6.48E-28	-0.5843432	2.51E-18
2.08E-21	0.35883804	5.28E-07	-0.8027275	6.03E-43	-0.6116305	2.29E-20
3.03E-26	0.3964618	2.32E-08	-0.7740199	3.61E-38	-0.7637658	1.25E-36
4.62E-15	-0.0410877	0.57868977	0.46108581	3.98E-11	0.60085043	1.55E-19
2.20E-80	-0.5447961	1.09E-15	0.93659128	2.88E-85	0.57692137	8.35E-18
4.26E-16	0.26193967	0.00031613	-0.7337726	1.52E-32	-0.4410049	3.33E-10
5.83E-24	-0.2580134	0.00039142	0.66370858	7.38E-25	0.13397182	0.06905379
1.23E-17	0.56349136	6.81E-17	-0.6121191	2.10E-20	-0.6204507	4.55E-21
8.99E-77	-0.5670268	3.96E-17	0.90743108	8.02E-71	0.63827812	1.48E-22
2.59E-49	-0.5730083	1.55E-17	0.86471562	1.27E-56	0.42127464	2.35E-09
6.33E-34	0.54071997	1.94E-15	-0.8401438	1.66E-50	-0.6336435	3.68E-22
7.63E-06	0.16009262	0.02949579	-0.3234113	7.11E-06	-0.3132107	1.42E-05
2.92E-15	0.29621618	4.24E-05	-0.4341323	6.67E-10	-0.4764638	7.11E-12
0.00114808	0.54802023	6.82E-16	-0.451992	1.06E-10	-0.61542	1.15E-20
4.82E-28	-0.3216837	8.01E-06	0.72667545	1.17E-31	0.28272287	9.65E-05
7.60E-07	-0.3772442	1.20E-07	0.67596853	4.75E-26	0.36652821	2.88E-07
2.31E-37	-0.5445159	1.13E-15	0.89192761	5.51E-65	0.50923253	1.34E-13
0.00034828	-0.1006219	0.17294849	0.21628901	0.00310656	-0.1598193	0.02977797
0.00955374	-0.3547895	7.23E-07	0.56957531	2.66E-17	0.5829648	3.14E-18
2.52E-24	-0.5784927	6.49E-18	0.88043173	3.32E-61	0.44794422	1.62E-10
3.08E-51	0.45251884	1.00E-10	-0.9148672	5.34E-74	-0.665396	5.10E-25
4.18E-19	0.38783067	4.92E-08	-0.7557645	1.75E-35	-0.1738689	0.01793778
8.57E-23	0.43368658	6.98E-10	-0.7922647	4.06E-41	-0.7024355	7.88E-29
2.56E-17	-0.5234009	2.11E-14	0.85060276	5.64E-53	0.47952125	5.00E-12
9.24E-30	0.3615046	4.29E-07	-0.8654612	7.92E-57	-0.6364947	2.10E-22
9.49E-40	0.68894454	2.24E-27	-0.7090401	1.43E-29	-0.4475284	1.69E-10
1.12E-31	-0.5037134	2.70E-13	0.86128528	1.06E-55	0.87507346	1.42E-59
2.40E-12	0.3332558	3.57E-06	-0.7272369	9.96E-32	-0.2207118	0.00253629
2.28E-24	-0.4357726	5.66E-10	0.80890423	4.46E-44	0.32783772	5.23E-06
2.06E-14	-0.3979063	2.04E-08	0.66821209	2.74E-25	0.26537224	0.00026156
1.30E-43	-0.4816741	3.89E-12	0.84693084	4.35E-52	0.58831593	1.30E-18
5.24E-12	0.46668623	2.15E-11	-0.7364022	7.00E-33	-0.4793528	5.10E-12
1.38E-16	0.36594519	3.02E-07	-0.6602553	1.56E-24	-0.0604375	0.41380602
5.36E-32	0.43053668	9.54E-10	-0.8069615	1.02E-43	-0.5756219	1.03E-17
1.62E-12	0.17173629	0.01941699	-0.525058	1.69E-14	-0.2472095	0.00069275
3.69E-10	0.23324502	0.00139753	-0.4644747	2.74E-11	-0.3238655	6.89E-06
1.99E-07	0.30122825	3.09E-05	-0.5276741	1.19E-14	-0.624999	1.94E-21
1.55E-19	0.36544015	3.14E-07	-0.663052	8.52E-25	-0.7144503	3.40E-30
6.20E-16	-0.4751568	8.26E-12	0.78483096	6.99E-40	0.43527949	5.95E-10

1.44E-44	0.51265089	8.66E-14	-0.7653451	7.31E-37	-0.5461479	8.95E-16
2.57E-35	0.25897543	0.00037157	-0.6854483	5.19E-27	-0.4487607	1.49E-10
5.36E-12	-0.5497846	5.28E-16	0.80493769	2.40E-43	0.6317841	5.29E-22
0.87546808	0.25777983	0.00039638	-0.2793789	0.00011755	-0.1285863	0.08109684
7.21E-27	0.45189334	1.07E-10	-0.7547668	2.42E-35	-0.7014964	1.00E-28
3.02E-36	0.46068547	4.16E-11	-0.750522	9.36E-35	-0.5422187	1.57E-15
5.42E-11	-0.4596036	4.68E-11	0.7417093	1.43E-33	0.34295863	1.76E-06
5.05E-19	-0.25113	0.00056473	0.68163767	1.28E-26	0.2835687	9.18E-05
5.94E-34	0.27111242	0.00018945	-0.6745522	6.56E-26	-0.5647769	5.60E-17
0.02177845	0.30583179	2.30E-05	-0.5666067	4.22E-17	-0.3181555	1.02E-05
0.00011798	0.31873055	9.79E-06	-0.6323905	4.70E-22	-0.4395311	3.87E-10
2.24E-14	0.48541643	2.51E-12	-0.7211325	5.51E-31	-0.6090201	3.67E-20
9.72E-31	-0.3597151	4.93E-07	0.78702043	3.06E-40	0.54476271	1.09E-15
3.25E-24	0.37920603	1.02E-07	-0.7288922	6.22E-32	-0.4957381	7.25E-13
3.74E-28	0.53336023	5.44E-15	-0.8600637	2.23E-55	-0.5503796	4.84E-16
0.0031611	0.41699421	3.54E-09	-0.4801393	4.65E-12	-0.4593276	4.82E-11
7.29E-21	-0.45848	5.28E-11	0.68057606	1.64E-26	0.53431042	4.77E-15
1.87E-15	-0.4812024	4.11E-12	0.74405619	6.99E-34	0.15617984	0.03376036
5.05E-10	0.32345996	7.09E-06	-0.5571148	1.79E-16	-0.4064212	9.46E-09
1.91E-11	-0.5848966	2.29E-18	0.50487212	2.33E-13	0.41963056	2.75E-09
6.05E-11	-0.2759613	0.00014345	0.57411797	1.30E-17	0.10723945	0.14624563
4.69E-23	-0.4172689	3.45E-09	0.67560722	5.16E-26	0.7528849	4.42E-35
0.03557044	0.4035669	1.23E-08	-0.5454967	9.82E-16	-0.4340385	6.73E-10
2.25E-29	-0.4979145	5.55E-13	0.7347585	1.14E-32	0.30286738	2.79E-05
1.02E-08	0.3838725	6.89E-08	-0.5424384	1.52E-15	-0.5518836	3.88E-16
3.82E-16	-0.3214804	8.12E-06	0.74273232	1.05E-33	0.24700442	0.00070013
1.36E-08	0.41766645	3.32E-09	-0.6759416	4.78E-26	-0.504615	2.41E-13
1.08E-33	-0.476422	7.15E-12	0.85725446	1.20E-54	0.58242044	3.44E-18
1.68E-11	-0.3865689	5.48E-08	0.72430241	2.28E-31	0.74642656	3.37E-34
2.85E-06	-0.20536	0.0050439	0.48137344	4.03E-12	0.17504083	0.01716733
3.05E-76	-0.4734104	1.01E-11	0.85945716	3.22E-55	0.5063188	1.94E-13
1.75E-09	0.286287	7.80E-05	-0.6325672	4.54E-22	-0.559258	1.30E-16
0.00665643	-0.380995	8.79E-08	0.55037167	4.84E-16	0.58020982	4.92E-18
1.24E-17	-0.4592357	4.87E-11	0.70427203	4.93E-29	0.37482939	1.47E-07
3.71E-15	-0.3768146	1.25E-07	0.65164686	9.71E-24	0.37788422	1.14E-07
3.00E-11	-0.5239332	1.96E-14	0.7532636	3.92E-35	0.64231806	6.58E-23
6.86E-23	0.41607001	3.86E-09	-0.7952995	1.23E-41	-0.5563752	2.00E-16
1.54E-29	-0.4655936	2.42E-11	0.82769477	8.64E-48	0.32580827	6.02E-06
1.84E-13	0.34716243	1.29E-06	-0.7189483	1.00E-30	-0.4281814	1.20E-09
3.96E-26	-0.330114	4.46E-06	0.52209895	2.51E-14	0.11191645	0.12934757
1.30E-08	0.24971888	0.00060806	-0.4197592	2.72E-09	-0.415275	4.16E-09
6.25E-07	0.24294808	0.00086187	-0.4116792	5.82E-09	-0.452829	9.69E-11
0.36338012	-0.3336411	3.47E-06	0.4150484	4.25E-09	0.10741664	0.14557633
1.58E-19	0.37978408	9.73E-08	-0.7251302	1.81E-31	-0.5044306	2.47E-13
1.97E-09	-0.4287049	1.14E-09	0.77356078	4.24E-38	0.62988815	7.63E-22
1.24E-24	-0.2569201	0.00041516	0.54982403	5.25E-16	0.66548772	5.00E-25

2.22E-12	-0.2486342	0.00064342	0.48229707	3.62E-12	0.12844968	0.08142299
4.39E-33	-0.4113761	5.99E-09	0.89283705	2.65E-65	0.42594744	1.50E-09
3.52E-11	-0.1863027	0.01111428	0.33144731	4.05E-06	-0.0968067	0.18990154
1.29E-16	-0.318268	1.01E-05	0.62724599	1.27E-21	0.04115361	0.57808001
1.65E-28	-0.5544116	2.67E-16	0.91654958	9.31E-75	0.53368121	5.20E-15
1.42E-10	-0.2982167	3.74E-05	0.68300675	9.25E-27	0.67916378	2.27E-26
3.77E-06	0.55796525	1.57E-16	-0.6748357	6.15E-26	-0.3682642	2.50E-07
1.24E-47	-0.5815486	3.96E-18	0.97380102	1.19E-119	0.74182676	1.38E-33
2.33E-29	-0.3502882	1.02E-06	0.79803523	4.11E-42	0.27103986	0.00019023
5.84E-13	-0.5366745	3.43E-15	0.71682044	1.79E-30	0.7325619	2.16E-32
0.00137076	-0.4336095	7.03E-10	0.41141921	5.97E-09	0.56759163	3.63E-17
4.27E-15	0.4104381	6.54E-09	-0.6353185	2.65E-22	-0.4051342	1.06E-08
1.51E-20	0.23078573	0.00157485	-0.7130242	4.98E-30	-0.4374551	4.78E-10
1.96E-48	-0.6379913	1.56E-22	0.79651455	7.56E-42	0.42844899	1.17E-09
3.62E-35	0.50217592	3.27E-13	-0.794951	1.41E-41	-0.4526574	9.87E-11
7.71E-23	-0.6150137	1.24E-20	0.89558256	2.81E-66	0.56131625	9.49E-17
1.53E-31	0.22237138	0.00234808	-0.5988032	2.20E-19	-0.2912902	5.75E-05
4.83E-38	-0.2853247	8.26E-05	0.61171262	2.26E-20	0.14034378	0.05673009
1.17E-54	-0.4949887	7.94E-13	0.82555832	2.40E-47	0.37055719	2.08E-07
1.11E-19	-0.4744567	8.95E-12	0.7487019	1.66E-34	0.19440905	0.00800936
1.99E-25	-0.1221766	0.09757393	0.64245272	6.41E-23	0.22858713	0.00175051
7.72E-08	-0.334951	3.16E-06	0.49418954	8.75E-13	0.05201998	0.48191312
1.75E-11	-0.3587196	5.33E-07	0.72834473	7.27E-32	0.24082436	0.00095964
1.86E-47	-0.4999067	4.34E-13	0.80251416	6.59E-43	0.30848911	1.94E-05
1.70E-53	-0.5996051	1.92E-19	0.9392878	6.11E-87	0.70094728	1.15E-28
6.92E-16	-0.5519954	3.82E-16	0.79043496	8.26E-41	0.76492548	8.43E-37
1.16E-06	0.21114303	0.00391428	-0.4913702	1.23E-12	-0.4340106	6.75E-10
1.31E-26	-0.4492262	1.42E-10	0.67809518	2.91E-26	0.18648425	0.01103452
2.88E-05	-0.3225073	7.57E-06	0.60679819	5.44E-20	0.39593535	2.43E-08
0.00022842	-0.3050133	2.43E-05	0.62450799	2.13E-21	0.5350298	4.32E-15
6.43E-43	-0.5430987	1.39E-15	0.93897907	9.58E-87	0.5579438	1.58E-16
1.52E-13	0.27566807	0.00014591	-0.6458835	3.19E-23	-0.5000323	4.27E-13
1.11E-26	-0.3006928	3.20E-05	0.69891908	1.92E-28	0.250421	0.00058613
8.88E-33	-0.5934285	5.52E-19	0.90395553	1.99E-69	0.58740272	1.51E-18
0.00289383	0.3310421	4.17E-06	-0.5966857	3.17E-19	-0.4417689	3.08E-10
0.09304018	0.07713713	0.29665984	-0.2322833	0.00146458	-0.1435582	0.05123843
4.94E-08	0.2810551	0.00010651	-0.4987184	5.03E-13	-0.6047024	7.88E-20
2.14E-23	0.42464426	1.70E-09	-0.7360439	7.78E-33	-0.5706564	2.25E-17
7.02E-22	-0.5225929	2.35E-14	0.73548859	9.17E-33	0.86751861	2.13E-57
9.27E-46	-0.5337357	5.17E-15	0.8458952	7.67E-52	0.45172777	1.09E-10
0.00128443	0.51686057	5.01E-14	-0.47033	1.43E-11	-0.5073045	1.72E-13
1.15E-30	0.43839182	4.34E-10	-0.7036341	5.80E-29	-0.5127112	8.59E-14
1.40E-13	0.22947986	0.00167713	-0.4925411	1.07E-12	-0.6342861	3.25E-22
0.00014453	0.23110344	0.00155084	-0.3506458	9.92E-07	-0.4867182	2.15E-12
2.44E-09	0.61062357	2.75E-20	-0.756271	1.49E-35	-0.4281468	1.21E-09
1.35E-85	-0.4938505	9.12E-13	0.83767047	6.00E-50	0.51943511	3.57E-14

2.07E-29	-0.4177187	3.30E-09	0.74203366	1.30E-33	0.21528982	0.00325047
3.12E-05	0.26272936	0.00030271	-0.5690371	2.90E-17	-0.1547604	0.03543153
6.55E-47	-0.5198652	3.37E-14	0.79871817	3.12E-42	0.32789425	5.21E-06
6.25E-09	0.2785055	0.00012372	-0.7027208	7.33E-29	-0.3292647	4.73E-06
1.53E-26	0.51465731	6.68E-14	-0.5036145	2.73E-13	-0.3313134	4.09E-06
1.39E-20	0.35531397	6.94E-07	-0.7529829	4.28E-35	-0.7266005	1.19E-31
3.87E-05	-0.5120151	9.40E-14	0.49452403	8.41E-13	0.40349375	1.23E-08
2.85E-33	-0.4183438	3.11E-09	0.67944192	2.13E-26	0.31086872	1.66E-05
4.86E-31	0.26848655	0.00021977	-0.5732655	1.49E-17	-0.5004497	4.06E-13
1.75E-29	-0.2747159	0.00015415	0.59358928	5.37E-19	0.5976387	2.69E-19
2.03E-32	0.58016083	4.96E-18	-0.780092	4.05E-39	-0.7766773	1.40E-38
8.56E-67	-0.5099293	1.23E-13	0.80789126	6.88E-44	0.49587543	7.13E-13
1.58E-13	0.24759467	0.00067908	-0.4988173	4.97E-13	-0.4913149	1.24E-12
1.83E-14	0.54673404	8.22E-16	-0.7253448	1.70E-31	-0.580024	5.07E-18
1.71E-36	-0.528846	1.01E-14	0.94954746	4.32E-94	0.68262201	1.01E-26
6.80E-22	0.49099144	1.29E-12	-0.7670739	4.05E-37	-0.6523215	8.43E-24
3.89E-53	-0.4810373	4.19E-12	0.82831958	6.39E-48	0.75436866	2.75E-35
1.65E-49	-0.3950499	2.62E-08	0.83041576	2.30E-48	0.34433651	1.59E-06
5.82E-47	-0.4751741	8.24E-12	0.88069972	2.74E-61	0.46564286	2.41E-11
1.19E-15	-0.5509382	4.46E-16	0.69088033	1.40E-27	0.18931058	0.0098563
6.86E-33	0.56161485	9.07E-17	-0.9157711	2.10E-74	-0.7196232	8.34E-31
5.02E-09	0.39298069	3.15E-08	-0.5851674	2.19E-18	-0.596637	3.20E-19
1.10E-36	-0.5988662	2.18E-19	0.89867451	2.08E-67	0.72531941	1.71E-31
8.34E-45	-0.5985319	2.31E-19	0.91948594	4.03E-76	0.60145443	1.39E-19
7.19E-29	0.58806232	1.36E-18	-0.7459418	3.91E-34	-0.7933461	2.66E-41
2.47E-07	0.61147395	2.36E-20	-0.5734081	1.46E-17	-0.4279232	1.23E-09
2.45E-15	0.37214645	1.83E-07	-0.647283	2.40E-23	-0.633323	3.92E-22
4.52E-52	0.44876525	1.49E-10	-0.7331122	1.84E-32	-0.3621087	4.09E-07
3.48E-14	0.44036819	3.55E-10	-0.7716839	8.21E-38	-0.5304402	8.13E-15
1.85E-30	0.33696884	2.73E-06	-0.5929154	6.02E-19	-0.2299755	0.00163762
3.37E-45	0.40401541	1.18E-08	-0.6980194	2.41E-28	-0.4743301	9.08E-12
7.44E-09	-0.2230607	0.0022737	0.63516377	2.73E-22	0.36237146	4.01E-07
1.05E-39	-0.4787909	5.44E-12	0.76011977	4.21E-36	0.30996824	1.76E-05
6.63E-16	0.56488282	5.51E-17	-0.6169914	8.64E-21	-0.4515626	1.11E-10
7.98E-39	-0.4910156	1.29E-12	0.76023574	4.05E-36	0.30967071	1.79E-05
2.49E-11	-0.3948087	2.68E-08	0.71514384	2.82E-30	0.4599214	4.52E-11
1.49E-15	0.51246607	8.87E-14	-0.6781962	2.84E-26	-0.5463908	8.64E-16
0.23276976	0.20562649	0.00498602	-0.3767711	1.25E-07	0.08074133	0.27459484
1.23E-12	0.37269023	1.75E-07	-0.7255987	1.58E-31	-0.5833539	2.95E-18
3.60E-06	0.66450951	6.20E-25	-0.5562817	2.02E-16	-0.1398007	0.05770432
3.44E-17	0.61285539	1.84E-20	-0.6726032	1.02E-25	-0.2769522	0.00013544
3.17E-14	-0.433767	6.92E-10	0.74667376	3.12E-34	0.288636	6.77E-05
0.07995417	-0.3429811	1.76E-06	0.44216715	2.95E-10	0.51442541	6.88E-14
4.07E-46	0.52589264	1.51E-14	-0.9190825	6.24E-76	-0.7267052	1.16E-31
4.38E-07	-0.3885915	4.60E-08	0.70873959	1.55E-29	0.24316762	0.00085231
2.12E-25	-0.249569	0.00061283	0.46679578	2.12E-11	0.06145626	0.40596471

5.77E-33	0.54592503	9.24E-16	-0.8307727	1.93E-48	-0.6867538	3.80E-27
1.98E-23	0.29880301	3.61E-05	-0.553178	3.21E-16	-0.4894173	1.56E-12
4.80E-25	0.37570218	1.37E-07	-0.6482169	1.98E-23	-0.496413	6.67E-13
4.83E-26	0.35979411	4.90E-07	-0.6534484	6.66E-24	-0.4957953	7.20E-13
1.77E-29	-0.5880333	1.36E-18	0.91731197	4.16E-75	0.55207394	3.77E-16
0.41678834	0.45709128	6.14E-11	-0.5168097	5.04E-14	-0.3371777	2.69E-06
4.77E-52	-0.5489861	5.93E-16	0.87750087	2.65E-60	0.5647097	5.65E-17
4.17E-31	0.39040288	3.94E-08	-0.6092687	3.51E-20	-0.178403	0.01511465
6.78E-27	-0.5046176	2.41E-13	0.81745895	1.03E-45	0.33447026	3.27E-06
1.99E-07	-0.5011463	3.72E-13	0.61785688	7.37E-21	0.401844	1.43E-08
4.83E-14	-0.5248102	1.74E-14	0.79680368	6.74E-42	0.55460719	2.60E-16
1.61E-16	-0.0173411	0.81476545	-0.4818773	3.80E-12	-0.3835578	7.08E-08
0.00012807	-0.4139082	4.73E-09	0.68276089	9.80E-27	0.47238384	1.13E-11
6.56E-40	0.34171884	1.93E-06	-0.8252017	2.85E-47	-0.6165329	9.40E-21
2.09E-06	-0.428169	1.20E-09	0.717501	1.49E-30	0.46425421	2.81E-11
8.24E-21	-0.5571385	1.78E-16	0.83931247	2.56E-50	0.63621646	2.22E-22
2.17E-24	-0.4309151	9.19E-10	0.79918414	2.58E-42	0.70664207	2.67E-29
2.16E-11	-0.4064546	9.43E-09	0.75916565	5.77E-36	0.59477229	4.40E-19
1.18E-10	-0.5665918	4.23E-17	0.72160119	4.84E-31	0.46901308	1.66E-11
1.01E-36	0.45366031	8.87E-11	-0.7951065	1.33E-41	-0.7087437	1.54E-29
1.23E-26	0.80545701	1.93E-43	-0.7429489	9.81E-34	-0.6713219	1.36E-25
6.82E-39	-0.5332852	5.50E-15	0.93534327	1.62E-84	0.77319605	4.83E-38
5.81E-14	-0.4533668	9.15E-11	0.80042438	1.56E-42	0.43548353	5.83E-10
7.35E-21	-0.5742806	1.27E-17	0.87037121	3.34E-58	0.6607249	1.41E-24
1.97E-16	0.30363408	2.65E-05	-0.6553642	4.44E-24	-0.6847389	6.14E-27
4.36E-42	0.4544391	8.16E-11	-0.870993	2.21E-58	-0.667046	3.54E-25
4.34E-34	-0.5927895	6.15E-19	0.9471182	2.85E-92	0.61271045	1.89E-20
1.39E-23	-0.4891263	1.61E-12	0.75870287	6.71E-36	0.39648962	2.31E-08
2.37E-31	-0.4607112	4.15E-11	0.92895205	6.69E-81	0.60694435	5.30E-20
5.99E-29	0.27534256	0.00014868	-0.7595839	5.02E-36	-0.3893108	4.33E-08
3.32E-22	-0.446253	1.94E-10	0.68716421	3.44E-27	0.32222182	7.72E-06
9.88E-42	-0.5780684	6.95E-18	0.79934645	2.42E-42	0.52600673	1.49E-14
8.64E-18	0.72663978	1.18E-31	-0.5332082	5.56E-15	-0.366715	2.84E-07
5.01E-34	0.4359109	5.58E-10	-0.7768592	1.31E-38	-0.7517332	6.38E-35
3.26E-31	0.40379787	1.20E-08	-0.6746667	6.39E-26	-0.5651696	5.27E-17
8.97E-13	-0.4468337	1.82E-10	0.76246368	1.93E-36	0.48295998	3.35E-12
1.91E-15	0.22122307	0.0024769	-0.5703005	2.38E-17	-0.684207	6.96E-27
2.60E-32	-0.4568952	6.27E-11	0.8011343	1.16E-42	0.82538072	2.62E-47
3.06E-45	-0.5336253	5.24E-15	0.95629709	1.15E-99	0.78928418	1.29E-40
8.50E-27	-0.4241313	1.79E-09	0.85184499	2.79E-53	0.38099278	8.79E-08
1.58E-38	-0.4111106	6.14E-09	0.78878767	1.56E-40	0.34942262	1.09E-06
4.27E-23	-0.4490159	1.45E-10	0.61376029	1.56E-20	0.34223707	1.86E-06
1.96E-17	0.38841734	4.67E-08	-0.7431906	9.11E-34	-0.5700211	2.48E-17
2.25E-12	-0.3710585	2.00E-07	0.64145068	7.84E-23	0.55952478	1.24E-16
6.35E-34	0.18504662	0.01168015	-0.6113023	2.43E-20	-0.306921	2.14E-05
8.41E-24	-0.4791882	5.19E-12	0.73355159	1.62E-32	0.16261618	0.02699642

1.49E-17	-0.4098542	6.90E-09	0.77758732	1.01E-38	0.5324775	6.15E-15
2.80E-48	0.35851962	5.42E-07	-0.6913494	1.25E-27	-0.5294709	9.28E-15
7.14E-09	0.33159968	4.01E-06	-0.719002	9.89E-31	-0.1807498	0.0138121
0.03426096	-0.423464	1.91E-09	0.53927427	2.38E-15	0.51442315	6.88E-14
2.56E-60	-0.5836209	2.82E-18	0.93437351	6.03E-84	0.63990498	1.07E-22
2.14E-41	-0.4859747	2.35E-12	0.63243702	4.66E-22	0.28800577	7.03E-05
5.85E-17	-0.4774648	6.34E-12	0.75042398	9.65E-35	0.52899454	9.90E-15
9.70E-17	0.03022638	0.68295799	0.67886862	2.43E-26	0.33072803	4.27E-06
9.43E-32	0.38228999	7.88E-08	-0.848838	1.52E-52	-0.7862138	4.15E-40
8.70E-18	0.27035079	0.00019781	-0.7056383	3.46E-29	-0.1605105	0.02906879
2.88E-34	-0.3940912	2.85E-08	0.74536988	4.67E-34	0.26546968	0.00026015
1.79E-16	0.39771886	2.07E-08	-0.6984343	2.17E-28	-0.5829756	3.14E-18
2.10E-24	0.52193421	2.56E-14	-0.6936244	7.17E-28	-0.5575492	1.67E-16
4.82E-29	-0.4866187	2.17E-12	0.81806459	7.81E-46	0.56990706	2.53E-17
6.06E-22	-0.4728069	1.08E-11	0.84453628	1.60E-51	0.78405315	9.35E-40
1.53E-13	-0.4184474	3.08E-09	0.79773354	4.64E-42	0.70400435	5.28E-29
1.04E-12	-0.5401756	2.10E-15	0.60986209	3.15E-20	0.10553808	0.15279176
2.01E-48	-0.3990152	1.85E-08	0.68969332	1.87E-27	0.28863153	6.77E-05
2.25E-09	-0.4847382	2.72E-12	0.67141165	1.34E-25	0.34081842	2.06E-06
8.64E-47	-0.5048803	2.33E-13	0.78619667	4.18E-40	0.51278158	8.51E-14
1.40E-64	-0.5753265	1.08E-17	0.96307685	3.15E-106	0.67553004	5.25E-26
4.63E-25	-0.53304	5.69E-15	0.88556062	7.67E-63	0.57774153	7.32E-18
3.81E-12	-0.4947371	8.19E-13	0.8298607	3.02E-48	0.54394109	1.23E-15
8.15E-24	0.62024036	4.74E-21	-0.607325	4.96E-20	-0.4731217	1.04E-11
1.23E-12	0.30682006	2.16E-05	-0.6876978	3.03E-27	-0.5429871	1.41E-15
2.36E-45	0.55822516	1.51E-16	-0.6817303	1.25E-26	-0.56638	4.37E-17
4.74E-22	-0.3138945	1.36E-05	0.83543396	1.88E-49	0.49206319	1.13E-12
5.70E-13	0.73127656	3.13E-32	-0.5518072	3.92E-16	-0.1831465	0.01258432
1.14E-09	0.30983793	1.77E-05	-0.6705343	1.63E-25	-0.6678898	2.94E-25
4.93E-18	0.34050601	2.11E-06	-0.733787	1.51E-32	-0.7697932	1.59E-37
2.29E-16	0.44412633	2.41E-10	-0.7619902	2.26E-36	-0.6650487	5.50E-25
9.16E-12	0.28634937	7.77E-05	-0.7711779	9.80E-38	-0.4534608	9.06E-11
8.16E-07	0.22704585	0.00188411	-0.5877636	1.43E-18	-0.6464248	2.86E-23
5.17E-35	-0.4641679	2.84E-11	0.82325109	7.15E-47	0.38091788	8.85E-08
1.25E-42	-0.6115843	2.31E-20	0.89235527	3.91E-65	0.57136953	2.01E-17
0.03980819	-0.2120851	0.00375359	0.20256847	0.00568735	0.37790113	1.14E-07
1.01E-08	-0.4933496	9.69E-13	0.73602981	7.82E-33	0.37832621	1.10E-07
9.54E-10	-0.3776119	1.17E-07	0.5679251	3.44E-17	0.16917543	0.02133195
1.23E-09	0.55981566	1.19E-16	-0.7332654	1.76E-32	-0.3244962	6.60E-06
1.06E-24	-0.3706897	2.06E-07	0.84314366	3.39E-51	0.32769894	5.28E-06
2.28E-26	0.43720253	4.90E-10	-0.7345189	1.22E-32	-0.4505338	1.24E-10
1.75E-16	-0.2518148	0.00054475	0.59198952	7.04E-19	0.06334407	0.39166954
1.95E-49	0.43592959	5.57E-10	-0.8595444	3.06E-55	-0.5975046	2.76E-19
5.22E-26	-0.3033951	2.69E-05	0.62240547	3.16E-21	0.44618369	1.95E-10
5.46E-15	-0.4992237	4.72E-13	0.77463736	2.90E-38	0.56389636	6.40E-17
7.33E-06	-0.4465877	1.87E-10	0.63056931	6.69E-22	0.46461677	2.70E-11

3.53E-07	-0.325641	6.09E-06	0.63809361	1.53E-22	0.43215485	8.13E-10
2.23E-27	-0.1200553	0.1035771	0.59212506	6.88E-19	0.19683562	0.00724363
8.59E-06	0.21486892	0.00331288	-0.5277382	1.17E-14	-0.4757941	7.68E-12
9.94E-18	0.34094936	2.04E-06	-0.6897576	1.84E-27	-0.6552275	4.57E-24
3.13E-28	-0.3517721	9.11E-07	0.62127768	3.90E-21	0.07320194	0.32205937
2.00E-08	-0.3638298	3.57E-07	0.38044726	9.20E-08	0.04248756	0.5658082
2.93E-34	0.62086531	4.22E-21	-0.707439	2.17E-29	-0.6225533	3.07E-21
0.4936175	-0.3239201	6.87E-06	0.48943773	1.55E-12	0.23591434	0.00122589
1.61E-30	0.46259146	3.38E-11	-0.8952889	3.58E-66	-0.6271339	1.29E-21
5.09E-09	0.39473887	2.70E-08	-0.6699158	1.87E-25	-0.1058141	0.15171489
1.02E-44	0.41070669	6.38E-09	-0.7414721	1.54E-33	-0.5139612	7.31E-14
5.81E-11	-0.3713916	1.94E-07	0.78580432	4.85E-40	0.33781324	2.57E-06
3.08E-32	-0.4754004	8.03E-12	0.84830837	2.03E-52	0.74885811	1.58E-34
3.95E-19	0.52335051	2.12E-14	-0.6896272	1.90E-27	-0.0663705	0.3693946
1.64E-09	-0.4922275	1.11E-12	0.70969018	1.20E-29	0.34721963	1.28E-06
0.00157956	0.48009944	4.67E-12	-0.4700505	1.47E-11	-0.4608793	4.07E-11
2.05E-08	-0.4823067	3.61E-12	0.46614942	2.28E-11	0.60096972	1.51E-19
4.16E-28	0.37375148	1.60E-07	-0.6957246	4.27E-28	-0.7595776	5.03E-36
3.33E-15	-0.4585158	5.26E-11	0.78193704	2.05E-39	0.71902513	9.83E-31
9.88E-29	-0.5443463	1.16E-15	0.89536092	3.38E-66	0.7400223	2.38E-33
3.56E-22	-0.5344719	4.66E-15	0.89423128	8.55E-66	0.72042796	6.69E-31
0.00011667	-0.3380351	2.53E-06	0.48204058	3.73E-12	0.80159682	9.62E-43
5.31E-54	-0.5589774	1.35E-16	0.90055331	4.09E-68	0.65926503	1.93E-24
3.72E-24	-0.5787693	6.21E-18	0.63401462	3.42E-22	0.32071793	8.55E-06
1.01E-29	-0.5673246	3.78E-17	0.9491292	9.01E-94	0.6834947	8.24E-27
3.87E-25	-0.4887544	1.69E-12	0.86251213	5.00E-56	0.80923225	3.87E-44
1.32E-59	-0.3919398	3.44E-08	0.73156902	2.88E-32	0.32211052	7.78E-06
6.11E-53	-0.5592822	1.29E-16	0.91894469	7.25E-76	0.82406172	4.88E-47
2.43E-12	0.76528993	7.45E-37	-0.7284422	7.07E-32	-0.4185899	3.04E-09
2.99E-28	-0.5040152	2.60E-13	0.7598873	4.54E-36	0.46996297	1.49E-11
1.32E-37	0.3647368	3.32E-07	-0.791529	5.41E-41	-0.6570024	3.14E-24
1.51E-28	0.32499347	6.37E-06	-0.7860769	4.37E-40	-0.6184402	6.61E-21
1.70E-39	-0.5996813	1.89E-19	0.89374334	1.27E-65	0.57393616	1.34E-17
0.82060057	0.24010973	0.00099476	-0.4487806	1.49E-10	-0.2573069	0.00040661
1.51E-35	0.53004054	8.59E-15	-0.8392051	2.71E-50	-0.8442221	1.90E-51
0.00392399	-0.0723025	0.32805694	-0.1419195	0.05398023	0.39177918	3.49E-08
5.59E-15	-0.3902326	4.00E-08	0.71524011	2.75E-30	0.85586998	2.72E-54
9.09E-38	0.41361492	4.86E-09	-0.8341083	3.67E-49	-0.6432521	5.45E-23
5.56E-18	-0.337749	2.58E-06	0.5415237	1.73E-15	0.09829441	0.18315196
9.19E-24	-0.5785532	6.43E-18	0.76690166	4.30E-37	0.261314	0.00032715
1.11E-12	-0.2846234	8.62E-05	0.62879569	9.41E-22	0.25703259	0.00041266
0.47818936	0.2786941	0.00012236	-0.4020269	1.41E-08	-0.2363871	0.00119758
9.35E-13	-0.5275761	1.20E-14	0.81290252	7.84E-45	0.61283913	1.84E-20
1.19E-10	0.42211174	2.17E-09	-0.7238357	2.60E-31	-0.1983609	0.00679632
0.00013417	0.25035634	0.00058812	-0.5448754	1.07E-15	-0.5742193	1.28E-17
8.55E-15	0.24573724	0.00074737	-0.3828454	7.52E-08	0.09426102	0.20186989

2.42E-07	0.21936083	0.0026995	-0.4107583	6.34E-09	-0.6204855	4.53E-21
2.33E-17	-0.5138759	7.39E-14	0.72136899	5.16E-31	0.60575542	6.55E-20
1.13E-25	-0.4405899	3.47E-10	0.82352483	6.29E-47	0.69329209	7.78E-28
6.63E-05	-0.3629869	3.82E-07	0.53821937	2.76E-15	0.56892885	2.95E-17
2.84E-35	0.52541543	1.61E-14	-0.6067043	5.54E-20	-0.5908515	8.52E-19
8.78E-19	-0.4535072	9.02E-11	0.66948604	2.06E-25	0.19933851	0.00652275
0.41461628	-0.1644194	0.02532355	0.41373435	4.81E-09	0.12481887	0.09048592
0.02802486	0.10438675	0.15734541	-0.3148459	1.27E-05	-0.3798898	9.64E-08
7.31E-06	-0.2774725	0.0001314	0.63780052	1.62E-22	0.49449786	8.43E-13
6.25E-43	0.35634219	6.41E-07	-0.7883528	1.84E-40	-0.3625234	3.96E-07
2.52E-17	0.15326504	0.0372674	-0.5382807	2.74E-15	-0.6200178	4.94E-21
4.93E-30	0.6147129	1.31E-20	-0.8952601	3.67E-66	-0.5876565	1.45E-18
4.49E-27	0.46986687	1.50E-11	-0.7404306	2.10E-33	-0.842946	3.77E-51
1.36E-11	0.30622878	2.24E-05	-0.6220153	3.40E-21	-0.658176	2.44E-24
8.17E-13	-0.4312577	8.88E-10	0.75154139	6.78E-35	0.6255954	1.73E-21
4.71E-18	0.3586468	5.36E-07	-0.6918443	1.11E-27	-0.5575888	1.66E-16
3.84E-12	-0.4608929	4.06E-11	0.58502897	2.24E-18	0.21394058	0.00345435
3.63E-14	0.25958807	0.00035942	-0.6281372	1.07E-21	-0.6033853	9.94E-20
2.39E-14	0.320469	8.70E-06	-0.6573453	2.92E-24	-0.7716857	8.21E-38
5.24E-41	0.5201124	3.26E-14	-0.8998099	7.81E-68	-0.7252197	1.76E-31
8.31E-39	0.2159421	0.00315586	-0.6776047	3.26E-26	-0.4626189	3.37E-11
5.60E-27	-0.4999565	4.31E-13	0.67903634	2.34E-26	0.2800936	0.00011272
3.17E-21	-0.0714077	0.33409406	0.55340913	3.10E-16	0.28316305	9.40E-05
0.00318281	-0.0271507	0.71372771	0.39407148	2.86E-08	0.23037861	0.00160611
6.91E-30	0.35924629	5.12E-07	-0.7483426	1.86E-34	-0.4040119	1.18E-08
0.00033869	0.27244154	0.00017563	-0.4279431	1.23E-09	-0.6128858	1.83E-20
1.92E-25	-0.4964772	6.62E-13	0.60284019	1.09E-19	0.12942597	0.07911495
1.53E-26	0.35123943	9.48E-07	-0.755653	1.81E-35	-0.539381	2.35E-15
3.21E-10	-0.4164538	3.72E-09	0.7900923	9.43E-41	0.59644755	3.30E-19
2.17E-13	-0.4572356	6.05E-11	0.48145268	3.99E-12	0.2084062	0.00441689
2.95E-08	0.69754332	2.71E-28	-0.6109627	2.59E-20	-0.369783	2.22E-07
4.41E-05	0.24896396	0.00063247	-0.5700977	2.45E-17	-0.5285668	1.05E-14
4.37E-35	-0.5720898	1.80E-17	0.86489996	1.13E-56	0.74062129	1.99E-33
0.00015029	0.28658648	7.66E-05	-0.5001295	4.22E-13	-0.1171642	0.11222307
1.34E-23	-0.286947	7.49E-05	0.73007232	4.43E-32	0.44847798	1.53E-10
2.29E-11	-0.4544402	8.16E-11	0.70645912	2.80E-29	0.55625152	2.03E-16
8.59E-26	0.3504201	1.01E-06	-0.7833784	1.20E-39	-0.4546137	8.01E-11
3.73E-17	-0.5047376	2.37E-13	0.86276773	4.27E-56	0.57148178	1.98E-17
2.14E-43	-0.5246697	1.78E-14	0.72112258	5.52E-31	0.46148548	3.81E-11
8.62E-08	-0.4451822	2.16E-10	0.70392617	5.38E-29	0.32294646	7.34E-06
8.16E-36	0.45264219	9.89E-11	-0.8188519	5.46E-46	-0.7808779	3.03E-39
4.47E-28	0.24185657	0.00091091	-0.7994601	2.31E-42	-0.4163635	3.76E-09
1.78E-06	0.34892831	1.13E-06	-0.717309	1.57E-30	-0.36301	3.81E-07
1.39E-28	0.36326087	3.73E-07	-0.6709732	1.48E-25	-0.4945392	8.39E-13
6.19E-15	-0.4906828	1.34E-12	0.76003435	4.33E-36	0.33377547	3.44E-06
0.03627758	-0.3389929	2.36E-06	0.52332573	2.13E-14	0.59042727	9.15E-19

6.20E-07	0.09410245	0.20263304	-0.488427	1.75E-12	-0.6840699	7.19E-27
7.56E-27	-0.5658841	4.72E-17	0.85990153	2.47E-55	0.54694878	7.97E-16
1.78E-32	-0.5719625	1.83E-17	0.9049759	7.85E-70	0.77945513	5.11E-39
0.00399577	0.56217069	8.33E-17	-0.5483942	6.46E-16	-0.364411	3.41E-07
4.01E-11	0.22194756	0.0023949	-0.5776337	7.45E-18	-0.6426529	6.15E-23
1.06E-24	-0.3755147	1.39E-07	0.78145086	2.46E-39	0.52865628	1.04E-14
1.50E-20	0.34796184	1.21E-06	-0.7662879	5.30E-37	-0.4942213	8.72E-13
7.48E-09	0.29975382	3.40E-05	-0.588508	1.26E-18	-0.4443098	2.37E-10
2.00E-07	-0.2660249	0.00025224	0.61998914	4.96E-21	0.66341361	7.88E-25
3.27E-28	-0.5300213	8.61E-15	0.87091683	2.33E-58	0.51434091	6.96E-14
2.15E-08	0.16636903	0.02361578	-0.4239737	1.81E-09	-0.5087525	1.43E-13
0.00218227	0.3020232	2.94E-05	-0.6578296	2.63E-24	-0.4442874	2.37E-10
9.42E-39	0.34100426	2.04E-06	-0.8310851	1.66E-48	-0.5741478	1.30E-17
3.39E-05	0.22532209	0.00204448	-0.4985398	5.14E-13	-0.4416097	3.13E-10
5.67E-22	-0.5284352	1.07E-14	0.84413372	1.99E-51	0.72531145	1.72E-31
3.05E-34	0.2323646	0.0014588	-0.7075573	2.10E-29	-0.3206195	8.61E-06
2.31E-43	-0.5823774	3.46E-18	0.96026354	2.29E-103	0.64610834	3.05E-23
1.29E-14	-0.4397484	3.78E-10	0.78533208	5.79E-40	0.46985696	1.51E-11
1.25E-41	-0.4711367	1.30E-11	0.78435256	8.36E-40	0.28078591	0.00010822
2.10E-13	-0.3855732	5.96E-08	0.74490334	5.39E-34	0.29066921	5.98E-05
2.97E-22	-0.4376734	4.67E-10	0.68193529	1.19E-26	0.18979913	0.00966432
0.11803066	0.17717915	0.01583558	-0.4921131	1.13E-12	-0.0681486	0.35667977
7.87E-20	-0.4471869	1.76E-10	0.81389111	5.07E-45	0.33737354	2.65E-06
4.18E-38	0.57880199	6.17E-18	-0.8019248	8.41E-43	-0.737163	5.59E-33
3.37E-30	-0.2913138	5.74E-05	0.57079885	2.20E-17	0.08507373	0.24957858
1.11E-25	-0.5132092	8.06E-14	0.90475699	9.59E-70	0.76329858	1.46E-36
2.58E-16	0.00729318	0.92151388	-0.3462804	1.38E-06	-0.0293823	0.69135322
1.66E-25	-0.4656956	2.40E-11	0.81154764	1.42E-44	0.63328127	3.95E-22
0.00107862	0.44343714	2.59E-10	-0.61225	2.05E-20	-0.0001166	0.99874373
3.19E-06	0.25220498	0.00053366	-0.6440924	4.60E-23	-0.352093	8.89E-07
3.90E-46	-0.5827206	3.27E-18	0.88544779	8.35E-63	0.76849756	2.49E-37
2.24E-20	0.55030462	4.89E-16	-0.7714069	9.05E-38	-0.6190562	5.90E-21
1.65E-15	0.22637357	0.00194524	-0.5999363	1.81E-19	-0.7725223	6.12E-38
5.28E-46	-0.3686441	2.43E-07	0.80143991	1.03E-42	0.4339287	6.81E-10
3.10E-47	-0.4772468	6.50E-12	0.85009173	7.52E-53	0.43097257	9.14E-10
3.98E-42	0.25079779	0.00057467	-0.6188947	6.08E-21	-0.4334704	7.13E-10
1.77E-30	-0.5451321	1.04E-15	0.88719965	2.21E-63	0.605437	6.93E-20
2.30E-29	-0.323417	7.11E-06	0.85439659	6.43E-54	0.42571793	1.53E-09
6.56E-38	0.58169982	3.86E-18	-0.7027883	7.21E-29	-0.511712	9.77E-14
8.61E-47	-0.5903354	9.29E-19	0.87954404	6.26E-61	0.46217988	3.53E-11
0.251209	0.10666968	0.14841371	-0.4190135	2.92E-09	-0.4178137	3.27E-09
0.0017348	0.56883197	2.99E-17	-0.4649137	2.61E-11	-0.5419198	1.64E-15
0.0016704	-0.4999431	4.32E-13	0.60296809	1.07E-19	0.53978513	2.22E-15
4.93E-39	0.3101839	1.73E-05	-0.6959463	4.04E-28	-0.4712466	1.29E-11
3.35E-11	0.57537039	1.07E-17	-0.4023714	1.37E-08	-0.2349077	0.00128821
2.87E-10	-0.0666423	0.36743337	0.67606351	4.65E-26	0.42111888	2.39E-09

1.75E-23	0.379032	1.04E-07	-0.7793091	5.39E-39	-0.6977826	2.56E-28
1.19E-08	0.66742147	3.26E-25	-0.6685518	2.54E-25	-0.0798275	0.28008109
1.85E-57	-0.3925626	3.26E-08	0.71165493	7.16E-30	0.4743109	9.10E-12
1.07E-21	-0.4566734	6.42E-11	0.78848888	1.75E-40	0.34506161	1.51E-06
5.91E-21	0.35182518	9.07E-07	-0.6016653	1.34E-19	-0.7183321	1.19E-30
7.92E-11	0.1966558	0.00729805	-0.4718204	1.21E-11	0.00284694	0.96932079
8.68E-47	-0.594451	4.64E-19	0.89949719	1.02E-67	0.50967596	1.27E-13
2.35E-17	0.4985953	5.10E-13	-0.7849376	6.71E-40	-0.1544271	0.03583397
9.97E-44	0.5602579	1.11E-16	-0.8127591	8.35E-45	-0.6771672	3.60E-26
1.81E-69	-0.4896402	1.52E-12	0.91086733	2.96E-72	0.61939808	5.54E-21
7.70E-18	-0.4402664	3.59E-10	0.72549651	1.63E-31	0.57473075	1.18E-17
1.93E-37	-0.5199354	3.34E-14	0.9181627	1.68E-75	0.73612356	7.60E-33
2.02E-30	0.2829512	9.52E-05	-0.7061074	3.07E-29	-0.4066059	9.30E-09
4.63E-28	0.41130127	6.03E-09	-0.758693	6.74E-36	-0.2246924	0.0021061
0.00014362	0.30809991	1.99E-05	-0.5077189	1.63E-13	-0.0940646	0.20281553
3.82E-08	-0.4016938	1.45E-08	0.71161632	7.24E-30	0.55656518	1.94E-16
3.46E-32	-0.2978809	3.82E-05	0.6719948	1.17E-25	0.34829786	1.18E-06
6.51E-16	-0.4314198	8.74E-10	0.70725493	2.28E-29	0.45517201	7.55E-11
9.09E-31	-0.5945335	4.58E-19	0.92233386	1.70E-77	0.60650579	5.73E-20
0.00416183	-0.3045891	2.49E-05	0.54283971	1.44E-15	0.12663866	0.08584774
8.19E-05	0.39575283	2.47E-08	-0.5661798	4.51E-17	-0.452516	1.00E-10
1.94E-16	-0.4424277	2.88E-10	0.8114734	1.47E-44	0.57833482	6.66E-18
6.98E-08	0.26435025	0.00027682	-0.6312001	5.92E-22	-0.4379767	4.53E-10
0.08245343	-0.2354457	0.00125455	0.53512213	4.26E-15	0.21028497	0.00406602
3.64E-19	0.36584976	3.04E-07	-0.491319	1.24E-12	-0.506314	1.95E-13
7.96E-09	-0.3714373	1.94E-07	0.76871953	2.30E-37	0.34935374	1.09E-06
2.30E-46	-0.5163616	5.35E-14	0.87477476	1.74E-59	0.6478638	2.13E-23
0.00036834	-0.3288897	4.86E-06	0.37652317	1.28E-07	-0.0086928	0.90651472
0.57381526	-0.3372338	2.68E-06	0.33370713	3.45E-06	-0.0174769	0.81334073
5.08E-27	-0.5393898	2.34E-15	0.87854615	1.27E-60	0.67215068	1.13E-25
4.05E-17	-0.1908066	0.00927882	0.78335649	1.21E-39	0.62059478	4.43E-21
1.19E-39	0.44886227	1.47E-10	-0.6432387	5.47E-23	-0.289827	6.29E-05
4.79E-58	0.39277242	3.20E-08	-0.6664819	4.01E-25	-0.4306008	9.48E-10
0.00092743	-0.3681335	2.53E-07	0.62178074	3.55E-21	0.53541648	4.09E-15
9.54E-30	-0.5394616	2.32E-15	0.80039442	1.58E-42	0.51067199	1.12E-13
1.87E-28	-0.414237	4.59E-09	0.65632941	3.62E-24	0.30872671	1.91E-05
8.59E-15	0.38681011	5.37E-08	-0.7067933	2.57E-29	-0.5077032	1.63E-13
2.38E-14	0.44590045	2.01E-10	-0.7633378	1.44E-36	-0.5689253	2.95E-17
1.04E-28	-0.5156357	5.88E-14	0.9274317	4.33E-80	0.66108101	1.31E-24
1.49E-45	0.45635588	6.65E-11	-0.6640748	6.82E-25	-0.5213097	2.79E-14
2.02E-06	-0.3227277	7.45E-06	0.63877562	1.34E-22	0.41761822	3.33E-09
1.31E-19	0.32838985	5.03E-06	-0.7018946	9.05E-29	-0.5533327	3.13E-16
1.85E-13	-0.4304257	9.65E-10	0.8145228	3.83E-45	0.61438992	1.39E-20
9.05E-33	-0.5988084	2.20E-19	0.74606745	3.77E-34	0.47535771	8.07E-12
9.51E-50	-0.5142727	7.02E-14	0.92078481	9.66E-77	0.62270579	2.99E-21
1.61E-05	-0.2948271	4.62E-05	0.61679452	8.96E-21	0.43756613	4.72E-10

9.21E-18	-0.3935538	2.99E-08	0.71676622	1.82E-30	0.26429263	0.0002777
1.18E-16	-0.4891406	1.61E-12	0.84038268	1.46E-50	0.65916288	1.98E-24
1.63E-25	-0.3297342	4.58E-06	0.7219918	4.34E-31	0.38598943	5.76E-08
9.56E-19	0.47380305	9.64E-12	-0.7226653	3.60E-31	-0.1618065	0.02777784
2.08E-40	0.39431171	2.80E-08	-0.7512023	7.55E-35	-0.4850348	2.62E-12
3.60E-13	0.3190058	9.61E-06	-0.6500943	1.34E-23	-0.7351509	1.01E-32
3.84E-09	-0.402709	1.33E-08	0.67522558	5.63E-26	0.52591421	1.50E-14
3.92E-38	-0.3519736	8.97E-07	0.63319352	4.02E-22	0.2053019	0.00505659
5.59E-08	0.33693161	2.74E-06	-0.6825409	1.03E-26	-0.5786128	6.36E-18
2.11E-12	-0.4894642	1.55E-12	0.76928622	1.89E-37	0.25866002	0.00037797
1.17E-37	-0.5170289	4.90E-14	0.92068561	1.08E-76	0.70023591	1.38E-28
2.51E-46	-0.569321	2.77E-17	0.95176945	7.75E-96	0.78598255	4.53E-40
6.75E-20	-0.4299327	1.01E-09	0.70272797	7.32E-29	0.14570444	0.04782322
1.06E-11	-0.4429209	2.73E-10	0.47625277	7.29E-12	0.15910909	0.03052201
4.53E-26	0.37224626	1.81E-07	-0.6770835	3.68E-26	-0.3015963	3.02E-05
7.08E-16	-0.4754392	8.00E-12	0.84398097	2.16E-51	0.67297567	9.39E-26
5.01E-23	0.26981959	0.00020385	-0.7198133	7.92E-31	-0.3717106	1.89E-07
7.28E-11	-0.3632619	3.73E-07	0.79971504	2.08E-42	0.49770272	5.70E-13
2.70E-22	-0.4869418	2.09E-12	0.87120334	1.93E-58	0.53814534	2.79E-15
7.15E-09	0.38148958	8.43E-08	-0.6707415	1.55E-25	-0.559903	1.18E-16
1.15E-21	0.66328874	8.09E-25	-0.8360113	1.40E-49	-0.3686726	2.42E-07
8.30E-25	-0.3727428	1.74E-07	0.81442423	4.00E-45	0.81637827	1.67E-45
1.05E-21	0.38896879	4.46E-08	-0.626005	1.60E-21	-0.7040181	5.26E-29
3.28E-30	-0.6295634	8.12E-22	0.93258352	6.51E-83	0.64181104	7.29E-23
1.46E-11	0.33974066	2.23E-06	-0.5493731	5.60E-16	-0.5670826	3.92E-17
2.04E-10	-0.4776836	6.18E-12	0.75048859	9.46E-35	0.64198632	7.04E-23
2.90E-41	-0.5936693	5.30E-19	0.96510989	1.94E-108	0.6715502	1.30E-25
5.05E-18	-0.3895029	4.26E-08	0.77343279	4.44E-38	0.38030907	9.31E-08
5.92E-17	-0.3190724	9.57E-06	0.70247434	7.81E-29	0.41510853	4.23E-09
1.08E-05	-0.4617629	3.70E-11	0.56495282	5.45E-17	0.720232	7.06E-31
2.69E-63	-0.4698542	1.51E-11	0.92932588	4.20E-81	0.64952497	1.51E-23
0.00286094	0.45341665	9.10E-11	-0.5104818	1.14E-13	-0.4877256	1.91E-12
9.87E-36	-0.4414505	3.18E-10	0.64069132	9.13E-23	0.26427657	0.00027795
9.43E-35	-0.5475171	7.34E-16	0.90661265	1.73E-70	0.66220969	1.02E-24
2.50E-15	0.37744998	1.18E-07	-0.6622628	1.01E-24	-0.3376537	2.60E-06
1.05E-14	-0.520307	3.18E-14	0.69417075	6.27E-28	0.39106877	3.72E-08
8.40E-42	-0.2225209	0.00233176	0.70607874	3.09E-29	0.24907904	0.00062869
1.20E-44	-0.3668551	2.80E-07	0.80499829	2.34E-43	0.34704517	1.30E-06
1.67E-15	0.37118215	1.98E-07	-0.8105906	2.15E-44	-0.4450641	2.19E-10
5.09E-22	-0.4831091	3.29E-12	0.86374739	2.32E-56	0.77984121	4.43E-39
1.19E-12	0.27837235	0.00012469	-0.7542854	2.82E-35	-0.6475854	2.25E-23
3.08E-18	-0.1301441	0.07745118	0.66990267	1.88E-25	0.32714839	5.49E-06
5.12E-17	0.3004496	3.25E-05	-0.5743674	1.25E-17	-0.7787061	6.71E-39
1.99E-36	-0.3302324	4.42E-06	0.61669296	9.13E-21	0.18515276	0.01163137
1.98E-51	-0.4134056	4.96E-09	0.7761534	1.68E-38	0.33781484	2.57E-06
4.99E-05	-0.3740492	1.56E-07	0.65532028	4.49E-24	0.37296038	1.71E-07

5.21E-12	-0.4657795	2.37E-11	0.77515859	2.40E-38	0.73447417	1.24E-32
8.22E-46	-0.5902003	9.51E-19	0.96597866	2.01E-109	0.69621231	3.78E-28
8.60E-15	-0.2122258	0.0037301	0.72173265	4.66E-31	0.70186615	9.12E-29
2.64E-20	0.20064571	0.0061723	-0.5035655	2.75E-13	-0.2138722	0.00346498
1.67E-34	-0.5025929	3.11E-13	0.86891571	8.64E-58	0.85861989	5.33E-55
5.29E-60	0.48727109	2.01E-12	-0.7095578	1.25E-29	-0.4135822	4.88E-09
8.19E-35	-0.5345537	4.61E-15	0.89860312	2.21E-67	0.59134647	7.84E-19
5.40E-32	-0.3310764	4.16E-06	0.55308928	3.25E-16	0.12146753	0.0995491
6.02E-19	0.31062111	1.68E-05	-0.7998397	1.98E-42	-0.4251959	1.61E-09
1.61E-29	0.41909966	2.90E-09	-0.7497825	1.18E-34	-0.4563397	6.66E-11
5.20E-44	-0.572793	1.61E-17	0.86996176	4.37E-58	0.56599509	4.64E-17
2.57E-34	-0.5117771	9.69E-14	0.91639752	1.09E-74	0.62173575	3.58E-21
0.37495291	-0.2506526	0.00057906	0.35283928	8.39E-07	-0.0219483	0.76681699
6.45E-27	-0.3548604	7.19E-07	0.60407192	8.81E-20	0.08556591	0.24683989
0.81995344	-0.3358063	2.97E-06	0.44568681	2.05E-10	0.08903235	0.22814233
2.41E-29	0.63006167	7.37E-22	-0.7498592	1.15E-34	-0.548553	6.31E-16
1.87E-12	0.59720374	2.90E-19	-0.637841	1.61E-22	-0.3648961	3.28E-07
0.06626023	-0.209822	0.00415006	0.50793115	1.59E-13	0.37365763	1.62E-07
2.75E-74	-0.5120969	9.30E-14	0.8531373	1.33E-53	0.51236185	8.99E-14
3.07E-08	-0.3654936	3.13E-07	0.6650184	5.54E-25	0.2483941	0.0006515
4.91E-75	-0.5651363	5.30E-17	0.87623219	6.40E-60	0.63720552	1.83E-22
5.17E-19	-0.4937853	9.20E-13	0.76944925	1.79E-37	0.35287936	8.37E-07
3.78E-43	0.44655336	1.88E-10	-0.8562548	2.17E-54	-0.5578461	1.60E-16
0.00389847	0.18460217	0.01188636	-0.5532765	3.16E-16	0.05156173	0.48578664
4.45E-18	-0.6211413	4.00E-21	0.82509109	3.00E-47	0.62554579	1.75E-21
6.98E-08	-0.3012098	3.10E-05	0.55092825	4.47E-16	0.50648709	1.90E-13
4.95E-22	-0.4833361	3.20E-12	0.55247269	3.56E-16	0.70545807	3.63E-29
0.06820414	-0.3948819	2.66E-08	0.53465933	4.54E-15	0.47597935	7.52E-12
1.81E-15	0.63153251	5.55E-22	-0.5960465	3.54E-19	-0.5102348	1.18E-13
0.09291735	0.68525139	5.43E-27	-0.4932104	9.86E-13	-0.3165358	1.14E-05
4.69E-37	-0.5079072	1.59E-13	0.8562821	2.14E-54	0.37554266	1.38E-07
3.13E-08	0.51828414	4.15E-14	-0.6669239	3.64E-25	-0.7346364	1.18E-32
1.05E-27	0.18836905	0.01023582	-0.6285931	9.78E-22	-0.5465061	8.49E-16
3.54E-63	-0.479136	5.23E-12	0.91342118	2.33E-73	0.50591182	2.05E-13
1.32E-07	0.54047569	2.01E-15	-0.7215362	4.92E-31	-0.2756069	0.00014642
4.97E-13	0.28951369	6.41E-05	-0.5231733	2.17E-14	-0.2844353	8.71E-05
2.26E-06	-0.3170794	1.09E-05	0.55361837	3.01E-16	-0.0629818	0.3943894
6.87E-26	0.11214591	0.12855899	-0.5181035	4.25E-14	-0.4310197	9.10E-10
4.66E-35	-0.3102245	1.73E-05	0.72988735	4.68E-32	0.26307701	0.00029698
6.73E-49	-0.4751464	8.27E-12	0.9023861	8.13E-69	0.60442719	8.28E-20
0.00219805	0.12669572	0.08570543	-0.4744453	8.96E-12	-0.4761315	7.39E-12
2.80E-24	-0.5296145	9.10E-15	0.73273212	2.05E-32	0.37916968	1.02E-07
1.00E-16	-0.3910539	3.72E-08	0.75792973	8.65E-36	0.18609393	0.01120662
9.11E-13	-0.4669493	2.08E-11	0.75518378	2.11E-35	0.30943465	1.82E-05
3.15E-43	-0.5131703	8.10E-14	0.88551176	7.96E-63	0.40810361	8.11E-09
1.33E-75	-0.5872604	1.55E-18	0.86253023	4.94E-56	0.54877622	6.11E-16

1.48E-66	-0.4572629	6.03E-11	0.85081351	5.00E-53	0.5403588	2.04E-15
1.04E-41	-0.4886549	1.71E-12	0.82606303	1.89E-47	0.382742	7.59E-08
1.61E-54	-0.522628	2.34E-14	0.97235291	1.53E-117	0.75241686	5.13E-35
0.00026491	0.03259017	0.659657	-0.4408896	3.37E-10	-0.0857326	0.24591726
2.97E-24	0.12667117	0.08576664	-0.4264135	1.43E-09	-0.1692733	0.02125587
8.36E-23	-0.4341971	6.63E-10	0.72791351	8.22E-32	0.56572164	4.84E-17
4.03E-23	0.44085727	3.38E-10	-0.7465771	3.22E-34	-0.6351538	2.74E-22
4.62E-15	0.50752594	1.67E-13	-0.6132594	1.71E-20	-0.4020775	1.40E-08
1.89E-09	-0.499001	4.85E-13	0.55198966	3.82E-16	0.57741788	7.71E-18
1.18E-19	-0.3397991	2.22E-06	0.74114943	1.69E-33	0.19807678	0.00687774
0.03426386	0.08067168	0.27501044	-0.3278126	5.24E-06	-0.199492	0.00648069
2.95E-11	-0.3426651	1.80E-06	0.660988	1.33E-24	0.21532043	0.00324598
9.94E-35	0.2244388	0.00213139	-0.6625038	9.60E-25	-0.3641484	3.48E-07
9.00E-07	0.25254495	0.00052417	-0.5010814	3.75E-13	-0.5573086	1.74E-16
4.93E-11	-0.3187049	9.81E-06	0.50214798	3.28E-13	0.54069397	1.95E-15
9.83E-30	-0.5958705	3.65E-19	0.81166519	1.35E-44	0.61619452	1.00E-20
0.30904653	0.09655037	0.19108277	-0.3162309	1.16E-05	-0.5897349	1.03E-18
3.26E-23	0.50466583	2.40E-13	-0.7160704	2.20E-30	-0.1282536	0.08189303
3.52E-58	-0.4526483	9.88E-11	0.81631672	1.72E-45	0.60969023	3.25E-20
4.46E-24	0.316747	1.12E-05	-0.5060261	2.02E-13	-0.1475078	0.0451024
7.68E-07	0.30481668	2.46E-05	-0.5667415	4.14E-17	-0.6743136	6.93E-26
4.57E-13	0.45421788	8.36E-11	-0.6506661	1.19E-23	-0.5948631	4.33E-19
4.76E-44	-0.3207244	8.55E-06	0.78635077	3.94E-40	0.43180059	8.42E-10
2.02E-45	-0.4265935	1.41E-09	0.73626129	7.30E-33	0.27237785	0.00017627
1.20E-37	-0.4523072	1.02E-10	0.83902646	2.97E-50	0.43506823	6.07E-10
1.68E-08	0.43173721	8.47E-10	-0.709404	1.30E-29	-0.0869845	0.23906345
3.29E-63	-0.5168485	5.02E-14	0.80060965	1.44E-42	0.49491975	8.01E-13
1.33E-22	-0.5076707	1.64E-13	0.88890521	5.95E-64	0.69450703	5.77E-28
1.15E-52	-0.4708203	1.35E-11	0.84391791	2.24E-51	0.35141849	9.35E-07
2.48E-13	0.63273158	4.40E-22	-0.7436246	7.98E-34	-0.46439	2.77E-11
3.70E-27	-0.6594368	1.86E-24	0.83286445	6.85E-49	0.60944031	3.40E-20
6.20E-17	0.59619807	3.45E-19	-0.5631088	7.22E-17	-0.0916956	0.21447342
8.53E-15	0.48588234	2.37E-12	-0.8378431	5.49E-50	-0.5515035	4.10E-16
4.48E-63	-0.4689894	1.66E-11	0.91779292	2.50E-75	0.75365329	3.46E-35
1.09E-12	0.57700494	8.24E-18	-0.7877901	2.28E-40	-0.6252425	1.85E-21
2.02E-40	-0.3872577	5.16E-08	0.90875574	2.28E-71	0.73791734	4.47E-33
8.17E-42	-0.5614168	9.35E-17	0.93705874	1.49E-85	0.64941834	1.54E-23
1.92E-11	0.61348361	1.64E-20	-0.6560814	3.82E-24	-0.1301933	0.07733812
3.20E-42	-0.2189548	0.00275038	0.64377306	4.91E-23	0.21715689	0.00298627
1.80E-26	-0.3944262	2.77E-08	0.45743076	5.92E-11	0.24084109	0.00095883
3.01E-32	0.37986903	9.66E-08	-0.6530287	7.27E-24	-0.7251911	1.78E-31
0.00882567	-0.2892247	6.53E-05	0.42468928	1.69E-09	0.52961171	9.11E-15
1.42E-07	-0.4535032	9.02E-11	0.57563794	1.02E-17	0.43492677	6.16E-10
3.30E-11	-0.4200988	2.63E-09	0.74508686	5.10E-34	0.57630631	9.21E-18
3.26E-12	-0.3976341	2.09E-08	0.78942759	1.22E-40	0.31901832	9.60E-06
1.61E-29	-0.4884845	1.74E-12	0.84349857	2.80E-51	0.3568749	6.15E-07

2.69E-41	-0.508207	1.53E-13	0.92315972	6.66E-78	0.61782191	7.41E-21
6.37E-22	-0.4561313	6.81E-11	0.72706959	1.04E-31	0.2953745	4.47E-05
1.84E-17	-0.5322325	6.36E-15	0.72098756	5.73E-31	0.29937433	3.48E-05
1.03E-45	-0.468296	1.79E-11	0.80335359	4.65E-43	0.32611195	5.90E-06
9.52E-24	0.59776327	2.64E-19	-0.7649655	8.31E-37	-0.2464133	0.00072182
1.22E-20	-0.3623378	4.02E-07	0.63942286	1.18E-22	0.29375352	4.94E-05
0.00035982	-0.3242766	6.70E-06	0.64409514	4.60E-23	0.31294259	1.44E-05
7.89E-20	-0.5364343	3.55E-15	0.81790607	8.40E-46	0.57753882	7.56E-18
2.49E-33	-0.4392664	3.97E-10	0.81695599	1.29E-45	0.27161276	0.00018413
4.51E-53	-0.5784977	6.48E-18	0.92293993	8.56E-78	0.58526794	2.16E-18
0.00013461	0.33591554	2.95E-06	-0.6667079	3.82E-25	-0.0388486	0.59957317
4.56E-18	-0.523784	2.00E-14	0.80765747	7.60E-44	0.67637843	4.32E-26
0.60043545	-0.2594853	0.00036143	0.46098157	4.03E-11	0.44531679	2.13E-10
5.94E-06	-0.4317261	8.48E-10	0.62846556	1.00E-21	0.71670598	1.85E-30
9.83E-05	-0.3628398	3.86E-07	0.66585636	4.61E-25	0.62482194	2.01E-21
2.33E-05	-0.2940475	4.85E-05	0.48902371	1.63E-12	0.68047395	1.67E-26
3.03E-40	-0.5885116	1.26E-18	0.7802933	3.76E-39	0.28818053	6.95E-05
2.22E-28	-0.5580135	1.56E-16	0.82910641	4.36E-48	0.47737391	6.40E-12
7.98E-38	0.36516998	3.21E-07	-0.6826389	1.01E-26	-0.279181	0.00011892
1.51E-36	-0.4224355	2.10E-09	0.89901924	1.55E-67	0.74421538	6.66E-34
8.84E-14	0.5518922	3.88E-16	-0.438304	4.38E-10	-0.1665053	0.02350025
2.88E-15	-0.4523585	1.02E-10	0.82028325	2.84E-46	0.58232596	3.49E-18
4.29E-21	0.23826362	0.00109103	-0.5380488	2.83E-15	-0.6351419	2.74E-22
5.10E-33	0.15366353	0.0367705	-0.5569238	1.84E-16	-0.330016	4.49E-06
2.07E-42	-0.4758509	7.63E-12	0.85073605	5.23E-53	0.47170954	1.22E-11
3.90E-38	0.40863489	7.72E-09	-0.7617246	2.47E-36	-0.4062482	9.61E-09
2.15E-52	-0.3578374	5.71E-07	0.79678055	6.80E-42	0.43106451	9.06E-10
1.19E-23	-0.2690511	0.00021289	0.52002858	3.30E-14	-0.0224605	0.76153695
3.92E-22	0.48115557	4.13E-12	-0.7958814	9.74E-42	-0.4382307	4.41E-10
6.36E-17	0.43221199	8.08E-10	-0.8174427	1.04E-45	-0.6230163	2.82E-21
7.14E-07	-0.1841484	0.01210019	0.65996424	1.66E-24	0.56432681	6.00E-17
8.95E-25	-0.481892	3.79E-12	0.87291111	6.16E-59	0.57833416	6.66E-18
8.37E-23	0.52184646	2.59E-14	-0.6756707	5.08E-26	-0.2015076	0.00595053
1.05E-08	-0.5429692	1.41E-15	0.76445073	9.89E-37	0.62112129	4.02E-21
3.76E-13	0.32464923	6.53E-06	-0.5958835	3.64E-19	-0.1035982	0.1605227
2.43E-13	0.33736089	2.65E-06	-0.7188057	1.04E-30	-0.0703827	0.34109617
1.16E-25	0.2913813	5.72E-05	-0.7075412	2.11E-29	-0.3184541	9.98E-06
2.54E-35	0.27782694	0.00012872	-0.7753131	2.28E-38	-0.4423916	2.89E-10
3.90E-48	0.42863	1.15E-09	-0.7349943	1.06E-32	-0.3557058	6.74E-07
3.99E-12	-0.4381042	4.47E-10	0.63371021	3.63E-22	0.29064702	5.98E-05
4.96E-25	-0.4221907	2.15E-09	0.83311625	6.04E-49	0.3048718	2.45E-05
1.06E-11	0.72262739	3.64E-31	-0.6574767	2.84E-24	-0.6544447	5.40E-24
1.60E-11	0.36305739	3.79E-07	-0.5205171	3.09E-14	-0.1160759	0.1156204
2.28E-36	0.39859425	1.92E-08	-0.6919371	1.08E-27	-0.567251	3.82E-17
8.54E-05	0.40603267	9.80E-09	-0.4964678	6.63E-13	0.05498725	0.45723897
1.93E-25	0.64744321	2.32E-23	-0.7769511	1.27E-38	-0.3658664	3.03E-07

1.39E-29	-0.5729788	1.56E-17	0.92917661	5.06E-81	0.6331219	4.08E-22
2.35E-19	-0.47049	1.40E-11	0.84515105	1.15E-51	0.70977124	1.18E-29
1.14E-59	-0.5050925	2.27E-13	0.91432164	9.35E-74	0.55314905	3.22E-16
1.11E-12	0.28624975	7.81E-05	-0.6645174	6.19E-25	-0.4786387	5.54E-12
0.01097994	0.42393237	1.82E-09	-0.654657	5.16E-24	-0.3222829	7.69E-06
4.29E-21	0.29140579	5.71E-05	-0.6857484	4.83E-27	-0.6467437	2.68E-23
7.17E-21	0.38762981	5.00E-08	-0.7762251	1.64E-38	-0.5540019	2.84E-16
1.40E-21	0.48188074	3.80E-12	-0.8520493	2.48E-53	-0.5333091	5.48E-15
2.52E-34	0.43903116	4.07E-10	-0.8911903	9.91E-65	-0.4308365	9.26E-10
0.02519002	-0.2635537	0.00028928	0.5528556	3.36E-16	0.30346526	2.68E-05
2.71E-20	-0.4680787	1.84E-11	0.61590557	1.05E-20	0.41203693	5.63E-09
6.89E-51	-0.4118906	5.71E-09	0.71068216	9.27E-30	0.53318575	5.57E-15
3.15E-09	-0.2729387	0.00017071	0.56536704	5.11E-17	0.66768659	3.07E-25
1.92E-16	-0.5651691	5.27E-17	0.79441995	1.74E-41	0.41286458	5.22E-09
4.04E-45	-0.5306666	7.88E-15	0.96192555	4.96E-105	0.73399092	1.42E-32
6.18E-59	-0.5529695	3.31E-16	0.83957113	2.24E-50	0.42908234	1.10E-09
1.70E-31	0.33818206	2.50E-06	-0.6288251	9.35E-22	-0.4972752	6.00E-13
1.70E-48	-0.6138323	1.54E-20	0.94840548	3.18E-93	0.61957169	5.36E-21
5.60E-32	-0.446022	1.98E-10	0.85945455	3.23E-55	0.63463868	3.03E-22
1.83E-18	-0.3029432	2.77E-05	0.74812777	1.98E-34	0.35655382	6.31E-07
4.50E-18	0.35805843	5.61E-07	-0.6362608	2.20E-22	-0.7462059	3.61E-34
5.20E-40	0.15647223	0.03342454	-0.6060519	6.21E-20	-0.4238851	1.83E-09
5.35E-08	0.35364742	7.89E-07	-0.6265383	1.45E-21	-0.5751171	1.11E-17
1.58E-21	-0.0637526	0.3886161	0.42907371	1.10E-09	0.09643987	0.19159356
0.00049838	0.39609845	2.39E-08	-0.5677148	3.56E-17	-0.0248577	0.73697512
1.12E-18	-0.4804151	4.50E-12	0.79058656	7.79E-41	0.49449977	8.43E-13
3.58E-33	-0.554496	2.64E-16	0.81829731	7.03E-46	0.6688422	2.38E-25
3.87E-12	0.30057969	3.22E-05	-0.5824547	3.42E-18	-0.5818881	3.75E-18
9.37E-11	0.42163992	2.27E-09	-0.7668171	4.43E-37	-0.4245825	1.71E-09
1.17E-38	-0.5873429	1.53E-18	0.9533648	3.84E-97	0.7313867	3.04E-32
6.13E-32	0.36825289	2.51E-07	-0.6767942	3.93E-26	-0.6097066	3.24E-20
7.50E-09	0.74455913	5.99E-34	-0.7452204	4.89E-34	-0.3693069	2.30E-07
0.00022124	0.21623642	0.00311399	-0.5392884	2.38E-15	-0.3821108	8.00E-08
1.55E-14	0.56859797	3.10E-17	-0.6996749	1.59E-28	-0.2836889	9.11E-05
8.67E-11	0.78370078	1.07E-39	-0.6062448	6.00E-20	-0.1606231	0.02895465
4.42E-21	-0.5371468	3.21E-15	0.87983208	5.10E-61	0.71314413	4.82E-30
0.00433549	-0.3474506	1.26E-06	0.44696748	1.80E-10	0.54582792	9.37E-16
7.91E-08	-0.2854587	8.20E-05	0.65480319	5.00E-24	0.57418157	1.29E-17
1.03E-18	-0.4064735	9.41E-09	0.65992315	1.68E-24	0.72831228	7.34E-32
6.08E-10	0.7555327	1.89E-35	-0.7367566	6.30E-33	-0.3891433	4.39E-08
1.06E-33	-0.563549	6.75E-17	0.89542816	3.19E-66	0.69208554	1.05E-27
6.82E-14	-0.5373119	3.14E-15	0.69094146	1.38E-27	0.25619316	0.00043168
8.38E-30	-0.3314728	4.05E-06	0.57750222	7.61E-18	0.43638365	5.32E-10
4.57E-33	0.41218351	5.56E-09	-0.7451757	4.96E-34	-0.4661909	2.27E-11
1.24E-22	-0.1459006	0.04752081	0.68109411	1.45E-26	0.2120288	0.00376302
4.25E-20	-0.3893957	4.30E-08	0.70053669	1.28E-28	0.21287438	0.00362355

2.44E-22	-0.3783336	1.10E-07	0.69359285	7.23E-28		0.32484289	6.44E-06
2.85E-16	0.30410407	2.57E-05	-0.6514316	1.02E-23		-0.8058287	1.65E-43
4.90E-35	-0.3380102	2.53E-06	0.82518847	2.87E-47		0.60467524	7.92E-20
1.71E-06	-0.6374294	1.75E-22	0.5765286	8.89E-18		0.50526153	2.22E-13
3.70E-16	0.37010548	2.16E-07	-0.6443173	4.39E-23		-0.4581896	5.45E-11
5.76E-14	-0.3776676	1.16E-07	0.67471529	6.32E-26		0.09098379	0.21806789
1.96E-39	-0.5060381	2.02E-13	0.92387826	2.91E-78		0.52676843	1.34E-14
5.13E-16	-0.4567501	6.37E-11	0.67995324	1.89E-26		0.65030849	1.28E-23
3.90E-09	0.33357518	3.48E-06	-0.6591495	1.98E-24		-0.4546543	7.98E-11
2.44E-23	-0.3569435	6.12E-07	0.75700702	1.17E-35		0.44511083	2.18E-10
3.39E-16	0.52774017	1.17E-14	-0.5879512	1.38E-18		-0.3926543	3.24E-08
0.01291985	0.17849248	0.01506309	-0.4380416	4.50E-10		0.27536471	0.00014849
1.81E-05	-0.2710994	0.00018959	0.41047272	6.51E-09		0.03984097	0.59027555
1.68E-42	0.34929196	1.10E-06	-0.8436125	2.64E-51		-0.6446612	4.10E-23
2.82E-30	0.59676399	3.13E-19	-0.8350033	2.34E-49		-0.8010778	1.19E-42
1.06E-24	0.55030177	4.89E-16	-0.8956716	2.61E-66		-0.5391223	2.43E-15
0.00115974	-0.4221504	2.16E-09	0.51263632	8.67E-14		0.3303321	4.39E-06
1.86E-18	-0.34771	1.24E-06	0.69636066	3.64E-28		0.09747367	0.18685351
8.51E-08	0.41518564	4.20E-09	-0.5713815	2.01E-17		-0.6675904	3.14E-25
6.96E-05	0.51689891	4.98E-14	-0.7082886	1.74E-29		-0.2328657	0.00142363
1.61E-67	-0.4017604	1.44E-08	0.82077511	2.26E-46		0.5736843	1.40E-17
2.39E-50	-0.533516	5.32E-15	0.8449473	1.28E-51		0.39199457	3.43E-08
3.46E-27	-0.3933378	3.05E-08	0.60334258	1.00E-19		0.17929048	0.01460997
5.29E-16	-0.4940152	8.94E-13	0.81884455	5.48E-46		0.56120419	9.65E-17
2.00E-08	0.43767829	4.67E-10	-0.7662161	5.43E-37		-0.2622217	0.00031128
1.72E-11	0.30706588	2.12E-05	-0.5720881	1.80E-17		-0.7037128	5.69E-29
5.04E-15	0.3205133	8.67E-06	-0.7154601	2.59E-30		-0.6798366	1.94E-26
7.39E-22	-0.2163687	0.00309534	0.79253044	3.66E-41		0.48719332	2.03E-12
1.35E-19	0.17537265	0.0169545	-0.4725451	1.11E-11		-0.1897198	0.00969525
6.44E-33	-0.1895488	0.00976226	0.62537601	1.81E-21		0.32613313	5.89E-06
4.79E-18	-0.4216649	2.27E-09	0.60395485	8.99E-20		0.22586992	0.00199222
4.12E-28	-0.562626	7.78E-17	0.90986115	7.88E-72		0.80130191	1.09E-42
0.17075879	0.60153667	1.37E-19	-0.4715096	1.25E-11		-0.2439426	0.00081931
2.59E-33	0.76877066	2.26E-37	-0.8430064	3.65E-51		-0.4920422	1.14E-12
1.40E-38	0.39925471	1.81E-08	-0.6877504	2.99E-27		-0.5632127	7.11E-17
7.08E-34	0.24782099	0.00067116	-0.636576	2.07E-22		-0.1628066	0.02681542
0.0004207	0.26189976	0.00031682	-0.4504443	1.25E-10		-0.4878158	1.89E-12
6.15E-06	0.25009575	0.0005962	-0.4979561	5.52E-13		-0.5199314	3.34E-14
4.25E-15	0.48116841	4.13E-12	-0.687857	2.91E-27		-0.6139176	1.52E-20
6.22E-28	-0.259901	0.00035336	0.72985238	4.72E-32		0.5594046	1.27E-16
2.55E-09	-0.3934394	3.02E-08	0.58831838	1.30E-18		0.49522664	7.72E-13
6.85E-24	-0.4899005	1.47E-12	0.85194056	2.64E-53		0.75178525	6.27E-35
3.35E-27	-0.4183975	3.10E-09	0.79407881	1.99E-41		0.38971019	4.18E-08
5.46E-17	0.36828278	2.50E-07	-0.7380171	4.34E-33		-0.3915035	3.58E-08
7.33E-25	0.3618761	4.16E-07	-0.5880732	1.36E-18		-0.4022419	1.38E-08
0.00078518	-0.4341518	6.66E-10	0.51299939	8.28E-14		0.03252862	0.66025972

4.13E-28	-0.4668417	2.11E-11	0.89727213	6.84E-67	0.70799332	1.88E-29
2.20E-25	0.30591788	2.29E-05	-0.7158239	2.35E-30	-0.403536	1.23E-08
3.09E-21	0.06336029	0.39154809	-0.4781716	5.84E-12	-0.3147161	1.28E-05
3.43E-16	-0.1201801	0.10321609	0.55372462	2.96E-16	0.3326351	3.73E-06
2.28E-19	-0.4510566	1.17E-10	0.4446299	2.29E-10	0.04764894	0.51953076
6.36E-17	-0.5485244	6.34E-16	0.73817499	4.14E-33	0.17602391	0.01654343
9.92E-25	-0.5276355	1.19E-14	0.85307413	1.38E-53	0.66805155	2.84E-25
1.53E-32	-0.4474378	1.71E-10	0.72040331	6.73E-31	0.23547442	0.00125278
4.51E-39	-0.5586687	1.42E-16	0.83343863	5.14E-49	0.45856303	5.24E-11
7.84E-43	-0.5997808	1.86E-19	0.88157098	1.46E-61	0.61749852	7.87E-21
7.94E-05	-0.4028848	1.30E-08	0.49819779	5.36E-13	0.29408227	4.84E-05
2.41E-21	-0.4121496	5.58E-09	0.70183743	9.18E-29	0.65897812	2.06E-24
3.32E-16	-0.546516	8.48E-16	0.82343241	6.56E-47	0.3549984	7.11E-07
6.19E-27	-0.621911	3.47E-21	0.81986131	3.44E-46	0.41871698	3.00E-09
1.20E-39	-0.3607703	4.54E-07	0.78713459	2.93E-40	0.27687822	0.00013602
3.87E-49	-0.5311703	7.36E-15	0.89388878	1.13E-65	0.43279493	7.63E-10
6.55E-27	-0.4425978	2.83E-10	0.90149155	1.80E-68	0.5620458	8.49E-17
3.61E-12	-0.3104357	1.70E-05	0.6649075	5.68E-25	0.08214528	0.26630864
4.77E-12	-0.5108548	1.09E-13	0.70381281	5.54E-29	0.70717716	2.32E-29
0.17960414	-0.0791206	0.28437571	0.32092419	8.43E-06	0.11092222	0.13280765
3.51E-32	-0.3539276	7.72E-07	0.65998228	1.66E-24	0.81747458	1.02E-45
5.73E-36	-0.2375777	0.00112893	0.73217931	2.41E-32	0.5499519	5.15E-16
6.71E-22	0.26111867	0.00033066	-0.6048287	7.71E-20	-0.1402888	0.05682811
8.67E-22	-0.0798284	0.2800756	0.58928514	1.11E-18	0.12316304	0.09487798
1.15E-23	-0.5409456	1.88E-15	0.71346235	4.43E-30	0.13042023	0.07681897
2.31E-06	0.61631418	9.78E-21	-0.3983515	1.96E-08	-0.228257	0.00177838
1.67E-24	0.50329529	2.85E-13	-0.8325363	8.07E-49	-0.4353609	5.90E-10
0.58770201	0.1157656	0.11660351	-0.2278478	0.00181347	-0.4763816	7.18E-12
7.96E-05	0.28384976	9.02E-05	-0.4638851	2.93E-11	-0.4963563	6.72E-13
1.19E-10	0.36904913	2.35E-07	-0.6810607	1.46E-26	-0.114365	0.12112254
3.14E-07	-0.4461484	1.96E-10	0.66183506	1.11E-24	0.55143121	4.15E-16
1.82E-22	-0.4921809	1.12E-12	0.83582631	1.54E-49	0.59101658	8.29E-19
1.43E-13	0.39149012	3.58E-08	-0.7216267	4.80E-31	-0.2636652	0.0002875
0.21715772	0.22863554	0.00174645	-0.4421777	2.95E-10	-0.4721062	1.17E-11
3.02E-35	0.46152005	3.80E-11	-0.7552915	2.04E-35	-0.6198434	5.10E-21
2.32E-40	0.58451328	2.44E-18	-0.636063	2.29E-22	-0.4307754	9.32E-10
2.96E-16	-0.4297713	1.03E-09	0.70008656	1.43E-28	0.34105218	2.03E-06
9.23E-15	0.28496013	8.44E-05	-0.5394991	2.31E-15	-0.6701387	1.78E-25
0.04806696	0.48356835	3.12E-12	-0.3248568	6.44E-06	-0.3053964	2.37E-05
1.78E-42	-0.4274823	1.29E-09	0.73038734	4.05E-32	0.37752801	1.17E-07
4.56E-14	-0.4345546	6.40E-10	0.81114211	1.69E-44	0.47897952	5.32E-12
1.07E-33	-0.601428	1.40E-19	0.90838979	3.24E-71	0.60059602	1.62E-19
1.76E-43	-0.5337994	5.12E-15	0.73652535	6.75E-33	0.37958244	9.89E-08
8.52E-22	-0.5774273	7.70E-18	0.8839351	2.58E-62	0.67686119	3.87E-26
2.27E-28	0.55292897	3.33E-16	-0.712282	6.07E-30	-0.6379843	1.57E-22
1.98E-36	-0.4839555	2.98E-12	0.82437799	4.21E-47	0.40783749	8.31E-09

2.71E-43	-0.5534291	3.09E-16	0.83264412	7.65E-49	0.34241559	1.83E-06
9.99E-59	0.58506185	2.23E-18	-0.9163481	1.15E-74	-0.5585542	1.44E-16
9.85E-09	0.23107339	0.00155309	-0.4569699	6.22E-11	-0.6662765	4.20E-25
2.01E-12	0.71852445	1.13E-30	-0.6475149	2.29E-23	-0.6494535	1.53E-23
1.24E-20	-0.472573	1.11E-11	0.85500097	4.53E-54	0.63773249	1.65E-22
7.61E-25	-0.0316393	0.66899206	-0.455484	7.30E-11	-0.237021	0.00116057
2.50E-34	-0.2416601	0.00092001	0.49948348	4.57E-13	0.1365835	0.06376173
2.93E-21	0.45063305	1.22E-10	-0.7947207	1.54E-41	-0.7440955	6.91E-34
1.07E-18	-0.4461246	1.96E-10	0.85966751	2.84E-55	0.44677069	1.83E-10
1.30E-11	0.2454688	0.00075775	-0.6027818	1.10E-19	-0.6412519	8.16E-23
6.94E-51	-0.5329175	5.78E-15	0.78601595	4.47E-40	0.50174136	3.46E-13
3.20E-43	-0.5795159	5.50E-18	0.95007125	1.70E-94	0.63421	3.29E-22
4.83E-18	-0.4943363	8.60E-13	0.74702136	2.80E-34	0.42899763	1.11E-09
4.62E-21	-0.539228	2.40E-15	0.78980858	1.05E-40	0.29269711	5.28E-05
9.65E-22	-0.2621109	0.00031318	0.62666806	1.41E-21	0.73601518	7.85E-33
2.54E-38	0.27939152	0.00011746	-0.7811276	2.77E-39	-0.4816126	3.92E-12
2.29E-17	-0.5299175	8.73E-15	0.81447665	3.91E-45	0.45524092	7.49E-11
3.20E-15	0.47839047	5.70E-12	-0.5543013	2.72E-16	-0.475359	8.07E-12
3.88E-35	-0.3054343	2.36E-05	0.652339	8.40E-24	0.1819806	0.01316907
3.93E-08	0.42810314	1.21E-09	-0.7106053	9.46E-30	-0.0546712	0.45983274
6.51E-12	-0.4993904	4.63E-13	0.62763617	1.17E-21	0.17474738	0.01735749
1.13E-38	-0.4476203	1.68E-10	0.69795976	2.44E-28	0.22041533	0.00257132
6.63E-05	0.3321071	3.87E-06	-0.6042395	8.55E-20	-0.5218344	2.60E-14
1.09E-07	-0.1462635	0.04696538	0.3804381	9.21E-08	0.01793286	0.80856353
8.49E-15	0.32457012	6.56E-06	-0.62124	3.93E-21	-0.7004647	1.30E-28
1.19E-10	0.33057369	4.31E-06	-0.5559848	2.12E-16	-0.7335388	1.62E-32
5.55E-33	0.30785113	2.02E-05	-0.5908106	8.58E-19	-0.3700595	2.17E-07
4.31E-29	0.46656978	2.17E-11	-0.8857012	6.90E-63	-0.5863971	1.79E-18
4.15E-18	0.6353568	2.63E-22	-0.6884441	2.53E-27	-0.5150049	6.38E-14
2.22E-13	0.47075275	1.36E-11	-0.6170927	8.48E-21	-0.7098458	1.16E-29
7.89E-11	0.40652092	9.37E-09	-0.536133	3.70E-15	-0.0815742	0.2696584
9.88E-55	-0.2039089	0.00536973	0.70475682	4.35E-29	0.36231918	4.02E-07
1.32E-31	-0.3494042	1.09E-06	0.71309641	4.88E-30	0.58740264	1.51E-18
6.65E-42	-0.5315224	7.01E-15	0.86306879	3.54E-56	0.72518924	1.78E-31
3.74E-19	-0.5175051	4.60E-14	0.82110758	1.94E-46	0.7928033	3.29E-41
1.92E-09	-0.3084834	1.94E-05	0.53938448	2.35E-15	0.52778765	1.17E-14
2.65E-39	-0.4013022	1.50E-08	0.56966193	2.63E-17	0.31044594	1.70E-05
0.1104741	0.23006421	0.00163063	-0.4716923	1.22E-11	0.16690551	0.02316361
3.71E-35	0.29588453	4.33E-05	-0.7792315	5.54E-39	-0.4230759	1.98E-09
2.11E-10	-0.5307691	7.77E-15	0.78824069	1.92E-40	0.53595389	3.80E-15
4.46E-11	0.18297912	0.01266686	-0.4867415	2.14E-12	-0.6898557	1.80E-27
7.12E-32	0.57305549	1.54E-17	-0.7686216	2.38E-37	-0.5950975	4.16E-19
4.47E-11	0.48201021	3.74E-12	-0.7825833	1.62E-39	-0.4004231	1.63E-08
1.27E-16	0.40658348	9.32E-09	-0.6878649	2.91E-27	-0.7179627	1.31E-30
7.92E-18	-0.4479743	1.62E-10	0.82909792	4.38E-48	0.73150879	2.93E-32
1.08E-43	-0.5434608	1.32E-15	0.7775164	1.03E-38	0.53001672	8.62E-15

4.24E-33	-0.5317368	6.81E-15	0.90364814	2.62E-69	0.79088394	6.95E-41
3.96E-25	-0.4645321	2.73E-11	0.8168352	1.36E-45	0.2898029	6.30E-05
2.07E-13	-0.498832	4.96E-13	0.63378609	3.58E-22	0.56261793	7.79E-17
5.36E-31	-0.3904492	3.92E-08	0.75256273	4.90E-35	0.18266372	0.01282364
9.28E-48	-0.4682313	1.81E-11	0.73398411	1.43E-32	0.36816976	2.52E-07
7.97E-13	0.34994101	1.05E-06	-0.7493689	1.35E-34	-0.0831515	0.26047587
3.65E-10	-0.5778776	7.16E-18	0.78909797	1.38E-40	0.50230419	3.22E-13
8.22E-47	-0.5799768	5.11E-18	0.90809529	4.28E-71	0.52912755	9.73E-15
1.79E-16	0.441501	3.16E-10	-0.621999	3.41E-21	-0.0717285	0.3319215
2.05E-13	0.55698453	1.82E-16	-0.546338	8.70E-16	-0.2495039	0.00061492
2.27E-47	-0.5399188	2.18E-15	0.97260301	6.73E-118	0.75693275	1.20E-35
5.93E-11	-0.3048711	2.45E-05	0.59466353	4.48E-19	0.6829279	9.42E-27
2.95E-22	0.47732791	6.44E-12	-0.7535665	3.55E-35	-0.6710533	1.45E-25
4.08E-35	0.46193652	3.63E-11	-0.7802864	3.77E-39	-0.2702015	0.00019949
9.91E-36	0.7337128	1.54E-32	-0.8899644	2.61E-64	-0.729798	4.80E-32
8.07E-07	-0.3157872	1.19E-05	0.51548667	5.99E-14	0.30647277	2.21E-05
3.82E-26	-0.4618433	3.66E-11	0.73613655	7.57E-33	0.79242023	3.82E-41
2.68E-20	-0.4919515	1.15E-12	0.67871524	2.52E-26	0.3123558	1.50E-05
1.07E-13	0.70040145	1.32E-28	-0.6092468	3.52E-20	-0.2032102	0.00553327
1.88E-27	0.41044258	6.53E-09	-0.8008619	1.30E-42	-0.7996502	2.13E-42
1.99E-27	0.68060158	1.63E-26	-0.5594479	1.26E-16	-0.4678144	1.89E-11
3.26E-19	-0.3150551	1.25E-05	0.63906842	1.26E-22	0.09632473	0.19212687
2.66E-07	0.33453937	3.25E-06	-0.5128642	8.42E-14	-0.6341347	3.34E-22
3.06E-19	0.3103306	1.72E-05	-0.6733665	8.59E-26	-0.6654062	5.09E-25
5.23E-05	-0.152039	0.03883194	0.46282767	3.29E-11	0.18667745	0.0109502
4.71E-10	0.3823033	7.87E-08	-0.657779	2.66E-24	-0.4613247	3.88E-11
9.82E-48	-0.5637575	6.54E-17	0.88079795	2.55E-61	0.44247078	2.86E-10
1.94E-26	-0.3010676	3.12E-05	0.81013485	2.62E-44	0.56638761	4.37E-17
1.56E-05	-0.3710879	1.99E-07	0.48843184	1.75E-12	0.20930515	0.00424574
1.19E-11	-0.4532228	9.29E-11	0.72038305	6.77E-31	0.74765906	2.30E-34
2.88E-10	0.75608338	1.58E-35	-0.6992879	1.75E-28	-0.6950908	4.99E-28
7.09E-29	0.59251305	6.44E-19	-0.8768796	4.08E-60	-0.4877239	1.91E-12
4.36E-09	-0.1482219	0.04406136	0.43557747	5.77E-10	-0.1401589	0.05706026
8.87E-31	-0.3641161	3.49E-07	0.68694769	3.63E-27	0.49033792	1.39E-12
3.40E-41	-0.5347109	4.51E-15	0.95794796	3.67E-101	0.68040551	1.70E-26
5.29E-69	0.3991445	1.82E-08	-0.7308192	3.58E-32	-0.4744113	8.99E-12
7.15E-21	0.55148278	4.12E-16	-0.677364	3.45E-26	-0.3795137	9.95E-08
4.74E-06	-0.3981197	2.00E-08	0.66272444	9.15E-25	0.43926585	3.97E-10
2.13E-37	-0.5365247	3.51E-15	0.88796988	1.23E-63	0.69223072	1.01E-27
0.00143615	0.21115647	0.00391195	-0.4444136	2.34E-10	-0.0344172	0.64186733
8.84E-10	-0.3753658	1.40E-07	0.61369028	1.58E-20	0.80335544	4.65E-43
0.00021382	0.38428003	6.66E-08	-0.6139629	1.50E-20	-0.2832075	9.38E-05
9.00E-08	0.30565481	2.33E-05	-0.660074	1.63E-24	-0.4402537	3.59E-10
1.13E-08	0.14754081	0.04505377	-0.5353533	4.13E-15	-0.5194166	3.58E-14
2.30E-15	-0.5866188	1.72E-18	0.78928571	1.29E-40	0.48195234	3.77E-12
1.45E-10	-0.2519994	0.00053948	0.65211652	8.80E-24	0.35966175	4.95E-07

2.37E-35	0.74162339	1.47E-33	-0.6499783	1.37E-23	-0.5401187	2.12E-15
0.00104778	-0.5025209	3.14E-13	0.64129117	8.09E-23	0.42334461	1.93E-09
2.46E-23	-0.6008243	1.55E-19	0.80980683	3.02E-44	0.33418522	3.34E-06
3.72E-09	0.24281898	0.00086754	-0.5877853	1.42E-18	-0.4974905	5.85E-13
6.82E-17	0.66970695	1.96E-25	-0.5187519	3.91E-14	-0.5579841	1.57E-16
2.39E-37	0.64825484	1.96E-23	-0.730027	4.49E-32	-0.5917722	7.30E-19
6.06E-13	-0.4728839	1.07E-11	0.76392173	1.18E-36	0.73515702	1.01E-32
8.18E-24	-0.3894569	4.27E-08	0.69665108	3.39E-28	0.84017097	1.64E-50
1.39E-10	0.49858356	5.11E-13	-0.7813755	2.53E-39	-0.4130339	5.13E-09
4.01E-07	-0.3875478	5.04E-08	0.67709671	3.66E-26	0.68817744	2.70E-27
2.13E-10	0.30249956	2.85E-05	-0.6586774	2.19E-24	-0.6626823	9.24E-25
2.65E-49	0.74267076	1.07E-33	-0.7415868	1.48E-33	-0.5045568	2.43E-13
2.90E-41	-0.5999232	1.82E-19	0.90940071	1.23E-71	0.7816893	2.25E-39
1.73E-25	0.41081389	6.31E-09	-0.7808979	3.01E-39	-0.5676103	3.62E-17
5.16E-14	-0.4930019	1.01E-12	0.80908924	4.12E-44	0.69774183	2.58E-28
3.13E-18	0.39269939	3.22E-08	-0.6526908	7.81E-24	-0.3630779	3.79E-07
0.00043494	-0.4939817	8.98E-13	0.49404411	8.91E-13	0.66435908	6.40E-25
3.01E-34	0.43340962	7.17E-10	-0.7451504	5.00E-34	-0.5355222	4.03E-15
3.52E-33	-0.5260792	1.47E-14	0.94070631	7.50E-88	0.6944796	5.81E-28
7.08E-21	-0.5337148	5.18E-15	0.87896378	9.46E-61	0.69891669	1.92E-28
3.07E-20	-0.5220051	2.54E-14	0.82992453	2.93E-48	0.50472812	2.38E-13
9.92E-08	-0.4460058	1.99E-10	0.68974434	1.85E-27	0.65046635	1.24E-23
2.62E-08	-0.3485248	1.16E-06	0.67218195	1.12E-25	0.41248974	5.40E-09
1.98E-12	-0.4265494	1.41E-09	0.79664935	7.17E-42	0.43848167	4.30E-10
5.58E-36	-0.5084823	1.48E-13	0.93558255	1.16E-84	0.62435071	2.19E-21
2.87E-08	0.50261634	3.10E-13	-0.6074872	4.82E-20	-0.0062467	0.93274697
1.32E-38	-0.3919018	3.46E-08	0.73445387	1.24E-32	0.4147632	4.37E-09
0.02449979	0.38364757	7.03E-08	-0.549011	5.91E-16	-0.3475593	1.25E-06
2.35E-31	0.57535668	1.07E-17	-0.5730881	1.53E-17	-0.471126	1.31E-11
1.74E-24	-0.568366	3.22E-17	0.79725718	5.62E-42	0.66042737	1.51E-24
1.12E-42	0.73556711	8.96E-33	-0.8665495	3.97E-57	-0.5578345	1.60E-16
1.06E-32	0.29993611	3.36E-05	-0.7708509	1.10E-37	-0.5061324	1.99E-13
6.02E-30	0.39355054	2.99E-08	-0.6828802	9.53E-27	-0.6260846	1.58E-21
0.01834905	0.02134664	0.77303237	-0.2621702	0.00031216	-0.5318937	6.66E-15
1.52E-23	-0.4004303	1.63E-08	0.88096564	2.26E-61	0.68247412	1.05E-26
0.09176839	0.36935765	2.29E-07	-0.2704762	0.00019641	-0.356365	6.40E-07
3.49E-09	0.36459819	3.36E-07	-0.4996728	4.47E-13	-0.6647682	5.85E-25
0.00011039	-0.1825082	0.0129016	0.36936182	2.29E-07	0.33913704	2.33E-06
1.17E-08	-0.3889375	4.47E-08	0.60642415	5.82E-20	0.46259319	3.37E-11
4.53E-58	0.50789833	1.59E-13	-0.9160523	1.57E-74	-0.7047536	4.35E-29
0.01594086	0.15512078	0.03500079	-0.3607923	4.53E-07	-0.3106566	1.68E-05
7.83E-39	-0.4934761	9.55E-13	0.78160883	2.32E-39	0.34348785	1.70E-06
1.22E-33	-0.4415497	3.15E-10	0.73433997	1.28E-32	0.29373512	4.95E-05
1.47E-49	-0.4304656	9.61E-10	0.84165102	7.50E-51	0.40955413	7.09E-09
1.51E-37	0.37557407	1.38E-07	-0.6231843	2.73E-21	-0.306297	2.23E-05
3.87E-06	-0.3408506	2.06E-06	0.59353101	5.43E-19	0.2812874	0.00010506

1.62E-27	0.19879234	0.00667436	-0.538669	2.60E-15	-0.2264183	0.00194112
1.96E-34	-0.3389244	2.37E-06	0.8063547	1.32E-43	0.30145521	3.05E-05
9.68E-18	-0.4209452	2.43E-09	0.76746192	3.55E-37	0.32499714	6.37E-06
3.91E-14	-0.5948022	4.37E-19	0.59954396	1.94E-19	0.33193239	3.92E-06
4.53E-25	0.42623905	1.46E-09	-0.7601246	4.20E-36	-0.4661612	2.28E-11
2.41E-49	-0.5331284	5.62E-15	0.83586183	1.51E-49	0.38378232	6.95E-08
2.32E-24	0.43635403	5.34E-10	-0.7713089	9.37E-38	-0.5014309	3.59E-13
4.49E-29	-0.5753614	1.07E-17	0.78606845	4.39E-40	0.8732283	4.98E-59
2.15E-17	-0.487972	1.85E-12	0.81012865	2.63E-44	0.76336899	1.42E-36
0.00065393	0.43470823	6.30E-10	-0.6012583	1.44E-19	-0.3613388	4.34E-07
1.57E-42	0.21688008	0.00302417	-0.69009	1.70E-27	-0.5864206	1.78E-18
9.51E-10	-0.5609171	1.01E-16	0.76563972	6.61E-37	0.5572991	1.74E-16
5.04E-10	-0.4166929	3.64E-09	0.69630596	3.69E-28	0.60851417	4.01E-20
2.77E-20	0.11085908	0.13302974	-0.3684982	2.46E-07	-0.0200982	0.7859755
3.97E-27	0.41602001	3.88E-09	-0.6070443	5.21E-20	-0.5032053	2.88E-13
2.62E-27	-0.4688847	1.68E-11	0.8601618	2.11E-55	0.52236284	2.42E-14
3.97E-30	0.37289166	1.72E-07	-0.6824995	1.04E-26	-0.7956462	1.07E-41
3.05E-11	-0.1952641	0.00773172	0.50025142	4.16E-13	0.65850797	2.28E-24
4.20E-24	0.55039132	4.83E-16	-0.7465491	3.24E-34	-0.3390818	2.34E-06
5.12E-31	-0.4454255	2.11E-10	0.7409782	1.78E-33	0.63099967	6.15E-22
3.25E-11	-0.2567895	0.00041809	0.45376711	8.77E-11	0.24329859	0.00084665
6.64E-22	0.36310741	3.78E-07	-0.6740878	7.30E-26	-0.6981328	2.34E-28
9.50E-40	-0.6620631	1.06E-24	0.9481121	5.26E-93	0.70396893	5.32E-29
3.63E-25	-0.4653379	2.49E-11	0.87685148	4.16E-60	0.71015712	1.06E-29
0.03840085	0.45137209	1.13E-10	-0.395776	2.46E-08	-0.5598286	1.19E-16
4.84E-27	0.37204679	1.84E-07	-0.6825369	1.03E-26	-0.7501903	1.04E-34
8.65E-32	-0.4894943	1.54E-12	0.84192579	6.49E-51	0.40292099	1.30E-08
0.00016075	0.46321168	3.15E-11	-0.6746093	6.48E-26	-0.4184873	3.07E-09
0.00016211	-0.3016932	3.00E-05	0.56746562	3.70E-17	0.68228144	1.10E-26
1.76E-10	-0.4034427	1.24E-08	0.75824775	7.80E-36	0.46997101	1.49E-11
1.46E-10	-0.4995334	4.55E-13	0.6415895	7.62E-23	0.48251685	3.52E-12
2.33E-21	0.31898062	9.63E-06	-0.7239911	2.49E-31	-0.1177923	0.11029829
5.00E-27	0.38729189	5.15E-08	-0.7636325	1.30E-36	-0.5836065	2.83E-18
4.00E-19	0.28124387	0.00010533	-0.579871	5.20E-18	-0.4624108	3.44E-11
1.91E-16	0.26391667	0.00028354	-0.5418382	1.66E-15	-0.715609	2.49E-30
1.02E-33	-0.3152297	1.24E-05	0.82546449	2.51E-47	0.65741171	2.88E-24
9.81E-15	-0.5016614	3.49E-13	0.81607981	1.91E-45	0.6079944	4.40E-20
3.60E-14	0.34172135	1.93E-06	-0.6866773	3.87E-27	-0.4072724	8.75E-09
5.18E-30	-0.509836	1.24E-13	0.9274602	4.18E-80	0.71580841	2.36E-30
2.23E-09	-0.534338	4.75E-15	0.64898501	1.69E-23	0.31956854	9.25E-06
5.19E-40	-0.5526229	3.48E-16	0.84490858	1.31E-51	0.34982845	1.06E-06
3.03E-13	-0.3946075	2.73E-08	0.77892205	6.20E-39	0.74963274	1.24E-34
3.67E-14	-0.5214038	2.75E-14	0.79535122	1.20E-41	0.45529061	7.45E-11
0.00040014	-0.4369395	5.03E-10	0.57217556	1.77E-17	0.36898306	2.36E-07
4.41E-05	-0.4425197	2.85E-10	0.5999243	1.82E-19	0.70098004	1.14E-28
4.54E-33	-0.5688612	2.98E-17	0.89603593	1.93E-66	0.5763134	9.20E-18

3.83E-21	-0.3709363	2.02E-07	0.5883489	1.29E-18	0.27450542	0.00015603
1.58E-06	-0.3870198	5.27E-08	0.63765516	1.67E-22	0.52482989	1.74E-14
1.55E-08	0.14284872	0.05241091	-0.2953375	4.48E-05	0.28075632	0.00010841
5.45E-48	-0.5618219	8.79E-17	0.89282775	2.67E-65	0.79946838	2.30E-42
0.00097565	-0.3639033	3.55E-07	0.48427005	2.87E-12	0.74216398	1.25E-33
5.51E-13	0.36766024	2.63E-07	-0.6213168	3.88E-21	-0.5786137	6.36E-18
2.76E-30	-0.4926323	1.06E-12	0.83868313	3.55E-50	0.86990811	4.52E-58
6.52E-48	-0.4993367	4.66E-13	0.88235683	8.24E-62	0.5757779	1.00E-17
5.21E-40	-0.5408003	1.92E-15	0.90183318	1.33E-68	0.7430626	9.47E-34
4.76E-63	0.48353415	3.13E-12	-0.8954508	3.13E-66	-0.6218282	3.52E-21
1.19E-16	-0.5276986	1.18E-14	0.76279338	1.73E-36	0.38852261	4.63E-08
6.94E-19	-0.472328	1.14E-11	0.84047015	1.40E-50	0.56628823	4.44E-17
9.29E-41	-0.4981852	5.37E-13	0.94320988	1.62E-89	0.7699912	1.48E-37
9.10E-34	-0.4312583	8.88E-10	0.72852194	6.91E-32	0.29625043	4.23E-05
9.54E-08	0.14715548	0.0456235	-0.3704314	2.10E-07	-0.3552088	7.00E-07
7.34E-08	-0.375467	1.39E-07	0.65118835	1.07E-23	0.45379948	8.74E-11
5.45E-14	-0.5914198	7.75E-19	0.77252639	6.11E-38	0.74994895	1.12E-34
0.00239021	-0.3130568	1.43E-05	0.44815275	1.59E-10	0.70821672	1.77E-29
2.01E-15	-0.4794689	5.03E-12	0.86352487	2.67E-56	0.64968311	1.46E-23
7.23E-26	0.26935707	0.00020925	-0.4802375	4.60E-12	-0.1400364	0.05727979
4.83E-06	-0.3577064	5.77E-07	0.56188324	8.71E-17	0.48936447	1.57E-12
1.78E-35	0.46521089	2.53E-11	-0.7618634	2.36E-36	-0.4627483	3.32E-11
3.67E-12	-0.3301542	4.44E-06	0.77466131	2.87E-38	0.65421541	5.67E-24
1.78E-14	0.26767656	0.00022999	-0.5089455	1.39E-13	-0.5029358	2.98E-13
7.67E-43	-0.4861982	2.28E-12	0.8649181	1.12E-56	0.39819479	1.99E-08
2.23E-10	-0.5263515	1.42E-14	0.73770997	4.75E-33	0.67123099	1.39E-25
4.08E-16	-0.4339664	6.78E-10	0.77117137	9.83E-38	0.30059416	3.22E-05
5.16E-11	-0.4141874	4.61E-09	0.71569713	2.43E-30	0.35651728	6.33E-07
3.44E-21	0.36444521	3.40E-07	-0.7338358	1.49E-32	-0.3771347	1.21E-07
7.88E-09	-0.2716063	0.0001842	0.54971958	5.33E-16	-0.0244607	0.74102476
3.16E-39	-0.3760655	1.33E-07	0.82208421	1.23E-46	0.48742047	1.98E-12
1.86E-33	-0.4764876	7.09E-12	0.86461321	1.35E-56	0.34631067	1.37E-06
1.67E-20	-0.438641	4.23E-10	0.82196437	1.30E-46	0.30644399	2.21E-05
1.09E-21	-0.39568	2.48E-08	0.82746193	9.67E-48	0.50079648	3.89E-13
1.30E-32	-0.3259817	5.95E-06	0.63559187	2.51E-22	0.34169997	1.93E-06
7.95E-26	0.40618229	9.67E-09	-0.7165651	1.92E-30	-0.5651091	5.32E-17
5.23E-13	-0.2979719	3.80E-05	0.70755732	2.10E-29	0.37118007	1.98E-07
3.30E-43	-0.5909626	8.37E-19	0.92403203	2.44E-78	0.53400152	4.98E-15
3.71E-16	0.39271195	3.22E-08	-0.5325533	6.08E-15	-0.2096783	0.00417647
5.87E-27	-0.6421325	6.83E-23	0.92649521	1.34E-79	0.66125154	1.26E-24
5.65E-35	-0.4856769	2.43E-12	0.82788422	7.89E-48	0.34834928	1.18E-06
1.49E-48	0.55733211	1.73E-16	-0.7955199	1.12E-41	-0.376165	1.31E-07
4.76E-33	-0.3668448	2.81E-07	0.57631817	9.19E-18	0.20249235	0.00570589
4.73E-07	0.40641702	9.46E-09	-0.6765098	4.19E-26	-0.2779591	0.00012773
5.95E-21	0.41060095	6.44E-09	-0.8253875	2.61E-47	-0.5825943	3.34E-18
5.32E-11	-0.5139103	7.36E-14	0.74022418	2.24E-33	0.5667312	4.14E-17

9.43E-59	-0.493038	1.01E-12	0.72350957	2.85E-31	0.40144314	1.49E-08
0.02032807	0.29177009	5.59E-05	-0.5177816	4.44E-14	-0.3940284	2.87E-08
3.20E-25	-0.4731958	1.03E-11	0.64348737	5.20E-23	0.35369412	7.86E-07
4.31E-16	-0.3055605	2.34E-05	0.73535429	9.54E-33	0.34209096	1.88E-06
8.97E-08	-0.4418632	3.05E-10	0.56367384	6.63E-17	0.69140143	1.24E-27
5.03E-09	0.63779466	1.63E-22	-0.7809577	2.95E-39	-0.4503203	1.26E-10
1.48E-13	0.27301942	0.00016992	-0.6758975	4.83E-26	-0.5916081	7.51E-19
9.11E-30	-0.5075079	1.67E-13	0.80098276	1.24E-42	0.4309763	9.14E-10
1.33E-31	0.49898606	4.86E-13	-0.803954	3.62E-43	-0.5367474	3.40E-15
5.58E-21	0.39919494	1.82E-08	-0.779794	4.51E-39	-0.4199164	2.68E-09
0.00242561	-0.223796	0.00219673	0.26783872	0.00022791	-0.1291139	0.07984691
0.00589785	-0.3001508	3.31E-05	0.50000319	4.29E-13	0.48579848	2.40E-12
0.00078472	-0.3308445	4.23E-06	0.38812386	4.79E-08	0.33652382	2.82E-06
7.14E-17	-0.4779229	6.01E-12	0.71613584	2.16E-30	0.54687169	8.06E-16
0.55578347	-0.164487	0.02526263	0.4127288	5.28E-09	0.3007894	3.18E-05
1.88E-51	0.60439719	8.32E-20	-0.8924549	3.61E-65	-0.701518	9.96E-29
1.19E-35	0.536605	3.47E-15	-0.8263689	1.63E-47	-0.6032139	1.02E-19
9.07E-40	-0.6141448	1.45E-20	0.85697367	1.42E-54	0.748269	1.90E-34
1.19E-30	-0.2077255	0.00455058	0.82822866	6.68E-48	0.61314881	1.74E-20
4.96E-25	-0.4290039	1.11E-09	0.73041866	4.01E-32	0.57058801	2.27E-17
2.43E-41	-0.4681874	1.82E-11	0.86532049	8.65E-57	0.50909584	1.37E-13
1.31E-09	-0.3832941	7.24E-08	0.44741932	1.71E-10	-0.0443465	0.54891621
0.0005316	0.22284616	0.00229662	-0.4756384	7.82E-12	-0.3353271	3.07E-06
1.37E-12	-0.5272576	1.25E-14	0.83214572	9.80E-49	0.6997548	1.56E-28
6.17E-16	-0.4617554	3.70E-11	0.7172699	1.59E-30	0.33443526	3.28E-06
4.67E-10	-0.3656898	3.08E-07	0.80614455	1.44E-43	0.41663594	3.66E-09
3.65E-18	0.30978878	1.78E-05	-0.6943433	6.01E-28	-0.7196284	8.33E-31
0.01311081	-0.3787771	1.06E-07	0.58437682	2.50E-18	0.60474194	7.83E-20
1.76E-06	0.34497092	1.52E-06	-0.6254536	1.78E-21	-0.3902271	4.00E-08
1.56E-23	-0.5106356	1.12E-13	0.89373615	1.28E-65	0.67285547	9.65E-26
1.62E-25	0.49653819	6.57E-13	-0.6950722	5.02E-28	-0.6033146	1.01E-19
5.26E-21	-0.3280642	5.15E-06	0.65751381	2.81E-24	0.07656582	0.30026246
1.63E-47	-0.4373765	4.81E-10	0.86142822	9.73E-56	0.37789607	1.14E-07
5.08E-25	-0.4269487	1.36E-09	0.87447195	2.14E-59	0.63629701	2.19E-22
6.66E-37	0.20540953	0.00503309	-0.6202732	4.71E-21	-0.3160206	1.18E-05
9.49E-51	-0.5703435	2.36E-17	0.95088599	3.92E-95	0.60660118	5.64E-20
1.77E-32	-0.4658077	2.37E-11	0.64885501	1.73E-23	0.19136654	0.0090705
3.51E-06	-0.4825397	3.52E-12	0.6155553	1.12E-20	0.66891735	2.34E-25
7.73E-33	0.45536968	7.39E-11	-0.6673047	3.35E-25	-0.2406594	0.00096764
4.89E-05	-0.3874818	5.07E-08	0.51830334	4.14E-14	0.77475771	2.77E-38
1.50E-18	-0.5420776	1.60E-15	0.84175497	7.10E-51	0.70264859	7.47E-29
1.57E-16	0.26384262	0.0002847	-0.6492023	1.61E-23	-0.0004902	0.99471602
7.10E-18	-0.5945509	4.56E-19	0.80272574	6.04E-43	0.62493854	1.96E-21
1.42E-34	-0.3751551	1.43E-07	0.66846634	2.59E-25	0.71475871	3.13E-30
1.16E-24	-0.4993994	4.62E-13	0.70880577	1.52E-29	0.83046661	2.24E-48
1.85E-32	-0.4931113	9.98E-13	0.92982686	2.24E-81	0.4936811	9.31E-13

6.17E-08	0.47891541	5.36E-12	-0.5607531	1.03E-16	-0.3293286	4.71E-06
1.85E-31	-0.3442354	1.60E-06	0.71480196	3.09E-30	0.24269611	0.00087297
7.65E-43	-0.4979673	5.51E-13	0.8101006	2.66E-44	0.3192836	9.43E-06
5.66E-38	-0.5051952	2.24E-13	0.59136123	7.82E-19	0.34943362	1.09E-06
1.18E-28	0.38544423	6.03E-08	-0.7949132	1.43E-41	-0.4193041	2.84E-09
2.79E-21	0.37781903	1.15E-07	-0.6896309	1.90E-27	-0.7267017	1.16E-31
7.52E-05	-0.2440037	0.00081676	0.53804994	2.83E-15	0.09051276	0.22047008
4.70E-32	-0.5320665	6.50E-15	0.83383946	4.21E-49	0.46810135	1.83E-11
4.26E-35	-0.5860338	1.90E-18	0.88173019	1.30E-61	0.43707539	4.96E-10
2.45E-46	-0.5354664	4.06E-15	0.93733161	1.02E-85	0.60129039	1.43E-19
5.71E-07	0.55021467	4.96E-16	-0.6899441	1.76E-27	-0.3390533	2.35E-06
0.00417526	-0.4417978	3.07E-10	0.54963939	5.39E-16	0.22821496	0.00178195
7.13E-15	-0.2895228	6.41E-05	0.69744073	2.78E-28	0.36441679	3.41E-07
0.03720149	0.66367466	7.44E-25	-0.4237148	1.86E-09	0.14053311	0.05639368
6.17E-10	-0.3405891	2.10E-06	0.36459883	3.36E-07	0.26665538	0.00024352
5.05E-13	-0.598037	2.51E-19	0.76909651	2.02E-37	0.57585733	9.89E-18
3.59E-30	-0.5442318	1.18E-15	0.92553953	4.18E-79	0.53242841	6.19E-15
8.65E-33	0.53985514	2.20E-15	-0.8585692	5.50E-55	-0.7261015	1.37E-31
8.97E-44	-0.5630701	7.27E-17	0.94677295	5.09E-92	0.73940772	2.86E-33
1.28E-94	-0.491255	1.25E-12	0.91645232	1.03E-74	0.64533232	3.57E-23
8.88E-38	-0.5076638	1.64E-13	0.93934629	5.61E-87	0.76844715	2.53E-37
3.67E-34	-0.5386145	2.62E-15	0.94015677	1.70E-87	0.76801718	2.93E-37
1.71E-18	0.2328459	0.00142501	-0.5361582	3.69E-15	-0.7817562	2.19E-39
6.13E-35	-0.5565417	1.95E-16	0.88430117	1.97E-62	0.59585202	3.66E-19
1.92E-34	-0.4667731	2.13E-11	0.74816308	1.96E-34	0.44399272	2.45E-10
2.33E-15	-0.480479	4.47E-12	0.7871121	2.95E-40	0.59475581	4.41E-19
9.03E-32	-0.3076644	2.04E-05	0.76655817	4.84E-37	0.39677787	2.25E-08
7.64E-31	0.36041478	4.67E-07	-0.7359486	8.01E-33	-0.682033	1.16E-26
8.90E-24	0.38263154	7.66E-08	-0.7438171	7.52E-34	-0.6523255	8.43E-24
0.00322734	0.21131632	0.00388427	-0.5417978	1.67E-15	-0.2298289	0.00164922
3.39E-20	0.29316799	5.13E-05	-0.6652667	5.25E-25	-0.7559338	1.66E-35
3.46E-05	-0.3088993	1.88E-05	0.66494576	5.63E-25	0.54009535	2.12E-15
3.33E-13	-0.4589981	5.00E-11	0.78402131	9.46E-40	0.33990509	2.21E-06
1.45E-12	0.40764643	8.45E-09	-0.7489396	1.54E-34	-0.4585979	5.22E-11
4.91E-32	-0.5440865	1.20E-15	0.87518372	1.32E-59	0.42967267	1.04E-09
3.42E-14	-0.4174061	3.40E-09	0.73633463	7.14E-33	0.24067124	0.00096707
1.56E-10	0.71932403	9.06E-31	-0.6886685	2.40E-27	-0.382005	8.07E-08
1.52E-11	0.42617953	1.46E-09	-0.7683671	2.60E-37	-0.3773787	1.19E-07
2.58E-06	0.59263298	6.32E-19	-0.6113464	2.41E-20	-0.5514769	4.12E-16
6.31E-08	-0.3815149	8.41E-08	0.7057133	3.40E-29	0.57623751	9.31E-18
3.43E-42	0.34599397	1.41E-06	-0.6353417	2.64E-22	-0.4020154	1.41E-08
3.26E-17	-0.3363786	2.85E-06	0.64303181	5.70E-23	0.03526056	0.63372312
4.56E-40	0.31487889	1.27E-05	-0.7960044	9.27E-42	-0.5058924	2.05E-13
3.75E-26	0.62731308	1.25E-21	-0.7710381	1.03E-37	-0.7039407	5.36E-29
1.73E-15	0.54542268	9.93E-16	-0.6994863	1.67E-28	-0.7772023	1.16E-38
3.36E-30	-0.498388	5.24E-13	0.86866411	1.02E-57	0.83950018	2.32E-50

2.87E-22	0.55269868	3.44E-16	-0.6758431	4.89E-26	-0.4977995	5.63E-13
6.43E-23	0.24673812	0.00070983	-0.6966961	3.35E-28	-0.2319438	0.00148894
8.45E-23	0.418539	3.06E-09	-0.6634942	7.74E-25	-0.4493497	1.40E-10
1.06E-11	-0.4046848	1.11E-08	0.60129798	1.43E-19	0.83540743	1.91E-49
5.50E-18	0.37687392	1.24E-07	-0.5508586	4.51E-16	-0.4476478	1.67E-10
0.00019523	-0.3102266	1.73E-05	0.58409571	2.61E-18	0.60243955	1.17E-19
5.89E-05	0.23225244	0.00146678	-0.4708676	1.34E-11	-0.5315367	6.99E-15
2.04E-20	0.43393104	6.81E-10	-0.6431353	5.58E-23	-0.5050517	2.28E-13
2.29E-08	-0.4440087	2.44E-10	0.66951456	2.05E-25	0.68410695	7.13E-27
2.73E-28	-0.6271545	1.29E-21	0.92474228	1.07E-78	0.54871295	6.17E-16
1.19E-29	-0.4892405	1.59E-12	0.861774	7.87E-56	0.63972514	1.11E-22
1.96E-06	-0.2084866	0.00440132	0.63917151	1.24E-22	0.324161	6.75E-06
3.00E-17	-0.6078308	4.53E-20	0.85541788	3.55E-54	0.5867184	1.70E-18
5.46E-50	-0.5026226	3.10E-13	0.84873376	1.61E-52	0.40162729	1.46E-08
5.10E-08	0.5417929	1.67E-15	-0.5787932	6.18E-18	-0.4867228	2.15E-12
2.40E-39	0.54389689	1.24E-15	-0.7992921	2.47E-42	-0.4330297	7.45E-10
0.0004552	-0.3826022	7.68E-08	0.64818008	1.99E-23	0.4961706	6.88E-13
4.18E-52	-0.5306749	7.87E-15	0.93392971	1.09E-83	0.5484965	6.37E-16
3.68E-12	0.34626984	1.38E-06	-0.4585624	5.24E-11	0.14702343	0.04582011
2.88E-54	0.39527137	2.57E-08	-0.6728581	9.64E-26	-0.3472596	1.28E-06
4.98E-15	0.44068676	3.44E-10	-0.6178682	7.35E-21	-0.691128	1.32E-27
6.49E-34	-0.5504226	4.81E-16	0.90250665	7.31E-69	0.58004421	5.05E-18
7.50E-62	-0.495517	7.45E-13	0.92101524	7.48E-77	0.71627096	2.08E-30
2.24E-05	-0.4566649	6.43E-11	0.61630899	9.79E-21	0.56168578	8.97E-17
0.00143752	-0.4148628	4.33E-09	0.58482193	2.32E-18	0.55726929	1.75E-16
1.53E-08	-0.3988185	1.88E-08	0.74043691	2.10E-33	0.628705	9.57E-22
1.44E-46	-0.5649866	5.42E-17	0.94260667	4.15E-89	0.65170793	9.59E-24
4.16E-61	-0.5615319	9.18E-17	0.92222001	1.94E-77	0.80867597	4.92E-44
8.35E-16	-0.4277791	1.25E-09	0.8080699	6.37E-44	0.75344535	3.69E-35
2.87E-35	-0.5795057	5.51E-18	0.94727342	2.20E-92	0.69673574	3.32E-28
7.09E-16	-0.5016473	3.50E-13	0.79975302	2.05E-42	0.71058488	9.51E-30
1.45E-25	-0.4839725	2.97E-12	0.87849748	1.31E-60	0.81575082	2.22E-45
0.73983219	-0.1837783	0.01227709	0.27190393	0.0001811	0.27319243	0.00016825
5.60E-19	0.51183701	9.62E-14	-0.7729389	5.28E-38	-0.8101443	2.61E-44
4.74E-71	-0.5470443	7.86E-16	0.91437738	8.83E-74	0.54314232	1.38E-15
1.24E-71	-0.4678766	1.88E-11	0.74155282	1.50E-33	0.44853599	1.53E-10
3.63E-36	-0.6180986	7.04E-21	0.88040292	3.39E-61	0.52495504	1.71E-14
1.40E-43	-0.4683866	1.78E-11	0.66511012	5.43E-25	0.27588008	0.00014413
1.96E-28	-0.5336621	5.22E-15	0.79024452	8.90E-41	0.28843617	6.85E-05
2.60E-57	-0.4580037	5.56E-11	0.72197118	4.37E-31	0.32492047	6.41E-06
2.37E-54	-0.5478711	6.97E-16	0.91743477	3.66E-75	0.63689666	1.94E-22
6.36E-74	-0.5353415	4.13E-15	0.83241201	8.58E-49	0.47871114	5.49E-12
4.52E-18	-0.4659822	2.32E-11	0.84154132	7.95E-51	0.41057036	6.46E-09
6.07E-46	-0.5637408	6.56E-17	0.93654659	3.06E-85	0.78043711	3.57E-39
7.61E-19	-0.552824	3.38E-16	0.80520274	2.15E-43	0.77763277	9.89E-39
5.69E-05	-0.4016077	1.46E-08	0.65069189	1.18E-23	0.48590909	2.36E-12

1.69E-63	-0.5697982	2.57E-17	0.94192481	1.19E-88	0.67627609	4.42E-26
5.97E-28	-0.3713805	1.95E-07	0.87598606	7.59E-60	0.77885839	6.35E-39
1.74E-48	-0.4783019	5.75E-12	0.87313079	5.31E-59	0.80258092	6.41E-43
1.11E-27	0.4003201	1.64E-08	-0.7012388	1.07E-28	-0.7115027	7.46E-30
1.91E-38	-0.3931078	3.11E-08	0.67681828	3.91E-26	0.24713711	0.00069535
0.00152495	-0.2593446	0.00036421	0.50946197	1.30E-13	0.59101812	8.29E-19
2.74E-13	0.39839964	1.95E-08	-0.5613238	9.48E-17	-0.0203818	0.78303021
2.65E-40	0.52167965	2.65E-14	-0.7176373	1.44E-30	-0.3604135	4.67E-07
1.31E-15	0.29769822	3.87E-05	-0.4137397	4.81E-09	-0.0500722	0.49849222
5.94E-27	-0.5240414	1.93E-14	0.67748593	3.35E-26	0.69472515	5.47E-28
2.68E-17	-0.5447509	1.09E-15	0.74192532	1.34E-33	0.18520194	0.01160882
1.08E-27	0.53971095	2.24E-15	-0.6360641	2.29E-22	-0.1993895	0.00650875
7.00E-48	-0.5256691	1.55E-14	0.78669512	3.46E-40	0.5491109	5.82E-16
1.43E-08	0.17436165	0.01761024	-0.5738192	1.37E-17	-0.5429239	1.42E-15
1.16E-59	0.52775826	1.17E-14	-0.8152427	2.78E-45	-0.3939399	2.89E-08
0.00025126	-0.4051416	1.06E-08	0.5531968	3.20E-16	0.61880548	6.18E-21
1.22E-17	-0.5623669	8.09E-17	0.74260383	1.09E-33	0.37289893	1.72E-07
1.11E-44	-0.4075013	8.57E-09	0.73508858	1.03E-32	0.30788272	2.01E-05
6.55E-18	-0.3926444	3.24E-08	0.79867706	3.17E-42	0.71460394	3.26E-30
7.01E-32	0.4062367	9.62E-09	-0.6345963	3.05E-22	-0.304291	2.54E-05
0.00075662	-0.2156061	0.00320428	0.48774708	1.90E-12	0.74758766	2.35E-34
1.07E-27	-0.418084	3.19E-09	0.83428872	3.36E-49	0.58288804	3.18E-18
8.75E-15	0.27903313	0.00011996	-0.6139846	1.50E-20	-0.6971284	3.01E-28
1.44E-24	0.41313551	5.09E-09	-0.8186801	5.91E-46	-0.3666946	2.84E-07
9.63E-34	-0.3825832	7.69E-08	0.80044293	1.54E-42	0.34205598	1.88E-06
9.02E-24	-0.4737744	9.67E-12	0.8234821	6.41E-47	0.36690413	2.79E-07
1.62E-42	0.43246986	7.88E-10	-0.6269919	1.33E-21	-0.5138625	7.40E-14
2.54E-11	0.52378954	2.00E-14	-0.5763414	9.16E-18	-0.6166902	9.13E-21
1.05E-39	0.7155874	2.50E-30	-0.8245974	3.79E-47	-0.5842206	2.56E-18
0.00010922	-0.4160314	3.87E-09	0.58611643	1.87E-18	0.56981396	2.57E-17
7.46E-33	-0.3816483	8.32E-08	0.72383717	2.60E-31	0.495147	7.79E-13
3.26E-35	-0.4876331	1.93E-12	0.89322376	1.94E-65	0.54143759	1.75E-15
2.55E-41	-0.451763	1.09E-10	0.72289906	3.37E-31	0.27429483	0.00015793
1.21E-62	-0.4784568	5.65E-12	0.78975614	1.07E-40	0.35706111	6.07E-07
1.67E-39	-0.49982	4.39E-13	0.86403156	1.94E-56	0.42449583	1.72E-09
3.96E-74	-0.5319062	6.65E-15	0.83595084	1.45E-49	0.48939307	1.56E-12
3.05E-12	-0.3195778	9.25E-06	0.77318314	4.85E-38	0.35061876	9.94E-07
1.02E-49	-0.5913849	7.79E-19	0.88660451	3.48E-63	0.57071819	2.23E-17
5.45E-24	-0.5360106	3.77E-15	0.88629309	4.41E-63	0.44693994	1.80E-10
5.95E-27	0.23015383	0.00162361	-0.52731	1.25E-14	-0.4412823	3.23E-10
8.78E-07	-0.4719433	1.19E-11	0.69808735	2.37E-28	0.47100541	1.32E-11
2.55E-20	-0.4255316	1.56E-09	0.63553435	2.54E-22	0.74869274	1.66E-34
3.97E-28	-0.4962777	6.79E-13	0.79204446	4.42E-41	0.48393484	2.98E-12
2.85E-48	-0.5186256	3.97E-14	0.90805623	4.45E-71	0.55164069	4.02E-16
1.70E-43	0.36871076	2.42E-07	-0.7412346	1.65E-33	-0.3745227	1.50E-07
3.74E-17	-0.4551215	7.59E-11	0.8257418	2.20E-47	0.71081227	8.96E-30

4.73E-27	0.66307196	8.48E-25	-0.6147292	1.31E-20	-0.4624333	3.43E-11
7.97E-15	0.65430326	5.56E-24	-0.7354692	9.22E-33	-0.7405511	2.03E-33
9.26E-08	0.22014213	0.00260399	-0.4925401	1.07E-12	-0.6617168	1.14E-24
1.41E-07	0.19671045	0.00728147	-0.5277153	1.18E-14	-0.5043632	2.49E-13
8.15E-07	-0.492611	1.06E-12	0.62802155	1.09E-21	0.5615735	9.13E-17
1.28E-27	0.38462258	6.47E-08	-0.7542887	2.82E-35	-0.4922278	1.11E-12
2.41E-15	0.33167927	3.99E-06	-0.5748278	1.17E-17	-0.2047009	0.00518963
5.85E-55	-0.5904924	9.05E-19	0.89424759	8.44E-66	0.76134936	2.80E-36
0.02800353	0.23696744	0.00116365	-0.4816643	3.89E-12	-0.4898234	1.48E-12
1.80E-07	0.24932303	0.00062075	-0.6425093	6.33E-23	-0.570254	2.40E-17
3.73E-30	0.68001822	1.86E-26	-0.7361372	7.57E-33	-0.4327554	7.66E-10
4.88E-47	-0.5746642	1.20E-17	0.94123568	3.38E-88	0.60690311	5.34E-20
1.21E-18	0.47419624	9.22E-12	-0.6514999	1.00E-23	-0.6892404	2.09E-27
3.55E-35	0.20008714	0.00631994	-0.6493295	1.57E-23	-0.4188486	2.97E-09
4.21E-19	-0.0573897	0.43778844	0.53248941	6.14E-15	0.08588974	0.24504939
1.60E-11	-0.1381637	0.06072437	0.65299519	7.32E-24	0.39370714	2.95E-08
6.23E-15	-0.4199812	2.66E-09	0.67140753	1.34E-25	0.73478087	1.13E-32
1.41E-28	0.58029835	4.85E-18	-0.8167016	1.45E-45	-0.4712354	1.29E-11
8.63E-13	-0.450935	1.18E-10	0.77445495	3.09E-38	0.29677627	4.10E-05
6.25E-22	0.35982566	4.89E-07	-0.7428105	1.02E-33	-0.3554586	6.87E-07
1.35E-20	-0.3834697	7.13E-08	0.806274	1.37E-43	0.32866836	4.93E-06
1.37E-65	-0.3651484	3.21E-07	0.83454414	2.95E-49	0.61975177	5.19E-21
2.70E-64	-0.6126559	1.91E-20	0.85867674	5.15E-55	0.57752985	7.57E-18
0.00302501	0.18740109	0.01063939	-0.4825385	3.52E-12	-0.4892744	1.58E-12
1.11E-10	0.43418929	6.63E-10	-0.7072705	2.27E-29	-0.5332956	5.49E-15
1.85E-13	0.3522582	8.78E-07	-0.6440185	4.67E-23	-0.5038281	2.66E-13
7.85E-33	0.2606867	0.00033856	-0.6685627	2.53E-25	-0.189676	0.00971239
6.59E-13	0.42602327	1.49E-09	-0.5342622	4.80E-15	-0.3738158	1.59E-07
0.00026286	0.20047773	0.00621637	-0.372675	1.75E-07	-0.243517	0.00083728
5.17E-23	-0.3820699	8.03E-08	0.70460982	4.52E-29	0.8935309	1.51E-65
0.04320704	-0.3134729	1.39E-05	0.51845877	4.06E-14	0.07738939	0.29507823
1.54E-48	-0.5389984	2.48E-15	0.78027127	3.79E-39	0.34658704	1.35E-06
2.21E-09	-0.4856646	2.43E-12	0.74567585	4.25E-34	0.68319172	8.85E-27
1.08E-11	-0.3888219	4.51E-08	0.77119511	9.75E-38	0.40260379	1.34E-08
2.01E-49	-0.5699633	2.51E-17	0.85005056	7.69E-53	0.64049185	9.50E-23
7.48E-20	-0.4437541	2.51E-10	0.66261405	9.37E-25	0.2595712	0.00035975
1.80E-14	-0.4862137	2.28E-12	0.76366601	1.29E-36	0.69427875	6.10E-28
1.92E-07	0.36704416	2.76E-07	-0.5798697	5.20E-18	-0.6372189	1.82E-22
1.29E-31	0.69855031	2.11E-28	-0.8189581	5.20E-46	-0.6555817	4.24E-24
3.93E-10	-0.4776675	6.19E-12	0.75275296	4.61E-35	0.67464404	6.43E-26
1.56E-13	0.12237278	0.09703299	-0.5891489	1.13E-18	-0.2100503	0.00410843
9.18E-05	-0.4126001	5.35E-09	0.60664951	5.59E-20	0.32425464	6.71E-06
6.04E-18	-0.4095889	7.07E-09	0.65156313	9.88E-24	0.56696411	4.00E-17
3.13E-37	-0.5123848	8.96E-14	0.92111097	6.72E-77	0.59595337	3.59E-19
1.66E-16	0.53482159	4.44E-15	-0.8018479	8.68E-43	-0.497118	6.12E-13
6.13E-10	-0.4400999	3.65E-10	0.65139061	1.02E-23	0.77858823	7.00E-39

9.41E-22	0.23952988	0.00102413	-0.6178611	7.36E-21	-0.3626612	3.91E-07
5.91E-12	-0.4938974	9.07E-13	0.6877325	3.00E-27	0.24858629	0.00064502
7.32E-14	-0.427266	1.32E-09	0.55707441	1.80E-16	0.25995446	0.00035233
1.79E-25	0.2772086	0.00013344	-0.6223223	3.21E-21	-0.2070096	0.0046951
2.65E-23	0.53055549	8.00E-15	-0.564967	5.44E-17	-0.24769	0.00067574
2.46E-41	-0.4286005	1.15E-09	0.82521796	2.83E-47	0.3503001	1.02E-06
2.14E-17	-0.2857334	8.06E-05	0.70305062	6.74E-29	0.17582517	0.01666794
1.70E-46	-0.5240978	1.92E-14	0.90789451	5.18E-71	0.48818097	1.81E-12
2.61E-31	-0.393031	3.13E-08	0.77097135	1.05E-37	0.34153918	1.96E-06
6.33E-16	0.67826521	2.80E-26	-0.572344	1.73E-17	-0.6887634	2.34E-27
2.91E-06	0.47242764	1.13E-11	-0.5064875	1.90E-13	-0.6932967	7.77E-28
1.03E-15	0.0771506	0.29657525	-0.4071073	8.88E-09	-0.2942751	4.79E-05
0.00086073	-0.0844622	0.25301058	0.51824818	4.17E-14	0.3013395	3.07E-05
1.40E-11	0.2937528	4.94E-05	-0.6043251	8.43E-20	-0.3848443	6.35E-08
1.83E-07	0.28981516	6.30E-05	-0.5173283	4.71E-14	-0.5359045	3.82E-15
9.64E-20	0.31389093	1.36E-05	-0.5361916	3.67E-15	-0.7674981	3.51E-37
4.72E-35	-0.5920083	7.02E-19	0.83980267	1.98E-50	0.47958038	4.96E-12
2.04E-32	-0.3715139	1.92E-07	0.71529507	2.71E-30	0.18877252	0.01007164
0.4916009	-0.1183689	0.10855417	0.34062449	2.09E-06	0.12786012	0.08284281
0.92138317	-0.2520902	0.00053691	0.31909228	9.56E-06	0.03494235	0.63679095
1.71E-25	-0.5242956	1.87E-14	0.9212786	5.58E-77	0.69546733	4.55E-28
3.38E-33	-0.4363821	5.32E-10	0.82399566	5.04E-47	0.45298248	9.53E-11
7.87E-10	0.23446685	0.00131641	-0.5343408	4.75E-15	-0.5513204	4.22E-16
6.81E-10	-0.0369309	0.61772297	0.44908278	1.44E-10	0.05035704	0.49604902
8.57E-52	0.36922441	2.32E-07	-0.65765	2.73E-24	-0.3729834	1.71E-07
4.20E-23	-0.5643503	5.97E-17	0.60716822	5.10E-20	0.18919891	0.00990065
1.08E-10	0.33951764	2.27E-06	-0.7250146	1.87E-31	-0.4260359	1.48E-09
9.81E-33	0.45433301	8.26E-11	-0.8681518	1.42E-57	-0.4479869	1.62E-10
7.02E-22	0.38659345	5.47E-08	-0.7394059	2.86E-33	-0.7796921	4.68E-39
6.77E-08	0.34826189	1.19E-06	-0.6727978	9.78E-26	-0.3114499	1.59E-05
4.18E-63	-0.5077332	1.63E-13	0.80065631	1.42E-42	0.4467567	1.84E-10
2.34E-07	0.35726784	5.97E-07	-0.4290348	1.11E-09	0.13779744	0.06141756
7.46E-37	-0.5025749	3.11E-13	0.90738247	8.40E-71	0.67833966	2.75E-26
0.08097927	-0.4308607	9.24E-10	0.33981978	2.22E-06	-0.1179282	0.10988537
6.86E-46	-0.571353	2.02E-17	0.94145222	2.44E-88	0.547684	7.16E-16
2.09E-21	-0.3486207	1.16E-06	0.8314145	1.41E-48	0.76800268	2.95E-37
7.41E-19	0.08838731	0.2315436	-0.5481183	6.73E-16	-0.5206332	3.05E-14
3.06E-12	0.58428563	2.53E-18	-0.7471653	2.68E-34	-0.5649852	5.42E-17
0.00024275	-0.1589937	0.03064436	0.35811767	5.59E-07	0.03983328	0.59034736
1.45E-51	-0.3762229	1.31E-07	0.71185938	6.79E-30	0.51012333	1.20E-13
2.43E-14	0.35657862	6.30E-07	-0.7891708	1.35E-40	-0.3541726	7.58E-07
1.38E-19	0.79988613	1.94E-42	-0.7550367	2.21E-35	-0.4775617	6.27E-12
3.50E-29	0.5173574	4.69E-14	-0.8683036	1.29E-57	-0.7387734	3.46E-33
2.18E-06	-0.3130395	1.43E-05	0.6668265	3.72E-25	0.64385201	4.83E-23
6.24E-06	0.0467913	0.52708415	0.50316997	2.89E-13	0.63492308	2.87E-22
3.38E-44	-0.580946	4.37E-18	0.9715126	2.28E-116	0.65630162	3.64E-24

2.96E-25	-0.5298531	8.81E-15	0.8771175	3.46E-60	0.49721281	6.05E-13
4.30E-10	0.2530398	0.00051063	-0.6823275	1.08E-26	-0.4313784	8.78E-10
2.16E-33	-0.4425745	2.83E-10	0.77540302	2.20E-38	0.2504829	0.00058423
6.82E-24	-0.496008	7.01E-13	0.84109114	1.01E-50	0.78820254	1.95E-40
2.24E-06	-0.4765885	7.01E-12	0.64687047	2.61E-23	0.44533715	2.13E-10
6.12E-12	-0.4315437	8.64E-10	0.79343365	2.57E-41	0.64779322	2.16E-23
5.41E-12	0.41030769	6.62E-09	-0.6888692	2.28E-27	-0.4698233	1.51E-11
9.35E-20	0.43292977	7.52E-10	-0.7619024	2.33E-36	-0.4735046	9.97E-12
9.58E-15	-0.325001	6.37E-06	0.78733355	2.72E-40	0.36639383	2.91E-07
1.73E-23	0.44581051	2.03E-10	-0.8237623	5.62E-47	-0.5589905	1.35E-16
0.00036856	0.17318301	0.01840245	-0.4800871	4.68E-12	-0.5884898	1.26E-18
1.34E-08	-0.4603932	4.29E-11	0.54108964	1.84E-15	0.0060726	0.93461715
0.0001876	-0.5234839	2.08E-14	0.59101947	8.29E-19	0.45205556	1.05E-10
0.04334226	0.21897006	0.00274845	-0.5625885	7.82E-17	-0.3137864	1.37E-05
1.48E-41	-0.5432506	1.36E-15	0.90100938	2.75E-68	0.49735341	5.95E-13
3.30E-18	0.35630431	6.43E-07	-0.7466309	3.16E-34	-0.3832093	7.29E-08
2.66E-13	-0.4880664	1.83E-12	0.73525274	9.83E-33	0.67378209	7.82E-26
9.40E-33	0.44624021	1.94E-10	-0.7995931	2.18E-42	-0.6108822	2.62E-20
3.37E-40	-0.5386168	2.61E-15	0.84946698	1.07E-52	0.83032518	2.41E-48
3.82E-06	0.67087472	1.51E-25	-0.5048299	2.35E-13	-0.5321884	6.40E-15
8.74E-08	-0.5144544	6.85E-14	0.70595485	3.19E-29	0.65376431	6.23E-24
7.71E-19	0.28814359	6.97E-05	-0.640144	1.02E-22	-0.6544573	5.38E-24
7.26E-38	0.28356889	9.18E-05	-0.6222434	3.26E-21	-0.2481472	0.00065991
8.89E-32	0.35812809	5.58E-07	-0.6648678	5.73E-25	-0.3489322	1.13E-06
0.00163834	0.18320716	0.01255455	-0.3387821	2.39E-06	-0.3696469	2.24E-07
4.85E-05	-0.2906953	5.97E-05	0.52698766	1.30E-14	0.57171811	1.90E-17
0.00034111	0.34153685	1.96E-06	-0.4444639	2.33E-10	-0.5292387	9.58E-15
2.16E-22	-0.5113729	1.02E-13	0.84680884	4.65E-52	0.8186392	6.02E-46
3.47E-08	0.69697266	3.13E-28	-0.5928691	6.07E-19	-0.2397339	0.0010137
4.53E-44	-0.4065995	9.31E-09	0.57616976	9.41E-18	0.34845965	1.17E-06
2.20E-48	-0.4352064	5.99E-10	0.81792353	8.33E-46	0.50894525	1.39E-13
3.55E-45	-0.4499558	1.31E-10	0.88830843	9.45E-64	0.50584227	2.07E-13
5.84E-23	-0.4062135	9.64E-09	0.82150764	1.61E-46	0.38451295	6.53E-08
1.14E-49	-0.527353	1.24E-14	0.85112741	4.19E-53	0.73105341	3.34E-32
0.18121192	-0.3057669	2.31E-05	0.32718645	5.47E-06	-0.0109719	0.88216379
8.97E-31	-0.5255869	1.57E-14	0.77402641	3.60E-38	0.32375864	6.94E-06
1.37E-75	-0.5507027	4.61E-16	0.90668784	1.61E-70	0.55332699	3.14E-16
3.81E-06	0.43451665	6.42E-10	-0.6348036	2.93E-22	0.01828186	0.80491174
7.52E-32	-0.4648177	2.64E-11	0.93619651	4.98E-85	0.65592316	3.95E-24
2.71E-31	0.23643929	0.00119449	-0.7007469	1.21E-28	-0.3000157	3.34E-05
0.00056518	-0.3918604	3.47E-08	0.50867638	1.44E-13	0.7355861	8.91E-33
1.49E-26	-0.4169175	3.56E-09	0.65342994	6.68E-24	0.53092282	7.61E-15
8.44E-32	-0.2501217	0.00059539	0.52133512	2.78E-14	0.72550594	1.63E-31
0.00370549	0.27965392	0.00011567	-0.3944551	2.76E-08	-0.5070361	1.78E-13
1.89E-24	0.27904965	0.00011984	-0.6932839	7.80E-28	-0.7278498	8.37E-32
7.40E-46	0.68107039	1.46E-26	-0.6640116	6.91E-25	-0.4175402	3.36E-09

5.45E-36	0.52367594	2.03E-14	-0.8289064	4.81E-48		-0.5918134	7.25E-19
3.65E-10	-0.3995206	1.76E-08	0.51671992	5.10E-14		0.29251049	5.34E-05
7.72E-53	-0.538604	2.62E-15	0.92912685	5.39E-81		0.52804743	1.13E-14
5.73E-44	-0.5116257	9.88E-14	0.77830367	7.76E-39		0.33594628	2.94E-06
1.64E-43	0.46902898	1.65E-11	-0.859619	2.92E-55		-0.5627325	7.65E-17
2.24E-14	0.3023839	2.87E-05	-0.699323	1.74E-28		-0.5923572	6.62E-19
4.71E-78	-0.5038659	2.65E-13	0.85996854	2.37E-55		0.51094769	1.08E-13
1.07E-05	-0.4157347	3.98E-09	0.68483767	6.00E-27		0.60682281	5.42E-20
1.92E-16	0.24104966	0.0009488	-0.5561871	2.05E-16		-0.5276872	1.18E-14
2.59E-10	0.12793021	0.08267299	-0.5270113	1.30E-14		-0.7062237	2.98E-29
9.16E-11	0.53588607	3.83E-15	-0.5727711	1.61E-17		-0.3416433	1.94E-06
2.30E-46	-0.3567088	6.23E-07	0.79003709	9.64E-41		0.44266452	2.81E-10
3.36E-12	0.49946546	4.58E-13	-0.8001448	1.74E-42		-0.3164812	1.14E-05
3.83E-39	-0.2357212	0.00123763	0.62531562	1.83E-21		0.27815201	0.0001263
0.00016996	0.57082098	2.19E-17	-0.5906613	8.80E-19		-0.178528	0.01504264
0.00121925	-0.4923731	1.09E-12	0.55212499	3.75E-16		0.28062817	0.00010923
5.75E-12	0.37637173	1.29E-07	-0.7083227	1.72E-29		-0.5297838	8.89E-15
2.98E-14	-0.5104585	1.15E-13	0.60423555	8.56E-20		0.0588003	0.42659139
1.21E-13	0.68115387	1.43E-26	-0.6350597	2.79E-22		-0.532741	5.93E-15
2.64E-55	-0.4212716	2.35E-09	0.85988275	2.49E-55		0.4391033	4.04E-10
4.29E-06	-0.4449826	2.21E-10	0.69208077	1.05E-27		0.50721832	1.74E-13
3.26E-26	-0.3198227	9.09E-06	0.64804192	2.05E-23		0.08073114	0.27465564
1.98E-15	0.32862685	4.95E-06	-0.5142979	7.00E-14		0.00433401	0.9533108
2.19E-08	-0.4199766	2.66E-09	0.77241079	6.36E-38		0.49091479	1.30E-12
7.70E-15	0.40085438	1.57E-08	-0.6327243	4.40E-22		-0.7348142	1.12E-32
3.60E-10	0.52449954	1.82E-14	-0.7175319	1.48E-30		-0.2976682	3.87E-05
1.73E-43	-0.5254112	1.61E-14	0.94329942	1.41E-89		0.53418241	4.86E-15
1.94E-13	-0.5326895	5.97E-15	0.6567807	3.29E-24		0.71053776	9.63E-30
6.70E-23	0.54424459	1.18E-15	-0.6732557	8.81E-26		-0.8338212	4.24E-49
2.50E-08	0.16776021	0.02245862	-0.4723056	1.14E-11		-0.7360532	7.76E-33
1.07E-17	0.29168297	5.62E-05	-0.6038959	9.09E-20		-0.8104055	2.33E-44
3.85E-26	-0.3843837	6.60E-08	0.50325375	2.86E-13		0.84383813	2.34E-51
3.44E-23	0.40084508	1.57E-08	-0.6871038	3.49E-27		-0.8568327	1.54E-54
1.14E-12	-0.415035	4.26E-09	0.49863976	5.08E-13		0.92022162	1.80E-76
6.49E-20	-0.3361607	2.89E-06	0.50747751	1.68E-13		0.9038987	2.09E-69
1.21E-11	-0.2682697	0.00022246	0.49206716	1.13E-12		0.830309	2.42E-48
2.13E-09	-0.4792185	5.18E-12	0.49814614	5.39E-13		0.74145782	1.54E-33
1.28E-07	-0.2902415	6.13E-05	0.44066667	3.44E-10		0.86512832	9.77E-57
4.99E-33	-0.3347527	3.20E-06	0.55799184	1.57E-16		0.91611554	1.47E-74
5.02E-45	-0.4614802	3.81E-11	0.72339073	2.94E-31		0.92627602	1.74E-79
9.50E-20	-0.3584577	5.44E-07	0.55104284	4.39E-16		0.922701	1.12E-77
1.03E-77	-0.4985624	5.12E-13	0.81813984	7.55E-46		0.87223931	9.66E-59
5.63E-31	-0.3357393	2.98E-06	0.53831908	2.73E-15		0.9209374	8.15E-77
2.15E-25	0.44806081	1.60E-10	-0.6317248	5.35E-22		-0.8030523	5.27E-43
3.24E-30	-0.4363093	5.36E-10	0.65637623	3.59E-24		0.93964113	3.64E-87
3.13E-14	-0.3294471	4.67E-06	0.44688349	1.81E-10		0.9344162	5.69E-84

2.34E-26	0.49416614	8.78E-13	-0.692093	1.04E-27	-0.8872113	2.19E-63
2.27E-15	-0.4891189	1.61E-12	0.50661659	1.87E-13	0.84145766	8.31E-51
1.74E-34	-0.3766949	1.26E-07	0.72113421	5.50E-31	0.85273497	1.68E-53
1.47E-08	-0.2729203	0.00017089	0.44743151	1.71E-10	0.85077767	5.11E-53
6.41E-19	-0.384099	6.76E-08	0.55326621	3.17E-16	0.91317955	2.98E-73
1.70E-16	-0.3098474	1.77E-05	0.47670157	6.92E-12	0.87325592	4.88E-59
3.90E-08	-0.1997029	0.00642331	0.40671868	9.20E-09	0.89502018	4.47E-66
8.99E-16	-0.369848	2.20E-07	0.60469492	7.89E-20	0.94325302	1.52E-89
4.19E-12	-0.3616634	4.23E-07	0.59174192	7.34E-19	0.90780937	5.62E-71
6.56E-20	-0.3479635	1.21E-06	0.45757096	5.83E-11	0.90402205	1.87E-69
2.92E-18	-0.5247115	1.77E-14	0.59369553	5.28E-19	0.89468351	5.90E-66
8.41E-11	-0.5454373	9.91E-16	0.44643125	1.90E-10	0.72516196	1.79E-31
9.90E-31	0.52715826	1.27E-14	-0.6506369	1.20E-23	-0.8303641	2.36E-48
6.27E-10	-0.2922339	5.43E-05	0.53048526	8.08E-15	0.82989657	2.97E-48
3.03E-18	-0.4117976	5.76E-09	0.57858986	6.39E-18	0.88146427	1.58E-61
1.75E-18	-0.3431974	1.73E-06	0.45439666	8.20E-11	0.92672024	1.02E-79
1.43E-28	-0.4404874	3.51E-10	0.51064841	1.12E-13	0.85183617	2.80E-53
2.06E-16	-0.4316024	8.59E-10	0.5379305	2.88E-15	0.85508191	4.32E-54
2.51E-20	-0.3799094	9.63E-08	0.57016708	2.43E-17	0.85050741	5.95E-53
2.50E-24	-0.2524037	0.0005281	0.57587167	9.87E-18	0.87917308	8.15E-61
9.56E-33	-0.4346029	6.36E-10	0.74735467	2.53E-34	0.93323063	2.78E-83
5.14E-08	-0.2460864	0.00073407	0.42087163	2.45E-09	0.83868146	3.56E-50
1.11E-06	-0.3606185	4.60E-07	0.4393003	3.96E-10	0.83805828	4.91E-50
2.02E-16	0.27230424	0.00017702	-0.6159623	1.04E-20	-0.8500458	7.71E-53
6.70E-08	-0.4148846	4.32E-09	0.50569338	2.10E-13	0.76135814	2.79E-36
2.02E-25	-0.3936186	2.97E-08	0.60040406	1.67E-19	0.92533933	5.29E-79
1.00E-17	0.31642893	1.14E-05	-0.5669903	3.98E-17	-0.7982181	3.81E-42
1.43E-22	-0.3308836	4.22E-06	0.52393411	1.96E-14	0.91685602	6.74E-75
1.21E-24	-0.1942286	0.00806906	0.59722124	2.89E-19	0.91889769	7.62E-76
1.37E-05	0.01484423	0.84105367	-0.3431341	1.74E-06	-0.649822	1.42E-23
2.08E-12	0.30849028	1.94E-05	-0.5198399	3.38E-14	-0.8126742	8.67E-45
3.98E-22	-0.379138	1.03E-07	0.61536718	1.16E-20	0.84732117	3.51E-52
1.82E-24	-0.3306632	4.29E-06	0.53383759	5.09E-15	0.91380499	1.58E-73
7.75E-16	-0.3541355	7.60E-07	0.65873094	2.17E-24	0.94384864	5.94E-90
3.28E-29	-0.5360381	3.75E-15	0.72195666	4.38E-31	0.88362009	3.26E-62
0.00010963	-0.144163	0.05025607	0.34018859	2.16E-06	0.74921413	1.41E-34
7.28E-19	-0.5170875	4.86E-14	0.7034718	6.05E-29	0.91668792	8.05E-75
1.30E-20	-0.427463	1.29E-09	0.60029804	1.70E-19	0.90909419	1.65E-71
7.86E-22	-0.3551806	7.01E-07	0.58817307	1.33E-18	0.84641865	5.76E-52
3.02E-15	-0.3295676	4.63E-06	0.54139846	1.76E-15	0.85623459	2.20E-54
2.08E-38	-0.4718131	1.21E-11	0.71054894	9.60E-30	0.87683004	4.23E-60
1.76E-30	-0.4323314	7.99E-10	0.73139397	3.03E-32	0.92751614	3.91E-80
3.02E-18	-0.3558355	6.67E-07	0.69195795	1.08E-27	0.8875404	1.71E-63
2.75E-24	-0.377194	1.21E-07	0.72326717	3.04E-31	0.92455948	1.32E-78
3.43E-25	-0.4648445	2.63E-11	0.51213198	9.26E-14	0.83320109	5.79E-49
3.49E-30	-0.4255471	1.56E-09	0.68062725	1.62E-26	0.9500943	1.63E-94

3.05E-15	-0.1667874	0.02326252	0.52243048	2.40E-14	0.80653957	1.22E-43
1.39E-05	-0.30435	2.53E-05	0.43631973	5.36E-10	0.76275611	1.75E-36
1.53E-16	-0.4197215	2.73E-09	0.68167674	1.26E-26	0.86817307	1.40E-57
5.99E-29	-0.3966305	2.28E-08	0.56140224	9.37E-17	0.90432488	1.42E-69
5.22E-13	-0.4217999	2.24E-09	0.60216224	1.23E-19	0.90852512	2.85E-71
2.07E-29	-0.4633649	3.10E-11	0.67052353	1.63E-25	0.8966912	1.11E-66
5.43E-12	-0.2724798	0.00017525	0.52818532	1.11E-14	0.81013944	2.62E-44
8.13E-14	-0.3568348	6.17E-07	0.42479143	1.68E-09	0.83063636	2.06E-48
6.68E-20	-0.5022621	3.24E-13	0.70137097	1.03E-28	0.89731974	6.57E-67

MMblack	p.MMblack	MMbrown	p.MMbrown	MMgrey	p.MMgrey
0.92033899	1.58E-76	0.6559668	3.91E-24	0.07184689	0.33112194
0.97318506	9.69E-119	0.64621169	2.99E-23	0.00161373	0.9826072
0.85190353	2.70E-53	0.50571505	2.10E-13	-0.045436	0.53913296
0.96510029	1.99E-108	0.59263045	6.32E-19	0.013584	0.85439121
0.94326135	1.50E-89	0.71452466	3.33E-30	-0.0052526	0.94343014
0.95283749	1.05E-96	0.59112889	8.14E-19	0.0290481	0.69468746
0.87527222	1.24E-59	0.54063891	1.97E-15	0.01645412	0.82408156
0.94404736	4.34E-90	0.72623497	1.32E-31	0.0739154	0.31735283
0.85612612	2.34E-54	0.51605122	5.57E-14	0.18707252	0.01077953
0.90870984	2.39E-71	0.54701125	7.90E-16	-0.017405	0.81409511
0.91859573	1.06E-75	0.60442414	8.28E-20	0.04439746	0.54845705
0.73541244	9.38E-33	0.41984517	2.70E-09	0.25801127	0.00039146
0.92607966	2.20E-79	0.59984886	1.84E-19	0.01836035	0.80409108
0.93400598	9.89E-84	0.5949349	4.28E-19	0.0886693	0.23005229
0.94226677	7.02E-89	0.57844748	6.54E-18	0.07303422	0.32317227
0.93662906	2.73E-85	0.65524419	4.56E-24	0.09050629	0.22050323
0.5488613	6.04E-16	0.34426158	1.60E-06	0.19266807	0.00860221
0.96285976	5.33E-106	0.56492305	5.47E-17	0.0886548	0.23012879
0.8925142	3.44E-65	0.63140723	5.69E-22	0.04408056	0.5513179
0.47841907	5.68E-12	0.3706704	2.06E-07	-0.2271998	0.00187036
0.87197312	1.15E-58	0.52296788	2.23E-14	0.07256253	0.32631555
0.82655366	1.50E-47	0.61943839	5.50E-21	-0.0401066	0.58779834
0.90666574	1.64E-70	0.43202691	8.23E-10	0.10666208	0.14844282
0.95213581	3.92E-96	0.64912014	1.64E-23	0.06154409	0.40529284
0.88521878	9.91E-63	0.47938002	5.08E-12	0.01730379	0.81515631
0.8687526	9.61E-58	0.542988	1.41E-15	-0.0492133	0.50589727
0.9712303	5.54E-116	0.63144557	5.64E-22	0.09721264	0.18804216
0.93109535	4.48E-82	0.52218333	2.48E-14	-0.0004691	0.99494336
0.8341099	3.67E-49	0.58868456	1.22E-18	0.14672352	0.04626931
0.90920205	1.49E-71	0.65721881	3.00E-24	0.0607144	0.41166581
0.913683	1.79E-73	0.53001668	8.62E-15	0.07927885	0.28341036
0.91301933	3.50E-73	0.58027826	4.86E-18	0.05730304	0.43848163
-0.3620334	4.11E-07	-0.0139783	0.85021352	-0.2849742	8.44E-05
-0.2985379	3.67E-05	-0.0951315	0.19771736	-0.3831538	7.33E-08
-0.1599244	0.0296692	-0.1362711	0.06437666	-0.3462633	1.38E-06
-0.184292	0.01203217	0.07522176	0.30885172	-0.2300699	0.00163018
-0.453968	8.58E-11	-0.2124065	0.00370014	-0.2391204	0.00104534
-0.1669747	0.02310585	0.00302616	0.96739052	-0.3077623	2.03E-05
-0.3408493	2.06E-06	-0.2357288	0.00123716	-0.1649085	0.02488544
-0.287572	7.22E-05	-0.1722325	0.01906365	-0.403255	1.26E-08
-0.301725	3.00E-05	-0.2797272	0.00011517	-0.0916526	0.21468937
-0.1742527	0.01768222	-0.3123782	1.50E-05	0.1372048	0.06255298
-0.2690038	0.00021346	-0.0685147	0.35409572	-0.3907075	3.83E-08
-0.2915389	5.67E-05	-0.0866903	0.24066166	-0.0267863	0.71740525
-0.4072209	8.79E-09	-0.3196217	9.22E-06	-0.1283929	0.08155883

-0.3084079	1.95E-05	-0.1872749	0.01069304	-0.3660938	2.98E-07
-0.4494168	1.39E-10	-0.3249358	6.40E-06	-0.2126773	0.00365564
-0.3806622	9.04E-08	-0.2379514	0.00110813	0.0368744	0.61826121
-0.3599179	4.86E-07	-0.2688751	0.00021501	-0.2732292	0.0001679
-0.373423	1.65E-07	-0.220223	0.00259428	-0.209206	0.00426432
-0.2352872	0.00126438	-0.2901422	6.17E-05	0.2273893	0.00185356
-0.1674106	0.02274475	-0.1293484	0.07929636	-0.272267	0.00017739
-0.3996349	1.75E-08	-0.0249847	0.73568138	-0.1230457	0.09519548
-0.3452128	1.49E-06	-0.1649558	0.02484344	-0.005234	0.9436303
0.17642632	0.01629379	0.25977963	0.0003557	0.14304559	0.05208335
-0.3154542	1.22E-05	-0.1302222	0.07727201	-0.297372	3.95E-05
-0.2285481	0.00175378	-0.2479491	0.00066672	-0.3111382	1.63E-05
-0.4048679	1.09E-08	-0.4106895	6.39E-09	0.15966933	0.02993379
-0.3366244	2.80E-06	-0.2298938	0.00164407	-0.3494541	1.09E-06
-0.5954485	3.92E-19	-0.316071	1.17E-05	-0.1260416	0.08734802
-0.3501615	1.03E-06	-0.3348933	3.17E-06	-0.2536177	0.00049523
-0.3087311	1.91E-05	-0.1313415	0.07473986	-0.4113857	5.99E-09
-0.5586307	1.42E-16	-0.3420527	1.88E-06	-0.1893443	0.00984293
-0.4093728	7.21E-09	-0.1740435	0.01782112	-0.1699715	0.0207199
-0.2772355	0.00013323	-0.1874617	0.01061373	-0.2449469	0.00077831
-0.3426262	1.81E-06	-0.1570761	0.03273995	-0.2282768	0.00177669
-0.5203467	3.16E-14	-0.3316152	4.01E-06	-0.3103811	1.71E-05
-0.3406673	2.09E-06	-0.1634958	0.0261689	-0.1477339	0.04477055
-0.2943502	4.76E-05	-0.1498695	0.04173593	-0.4846166	2.75E-12
-0.4745886	8.81E-12	-0.1873098	0.01067817	-0.1985611	0.00673948
-0.3301135	4.46E-06	-0.1550318	0.03510671	-0.1681665	0.02213004
-0.1543654	0.0359089	-0.0206801	0.77993594	-0.2155593	0.00321108
-0.3587219	5.33E-07	-0.1783359	0.01515344	-0.2940858	4.84E-05
-0.5645958	5.75E-17	-0.3012955	3.08E-05	-0.3717255	1.89E-07
-0.2902621	6.13E-05	-0.1650708	0.02474151	-0.3338179	3.42E-06
-0.2945022	4.72E-05	-0.061884	0.40269899	-0.2550334	0.0004593
-0.4645428	2.72E-11	-0.1236128	0.09366848	-0.176072	0.01651342
0.44960228	1.36E-10	0.35834255	5.49E-07	0.24821431	0.00065761
-0.4468115	1.83E-10	-0.2622102	0.00031147	-0.329392	4.69E-06
-0.2141145	0.00342743	-0.1589	0.03074401	-0.1422875	0.05335418
-0.3284719	5.00E-06	-0.1275642	0.08356284	-0.1600732	0.02951576
0.76275623	1.75E-36	0.42581525	1.52E-09	0.18182393	0.01324944
0.38537967	6.06E-08	0.12100047	0.10086732	0.18657307	0.01099568
-0.3802412	9.36E-08	-0.2664395	0.00024647	-0.0992294	0.17900116
-0.532154	6.43E-15	-0.2796039	0.00011601	-0.2995058	3.45E-05
-0.3008985	3.16E-05	-0.2737281	0.00016316	-0.3028118	2.79E-05
-0.2961661	4.25E-05	-0.1453062	0.04844218	-0.3012931	3.08E-05
-0.2412008	0.0009416	-0.1110049	0.13251715	-0.3366284	2.80E-06
0.2758253	0.00014459	0.07309663	0.32275786	0.04298796	0.56123695
-0.2662819	0.00024865	-0.2023037	0.00575205	-0.1085554	0.14133022
-0.4769671	6.71E-12	-0.1678091	0.02241882	-0.3498501	1.05E-06

-0.3784792	1.08E-07	-0.1923548	0.0087129	-0.3389344	2.37E-06
-0.4417423	3.09E-10	-0.2474145	0.00068544	-0.293001	5.18E-05
0.47464085	8.76E-12	0.08978096	0.22423941	0.15152346	0.0395061
-0.2288268	0.00173052	-0.0127635	0.86309714	-0.2791298	0.00011928
0.52576807	1.53E-14	0.23438245	0.00132187	0.24417375	0.0008097
-0.1638072	0.02588125	0.07309081	0.32279648	-0.2488631	0.0006358
0.34250082	1.82E-06	0.1896171	0.00973546	0.27380116	0.00016248
-0.2360312	0.00121884	-0.0224654	0.76148611	-0.0865656	0.24134175
-0.3670835	2.75E-07	-0.1419637	0.0539046	-0.2033533	0.00549942
-0.4626738	3.34E-11	-0.2628654	0.00030046	-0.075554	0.30671383
-0.4344292	6.48E-10	-0.2639083	0.00028367	-0.1123598	0.12782716
-0.3456816	1.44E-06	-0.0967894	0.18998128	-0.0779442	0.2916198
-0.3335171	3.50E-06	-0.1916404	0.00897012	-0.2188975	0.00275762
0.38869772	4.56E-08	-0.0377991	0.60947588	0.22352782	0.00222452
-0.2410969	0.00094655	-0.1198301	0.10423106	-0.4389326	4.11E-10
-0.2760353	0.00014284	-0.2665319	0.0002452	-0.2028223	0.00562595
-0.3360042	2.93E-06	-0.1702102	0.02053941	-0.3623306	4.02E-07
0.52304829	2.21E-14	0.24899188	0.00063155	0.21242327	0.00369737
0.0217325	0.76904437	0.0073406	0.9210052	-0.2150234	0.00328984
0.50220711	3.26E-13	0.31267367	1.47E-05	0.22918405	0.00170113
-0.481127	4.15E-12	-0.2618459	0.00031776	-0.0595205	0.42093937
-0.5372272	3.18E-15	-0.2972498	3.98E-05	-0.242309	0.00089028
-0.1763436	0.01634485	-0.0566098	0.44405059	-0.3399457	2.20E-06
-0.3069813	2.14E-05	-0.1397747	0.05775116	-0.0771721	0.29644
-0.2467147	0.00071068	-0.2731899	0.00016827	-0.2859941	7.94E-05
0.39734059	2.14E-08	0.07700577	0.29748566	0.20818349	0.00446023
-0.2410457	0.00094899	-0.1377815	0.06144792	-0.0632904	0.39207207
-0.4231357	1.97E-09	-0.1984812	0.00676213	-0.2424356	0.00088459
-0.3685664	2.44E-07	-0.1810463	0.01365472	-0.3513827	9.38E-07
-0.30177	2.99E-05	-0.2971159	4.01E-05	-0.3075011	2.07E-05
-0.2594962	0.00036122	-0.1174427	0.11136648	0.10780066	0.1441337
-0.3557248	6.73E-07	-0.1960394	0.00748737	-0.3219288	7.87E-06
-0.3170523	1.10E-05	-0.0013408	0.98554815	-0.0738861	0.31754543
-0.3207185	8.55E-06	-0.231387	0.00152969	-0.3451875	1.49E-06
-0.3389545	2.36E-06	-0.1911125	0.00916449	-0.1020892	0.16673614
-0.3063087	2.23E-05	0.0111902	0.87983662	-0.1308382	0.07587002
-0.3561471	6.51E-07	-0.244608	0.00079193	-0.2199834	0.00262314
-0.4261479	1.47E-09	-0.2060904	0.00488668	-0.2037101	0.00541582
-0.4523556	1.02E-10	-0.2807755	0.00010828	-0.0343666	0.64235713
-0.314559	1.30E-05	-0.1261142	0.08716445	-0.0549407	0.45762031
0.45446236	8.14E-11	0.08058185	0.27554704	0.04853196	0.51181213
-0.4623577	3.46E-11	-0.3393145	2.30E-06	-0.4864963	2.21E-12
-0.4274822	1.29E-09	-0.306557	2.20E-05	-0.2563865	0.00042723
0.6856908	4.89E-27	0.33569089	2.99E-06	0.11939899	0.10549228
-0.1962782	0.00741351	-0.2445855	0.00079284	0.18328488	0.01251647
-0.3419724	1.90E-06	-0.2678675	0.00022754	-0.1419328	0.05395737

-0.2244642	0.00212885	-0.1851382	0.01163806	-0.2402859	0.000986
-0.2019278	0.00584502	-0.1071716	0.14650244	-0.4708903	1.34E-11
-0.3868573	5.34E-08	-0.2250617	0.00206976	0.01757624	0.81229934
0.27013809	0.00020021	0.16697282	0.0231074	0.2152262	0.00325984
-0.4245338	1.72E-09	-0.1723597	0.01897401	-0.0317306	0.66809374
0.02346735	0.75118999	-0.0204032	0.78280776	-0.2417969	0.00091367
-0.0951754	0.19750979	-0.2214623	0.00244955	-0.2090721	0.00428953
-0.2921138	5.47E-05	-0.1768191	0.01605333	-0.2853844	8.23E-05
-0.2813071	0.00010494	-0.2152779	0.00325222	-0.3328489	3.67E-06
-0.4296295	1.04E-09	-0.2514342	0.00055578	-0.0494698	0.50367992
-0.2610319	0.00033223	-0.0249836	0.73569216	-0.0571239	0.43991691
-0.242965	0.00086113	-0.1989573	0.00662824	-0.2077315	0.00454938
-0.3646704	3.34E-07	-0.1692119	0.02130356	-0.0264309	0.72099831
-0.4737411	9.71E-12	-0.2955177	4.43E-05	-0.3212715	8.24E-06
-0.4458756	2.01E-10	-0.3159978	1.18E-05	-0.2789985	0.0001202
-0.2887702	6.71E-05	-0.1148901	0.11941247	-0.209755	0.00416236
-0.2177174	0.00291084	-0.0268408	0.71685462	0.10436461	0.15743396
-0.3304809	4.34E-06	-0.0749914	0.31034013	-0.4371185	4.94E-10
-0.0802293	0.27765975	-0.0418998	0.57119963	-0.2802918	0.00011141
-0.1624496	0.02715563	0.00675587	0.92727964	-0.1191259	0.10629729
-0.4262139	1.46E-09	-0.236645	0.00118239	-0.3366127	2.80E-06
-0.3424838	1.83E-06	-0.1686141	0.02177286	-0.5097514	1.26E-13
-0.2007716	0.00613944	-0.1049053	0.15528193	-0.1207468	0.10158906
0.36967041	2.24E-07	0.18694372	0.01083491	0.41108203	6.16E-09
-0.3672739	2.71E-07	-0.1734425	0.01822543	-0.1442314	0.05014601
-0.3664504	2.90E-07	-0.1766143	0.01617829	-0.0629928	0.39430643
0.31373925	1.37E-05	0.05506923	0.45656746	0.18075312	0.01381031
-0.2444434	0.00079862	-0.1587289	0.03092681	-0.288129	6.98E-05
0.01098071	0.88206969	-0.1168034	0.11334059	-0.2170659	0.00299868
-0.3181529	1.02E-05	-0.1629544	0.02667559	-0.2547372	0.00046661
-0.4270078	1.35E-09	-0.2875137	7.24E-05	-0.2682595	0.00022259
-0.1734989	0.01818714	-0.0226788	0.75928998	-0.1835133	0.01240514
-0.4724236	1.13E-11	-0.1898742	0.00963511	0.02774742	0.70771999
-0.3830692	7.38E-08	-0.1533961	0.0371034	-0.0199727	0.78728045
-0.2451007	0.0007722	-0.11579	0.11652591	-0.1321003	0.0730619
-0.3385378	2.44E-06	-0.2333129	0.00139291	-0.2319324	0.00148976
-0.1273891	0.0839915	-0.1190358	0.10656405	-0.5074589	1.68E-13
-0.1420978	0.05367608	-0.0462624	0.53176948	-0.0668714	0.36578455
0.33287008	3.66E-06	0.09321734	0.20693111	0.07555926	0.30667991
-0.3588301	5.29E-07	-0.2153618	0.00323991	-0.1236323	0.09361644
-0.3724697	1.78E-07	-0.1297458	0.07837041	0.01933669	0.79390036
-0.426504	1.42E-09	-0.2067499	0.00474853	-0.3912116	3.67E-08
-0.2394388	0.00102881	-0.0521111	0.48114472	-0.323843	6.90E-06
-0.3874069	5.10E-08	-0.1729599	0.01855584	-0.2146181	0.00335057
-0.3296491	4.60E-06	-0.1427959	0.05249911	-0.0581649	0.43161426
-0.1901043	0.00954607	0.0143289	0.84650233	-0.1325266	0.07213273

-0.1456265	0.04794386	0.0399891	0.58889343	-0.3727015	1.75E-07
-0.4526666	9.86E-11	-0.2354554	0.00125395	-0.3050502	2.42E-05
-0.3229281	7.35E-06	-0.0296178	0.68900725	-0.1243659	0.09167107
-0.3862544	5.63E-08	-0.0805106	0.27597342	-0.1254884	0.08875682
-0.1797387	0.0143607	-0.163052	0.02658369	-0.2415443	0.00092541
-0.4223991	2.11E-09	-0.2223001	0.00235589	-0.2892305	6.52E-05
-0.2549224	0.00046203	-0.1208785	0.10121386	-0.3638461	3.56E-07
-0.4952767	7.67E-13	-0.2462768	0.00072691	-0.1488606	0.04314734
-0.4717244	1.22E-11	-0.3014091	3.06E-05	-0.2290156	0.00171493
-0.3727472	1.74E-07	-0.2875484	7.23E-05	-0.2516001	0.00055095
-0.5565509	1.94E-16	-0.3458584	1.42E-06	-0.3179207	1.03E-05
-0.3556524	6.76E-07	-0.1840269	0.01215801	-0.2136928	0.00349303
-0.2643139	0.00027738	-0.0690988	0.34999799	-0.2598593	0.00035416
-0.2811763	0.00010575	-0.1291936	0.07965953	-0.1373113	0.06234772
-0.3354483	3.05E-06	-0.0293608	0.6915674	-0.2941212	4.83E-05
-0.2805872	0.00010949	-0.1451091	0.04875096	-0.2205285	0.0025579
-0.3709736	2.01E-07	-0.1627339	0.02688441	-0.3068265	2.16E-05
0.65070531	1.18E-23	0.35954553	5.00E-07	0.17056774	0.0202715
-0.2462438	0.00072815	-0.2578094	0.00039575	-0.2405811	0.00097147
-0.0924454	0.21073305	-0.1527398	0.03793103	-0.2017368	0.00589277
-0.2680958	0.00022465	0.02232784	0.76290306	-0.2601209	0.00034916
0.21075438	0.00398237	0.18466683	0.01185616	0.35709271	6.05E-07
-0.2740569	0.00016011	-0.2170914	0.00299519	-0.3434254	1.70E-06
0.3168584	1.11E-05	0.16700759	0.02307842	0.21350333	0.00352286
-0.2603252	0.00034529	-0.038599	0.60192183	-0.2322024	0.00147035
-0.2685302	0.00021923	-0.0389817	0.59832207	-0.3422007	1.86E-06
-0.2225435	0.0023293	-0.1278276	0.08292165	-0.0751952	0.30902275
-0.364908	3.28E-07	-0.3143897	1.31E-05	-0.1858654	0.01130847
0.27927483	0.00011827	0.10419089	0.15813013	0.27552622	0.00014711
0.20486298	0.00515345	0.23679882	0.00117342	-0.0214525	0.77193794
-0.531081	7.45E-15	-0.2569072	0.00041545	-0.3073692	2.08E-05
-0.2595406	0.00036035	-0.11587	0.11627186	-0.1677161	0.02249455
-0.4594746	4.74E-11	-0.2155986	0.00320537	-0.3841705	6.72E-08
-0.3742515	1.54E-07	-0.1830407	0.01263644	-0.0670928	0.36419593
-0.1601571	0.0294296	-0.1187522	0.10740674	-0.4329105	7.54E-10
-0.3961911	2.37E-08	-0.3352089	3.10E-06	-0.3830321	7.40E-08
-0.2581788	0.00038794	-0.05215	0.4808175	-0.2011799	0.006034
-0.2073806	0.0046197	-0.1286453	0.08095634	-0.181108	0.01362217
-0.4165349	3.69E-09	-0.0736155	0.31932604	-0.01214	0.86972399
0.55946756	1.26E-16	0.1094959	0.13789498	0.1417968	0.05419029
-0.4089821	7.48E-09	-0.1305861	0.07644126	-0.2238791	0.00218818
-0.3990982	1.83E-08	-0.2929833	5.18E-05	-0.3249218	6.41E-06
-0.2847183	8.57E-05	-0.1165992	0.11397711	-0.187177	0.01073479
-0.358364	5.48E-07	-0.1121471	0.12855492	-0.1356269	0.06566007
-0.3007559	3.19E-05	-0.1906762	0.00932794	-0.2985379	3.67E-05
0.27889995	0.0001209	0.1495882	0.0421255	0.05872478	0.42718662

-0.1831681	0.01257372	-0.2083103	0.00443551	-0.3134528	1.40E-05
-0.3214909	8.11E-06	-0.259859	0.00035417	-0.217603	0.00292608
-0.3984758	1.94E-08	-0.1187667	0.1073635	-0.045787	0.53599935
0.17528522	0.01701035	0.24481287	0.00078367	0.37853649	1.08E-07
-0.2794992	0.00011672	-0.1681484	0.02214458	-0.3673551	2.69E-07
-0.2861522	7.86E-05	-0.1928896	0.00852466	-0.114504	0.12066796
-0.4026471	1.33E-08	-0.3137135	1.37E-05	-0.1925593	0.00864051
-0.083124	0.26063398	-0.1403528	0.05671397	-0.2451797	0.00076908
0.30433676	2.53E-05	0.08519089	0.24892475	0.20955328	0.00419957
0.0264772	0.72052994	-0.055611	0.45214346	-0.2556736	0.00044386
-0.4302038	9.86E-10	-0.2519035	0.00054222	-0.3544517	7.42E-07
-0.4692176	1.62E-11	-0.3425698	1.81E-06	-0.2958246	4.35E-05
0.39546027	2.53E-08	0.18951612	0.00977512	0.21928366	0.0027091
-0.262095	0.00031345	0.01645902	0.82403012	-0.4615073	3.80E-11
-0.2398998	0.00100531	-0.2297641	0.00165436	-0.357892	5.69E-07
-0.2088054	0.00434014	-0.1294666	0.07902015	-0.2075302	0.0045896
-0.2909066	5.89E-05	-0.051794	0.48382117	-0.0913405	0.2162611
-0.4928792	1.03E-12	-0.3244473	6.62E-06	-0.2820797	0.00010025
-0.5652525	5.20E-17	-0.3229506	7.34E-06	-0.2474755	0.00068328
-0.4757002	7.76E-12	-0.2607976	0.00033651	-0.0434488	0.55704332
0.4414925	3.17E-10	0.18021805	0.01409828	0.16222624	0.02737036
-0.2736129	0.00016424	-0.1364001	0.06412216	-0.2290787	0.00170975
-0.4540684	8.49E-11	-0.3282678	5.07E-06	-0.0534748	0.46972643
-0.4942534	8.69E-13	-0.3998424	1.71E-08	-0.2936837	4.96E-05
0.57485322	1.16E-17	0.21841077	0.00281991	0.4352902	5.94E-10
-0.2908053	5.93E-05	-0.2026584	0.00566553	-0.2509754	0.00056934
-0.3526756	8.50E-07	-0.2250491	0.00207099	-0.2433409	0.00084483
-0.3282895	5.07E-06	-0.2335696	0.00137555	-0.3474895	1.26E-06
-0.4040916	1.17E-08	-0.1640028	0.02570197	-0.2536353	0.00049477
-0.3581001	5.60E-07	-0.1194974	0.10520346	-0.0167629	0.82083492
-0.4019391	1.42E-08	-0.1888714	0.01003177	-0.3852991	6.11E-08
-0.3735612	1.63E-07	-0.2590256	0.00037056	-0.0492897	0.5052364
-0.377403	1.19E-07	-0.1410848	0.05542279	-0.2802324	0.0001118
-0.3233901	7.12E-06	-0.2318158	0.00149822	-0.3794526	1.00E-07
-0.3728517	1.73E-07	-0.1933938	0.0083505	-0.0956047	0.19548633
-0.518821	3.87E-14	-0.2962652	4.23E-05	-0.3958168	2.45E-08
-0.3459424	1.41E-06	-0.1509717	0.04023857	-0.3508735	9.75E-07
0.36066115	4.58E-07	0.06486227	0.38039608	0.23200372	0.00148461
0.37027345	2.13E-07	0.06448464	0.3831815	0.17861837	0.0149908
0.18329627	0.0125109	-0.1347748	0.06738999	0.0500988	0.49826372
-0.5377852	2.94E-15	-0.2801563	0.0001123	-0.4794475	5.04E-12
-0.4885178	1.73E-12	-0.3186725	9.83E-06	-0.0895248	0.22556956
-0.3759716	1.34E-07	-0.0905517	0.22027085	-0.1284036	0.08153317
-0.3106161	1.68E-05	-0.2230387	0.00227604	-0.120412	0.10254766
-0.4765496	7.04E-12	-0.2864238	7.73E-05	-0.3444621	1.58E-06
-0.2426966	0.00087295	-0.0761453	0.30293262	-0.3737851	1.60E-07

-0.4270194	1.35E-09	-0.2941004	4.84E-05	-0.2336093	0.00137288
0.44679079	1.83E-10	0.1465859	0.04647666	0.18542835	0.01150555
0.55377297	2.94E-16	0.25063671	0.00057954	0.22914593	0.00170424
-0.2216628	0.00242683	-0.0879698	0.23376389	-0.4216414	2.27E-09
0.44475946	2.26E-10	0.21597721	0.00315084	0.33997653	2.19E-06
0.51749097	4.61E-14	0.27153188	0.00018498	0.25669249	0.00042027
0.65092923	1.13E-23	0.32686052	5.60E-06	0.16265754	0.02695702
0.35186199	9.04E-07	0.2138163	0.0034737	0.20173415	0.00589343
-0.1534807	0.03699781	0.00847881	0.90880606	-0.3235845	7.03E-06
-0.3410137	2.03E-06	-0.1732449	0.01836008	-0.1589377	0.03070386
-0.4308953	9.21E-10	-0.102814	0.16372976	-0.0853024	0.24830345
-0.4116703	5.83E-09	-0.2914521	5.70E-05	-0.2004083	0.00623467
-0.2635344	0.00028958	-0.2044997	0.00523486	-0.2977845	3.84E-05
-0.3934964	3.01E-08	-0.1712308	0.01978272	0.00145279	0.98434165
-0.4123141	5.49E-09	-0.258778	0.00037557	-0.1428773	0.05236329
-0.396073	2.40E-08	-0.3270649	5.52E-06	-0.1999765	0.00634954
0.73674426	6.33E-33	0.38645422	5.53E-08	0.17516662	0.01708638
-0.431624	8.57E-10	-0.0092321	0.90074358	-0.1891099	0.00993615
-0.1260262	0.08738708	0.0307818	0.67745494	-0.2240196	0.0021738
-0.2602303	0.00034708	-0.147894	0.04453683	-0.0181292	0.80650896
-0.3071713	2.11E-05	-0.208819	0.00433754	0.01849765	0.80265587
-0.3513644	9.39E-07	-0.1890589	0.00995652	-0.2147372	0.00333262
-0.0252631	0.73284668	0.05685091	0.44210891	0.16781981	0.02241016
-0.4125794	5.36E-09	-0.305439	2.36E-05	-0.2973068	3.96E-05
-0.2844123	8.73E-05	-0.1768909	0.01600969	-0.2520684	0.00053752
-0.5191633	3.70E-14	-0.3524964	8.62E-07	-0.3979493	2.03E-08
-0.3890637	4.42E-08	-0.2054235	0.00503005	-0.3719065	1.86E-07
-0.3897273	4.17E-08	-0.2089378	0.00431495	-0.016851	0.81990986
-0.2124027	0.00370077	-0.1090184	0.1396311	-0.0638293	0.38804501
-0.1158554	0.11631832	-0.0685986	0.3535055	-0.2188339	0.00276569
-0.2348737	0.00129037	-0.1159763	0.11593532	-0.3624744	3.97E-07
-0.483883	3.00E-12	-0.166292	0.02368129	-0.1213511	0.09987641
-0.3387744	2.40E-06	-0.1486703	0.0434179	-0.260031	0.00035087
-0.4416066	3.13E-10	-0.2848226	8.51E-05	-0.0352526	0.63379952
-0.2313276	0.0015341	-0.1470002	0.04585475	0.07231525	0.32797122
-0.3699773	2.18E-07	-0.1398033	0.05769957	-0.2249352	0.00208214
-0.5110614	1.06E-13	-0.2727287	0.00017277	-0.2638659	0.00028433
-0.3992255	1.81E-08	-0.1851321	0.01164086	-0.3022763	2.89E-05
-0.3670823	2.75E-07	-0.237897	0.00111114	-0.2250302	0.00207284
-0.2990211	3.56E-05	-0.1746316	0.01743305	-0.2260222	0.0019779
-0.2954892	4.44E-05	-0.1327041	0.07174862	-0.165676	0.02421117
0.31007219	1.75E-05	0.08656942	0.24132082	0.24970544	0.00060848
0.54482748	1.08E-15	0.26716565	0.00023667	0.18675017	0.0109186
-0.2347583	0.00129771	-0.19496	0.00782945	-0.0795933	0.28149887
-0.4819238	3.78E-12	-0.2684017	0.00022082	-0.2248708	0.00208847
-0.4273713	1.30E-09	-0.2140118	0.00344331	-0.1150248	0.11897708

-0.2029264	0.00560094	-0.1217762	0.09868535	-0.2449277	0.00077907
0.32957947	4.63E-06	0.11850915	0.10813326	0.17061812	0.020234
0.68327984	8.67E-27	0.37454465	1.50E-07	0.2484837	0.00064847
-0.0817897	0.26839096	-0.1500896	0.04143326	-0.2918546	5.56E-05
-0.3504822	1.00E-06	-0.3374733	2.63E-06	-0.3199549	9.01E-06
0.38229099	7.88E-08	0.19467887	0.0079208	0.26440031	0.00027605
-0.1832192	0.01254864	-0.0248996	0.73654783	-0.4268238	1.37E-09
-0.2519306	0.00054144	-0.0525654	0.4773249	-0.2391209	0.00104532
-0.2304871	0.00159772	-0.063695	0.38904563	-0.2416434	0.00092079
-0.2804374	0.00011046	-0.2981865	3.75E-05	-0.3304376	4.36E-06
-0.3900946	4.04E-08	-0.2910819	5.83E-05	-0.2449547	0.000778
-0.2380811	0.001101	-0.0451948	0.54129121	-0.2124811	0.00368782
-0.1262577	0.08680255	-0.0239473	0.74627329	-0.2929174	5.21E-05
-0.157628	0.03212474	-0.1645252	0.02522828	-0.1810429	0.01365652
-0.4354724	5.83E-10	-0.2253535	0.00204145	-0.3781689	1.11E-07
-0.2428521	0.00086609	-0.106816	0.14785453	-0.2698973	0.00020296
-0.3230423	7.29E-06	-0.1887266	0.01009019	-0.2473737	0.00068689
-0.1445106	0.04969865	-0.005003	0.94611452	-0.4791318	5.23E-12
-0.0613576	0.40672019	0.02432185	0.74244341	-0.4357449	5.68E-10
-0.4466275	1.86E-10	-0.2815426	0.00010349	-0.0360515	0.62612468
-0.434144	6.66E-10	-0.1418222	0.05414675	-0.2963095	4.22E-05
-0.3806223	9.07E-08	-0.1604279	0.02915271	-0.3198262	9.09E-06
-0.3689893	2.36E-07	-0.2596913	0.00035741	-0.1340204	0.06895216
-0.0344455	0.6415932	-0.1093187	0.13853711	-0.1655757	0.0242984
-0.3883441	4.70E-08	-0.3535316	7.96E-07	-0.1404485	0.05654375
-0.2239408	0.00218185	-0.0945291	0.20058461	-0.3265018	5.74E-06
-0.427256	1.32E-09	-0.2302	0.00162	-0.2455533	0.00075447
-0.2301409	0.00162462	0.01141146	0.87747914	-0.1224346	0.09686315
-0.1831467	0.01258422	-0.0992354	0.17897487	-0.1765547	0.01621487
-0.5600267	1.15E-16	-0.3721292	1.83E-07	-0.2376132	0.00112694
0.13911576	0.05895246	-0.0959262	0.19398097	0.16225885	0.02733892
-0.3381221	2.51E-06	-0.3718098	1.88E-07	0.14279765	0.05249617
0.18151831	0.01340744	0.07066632	0.33914965	0.32110307	8.33E-06
0.44929286	1.41E-10	0.13140498	0.07459834	0.16048569	0.02909397
-0.4311824	8.95E-10	-0.3863341	5.59E-08	-0.3707479	2.05E-07
-0.2941496	4.82E-05	-0.1247824	0.09058088	-0.2234625	0.00223134
-0.2111066	0.00392061	-0.2287276	0.00173877	-0.1951739	0.00776058
-0.3647473	3.32E-07	-0.2500808	0.00059666	-0.3492687	1.10E-06
-0.1651016	0.02471428	-0.0686517	0.35313199	-0.3434887	1.70E-06
-0.1064399	0.14929511	-0.1883334	0.01025043	-0.2899055	6.26E-05
-0.1718876	0.01930868	-0.208253	0.00444666	0.16247725	0.02712914
-0.3852714	6.12E-08	-0.299013	3.56E-05	-0.2586236	0.00037872
-0.3063982	2.22E-05	-0.1637316	0.02595086	-0.3839577	6.84E-08
-0.3473202	1.27E-06	-0.1223546	0.09708313	-0.3020123	2.94E-05
-0.2393525	0.00103326	0.00755039	0.91875527	-0.4227894	2.03E-09
-0.1711476	0.01984351	-0.1857519	0.01135935	-0.3536227	7.91E-07

-0.2832183	9.37E-05	-0.0643098	0.38447545	-0.0717568	0.33173011
-0.3969863	2.21E-08	-0.2525286	0.00052462	-0.1309918	0.07552361
-0.4724512	1.12E-11	-0.3116811	1.57E-05	-0.4283748	1.18E-09
-0.2718405	0.00018176	-0.0832105	0.26013661	-0.1572127	0.03258674
-0.2908534	5.91E-05	0.04798677	0.5165707	-0.1193838	0.10553704
-0.5472808	7.59E-16	-0.3675436	2.65E-07	-0.3344816	3.27E-06
0.29141974	5.71E-05	0.07278582	0.32482512	0.01779083	0.81005092
-0.280404	0.00011068	-0.1644646	0.02528281	-0.1787918	0.0148917
-0.3077886	2.03E-05	-0.1392259	0.05875035	-0.0879857	0.23367933
0.23555235	0.00124797	-0.050463	0.49514217	0.371191	1.98E-07
-0.6037227	9.37E-20	-0.3546423	7.31E-07	-0.2457442	0.00074711
0.67955891	2.07E-26	0.34432347	1.59E-06	0.13611397	0.06468784
-0.2202831	0.00258708	-0.08953	0.22554245	-0.2137162	0.00348936
-0.3979313	2.03E-08	-0.2206049	0.00254887	-0.2407806	0.00096175
-0.2731061	0.00016908	-0.2211731	0.00248265	-0.1714815	0.01960061
-0.2781033	0.00012666	-0.0031868	0.96566068	-0.2434835	0.00083871
-0.3797595	9.75E-08	-0.2081324	0.00447023	-0.3352437	3.09E-06
-0.43071	9.38E-10	-0.3986569	1.91E-08	0.03467565	0.63936694
-0.342849	1.78E-06	-0.3464101	1.36E-06	-0.305989	2.28E-05
-0.2953305	4.48E-05	-0.2286306	0.00174687	-0.2452126	0.00076778
-0.3127786	1.46E-05	-0.0741492	0.31582025	-0.2297468	0.00165574
-0.3284143	5.02E-06	-0.1559097	0.03407317	-0.2557371	0.00044236
-0.2295379	0.00167246	-0.0708883	0.33763096	-0.3018399	2.97E-05
-0.3002462	3.29E-05	-0.1380178	0.06099965	-0.5247157	1.77E-14
0.30045306	3.25E-05	0.32341222	7.11E-06	0.16565942	0.02422554
-0.3708595	2.03E-07	-0.1509828	0.04022375	-0.4084872	7.83E-09
-0.4432481	2.64E-10	-0.3136462	1.38E-05	-0.4714315	1.26E-11
-0.2660566	0.00025179	-0.1981793	0.00684825	-0.3573589	5.93E-07
-0.4090871	7.41E-09	-0.2068797	0.00472174	-0.2426377	0.00087557
-0.2982906	3.72E-05	-0.1747054	0.01738486	-0.3670417	2.76E-07
-0.2351863	0.00127068	-0.1068005	0.14791387	-0.203295	0.00551318
-0.1778798	0.0154193	-0.2276418	0.00183139	-0.3053832	2.37E-05
-0.3392744	2.31E-06	0.01672465	0.82123736	-0.1442636	0.05009428
-0.4702998	1.43E-11	-0.3424177	1.83E-06	-0.284512	8.67E-05
-0.3159195	1.18E-05	-0.1776116	0.0155775	-0.2497244	0.00060788
-0.3434828	1.70E-06	-0.2087836	0.0043443	-0.0717257	0.33194014
-0.3545002	7.39E-07	-0.298677	3.63E-05	-0.2426139	0.00087662
0.31866542	9.84E-06	0.06994336	0.34412629	0.17078627	0.02010926
-0.3603065	4.71E-07	-0.1885894	0.01014588	-0.0179336	0.80855613
-0.1997088	0.00642172	-0.3494634	1.08E-06	-0.2709685	0.000191
-0.3830367	7.40E-08	-0.2171381	0.00298883	-0.2368278	0.00117173
-0.2689074	0.00021462	-0.1223984	0.09696253	-0.2611151	0.00033073
-0.4459868	1.99E-10	-0.1316018	0.07416081	-0.2089728	0.00430832
-0.2384924	0.00107866	-0.163769	0.02591639	-0.2677153	0.0002295
-0.1799826	0.01422665	-0.1690542	0.02142648	-0.272102	0.00017907
-0.2830216	9.48E-05	-0.1143993	0.12101029	-0.2100991	0.00409958

-0.1855549	0.01144819	0.01598042	0.82906738	-0.1097571	0.13695225
-0.5385851	2.63E-15	-0.3108085	1.66E-05	-0.1290998	0.07988011
-0.2346679	0.00130348	-0.2868355	7.54E-05	0.21140251	0.00386942
-0.4265907	1.41E-09	-0.3340577	3.37E-06	-0.2234591	0.00223169
-0.3394509	2.28E-06	-0.1834204	0.01245031	-0.4381124	4.47E-10
-0.3385051	2.44E-06	-0.1939171	0.00817308	-0.2375516	0.00113039
0.25407898	0.00048325	0.18210225	0.01310697	0.17295916	0.01855635
-0.269316	0.00020973	-0.15318	0.03737414	-0.1827708	0.01277021
-0.2085792	0.00438348	-0.0371625	0.61551869	-0.0846634	0.25187788
-0.2774926	0.00013125	-0.1021108	0.16664622	-0.0719396	0.33049688
-0.3884889	4.65E-08	-0.1729208	0.01858283	0.01761386	0.81190505
-0.430222	9.84E-10	-0.2600465	0.00035057	-0.0144426	0.84529989
-0.1433491	0.05158172	-0.0780644	0.29087374	-0.2405063	0.00097513
-0.2827615	9.63E-05	-0.0701374	0.3427863	-0.2457578	0.00074658
-0.2780392	0.00012713	-0.2258557	0.00199355	-0.1236518	0.09356421
-0.4381715	4.44E-10	-0.1651713	0.02465278	-0.3010881	3.12E-05
-0.1762544	0.01640005	-0.0439702	0.55231582	-0.2185801	0.00279809
-0.4017184	1.45E-08	-0.1525625	0.0381573	-0.2260312	0.00197706
-0.3883438	4.70E-08	-0.1350564	0.06681412	-0.1212974	0.10002759
-0.3513062	9.43E-07	-0.1655964	0.02428038	-0.2739738	0.00016088
-0.2451244	0.00077126	-0.1101552	0.13552508	-0.122484	0.09672739
-0.4270984	1.34E-09	-0.2431826	0.00085166	-0.0970439	0.18881367
-0.3779927	1.13E-07	-0.2306214	0.00158739	-0.0629299	0.39477923
-0.1525776	0.03813799	-0.0873676	0.2369931	-0.2480703	0.00066254
0.32514656	6.31E-06	0.03843712	0.60344707	0.02553705	0.7300617
-0.4869206	2.10E-12	-0.2068461	0.00472866	-0.2533849	0.00050139
0.31080698	1.66E-05	0.23433365	0.00132504	0.24768482	0.00067592
-0.3148253	1.27E-05	-0.1612805	0.02829572	-0.2202449	0.00259165
-0.1470418	0.04579268	-0.253442	0.00049987	-0.2972296	3.98E-05
-0.4758319	7.65E-12	-0.3236951	6.97E-06	-0.3038368	2.62E-05
-0.3279755	5.18E-06	-0.15467	0.0355404	-0.1877816	0.01047908
-0.4473719	1.72E-10	-0.1884008	0.01022282	-0.0510431	0.49019026
-0.4686585	1.72E-11	-0.1679348	0.02231693	-0.2801133	0.00011259
-0.4671423	2.04E-11	-0.3564684	6.35E-07	-0.0369995	0.61706994
-0.3389959	2.36E-06	-0.1778322	0.01544724	-0.2964313	4.18E-05
-0.269387	0.00020889	-0.1311356	0.0752006	-0.2044625	0.00524326
-0.381452	8.46E-08	-0.2409344	0.00095433	-0.3457304	1.44E-06
-0.4096223	7.05E-09	-0.1760521	0.01652585	-0.0330432	0.65522712
-0.290526	6.03E-05	-0.1965715	0.00732369	-0.1758523	0.01665089
0.41335834	4.98E-09	0.11049525	0.13431518	0.15272365	0.03795156
-0.393125	3.11E-08	-0.2682717	0.00022244	-0.2543071	0.00047743
-0.4149614	4.29E-09	-0.2650836	0.00026579	-0.3672286	2.72E-07
-0.3766966	1.26E-07	-0.1751455	0.01709995	-0.2389054	0.00105664
-0.4286221	1.15E-09	-0.3192888	9.43E-06	-0.4420495	2.99E-10
-0.2870389	7.45E-05	-0.2221869	0.00236836	-0.3463253	1.37E-06
0.05405754	0.46489333	-0.169765	0.02087722	-0.0993449	0.17849322

0.65982446	1.71E-24	0.41758762	3.34E-09	0.19230816	0.00872951
-0.2879118	7.07E-05	-0.3464739	1.36E-06	0.18071702	0.01382957
-0.2164168	0.00308858	-0.242975	0.0008607	-0.1151977	0.11841985
0.23903446	0.00104984	0.05767376	0.43552015	0.15081813	0.04044443
-0.3256731	6.08E-06	-0.2136183	0.00350472	-0.2844984	8.68E-05
-0.4579021	5.63E-11	-0.2674037	0.00023354	-0.2444096	0.00080001
-0.1691571	0.0213462	-0.1963654	0.0073867	-0.2355536	0.0012479
-0.3876668	4.99E-08	-0.2036684	0.00542553	-0.0588739	0.42601203
-0.2516013	0.00055091	-0.1709687	0.01997468	-0.4277995	1.25E-09
-0.3616751	4.23E-07	-0.3732691	1.67E-07	-0.4039812	1.18E-08
-0.3874265	5.09E-08	-0.314047	1.34E-05	-0.3629112	3.84E-07
-0.3165912	1.13E-05	-0.1999766	0.00634952	-0.2604451	0.00034305
-0.2514528	0.00055523	-0.0761446	0.30293735	-0.3405453	2.11E-06
-0.5607812	1.03E-16	-0.322456	7.59E-06	-0.2651457	0.00026488
-0.4151138	4.22E-09	-0.0096854	0.89589766	-0.3939804	2.88E-08
0.38225981	7.90E-08	0.20117054	0.00603639	0.18728663	0.01068803
-0.2703559	0.00019776	-0.1372512	0.06246351	-0.0702373	0.34209699
-0.0234122	0.75175587	-0.1599174	0.0296764	-0.2156206	0.00320217
-0.327766	5.26E-06	-0.2654696	0.00026015	-0.2627806	0.00030186
-0.3056602	2.33E-05	-0.0744472	0.31387419	-0.2245337	0.00212189
-0.5401882	2.09E-15	-0.1450283	0.04887803	-0.2685831	0.00021858
-0.1936195	0.00827358	-0.0339924	0.64598638	0.13246684	0.07226239
-0.2940756	4.85E-05	-0.1443111	0.05001791	-0.3699822	2.18E-07
-0.2573929	0.00040474	-0.0220703	0.76555826	-0.1067611	0.1480643
-0.472444	1.12E-11	-0.1159761	0.11593588	-0.1560763	0.03387994
-0.2845848	8.64E-05	-0.2578361	0.00039518	-0.2353768	0.00125882
-0.0536366	0.46838235	-0.0324036	0.66148422	-0.2756245	0.00014628
-0.3845147	6.53E-08	-0.2616879	0.00032052	-0.2546298	0.00046929
-0.3960946	2.39E-08	-0.2098815	0.00413918	-0.3144264	1.31E-05
-0.4730544	1.05E-11	-0.2850499	8.40E-05	-0.1643697	0.02536849
-0.294902	4.60E-05	-0.1546928	0.03551293	-0.3834217	7.16E-08
-0.2283906	0.00176704	-0.1010494	0.1711211	-0.0942034	0.20214689
0.44854991	1.52E-10	0.23899805	0.00105175	0.19150138	0.00902096
-0.1685168	0.02185006	-0.0959569	0.19383801	-0.2150858	0.00328058
-0.4071186	8.87E-09	-0.1053728	0.1534394	-0.2850634	8.39E-05
-0.3246773	6.52E-06	-0.1330017	0.07110845	-0.1024531	0.16522145
-0.1674541	0.02270897	-0.3108461	1.66E-05	0.12486216	0.09037331
-0.4264535	1.43E-09	-0.1189258	0.10689025	-0.1944908	0.00798242
-0.5028008	3.03E-13	-0.2568618	0.00041647	-0.3653163	3.17E-07
-0.2687547	0.00021647	-0.1568639	0.03297914	-0.1728456	0.01863486
-0.1889141	0.01001458	-0.0832702	0.25979377	-0.122246	0.09738244
-0.2860233	7.92E-05	-0.2181048	0.00285971	-0.0811724	0.2720325
-0.34275	1.79E-06	-0.3465812	1.35E-06	-0.238821	0.0010611
0.36627748	2.94E-07	0.08346693	0.2586657	0.1699657	0.02072431
-0.3610284	4.45E-07	-0.2262686	0.00195495	-0.0403749	0.58530064
-0.2534955	0.00049845	-0.1537867	0.03661805	-0.233283	0.00139495

-0.3151878	1.24E-05	-0.0246767	0.7388204	-0.2620344	0.00031449
-0.3370794	2.71E-06	-0.1326221	0.07192585	0.0001893	0.99795956
0.46483218	2.64E-11	0.10953296	0.13776094	0.16948363	0.02109317
-0.3751132	1.43E-07	-0.2600366	0.00035076	-0.3234168	7.11E-06
-0.1070776	0.14685901	-0.1426268	0.05278227	-0.3593782	5.07E-07
-0.1382694	0.06052544	-0.0630614	0.39379046	-0.3234706	7.08E-06
-0.2485002	0.00064792	-0.1681246	0.02216372	-0.297989	3.80E-05
-0.3873938	5.10E-08	-0.1381396	0.06076972	-0.3045573	2.50E-05
-0.4113928	5.98E-09	-0.1723497	0.01898102	-0.104746	0.15591364
0.34346161	1.70E-06	0.13538993	0.06613752	0.39332347	3.05E-08
-0.3052447	2.39E-05	-0.3070183	2.13E-05	-0.3614019	4.32E-07
-0.3074769	2.07E-05	-0.1785825	0.01501137	-0.1707503	0.02013588
-0.273165	0.00016851	-0.1363493	0.0642222	-0.1845939	0.01189024
-0.1175537	0.1110266	-0.0607158	0.41165535	-0.2979522	3.80E-05
0.45280037	9.72E-11	0.20447694	0.00524	0.12867695	0.08088092
-0.3281501	5.12E-06	-0.1310336	0.07542954	-0.1417254	0.05431291
-0.4040033	1.18E-08	-0.2442341	0.00080721	-0.1322355	0.07276629
-0.2204568	0.0025664	-0.1390444	0.05908387	-0.1626606	0.02695413
-0.1422806	0.05336586	-0.1331996	0.0706853	-0.1835708	0.01237728
-0.3702044	2.14E-07	-0.2545489	0.00047132	-0.2386556	0.0010699
-0.3175232	1.06E-05	-0.1410198	0.05553642	-0.205491	0.00501537
0.63018199	7.21E-22	0.38317954	7.31E-08	0.12810471	0.08225137
-0.4122353	5.53E-09	-0.2399629	0.00100212	-0.1731636	0.01841574
-0.2563352	0.00042841	-0.0790413	0.28486001	-0.1977506	0.00697227
0.37256476	1.77E-07	0.11281586	0.12627771	0.24140739	0.00093183
-0.3144936	1.30E-05	-0.1470641	0.04575953	-0.3508341	9.78E-07
-0.3974049	2.13E-08	-0.1271959	0.08446621	-0.22914	0.00170473
-0.3848643	6.34E-08	-0.2356331	0.00124302	-0.0584892	0.42904648
-0.2298246	0.00164956	-0.086232	0.24316687	0.01651207	0.82347211
-0.4173212	3.43E-09	-0.1251935	0.08951516	-0.2058215	0.00494405
0.27926622	0.00011833	0.07118741	0.33559115	0.31378615	1.37E-05
-0.2740307	0.00016035	-0.0995442	0.1776192	-0.3226962	7.47E-06
-0.1925788	0.00863363	-0.2945548	4.70E-05	-0.3418875	1.91E-06
-0.5035361	2.76E-13	-0.3066599	2.18E-05	-0.3402449	2.15E-06
-0.2099886	0.00411964	-0.1796613	0.01440351	-0.1929408	0.00850685
-0.2337013	0.00136672	-0.0420855	0.56949377	-0.3254319	6.18E-06
-0.2186444	0.00278985	-0.0352744	0.6335899	-0.3877841	4.94E-08
-0.2787898	0.00012168	-0.0297614	0.68757819	-0.0150494	0.83888691
-0.3425584	1.82E-06	-0.2299297	0.00164123	-0.2802817	0.00011148
-0.4458162	2.03E-10	-0.2309843	0.0015598	-0.3326914	3.71E-06
-0.3252745	6.25E-06	-0.1371089	0.06273836	-0.2104626	0.00403418
-0.2055581	0.00500081	-0.1941	0.00811189	-0.255488	0.00044829
-0.2611961	0.00032927	-0.1617986	0.02778551	-0.1618608	0.02772477
-0.2168886	0.003023	-0.2048549	0.00515524	-0.2560822	0.00043426
-0.2574083	0.0004044	-0.2003577	0.00624804	-0.1508055	0.04046145
-0.4753485	8.08E-12	-0.2688269	0.0002156	-0.1535634	0.03689484

-0.2906893	5.97E-05	-0.1016422	0.16861067	-0.2745927	0.00015525
-0.4183358	3.11E-09	-0.2140344	0.00343982	-0.1704719	0.02034303
-0.305324	2.38E-05	-0.3085903	1.92E-05	-0.3158093	1.19E-05
-0.2692316	0.00021073	-0.2871336	7.41E-05	-0.2793983	0.00011742
-0.1772686	0.01578192	-0.1708543	0.02005897	-0.3646471	3.34E-07
-0.4535168	9.01E-11	-0.2670463	0.00023826	-0.3543478	7.48E-07
0.02068776	0.77985601	-0.1710381	0.01992373	-0.2793872	0.00011749
-0.2892373	6.52E-05	-0.1359873	0.0649395	-0.0566929	0.44338068
-0.4189505	2.94E-09	-0.1770231	0.01592963	-0.2146793	0.00334135
-0.2769023	0.00013583	-0.0206174	0.78058552	-0.2849403	8.45E-05
-0.2826345	9.70E-05	-0.0706219	0.33945388	-0.2985497	3.66E-05
-0.3869921	5.28E-08	-0.2377118	0.00112143	-0.0830654	0.26097147
-0.4454685	2.10E-10	-0.3110905	1.63E-05	-0.3006344	3.21E-05
-0.4378335	4.60E-10	-0.2169706	0.00301173	-0.3497178	1.06E-06
-0.006096	0.93436624	0.03772101	0.61021594	-0.4549078	7.76E-11
-0.2441176	0.00081203	-0.2603988	0.00034391	-0.3116236	1.58E-05
-0.3736216	1.62E-07	-0.2996226	3.42E-05	-0.0530956	0.47288679
0.13293466	0.07125229	0.02719289	0.71330251	0.25724129	0.00040805
-0.399615	1.75E-08	-0.1574272	0.03234744	-0.3789186	1.05E-07
-0.2945558	4.70E-05	-0.2131265	0.00358289	-0.2669272	0.00023985
-0.216562	0.00306826	-0.1911867	0.00913694	-0.2424805	0.00088257
-0.4338784	6.84E-10	-0.1475981	0.04496958	-0.1869171	0.01084639
-0.4467005	1.85E-10	-0.3094795	1.81E-05	-0.0541177	0.46439599
0.19838776	0.00678868	0.15168229	0.03929734	0.28392785	8.98E-05
-0.2657981	0.00025544	-0.2131817	0.00357403	-0.2111425	0.00391438
-0.3462543	1.38E-06	-0.1892705	0.00987221	-0.2522945	0.00053115
-0.1786246	0.01498722	-0.0582828	0.43067952	-0.0880827	0.23316199
-0.5550156	2.44E-16	-0.1759359	0.01659845	-0.0797977	0.28026113
-0.386339	5.59E-08	-0.1210375	0.10076239	0.00064687	0.99302755
0.59847965	2.33E-19	0.22201017	0.00238793	0.25447593	0.00047315
-0.1682701	0.02204691	0.0430603	0.56057758	-0.1246627	0.09089316
-0.3168687	1.11E-05	-0.250109	0.00059578	-0.1912479	0.00911431
-0.2110817	0.00392495	-0.0726007	0.32606042	-0.3802154	9.38E-08
-0.4073756	8.67E-09	0.05371012	0.46777165	-0.2480523	0.00066316
-0.6034169	9.88E-20	-0.356898	6.14E-07	-0.2325264	0.00144736
-0.3852882	6.11E-08	-0.1469502	0.04592952	-0.3054992	2.35E-05
-0.3356164	3.01E-06	-0.1659131	0.02400599	-0.5198289	3.39E-14
-0.3227941	7.42E-06	0.00163029	0.98242881	0.00958296	0.8969922
-0.1837459	0.01229267	-0.0437911	0.55393743	-0.1718978	0.01930139
-0.3781565	1.11E-07	-0.2624047	0.00030817	0.02308408	0.75512342
-0.4039659	1.18E-08	-0.2858461	8.01E-05	-0.0158368	0.83058002
0.26685901	0.00024076	0.15902832	0.03060763	0.34592776	1.41E-06
-0.2250986	0.00206616	-0.1977467	0.00697341	-0.1894329	0.00980792
-0.2811529	0.0001059	-0.012175	0.86935211	-0.2543712	0.0004758
-0.3433614	1.71E-06	-0.3548955	7.17E-07	-0.1735675	0.01814071
-0.2168592	0.00302704	-0.0326973	0.65860857	-0.1754417	0.01691046

0.19703336	0.0071842	0.08600329	0.24442375	0.33746664	2.63E-06
0.34242956	1.83E-06	0.12643056	0.08636827	0.08497952	0.25010516
-0.4316266	8.56E-10	-0.1895574	0.00975887	-0.2525673	0.00052355
-0.2191059	0.00273134	-0.1200883	0.10348145	-0.2633047	0.00029327
-0.2471186	0.00069601	-0.0062192	0.93304237	-0.0812828	0.2713789
-0.3227944	7.42E-06	-0.2010559	0.00606584	-0.2116608	0.00382522
-0.1766114	0.01618009	-0.1552143	0.03488969	-0.3144596	1.31E-05
-0.4867134	2.15E-12	-0.207817	0.00453241	-0.257624	0.00039973
-0.4739802	9.45E-12	-0.2290202	0.00171456	-0.1341835	0.06861201
-0.31183	1.55E-05	-0.1668247	0.02323128	-0.0881054	0.23304148
-0.060193	0.41570112	0.06763323	0.36033639	-0.2267822	0.00190787
0.41848169	3.07E-09	0.21058159	0.00401298	0.17107444	0.01989706
-0.3927214	3.22E-08	-0.2198321	0.00264151	-0.2320829	0.00147891
-0.108622	0.14108466	-0.0677996	0.35915364	-0.3014632	3.05E-05
0.25615862	0.00043248	-0.0284501	0.70066836	0.20903237	0.00429703
-0.4364047	5.31E-10	-0.2090639	0.00429109	-0.3521331	8.86E-07
-0.2181759	0.00285042	-0.099068	0.17971247	-0.3294203	4.68E-06
0.50775846	1.62E-13	0.29343007	5.04E-05	0.3332894	3.56E-06
0.35587138	6.65E-07	0.1454363	0.04823922	0.13352151	0.07000143
-0.3483706	1.18E-06	-0.1310924	0.07529748	-0.1621489	0.02744503
-0.1867979	0.01089792	-0.3054583	2.36E-05	-0.2363952	0.0011971
0.26805827	0.00022512	0.14375568	0.0509159	0.29708917	4.02E-05
0.28080891	0.00010807	0.01541669	0.83500975	0.12897696	0.08016981
0.62647033	1.47E-21	0.27294271	0.00017067	0.24738992	0.00068632
-0.3320901	3.87E-06	-0.191046	0.00918923	-0.255955	0.00043723
-0.1446986	0.04939934	0.05849104	0.42903191	-0.1656829	0.02420518
-0.3022421	2.90E-05	-0.2598399	0.00035454	-0.4023957	1.36E-08
-0.4128238	5.24E-09	-0.2390032	0.00105148	-0.0908109	0.21894746
-0.3887318	4.55E-08	-0.2500461	0.00059774	-0.0509755	0.49076633
0.69580661	4.18E-28	0.2965454	4.15E-05	0.14195064	0.05392698
-0.1955377	0.00764469	-0.03509	0.63536636	-0.1021566	0.16645513
-0.1663311	0.02364805	-0.2630706	0.00029708	-0.2084048	0.00441715
-0.3979569	2.03E-08	-0.2653111	0.00026245	-0.2730991	0.00016915
-0.3193852	9.37E-06	-0.2776447	0.00013009	-0.4263227	1.44E-09
-0.2644999	0.00027453	-0.1238674	0.0929894	-0.0025352	0.97267859
-0.3013233	3.07E-05	-0.1309703	0.0755721	-0.091866	0.2136189
-0.271652	0.00018372	-0.1205199	0.10223791	-0.2968627	4.07E-05
-0.2679416	0.0002266	-0.0946455	0.20002839	-0.0486038	0.5111869
-0.2797453	0.00011505	-0.2727643	0.00017242	-0.2561606	0.00043244
-0.4469898	1.79E-10	-0.2111823	0.00390746	-0.167766	0.0224539
-0.4105122	6.49E-09	-0.2080889	0.00447876	-0.0163321	0.82536548
-0.1959833	0.00750481	0.02211667	0.76507978	0.01808209	0.80700159
-0.2309652	0.00156124	-0.1343269	0.06831386	-0.2554071	0.00045023
-0.4290989	1.10E-09	-0.1609893	0.02858602	-0.2239629	0.00217959
-0.4318173	8.40E-10	-0.1246134	0.09102193	-0.2805428	0.00010978
-0.316589	1.13E-05	-0.0448747	0.5441623	-0.344959	1.52E-06

-0.3906138	3.87E-08	-0.1420022	0.05383891	-0.1534067	0.0370901
-0.2265479	0.00192922	-0.1537526	0.03666015	-0.2356945	0.00123926
-0.1636421	0.02603348	-0.2146153	0.00335099	-0.237652	0.00112476
0.46123925	3.91E-11	0.90565335	4.21E-70	-0.0641887	0.38537339
0.57743217	7.69E-18	0.95496766	1.69E-98	0.02374458	0.74834869
0.77190227	7.61E-38	0.79082946	7.09E-41	-0.0647549	0.38118695
0.60170672	1.33E-19	0.93964547	3.62E-87	-0.0215739	0.77068318
0.58709484	1.59E-18	0.97531996	5.39E-122	0.03475884	0.63856299
0.88761693	1.61E-63	0.70009399	1.43E-28	0.0416728	0.57328894
0.55914029	1.32E-16	0.94245049	5.29E-89	0.00262748	0.97168469
0.56252099	7.90E-17	0.93887543	1.11E-86	0.04769053	0.51916587
0.81386156	5.14E-45	0.89282112	2.69E-65	-0.0412593	0.57710285
0.62498697	1.94E-21	0.93137701	3.12E-82	0.07209837	0.3294278
0.58104242	4.30E-18	0.95001757	1.87E-94	-0.0135666	0.85457516
0.5513186	4.22E-16	0.96314723	2.65E-106	0.02478518	0.73771448
0.50026344	4.15E-13	0.94597215	1.93E-91	0.01614669	0.8273166
0.53644499	3.54E-15	0.9646869	5.73E-108	-0.0185501	0.80210762
0.74032678	2.17E-33	0.88969429	3.22E-64	-0.0883849	0.23155635
0.68900681	2.21E-27	0.91703535	5.58E-75	0.00739517	0.9204199
0.46034854	4.31E-11	0.53251212	6.12E-15	0.28194954	0.00010103
0.53442441	4.70E-15	0.96773608	1.70E-111	-0.0429113	0.5619362
0.61151562	2.34E-20	0.97276662	3.92E-118	0.00965912	0.89617809
0.89065278	1.52E-64	0.83558527	1.74E-49	0.04051401	0.58400772
0.68968863	1.87E-27	0.95410225	9.24E-98	0.07476069	0.3118349
0.8368286	9.24E-50	0.90435453	1.38E-69	0.06025846	0.41519294
0.61138212	2.40E-20	0.97251435	9.01E-118	0.03327165	0.65299848
0.8514966	3.40E-53	0.83153382	1.33E-48	0.08555022	0.24692685
0.50927635	1.34E-13	0.87844168	1.37E-60	-0.0039017	0.95796347
0.73528516	9.74E-33	0.64722277	2.43E-23	-0.1558238	0.03417316
0.61968008	5.26E-21	0.97817025	8.14E-127	0.01666693	0.82184399
0.56221203	8.28E-17	0.97456314	8.25E-121	-0.0087084	0.90634809
0.71180541	6.88E-30	0.93894252	1.01E-86	0.01021307	0.89026026
0.4733044	1.02E-11	0.92154748	4.13E-77	-0.0209516	0.77712117
0.48871201	1.69E-12	0.90090199	3.02E-68	-0.0439507	0.55249264
0.44528082	2.14E-10	0.93427048	6.93E-84	-0.0266388	0.7188954
0.60258885	1.14E-19	0.96512121	1.88E-108	0.0121907	0.86918503
0.55956794	1.24E-16	0.93595093	7.00E-85	0.07394541	0.31715589
0.5333719	5.43E-15	0.93493236	2.83E-84	-0.0343296	0.64271617
0.64735248	2.36E-23	0.95682926	3.85E-100	0.03462064	0.63989879
0.69261315	9.19E-28	0.94688713	4.21E-92	0.05293021	0.47426869
0.64354242	5.14E-23	0.97041626	6.86E-115	0.02826162	0.70255741
0.62297009	2.84E-21	0.95420788	7.52E-98	-0.011173	0.88001994
0.59298718	5.95E-19	0.97682647	1.81E-124	0.00478684	0.94843905
0.52178809	2.61E-14	0.96216787	2.79E-105	-0.01601	0.82875635
0.89051184	1.69E-64	0.78142577	2.48E-39	0.05792772	0.43349798
0.59726134	2.87E-19	0.98251329	1.52E-135	0.01354482	0.85480652

0.61424113	1.43E-20	0.95475561	2.57E-98	0.01804383	0.80740194
0.64038102	9.71E-23	0.97584197	7.80E-123	0.0441921	0.55031018
0.82985184	3.03E-48	0.8463891	5.86E-52	0.16812233	0.02216555
0.58681116	1.67E-18	0.94444294	2.31E-90	0.02893561	0.69581114
0.59071808	8.72E-19	0.9521358	3.92E-96	0.04172541	0.57280447
0.62278586	2.94E-21	0.93494449	2.78E-84	-0.0154598	0.83455476
0.76150668	2.66E-36	0.87700136	3.75E-60	-0.0069927	0.92473797
0.60336323	9.97E-20	0.93908312	8.24E-87	0.035552	0.63091886
0.5810705	4.28E-18	0.9579763	3.46E-101	0.05113089	0.48944372
0.60391292	9.06E-20	0.94503846	8.85E-91	0.02346322	0.75123232
0.56051584	1.07E-16	0.90924946	1.42E-71	-0.0724259	0.32722971
0.67102014	1.46E-25	0.92878407	8.24E-81	0.0223899	0.76226376
0.58696415	1.63E-18	0.95588473	2.67E-99	0.01665603	0.82195861
0.60946138	3.39E-20	0.92156046	4.07E-77	-0.0149812	0.83960696
0.53147351	7.06E-15	0.92261902	1.23E-77	0.00305578	0.96707153
0.85643495	1.95E-54	0.71335471	4.56E-30	-0.0042997	0.95368047
0.30832722	1.96E-05	0.86879694	9.34E-58	-0.0711957	0.33553478
0.55979568	1.19E-16	0.94813922	5.02E-93	0.01047322	0.88748323
0.46052759	4.23E-11	0.94782186	8.65E-93	-0.0253992	0.73146257
0.54171046	1.69E-15	0.95188886	6.21E-96	0.01214105	0.86971307
0.5990396	2.12E-19	0.96603631	1.72E-109	0.00488027	0.94743409
0.60917302	3.57E-20	0.97064993	3.36E-115	0.01369675	0.85319617
0.6042773	8.50E-20	0.96061955	1.02E-103	0.02599266	0.7254372
0.58499218	2.26E-18	0.97363901	2.07E-119	0.04839275	0.51302506
0.57578897	1.00E-17	0.97622558	1.83E-123	0.02011251	0.78582725
0.50229007	3.23E-13	0.94418827	3.47E-90	-0.0247305	0.73827214
0.49208293	1.13E-12	0.94357887	9.10E-90	0.00706619	0.92394921
0.56853924	3.13E-17	0.9685369	1.77E-112	0.03259004	0.65965829
0.52404077	1.93E-14	0.94289593	2.65E-89	-0.0380769	0.60684771
0.58545789	2.09E-18	0.96540994	8.92E-109	0.02248045	0.76133127
0.59729201	2.86E-19	0.97722962	3.70E-125	-0.0085887	0.90762933
0.52947946	9.27E-15	0.95834741	1.56E-101	0.0058571	0.93693251
0.6034831	9.77E-20	0.96793159	9.82E-112	0.02802199	0.70496164
0.47615018	7.37E-12	0.94584864	2.36E-91	-0.0092949	0.90007189
0.43328396	7.26E-10	0.93863742	1.57E-86	0.00543458	0.94147367
0.83845074	4.01E-50	0.71419735	3.64E-30	0.10971786	0.13709364
0.60536841	7.01E-20	0.97071724	2.73E-115	0.02363657	0.74945524
0.59590889	3.62E-19	0.96634307	7.62E-110	-0.0081187	0.91266333
0.35417726	7.58E-07	0.65974113	1.75E-24	-0.0925007	0.21045902
0.6510428	1.10E-23	0.93535341	1.59E-84	0.00250425	0.97301219
0.62827992	1.04E-21	0.9788684	4.29E-128	0.00928567	0.9001709
0.54204311	1.61E-15	0.93026144	1.30E-81	-0.0628642	0.39527426
0.55701718	1.81E-16	0.94751646	1.46E-92	0.01252195	0.86566356
0.62645136	1.47E-21	0.96416912	2.12E-107	0.03213762	0.6640936
0.56006108	1.15E-16	0.95639155	9.51E-100	0.0338802	0.64707556
0.57450354	1.23E-17	0.97976388	8.50E-130	0.00720289	0.92248248

0.55265638	3.46E-16	0.95976338	7.03E-103	0.0071365	0.92319478
0.6662132	4.26E-25	0.97832725	4.24E-127	0.01017261	0.8906923
0.6672811	3.36E-25	0.90402579	1.86E-69	0.09381886	0.20400307
0.5196777	3.46E-14	0.94952942	4.46E-94	0.01408834	0.84904838
0.58643063	1.78E-18	0.92542211	4.80E-79	0.00588402	0.93664322
0.53382791	5.10E-15	0.95733473	1.34E-100	-0.019316	0.79411628
0.64068944	9.13E-23	0.94786332	8.06E-93	0.00130877	0.98589368
0.52048431	3.11E-14	0.95923711	2.26E-102	-0.0155332	0.8337811
0.50847309	1.48E-13	0.94635581	1.02E-91	-0.0287594	0.697573
0.59069221	8.75E-19	0.9723198	1.70E-117	0.02537913	0.73166687
0.46232635	3.47E-11	0.93894728	1.00E-86	-0.0294096	0.69108121
0.52178606	2.61E-14	0.94778989	9.14E-93	0.04580087	0.53587562
0.51452971	6.79E-14	0.96630653	8.40E-110	0.0213059	0.77345381
0.51418181	7.10E-14	0.9547494	2.60E-98	-0.0212114	0.77443187
0.67082814	1.52E-25	0.94348249	1.06E-89	0.01594051	0.82948778
0.57849695	6.48E-18	0.96441955	1.13E-107	0.01784305	0.80950394
0.61089387	2.62E-20	0.96823579	4.16E-112	0.03285626	0.65705384
0.61165746	2.28E-20	0.96852683	1.82E-112	-0.0067043	0.92783376
0.85680776	1.57E-54	0.83335444	5.36E-49	0.16057141	0.02900696
0.57564463	1.02E-17	0.96667099	3.16E-110	0.0123087	0.86793036
0.58782258	1.41E-18	0.96171852	8.06E-105	0.02582104	0.72717801
0.55787332	1.59E-16	0.94740012	1.77E-92	0.01780315	0.80992189
0.57306933	1.54E-17	0.96070485	8.42E-104	0.0593558	0.42222784
0.60379635	9.25E-20	0.97100229	1.13E-115	0.00348572	0.96244177
0.56939507	2.74E-17	0.95618599	1.45E-99	0.04554311	0.53817555
0.62290787	2.88E-21	0.97391988	7.88E-120	0.04455467	0.54704054
0.62949926	8.22E-22	0.9485345	2.54E-93	-0.0250729	0.73478309
0.57838141	6.61E-18	0.94680983	4.79E-92	-0.0182793	0.80493881
0.87133465	1.77E-58	0.82911595	4.34E-48	-0.0240733	0.74498369
0.77322727	4.77E-38	0.87832516	1.48E-60	0.07802634	0.2911098
0.56881366	3.00E-17	0.95176621	7.80E-96	0.01058367	0.88630455
0.59688547	3.06E-19	0.9725841	7.16E-118	0.00474653	0.94887258
0.59341094	5.54E-19	0.95743023	1.10E-100	0.04012335	0.58764214
0.45162912	1.10E-10	0.91934676	4.68E-76	-0.0260848	0.72450338
0.53620243	3.67E-15	0.94737845	1.84E-92	0.0092093	0.90098772
0.56745422	3.70E-17	0.97261098	6.56E-118	0.00587806	0.93670722
0.58831682	1.30E-18	0.9743935	1.50E-120	0.04289528	0.56208227
0.64437043	4.35E-23	0.96841133	2.53E-112	0.04209659	0.56939208
0.61967012	5.27E-21	0.95970079	8.09E-103	0.04337966	0.55767118
0.60453852	8.11E-20	0.98059545	1.90E-131	0.02157009	0.77072217
0.36544794	3.14E-07	0.87060404	2.86E-58	-0.0288535	0.69663181
0.57089583	2.17E-17	0.93564626	1.07E-84	-0.0642262	0.3850951
0.59207512	6.94E-19	0.96926999	2.11E-113	0.01174423	0.87393548
0.52883436	1.01E-14	0.91361198	1.92E-73	0.02293529	0.75665215
0.57921625	5.77E-18	0.95881545	5.67E-102	0.03353862	0.65039737
0.57237907	1.72E-17	0.97641664	8.84E-124	0.05088672	0.4915228

0.62704508	1.31E-21	0.95865509	8.04E-102	0.00771117	0.91703133
0.58162216	3.91E-18	0.95537055	7.54E-99	-0.0555358	0.45275621
0.60731364	4.97E-20	0.9764366	8.19E-124	0.01856537	0.80194826
0.51156995	9.95E-14	0.9595466	1.14E-102	-0.0273545	0.71167441
0.49472727	8.20E-13	0.95378366	1.72E-97	-0.0306986	0.67827792
0.59162419	7.49E-19	0.93782574	5.04E-86	-0.0106252	0.88586126
0.80204969	7.98E-43	0.82400528	5.01E-47	-0.0355913	0.63054143
0.77535607	2.24E-38	0.84771256	2.83E-52	0.06478139	0.38099161
0.58473981	2.35E-18	0.97655578	5.18E-124	0.02016566	0.78527504
0.54383447	1.25E-15	0.93526699	1.79E-84	-0.03173	0.66809987
0.56217601	8.33E-17	0.96148832	1.38E-104	0.06420302	0.38526687
0.64556004	3.41E-23	0.95863939	8.31E-102	0.00280004	0.96982597
0.57413093	1.30E-17	0.91075902	3.29E-72	0.0271582	0.71365219
0.53771658	2.97E-15	0.96067273	9.06E-104	-0.0439646	0.55236667
0.59758597	2.72E-19	0.96544761	8.09E-109	0.03783431	0.60914276
0.57834613	6.64E-18	0.97685557	1.62E-124	0.02457225	0.73988652
0.61899762	5.97E-21	0.98004846	2.36E-130	0.01674659	0.82100682
0.52847322	1.06E-14	0.93877935	1.28E-86	-0.0504429	0.49531411
0.50848726	1.48E-13	0.93925413	6.42E-87	0.00819732	0.91182116
0.57839822	6.59E-18	0.95747334	1.00E-100	0.03675452	0.61940414
0.60801809	4.38E-20	0.96359285	8.89E-107	0.06242747	0.39857216
0.58526772	2.16E-18	0.96255666	1.11E-105	0.05320751	0.47195304
0.60358424	9.60E-20	0.97391532	8.00E-120	0.0192411	0.79489651
0.60559145	6.74E-20	0.9738127	1.14E-119	-0.001827	0.98030889
0.6521841	8.68E-24	0.96365017	7.72E-107	0.03703249	0.61675536
0.5775859	7.51E-18	0.94831339	3.72E-93	0.01960802	0.79107436
0.58500047	2.25E-18	0.970402	7.17E-115	-0.0009696	0.98954951
0.47749623	6.32E-12	0.9319679	1.45E-82	-0.0176719	0.81129644
0.85515929	4.13E-54	0.702578	7.60E-29	0.08254394	0.2639872
0.58419936	2.57E-18	0.95350727	2.92E-97	0.0326617	0.6589568
0.57941906	5.59E-18	0.96654392	4.45E-110	0.03732535	0.61397011
0.82085916	2.17E-46	0.82660385	1.46E-47	0.05614179	0.44783209
0.59053258	8.99E-19	0.97657746	4.76E-124	0.01047076	0.88750947
0.59345264	5.50E-19	0.97522203	7.70E-122	0.03898983	0.59824569
0.51948223	3.55E-14	0.96027226	2.25E-103	0.03854289	0.6024501
0.51541493	6.05E-14	0.50477068	2.36E-13	0.04850054	0.51208576
0.5738488	1.36E-17	0.96181444	6.44E-105	0.04207483	0.56959193
0.58393728	2.68E-18	0.95673617	4.67E-100	0.04382215	0.55365616
0.82348108	6.42E-47	0.89364412	1.38E-65	0.09645746	0.19151221
0.63119885	5.92E-22	0.97647213	7.15E-124	0.04506353	0.54246777
0.52926109	9.55E-15	0.92482539	9.68E-79	0.03850229	0.60283273
0.78594587	4.59E-40	0.85239434	2.04E-53	0.13393062	0.06914004
0.60541051	6.96E-20	0.96816172	5.13E-112	0.02660383	0.7192493
0.6156728	1.10E-20	0.95797174	3.49E-101	-0.038198	0.60570397
0.76081869	3.34E-36	0.92922059	4.79E-81	0.01144799	0.87709001
0.64414022	4.55E-23	0.96096704	4.62E-104	0.01311603	0.85935463

0.54768176	7.17E-16	0.95717149	1.89E-100	-0.0034777	0.96252853
0.62814007	1.07E-21	0.97869358	9.05E-128	0.01160542	0.87541342
0.54742673	7.44E-16	0.95925845	2.15E-102	0.03569732	0.62952259
0.60793289	4.45E-20	0.9710943	8.48E-116	0.01424887	0.84734914
0.57932644	5.67E-18	0.97470438	4.99E-121	0.0552607	0.45500111
0.87284552	6.44E-59	0.81041806	2.32E-44	0.01333064	0.85707769
0.59860114	2.28E-19	0.96007767	3.48E-103	0.02867723	0.69839462
0.5293025	9.50E-15	0.92999894	1.81E-81	0.00856382	0.90789572
0.66289028	8.83E-25	0.91411815	1.15E-73	0.00374369	0.95966438
0.65024357	1.30E-23	0.96279835	6.18E-106	-0.0077079	0.91706656
0.57213532	1.78E-17	0.97164969	1.47E-116	0.0408947	0.58047619
0.22446015	0.00212925	0.18037728	0.01401204	-0.2830814	9.45E-05
-0.2980394	3.78E-05	-0.1192396	0.10596168	-0.3090216	1.87E-05
-0.1761064	0.01649196	-0.1757112	0.01673969	-0.1742469	0.01768604
-0.0922526	0.21169008	-0.037094	0.61616984	0.00101151	0.98909744
-0.0251594	0.73390277	-0.0239137	0.7466167	0.26187188	0.00031731
0.20877841	0.00434529	0.15101955	0.04017458	-0.1846579	0.01186034
0.02152177	0.77122164	0.0101659	0.89076399	-0.077324	0.29548772
-0.0349887	0.63634357	-0.1014502	0.16942089	-0.1703584	0.02042794
-0.1871759	0.01073526	-0.133838	0.06933439	-0.2586895	0.00037737
-0.0043414	0.95323132	-0.0571285	0.4398801	-0.1556635	0.03436037
-0.0732275	0.3218898	-0.0754271	0.30752918	-0.2074961	0.00459644
0.11058672	0.13399114	0.02167838	0.76960335	-0.0891449	0.22755229
-0.1898831	0.00963166	-0.1981893	0.00684538	-0.086218	0.24324346
-0.0296401	0.68878449	-0.0141875	0.84799875	-0.3004476	3.25E-05
-0.0008495	0.99084325	-0.0598492	0.41837428	-0.1526136	0.03809193
0.00788101	0.91521074	-0.0360959	0.62569992	-0.194633	0.00793578
0.09237798	0.21106723	-0.0154783	0.83435948	-0.0061278	0.93402449
-0.1131154	0.1252679	-0.0716445	0.33248963	-0.2994725	3.46E-05
-0.202702	0.00565499	-0.1073103	0.14597755	-0.2185543	0.00280141
0.1296561	0.07857867	0.00555636	0.94016454	-0.2411424	0.00094438
-0.1076004	0.14488458	-0.1668204	0.02323486	-0.0798768	0.27978334
-0.1447024	0.04939333	-0.1653501	0.02449556	-0.1698249	0.02083145
0.06159039	0.40493896	0.0619044	0.40254357	-0.189538	0.00976652
0.00096787	0.98956774	-0.0365876	0.62099726	0.12011284	0.10341053
0.14387293	0.05072524	0.07253438	0.32650375	-0.0617768	0.40351626
0.17554072	0.01684757	0.03295978	0.65604226	0.04756682	0.52025157
0.05747522	0.43710473	0.02362353	0.74958889	-0.111948	0.12923909
-0.1003606	0.17407246	-0.0327863	0.65773755	-0.1992739	0.00654052
0.02486816	0.73686862	-0.0475548	0.5203567	-0.0884776	0.23106548
-0.1662513	0.02371602	-0.1730256	0.01851052	-0.2816095	0.00010308
-0.059564	0.42059916	-0.0401655	0.58724954	-0.1015572	0.16896904
0.1978531	0.00694244	0.08484797	0.25084182	-0.2839606	8.96E-05
0.178544	0.01503347	0.01823463	0.80540569	0.22416505	0.002159
0.07111623	0.33607582	-0.0256204	0.72921531	0.21535031	0.00324159
-0.0759814	0.30397735	-0.1037698	0.15982715	-0.2330803	0.00140881

-0.1229014	0.0955872	-0.152124	0.03872174	-0.0341325	0.64462663
-0.0402398	0.58655771	-0.0390996	0.59721515	-0.1228035	0.09585365
-0.1468495	0.04608013	-0.0142402	0.84744123	-0.2939485	4.88E-05
-0.0315407	0.66996358	-0.0187726	0.79978383	-0.1778131	0.01545847
-0.1229587	0.0954316	-0.1235287	0.09389367	-0.176911	0.0159975
0.08303664	0.26113725	0.05375585	0.46739224	-0.0066314	0.92861583
0.00864246	0.90705373	-0.1150207	0.11899021	-0.228253	0.00177871
-0.0332257	0.65344611	-0.1359291	0.06505543	-0.2617695	0.00031909
-0.1598302	0.02976663	-0.1253805	0.08903359	-0.0938062	0.20406456
0.10494571	0.15512207	0.09458622	0.20031131	-0.1558073	0.03419241
-0.2070668	0.00468339	-0.1933517	0.00836493	-0.2354415	0.00125481
0.01459595	0.84367779	0.03043859	0.68085345	-0.143328	0.05161648
-0.0360296	0.62633521	-0.0493144	0.50502294	-0.2341206	0.00133895
-0.0068115	0.92668208	-0.0108286	0.88369195	-0.3605993	4.60E-07
-0.0672184	0.36329694	-0.0666103	0.36766396	-0.2276786	0.00182818
-0.0138023	0.85207804	-0.0272957	0.71226681	0.08272746	0.26292318
-0.0762745	0.3021107	-0.0609887	0.4095522	-0.1514708	0.03957547
-0.1890143	0.00997436	-0.0807486	0.27455159	-0.2290501	0.0017121
-0.1979341	0.00691894	-0.203237	0.00552689	-0.3111453	1.63E-05
-0.1999808	0.00634841	-0.1345461	0.0678604	-0.0743604	0.31443998
0.01947212	0.79248943	0.00066229	0.99286134	0.00794944	0.91447725
0.08474878	0.2513982	-0.0054391	0.9414248	-0.1604696	0.02911029
-0.1121623	0.1285028	-0.2043291	0.0052735	-0.2358807	0.00122793
-0.1540032	0.03635137	-0.3275901	5.32E-06	0.06655203	0.36808379
-0.0196154	0.79099753	-0.0054109	0.9417284	0.36674526	2.83E-07
-0.0328467	0.65714746	-0.0691886	0.34937067	0.54467196	1.11E-15
-0.2985818	3.66E-05	-0.1218079	0.09859713	-0.5337506	5.15E-15
-0.0361469	0.62521079	-0.0200471	0.78650684	0.71998544	7.55E-31
0.09464589	0.20002629	0.18233362	0.01298956	0.06781962	0.35901117
-0.0018409	0.98015906	0.03554396	0.63099622	0.74252525	1.12E-33
-0.2381956	0.00109474	-0.158999	0.03063872	-0.0335687	0.65010432
0.01391372	0.85089744	0.04918374	0.50615314	-0.1045181	0.15682064
-0.2100248	0.00411307	-0.0955722	0.19563928	-0.3117779	1.56E-05
-0.1078391	0.14398988	-0.1869676	0.01082464	-0.2197332	0.00265358
-0.1807754	0.01379845	-0.1601726	0.02941368	-0.3449091	1.53E-06
-0.1178661	0.11007397	-0.1911071	0.00916649	-0.1715759	0.01953243
-0.0267887	0.71738125	-0.1152982	0.11809688	-0.3421046	1.88E-06
0.27517338	0.00015014	-0.0133035	0.85736595	-0.151937	0.03896446
0.17279902	0.01866714	0.00991107	0.89348581	-0.2210456	0.00249737
-0.2051047	0.00509989	-0.1598173	0.02978007	-0.0944811	0.20081438
0.09081503	0.21892642	-0.045473	0.53880223	-0.1881613	0.01032129
-0.1415616	0.05459493	-0.1896614	0.00971812	-0.304679	2.48E-05
0.11268855	0.12670879	-0.0601157	0.41630085	0.02823235	0.70285098
0.11887087	0.10705351	0.0139625	0.85038088	-0.1084162	0.1418442
-0.1465419	0.04654313	-0.0222568	0.7636356	-0.2598502	0.00035434
0.068044	0.35741981	-0.0189022	0.79843157	-0.3090931	1.86E-05

-0.1253126	0.08920824	-0.0890219	0.22819702	-0.1717036	0.01944043
-0.1688929	0.02155286	-0.2080027	0.00449571	-0.2246516	0.00211015
0.15586185	0.03412883	0.08141199	0.27061507	-0.3276384	5.30E-06
0.05145354	0.48670361	0.12275292	0.09599158	0.03411371	0.64480873
-0.11168	0.13016423	-0.1663555	0.02362733	-0.3957937	2.46E-08
0.00990091	0.89359436	0.08481078	0.25105032	-0.1514743	0.03957086
0.11237707	0.12776827	-0.0177203	0.81079005	-0.4115186	5.91E-09
0.20956315	0.00419774	0.11056976	0.13405118	-0.1576791	0.03206823
0.09855335	0.18199538	-0.0622834	0.39966357	-0.2856855	8.08E-05
0.00259668	0.97201646	-0.0511094	0.48962664	-0.0791852	0.28398106
-0.2151177	0.00327586	-0.178533	0.01503982	-0.262954	0.000299
0.02684591	0.71680334	-0.0204913	0.78189413	-0.2218535	0.0024054
-0.0541022	0.46452378	-0.1061426	0.15044114	-0.3869979	5.28E-08
-0.1391653	0.05886139	-0.1475458	0.04504636	-0.3053107	2.38E-05
-0.136962	0.06302315	-0.2356194	0.00124386	-0.1560446	0.03391669
-0.1393813	0.05846595	-0.1366875	0.06355808	-0.3455741	1.45E-06
-0.2247343	0.00210194	-0.1047652	0.15583766	-0.2394959	0.00102587
-0.2075492	0.00458579	-0.1631717	0.02647122	-0.2877546	7.14E-05
0.1232026	0.09477111	-0.0033362	0.96405196	-0.4367834	5.11E-10
-0.112455	0.12750263	-0.1498078	0.04182113	-0.2608313	0.00033589
0.14911801	0.04278345	-0.0004055	0.99562888	0.04445815	0.54791004
-0.2373249	0.0011432	-0.1422015	0.05349983	-0.1107267	0.13349648
0.02058698	0.78090123	-0.085206	0.24884043	-0.2847348	8.56E-05
0.17057499	0.0202661	-0.031196	0.67336186	-0.0539574	0.4657217
0.05145958	0.4866524	-0.0589284	0.42558283	-0.2230656	0.00227318
0.08684497	0.23982062	0.02531895	0.73227885	-0.1674657	0.02269944
0.2348283	0.00129325	0.00906526	0.90252864	-0.1988458	0.00665939
-0.0115819	0.87566388	-0.0672778	0.36287203	-0.3308118	4.24E-06
0.17497725	0.01720838	0.00629966	0.93217809	-0.3129744	1.44E-05
-0.0013177	0.98579746	-0.0564593	0.44526473	-0.4285387	1.16E-09
-0.1931681	0.00842807	-0.092993	0.20803084	0.13575049	0.06541231
-0.2268725	0.0018997	-0.2748377	0.00015307	0.39562601	2.49E-08
-0.3274821	5.36E-06	-0.3612314	4.38E-07	0.15955232	0.03005584
0.37245616	1.78E-07	0.1983487	0.00679981	-0.474681	8.72E-12
-0.2767339	0.00013717	-0.1351252	0.06667422	-0.1894856	0.00978714
-0.2861429	7.87E-05	-0.2172778	0.00296985	-0.2965596	4.15E-05
-0.0384961	0.602891	0.31022324	1.73E-05	-0.2860628	7.90E-05
-0.1596466	0.02995744	-0.0424672	0.56599485	-0.3876718	4.98E-08
0.31764147	1.05E-05	0.06960512	0.3464704	0.19786567	0.00693879
-0.1055573	0.15271668	-0.0831891	0.26025944	0.29231904	5.40E-05
-0.2452913	0.00076468	0.02834474	0.70172415	-0.0861691	0.24351213
-0.1465159	0.04658237	-0.1278402	0.08289108	-0.3199896	8.99E-06
0.02241039	0.76205268	-0.0303999	0.68123706	0.24783829	0.00067056
0.35900273	5.22E-07	0.0312904	0.67243073	-0.1414418	0.054802
0.27168755	0.00018335	-0.0309672	0.67562143	0.21477432	0.00332705
-0.0174616	0.81350174	-0.1176133	0.11084433	-0.2352271	0.00126813

0.10965344	0.13732585	-0.0714731	0.33365045	0.09527745	0.19702749
0.35119248	9.52E-07	0.13654403	0.06383916	-0.3679641	2.57E-07
-0.2910591	5.84E-05	-0.0705412	0.34000792	-0.1169095	0.11301122
-0.3139156	1.35E-05	-0.3506014	9.95E-07	0.39360301	2.98E-08
-0.0284347	0.70082226	-0.144474	0.04975712	-0.298976	3.57E-05
-0.1142578	0.12147398	-0.2239654	0.00217934	0.40481219	1.10E-08
0.10272194	0.1641093	-0.0660185	0.3719442	0.4074812	8.58E-09
-0.0404728	0.58439063	-0.2314925	0.00152189	0.19955756	0.00646282
-0.2077186	0.00455195	-0.1193133	0.10574437	-0.3112503	1.62E-05
-0.1955389	0.00764429	0.11438599	0.12105377	-0.2319861	0.00148588
0.17117838	0.01982099	0.00587308	0.93676074	-0.3529726	8.31E-07
-0.3567651	6.21E-07	-0.1376046	0.06178508	0.33756789	2.61E-06
-0.0522367	0.48008707	0.02393746	0.74637397	-0.3207695	8.52E-06
-0.3595561	5.00E-07	-0.2588746	0.00037361	-0.3717793	1.88E-07
0.22832355	0.00177272	0.02464072	0.73918789	-0.5483256	6.53E-16
-0.2570756	0.00041171	-0.3150225	1.26E-05	0.24638809	0.00072275
0.29180907	5.57E-05	0.01939482	0.79329462	-0.4357639	5.66E-10
-0.1121229	0.12863798	-0.1787068	0.01494021	-0.566108	4.56E-17
0.18728712	0.01068782	-0.0022291	0.97597623	0.2390275	0.00105021
-0.3492329	1.10E-06	-0.3544277	7.43E-07	-0.3966346	2.28E-08
-0.3107355	1.67E-05	-0.2825326	9.76E-05	-0.1806902	0.01384391
-0.3194072	9.35E-06	-0.1342987	0.06837238	-0.3364433	2.84E-06
0.58857782	1.25E-18	0.51144776	1.01E-13	-0.2898561	6.28E-05
-0.0315592	0.66978153	-0.1589093	0.03073415	-0.4785828	5.57E-12
0.15788471	0.0318419	-0.0327087	0.65849725	-0.2187397	0.00277768
-0.1021016	0.16668459	-0.1274357	0.0838773	0.09268849	0.20953028
0.119647	0.10476533	0.24378843	0.00082578	-0.2643284	0.00027715
-0.0554806	0.45320552	-0.1189694	0.10676083	0.17393302	0.01789484
-0.2406859	0.00096635	-0.1914045	0.00905655	0.36069793	4.57E-07
0.08175033	0.26862223	0.2133368	0.00354927	0.11133342	0.13136807
0.14546851	0.04818909	-0.0184653	0.8029942	-0.3462833	1.38E-06
-0.146909	0.045991	-0.1086118	0.14112216	-0.4938618	9.11E-13
-0.1689007	0.02154675	0.00305211	0.967111	-0.2275168	0.00184233
0.01127826	0.87889823	-0.0767477	0.29911273	0.1088421	0.14027616
-0.1727516	0.01870004	-0.1755351	0.01685115	0.26263467	0.0003043
-0.2203374	0.0025806	-0.2533538	0.00050221	-0.2794932	0.00011677
-0.1951665	0.00776296	-0.1713555	0.01969192	0.30698976	2.13E-05
-0.0694288	0.34769637	-0.1857534	0.01135872	0.51465371	6.68E-14
0.2875036	7.25E-05	0.17470778	0.01738329	-0.4009992	1.55E-08
-0.2527906	0.00051741	-0.2902647	6.13E-05	0.25687846	0.00041609
-0.1194538	0.10533127	-0.1383118	0.06044591	-0.3611869	4.40E-07
-0.0415038	0.57484603	-0.1398419	0.05762987	-0.0349215	0.63699176
-0.0458319	0.53559935	-0.1497566	0.04189192	0.27035118	0.00019781
-0.3029052	2.78E-05	-0.1266604	0.08579345	-0.1090874	0.13937915
-0.0417203	0.57285159	-0.1116696	0.13020009	0.30893735	1.88E-05
-0.2545585	0.00047108	-0.2455427	0.00075488	-0.5231908	2.17E-14

-0.2449498	0.00077819	-0.2091111	0.00428218	-0.2055771	0.0049967
0.25207169	0.00053743	0.12430009	0.09184417	-0.4791734	5.20E-12
0.01524933	0.8367757	-0.2212658	0.00247199	0.0867471	0.24035271
0.06525883	0.37748424	-0.0914761	0.215577	0.03629431	0.62380012
0.12674278	0.08558821	-0.0380589	0.60701787	-0.4201778	2.61E-09
0.02006888	0.78628066	-0.049585	0.5026853	0.22054889	0.00255549
-0.0863943	0.24227756	-0.1340643	0.06886055	0.1965821	0.00732046
0.16154563	0.02803365	0.1903092	0.00946741	-0.1709111	0.02001708
-0.2703429	0.0001979	-0.1700605	0.0206524	-0.3067578	2.17E-05
-0.2125324	0.00367939	-0.197817	0.00695294	0.13710326	0.06274923
-0.1925634	0.00863905	-0.2793975	0.00011742	0.21659851	0.00306317
-0.1768012	0.01606422	-0.2382751	0.00109041	0.16062542	0.02895226
-0.5141402	7.14E-14	-0.5332449	5.53E-15	0.15199256	0.03889224
0.14184984	0.05409937	0.12552633	0.08865958	-0.2664574	0.00024623
0.09953541	0.17765778	-0.0793625	0.28290131	0.18009615	0.01416462
-0.3691359	2.33E-07	-0.2076945	0.00455675	-0.5425547	1.50E-15
0.03355026	0.65028407	-0.0408533	0.58085966	0.1895849	0.00974809
-0.0492829	0.50529542	-0.1098145	0.13674578	0.22100103	0.00250254
0.32335249	7.14E-06	0.14090398	0.05573949	-0.0162202	0.82654294
-0.1544601	0.03579399	-0.2757285	0.0001454	-0.3015921	3.02E-05
-0.3087628	1.90E-05	-0.308614	1.92E-05	0.05350149	0.46950484
0.0605842	0.41267128	0.03544198	0.63197687	-0.3875827	5.02E-08
0.13693399	0.06307751	-0.1960218	0.00749285	-0.3667009	2.84E-07
0.29302172	5.17E-05	0.11870399	0.10755062	-0.4299286	1.01E-09
-0.4589164	5.04E-11	-0.3925447	3.27E-08	0.32545424	6.17E-06
-0.2157837	0.00317861	-0.237554	0.00113026	0.09662417	0.19074219
0.16494418	0.02485377	0.01380837	0.85201345	-0.106204	0.15020387
-0.298077	3.77E-05	-0.3687691	2.40E-07	-0.370581	2.08E-07
0.21175088	0.0038099	0.0401921	0.58700175	0.26566213	0.00025738
-0.0405684	0.5835028	-0.1070225	0.14706829	0.14347584	0.05137336
-0.0040577	0.95628412	-0.0638084	0.3882003	0.32276176	7.44E-06
-0.0534965	0.46954676	-0.0585217	0.42878974	0.23071242	0.00158043
-0.1496414	0.04205164	-0.1002294	0.17463881	0.26579117	0.00025554
0.03567623	0.62972512	-0.1104924	0.13432512	0.2584489	0.00038231
0.07593194	0.30429345	-0.0474648	0.52114817	0.18553095	0.01145902
-0.1070258	0.1470556	-0.189626	0.00973196	-0.0156956	0.83206894
-0.1595053	0.030105	-0.1586011	0.03106391	-0.140028	0.05729486
-0.4601443	4.41E-11	-0.3551551	7.03E-07	0.34046154	2.12E-06
-0.2092057	0.00426437	-0.2041361	0.00531752	0.18448229	0.01194252
-0.1530408	0.03754949	-0.1294703	0.07901146	-0.1485201	0.04363262
-0.0331019	0.65465414	-0.1714114	0.01965135	0.37793768	1.13E-07
0.10303988	0.16280106	-0.0503809	0.49584442	0.07010374	0.34301828
0.20434409	0.00527009	0.07787092	0.29207482	-0.2695353	0.00020715
0.00741107	0.92024937	-0.1403147	0.05678185	0.15456138	0.03567141
-0.3419215	1.90E-06	-0.1713578	0.01969025	0.03793586	0.60818163
0.15380912	0.03659037	-0.0568734	0.44192836	0.38426884	6.67E-08

-0.2090363	0.00429629	-0.035606	0.63039977	-0.1337474	0.06952477
0.35349849	7.98E-07	0.1087747	0.14052338	-0.1066541	0.14847338
0.2958121	4.35E-05	0.0370693	0.61640502	-0.3375168	2.62E-06
0.09947147	0.17793783	-0.092255	0.21167817	0.1198458	0.10418547
-0.045655	0.53717636	-0.1200451	0.10360672	0.23668368	0.00118013
0.14639743	0.04676187	0.07242166	0.32725808	-0.4190372	2.91E-09
-0.0667528	0.36663727	-0.0024741	0.97333684	-0.456168	6.78E-11
-0.0088789	0.90452247	-0.1010485	0.17112492	0.09936663	0.17839779
-0.0290084	0.69508403	0.05197755	0.48227106	-0.071167	0.33573022
0.10604654	0.15081269	0.02675129	0.71775901	0.29863848	3.64E-05
-0.0788256	0.28618085	-0.1566963	0.03316907	0.09189448	0.21347658
-0.3262975	5.82E-06	-0.1937217	0.00823894	-0.4245991	1.71E-09
0.02854228	0.6997453	-0.0833787	0.25917102	0.17153047	0.01956519
0.16521152	0.02461734	0.05225363	0.47994453	0.37527879	1.41E-07
0.26157751	0.00032247	0.12631873	0.08664906	-0.4026814	1.33E-08
0.15712769	0.032682	-0.0578919	0.43378264	-0.2657364	0.00025632
0.29469285	4.66E-05	0.09133509	0.21628851	-0.2567374	0.00041926
0.05982951	0.41852738	-0.0341798	0.64416793	0.17870691	0.01494014
0.06764286	0.36026787	-0.074354	0.31448174	-0.4776689	6.19E-12
-0.0327809	0.65779071	-0.0925141	0.21039237	0.29865674	3.64E-05
-0.1905148	0.00938904	-0.1628904	0.02673605	-0.4481833	1.58E-10
0.04955159	0.50297382	-0.0117919	0.87342763	0.32384668	6.90E-06
-0.2706873	0.00019408	0.06080886	0.41093729	-0.18468	0.01185
-0.0261196	0.72415005	-0.0804944	0.27606999	0.20945769	0.0042173
-0.0756465	0.30612	0.06971807	0.3456865	-0.2732176	0.00016801
-0.1893384	0.00984527	-0.0397438	0.59118268	-0.3662285	2.95E-07
0.43877846	4.18E-10	0.30701386	2.13E-05	-0.1238667	0.09299122
0.05133925	0.48767331	-0.0515512	0.48587614	0.37900258	1.04E-07
0.10894795	0.13988853	0.1812368	0.01355444	-0.1139061	0.12263236
-0.3103599	1.71E-05	-0.3403254	2.14E-06	0.46890597	1.68E-11
-0.1752717	0.017019	-0.2721192	0.00017889	0.2564428	0.00042594
0.31023087	1.73E-05	0.07667448	0.29957508	0.04759102	0.52003906
-0.2745664	0.00015548	-0.4330376	7.44E-10	0.00732347	0.92118899
-0.0424443	0.5662046	-0.1252342	0.08941003	0.17799906	0.01534937
0.03190782	0.66635088	0.10109526	0.17092586	-0.1777808	0.01547749
-0.1516561	0.03933176	-0.170838	0.02007103	-0.1251399	0.08965341
-0.1690127	0.02145898	-0.3223539	7.65E-06	0.26119722	0.00032925
0.03091706	0.67611739	0.00775582	0.91655262	-0.0286953	0.69821371
-0.2291039	0.00170769	-0.0457106	0.53668074	-0.2550691	0.00045843
-0.2326797	0.00143659	-0.2711625	0.00018891	0.39134632	3.63E-08
0.10468224	0.15616709	-0.0041992	0.95476185	0.3544944	7.40E-07
0.17145073	0.01962287	0.14631499	0.0468871	-0.314656	1.29E-05
-0.3296108	4.62E-06	-0.2361849	0.00120962	0.18679748	0.0108981
-0.0739809	0.31692328	-0.1364839	0.0639573	0.18771289	0.01050788
0.00266065	0.97132741	-0.2424582	0.00088357	0.29928162	3.50E-05
0.03547346	0.63167413	-0.0643456	0.38420996	0.25339454	0.00050113

-0.2562487	0.0004304	-0.1668488	0.02321108	-0.0279993	0.7051898
-0.0774139	0.29492499	-0.0253378	0.73208684	-0.0776012	0.29375492
-0.0536831	0.46799595	-0.1949229	0.00784144	0.18478193	0.01180257
0.0979541	0.18468017	0.16634409	0.02363699	0.18676352	0.01091282
0.16622688	0.02373686	0.21859494	0.00279619	-0.2969652	4.05E-05
-0.194447	0.00799686	-0.1443557	0.04994647	0.17549717	0.01687522
0.09033274	0.22139313	0.01843227	0.80333925	0.22415089	0.00216043
-0.1193101	0.10575382	0.00168649	0.9818231	-0.3594098	5.05E-07
0.25819546	0.00038759	0.23267885	0.00143665	-0.1439551	0.05059201
0.20609116	0.00488652	0.10232477	0.1657546	0.28662252	7.64E-05
-0.2786141	0.00012294	-0.2610233	0.00033239	0.25251041	0.00052513
-0.2591628	0.00036782	-0.1421929	0.05351451	-0.118603	0.10785234
-0.2158963	0.00316242	-0.1337617	0.06949467	-0.174443	0.01755664
0.00517028	0.94431522	-0.0513764	0.48735784	0.25241079	0.0005279
-0.040627	0.58295899	-0.0626589	0.39682261	0.35246525	8.64E-07
0.39066726	3.85E-08	0.27720892	0.00013343	-0.3096885	1.79E-05
-0.1173678	0.11159645	-0.1489926	0.04296038	-0.3671639	2.74E-07
0.14946481	0.04229734	0.13445548	0.06804762	-0.055451	0.45344728
0.13680612	0.06332643	0.26240366	0.00030818	-0.3662349	2.95E-07
-0.3256079	6.11E-06	-0.2550987	0.00045771	0.30906886	1.86E-05
0.30004577	3.33E-05	0.24697484	0.0007012	-0.0741951	0.31552024
-0.0437825	0.55401577	-0.1046238	0.1563997	0.2684054	0.00022077
0.25314991	0.00050767	0.15847839	0.03119596	-0.0410805	0.57875605
0.22763299	0.00183215	0.10572173	0.15207468	-0.1151651	0.11852469
0.04739433	0.5217673	-0.0807901	0.27430389	0.18643332	0.01105684
0.12195349	0.09819207	0.01260494	0.8647817	-0.3588399	5.28E-07
-0.2596399	0.00035841	-0.1609333	0.02864211	-0.0412487	0.57720097
-0.253738	0.00049208	-0.2821702	9.97E-05	-0.3240079	6.82E-06
0.36819011	2.52E-07	-0.0187886	0.7996168	-0.3662727	2.94E-07
-0.2086751	0.00436506	-0.3234133	7.11E-06	0.07965916	0.28109986
0.24665029	0.00071305	-0.0835664	0.25809649	0.10679689	0.14792756
-0.1944887	0.00798313	-0.2274715	0.00184631	0.2441951	0.00080882
0.70831661	1.73E-29	0.45971436	4.62E-11	-0.1690547	0.02142609
0.05579144	0.45067506	-0.0736141	0.31933496	0.11366946	0.12341654
-0.0145439	0.84422802	0.09622867	0.19257262	0.43431096	6.55E-10
0.10341872	0.16125242	-0.0303814	0.68141993	0.23746302	0.00113538
0.20775692	0.00454433	0.07874018	0.28670476	0.18488349	0.01175546
0.05504616	0.45675634	-0.0197312	0.78979259	0.49245122	1.08E-12
-0.1116927	0.13012017	-0.0790578	0.28475925	0.51760105	4.54E-14
-0.4997577	4.42E-13	-0.365563	3.11E-07	0.1672938	0.022841
-0.2742136	0.00015867	-0.1438618	0.05074333	-0.0834134	0.25897204
-0.0420707	0.56962988	-0.0816189	0.26939511	-0.0068524	0.92624337
0.28245496	9.80E-05	0.16291715	0.0267108	-0.4407045	3.43E-10
0.00011634	0.99874603	-0.0026781	0.97113979	-0.1535287	0.03693799
-0.138557	0.05998723	-0.2151781	0.00326694	-0.1198606	0.10414234
-0.100695	0.17263506	-0.1299691	0.07785393	0.05327588	0.47138308

0.09299825	0.20800504	0.01084148	0.88355439	0.15141745	0.03964594
-0.135019	0.06689048	-0.1779065	0.01540359	-0.245992	0.00073765
-0.0438065	0.55379795	-0.1536689	0.0367638	-0.2196836	0.00265966
0.0695275	0.34700978	-0.1369549	0.06303695	0.29663351	4.13E-05
-0.4521037	1.05E-10	-0.2448657	0.00078155	-0.429956	1.01E-09
-0.0192885	0.79440271	-0.1150773	0.1188075	0.265423	0.00026083
-0.0691575	0.34958815	-0.1010129	0.17127638	-0.4194857	2.79E-09
-0.1065239	0.14897244	-0.0306649	0.67861219	0.126207	0.08693031
0.08920903	0.22721687	-0.015669	0.83234896	0.20874611	0.00435146
-0.1091727	0.13906825	-0.1568824	0.03295824	0.26798114	0.0002261
-0.0612132	0.40782748	-0.1143621	0.1211321	0.21394883	0.00345307
0.17406664	0.01780569	-0.0121016	0.87013278	0.18096117	0.01369974
0.31552931	1.22E-05	0.18900207	0.00997927	-0.2398399	0.00100833
-0.1700539	0.0206574	-0.2745138	0.00015596	0.13975595	0.05778512
-0.0940272	0.20299573	-0.015508	0.83404614	-0.3750201	1.44E-07
0.04352359	0.55636359	-0.0883261	0.23186819	0.26908205	0.00021252
-0.0968214	0.18983403	-0.1395655	0.05813047	0.24137442	0.00093338
-0.3922509	3.35E-08	-0.259715	0.00035695	0.42363126	1.87E-09
-0.2735089	0.00016523	-0.3805608	9.11E-08	0.15729689	0.03249261
-0.2020937	0.00580382	-0.1976468	0.00700258	0.09537737	0.19655604
-0.4806274	4.39E-12	-0.4017483	1.45E-08	0.34245365	1.83E-06
-0.1886347	0.01012744	-0.0551126	0.45621259	0.18403083	0.01215614
-0.0409701	0.57977769	-0.1357635	0.06538619	0.32120594	8.27E-06
-0.1100882	0.13576471	-0.2252659	0.00204991	0.16491825	0.02487681
-0.1422417	0.05343169	-0.0886541	0.23013237	0.36262875	3.92E-07
-0.1135537	0.12380147	0.02566101	0.72880251	-0.2327574	0.00143116
-0.0235977	0.74985398	0.09468062	0.19986052	-0.0738563	0.31774092
0.12957369	0.07877036	0.00573922	0.93819927	0.22054868	0.00255551
0.25281257	0.00051681	0.06095729	0.40979407	0.2263638	0.00194614
-0.0787023	0.28693764	-0.1164997	0.11428804	-0.0032641	0.96482792
-0.258028	0.00039111	-0.1715107	0.01957948	0.14407452	0.05039883
0.0215779	0.77064154	-0.0162184	0.82656183	0.01742442	0.81389112
-0.3939673	2.89E-08	-0.3177451	1.05E-05	0.12385646	0.09301851
-0.4931179	9.97E-13	-0.3521391	8.86E-07	0.47017102	1.45E-11
-0.1079505	0.14357389	-0.2159536	0.00315421	0.18828934	0.01026854
-0.1982607	0.00682493	-0.2121951	0.00373522	0.20620885	0.00486161
0.05554874	0.45265045	0.05983768	0.41846371	0.14219683	0.05350781
-0.1827284	0.01279136	-0.278345	0.00012488	0.42318771	1.96E-09
-0.0775994	0.29376585	-0.0226631	0.75945136	-0.1039621	0.15905036
-0.3304303	4.36E-06	-0.3149607	1.26E-05	0.484542	2.78E-12
0.09434392	0.20147178	0.20365798	0.00542796	0.15436275	0.03591214
-0.1897324	0.00969033	-0.0792487	0.28359385	-0.1027958	0.16380479
0.21626345	0.00311017	0.05864151	0.4278435	0.20749623	0.00459642
0.04494736	0.54351009	-0.0897646	0.22432438	0.25417953	0.00048067
0.31807292	1.02E-05	0.05718131	0.43945652	0.19955353	0.00646391
-0.3011426	3.11E-05	-0.2756496	0.00014606	0.14301405	0.05213571

0.37289078	1.72E-07	0.10070256	0.17260252	-0.1858322	0.01132332
-0.2172483	0.00297385	-0.3040878	2.58E-05	0.33977495	2.23E-06
-0.3056302	2.33E-05	0.04218003	0.56862633	0.09893249	0.18031163
-0.2340992	0.00134036	-0.308959	1.88E-05	0.3428851	1.77E-06
0.0842142	0.25441168	-0.0181065	0.80674584	0.06647681	0.36862662
0.57044181	2.33E-17	0.25122298	0.00056198	0.25711197	0.0004109
0.02620675	0.72326758	-0.0504252	0.49546513	0.25651676	0.00042426
0.08227488	0.26555244	0.09296716	0.20815776	0.3909613	3.75E-08
-0.2030439	0.00557283	-0.3286423	4.94E-06	0.32452084	6.59E-06
-0.2024061	0.00572695	-0.1315685	0.07423457	0.36878311	2.40E-07
0.32309817	7.27E-06	0.31183695	1.55E-05	0.32303423	7.30E-06
0.23791173	0.00111033	0.0610686	0.40893799	0.21264882	0.00366029
0.39355149	2.99E-08	0.19899942	0.00661651	-0.0682255	0.35613595
0.13016515	0.0774028	-0.0425421	0.56530951	0.1139163	0.12259859
-0.0321783	0.66369447	-0.1404486	0.05654359	0.12321821	0.09472896
0.11112794	0.13208594	0.12127595	0.10008816	-0.2202085	0.00259602
-0.0144084	0.84566177	-0.1934515	0.00833079	0.28743531	7.28E-05
-0.2595777	0.00035963	-0.3493914	1.09E-06	0.31719301	1.09E-05
-0.0304276	0.68096284	-0.0368642	0.6183588	-0.1098464	0.13663125
0.17858291	0.01501113	-0.0845689	0.25240935	-0.3214639	8.13E-06
0.0310547	0.67475727	-0.0487405	0.50999772	0.40201548	1.41E-08
0.07699669	0.29754274	-0.2181296	0.00285647	-0.263235	0.0002944
-0.0252583	0.73289632	-0.1673318	0.02280968	-0.0966675	0.19054221
-0.0069028	0.92570278	-0.1723931	0.01895051	0.17503256	0.01717266
-0.1365293	0.06386808	-0.0421954	0.56848564	0.02116209	0.77494191
-0.1324606	0.07227586	-0.2081928	0.0044584	0.30163302	3.01E-05
0.04783599	0.51789071	-0.0439589	0.55241817	0.26517465	0.00026445
-0.1818583	0.01323179	-0.1120118	0.12901941	-0.2253054	0.0020461
-0.2881883	6.95E-05	-0.0634311	0.391018	-0.1646466	0.02511925
-0.2990691	3.55E-05	-0.1206197	0.10195227	-0.1746212	0.01743981
-0.3057075	2.32E-05	-0.0220823	0.76543452	-0.332561	3.75E-06
0.01848717	0.80276543	-0.0586804	0.42753634	0.19431727	0.00803968
-0.2276723	0.00182872	-0.2923115	5.40E-05	0.25483576	0.00046417
-0.0627543	0.39610254	-0.2023068	0.00575127	0.31924708	9.46E-06
0.2230872	0.00227089	0.01361296	0.85408414	0.21382634	0.00347214
-0.1989821	0.00662132	-0.2408486	0.00095847	0.16048554	0.02909412
-0.2175273	0.00293622	-0.1036325	0.1603833	0.20674654	0.00474922
-0.4298678	1.02E-09	-0.3988367	1.88E-08	0.17635254	0.01633932
-0.2348692	0.00129065	-0.196601	0.0073147	0.08907746	0.22790578
-0.0569148	0.44159511	-0.148404	0.04379914	0.22752889	0.00184127
-0.1818519	0.01323508	-0.2903829	6.08E-05	0.37327452	1.67E-07
0.29730916	3.96E-05	0.1502164	0.04125968	-0.2005789	0.00618979
-0.0876496	0.23547681	-0.1400254	0.05729943	0.44879425	1.48E-10
-0.2062912	0.00484423	-0.0963018	0.19223306	-0.3052047	2.40E-05
0.02272461	0.75881836	-0.0563015	0.44653942	0.13135876	0.0747014
0.27854361	0.00012344	0.15065232	0.04066771	-0.0513608	0.48749059

-0.2511651	0.00056369	-0.2808463	0.00010783	0.11720031	0.1121118
0.26353003	0.00028965	0.04639016	0.53063599	-0.3679835	2.56E-07
0.3521117	8.87E-07	0.6025391	1.15E-19	-0.1999684	0.00635172
0.01939034	0.79334136	-0.0393125	0.59521859	0.18979431	0.00966619
0.1137819	0.12304342	0.1335619	0.069916	-0.2378929	0.00111137
-0.0752773	0.30849381	-0.1235752	0.09376924	-0.2343741	0.00132241
0.06234424	0.39920254	0.04001765	0.58862726	-0.2277115	0.00182531
0.196704	0.00728343	0.46755591	1.95E-11	0.1730655	0.0184831
0.23504099	0.0012798	0.47009637	1.47E-11	0.17183655	0.01934515
0.60518702	7.24E-20	0.43537792	5.89E-10	-0.1145569	0.12049544
0.41663559	3.66E-09	0.5817035	3.86E-18	-0.3175754	1.06E-05
-0.1884049	0.01022112	-0.0079061	0.9149414	-0.396261	2.36E-08
-0.0041567	0.9552185	-0.0999524	0.17583925	0.18591684	0.01128548
-0.2945283	4.71E-05	-0.2600693	0.00035014	0.29391512	4.89E-05
-0.0377491	0.6099496	-0.0704938	0.34033296	0.20844304	0.00440975
0.02999761	0.68522952	-0.1239999	0.09263751	0.09007035	0.2227434
-0.2519617	0.00054056	-0.1658074	0.0240973	0.3496386	1.07E-06
-0.023799	0.74779148	0.11424951	0.12150111	-0.0849615	0.25020603
-0.1469903	0.04586956	-0.0351572	0.63471889	-0.3082999	1.96E-05
-0.1826627	0.01282414	-0.1505792	0.04076645	0.23165203	0.00151017
0.04889167	0.50868494	-0.046524	0.52944993	0.40767471	8.43E-09
-0.0793889	0.28274023	-0.1800967	0.01416434	0.27066874	0.00019428
-0.2090545	0.00429285	-0.1545561	0.03567776	0.55023324	4.94E-16
-0.0340171	0.64574663	-0.0770528	0.29719009	-0.2027148	0.00565189
-0.3117861	1.56E-05	-0.184031	0.01215608	-0.507348	1.71E-13
-0.4277851	1.25E-09	-0.2453102	0.00076394	0.03035566	0.68167562
0.25378984	0.00049073	0.01538849	0.83530727	0.03866171	0.60133105
0.12253449	0.09658889	0.07040991	0.34090939	-0.2751844	0.00015004
-0.2310558	0.00155441	-0.1743084	0.01764537	0.12735988	0.08406307
0.37510419	1.43E-07	0.1192904	0.10581183	0.23726904	0.00114637
-0.269855	0.00020344	-0.0193298	0.79397174	-0.3903718	3.95E-08
-0.3089038	1.88E-05	-0.2109747	0.00394364	-0.2269313	0.0018944
0.24362009	0.0008329	0.0260236	0.72512344	0.08832419	0.23187831
-0.0789403	0.28547772	-0.1942759	0.00805338	0.09029599	0.2215819
-0.245172	0.00076938	-0.2460079	0.00073704	0.13683975	0.06326089
-0.3529334	8.33E-07	-0.2929081	5.21E-05	0.22556256	0.00202139
0.11680277	0.11334263	-0.0603723	0.41431035	0.23730817	0.00114415
-0.3760391	1.33E-07	-0.217175	0.00298381	0.20050103	0.00621024
0.11513615	0.11861789	-0.0157754	0.83122739	-0.546279	8.78E-16
0.14420851	0.05018283	0.00189072	0.97962241	0.18930757	0.00985749
-0.162197	0.02739859	-0.1881942	0.0103077	-0.2512675	0.00056067
-0.0352958	0.63338405	0.020906	0.77759394	0.22090521	0.00251367
-0.1110118	0.13249297	-0.1992205	0.00655525	0.14775877	0.04473414
-0.3677429	2.61E-07	-0.1892922	0.0098636	-0.0372695	0.61450131
-0.0128211	0.86248532	-0.0211122	0.77545865	0.27976507	0.00011492
0.08960315	0.22516212	0.11678228	0.11340634	-0.2939292	4.89E-05

0.02231748	0.76300984	0.03849418	0.60290909	-0.3223919	7.63E-06
-0.0165611	0.8229571	-0.016438	0.82425156	-0.4513225	1.14E-10
-0.1677178	0.02249319	-0.0035426	0.96182916	-0.3732487	1.67E-07
-0.1357186	0.06547629	0.08354262	0.25823261	-0.4038967	1.19E-08
-0.0950706	0.19800627	-0.0605056	0.41327891	-0.3283288	5.05E-06
0.44415978	2.41E-10	0.32349668	7.07E-06	-0.3529081	8.35E-07
-0.3231811	7.23E-06	-0.3037563	2.63E-05	-0.3524759	8.63E-07
-0.1550162	0.03512536	-0.2361898	0.00120932	-0.3947506	2.69E-08
-0.059335	0.42239061	-0.1264869	0.08622722	0.09850571	0.18220778
-0.2001624	0.00629987	-0.0589262	0.42559984	-0.0716985	0.33212446
0.22924958	0.00169578	0.19543528	0.00767716	-0.1507203	0.04057611
-0.2480613	0.00066285	-0.1558313	0.03416444	-0.2034189	0.00548396
-0.0793226	0.28314389	-0.2318378	0.00149662	0.26685644	0.0002408
-0.0123927	0.8670368	-0.0489709	0.50799744	0.27671838	0.00013729
0.09697847	0.18911321	0.08486058	0.2507711	0.0860707	0.2440528
0.02670442	0.71823262	-0.1630838	0.02655374	0.15653462	0.03335325
0.07890321	0.28570502	-0.043622	0.55547047	-0.2618179	0.00031825
-0.1484857	0.04368181	-0.1340372	0.06891714	0.2270961	0.00187961
-0.2129087	0.00361799	-0.153183	0.03737042	0.11320718	0.12495974
0.06168295	0.40423199	-0.0216247	0.77015821	-0.1729262	0.01857911
-0.2679187	0.00022689	-0.2211049	0.00249051	0.28594206	7.96E-05
-0.2773922	0.00013202	-0.2741183	0.00015954	0.02152437	0.77119477
0.06983699	0.34486238	0.03600149	0.62660416	0.02653157	0.71997992
0.01918118	0.79552125	-0.0193584	0.79367403	0.20692694	0.00471204
-0.0937288	0.20443939	-0.1955565	0.00763874	0.17718484	0.01583216
0.09446571	0.20088786	-0.003387	0.96350427	0.1798909	0.01427693
-0.3362086	2.88E-06	-0.1041748	0.15819463	0.02217877	0.76443947
-0.0547614	0.45909153	-0.1883291	0.0102522	0.16982369	0.02083239
-0.3198455	9.08E-06	-0.090809	0.21895734	0.48739147	1.98E-12
0.21108164	0.00392497	0.14561461	0.04796225	-0.3103991	1.71E-05
-0.1351377	0.0666488	-0.2147618	0.00332893	0.1522815	0.03851814
-0.0317593	0.66781112	-0.1699141	0.02076353	0.06819813	0.35632924
-0.1442251	0.05015615	-0.2231581	0.00226336	0.3103766	1.71E-05
0.0546567	0.45995233	-0.0998356	0.17634713	0.18879157	0.01006394
0.33205214	3.88E-06	0.24637989	0.00072306	-0.2415027	0.00092736
0.07095508	0.33717476	-0.0331316	0.6543649	0.4398105	3.76E-10
-0.1129871	0.12569978	-0.0648563	0.38043963	-0.2693305	0.00020956
0.10822405	0.14255582	0.01496801	0.83974603	-0.1277507	0.08310841
-0.3081201	1.98E-05	-0.2876925	7.16E-05	0.24625186	0.00072784
-0.0017778	0.98083971	-0.1050706	0.15462851	0.04389431	0.55300271
0.01067489	0.88533138	-0.0826356	0.26345558	-0.1239028	0.09289521
-0.0030685	0.96693414	-0.0797454	0.28057757	-0.1090876	0.1393784
0.37450208	1.51E-07	0.50654396	1.89E-13	-0.114152	0.12182158
-0.1136774	0.12339025	-0.1357045	0.06550441	0.09232149	0.21134772
0.18619772	0.01116063	0.05275206	0.47575962	0.26342126	0.0002914
0.04418105	0.55040999	0.19974478	0.00641198	-0.0736505	0.31909501

-0.1069833	0.14721742	-0.2422876	0.00089125	0.33537472	3.06E-06
-0.3550106	7.11E-07	-0.3800857	9.49E-08	0.13537324	0.06617124
0.20538481	0.00503848	0.13828738	0.06049173	0.20342871	0.00548165
-0.1177139	0.11053722	0.07611498	0.30312577	-0.0180635	0.8071966
-0.0785694	0.28775478	-0.1651544	0.02466769	0.14657331	0.04649566
-0.0070379	0.92425267	-0.0574259	0.43749926	0.49847476	5.18E-13
0.01215369	0.86957867	-0.0742935	0.31487716	0.22206548	0.00238179
-0.0627767	0.39593333	-0.0573205	0.43834194	0.12290681	0.0955725
-0.102526	0.16491958	-0.0236488	0.74932948	0.03255239	0.66002692
0.0954125	0.19639049	0.03351488	0.65062849	0.12482025	0.09048235
0.00077151	0.99168421	-0.0590606	0.42454356	-0.1227446	0.09601414
-0.2519019	0.00054226	-0.2908087	5.93E-05	0.52164418	2.66E-14
-0.1704674	0.02034637	-0.2166651	0.00305391	0.13214262	0.07296925
0.07801696	0.29116795	0.00423961	0.95432661	0.15776854	0.0319696
0.16263	0.02698325	0.07534172	0.30807862	-0.3228153	7.41E-06
0.44912137	1.43E-10	0.25775642	0.00039688	-0.1588476	0.03079992
0.08005378	0.27871587	-0.1015172	0.16913787	0.27801621	0.0001273
-0.1110151	0.13248148	-0.1609785	0.02859682	-0.0049393	0.94679866
-0.3054609	2.36E-05	-0.3624884	3.97E-07	0.32732252	5.42E-06
0.12975842	0.07834117	0.03407378	0.64519609	0.20793771	0.00450851
0.0932762	0.20664327	-0.0259441	0.72592959	0.10167869	0.16845725
-0.1388647	0.05941557	-0.1982828	0.00681864	0.17893257	0.0148117
-0.0207579	0.77912867	-0.1146947	0.12004672	-0.2345767	0.00130933
0.4011722	1.52E-08	0.14162534	0.05448499	-0.4200641	2.64E-09
0.04425346	0.54975618	-0.0001069	0.99884752	-0.3187394	9.79E-06
0.11207469	0.12880333	0.14084183	0.05584867	0.0607932	0.41105801
0.02080287	0.77866268	-0.026718	0.71809556	0.27944319	0.00011711
-0.0277507	0.70768659	-0.074705	0.31219673	0.27494276	0.00015215
0.14556907	0.04803286	0.03026392	0.68258553	0.0915119	0.21539689
-0.3193869	9.37E-06	-0.3534632	8.00E-07	0.34716748	1.29E-06
-0.1579822	0.03173506	-0.1842666	0.01204418	-0.3314679	4.05E-06
0.03371934	0.64863901	-0.0120775	0.87038942	0.17038777	0.02040595
-0.0879886	0.23366365	-0.1461481	0.04714138	0.23495972	0.00128492
-0.0386006	0.60190683	0.06084939	0.41062499	-0.427506	1.29E-09
-0.2030007	0.00558316	-0.0658885	0.37288915	-0.3766127	1.27E-07
0.09024832	0.22182694	-0.0828846	0.2620145	-0.1282062	0.08200686
0.08106887	0.27264627	0.02471182	0.73846261	-0.2042268	0.00529678
-0.3996221	1.75E-08	-0.2939516	4.88E-05	0.35216243	8.84E-07
0.21732162	0.00296392	0.130119	0.07750881	-0.0136799	0.85337489
0.12112787	0.1005064	-0.0851638	0.24907575	0.21640524	0.0030902
-0.5560277	2.10E-16	-0.4928101	1.03E-12	0.26290482	0.00029981
0.1392362	0.05873139	0.07140694	0.33409918	0.33158009	4.02E-06
-0.3796929	9.80E-08	-0.1599504	0.02964231	-0.3385433	2.44E-06
-0.1150232	0.11898221	-0.1067268	0.14819513	-0.231766	0.00150184
-0.0691633	0.34954737	-0.1307367	0.07609947	-0.0288066	0.69710058
0.16478587	0.0249947	0.00804479	0.9134554	-0.3849028	6.32E-08

0.03431013	0.64290446	-0.1165812	0.11403333	0.21869257	0.0027837
0.13625653	0.06440549	-0.0227062	0.75900812	0.23264716	0.00143887
-0.4162865	3.78E-09	-0.4035819	1.22E-08	0.36238953	4.00E-07
0.12008209	0.10349952	0.17042661	0.02037687	-0.2182932	0.00283514
0.0995824	0.17745217	0.03823208	0.6053817	0.25439517	0.00047519
-0.167745	0.022471	-0.2500091	0.0005989	0.06803333	0.35749538
-0.1558263	0.03417028	-0.1689088	0.02154038	-0.1679771	0.02228272
-0.0885494	0.23068567	-0.1147289	0.11993547	0.37582575	1.35E-07
-0.1412621	0.05511376	-0.2661192	0.00025091	0.2355822	0.00124614
-0.1083187	0.14220479	-0.1028177	0.16371431	0.19627008	0.00741602
0.07921764	0.28378341	-0.0425932	0.56484133	-0.1650704	0.0247419
0.00858308	0.90768947	-0.08937	0.22637589	-0.1246249	0.09099195
0.14599755	0.04737188	0.23947284	0.00102706	-0.2581267	0.00038903
-0.0660966	0.37137781	-0.0776692	0.29333033	-0.3011752	3.10E-05
-0.1454797	0.04817169	-0.1176113	0.11085029	-0.2082998	0.00443755
0.0035715	0.96151818	-0.0432757	0.55861628	0.27335261	0.00016671
-0.1974371	0.00706421	-0.2583083	0.00038523	0.22130582	0.00246741
-0.3661995	2.96E-07	-0.3899914	4.08E-08	0.18000636	0.01421366
0.44762896	1.68E-10	0.11721489	0.11206686	-0.4585378	5.25E-11
0.22169894	0.00242276	-0.0293363	0.69181123	0.18274539	0.01278288
0.0496654	0.50199225	0.00122664	0.98677882	0.4346161	6.36E-10
-0.2352343	0.00126768	-0.1431838	0.05185431	-0.1568122	0.03303769
0.02470409	0.73854148	0.03757943	0.61155813	-0.3519494	8.98E-07
-0.2263951	0.00194325	-0.2815196	0.00010363	0.12252657	0.09661062
-0.1829262	0.01269306	-0.2821929	9.96E-05	0.24940393	0.00061813
0.00410574	0.95576735	-0.0093492	0.89949178	0.3148848	1.27E-05
-0.0261631	0.72371006	-0.0211993	0.77455714	0.24128849	0.00093744
0.33692218	2.74E-06	0.15376118	0.0366496	-0.2132049	0.00357033
0.49601271	7.01E-13	0.12695224	0.08506808	-0.0928178	0.20889243
0.35315071	8.20E-07	0.27106023	0.00019001	-0.3353592	3.07E-06
-0.2790828	0.00011961	-0.2564545	0.00042568	0.00306417	0.96698115
-0.4618495	3.66E-11	-0.4523724	1.02E-10	0.37737075	1.19E-07
0.0822852	0.26549231	0.02172906	0.76907993	0.19109323	0.00917166
0.18974262	0.00968635	0.07491806	0.31081458	0.18822198	0.01029625
0.0323915	0.66160324	-0.0278551	0.70663773	0.25669485	0.00042022
-0.2297327	0.00165687	-0.1202156	0.10311346	0.12844393	0.08143675
-0.0177756	0.81021037	-0.0399828	0.5889518	0.184163	0.01209326
0.1633492	0.02630532	0.04610709	0.53314963	0.21411874	0.00342679
-0.1835683	0.01237846	-0.1273563	0.08407176	-0.2947541	4.65E-05
0.14656687	0.04650539	0.04797493	0.51667432	-0.2364392	0.0011945
-0.0587935	0.42664459	-0.0998103	0.17645745	0.00146849	0.98417237
0.04614077	0.53285032	-0.1317203	0.07389833	0.20768988	0.00455767
-0.1295951	0.0787206	0.07803125	0.29107932	-0.3363478	2.86E-06
-0.2711236	0.00018933	-0.3083701	1.95E-05	0.0685073	0.35414801
0.07339361	0.32079054	-0.0502665	0.49682512	0.13691343	0.06311747
0.00495714	0.94660728	0.03006372	0.68457282	0.33112222	4.15E-06

0.05584462	0.45024289	-0.0964655	0.19147514	-0.2440622	0.00081433
-0.0581082	0.43206394	-0.0796356	0.2812425	0.33889099	2.38E-06
-0.2225447	0.00232917	-0.2179129	0.00288493	0.16771241	0.02249755
0.17151778	0.01957436	-0.0077604	0.91650385	-0.2762414	0.00014114
-0.1533855	0.03711656	-0.22132	0.00246579	-0.4223565	2.12E-09
-0.0633847	0.39136489	0.00586698	0.93682635	-0.0035294	0.96197123
0.29843663	3.69E-05	0.2392049	0.00104093	-0.3634711	3.67E-07
-0.0128499	0.86217934	0.05039311	0.49574006	-0.2291137	0.00170688
0.51089568	1.09E-13	0.14329701	0.05166747	-0.3424892	1.82E-06
0.24437508	0.00080142	0.13778914	0.06143335	-0.0940079	0.20308915
-0.3845842	6.49E-08	-0.1975181	0.00704034	0.07733213	0.29543675
-0.2667459	0.00024229	-0.2055655	0.0049992	0.51804869	4.29E-14
-0.2204892	0.00256256	-0.2810067	0.00010682	0.38898978	4.45E-08
-0.0348573	0.63761215	-0.0702815	0.34179317	-0.0904633	0.22072324
-0.0772122	0.29618878	-0.1571762	0.03262757	0.01732548	0.81492877
-0.1481466	0.04417008	-0.1738454	0.01795353	0.25171134	0.00054773
0.17461014	0.01744706	-0.0199017	0.78801822	0.18074508	0.0138146
-0.1173393	0.1116841	-0.091699	0.21445635	-0.0858774	0.24511754
0.04515189	0.54167573	-0.1135992	0.12365014	0.43556655	5.78E-10
0.08973005	0.22450329	0.08090076	0.27364521	-0.1817064	0.01331001
-0.1359334	0.06504701	-0.2145134	0.00336642	-0.5667774	4.11E-17
0.06535588	0.3767737	-0.0961405	0.19298227	0.17356783	0.01814046
-0.1903031	0.00946976	-0.1637099	0.02597083	-0.291856	5.56E-05
-0.1206916	0.10174657	-0.1355082	0.06589894	0.27285625	0.00017152
0.03433693	0.6426448	-0.0598585	0.4183013	0.12842015	0.08149365
-0.0875379	0.23607679	-0.1722694	0.01903765	0.25242186	0.00052759
0.0117196	0.87419773	0.1000097	0.17559049	-0.340559	2.10E-06
-0.0984266	0.18256091	-0.0636661	0.38926201	-0.2923554	5.39E-05
-0.166841	0.02321757	-0.0418108	0.57201895	-0.1887955	0.01006236
-0.1154341	0.11766106	-0.128597	0.08107135	0.13850563	0.06008301
-0.1317078	0.07392609	-0.1643914	0.02534885	-0.278766	0.00012185
-0.1460244	0.04733071	-0.1829786	0.01266709	0.14675243	0.04622586
0.03924953	0.59580868	-0.0890543	0.22802696	0.21793896	0.0028815
-0.0353699	0.63267019	-0.1197783	0.1043822	0.06841967	0.35476536
-0.2635008	0.00029012	-0.2122748	0.00372195	0.24731238	0.00068907
0.02196999	0.76659276	0.14524577	0.04853666	0.02752212	0.70998627
-0.1376305	0.06173562	-0.1119796	0.12913017	0.36062769	4.59E-07
-0.0788866	0.2858068	-0.136943	0.06306001	0.18842818	0.01021162
0.21586441	0.003167	0.16493794	0.02485932	-0.2149817	0.00329605
-0.0927194	0.20937757	-0.2143479	0.00339163	-0.1220328	0.09797205
0.01329544	0.85745108	-0.0278936	0.70625105	-0.3167352	1.12E-05
0.06549047	0.37578973	-0.0499718	0.49935456	0.19746277	0.00705663
-0.0131021	0.8595024	-0.0404511	0.58459221	0.22174253	0.00241785
-0.0928298	0.20883325	-0.0043702	0.95292088	-0.0874515	0.23654125
-0.0521444	0.48086439	-0.1077721	0.14424078	0.1446429	0.04948788
-0.2521676	0.00053472	-0.1414668	0.05475864	-0.3023442	2.88E-05

0.00289219	0.96883336	-0.1066205	0.14860182	0.24513232	0.00077095
-0.087008	0.23893577	-0.085698	0.24610865	-0.2811918	0.00010566
0.26101607	0.00033252	0.01065037	0.88559287	-0.1758382	0.01665976
0.00479238	0.94837943	-0.0890623	0.22798522	0.14479119	0.04925248
0.08708233	0.2385335	0.02597778	0.72558803	0.18551238	0.01146743
0.07239556	0.32743291	-0.0288348	0.69681832	0.11317837	0.12505643
-0.3606422	4.59E-07	-0.3065872	2.19E-05	0.43997051	3.70E-10
0.22918366	0.00170116	0.41298636	5.16E-09	0.24664839	0.00071312
0.04660361	0.52874455	-0.0318846	0.66657909	0.25572413	0.00044266
-0.0614019	0.40638113	-0.0205909	0.78086024	0.35440481	7.45E-07
-0.1908354	0.009268	-0.2535734	0.0004964	0.19733703	0.00709378
-0.3212305	8.26E-06	-0.3223525	7.65E-06	0.17794325	0.01538205
-0.0040619	0.95623892	-0.136433	0.06405733	0.19589901	0.0075311
0.03900664	0.59808779	0.07313654	0.322493	0.15551763	0.03453153
0.130563	0.07649373	0.09078144	0.21909759	-0.2158665	0.0031667
-0.1532753	0.03725457	-0.1507666	0.04051376	0.47371247	9.74E-12
-0.2653861	0.00026136	-0.2288321	0.00173009	-0.0425368	0.56535803
-0.0471225	0.52416069	-0.0665355	0.36820313	0.20068587	0.00616181
-0.2462861	0.00072656	-0.0632072	0.39269592	-0.3474053	1.27E-06
-0.0378898	0.60861724	-0.05875	0.42698766	0.12473448	0.09070577
-0.349467	1.08E-06	-0.210746	0.00398384	0.09398516	0.20319885
5.94E-05	0.99935949	-0.0367529	0.61941956	0.20419916	0.00530309
-0.0840922	0.25510314	-0.0361377	0.62529917	-0.4231124	1.97E-09
0.02838157	0.70135511	-0.0225958	0.76014414	-0.0143664	0.84610533
-0.1026514	0.16440048	-0.1308314	0.07588529	-0.3864633	5.53E-08
-0.1573979	0.03238002	-0.0402164	0.58677584	-0.1074829	0.14532647
-0.2243636	0.00213894	-0.1505719	0.04077637	-0.2134237	0.00353546
-0.182307	0.01300303	-0.1012561	0.17024262	-0.0522324	0.48012315
-0.0502249	0.49718146	0.04025832	0.58638529	-0.156779	0.03307529
0.2206645	0.00254185	0.09640896	0.19173663	-0.280774	0.00010829
0.08931301	0.22667352	-0.1498959	0.04169947	-0.3323384	3.81E-06
-0.2045706	0.00521888	-0.2066956	0.00475978	-0.1465834	0.04648045
-0.0855131	0.24713261	-0.1619016	0.027685	0.23807908	0.00110111
-0.1602676	0.02931635	-0.2155189	0.00321695	0.14352051	0.05130012
-0.0075565	0.91868922	-0.0340937	0.64500252	-0.1116838	0.13015082
-0.0072293	0.92219892	-0.0918505	0.21369672	0.29327899	5.09E-05
0.40635109	9.52E-09	0.18088288	0.01374126	0.06011148	0.4163337
-0.3049771	2.43E-05	-0.1789032	0.01482835	-0.1832297	0.01254349
-0.1849206	0.01173831	-0.134613	0.06772254	0.32640126	5.78E-06
0.14347792	0.05136994	0.09214665	0.21221753	0.12378705	0.09320333
0.21592477	0.00315834	0.12721178	0.08442709	-0.3131695	1.42E-05
0.65832005	2.37E-24	0.26455362	0.00027372	-0.3168397	1.11E-05
0.05596892	0.44923366	-0.0225908	0.76019566	0.22690373	0.00189688
-0.0188607	0.79886435	-0.1601708	0.02941545	0.21182574	0.00379722
0.34040005	2.13E-06	0.17698007	0.01595564	-0.1300487	0.07767063
-0.2473327	0.00068835	-0.2041139	0.00532259	0.1286937	0.08084108

-0.1480322	0.04433585	-0.1802408	0.01408592	0.05117926	0.48903241
-0.1168644	0.11315119	-0.1139593	0.12245661	0.24308851	0.00085574
0.48233628	3.60E-12	0.26482988	0.00026956	-0.1434576	0.05140332
0.1941391	0.00809884	-0.0738313	0.31790559	0.12526577	0.08932876
-0.3396504	2.25E-06	-0.274301	0.00015788	0.13359026	0.06985606
-0.3753181	1.41E-07	-0.3412046	2.01E-06	0.30035753	3.27E-05
0.23732039	0.00114345	0.05151223	0.48620611	0.27320833	0.0001681
-0.2966168	4.14E-05	-0.1330348	0.07103766	-0.2122564	0.00372502
-0.2230485	0.002275	-0.2454402	0.00075886	-0.3352443	3.09E-06
0.0704344	0.3407411	-0.1041638	0.15823869	-0.2902416	6.13E-05
0.19194727	0.00885883	0.05149129	0.48638359	0.31649057	1.14E-05
0.06338268	0.39138042	-0.0558523	0.45018072	0.12841096	0.08151564
-0.3759093	1.34E-07	-0.2935475	5.01E-05	0.18818779	0.01031035
-0.0046335	0.95008809	-0.0665401	0.36816966	0.29691453	4.06E-05
-0.171688	0.01945167	-0.1576988	0.03204652	0.14770577	0.0448117
0.08219978	0.26599048	-0.0183012	0.8047094	0.33026793	4.41E-06
0.19873687	0.00668993	0.03706377	0.61645764	0.34273506	1.79E-06
-0.1001999	0.17476648	-0.2036683	0.00542555	0.23818224	0.00109547
0.00239123	0.97422973	-0.1148658	0.11949127	0.05749445	0.43695111
0.12780343	0.08298039	0.10460413	0.15647792	-0.3012304	3.09E-05
-0.2005977	0.00618486	-0.2925088	5.34E-05	0.36373444	3.60E-07
-0.3460877	1.40E-06	-0.324315	6.68E-06	0.02844327	0.70073689
0.1129657	0.12577179	0.10754759	0.14508317	0.24515786	0.00076994
-0.1944723	0.00798851	-0.0987841	0.18096938	0.40056916	1.61E-08
0.08219617	0.26601155	-0.0320224	0.66522476	0.29428461	4.78E-05
-0.1978195	0.00695221	-0.1370983	0.06275876	0.01223341	0.86873091
-0.0441456	0.55073037	-0.1239939	0.09265354	-0.3034843	2.68E-05
0.0675246	0.36111015	-0.0496092	0.50247721	0.06490605	0.38007388
-0.0363758	0.6230205	-0.2035083	0.00546296	0.24064022	0.00096858
0.02223171	0.76389379	-0.0777442	0.29286338	0.20161234	0.00592407
-0.2337174	0.00136564	-0.3059257	2.29E-05	0.26474632	0.00027081
0.00052828	0.99430577	-0.0747853	0.31167547	0.09509926	0.19787027
0.01428634	0.84695267	0.04281188	0.56284346	-0.2400839	0.00099606
-0.0904137	0.22097785	-0.1384977	0.0600979	0.24879399	0.00063809
0.04822384	0.51449872	0.02770603	0.70813618	-0.5826125	3.33E-18
0.23115632	0.00154687	0.04726496	0.52290569	-0.0772477	0.29596609
0.2256548	0.00201259	0.13067226	0.0762456	0.07770152	0.29312909
-0.0897622	0.22433659	-0.0891759	0.22739011	-0.3877545	4.95E-08
-0.2308658	0.00156877	-0.2763039	0.00014063	0.08539171	0.24780683
-0.0475312	0.52056484	-0.0041173	0.95564328	-0.0296998	0.68819089
0.11744299	0.1113656	0.17527164	0.01701904	0.21466606	0.00334334
-0.1368851	0.06317259	0.06059532	0.41258535	-0.3390579	2.35E-06
0.08104163	0.27280798	0.01462703	0.84334919	-0.5654852	5.02E-17
0.18724922	0.01070397	0.00553848	0.94035673	0.00757226	0.91852074
-0.0583369	0.43025137	-0.2377917	0.00111698	-0.1206514	0.10186153
-0.0950456	0.1981247	-0.1091988	0.13897305	0.25922624	0.00036655

0.18076576	0.01380357	0.07375288	0.31842102	0.18999961	0.00958649
-0.0746298	0.3126855	-0.0200725	0.7862433	-0.0455805	0.53784204
-0.1585485	0.0311204	0.01040084	0.88825572	-0.3558234	6.68E-07
-0.0639454	0.38718046	-0.1063923	0.14947781	-0.0034498	0.96282862
0.03478611	0.63829952	-0.0989724	0.18013496	-0.0305624	0.67962698
-0.045317	0.54019688	0.27452937	0.00015582	-0.3045092	2.51E-05
-0.0116917	0.8744946	0.14472062	0.04936439	-0.3099126	1.76E-05
-0.2890116	6.61E-05	-0.1828538	0.01272894	-0.2655881	0.00025844
-0.1468248	0.04611723	-0.0763553	0.30159703	0.01585151	0.83042539
-0.0406527	0.58271956	0.14818223	0.0441186	-0.3570753	6.06E-07
-0.2425622	0.00087893	-0.0869337	0.23933862	-0.1884206	0.0102147
0.07794042	0.291643	-0.0406914	0.58236094	-0.022977	0.75622363
-0.1840589	0.01214279	-0.142231	0.05344977	0.21372877	0.00348739
-0.1547477	0.03544681	-0.1209237	0.10108538	-0.2954639	4.45E-05
-0.1237299	0.09335572	-0.2603147	0.00034549	0.04242672	0.56636525
-0.1423284	0.05328499	0.02840535	0.70111683	-0.2855738	8.14E-05
-0.1867281	0.01092818	-0.2244263	0.00213264	-0.0169282	0.81909898
-0.2038769	0.00537713	-0.2279473	0.00180488	0.40890462	7.53E-09
-0.0704219	0.34082691	-0.115032	0.11895392	0.10366554	0.16024939
0.03534399	0.6329198	-0.0676338	0.36033208	-0.0212471	0.77406181
0.02142925	0.77217807	-0.0146466	0.84314183	-0.1729504	0.01856242
-0.3981113	2.00E-08	-0.3504249	1.01E-06	0.38790687	4.88E-08
-0.1033759	0.16142693	-0.1436352	0.05111247	0.36182258	4.18E-07
-0.2383672	0.00108541	-0.0848702	0.25071738	0.07957961	0.28158212
-0.1650259	0.02478133	-0.3028869	2.78E-05	0.19020522	0.00950726
0.04311367	0.56009142	0.02947368	0.69044242	0.18419268	0.01207918
-0.2261175	0.00196899	-0.1691736	0.02133334	0.31753664	1.06E-05
0.0563793	0.44591055	-0.0735712	0.3196178	0.36575903	3.06E-07
-0.1635207	0.02614584	-0.1860479	0.01122709	-0.2327817	0.00142947
0.125318	0.08919432	0.04202907	0.57001211	0.18660464	0.01098191
-0.3386445	2.42E-06	-0.2071552	0.00466538	-0.0392078	0.59620023
-0.3249673	6.39E-06	-0.2645968	0.00027306	0.26987556	0.00020321
-0.1958623	0.00754256	-0.2222369	0.00236285	-0.3119475	1.54E-05
-0.2731099	0.00016905	-0.4051379	1.06E-08	0.27738312	0.00013209
0.01724274	0.81579686	0.11183968	0.12961224	0.18824079	0.01028851
-0.1238656	0.0929941	-0.0091322	0.90181221	-0.1412943	0.05505773
0.08855475	0.23065724	-0.0295235	0.68994633	0.1828953	0.01270836
0.0558591	0.45012525	0.02005893	0.78638414	-0.231312	0.00153526
-0.2550507	0.00045888	-0.242499	0.00088175	0.07662404	0.29989405
0.07037206	0.34116968	-0.0256759	0.72865161	0.18655051	0.01100554
-0.0492633	0.5054648	-0.1473129	0.04539002	0.31859987	9.88E-06
-0.0026294	0.97166432	-0.0898785	0.2237342	0.19256118	0.00863984
0.14953155	0.04220431	0.00543834	0.94143327	0.18198654	0.01316603
-0.2915772	5.65E-05	-0.3545027	7.39E-07	0.13710493	0.06274599
-0.0308401	0.67687807	-0.1353758	0.06616608	0.12401769	0.09259035
0.18922691	0.00988952	0.2394608	0.00102768	-0.3001126	3.32E-05

0.11032036	0.13493647	0.10644927	0.14925892	-0.26296	0.0002989
0.06424608	0.38494754	0.01192605	0.87200042	-0.3174285	1.07E-05
-0.0279688	0.70549532	-0.037839	0.60909796	0.31988675	9.05E-06
-0.1433983	0.05150069	-0.1973183	0.00709932	0.05635334	0.44612039
0.1817623	0.01328117	0.34640967	1.36E-06	-0.4856721	2.43E-12
0.01358014	0.85443211	-0.1166889	0.11369726	0.03406274	0.64530319
-0.0125541	0.86532184	-0.0502724	0.49677389	-0.2713508	0.0001869
-0.0410996	0.57857902	-0.0655648	0.37524676	-0.1570967	0.03271685
0.02408612	0.74485306	0.08773556	0.23501631	-0.3325158	3.76E-06
-0.188083	0.01035367	-0.2869441	7.49E-05	0.22571704	0.00200668
-0.090361	0.22124793	-0.0318026	0.66738547	0.1230106	0.09529069
-0.2976324	3.88E-05	-0.0583004	0.4305406	0.37264922	1.75E-07
-0.4263131	1.44E-09	-0.2301021	0.00162766	0.09510584	0.19783906
-0.1537093	0.03671382	-0.2590453	0.00037017	0.08081907	0.2741315
-0.0960013	0.19363098	0.00448614	0.95167398	-0.2230073	0.00227939
0.03433979	0.64261708	0.09206409	0.21262916	-0.2138788	0.00346396
-0.1634197	0.02623962	-0.2022577	0.00576334	0.15250879	0.03822601
-0.0574922	0.43696886	-0.1349187	0.06709529	0.18908276	0.00994697
-0.20147	0.00596005	-0.2410332	0.00094959	0.03786183	0.60888222
0.20312561	0.00555335	0.00435639	0.95307001	0.11477375	0.11978986
-0.0856512	0.24636739	-0.1584138	0.03126566	0.34445428	1.58E-06
0.13237042	0.07247203	0.12831546	0.08174449	0.24361857	0.00083296
-0.1804696	0.01396228	-0.0001917	0.99793422	-0.2758557	0.00014433
-0.2872323	7.37E-05	-0.2660625	0.00025171	0.22018702	0.00259859
0.28991087	6.26E-05	0.1909455	0.00922675	-0.2454542	0.00075832
0.06575761	0.37384132	0.04327918	0.55858479	-0.1102995	0.13501063
0.05099386	0.49060989	0.02011811	0.78576906	0.47033354	1.43E-11
-0.0434402	0.55712107	-0.136918	0.06310855	-0.081353	0.27096353
-0.2145178	0.00336576	-0.1334791	0.07009119	0.04004306	0.58839036
-0.1192837	0.10583166	-0.1572721	0.03252035	0.18644085	0.01105354
0.11811659	0.10931475	0.25363609	0.00049475	-0.1498456	0.04176886
-0.1841308	0.01210857	-0.2411843	0.00094238	0.07625886	0.30221006
0.2362205	0.00120749	0.1178982	0.10997639	0.15725176	0.03254304
-0.1657584	0.02413966	-0.1745857	0.01746307	0.10551981	0.15286321
0.07983826	0.28001616	0.1628193	0.02680337	-0.3170741	1.10E-05
-0.0404799	0.58432494	-0.0458147	0.53575191	-0.2225456	0.00232907
-0.3674306	2.68E-07	-0.3433583	1.71E-06	0.06123568	0.40765496
0.13378648	0.06944254	0.05651107	0.44484653	-0.4877854	1.89E-12
0.52410574	1.92E-14	0.35413092	7.60E-07	-0.2170781	0.00299701
-0.0544948	0.46128426	-0.101721	0.16827919	0.19123695	0.00911834
0.23012535	0.00162584	0.16840695	0.02193753	-0.3699587	2.18E-07
0.07479136	0.31163589	0.06908814	0.3500727	-0.2777595	0.00012923
0.34908514	1.12E-06	0.19939172	0.00650815	0.25737106	0.00040521
0.13848099	0.06012904	0.27501972	0.00015147	-0.1520468	0.0388218
0.2447763	0.00078514	0.12267021	0.09621739	-0.252067	0.00053756
-0.0885647	0.23060462	-0.2471358	0.00069539	0.2431915	0.00085127

0.10814165	0.14286186	0.02849298	0.70023901	-0.4014244	1.49E-08
-0.424859	1.66E-09	-0.2433673	0.00084369	-0.0120365	0.87082497
0.48323546	3.24E-12	0.35566617	6.76E-07	-0.2384626	0.00108026
-0.0059061	0.93640635	0.07564636	0.30612106	-0.361045	4.45E-07
-0.1313832	0.0746468	-0.129059	0.07997629	0.32115888	8.30E-06
0.0933599	0.20623445	0.04715354	0.52388701	-0.2489127	0.00063416
0.01550847	0.83404163	-0.1396763	0.05792924	-0.1801728	0.01412286
0.25960291	0.00035913	0.06945346	0.34752472	0.0565296	0.44469704
0.04197147	0.57054129	-0.0519146	0.48280243	0.22254229	0.00232943
-0.0775659	0.2939747	-0.1257047	0.08820382	0.45439545	8.20E-11
-0.1321514	0.07294994	-0.0929071	0.20845302	0.19200308	0.00883872
0.24830827	0.00065441	0.17981722	0.01431744	0.27329329	0.00016728
-0.2161393	0.00312775	-0.1693767	0.02117573	0.28957016	6.39E-05
0.11004703	0.13591184	0.29139507	5.72E-05	0.15887662	0.03076897
-0.1774612	0.01566684	-0.1368024	0.06333362	0.28918571	6.54E-05
-0.1262418	0.08684273	-0.171163	0.01983224	0.28074092	0.0001085
0.10124702	0.17028114	0.24770984	0.00067504	0.20368888	0.00542076
-0.2359753	0.0012222	-0.1147127	0.11998818	-0.4457779	2.03E-10
-0.0234431	0.75143875	-0.0316676	0.66871428	-0.0096535	0.89623825
-0.0698141	0.34502065	0.10730437	0.14600012	-0.278072	0.00012689
0.2276354	0.00183194	0.10803694	0.14325152	-0.2574417	0.00040367
0.12639847	0.08644876	0.03054906	0.67975888	0.18708729	0.0107732
-0.4668994	2.10E-11	-0.4260147	1.49E-09	0.32754909	5.34E-06
0.17874739	0.01491703	0.07170155	0.33210362	0.22003911	0.0026164
-0.0210207	0.77640548	-0.1334496	0.07015368	0.36877243	2.40E-07
-0.1179552	0.10980351	-0.1471608	0.04561562	0.18953385	0.00976815
-0.3042656	2.55E-05	0.01541726	0.8350037	-0.4452728	2.14E-10
-0.0203186	0.78368686	-0.1038507	0.15949988	0.27923772	0.00011853
0.10780758	0.14410783	-0.0144562	0.84515516	0.18450268	0.01193295
0.06646209	0.36873291	0.03349057	0.65086519	-0.1788069	0.01488311
0.25105992	0.00056682	0.17198891	0.01923642	0.26748564	0.00023247
-0.1022279	0.16615782	-0.1364389	0.06404578	-0.3690187	2.36E-07
0.14216535	0.05356126	0.22938117	0.0016851	-0.3032984	2.71E-05
-0.0994062	0.17822394	-0.1240355	0.09254327	0.09274302	0.20926119
-0.1537877	0.03661687	-0.0947379	0.19958738	-0.2051068	0.00509944
0.06491051	0.38004111	0.04075767	0.58174622	-0.2635007	0.00029012
-0.110769	0.13334702	-0.1883727	0.01023432	0.17829124	0.01517928
-0.3027205	2.81E-05	-0.1275082	0.08369966	-0.2517223	0.00054741
0.1867198	0.01093179	0.06666064	0.3673009	0.2277611	0.00182099
-0.0268154	0.71711116	0.01575788	0.83141213	-0.2615543	0.00032288
-0.0693345	0.34835337	0.02111813	0.77539697	-0.0919158	0.21336972
0.01965688	0.79056575	0.07066761	0.33914079	0.04215925	0.568817
-0.0055411	0.94032832	-0.0070461	0.92416528	-0.2941469	4.82E-05
-0.2968505	4.08E-05	-0.14279	0.05250896	-0.0907669	0.21917156
0.14274701	0.05258081	0.0084736	0.90886184	-0.0107252	0.8847948
-0.1563619	0.03355087	-0.1850472	0.01167986	0.14793469	0.04447757

-0.0719852	0.33018975	-0.0665449	0.36813516	-0.2023819	0.00573288
0.02187814	0.76754063	0.05144133	0.48680715	0.04367969	0.55494728
0.16578332	0.02411811	0.13165477	0.07404341	0.27009796	0.00020067
-0.1314394	0.07452175	-0.0789652	0.28532565	-0.2433398	0.00084487
-0.2575637	0.00040103	-0.0525978	0.47705288	-0.0504106	0.49559007
-0.2908201	5.92E-05	-0.1159715	0.11595027	-0.233479	0.00138165
0.0083329	0.91036871	-0.0782339	0.28982453	0.09952451	0.17770552
-0.0153976	0.83521061	-0.0806474	0.27515537	-0.0400036	0.58875793
-0.3053917	2.37E-05	-0.2035107	0.00546238	0.40262485	1.34E-08
0.04547422	0.53879113	-0.0750384	0.31003589	-0.0627593	0.39606459
-0.0326395	0.65917408	-0.1330303	0.07104728	0.25400589	0.00048513
-0.001679	0.98190348	0.00770616	0.91708501	0.18061414	0.01388461
-0.2041496	0.00531441	-0.0527084	0.47612502	-0.0061624	0.93365237
0.20743146	0.00460944	-0.1115878	0.1304837	-0.1893614	0.00983616
0.00379455	0.95911686	-0.1834363	0.01244257	0.10475417	0.15588129
-0.0369717	0.61733415	-0.0347594	0.63855739	-0.0821001	0.26657239
-0.0491548	0.5064041	0.07087505	0.33772136	0.05852612	0.42875468
0.19539061	0.00769136	0.15776602	0.03197238	-0.1958258	0.00755399
0.07121611	0.33539587	-0.1402042	0.0569791	-0.3201391	8.90E-06
-0.3326419	3.72E-06	-0.1259051	0.08769405	-0.0426903	0.56395449
0.03840409	0.60375853	0.05062449	0.4937608	-0.4283428	1.18E-09
-0.4229136	2.01E-09	-0.2898022	6.30E-05	-0.0537946	0.46707056
0.2333143	0.00139281	0.12147524	0.09952746	-0.0740012	0.31678986
-0.3093382	1.83E-05	-0.2058634	0.00493507	0.19540809	0.0076858
0.15710315	0.03270955	0.00100432	0.98917493	-0.1798583	0.01429482
-0.1323811	0.07244869	-0.0352896	0.6334434	-0.3709914	2.01E-07
0.24855658	0.00064602	-0.0980509	0.18424467	-0.077954	0.29155844
-0.1143887	0.12104492	-0.1632641	0.02638475	0.09706523	0.18871589
0.22536374	0.00204046	0.12036997	0.10266866	-0.2797311	0.00011515
-0.4564259	6.60E-11	-0.2487997	0.0006379	0.11422863	0.12156965
0.13585381	0.06520571	0.01528951	0.83635165	0.20604355	0.00489663
-0.0284333	0.70083725	-0.0390973	0.59723647	0.17114027	0.01984885
-0.241801	0.00091348	-0.2766195	0.00013808	0.12367085	0.09351337
-0.0611404	0.40838653	-0.0878864	0.2342094	0.02958291	0.68935438
-0.3577445	5.75E-07	-0.1476114	0.04494998	0.03389142	0.64696654
-0.0461559	0.53271607	-0.0884269	0.23133368	0.2726378	0.00017367
0.27885168	0.00012124	0.2206446	0.00254419	-0.1574841	0.03228421
-0.1232766	0.09457149	0.07865777	0.28721102	-0.0158345	0.83060438
-0.0990896	0.17961721	-0.2229774	0.00228257	0.08758432	0.2358274
0.25033973	0.00058863	-0.0037462	0.95963752	-0.338071	2.52E-06
-0.0773069	0.29559459	-0.0185733	0.80186507	0.03359398	0.64985855
-0.0639398	0.38722228	-0.0925486	0.21022174	-0.2780752	0.00012687
-0.0947013	0.1997617	-0.2426045	0.00087704	-0.5769694	8.28E-18
-0.1058196	0.15169371	-0.167192	0.02292517	0.17537019	0.01695606
0.14776825	0.04472028	0.18529369	0.01156688	-0.258926	0.00037257
0.05790707	0.4336622	-0.0559115	0.44969955	0.20273608	0.00564674

0.39890521	1.86E-08	0.17317259	0.01840959	0.00460603	0.95038408
-0.2219836	0.00239088	-0.2715083	0.00018523	0.13229327	0.07264012
0.00340249	0.96333785	0.05301611	0.47355071	-0.2328587	0.00142412
0.09276509	0.20915234	0.02272956	0.75876748	0.17338134	0.01826702
-0.1769738	0.01595942	-0.1492935	0.0425369	-0.1174318	0.11139993
0.11706502	0.11252951	-0.0862894	0.24285204	0.06272972	0.39628806
0.10491251	0.15525347	0.02402314	0.7454973	0.32981694	4.55E-06
0.5433833	1.33E-15	0.16918964	0.02132089	-0.255824	0.0004403
-0.2439244	0.00082008	-0.3195855	9.24E-06	0.13951297	0.05822591
-0.05229	0.47963854	0.03018765	0.68334233	-0.1207705	0.10152132
-0.2498883	0.0006027	-0.3020132	2.94E-05	-0.0471621	0.52381176
-0.1366326	0.06366546	0.01440047	0.84574516	-0.0313251	0.67208838
0.12225207	0.09736558	-0.0034178	0.96317318	0.25088213	0.00057213
-0.1510365	0.04015189	-0.1742077	0.01771203	-0.4884145	1.76E-12
-0.0080228	0.91369096	-0.1755755	0.01682549	0.06928748	0.34868094
0.05848372	0.42908976	-0.0287705	0.69746204	0.14355309	0.05124674
-0.2699657	0.00020217	-0.2458148	0.0007444	0.14072604	0.05605257
0.35381246	7.79E-07	0.1962694	0.00741623	-0.0849286	0.25038994
-0.3877401	4.95E-08	-0.3465557	1.35E-06	0.14153703	0.0546373
0.14078092	0.05595586	0.05405762	0.46489261	0.19100888	0.00920308
-0.2417077	0.0009178	-0.2383559	0.00108603	-0.0517254	0.48440091
-0.0565367	0.44463945	-0.0136512	0.85367871	0.16632498	0.02365325
0.00134442	0.98550947	0.01137977	0.87781674	0.38379406	6.94E-08
-0.0751907	0.30905195	-0.1344311	0.06809801	0.0465543	0.52918118
-0.2473488	0.00068778	-0.3389707	2.36E-06	0.35900729	5.21E-07
-0.0367888	0.61907763	-0.0201645	0.78528727	-0.3259165	5.98E-06
-0.2515881	0.0005513	-0.2287163	0.00173971	0.22140234	0.00245638
0.10723819	0.14625042	0.01612351	0.82756062	0.29062413	5.99E-05
0.26379475	0.00028545	0.0343909	0.64212208	0.04472739	0.54548632
-0.2185044	0.00280784	-0.0682351	0.35606771	-0.1926945	0.00859291
0.22516567	0.00205963	0.01077062	0.88431014	0.31473403	1.28E-05
0.04203851	0.56992547	-0.0318512	0.66690715	-0.0655364	0.37545395
-0.0210225	0.77638765	0.04753751	0.520509	0.23912117	0.0010453
0.24625729	0.00072764	0.13736551	0.06224339	0.23012279	0.00162604
-0.1753929	0.01694155	-0.1244241	0.09151797	-0.3169706	1.10E-05
-0.3292936	4.72E-06	-0.1081336	0.14289188	0.31692838	1.11E-05
0.37081072	2.04E-07	0.04434063	0.54896959	0.16309302	0.02654511
0.22771548	0.00182496	-0.0227368	0.7586927	0.26882265	0.00021565
-0.046984	0.52538202	0.14919067	0.04268121	-0.1954024	0.00768761
-0.1107741	0.1333292	-0.1598132	0.02978426	-0.3394685	2.28E-06
-0.1129908	0.12568735	-0.0489377	0.50828573	-0.4313391	8.81E-10
-0.1378457	0.06132579	0.07766812	0.29333723	-0.1123451	0.12787742
-0.0851546	0.24912737	-0.048333	0.51354636	0.13675238	0.06343129
-0.1388823	0.05938293	-0.1504437	0.04095009	0.24863507	0.00064339
0.10049585	0.17349001	-0.0083467	0.91022123	0.28771575	7.15E-05
0.0137525	0.85260543	-0.0174559	0.81356093	0.35700803	6.09E-07

-0.0536765	0.46805066	-0.0206137	0.7806243	0.28870893	6.74E-05
0.14100239	0.05556695	0.10647258	0.14916937	0.15830043	0.0313884
0.12618659	0.08698175	-0.0112748	0.87893466	0.21467639	0.00334178
0.04851838	0.51193037	-0.1009365	0.17160216	-0.0822975	0.26542065
0.04286573	0.56235197	-0.0831164	0.26067807	0.13504727	0.06683279
-0.0871762	0.238026	-0.1225355	0.09658606	0.18388159	0.0122275
0.03294712	0.656166	0.063365	0.3915128	0.33266757	3.72E-06
-0.0510787	0.48988802	-0.104109	0.15845902	0.25788904	0.00039405
-0.1021287	0.16657141	-0.1505073	0.04086391	0.36451023	3.38E-07
-0.0013429	0.9855257	-0.0693985	0.34790736	0.16796649	0.02229127
-0.1846583	0.01186012	-0.1488057	0.04322521	-0.0274604	0.71060744
0.15016331	0.04133227	0.21224409	0.00372706	-0.184839	0.01177609
-0.3097323	1.78E-05	-0.2064012	0.00482114	0.04571952	0.53660096
-0.2115852	0.0038381	-0.2516917	0.0005483	0.24427704	0.00080544
-0.192321	0.00872496	-0.2089385	0.00431482	0.08989186	0.22366528
0.205253	0.0050673	-0.071713	0.3320261	0.0355401	0.63103332
0.09843952	0.18250316	0.05237313	0.47893938	-0.0173557	0.81461208
-0.216859	0.00302707	-0.2870175	7.46E-05	-0.5401494	2.11E-15
0.21549444	0.00322052	-0.0360118	0.62650528	0.07283196	0.32451771
0.03742385	0.6130346	0.01105622	0.88126471	0.22943111	0.00168106
0.0254087	0.73136624	-0.0850551	0.24968277	-0.1515353	0.03949056
-0.0261816	0.72352264	0.07960483	0.28142917	-0.2753018	0.00014903
0.21743459	0.00294868	0.20395111	0.00536	-0.2603575	0.00034469
0.49482317	8.10E-13	0.32053749	8.66E-06	-0.3665328	2.88E-07
-0.3150934	1.25E-05	-0.2597866	0.00035557	0.35355055	7.95E-07
-0.3782421	1.11E-07	-0.1785684	0.01501947	0.38927556	4.34E-08
0.14682429	0.04611797	0.12274762	0.09600603	-0.2910808	5.83E-05
0.09141969	0.21586157	0.00093222	0.98995203	-0.0969244	0.18936106
-0.0260636	0.7247181	0.01401706	0.84980306	-0.2109245	0.00395243
-0.0098405	0.89424018	-0.0447425	0.54535029	-0.201869	0.00585968
-0.1593689	0.03024796	-0.1835165	0.01240357	-0.3357577	2.98E-06
-0.0014761	0.98409081	0.14632309	0.04687478	-0.0147612	0.84193067
0.11676092	0.11347281	0.05007746	0.49844694	-0.0030619	0.9670054
-0.0713337	0.3345966	-0.0127635	0.86309709	-0.0253463	0.73200074
-0.3316929	3.98E-06	-0.1602294	0.02935544	-0.355001	7.11E-07
0.06221323	0.40019593	0.1547007	0.03550339	-0.3629459	3.83E-07
-0.1176953	0.11059405	-0.2484024	0.00065122	0.18388869	0.01222409
-0.1617671	0.02781636	-0.2099712	0.00412281	0.16371344	0.02596758
0.0014669	0.98418958	-0.0615087	0.40556319	0.02198373	0.76645105
-0.0499777	0.49930441	-0.1026421	0.16443891	0.30780363	2.02E-05
-0.012131	0.86982041	-0.1012961	0.17007296	0.23397626	0.00134845
-0.2098132	0.00415169	-0.2799683	0.00011355	0.31669378	1.12E-05
0.12088463	0.10119641	0.03192617	0.66617056	0.18509727	0.01165685
-0.0875487	0.23601882	-0.061536	0.40535457	0.12471128	0.09076628
0.0862437	0.24310269	-0.0416683	0.57332997	0.20132771	0.00599622
-0.1210351	0.10076907	-0.2333343	0.00139145	0.28329309	9.33E-05

-0.109755	0.13695985	-0.258388	0.00038358	0.12830251	0.08177558
-0.1178438	0.11014156	-0.181254	0.01354542	0.2150754	0.00328213
-0.3515294	9.28E-07	-0.2643324	0.00027709	0.18492946	0.0117342
0.14091239	0.05572473	0.1419232	0.05397387	-0.2393589	0.00103294
-0.2446571	0.00078994	-0.1667609	0.02328473	-0.3915795	3.55E-08
-0.1788221	0.01487444	-0.1959257	0.00752279	0.28017138	0.0001122
0.24733467	0.00068828	0.07567274	0.30595194	-0.1692796	0.02125098
0.15440133	0.03586527	0.0180176	0.80767651	-0.2348688	0.00129068
-0.000671	0.99276764	-0.0281516	0.70366042	0.33649899	2.82E-06
-0.0997196	0.17685267	-0.0422745	0.56775957	-0.2722778	0.00017728
0.38015357	9.43E-08	0.0417308	0.57275485	-0.1293629	0.07926236
0.25384189	0.00048938	0.17811623	0.01528096	-0.2883834	6.87E-05
0.23946541	0.00102744	0.11619867	0.11523315	-0.2743548	0.00015739
-0.2665675	0.00024472	-0.1775604	0.01560785	-0.3957265	2.47E-08
-0.0696336	0.34627281	0.03186307	0.66679089	-0.1721863	0.01909634
-0.047521	0.5206543	-0.0590543	0.42459273	0.08860368	0.23039874
-0.3621641	4.07E-07	-0.3155633	1.21E-05	-0.2347656	0.00129724
-0.3788531	1.05E-07	-0.2651633	0.00026462	0.25844652	0.00038236
-0.1875417	0.01057991	-0.3097355	1.78E-05	0.27059116	0.00019514
-0.2660133	0.0002524	-0.2006727	0.00616525	0.22726694	0.00186439
0.12354319	0.09385494	-0.1428966	0.05233114	-0.2157029	0.00319027
-0.1473171	0.04538385	-0.1544669	0.0357858	-0.3143908	1.31E-05
-0.1802737	0.01406807	-0.0452496	0.54080023	-0.1454866	0.04816102
-0.2098859	0.00413837	-0.080473	0.27619813	0.01918042	0.79552913
-0.1901694	0.00952103	-0.2089889	0.00430526	-0.1720464	0.0191955
0.44662875	1.86E-10	0.24093402	0.00095435	-0.1869994	0.01081095
0.36770269	2.62E-07	0.2278549	0.00181286	-0.0907277	0.21937153
-0.1708378	0.02007118	-0.0107723	0.88429198	-0.0229504	0.75649712
0.12300596	0.09530328	0.19512892	0.00777503	-0.1745558	0.01748262
-0.3828687	7.50E-08	-0.3936213	2.97E-08	0.19876497	0.00668204
-0.0407781	0.58155638	-0.2034057	0.00548706	0.13374565	0.06952842
-0.0328863	0.65676033	-0.1611261	0.0284493	0.2611971	0.00032925
0.00081813	0.99118166	-0.0732837	0.321518	0.3473621	1.27E-06
0.16844012	0.02191109	0.21128389	0.00388987	-0.251586	0.00055136
0.31500815	1.26E-05	0.15637982	0.03353037	-0.1013708	0.16975659
-0.0406578	0.58267223	-0.1601909	0.02939493	0.22307674	0.002272
-0.3673937	2.69E-07	-0.3849035	6.31E-08	0.44483998	2.24E-10
-0.0390719	0.59747489	0.10759653	0.14489917	-0.2117361	0.00381242
-0.2655162	0.00025948	-0.272726	0.0001728	-0.2390927	0.00104679
-0.1601033	0.02948483	-0.1079697	0.14350216	-0.4024737	1.35E-08
-0.3046964	2.48E-05	-0.1386645	0.05978685	-0.0546846	0.45972296
0.04307671	0.56042806	0.05941187	0.42178886	-0.1825881	0.0128615
-0.0410013	0.57948928	-0.0904457	0.22081346	0.13226645	0.07269862
0.19473146	0.00790364	0.00586243	0.93687526	0.07670825	0.29936163
-0.1985066	0.00675491	-0.3048697	2.45E-05	0.21697094	0.00301168
-0.317049	1.10E-05	-0.207887	0.00451852	0.10859454	0.14118592

-0.3869379	5.31E-08	-0.2052713	0.00506329	0.00595211	0.93591158
-0.2781703	0.00012617	-0.1879629	0.01040347	0.2218012	0.00241126
-0.1849239	0.01173676	-0.196444	0.00736263	0.14788753	0.04454622
-0.2035087	0.00546285	-0.2997397	3.40E-05	0.36974895	2.22E-07
-0.2429092	0.00086358	-0.0256548	0.72886593	-0.5108436	1.09E-13
-0.1816399	0.01334436	-0.1293503	0.07929188	0.28798061	7.04E-05
-0.1345846	0.06778096	-0.1367879	0.06336203	-0.2162851	0.00310712
-0.1318156	0.07368798	-0.0733861	0.32084011	-0.3118191	1.56E-05
-0.0651284	0.37844012	-0.0628887	0.39508999	0.16166269	0.0279186
0.26009557	0.00034964	0.41535509	4.13E-09	-0.3890993	4.41E-08
-0.156953	0.03287852	-0.0678888	0.35851971	-0.2702895	0.0001985
0.248182	0.00065871	-0.0157102	0.83191458	-0.5283562	1.08E-14
-0.3479175	1.22E-06	-0.3177253	1.05E-05	0.50392609	2.63E-13
0.05582436	0.45040754	0.16365652	0.02602012	0.17032132	0.02045579
-0.1744333	0.01756305	-0.273026	0.00016986	0.13102987	0.075438
-0.1681561	0.02213843	-0.2527288	0.00051911	0.0722871	0.32816001
-0.1195578	0.10502644	-0.0957072	0.19500586	-0.2145527	0.00336047
0.44521328	2.16E-10	0.36174857	4.21E-07	-0.3008591	3.17E-05
-0.1852418	0.01159057	-0.1688613	0.02157771	-0.0336307	0.64950162
-0.3467272	1.33E-06	-0.1678923	0.02235136	0.01421589	0.84769818
-0.2531871	0.00050667	-0.2929871	5.18E-05	0.09341105	0.20598488
-0.0587568	0.42693447	-0.1266555	0.08580581	0.22078093	0.00252819
-0.142003	0.05383754	-0.301925	2.96E-05	0.15570695	0.03430955
-0.1910238	0.00919753	-0.1515224	0.03950747	0.05542128	0.4536898
0.31229205	1.51E-05	0.2312162	0.0015424	-0.0792196	0.2837717
-0.0097653	0.89504345	-0.0834415	0.25881134	-0.3510527	9.62E-07
-0.0580857	0.43224274	0.02642045	0.72110409	-0.3146112	1.29E-05
0.68046433	1.68E-26	0.46671209	2.14E-11	-0.1775101	0.01563774
0.24648538	0.00071914	0.12467814	0.09085278	-0.062615	0.39715407
-0.109898	0.13644587	-0.1842131	0.01206949	0.32280161	7.42E-06
0.00317844	0.96575054	-0.012346	0.86753402	-0.1885341	0.01016838
0.09614619	0.192956	-0.0755113	0.30698765	-0.1619992	0.02759013
-0.0356335	0.63013553	-0.1691715	0.02133502	0.1402213	0.05694859
0.0446908	0.54581541	-0.0364994	0.62183953	0.2489231	0.00063382
-0.0107977	0.88402096	-0.0805203	0.27591522	0.2087391	0.0043528
-0.2782927	0.00012527	-0.1281865	0.0820543	-0.013231	0.858135
-0.1356759	0.0655619	-0.0405911	0.58329171	-0.2310813	0.0015525
0.05597387	0.44919344	-0.0154366	0.83479922	0.04230493	0.56748104
0.29554881	4.42E-05	0.33484859	3.18E-06	-0.243483	0.00083874
-0.061082	0.4088353	-0.1133742	0.12440058	-0.1831637	0.0125759
0.13352495	0.06999416	-0.1340986	0.06878885	-0.3057706	2.31E-05
0.26930318	0.00020988	0.02680678	0.71719851	-0.1470277	0.0458137
-0.1601153	0.02947243	-0.2634943	0.00029023	0.13756675	0.0618575
0.0906947	0.21953999	-0.0629133	0.39490478	-0.3043359	2.53E-05
-0.0571645	0.43959136	-0.1885814	0.01014912	0.15223929	0.0385726
-0.2478115	0.0006715	-0.1235172	0.09392464	0.23694621	0.00116488

-0.2864419	7.73E-05	-0.3448073	1.54E-06	0.15641705	0.0334877
-0.0716252	0.33262009	-0.1524616	0.03828653	0.19174542	0.00893191
-0.1412814	0.05508022	-0.2377878	0.0011172	0.37674839	1.25E-07
-0.3351611	3.11E-06	-0.2173907	0.00295458	-0.4851899	2.57E-12
0.61560995	1.11E-20	0.21436278	0.00338935	-0.09635	0.19200977
-0.2093439	0.00423851	-0.2070529	0.00468624	0.14949415	0.04225642
-0.0647104	0.38151457	-0.0901402	0.22238332	0.17264034	0.01877748
0.10220645	0.16624709	0.01019	0.89050667	-0.1901222	0.00953917
0.10496411	0.15504929	0.01431273	0.84667344	-0.0724461	0.32709451
-0.0785701	0.28775052	-0.0360173	0.62645299	-0.1525812	0.0381333
-0.2130506	0.00359509	-0.2828868	9.56E-05	0.35758204	5.83E-07
-0.003628	0.9609097	-0.1176951	0.11059456	0.25130714	0.0005595
-0.021924	0.76706743	-0.0868955	0.23954625	0.18273055	0.01279028
-0.2389415	0.00105473	-0.1488834	0.04311493	0.26223881	0.00031099
-0.2027211	0.00565037	-0.3029621	2.77E-05	-0.3594686	5.03E-07
0.145943	0.04745562	0.0178053	0.80989932	0.17840298	0.01511467
-0.3277005	5.28E-06	-0.1986309	0.00671978	-0.2090526	0.00429321
0.20395433	0.00535926	0.29273408	5.26E-05	-0.4925519	1.07E-12
-0.0522895	0.47964288	0.03853685	0.60250704	-0.4169036	3.57E-09
-0.1325115	0.07216545	-0.1865325	0.0110134	0.43775334	4.63E-10
0.00293113	0.96841403	-0.1439858	0.05054227	0.14532794	0.0484082
-0.1545967	0.03562876	-0.2379163	0.00111007	0.17022352	0.02052934
-0.2429349	0.00086245	-0.0985398	0.18205568	-0.4211012	2.39E-09
-0.1274665	0.08380181	-0.2376945	0.00112239	0.2878278	7.10E-05
-0.2265516	0.00192888	-0.0347339	0.63880404	-0.2415346	0.00092586
0.11527735	0.1181637	-0.0019474	0.97901196	0.19420405	0.00807723
0.01325672	0.85786177	-0.1169316	0.11294269	0.38784445	4.91E-08
-0.1403974	0.05663461	-0.2913537	5.73E-05	0.09243294	0.21079463
-0.2732823	0.00016739	-0.1819111	0.01320467	-0.350814	9.79E-07
-0.2102421	0.00407373	-0.2212608	0.00247256	-0.061861	0.40287399
0.35289707	8.36E-07	0.25939959	0.00036312	-0.2322767	0.00146505
0.03172279	0.66817069	-0.1570876	0.03272699	-0.2966306	4.13E-05
-0.1801103	0.01415691	-0.0965908	0.19089621	-0.1358003	0.06531273
-0.1078219	0.14405432	-0.147266	0.0454595	0.18906223	0.00995518
-0.2706659	0.00019431	-0.2439877	0.00081743	0.25519343	0.0004554
0.14854277	0.04360012	0.05521611	0.45536562	0.08680785	0.24002229
-0.1752222	0.01705071	-0.1823901	0.01296105	0.21893666	0.00275267
0.25202964	0.00053862	0.10164474	0.16860017	0.01700924	0.81824779
0.23866528	0.00106939	0.19306502	0.00846372	-0.1565583	0.03332619
-0.0885323	0.23077573	-0.185048	0.01167949	0.21525982	0.00325488
-0.1108313	0.13312761	-0.2096284	0.00418567	-0.0738294	0.31791763
-0.0487461	0.5099492	-0.0202737	0.78415265	-0.3916114	3.54E-08
-0.1295639	0.07879311	-0.0987426	0.1811536	0.17176586	0.01939578
-0.0975691	0.18642017	0.06981725	0.3449991	-0.4056999	1.01E-08
-0.1024341	0.16530051	-0.0858397	0.24532542	0.11462567	0.12027133
0.19837643	0.00679191	0.06567276	0.37445953	0.05541813	0.45371545

-0.3774634	1.18E-07	-0.3255665	6.13E-06	0.36676198	2.83E-07
-0.146991	0.04586854	-0.1936523	0.00826243	0.18080524	0.01378254
-0.14903	0.04290753	-0.2971705	4.00E-05	0.37629835	1.30E-07
-0.2225048	0.00233351	-0.2452698	0.00076553	0.4404052	3.54E-10
-0.1056211	0.15246719	-0.0832994	0.25962624	-0.136448	0.06402777
-0.0602637	0.41515205	-0.1336703	0.06968721	0.08106324	0.27267965
0.50526501	2.22E-13	0.21437749	0.0033871	-0.2121772	0.00373821
0.02171682	0.76920634	0.02721033	0.71312668	-0.3377968	2.57E-06
-0.2779645	0.00012769	-0.2212361	0.0024754	0.22648639	0.00193486
0.0646063	0.38228277	-0.0704616	0.34055451	0.1204044	0.10256965
-0.3249068	6.41E-06	-0.1072863	0.14606827	-0.4241907	1.78E-09
0.05624072	0.44703114	0.15229311	0.03850318	-0.3503286	1.02E-06
-0.3650282	3.24E-07	-0.3149212	1.27E-05	-0.4860842	2.32E-12
-0.2398934	0.00100563	0.01138977	0.87771023	-0.0750215	0.31014537
-0.0679098	0.35837108	-0.1991197	0.00658312	0.31941151	9.35E-06
-0.0027301	0.97057952	-0.0114923	0.87661792	0.38136021	8.52E-08
-0.3056625	2.33E-05	-0.1991326	0.00657954	0.34519193	1.49E-06
-0.4759306	7.56E-12	-0.4071294	8.86E-09	0.46299459	3.23E-11
0.03971165	0.59148341	0.01212123	0.86992392	-0.212687	0.00365405
-0.3385441	2.44E-06	-0.2001662	0.00629886	0.32564797	6.09E-06
0.03163885	0.66899682	0.0126635	0.86415961	-0.1799649	0.01423636
-0.1212779	0.10008265	-0.071711	0.33203948	0.01227254	0.86831476
0.12307962	0.09510364	0.00147184	0.98413627	0.28784584	7.10E-05
0.09879771	0.18090886	-0.0010196	0.98901059	0.26098247	0.00033313
-0.3119482	1.54E-05	-0.1405506	0.05636272	0.05058227	0.49412167
-0.0642287	0.38507667	0.02116343	0.77492805	-0.14351	0.05131732
0.11341497	0.12426424	0.07404304	0.31651556	0.19558806	0.00762876
-0.2799229	0.00011386	-0.3371461	2.70E-06	0.33013911	4.45E-06
0.10600579	0.15097056	-0.0434255	0.55725477	0.11837663	0.10853099
-0.1153523	0.11792317	0.00174604	0.98118148	-0.2620209	0.00031473
0.02161896	0.77021728	0.02908519	0.69431717	-0.3159421	1.18E-05
-0.2405479	0.00097309	-0.2349403	0.00128615	0.16724478	0.02288152
-0.1236715	0.09351159	-0.1126783	0.12674364	0.36635503	2.92E-07
0.04659322	0.52883653	-0.1127544	0.12648566	0.05149871	0.48632069
-0.1251283	0.08968341	-0.0278935	0.70625161	-0.2334511	0.00138353
0.05907826	0.42440471	0.01539974	0.83518856	-0.3088741	1.89E-05
0.05408054	0.4647031	-0.0023226	0.97496913	0.20773528	0.00454863
-0.2913755	5.72E-05	-0.2087553	0.00434969	0.28824333	6.93E-05
-0.0750091	0.31022508	-0.1698505	0.02081195	0.26021128	0.00034744
-0.242771	0.00086966	-0.0731585	0.32234756	0.18057347	0.01390642
-0.2599091	0.0003532	-0.0204228	0.78260509	-0.2284269	0.00176398
0.11790106	0.10996769	0.11335263	0.1244726	0.18721173	0.01071996
-0.1868872	0.0108593	-0.0322979	0.66252078	-0.0519038	0.48289369
-0.0275988	0.70921436	-0.0957858	0.19463774	-0.3600416	4.81E-07
0.4125661	5.36E-09	0.12620099	0.08694547	-0.2726819	0.00017324
0.16680506	0.0232477	0.17248584	0.01888546	-0.0464959	0.52969899

0.04596257	0.53443531	-0.0170066	0.81827522	0.21089358	0.00395786
-0.0857485	0.24582926	-0.1982128	0.00683866	0.29798132	3.80E-05
-0.0518274	0.48353878	-0.1651787	0.02464622	0.01307932	0.85974427
-0.0718547	0.33106929	-0.0531417	0.47250188	-0.1323252	0.07257059
-0.4240756	1.80E-09	-0.2797615	0.00011494	0.03118435	0.67347713
0.04227386	0.56776581	-0.0452433	0.54085656	0.20695483	0.00470632
-0.1506834	0.04062573	-0.2040784	0.00533072	0.14658447	0.04647881
-0.1491185	0.04278282	-0.0655626	0.37526314	-0.2589808	0.00037146
0.01925441	0.79475782	-0.0874743	0.23641855	0.05912668	0.42402446
-0.2984689	3.68E-05	-0.1982213	0.00683624	0.22360622	0.00221637
0.4542274	8.35E-11	0.32792103	5.20E-06	0.19850509	0.00675535
-0.0289825	0.69534222	-0.0795939	0.2814952	0.02449552	0.74066973
0.19093389	0.00923109	0.04368971	0.55485635	0.11983254	0.10422405
-0.1065056	0.14904239	-0.1732872	0.01833121	-0.1594935	0.0301173
-0.2553998	0.00045041	-0.228645	0.00174566	0.06765282	0.36019697
-0.1666019	0.02341859	-0.1933431	0.00836786	0.11447026	0.12077822
0.57787921	7.16E-18	0.27848796	0.00012385	-0.358573	5.39E-07
-0.1465858	0.04647686	-0.0472555	0.52298881	0.14202311	0.05380331
-0.1902422	0.00949307	-0.0198604	0.78844793	-0.3019939	2.94E-05
0.02630332	0.72228956	-0.1725063	0.01887113	0.18596018	0.01126614
0.00769005	0.91725771	-0.0258969	0.72640831	0.21403168	0.00344023
-0.1475822	0.04499299	-0.1250225	0.08995729	0.30095015	3.15E-05
0.26176003	0.00031926	0.17430216	0.0176495	-0.2996531	3.42E-05
-0.1818791	0.01322109	0.03978244	0.59082209	-0.2683939	0.00022091
-0.2098045	0.00415328	-0.1277619	0.08308121	-0.2503504	0.0005883
0.16974582	0.02089185	-0.1078016	0.14413028	0.30858623	1.92E-05
-0.0448613	0.54428246	-0.1001606	0.1749366	0.41992843	2.68E-09
-0.0649122	0.38002851	-0.128779	0.08063847	0.10485844	0.15546765
0.07738997	0.29507465	-0.0183669	0.80402291	0.29240522	5.37E-05
0.13365411	0.06972129	0.04289597	0.56207604	0.32184757	7.92E-06
-0.0386746	0.60120934	-0.1035381	0.16076688	0.10561756	0.1524811
0.11189305	0.12942822	0.06293759	0.39472167	0.17294394	0.01856686
0.11567647	0.11688713	0.07582021	0.3050076	0.2283252	0.00177258
-0.0653713	0.37666072	-0.188713	0.0100957	0.18460959	0.01188289
0.0443521	0.54886614	-0.0895762	0.22530231	0.23037695	0.00160623
-0.2474898	0.00068278	-0.1320777	0.07311139	0.13674631	0.06344313
-0.1100693	0.13583234	-0.1509401	0.04028082	-0.0349783	0.63644424
0.148479	0.04369145	-0.0810943	0.27249564	0.12905341	0.07998941
-0.0623388	0.39924393	-0.1627157	0.02690169	0.28661967	7.64E-05
0.38310038	7.36E-08	0.16572896	0.02416519	0.21050617	0.00402641
-0.192337	0.00871924	0.04369528	0.55480591	-0.1212229	0.10023779
-0.2443954	0.00080059	-0.2393207	0.00103491	0.39889721	1.87E-08
0.04372741	0.55451459	-0.0656005	0.37498681	0.33722067	2.68E-06
-0.201637	0.00591785	-0.2524086	0.00052796	0.20088929	0.00610889
0.11222678	0.12828191	0.00130501	0.98593427	0.38994377	4.10E-08
-0.049381	0.50444723	-0.1636377	0.02603747	0.27040077	0.00019725

-0.0597264	0.41933112	-0.0613543	0.4067451	-0.2843117	8.78E-05
0.14543265	0.04824491	-0.0389101	0.59899469	0.15112188	0.04003807
-0.2578081	0.00039578	-0.2018122	0.00587388	-0.3833931	7.18E-08
-0.3084934	1.94E-05	-0.1228054	0.0958485	-0.144118	0.05032872
0.15132445	0.03976897	-0.0781078	0.29060461	-0.0823045	0.26538004
-0.2000441	0.00633146	-0.0219002	0.76731296	-0.0941936	0.20219405
-0.4030799	1.28E-08	-0.2850496	8.40E-05	0.08685221	0.23978128
-0.3659084	3.02E-07	-0.208875	0.00432688	-0.4018161	1.44E-08
0.25193441	0.00054133	0.11083131	0.13312753	-0.0155601	0.83349719
-0.3403189	2.14E-06	-0.3910585	3.72E-08	0.15789705	0.03182835
0.13458821	0.06777359	0.01475948	0.84194922	0.33701169	2.72E-06
-0.0591139	0.42412456	-0.1380921	0.06085929	0.1841661	0.01209179
-0.2123169	0.00371496	-0.1932116	0.00841307	0.17387444	0.01793404
-0.3394512	2.28E-06	-0.3342786	3.31E-06	-0.2919925	5.51E-05
0.15147673	0.03956769	-0.0770167	0.29741694	-0.2891539	6.56E-05
-0.4293966	1.07E-09	-0.4508256	1.20E-10	0.28940779	6.45E-05
0.17423576	0.01769341	-0.0294063	0.69111382	0.09580474	0.19454888
-0.039966	0.58910935	-0.0923425	0.21124363	0.07657255	0.30021987
0.0065978	0.92897663	-0.0030404	0.96723695	0.15501728	0.03512405
-0.3171186	1.09E-05	-0.1534468	0.03704005	0.22601725	0.00197837
-0.0823748	0.26497046	-0.115283	0.11814569	0.37442599	1.52E-07
-0.0700033	0.3437116	-0.0848554	0.25080043	-0.2339292	0.00135156
-0.2252012	0.00205619	-0.159834	0.02976277	-0.4027035	1.33E-08
-0.3672204	2.72E-07	-0.3038877	2.61E-05	0.49392311	9.04E-13
-0.1025987	0.16461834	-0.2579486	0.00039279	0.45672211	6.39E-11
-0.4661724	2.27E-11	-0.3750869	1.44E-07	0.35521975	6.99E-07
-1.94E-06	0.9999791	-0.0387184	0.60079755	-0.1432379	0.051765
-0.1932101	0.00841359	-0.3192609	9.45E-06	0.13734119	0.06229015
0.00447414	0.95180304	-0.0903633	0.22123631	0.18570943	0.01137846
0.08049602	0.2760604	-0.0033382	0.96403016	0.20173927	0.00589215
-0.0141882	0.84799148	-0.1820407	0.01313835	0.24470941	0.00078783
-0.0973326	0.18749501	-0.0414762	0.57510064	-0.1129763	0.125736
0.43283296	7.60E-10	0.23788987	0.00111153	-0.2393017	0.0010359
-0.1277589	0.08308865	-0.1405362	0.05638819	0.31415165	1.33E-05
-0.1211992	0.10030472	-0.218591	0.0027967	0.1988333	0.00666288
-0.1371059	0.06274414	-0.0480826	0.51573241	-0.1112745	0.13157366
0.14450164	0.04971298	0.03045957	0.68064548	0.17344977	0.01822049
0.11364581	0.12349513	-0.0854011	0.24775444	-0.0744776	0.31367553
-0.2609783	0.00033321	-0.2718713	0.00018144	0.01689805	0.81941546
-0.042567	0.56508158	-0.086997	0.23899583	-0.0919502	0.2131978
-0.2140695	0.00343439	-0.2662037	0.00024973	0.01556573	0.83343773
-0.0994139	0.1781904	-0.1555192	0.03452968	0.22509881	0.00206614
0.22265665	0.00231703	0.23585592	0.00122943	-0.1885679	0.01015463
-0.1037338	0.15997273	-0.05287	0.4747722	0.36267141	3.91E-07
-0.3764048	1.29E-07	-0.2823629	9.86E-05	-0.33784	2.56E-06
-0.096743	0.19019482	-0.0475118	0.52073452	-0.3064591	2.21E-05

0.38950371	4.26E-08	0.31814069	1.02E-05	-0.3973838	2.13E-08
-0.1593578	0.03025964	-0.1636461	0.02602974	0.19568643	0.00759775
-0.1989527	0.00662952	-0.1573019	0.03248703	-0.2091769	0.0042698
-0.1227206	0.09607968	-0.1320849	0.07309566	0.18383431	0.01225018
-0.0673851	0.3621051	-0.1321092	0.07304252	0.13110526	0.07526864
-0.0503891	0.4957743	-0.0917008	0.21444698	0.455159	7.56E-11
-0.1426717	0.05270694	0.00414181	0.9553791	0.04883136	0.50920854
-0.1006598	0.17278587	-0.0954342	0.19628847	0.24658676	0.00071539
-0.1707237	0.0201556	-0.1484294	0.04376256	0.04734301	0.52221878
0.1096869	0.13720521	-0.0698138	0.34502296	-0.152348	0.03843247
-0.0312286	0.6730401	0.08195533	0.26741969	-0.2013308	0.00599543
-0.3572513	5.98E-07	-0.332118	3.87E-06	-0.3644036	3.41E-07
-0.0807835	0.27434375	-0.1661386	0.0238123	0.46086485	4.08E-11
0.04058122	0.58338354	-0.0056135	0.93955067	-0.3216194	8.04E-06
0.00132114	0.98576037	-0.0532823	0.4713294	-0.5217679	2.62E-14
-0.3059987	2.28E-05	-0.314967	1.26E-05	0.14672018	0.04627433
0.29378576	4.93E-05	0.19422604	0.00806993	-0.2540829	0.00048315
-0.1800587	0.01418506	-0.2189286	0.00275369	0.255995	0.00043629
-0.2554132	0.00045008	-0.0226123	0.75997421	-0.1663322	0.02364714
-0.2745314	0.0001558	-0.1924177	0.00869061	0.20067001	0.00616595
-0.242112	0.00089921	-0.245706	0.00074857	0.10416333	0.15824077
0.02862422	0.69892507	-0.0109204	0.8827128	0.17303273	0.01850565
-0.0517651	0.48406558	-0.2391823	0.00104211	0.15614639	0.03379896
0.167846	0.02238889	-0.0768109	0.29871348	0.15784238	0.03188839
-0.1585069	0.03116525	-0.2695635	0.00020682	0.36345964	3.67E-07
-0.1358282	0.06525684	-0.0487644	0.50979062	-0.4206792	2.49E-09
-0.2154009	0.00323418	-0.2341053	0.00133995	0.26260786	0.00030474
-0.1506682	0.04064631	-0.0616112	0.40477955	0.44927355	1.41E-10
-0.1804008	0.01399936	-0.2130829	0.00358989	-0.2167274	0.00304526
0.17488436	0.0172685	0.05898096	0.42516946	0.13973195	0.05782853
-0.2435786	0.00083466	-0.2019388	0.00584229	-0.3455232	1.46E-06
-0.123799	0.09317137	-0.0275966	0.70923631	-0.2361761	0.00121014
-0.4132697	5.02E-09	-0.3882124	4.76E-08	-0.0445572	0.54701772
0.23228758	0.00146427	0.04670664	0.52783272	0.13717137	0.06261753
0.33696334	2.73E-06	0.25557129	0.0004463	-0.2431009	0.0008552
-0.1380193	0.06099696	-0.0943506	0.20143991	0.35204086	8.92E-07
0.23205534	0.00148089	0.14902712	0.04291162	0.07411332	0.31605519
-0.2246824	0.00210709	-0.1848594	0.0117666	-0.3585981	5.38E-07
-0.0618133	0.40323732	-0.1345386	0.06787595	0.43833417	4.37E-10
-0.3014188	3.05E-05	-0.2129066	0.00361833	0.20765489	0.00456465
-0.0413144	0.57659405	-0.1383343	0.0604037	-0.3683421	2.49E-07
-0.0750252	0.3101214	-0.2503643	0.00058787	-0.1530206	0.03757496
0.12079757	0.10144428	-0.0491759	0.50622078	0.24275368	0.00087042
0.0485958	0.51125644	-0.0295953	0.689231	-0.4053811	1.04E-08
0.23967771	0.00101657	0.4288511	1.13E-09	-0.3879694	4.86E-08
0.21203406	0.00376214	0.01437223	0.84604397	-0.2636856	0.00028718

-0.4471971	1.75E-10	-0.312353	1.50E-05	-0.3764071	1.29E-07
-0.0506678	0.49339096	-0.1038444	0.15952558	-0.5754744	1.05E-17
-0.1435676	0.051223	-0.1035875	0.16056593	-0.2258338	0.00199563
-0.0550901	0.45639658	-0.0335624	0.65016634	0.40893622	7.51E-09
0.01886766	0.79879165	0.3061573	2.25E-05	-0.1752365	0.01704153
-0.1231042	0.09503701	-0.128248	0.0819065	-0.0193899	0.79334638
0.20961048	0.00418899	0.03845335	0.60329398	-0.0972485	0.18787849
-0.2756159	0.00014635	-0.2772769	0.00013291	0.18626457	0.01113109
-0.0938649	0.20378027	0.02366742	0.7491392	0.23341907	0.00138571
-0.0404571	0.58453656	-0.0967799	0.19002499	0.24314565	0.00085326
0.04416732	0.55053399	-0.0502243	0.49718715	0.2466258	0.00071395
0.03764933	0.61089533	-0.0371875	0.61528065	-0.1410081	0.05555703
0.07514874	0.30932286	0.06939803	0.34791057	-0.1453008	0.04845058
-0.3850734	6.22E-08	-0.3640631	3.50E-07	0.46526004	2.52E-11
-0.3500449	1.04E-06	-0.3242592	6.71E-06	0.33389366	3.41E-06
-0.0415458	0.57445927	0.00348998	0.96239585	-0.2659863	0.00025278
-0.0810168	0.27295511	-0.1296575	0.07857547	0.00890763	0.90421535
0.08698475	0.23906202	0.10736985	0.14575284	0.0629379	0.39471937
-0.2845941	8.63E-05	-0.2247267	0.0021027	-0.2009593	0.00609078
-0.139751	0.05779399	-0.0334205	0.6515479	-0.3778621	1.14E-07
0.50353426	2.76E-13	0.26798751	0.00022602	-0.2040182	0.00534455
0.10489107	0.15533837	0.03278889	0.65771258	-0.3759169	1.34E-07
0.16504665	0.02476292	0.14128599	0.05507221	-0.3558967	6.64E-07
0.02189843	0.76733124	0.00246548	0.97342982	-0.3920963	3.40E-08
-0.0063704	0.93141835	-0.080372	0.27680357	0.18255797	0.01287659
-0.3997186	1.73E-08	-0.3170566	1.10E-05	-0.0156003	0.83307331
-0.1385299	0.06003777	-0.1274876	0.08375017	0.19051324	0.00938964
-0.1008076	0.1721528	-0.1631308	0.02650967	0.34381185	1.66E-06
-0.1045015	0.15688684	-0.0501488	0.49783472	-0.0730566	0.32302346
-0.0838501	0.25647829	-0.146185	0.04708511	0.31128335	1.61E-05
0.00671368	0.9277325	0.04624555	0.53191944	-0.1686238	0.02176515
0.12345092	0.09410244	0.04305394	0.56063559	-0.097106	0.1885294
0.07787707	0.2920366	0.02967522	0.68843543	-0.1255448	0.08861222
0.22153506	0.00244128	0.15835231	0.0313322	-0.3985709	1.92E-08
0.69010655	1.69E-27	0.48848796	1.74E-12	-0.2192074	0.00271862
-0.4450495	2.19E-10	-0.3721479	1.83E-07	0.38022509	9.38E-08
-0.0424958	0.56573312	-0.0886404	0.23020503	-0.1636798	0.02599863
-0.1875046	0.01059558	-0.2244252	0.00213276	0.20819319	0.00445834
-0.1074883	0.14530612	-0.1105946	0.13396334	0.0672225	0.36326748
-0.3841187	6.75E-08	-0.3799712	9.58E-08	0.099778	0.17659817
-0.1193313	0.10569148	-0.1610772	0.02849814	-0.0640931	0.38608229
-0.1804397	0.01397838	-0.1652509	0.02458265	-0.4253559	1.59E-09
-0.174353	0.01761595	-0.2079076	0.00451446	-0.1821028	0.01310671
0.09669742	0.19040454	-0.0125606	0.86525261	-0.0837169	0.25723752
-0.0199971	0.78702701	0.04220715	0.56837756	0.25836305	0.00038409
0.18225073	0.01303152	0.06678245	0.36642418	0.07087151	0.33774556

0.09118651	0.21703985	0.07907058	0.28468107	-0.2487899	0.00063823
0.07788002	0.29201828	0.19828965	0.00681666	-0.3513165	9.43E-07
-0.0532126	0.47191098	-0.2219963	0.00238947	-0.1435403	0.05126763
-0.1481308	0.04419302	0.01285321	0.86214461	0.27220091	0.00017806
-0.0498823	0.50012455	0.00390485	0.95792957	0.19482175	0.00787426
0.17796598	0.01536873	0.01195278	0.87171592	0.17595015	0.01658954
0.07224943	0.32841286	-0.0497327	0.50141263	0.20681818	0.00473443
-0.1262056	0.08693393	-0.2300665	0.00163046	0.07128281	0.33494226
-0.0019274	0.97922667	-0.0237625	0.74816493	-0.2317764	0.00150109
-0.3248782	6.43E-06	-0.2317871	0.0015003	-0.3531365	8.21E-07
0.02852951	0.69987315	-0.133111	0.07087453	0.13142276	0.07455874
0.26826568	0.00022251	0.05343875	0.47002675	0.31678807	1.12E-05
0.14474879	0.04931969	0.04077485	0.58158696	0.14311833	0.05196274
0.07196879	0.33030003	-0.0940048	0.20310411	0.23879388	0.00106254
-0.0894088	0.22617357	-0.1848079	0.01179049	0.13531298	0.06629313
-0.4815822	3.93E-12	-0.3999895	1.69E-08	0.40198436	1.41E-08
0.05296325	0.47399244	-0.1257286	0.08814282	0.33768356	2.59E-06
-0.0466844	0.52802916	0.02925199	0.69265248	-0.2445653	0.00079366
0.34033227	2.14E-06	0.02544699	0.730977	-0.3630718	3.79E-07
0.17054394	0.02028923	0.02098839	0.77674051	0.08481796	0.25101008
-0.114746	0.11987996	-0.1111031	0.13217305	-0.1670112	0.02307544
0.39416532	2.84E-08	0.10264574	0.164424	-0.1170472	0.11258446
-0.3324906	3.77E-06	-0.2240582	0.00216985	0.38108035	8.73E-08
-0.2946329	4.68E-05	-0.3628871	3.85E-07	0.05327272	0.47140939
-0.2352222	0.00126844	0.0152692	0.83656596	-0.4583582	5.35E-11
-0.058422	0.42957773	-0.1628346	0.0267889	0.14905067	0.04287839
0.22836883	0.00176889	-0.0390346	0.59782505	-0.302711	2.81E-05
0.25101196	0.00056825	-0.0090892	0.90227206	0.10749114	0.1452956
0.01557694	0.83331961	-0.13949	0.05826774	0.13810353	0.06083779
-0.2085247	0.00439397	-0.1092355	0.13883953	-0.2492211	0.00062406
-0.1211864	0.10034102	-0.0244364	0.74127385	-0.2365308	0.0011891
0.16060163	0.02897634	-0.03768	0.61060414	0.08716197	0.23810276
-0.0005999	0.99353367	-0.0142245	0.84760693	-0.2418792	0.00090987
0.03535132	0.63284931	0.03118674	0.67345357	-0.3749214	1.46E-07
-0.0449906	0.54312215	-0.0166785	0.82172187	-0.3476643	1.24E-06
-0.003792	0.95914442	-0.0829041	0.26190164	0.30878041	1.90E-05
-0.1411551	0.05530013	-0.059741	0.419217	0.09204404	0.21272918
-0.2356607	0.00124133	-0.0508821	0.49156174	-0.3560955	6.54E-07
-0.2456904	0.00074918	-0.1839821	0.01217939	-0.2058722	0.00493317
0.16621647	0.02374575	0.19862786	0.00672063	-0.2561252	0.00043326
-0.0216595	0.7697983	-0.1222979	0.09723924	0.21679138	0.00303641
-0.1375439	0.06190121	-0.0438838	0.55309784	-0.1455522	0.04805904
0.19694954	0.00720934	0.10852369	0.14144709	-0.1482106	0.04407759
-0.274169	0.00015908	-0.416938	3.56E-09	0.23818512	0.00109531
-0.1003458	0.17413609	-0.1036128	0.16046328	0.09029162	0.22160434
0.25051889	0.00058313	0.09133929	0.21626731	0.30601514	2.27E-05

-0.1338577	0.06929288	-0.1629111	0.02671652	-0.159955	0.0296376
-0.2987792	3.61E-05	-0.2481893	0.00065846	0.26306443	0.00029718
-0.4510708	1.17E-10	-0.3124557	1.49E-05	-0.2045375	0.00522633
-0.0543988	0.46207563	-0.1335069	0.07003239	-0.2933241	5.08E-05
-0.2918714	5.55E-05	-0.2653812	0.00026143	0.26193445	0.00031622
-0.1194816	0.10524973	-0.2210605	0.00249565	0.3962484	2.36E-08
0.17915553	0.01468574	-0.0003483	0.99624551	-0.1559392	0.03403892
-0.153106	0.03746734	-0.1461939	0.04707149	0.24520068	0.00076825
0.22508857	0.00206714	-0.0686763	0.35295907	0.25235099	0.00052957
-0.3256836	6.08E-06	-0.2625816	0.00030518	0.11861066	0.10782941
-0.3108777	1.66E-05	-0.2371345	0.00115405	0.0784317	0.28860282
-0.0048642	0.94760724	0.03174431	0.6679589	0.14020882	0.05697089
-0.0595374	0.42080724	-0.1074434	0.14547529	0.40840012	7.89E-09
-0.1673244	0.02281575	-0.1409244	0.05570363	0.18330788	0.01250522
-0.3576513	5.80E-07	-0.3597133	4.93E-07	0.19908529	0.00659266
0.07018886	0.34243115	-0.0427484	0.56342288	0.25867329	0.0003777
0.06484376	0.38053229	0.10985178	0.1366119	0.31424886	1.32E-05
0.01022107	0.89017484	0.00106262	0.98854659	-0.3016201	3.02E-05
-0.1372132	0.0625367	-0.1716899	0.0194503	0.17308911	0.01846687
-0.2063024	0.00484188	-0.1040382	0.15874381	-0.2292228	0.00169796
-0.1791403	0.01469434	-0.0953068	0.1968889	-0.2277503	0.00182193
-0.1412556	0.05512499	-0.2433544	0.00084424	0.20641815	0.00481758
-0.3173913	1.07E-05	-0.3195783	9.25E-06	0.21370246	0.00349151
-0.0917936	0.21398198	-0.0610019	0.40945111	0.07002502	0.34356185
-0.030982	0.67547571	-0.0543715	0.46230049	-0.2204889	0.00256259
-0.0643584	0.38411563	-0.2230794	0.00227171	0.18220853	0.01305292
0.12582875	0.08788789	-0.0179066	0.80883884	0.33786464	2.56E-06
0.048554	0.51162024	-0.0894315	0.22605546	0.17166522	0.01946805
-0.1650964	0.02471888	-0.1727727	0.01868541	0.26186381	0.00031745
-0.045039	0.54268774	-0.1732555	0.01835288	0.52613725	1.46E-14
0.32547967	6.16E-06	0.30996284	1.76E-05	-0.2221799	0.00236913
-0.247181	0.00069377	-0.3054592	2.36E-05	0.34977258	1.06E-06
-0.0791486	0.28420465	-0.0997782	0.17659739	0.32854302	4.98E-06
-0.0543724	0.46229343	-0.1187768	0.10733362	0.09753032	0.18659627
-0.0180475	0.80736396	-0.0597663	0.41901968	0.17874692	0.01491729
-0.2087361	0.00435338	-0.1621442	0.02744963	0.17756715	0.01560385
-0.0458104	0.53579066	-0.1077063	0.14448717	0.15007894	0.04144785
-0.2761723	0.00014171	-0.2850088	8.42E-05	-0.4537203	8.81E-11
0.19658022	0.00732103	-0.0415396	0.57451651	-0.3412304	2.00E-06
0.30665912	2.18E-05	0.27949639	0.00011674	0.17271071	0.01872848
-0.2090053	0.00430216	-0.2922461	5.43E-05	0.31728531	1.08E-05
0.18367745	0.01232569	0.03821671	0.60552682	0.17844055	0.015093
-0.0281802	0.70337437	-0.0862321	0.24316609	0.21678879	0.00303677
-0.1164982	0.11429263	-0.1011496	0.17069472	0.1862533	0.01113607
-0.157982	0.03173526	-0.1689037	0.02154442	0.12185748	0.09845901
-0.2932943	5.09E-05	-0.1635726	0.02609775	-0.2235479	0.00222243

0.23381933	0.00135885	-0.0868668	0.23970217	0.03293074	0.656326
0.10943361	0.1381205	0.01360632	0.85415459	-0.0343146	0.64286158
0.60233655	1.19E-19	0.42049301	2.54E-09	-0.2643099	0.00027744
0.42425198	1.77E-09	0.37214796	1.83E-07	-0.1528044	0.03784879
-0.2036988	0.00541846	-0.2182121	0.0028457	-0.3346761	3.22E-06
0.31142265	1.60E-05	0.18562927	0.01141458	-0.2500112	0.00059884
0.07339855	0.32075789	-0.0239468	0.74627877	-0.0430697	0.56049236
-0.0457086	0.53669862	-0.0366446	0.62045285	-0.0497123	0.50158833
-0.2858335	8.01E-05	-0.1927237	0.00858268	-0.0902514	0.22181084
-0.0073691	0.92070004	-0.0803224	0.27710094	-0.1250941	0.08977187
-0.0353697	0.63267212	-0.0588488	0.42620907	0.22357815	0.00221928
-0.1501344	0.04137179	-0.2946934	4.66E-05	0.24254795	0.00087956
-0.137665	0.06166978	0.00392779	0.95768265	-0.2291303	0.00170552
0.32333594	7.15E-06	0.21855113	0.00280182	-0.1301643	0.07740487
-0.3619556	4.14E-07	-0.2044061	0.00525603	0.4792271	5.17E-12
-0.5224677	2.39E-14	-0.2918781	5.55E-05	-0.4527627	9.76E-11
-0.1661735	0.02378243	-0.1590519	0.03058256	0.07667416	0.29957708
-0.1452205	0.04857625	-0.2236761	0.00220912	0.33027201	4.41E-06
-0.0095743	0.89708464	0.07928219	0.28339001	-0.2419424	0.00090697
-0.1084504	0.14171771	-0.235472	0.00125292	-0.4441481	2.41E-10
-0.1943795	0.0080191	-0.164391	0.02534918	0.19653104	0.00733602
0.06975951	0.34539919	0.16788545	0.02235689	-0.3033305	2.70E-05
0.03622607	0.62445315	-0.0049979	0.94616857	-0.1400176	0.0573135
-0.0786009	0.28756068	-0.1534331	0.03705717	0.14001493	0.05731832
0.43452236	6.42E-10	0.45712249	6.12E-11	-0.3863106	5.60E-08
0.02879685	0.69719814	-0.10959	0.13755476	0.21956737	0.00267394
0.12906025	0.07997329	-0.0104464	0.88776961	0.20158045	0.00593211
-0.065889	0.37288527	0.0576098	0.43603026	0.25001861	0.00059861
0.01738771	0.81427605	-0.0440205	0.55186098	0.21012133	0.00409555
-0.0145464	0.84420136	0.07836144	0.28903631	-0.2795977	0.00011605
0.18734334	0.0106639	0.16010578	0.02948226	-0.2584032	0.00038326
-0.0769712	0.29770319	0.15910107	0.0305305	-0.3256826	6.08E-06
-0.2116931	0.00381971	-0.1026017	0.16460606	0.0402195	0.58674667
0.24764397	0.00067735	0.17700756	0.01593902	-0.2710628	0.00018998
-0.0025234	0.97280585	-0.127359	0.08406534	0.08588086	0.24509838
0.03198785	0.66556446	-0.0541979	0.46373353	0.08060773	0.27539237
0.31426575	1.32E-05	0.0030331	0.96731579	-0.102277	0.16595327
-0.0730425	0.32311703	0.1034928	0.16095089	-0.313622	1.38E-05
-0.112212	0.12833252	-0.1604946	0.02908491	0.17575975	0.01670911
-0.1734787	0.01820086	-0.2795364	0.00011647	0.19154888	0.00900356
-0.4276634	1.27E-09	-0.3240961	6.78E-06	0.33427597	3.31E-06
0.18464957	0.01186422	0.00534526	0.94243393	-0.0757895	0.30520424
-0.1757629	0.01670712	-0.1186237	0.10779049	-0.2730114	0.00017
-0.0628682	0.3952442	-0.166499	0.02350552	0.19355727	0.00829472
-0.1707446	0.02014008	-0.0676651	0.36010991	0.13635326	0.06421449
0.45236222	1.02E-10	0.26375495	0.00028608	-0.3578421	5.71E-07

-0.0640563	0.3863559	-0.2467386	0.00070981	0.13197553	0.07333571
-0.0829312	0.26174563	-0.156796	0.03305604	0.19439085	0.00801536
0.156871	0.03297111	-0.0406297	0.58293342	-0.2404697	0.00097693
-0.0349861	0.63636912	0.06712706	0.36395071	-0.2294621	0.00167856
-0.0789242	0.2855763	-0.1146772	0.12010353	0.18823168	0.01029226
-0.1097491	0.13698114	-0.1334027	0.0702533	0.08212998	0.26639804
-0.1947805	0.00788767	-0.1852532	0.01158537	0.13135434	0.07471124
-0.1476302	0.04492243	-0.1750994	0.01712962	0.09509163	0.19790639
0.05146712	0.48658849	-0.1271664	0.08453881	0.20625388	0.0048521
0.05068739	0.49322352	0.08427397	0.25407348	0.13335984	0.07034424
-0.0913215	0.21635732	-0.1248223	0.09047697	0.12133219	0.09992968
0.03961222	0.59241284	-0.0665383	0.36818258	0.3173224	1.08E-05
0.15019232	0.0412926	0.02830371	0.70213548	0.2377197	0.00112099
0.31120526	1.62E-05	0.08535251	0.24802473	-0.3086487	1.92E-05
0.12397007	0.09271663	0.18858611	0.0101472	-0.2757625	0.00014511
0.18846079	0.01019829	0.09412191	0.20253927	-0.3409513	2.04E-06
-0.1581536	0.03154793	0.01109347	0.88086765	0.23114202	0.00154794
-0.0389226	0.59887731	-0.1471221	0.04567306	0.19901247	0.00661288
0.18407836	0.01213349	0.08569391	0.2461311	0.06764481	0.36025395
-0.0611731	0.40813531	-0.010567	0.8864823	0.30514784	2.41E-05
-0.0873537	0.23706777	-0.0102163	0.8902259	0.28164446	0.00010287
-0.0836894	0.25739417	-0.190026	0.00957631	0.11152754	0.13069268
-0.1290331	0.0800372	-0.0893488	0.22648673	0.01317152	0.85876575
-0.0346401	0.63971027	-0.0992524	0.17889993	0.20235654	0.00573908
-0.0246064	0.73953759	0.10471077	0.15605369	0.05885965	0.42612396
0.03582047	0.62834034	-0.0766776	0.29955551	0.20126138	0.00601315
-0.1840896	0.01212814	-0.1016838	0.16843585	-0.0534085	0.47027866
-0.0832358	0.25999117	-0.1272576	0.08431424	0.2245355	0.00212171
-0.2967617	4.10E-05	-0.2159122	0.00316014	-0.1173051	0.11178915
-0.0704501	0.34063329	-0.0209528	0.77710859	-0.2597302	0.00035666
-0.2679288	0.00022676	-0.3027658	2.80E-05	0.13843474	0.06021552
0.13700398	0.06294161	0.0856582	0.24632871	-0.3365767	2.81E-06
-0.2706423	0.00019457	-0.3750152	1.45E-07	0.09829553	0.18314694
-0.2891716	6.55E-05	-0.3186568	9.84E-06	0.42377225	1.85E-09
-0.0989011	0.18045039	-0.1821962	0.01305916	-0.1844842	0.01194165
-0.0811843	0.27196174	-0.2253641	0.00204043	-0.3427537	1.79E-06
0.15588886	0.0340974	-0.0416963	0.57307232	0.16807928	0.02220022
-0.1972166	0.00712951	-0.2369855	0.00116261	0.30899732	1.87E-05
0.28062203	0.00010927	0.05727624	0.43869616	-0.3104214	1.71E-05
-0.0458846	0.53512965	-0.1190776	0.10644032	-0.0969599	0.18919845
-0.1663275	0.02365109	-0.2373045	0.00114435	0.38968569	4.19E-08
0.16609661	0.02384829	0.29334068	5.07E-05	-0.3526317	8.53E-07
-0.1015454	0.169019	-0.1459668	0.04741906	0.3170753	1.10E-05
0.0471264	0.52412619	0.00946714	0.89823033	0.24697638	0.00070115
0.1681817	0.02211783	0.14573009	0.04778357	-0.2364272	0.00119521
-0.3317971	3.96E-06	-0.3056706	2.33E-05	-0.1227692	0.09594713

-0.0898999	0.2236238	-0.0859445	0.24474733	0.20007052	0.00632438
-0.3543998	7.45E-07	-0.2852816	8.28E-05	0.39717678	2.17E-08
-0.2701158	0.00020046	-0.2300654	0.00163054	-0.2183772	0.00282425
-0.1852713	0.01157708	-0.1728558	0.0186278	0.09915666	0.17932152
0.40272436	1.32E-08	0.20983878	0.00414699	0.31006312	1.75E-05
-0.1935911	0.00828321	-0.1679245	0.02232521	0.164412	0.02533026
-0.154822	0.03535761	-0.1652652	0.02457009	0.17077576	0.02011704
0.0642896	0.38462505	-0.0137845	0.85226614	0.16811408	0.02217219
-0.2518808	0.00054286	-0.1087124	0.14075218	-0.1436319	0.05111775
0.10880281	0.14042023	0.02154397	0.77099217	-0.3741439	1.55E-07
-0.3106893	1.68E-05	-0.2825674	9.74E-05	-0.2070407	0.00468872
-0.1530395	0.03755111	0.06918546	0.34939276	-0.1891648	0.00991424
0.00952154	0.89764875	-0.0642787	0.38470605	-0.2991304	3.53E-05
-0.0884026	0.2314624	-0.1421943	0.05351203	0.24769734	0.00067548
-0.047343	0.52221923	-0.090899	0.21849902	0.2055857	0.00499484
0.12101076	0.10083813	-0.0104033	0.88822975	0.03576833	0.62884073
-0.2918941	5.54E-05	-0.2766874	0.00013754	0.37836764	1.09E-07
-0.088563	0.23061376	-0.1052421	0.1539527	0.25057124	0.00058154
0.05717337	0.43952019	0.01070671	0.8849919	0.3250406	6.35E-06
-0.0557201	0.4512552	-0.1222824	0.09728193	0.37633677	1.30E-07
-0.2393362	0.00103411	-0.1839533	0.01219316	0.17845904	0.01508234
0.04603303	0.53380832	-0.0173601	0.81456559	0.19454525	0.00796455
-0.1745962	0.01745617	-0.0415423	0.57449133	-0.2738036	0.00016246
-0.4227584	2.04E-09	-0.2989824	3.57E-05	0.42130212	2.35E-09
-0.29448	4.73E-05	-0.2390764	0.00104764	0.10118135	0.17055991
0.10325435	0.16192295	0.14076393	0.05598579	-0.3385583	2.43E-06
-0.0162491	0.82623862	-0.0045976	0.95047476	-0.1848486	0.01177162
-0.0781627	0.29026483	-0.1277521	0.08310501	0.24687087	0.00070498
-0.1770924	0.0158878	-0.0946871	0.19982974	-0.2544673	0.00047337
0.14326069	0.05172737	-0.0166386	0.82214139	0.15319831	0.03735116
0.11444122	0.12087313	0.00515994	0.94442644	0.15671182	0.03315148
-0.0995554	0.17757012	-0.1016694	0.1684963	-0.0504613	0.49515595
-0.1016921	0.16840079	-0.098511	0.18218424	0.16465307	0.02511346
0.17378356	0.01799501	0.11705861	0.11254932	-0.1815703	0.01338047
0.15564154	0.03438611	0.01365497	0.85363896	0.15847726	0.03119718
-0.1956222	0.007618	-0.1567272	0.03313401	-0.3748377	1.47E-07
-0.0779327	0.29169076	0.00970399	0.89569858	-0.0776773	0.29328031
0.16568567	0.02420274	0.06936953	0.34810911	0.03263706	0.65919794
-0.077178	0.29640315	-0.13684	0.06326049	0.41717592	3.48E-09
-0.1859293	0.01127993	-0.2299056	0.00164314	0.03291648	0.65646531
0.14033352	0.05674836	0.06014403	0.4160809	-0.2618949	0.00031691
-0.1652718	0.02456429	-0.2483361	0.00065347	0.04388031	0.55312942
-0.1162953	0.11492914	-0.0629778	0.39441912	0.05652169	0.44476085
-0.2352492	0.00126675	-0.0788788	0.28585464	-0.199631	0.00644283
-0.0038502	0.95851795	0.03595069	0.62709117	-0.3588891	5.26E-07
-0.3105884	1.69E-05	-0.0646117	0.38224306	-0.3657122	3.07E-07

0.13529166	0.06633631	0.0576433	0.43576304	0.10778982	0.14417428
-0.1845028	0.01193288	-0.1166155	0.11392619	0.16162659	0.02795403
-0.0725928	0.32611341	0.10101771	0.171256	-0.4131052	5.10E-09
0.08161988	0.26938939	-0.0697715	0.34531641	0.17041234	0.02038755
0.1651425	0.02467819	0.08953487	0.22551717	0.15314939	0.03741268
0.07235405	0.3277111	-0.0268444	0.71681868	0.10909664	0.13934539
-0.0638874	0.3876124	-0.0795007	0.28206075	0.23277879	0.00142968
-0.2445004	0.0007963	-0.21944	0.00268968	0.29125264	5.77E-05
0.52154729	2.70E-14	0.44805789	1.60E-10	-0.2019898	0.00582959
0.04303092	0.56084537	-0.0404115	0.58496054	-0.4102489	6.65E-09
-0.2215179	0.00244322	-0.1657841	0.02411743	0.42971701	1.03E-09
-0.3055202	2.35E-05	-0.2647019	0.00027148	0.3107047	1.67E-05
-0.3218463	7.92E-06	-0.0756819	0.30589323	-0.0917686	0.21410719
-0.3381884	2.50E-06	-0.2433285	0.00084536	0.02169067	0.76947645
0.08484407	0.25086367	0.02027095	0.78418138	-0.2963496	4.21E-05
-0.2773157	0.00013261	-0.2934933	5.02E-05	0.0078802	0.91521937
-0.242487	0.00088228	-0.2509102	0.00057129	0.19330775	0.00838001
-0.21383	0.00347157	-0.0527281	0.47596016	0.19418295	0.00808424
-0.2561737	0.00043213	-0.1743071	0.01764624	0.20345341	0.00547584
-0.2959704	4.31E-05	-0.3453074	1.48E-06	0.30734267	2.09E-05
-0.0190327	0.79706982	-0.1737033	0.018049	0.13548272	0.06595026
-0.496506	6.60E-13	-0.4074708	8.59E-09	-0.4565258	6.53E-11
-0.0085982	0.90752729	-0.0917028	0.21443719	-0.2992737	3.50E-05
-0.1861286	0.01119125	-0.2017383	0.00589239	0.42342989	1.91E-09
-0.0813801	0.27080365	-0.0998625	0.17623003	0.30020428	3.30E-05
-0.055814	0.45049133	-0.1465677	0.0465041	0.39798591	2.02E-08
0.07111916	0.33605586	-0.0320381	0.66507094	0.21168555	0.003821
-0.1494115	0.04237169	-0.0220158	0.76612001	-0.3365221	2.82E-06
-0.4412331	3.25E-10	-0.2592687	0.00036571	-0.3317426	3.97E-06
0.2404884	0.00097601	0.01857431	0.80185494	0.28250844	9.77E-05
-0.378378	1.09E-07	-0.4158864	3.93E-09	0.20742469	0.00461081
-0.1468235	0.04611923	-0.1348073	0.0673233	-0.2009515	0.0060928
0.00586638	0.93683282	-0.0070407	0.92422264	-0.3565984	6.29E-07
-0.2496626	0.00060985	-0.0964979	0.19132524	-0.3756856	1.37E-07
-0.030553	0.67972009	0.05224799	0.479992	-0.2427704	0.00086969
0.06587823	0.37296357	0.07902746	0.28494463	-0.3507385	9.85E-07
0.20744736	0.00460624	0.05274867	0.47578806	-0.0937816	0.20418358
-0.1675632	0.02261943	-0.0802382	0.27760668	-0.325183	6.29E-06
0.0187119	0.8004178	-0.044344	0.54893897	0.14459574	0.04956293
-0.2886124	6.77E-05	-0.0843596	0.25358951	-0.162046	0.02754469
-0.2616219	0.00032168	-0.0802362	0.27761843	-0.0965817	0.19093829
-0.1922117	0.0087639	-0.1338605	0.06928699	0.1453519	0.04837079
-0.1676306	0.02256433	-0.1185322	0.10806436	-0.0691244	0.34981947
0.36183432	4.18E-07	0.21212685	0.0037466	-0.3072334	2.10E-05
0.0116759	0.87466289	-0.1101188	0.13565527	0.17359292	0.01812349
-0.01849	0.80273545	-0.0861136	0.24381671	0.21671086	0.00304755

0.16612877	0.02382074	0.08386547	0.25639084	-0.2390058	0.00105135
-0.0894516	0.22595056	-0.1147174	0.119973	-0.2815768	0.00010328
-0.3371286	2.70E-06	-0.2693084	0.00020982	0.32670916	5.66E-06
-0.2324515	0.00145265	-0.2956389	4.40E-05	0.23353671	0.00137776
0.07221934	0.32861486	-0.0465155	0.52952502	0.23351354	0.00137932
-0.178425	0.01510199	-0.2383828	0.00108457	0.06338548	0.3913594
-0.2594434	0.00036226	-0.1826603	0.01282537	-0.0139285	0.85074075
-0.2119768	0.00377175	-0.0892848	0.22682059	-0.1524595	0.03828921
0.1224133	0.09692158	0.12409537	0.09238462	-0.1940299	0.00813529
-0.0209431	0.77720979	-0.0982556	0.18332553	0.21091365	0.00395434
0.06202607	0.40161768	-0.0474789	0.52102367	0.23422404	0.00133218
0.04575976	0.5362421	0.23265378	0.00143841	-0.2809665	0.00010707
0.00538032	0.94205696	-0.1681939	0.02210802	0.2125994	0.00366839
0.13231572	0.07259116	0.00598874	0.93551809	0.29886969	3.59E-05
-0.5568307	1.86E-16	-0.2583427	0.00038452	0.15411261	0.03621724
0.28494231	8.45E-05	0.25053787	0.00058255	0.41384735	4.76E-09
-0.0620824	0.40118983	-0.0459552	0.53450081	0.24433795	0.00080294
-0.0885516	0.23067377	-0.100708	0.17257912	0.28905855	6.59E-05
0.22110161	0.00249089	0.0935895	0.205116	0.16924579	0.02127723
-0.0641689	0.38552007	-0.1147465	0.11987849	0.2784981	0.00012377
-0.3252106	6.28E-06	-0.3512153	9.50E-07	0.39292081	3.16E-08
-0.1610548	0.02852048	-0.1665209	0.02348701	0.20049798	0.00621105
0.01588881	0.83003243	0.02454798	0.74013421	0.40079415	1.57E-08
0.04723764	0.52314622	-0.0689468	0.35106173	0.22272369	0.00230979
0.00830662	0.91065022	-0.039291	0.59541972	0.09079495	0.21902872
0.0983353	0.18296896	0.11427917	0.12140377	0.28130253	0.00010497
0.21142129	0.00386619	0.05842908	0.42952187	0.31410192	1.34E-05
0.01058228	0.88631939	-0.0900888	0.22264848	0.11149749	0.13079707
0.14728063	0.0454378	0.11763582	0.11077545	-0.2040497	0.00533731
-0.1375969	0.06179979	-0.2098241	0.00414968	0.20658891	0.00478192
-0.0247771	0.73779711	-0.0587011	0.42737366	0.23293706	0.00141869
-0.0760751	0.30337967	-0.1076993	0.1445134	0.27082694	0.00019254
-0.2643407	0.00027696	-0.1082044	0.14262891	0.09934046	0.17851271
0.00157175	0.98305962	0.02265141	0.75957143	-0.2959646	4.31E-05
0.04392354	0.55273816	-0.0700801	0.34318153	0.10001287	0.17557671
-0.0725388	0.32647438	-0.003523	0.96204025	-0.1134792	0.12404994
0.10566186	0.15230816	-0.0202367	0.78453677	0.12102842	0.10078807
-0.3335909	3.48E-06	-0.225308	0.00204584	0.20953079	0.00420373
-0.2137343	0.00348652	-0.1244664	0.091407	-0.3991903	1.82E-08
-0.1091334	0.13921129	-0.2435082	0.00083766	-0.2683084	0.00022198
-0.0834674	0.25866325	-0.1278321	0.08291081	0.19742121	0.00706889
-0.0725567	0.32635454	-0.0709831	0.33698352	0.16906322	0.02141946
-0.2606339	0.00033953	-0.2040766	0.00533114	-0.0793248	0.28313027
-0.1911641	0.00914532	-0.3153719	1.23E-05	0.00450347	0.95148746
0.1736861	0.01806059	0.03585194	0.62803836	-0.2303794	0.00160604
0.14981166	0.04181579	-0.0062448	0.93276718	0.22735	0.00185703

-0.2131674	0.00357633	-0.1532029	0.03734546	-0.2496896	0.00060898
-0.0134657	0.85564515	-0.0686979	0.35280718	0.24040856	0.00097993
0.01998119	0.78719219	-0.1299869	0.07781301	0.26619404	0.00024987
-0.2697881	0.00020421	-0.1353956	0.066126	-0.1981714	0.00685054
0.35320779	8.16E-07	0.18333726	0.01249087	-0.2338241	0.00135853
-0.3263721	5.79E-06	-0.2816429	0.00010288	-0.2184477	0.00281514
-0.3507006	9.88E-07	-0.1408195	0.05588791	-0.0502229	0.49719849
-0.2801623	0.00011226	-0.2053058	0.00505574	0.30385702	2.61E-05
-0.1125815	0.12707219	-0.1558986	0.0340861	0.14954102	0.04219113
0.17642908	0.01629209	0.16647226	0.02352819	0.30221558	2.90E-05
0.01328791	0.85753095	-0.0671692	0.36364876	0.2287333	0.0017383
-0.058201	0.43132794	-0.1180705	0.10945401	0.24809584	0.00066167
0.13416287	0.06865489	0.16196056	0.02762766	-0.2963566	4.20E-05
-0.0956729	0.19516648	-0.0768287	0.29860125	0.36874816	2.41E-07
0.04493565	0.54361514	-0.2020869	0.0058055	-0.1708621	0.02005324
0.04019342	0.58698946	0.00603932	0.93497466	-0.2151325	0.00327367
0.03143578	0.67099721	0.07694316	0.29787974	0.38784035	4.91E-08
0.37976478	9.74E-08	0.12742844	0.08389497	-0.3063159	2.23E-05
-0.2852539	8.30E-05	-0.2315712	0.0015161	0.31350579	1.39E-05
-0.226113	0.00196942	-0.1230671	0.09513754	-0.2630606	0.00029725
-0.2177135	0.00291135	-0.2765562	0.00013859	0.27375131	0.00016294
-0.003293	0.96451696	0.0059343	0.93610297	-0.2461618	0.00073123
-0.0315715	0.66965961	-0.1626018	0.02701016	0.3787916	1.06E-07
-0.1975412	0.00703355	-0.2281166	0.00179035	-0.42224	2.14E-09
0.08463966	0.25201132	-0.0399095	0.58963593	0.20413361	0.00531808
0.03889955	0.59909384	0.08931479	0.22666422	0.05205989	0.48157657
-0.1252138	0.08946277	-0.0576309	0.43586204	-0.3869824	5.29E-08
-0.002615	0.97181885	0.05049791	0.49484307	-0.1045836	0.15655958
0.04975237	0.50124285	-0.0115842	0.87563888	-0.0384478	0.60334675
0.14344592	0.05142247	0.01633809	0.82530217	-0.3245032	6.59E-06
-0.1027737	0.16389575	-0.1587655	0.03088764	-0.0599149	0.41786207
-0.1174887	0.11122543	-0.0232638	0.75327832	-0.0213658	0.77283436
0.09811981	0.18393485	0.05509818	0.45633038	-0.1023689	0.16557108
0.41009945	6.74E-09	0.24042865	0.00097895	-0.0990661	0.17972082
-0.2118829	0.00378757	-0.1434365	0.0514379	-0.1078772	0.14384752
-0.1892501	0.00988032	-0.1110409	0.13239103	0.07074663	0.33859966
0.00284665	0.96932393	-0.1664849	0.02351748	0.08225267	0.26568196
-0.1736634	0.01807591	-0.1126226	0.12693242	-0.2099189	0.00413236
-0.2233041	0.00224795	-0.1396921	0.0579007	-0.2400673	0.00099689
0.03907349	0.59746014	0.01297744	0.86082566	0.14082077	0.05588572
0.25989138	0.00035355	0.16054525	0.0290335	-0.3229597	7.34E-06
-0.1766074	0.01618251	-0.1283449	0.08167379	-0.10837	0.14201493
-0.2539371	0.00048691	-0.1078012	0.14413157	-0.1160776	0.11561501
-0.1447311	0.0493477	-0.2093541	0.00423659	0.07793138	0.29169914
-0.1776344	0.01556396	-0.2065232	0.00479561	0.19627687	0.00741393
-0.0290438	0.69473042	-0.090265	0.2217413	0.31526249	1.24E-05

-0.1999738	0.00635028	-0.2111037	0.00392113	-0.253414	0.00050061
-0.23825	0.00109177	-0.0725347	0.32650147	-0.4231063	1.97E-09
-0.1562623	0.03366538	-0.0157054	0.83196521	0.38895851	4.46E-08
-0.3305903	4.31E-06	-0.2337692	0.00136219	0.16613791	0.02381292
-0.2061545	0.00487311	-0.1670623	0.02303286	0.22819551	0.00178361
-0.4047079	1.11E-08	-0.2818752	0.00010147	0.33688633	2.75E-06
-0.2881832	6.95E-05	-0.3400005	2.19E-06	0.11723881	0.11199318
-0.2884108	6.86E-05	-0.2081854	0.00445985	-0.2549484	0.00046139
-0.2535856	0.00049608	-0.159926	0.02966754	-0.1180785	0.10942997
-0.0775884	0.29383447	-0.1775676	0.01560357	0.27316857	0.00016848
0.08513701	0.24922527	0.18779916	0.01047175	0.12346592	0.09406217
-0.0318649	0.66677253	-0.0239589	0.74615483	0.12576613	0.08804725
-0.1704173	0.02038387	-0.2152461	0.0032569	-0.2722636	0.00017743
0.07822466	0.28988147	0.13697512	0.06299761	-0.2803568	0.00011099
0.23462362	0.00130632	0.19660308	0.00731408	-0.2950141	4.57E-05
0.04145889	0.57526061	0.0630022	0.39423564	-0.0598955	0.41801306
-0.0486082	0.5111483	-0.0678492	0.35880104	-0.1003761	0.17400568
-0.1173159	0.11175591	-0.0152652	0.8366087	-0.2511062	0.00056544
-0.0915849	0.21502972	-0.0236854	0.74895534	-0.3208711	8.47E-06
-0.084744	0.25142498	-0.002063	0.9777658	-0.3280487	5.15E-06
-0.1363215	0.06427711	-0.0640901	0.38610523	-0.2126306	0.00366327
0.07235665	0.32769365	0.05496061	0.45745736	-0.2322709	0.00146546
0.07474412	0.31194245	0.10767387	0.1446088	-0.0373099	0.61411666
0.10468673	0.15614926	0.10444893	0.15709691	0.06528855	0.37726658
0.41820255	3.15E-09	0.30948921	1.81E-05	0.02678548	0.71741362
-0.1740796	0.01779709	-0.2034643	0.00547329	0.44982172	1.33E-10
-0.1744141	0.01757565	-0.2427984	0.00086845	0.46960608	1.55E-11
-0.1448832	0.04910683	-0.152112	0.03873716	-0.4503357	1.26E-10
0.31269204	1.47E-05	0.13379858	0.06941712	-0.048482	0.51224744
-0.4490193	1.45E-10	-0.2612003	0.00032919	0.1889914	0.00998354
0.07565532	0.3060636	-0.0045165	0.95134731	0.25604953	0.00043502
0.00519855	0.94401132	-0.1544731	0.03577827	0.37658241	1.27E-07
0.18177896	0.01327258	0.09219361	0.21198365	0.34996758	1.04E-06
-0.0870405	0.23875989	-0.2666751	0.00024325	0.29454638	4.71E-05
-0.0292645	0.69252764	-0.1008403	0.17201322	0.19989795	0.00637065
0.05441222	0.4619649	-0.0817882	0.26839992	0.29987419	3.37E-05
-0.3367059	2.78E-06	-0.2402677	0.0009869	-0.3423918	1.84E-06
-0.1058934	0.15140676	-0.138498	0.06009721	0.21197587	0.00377191
-0.5600953	1.14E-16	-0.445461	2.10E-10	0.20516294	0.00508708
-0.2593493	0.00036411	-0.3274451	5.37E-06	0.22415867	0.00215964
0.14168883	0.05437572	0.01260782	0.86475113	0.30288189	2.78E-05
0.07306732	0.32295244	-0.0275277	0.70993015	0.22333358	0.00224485
-0.1470753	0.04574275	-0.1970955	0.00716561	0.27136162	0.00018678
0.12925676	0.07951117	0.02339082	0.75197489	0.24860367	0.00064444
0.3638867	3.55E-07	0.25560394	0.00044552	0.22818647	0.00178438
-0.198828	0.00666438	-0.0513705	0.48740799	0.57083691	2.19E-17

-0.2865368	7.68E-05	-0.2918507	5.56E-05	0.38183647	8.19E-08
-0.0296788	0.68839994	-0.1268407	0.08534481	0.09935662	0.17844174
-0.0745004	0.31352713	-0.0729194	0.32393577	0.00464058	0.95001233
-0.1028462	0.16359698	-0.1371415	0.06267535	-0.3004617	3.25E-05
0.15427359	0.03602065	0.05251321	0.47776255	-0.2286585	0.00174453
-0.376388	1.29E-07	-0.3775628	1.17E-07	0.27293216	0.00017078
-0.0620696	0.40128684	-0.1675838	0.02260264	0.04978648	0.50094914
0.21598478	0.00314976	0.00990782	0.89352056	0.1206246	0.10193817
-0.1524094	0.03835356	-0.0841028	0.25504285	-0.344159	1.61E-06
0.2122658	0.00372345	0.1528005	0.0378538	0.25814267	0.0003887
0.027749	0.70770414	-0.0147912	0.84161374	0.26663776	0.00024376
0.23753999	0.00113105	0.08408578	0.25513928	-0.2417158	0.00091742
0.08006417	0.27865328	0.1683989	0.02194396	-0.1603966	0.02918463
0.09450966	0.2006775	-0.0709844	0.33697484	0.09818088	0.18366071
0.1234712	0.09404801	-0.0488166	0.50933718	0.16959503	0.02100743
-0.0166521	0.82200021	-0.0100439	0.89206728	-0.1408993	0.05574762
-0.0061424	0.93386775	-0.0231162	0.75479323	0.34418083	1.61E-06
-0.1474626	0.04516885	0.29604877	4.29E-05	-0.2809094	0.00010743
0.29065505	5.98E-05	0.36286569	3.85E-07	-0.4421248	2.97E-10
0.06377934	0.38841706	-0.0268865	0.71639359	0.23989562	0.00100551
-0.1324752	0.07224421	-0.2423634	0.00088783	0.2598233	0.00035486
-0.3791362	1.03E-07	-0.371286	1.96E-07	0.46163745	3.75E-11
0.04303652	0.56079434	-0.0357417	0.62909678	0.21395441	0.00345221
0.05546798	0.4533088	-0.0219192	0.76711737	0.22506252	0.00206968
0.10808489	0.143073	0.36053779	4.63E-07	-0.1325181	0.07215107
0.31186823	1.55E-05	0.02708414	0.71439908	-0.3033856	2.69E-05
-0.205451	0.00502407	-0.1469928	0.04586584	-0.3073022	2.09E-05
0.04947935	0.50359734	-0.0703877	0.34106201	0.18276321	0.012774
0.61855881	6.47E-21	0.25126773	0.00056066	-0.3331793	3.58E-06
0.03455521	0.64053165	0.00108807	0.98827232	-0.005481	0.94097486
-0.0377031	0.6103856	-0.0531755	0.47221982	0.3808646	8.89E-08
-0.437384	4.81E-10	-0.3108172	1.66E-05	0.03540469	0.63233568
-0.4224478	2.10E-09	-0.2429761	0.00086065	-0.2797982	0.00011469
-0.2060997	0.0048847	-0.1881628	0.01032064	-0.3022227	2.90E-05
-0.1958231	0.00755485	-0.1326283	0.07191242	-0.2518789	0.00054292
-0.2293194	0.00169011	-0.2339238	0.00135192	0.0810248	0.27290792
-0.0370092	0.61697737	-0.0162624	0.82609816	-0.2882519	6.92E-05
0.05992802	0.41776025	0.04840429	0.51292443	-0.4624203	3.44E-11
0.39379234	2.93E-08	0.2611815	0.00032953	-0.2817	0.00010253
-0.2760243	0.00014293	-0.1594651	0.03014714	-0.0845869	0.25230798
-0.0805018	0.27602611	-0.1142365	0.12154367	-0.4036824	1.21E-08
-0.3214395	8.14E-06	-0.3323526	3.80E-06	0.40082491	1.57E-08
0.42052968	2.53E-09	0.35091517	9.72E-07	-0.2101722	0.00408636
0.18185791	0.01323197	0.05054881	0.49440778	-0.4026427	1.33E-08
0.01200426	0.87116826	-0.0764513	0.30098842	0.23540566	0.00125703
0.47150047	1.25E-11	0.24561146	0.00075222	-0.2648552	0.00026918

-0.3771082	1.22E-07	-0.2723328	0.00017673	0.17900044	0.01477326
0.01556445	0.83345131	-0.0288476	0.69669082	0.26851211	0.00021945
0.2248251	0.00209297	0.1907706	0.00929236	-0.3372816	2.67E-06
-0.1917585	0.00892715	-0.174612	0.01744584	0.14539003	0.04831133
-0.2204479	0.00256745	-0.3137167	1.37E-05	0.14750991	0.04509924
-0.1108352	0.13311394	-0.0996771	0.17703829	-0.1847414	0.01182139
0.11578556	0.11654008	-0.047518	0.52068049	-0.2088623	0.0043293
-0.1376728	0.06165492	-0.0081429	0.91240457	-0.3001649	3.31E-05
-0.1493017	0.04252532	-0.1016611	0.16853128	-0.2884285	6.85E-05
-0.0756998	0.30577862	-0.0573303	0.43826352	-0.2513478	0.00055831
0.05477641	0.45896867	-0.1121477	0.12855298	0.07302024	0.32326515
0.15909413	0.03053785	0.00880159	0.9053502	-0.2550101	0.00045988
-0.2753265	0.00014882	-0.144599	0.04955777	-0.3103818	1.71E-05
0.17223933	0.01905885	0.135989	0.06493618	0.07396222	0.31704558
-0.1521664	0.03866684	-0.1626852	0.02693068	0.09830633	0.18309856
-0.2381207	0.00109883	-0.255081	0.00045814	0.14287018	0.05237512
-0.2536821	0.00049355	-0.1125355	0.12722846	-0.2467489	0.00070943
-0.0724379	0.32714953	-0.0667856	0.3664012	-0.1678033	0.02242358
-0.1640432	0.02566507	-0.2655789	0.00025858	0.13198273	0.07331989
-0.1402422	0.05691118	-0.2628951	0.00029997	0.28900508	6.61E-05
-0.2256511	0.00201294	-0.0812172	0.27176671	0.21418963	0.00341587
-0.0876204	0.23563377	-0.143082	0.05202289	0.10465911	0.15625908
0.02794371	0.70574772	-0.24262	0.00087635	0.15714479	0.03266282
-0.3269255	5.57E-06	-0.1747204	0.01737508	-0.0600825	0.41655868
0.04415902	0.55060892	0.02083987	0.77827923	-0.2840884	8.90E-05
-0.1072806	0.1460901	-0.103667	0.16024334	0.03489915	0.63720793
-0.0468572	0.52650134	-0.0955814	0.19559611	-0.1973554	0.00708835
-0.1165477	0.11413781	-0.1489358	0.04304073	0.16380015	0.02588774
-0.0373156	0.61406321	-0.2683231	0.0002218	0.23702193	0.00116051
-0.0156064	0.83300922	-0.0302497	0.68272647	0.31127416	1.61E-05
0.12982606	0.07818447	0.00257496	0.97225045	0.35803944	5.62E-07
-0.09296	0.20819275	-0.1344552	0.06804812	0.28902036	6.61E-05
-0.0275248	0.70995898	-0.0252839	0.7326352	0.33915489	2.33E-06
-0.3291745	4.76E-06	-0.4356221	5.75E-10	0.0485666	0.51151055
-0.0188562	0.79891138	0.35988992	4.87E-07	-0.4101752	6.70E-09
-0.0881356	0.23288038	-0.1691318	0.02136598	0.04140556	0.5757527
-0.196311	0.00740343	-0.0083929	0.90972564	-0.2237037	0.00220626
0.62224849	3.26E-21	0.35217198	8.83E-07	-0.2582716	0.000386
-0.0488329	0.50919562	-0.0783854	0.28888827	-0.4687973	1.70E-11
-0.3648176	3.30E-07	-0.2536272	0.00049498	0.36176218	4.20E-07
-0.328432	5.02E-06	-0.342604	1.81E-06	0.19428208	0.00805134
-0.2159298	0.00315763	-0.1375981	0.06179764	-0.1623192	0.02728078
-0.0494627	0.50374103	-0.1089803	0.13977005	0.20096665	0.00608887
0.44642725	1.90E-10	0.18276829	0.01277147	-0.3363789	2.85E-06
-0.3469102	1.31E-06	-0.3173876	1.07E-05	0.09818027	0.18366346
-0.2009174	0.00610162	-0.2886571	6.76E-05	0.30201933	2.94E-05

-0.0161369	0.82741985	-0.1383696	0.06033751	0.36019544	4.75E-07
-0.1773412	0.01573846	-0.1195942	0.10491982	0.13283727	0.07146162
-0.1745294	0.01749991	-0.132499	0.07219254	0.1827035	0.01280377
-0.2908804	5.90E-05	-0.1989062	0.00664248	0.0542391	0.46339295
-0.1198126	0.10428203	-0.1878247	0.01046109	0.26710176	0.00023752
-0.0728033	0.32470871	-0.1669296	0.02314346	0.32752781	5.34E-06
-0.321613	8.05E-06	-0.3144189	1.31E-05	0.12340099	0.09423659
0.14476163	0.04929933	-0.1151043	0.11872053	-0.2158445	0.00316986
-0.0738286	0.31792297	-0.0816229	0.2693716	0.16266993	0.02694522
-0.1070939	0.14679713	-0.0776764	0.29328573	0.22185298	0.00240546
-0.1049226	0.15521366	-0.0341893	0.64407547	-0.4059885	9.84E-09
0.11903866	0.10655553	0.06829068	0.35567543	0.12173097	0.09881161
-0.2083847	0.00442105	-0.2025372	0.00569496	-0.2160393	0.00314198
0.0102726	0.88962467	-0.0529444	0.47414982	0.19622692	0.00742932
-0.1068294	0.14780353	-0.1213928	0.09975908	0.44532838	2.13E-10
0.0056009	0.93968585	0.02266202	0.75946235	0.00637946	0.93132113
-0.160438	0.02914246	-0.252744	0.00051869	0.15010115	0.0414174
0.05024978	0.49696828	-0.1201181	0.10339528	0.34294575	1.76E-06
0.29077566	5.94E-05	0.12923979	0.07955098	-0.0013511	0.98543792
0.14287875	0.05236084	0.07382153	0.31796952	-0.3327485	3.70E-06
0.09243184	0.21080006	-0.0858444	0.24529975	0.17042675	0.02037677
-0.2595791	0.0003596	-0.2667438	0.00024232	0.10679014	0.14795334
-0.0473578	0.5220888	-0.0685919	0.35355232	0.22571688	0.00200669
-0.281322	0.00010485	-0.0215121	0.77132149	-0.2186813	0.00278513
-0.2770275	0.00013485	-0.1466586	0.04636708	-0.2688438	0.00021539
-0.1710744	0.01989709	-0.1695152	0.02106886	0.02418877	0.7438035
0.22511697	0.00206437	0.10327658	0.16183215	0.14891849	0.04306524
-0.2977296	3.86E-05	-0.1785537	0.01502791	0.11809111	0.10939177
-0.2395744	0.00102184	-0.1978942	0.0069305	-0.1479457	0.04446161
0.20306924	0.00556678	0.10563454	0.15241481	-0.3385402	2.44E-06
-0.0366921	0.6199995	-0.1683202	0.02200685	0.20619059	0.00486546
-0.2308529	0.00156974	0.02111624	0.77541656	-0.3032279	2.72E-05
-0.1000538	0.17539925	-0.1660861	0.02385732	0.201267	0.00601171
0.02727675	0.71245724	-0.0803121	0.27716268	0.08702945	0.2388198
-0.0583491	0.43015442	-0.1156499	0.1169717	-0.1635699	0.02610027
0.3171909	1.09E-05	0.21339413	0.00354016	-0.2769171	0.00013572
0.28270549	9.66E-05	0.21155999	0.00384241	-0.2194186	0.00269233
0.05737846	0.43787819	-0.0663663	0.36942476	0.09916191	0.1792984
0.06281712	0.39562903	-0.0703648	0.34121963	0.195029	0.00780718
-0.1259791	0.08750615	-0.261364	0.00032626	0.07128291	0.33494157
0.28655788	7.67E-05	0.04758366	0.52010371	0.20141963	0.00597284
-0.1120906	0.12874872	-0.2143218	0.00339562	0.26737814	0.00023387
-0.332436	3.78E-06	-0.1385169	0.06006195	-0.2626429	0.00030416
0.1536772	0.03675357	-0.0447491	0.54529099	0.11071402	0.13354111
-0.2304844	0.00159793	-0.2483802	0.00065197	0.14116624	0.05528067
0.25009536	0.00059621	0.1131372	0.12519466	0.24902256	0.00063054

-0.1110884	0.13222445	-0.1688666	0.02157353	0.21654961	0.00306999
-0.3011702	3.10E-05	-0.2491244	0.00062721	0.46379725	2.96E-11
-0.1349741	0.06698194	-0.169321	0.02121887	0.38328585	7.24E-08
-0.1468186	0.04612653	-0.1919109	0.00887197	0.26473265	0.00027102
-0.0949911	0.1983829	-0.095897	0.19411748	0.21197812	0.00377153
-0.211114	0.00391933	-0.2189802	0.00274717	-0.3108768	1.66E-05
-0.203518	0.00546067	-0.2928881	5.21E-05	0.36088707	4.50E-07
0.56224867	8.24E-17	0.40958808	7.07E-09	-0.1962423	0.00742457
-0.1815177	0.01340777	-0.3292941	4.72E-06	0.18951096	0.00977716
-0.1640636	0.02564646	-0.2634479	0.00029097	0.31019048	1.73E-05
0.2401767	0.00099142	-0.0314881	0.67048198	-0.1025441	0.16484437
0.28123272	0.0001054	0.42061106	2.51E-09	-0.3739114	1.58E-07
0.27789776	0.00012819	0.19543791	0.00767632	-0.3841795	6.72E-08
-0.1381635	0.06072474	-0.134477	0.06800308	0.21473219	0.00333338
0.03015583	0.68365819	-0.0239005	0.74675275	0.34667818	1.34E-06
0.01183435	0.87297622	-0.078873	0.28588983	0.41847486	3.07E-09
-0.2732616	0.00016758	-0.0716185	0.33266534	-0.1542555	0.03604271
-0.1997725	0.00640448	-0.2077528	0.00454516	-0.2869556	7.49E-05
0.03028687	0.68235782	0.00231816	0.97501699	-0.4013235	1.50E-08
0.28421412	8.83E-05	0.22832311	0.00177276	0.13624751	0.06442334
0.32640043	5.78E-06	0.11383842	0.1228562	-0.0891656	0.2274443
0.49850133	5.16E-13	0.22209787	0.0023782	-0.3178816	1.04E-05
0.34961222	1.07E-06	0.1548179	0.03536255	0.31118118	1.62E-05
-0.068191	0.35637983	-0.1822256	0.01304428	0.19645169	0.00736027
-0.3527285	8.47E-07	-0.412209	5.54E-09	0.18510303	0.0116542
-0.0324029	0.66149136	-0.174366	0.01760734	0.09496358	0.1985137
-0.1651512	0.02467053	-0.265348	0.00026192	0.09695418	0.18922457
-0.374164	1.55E-07	-0.1811477	0.01360124	-0.3725932	1.76E-07
0.02607492	0.72460324	-0.0705951	0.33963819	-0.0902372	0.22188394
-0.1194507	0.1053403	-0.1486527	0.04344305	0.24823213	0.000657
-0.0015093	0.98373214	0.01278872	0.86282951	-0.1865756	0.01099459
0.06317372	0.39294692	-0.0402363	0.5865902	0.10861509	0.14111023
-0.291218	5.78E-05	-0.2974517	3.93E-05	0.09881957	0.18081191
-0.1119197	0.12933652	-0.2553667	0.0004512	0.07736846	0.29520928
-0.1069541	0.14732825	-0.1444833	0.04974228	0.31583574	1.19E-05
-0.1084226	0.14182051	-0.2320115	0.00148405	0.29563505	4.40E-05
-0.1767045	0.01612315	-0.0352906	0.63343376	-0.0944631	0.20090035
-0.1506919	0.04061432	-0.0336663	0.64915463	-0.1227453	0.09601242
-0.2093711	0.00423342	-0.2394732	0.00102704	0.14914342	0.04274768
-0.1290378	0.08002632	-0.1410253	0.05552678	-0.360476	4.65E-07
-0.1454484	0.0482204	-0.1048609	0.15545804	0.17191867	0.01928648
0.34770063	1.24E-06	0.23958303	0.0010214	0.17718288	0.01583334
0.04290879	0.56195904	-0.0903252	0.22143198	0.22223044	0.00236356
0.32308791	7.27E-06	0.261724	0.00031989	-0.1692877	0.02124471
-0.1857926	0.01134111	-0.2677565	0.00022896	0.14904582	0.04288523
-0.1301005	0.07755133	-0.1740896	0.01779041	0.20711815	0.00467292

0.33971565	2.24E-06	0.30198438	2.95E-05	-0.4278377	1.24E-09
-0.2238287	0.00219336	-0.2359081	0.00122626	0.23124731	0.00154008
-0.0927654	0.20915107	0.01009745	0.89149502	-0.2115149	0.00385013
-0.1302444	0.0772211	-0.1487634	0.04328532	-0.1675174	0.022657
-0.0779318	0.29169631	-0.1034987	0.16092686	-0.1958352	0.00755106
-0.3330806	3.61E-06	-0.3117234	1.57E-05	-0.1127282	0.12657425
-0.1259653	0.08754129	-0.1963366	0.00739554	0.09590013	0.19410294
-0.0213099	0.77341276	0.04136814	0.57609805	0.16803772	0.02223374
0.14979004	0.04184566	0.02558353	0.72958944	0.3748337	1.47E-07
-0.1593177	0.03030184	-0.1652318	0.02459944	-0.2156269	0.00320127
-0.2056317	0.00498489	-0.2455674	0.00075392	0.03316294	0.65405881
-0.2697332	0.00020485	-0.1772817	0.01577402	-0.0038363	0.95866748
-0.3452361	1.49E-06	-0.3374444	2.64E-06	0.13038511	0.07689914
-0.0561319	0.44791258	-0.1190895	0.106405	0.15864265	0.03101923
0.12124481	0.10017602	0.01530469	0.83619146	0.23630104	0.00120269
0.0799905	0.27909724	-0.1934701	0.00832443	0.2355833	0.00124607
-0.1974592	0.00705769	-0.1203744	0.10265589	-0.2331846	0.00140166
-0.289479	6.43E-05	-0.1570606	0.03275731	-0.2583258	0.00038487
0.16111713	0.02845825	0.11014179	0.1355731	-0.0325076	0.66046601
-0.1370157	0.06291882	-0.2264175	0.00194119	0.00917634	0.90134033
-0.1959136	0.00752654	-0.2297966	0.00165178	0.15290539	0.03772072
-0.2252466	0.00205178	-0.2527274	0.00051914	0.26107669	0.00033142
-0.0685056	0.35415975	-0.02516	0.73389607	0.07923987	0.28364789
-0.103118	0.16248078	-0.1501527	0.04134679	0.09739905	0.18719274
-0.0155938	0.83314129	-0.0296444	0.68874224	0.32880494	4.89E-06
0.12848923	0.08132844	0.01897845	0.79763553	0.21793689	0.00288177
-0.0547737	0.45899088	-0.0912802	0.21656604	0.22924447	0.0016962
-0.275365	0.00014849	-0.1557238	0.03428981	-0.3269837	5.55E-06
-0.3117454	1.56E-05	-0.1874154	0.01063332	-0.4808224	4.30E-12
-0.1303661	0.07694258	-0.214962	0.00329898	0.17588865	0.01662808
-0.0645914	0.38239244	-0.2094269	0.00422303	-0.3354304	3.05E-06
0.02688712	0.71638724	-0.0579523	0.4333023	0.22193928	0.00239582
-0.035681	0.62967899	-0.0486609	0.51069	0.4073547	8.68E-09
0.01349557	0.85532865	-0.0046499	0.9499122	0.18811936	0.01033861
-0.2936326	4.98E-05	-0.2192337	0.00271534	-0.2201241	0.00260616
-0.2640505	0.00028145	-0.1364308	0.06406166	-0.3405681	2.10E-06
-0.186601	0.01098349	-0.0128698	0.86196896	0.0979527	0.18468646
-0.1305666	0.07648547	-0.0877807	0.23477438	-0.3065076	2.20E-05
-0.2469079	0.00070363	-0.2163997	0.00309098	-0.1660766	0.02386548
-0.100958	0.17151034	-0.1207213	0.10166194	0.02845704	0.70059893
-0.2265871	0.00192563	-0.1050822	0.15458295	-0.2556279	0.00044495
0.14302436	0.05211859	0.01014264	0.89101235	0.31188728	1.55E-05
0.60395572	8.99E-20	0.33573637	2.98E-06	-0.1014972	0.16922244
-0.1643805	0.02535873	-0.2998355	3.38E-05	0.34165765	1.94E-06
-0.2309053	0.00156577	-0.086869	0.23969027	0.35496778	7.13E-07
-0.087388	0.23688288	0.03261448	0.65941899	-0.0958447	0.1943619

0.37723831	1.20E-07	0.20407603	0.00533127	0.11822729	0.10898057
0.158326	0.03136069	0.07936787	0.28286838	-0.3408688	2.06E-06
0.1203993	0.1025843	0.02998299	0.68537481	0.4044263	1.13E-08
0.26328934	0.00029352	0.13343339	0.07018811	0.25965798	0.00035806
-0.0081193	0.91265702	-0.0485152	0.511958	0.17723067	0.01580464
0.00994607	0.89311189	-0.0220729	0.76553077	-0.1151274	0.11864618
0.59865798	2.26E-19	0.35900988	5.21E-07	-0.1538966	0.03648249
0.18647947	0.01103661	-0.0669887	0.36494279	0.26391694	0.00028353
-0.1068421	0.14775496	-0.0066458	0.92846088	0.12534403	0.08912735
-0.4119705	5.67E-09	-0.4034456	1.24E-08	0.40846348	7.84E-09
-0.0134805	0.85548844	0.00208172	0.97756437	-0.2940826	4.84E-05
-0.0362291	0.62442394	-0.0031978	0.96554197	0.06564928	0.37463064
0.01167284	0.87469548	-0.0951335	0.19770793	-0.1226374	0.09630705
0.27202893	0.00017982	0.08670315	0.24059194	0.29734537	3.95E-05
-0.1297704	0.07831341	-0.1195416	0.10507393	0.24747045	0.00068346
-0.0919581	0.21315863	-0.1084437	0.14174244	0.30275167	2.81E-05
0.27037818	0.00019751	0.16816167	0.02213392	-0.2677283	0.00022933
0.02842908	0.70087907	-0.0382507	0.60520596	0.17301274	0.01851941
-0.1474533	0.04518262	-0.1635173	0.026149	0.12737338	0.08402995
-0.0164595	0.82402524	-0.0358758	0.62780958	-0.1316332	0.07409127
-0.08148	0.27021351	0.02401818	0.74554806	-0.3048374	2.45E-05
-0.2143201	0.00339587	-0.2819066	0.00010128	0.33579038	2.97E-06
-0.2264023	0.00194259	-0.2656807	0.00025712	-0.195983	0.00750492
-0.1555063	0.03454481	-0.1812482	0.01354845	0.44355921	2.56E-10
-0.1465874	0.04647435	-0.2110021	0.00393885	-0.0864864	0.24177415
0.18760608	0.01055277	-0.1020949	0.16671237	0.2354879	0.00125194
-0.2561984	0.00043156	-0.2212342	0.00247562	-0.1870576	0.01078594
-0.0394485	0.59394476	-0.0769751	0.29767884	0.20060816	0.00618213
0.02728127	0.71241176	0.0394692	0.59375086	-0.2489873	0.0006317
0.17566863	0.01676659	0.47689181	6.77E-12	0.18146116	0.01343717
0.17672719	0.0161093	0.05696149	0.44122021	0.4362568	5.39E-10
-0.0359839	0.62677289	-0.0264455	0.72085093	0.25929625	0.00036516
0.19985567	0.00638204	0.07507344	0.3098093	0.27412462	0.00015949
-0.1495936	0.04211794	-0.0748933	0.31097518	-0.1973753	0.00708244
-0.0835083	0.25842875	-0.1359127	0.06508828	-0.2706834	0.00019412
-0.2966112	4.14E-05	-0.1947349	0.00790254	-0.3209367	8.43E-06
-0.3190822	9.56E-06	-0.1885094	0.01017845	0.09886075	0.18062933
-0.1576764	0.03207124	-0.1660189	0.02391499	0.16541769	0.02443632
0.00617445	0.93352299	-0.0916237	0.2148345	0.14398345	0.05054607
0.18505089	0.01167818	0.24101164	0.00095063	-0.2186798	0.00278533
-0.0124344	0.86659416	-0.0634529	0.39085461	0.17092527	0.02000666
0.04987321	0.50020266	0.21098907	0.00394113	0.10299025	0.16300475
-0.3094249	1.82E-05	-0.2196721	0.00266108	-0.1958964	0.00753191
0.10792002	0.14368756	-0.0612279	0.40771453	0.06491614	0.37999971
-0.1614062	0.02817117	-0.1185657	0.10796395	0.15330318	0.0372196
0.21782069	0.00289712	-0.0036014	0.96119648	0.311758	1.56E-05

-0.0932863	0.20659408	0.04227581	0.56774796	-0.2639392	0.00028318
-0.22655	0.00192902	-0.2357593	0.0012353	0.17309968	0.01845961
-0.0603067	0.41481917	-0.0906327	0.21985663	0.15995379	0.02963883
-0.0236466	0.74935262	-0.098248	0.18335972	-0.2034852	0.00546837
-0.1478985	0.04453024	-0.1010046	0.17131172	-0.2109673	0.00394494
0.07264053	0.32579441	0.2075137	0.00459291	-0.4373308	4.84E-10
-0.1155089	0.11742167	-0.0857688	0.24571724	0.12480756	0.09051536
-0.2089052	0.00432114	-0.2769882	0.00013516	0.30577762	2.31E-05
-0.200808	0.00612998	-0.2590182	0.00037071	0.14746898	0.04515951
0.02291208	0.75689077	0.04474733	0.54530704	0.1597412	0.02985904
0.16908719	0.02140074	0.08847081	0.23110131	-0.3522318	8.79E-07
0.13051543	0.07660198	0.09593602	0.19393533	-0.2708214	0.0001926
0.16656337	0.02345111	-0.0313963	0.67138616	-0.1034236	0.16123235
-0.1668629	0.02319926	-0.0477568	0.51858479	0.28510861	8.37E-05
0.04996714	0.49939486	-0.0313123	0.67221497	-0.0093903	0.89905244
-0.2118717	0.00378945	-0.1374516	0.06207802	0.20423729	0.00529439
0.10230518	0.16583604	0.21865339	0.0027887	-0.3316675	3.99E-06
0.29580365	4.35E-05	0.12040974	0.10255431	0.32283336	7.40E-06
0.05914527	0.42387856	-0.1216846	0.09894113	-0.191752	0.0089295
0.42393668	1.82E-09	0.20669664	0.00475955	-0.209846	0.00414568
0.20907228	0.0042895	0.02526709	0.73280638	0.1761849	0.01644316
-0.3353006	3.08E-06	-0.3893074	4.33E-08	0.2452342	0.00076693
-0.0758182	0.30502031	0.2038112	0.00539234	-0.3430064	1.76E-06
-0.0219149	0.76716165	-0.084786	0.25118934	-0.3694399	2.28E-07
-0.3367598	2.77E-06	-0.2105691	0.0040152	-0.2415642	0.00092448
-0.2419164	0.00090816	-0.2422876	0.00089125	-0.0033694	0.96369386
-0.1218497	0.09848078	-0.216986	0.00300961	0.12830915	0.08175965
-0.3871516	5.21E-08	-0.3937455	2.94E-08	0.02080524	0.77863812
0.04461488	0.54649849	-0.0616227	0.40469212	0.11662289	0.11390301
-0.2007937	0.00613369	-0.2815007	0.00010374	-0.3449897	1.52E-06
0.12096578	0.10096579	0.03167058	0.66868446	0.1248242	0.09047205
-0.2784147	0.00012438	-0.3023527	2.88E-05	-0.0404341	0.58475042
0.02748286	0.71038145	-0.1433911	0.05151252	0.24766297	0.00067668
-0.2539532	0.00048649	-0.1587111	0.03094584	-0.2888628	6.67E-05
-0.4165527	3.69E-09	-0.3426543	1.80E-06	0.24231024	0.00089023
-0.2681784	0.00022361	-0.3060454	2.27E-05	0.2135985	0.00350785
-0.0704941	0.34033077	0.17278839	0.01867451	-0.3527099	8.48E-07
0.24504862	0.00077426	0.17124222	0.01977439	-0.3061766	2.25E-05
0.11035177	0.13482471	-0.0016631	0.98207497	-0.1888259	0.0100501
0.22090939	0.00251319	0.0910936	0.21751057	0.35948026	5.03E-07
-0.0205323	0.78146863	0.00020051	0.99783871	0.05690965	0.44163668
-0.3104745	1.70E-05	-0.1888955	0.01002204	-0.4782572	5.78E-12
-0.1544859	0.03576271	-0.232933	0.00141897	0.16296507	0.02666557
-0.1114059	0.13111555	-0.1726356	0.01878077	0.12398688	0.09267203
-0.0393578	0.59479392	0.05598903	0.44907048	-0.1183397	0.10864206
-0.045724	0.53656128	-0.0001107	0.99880708	0.19924299	0.00654904

-0.3189585	9.64E-06	-0.2995394	3.44E-05	0.14142552	0.05483013
-0.2442958	0.00080467	-0.2955249	4.43E-05	0.13924159	0.05872151
0.0747029	0.31221015	-0.0704802	0.34042616	0.20062357	0.00617809
-0.0701715	0.34255072	-0.077664	0.29336275	0.20907353	0.00428926
0.09832902	0.18299704	-0.0197943	0.78913565	-0.1457479	0.04775602
0.04753409	0.52053902	0.00947365	0.89816077	-0.3282144	5.09E-06
-0.3146333	1.29E-05	-0.3253497	6.22E-06	0.05064451	0.49358979
-0.1510849	0.04008736	-0.1685801	0.02179978	0.26443817	0.00027547
-0.1825986	0.01285623	-0.1607134	0.02886331	-0.2170333	0.00300314
-0.0259176	0.72619848	-0.0498204	0.50065709	-0.034775	0.63840654
-0.1936314	0.00826954	-0.0590141	0.42490879	-0.2762728	0.00014089
-0.1794937	0.01449649	-0.1649717	0.0248293	-0.170647	0.02021256
-0.1134003	0.12431322	-0.1555574	0.03448475	0.14879092	0.04324623
0.01537872	0.83541034	-0.0669101	0.36550649	-0.2615661	0.00032267
-0.0485836	0.51136233	-0.1417267	0.05431067	0.14887978	0.0431201
-0.1958804	0.00753692	-0.2057951	0.00494971	0.21120196	0.00390405
-0.1202643	0.10297309	-0.165975	0.02395271	-0.3330183	3.63E-06
-0.3231767	7.23E-06	-0.2221284	0.00237482	0.16620262	0.02375758
-0.087457	0.23651143	-0.1559554	0.03402012	0.09361726	0.20498104
0.13593767	0.06503842	-0.0198765	0.78828082	-0.0740891	0.31621377
-0.216226	0.00311547	-0.1648069	0.02497595	0.19946435	0.00648826
0.17131055	0.01972463	0.3182208	1.01E-05	0.06632127	0.36975053
-0.4175486	3.36E-09	-0.2882338	6.93E-05	0.35977162	4.91E-07
-0.2410564	0.00094848	-0.2943716	4.76E-05	0.21895016	0.00275096
-0.1788164	0.01487773	-0.04327	0.5586685	0.12977256	0.07830838
0.07320267	0.32205451	0.16290488	0.0267224	-0.3544136	7.44E-07
-0.3112271	1.62E-05	-0.2616781	0.0003207	0.06167626	0.40428308
-0.0442264	0.55000013	-0.0720812	0.32954309	0.19989638	0.00637108
0.41827982	3.13E-09	0.16502976	0.02477788	-0.0002935	0.99683649
-0.0122479	0.86857687	-0.1001389	0.17503034	-0.3225553	7.54E-06
0.34166637	1.94E-06	0.23564292	0.00124241	-0.1744161	0.01757438
-0.0599675	0.41745285	-0.254725	0.00046692	0.20529206	0.00505874
0.09254544	0.21023737	-0.0284119	0.70105153	-0.2377984	0.00111661
-0.0610083	0.40940173	0.07852228	0.28804461	-0.2749995	0.00015165
-0.0557443	0.45105834	-0.1901291	0.00953652	0.27929535	0.00011813
-0.4033004	1.26E-08	-0.1505893	0.04075283	-0.3364154	2.84E-06
-0.0653536	0.3767903	-0.1384222	0.06023897	0.2548146	0.00046469
-0.1074124	0.14559216	-0.2161838	0.00312144	0.18357739	0.01237407
0.15487892	0.03528942	-0.061531	0.40539275	0.11459698	0.12036478
-0.0453415	0.5399775	-0.0140092	0.84988673	-0.20255	0.00569185
0.30280323	2.80E-05	0.09202409	0.21282877	-0.2111099	0.00392005
-0.2747361	0.00015397	-0.1932054	0.0084152	-0.5398809	2.19E-15
-0.3146446	1.29E-05	-0.2792542	0.00011841	0.21446336	0.00337403
0.23314625	0.00140429	0.07426677	0.31505147	-0.1598807	0.02971438
-0.160191	0.02939473	-0.1308343	0.07587872	-0.2082654	0.00444425
-0.1275137	0.08368634	0.00078676	0.9915198	-0.2897468	6.32E-05

-0.0078927	0.91508586	-0.1046163	0.15642962	0.13050364	0.07662884
0.29446869	4.73E-05	0.15110599	0.04005924	-0.2947663	4.64E-05
0.33597179	2.93E-06	0.1573847	0.0323947	0.15921656	0.03040841
-0.2964052	4.19E-05	-0.3143027	1.32E-05	-0.2802854	0.00011145
0.13611587	0.06468407	0.08162865	0.26933775	-0.0406325	0.58290738
0.25071169	0.00057727	0.07496939	0.31048227	0.13710424	0.06274733
0.06830906	0.35554566	-0.0615354	0.40535917	-0.1644098	0.02533222
-0.3240073	6.82E-06	-0.195823	0.00755489	-0.2269983	0.00188837
0.042362	0.56695807	0.20533579	0.00504918	-0.2486133	0.00064412
-0.1835551	0.01238487	-0.1543673	0.03590657	-0.3331414	3.59E-06
0.35282408	8.40E-07	0.02164783	0.769919	0.22613463	0.0019674
0.0854284	0.24760292	-0.0408311	0.58106511	0.26404019	0.00028161
0.15548622	0.03456847	0.44174281	3.09E-10	-0.3163644	1.15E-05
-0.1314104	0.0745863	-0.1567276	0.03313355	0.09802021	0.18438253
-0.2136408	0.00350119	-0.1687745	0.02164608	0.00762772	0.91792602
0.16971725	0.02091371	0.05240413	0.47867885	0.25831386	0.00038512
0.02786004	0.70658818	-0.0327216	0.65837073	0.35924876	5.12E-07
-0.0870272	0.23883221	-0.0500502	0.4986814	-0.3114706	1.59E-05
-0.1402538	0.05689051	0.00100515	0.989166	-0.104693	0.15612423
-0.389522	4.25E-08	-0.3080333	1.99E-05	-0.0559289	0.44955881
-0.0242801	0.74287011	-0.1002358	0.17461129	-0.1889341	0.01000655
-0.1025663	0.16475277	-0.2312066	0.00154311	0.16117171	0.02840386
-0.3643938	3.41E-07	-0.300149	3.31E-05	0.03667119	0.62019913
-0.1154902	0.11748158	-0.1696071	0.02099815	0.14835894	0.04386384
-0.0377927	0.60953693	-0.0888013	0.22935664	0.34077339	2.07E-06
-0.2779176	0.00012804	-0.2521648	0.0005348	0.09968905	0.17698613
-0.2571386	0.00041031	-0.225165	0.0020597	0.21214316	0.00374388
-0.0990972	0.17958397	0.11376578	0.12309685	-0.3942107	2.82E-08
0.336584	2.81E-06	0.15787549	0.03185202	-0.1631765	0.02646676
-0.0613512	0.40676932	-0.0921282	0.21230962	-0.2252309	0.0020533
-0.3230947	7.27E-06	-0.3540876	7.63E-07	0.12836753	0.08161966
0.054512	0.46114294	0.0476263	0.51972946	-0.2294211	0.00168187
-0.198628	0.00672059	-0.0515372	0.48599485	-0.2374934	0.00113367
0.13137299	0.07466965	-0.0292401	0.6927715	0.15804411	0.03166736
0.33245299	3.78E-06	0.33995104	2.20E-06	-0.4692835	1.61E-11
-0.2192362	0.00271502	-0.2710503	0.00019012	0.1188121	0.10722836
-0.1848407	0.01177528	-0.161987	0.027602	0.3198722	9.06E-06
0.0895588	0.22539265	0.0662655	0.37015408	0.19003788	0.0095717
0.2174164	0.00295113	0.06646621	0.36870311	-0.2873807	7.30E-05
0.25302351	0.00051107	0.15530557	0.03478161	-0.2462271	0.00072877
-0.056857	0.44205956	-0.0320758	0.66470012	-0.1174917	0.11121633
0.07488139	0.31105212	0.05063981	0.49362993	-0.0950951	0.19789015
-0.0550327	0.45686667	-0.0767775	0.29892415	0.0895389	0.22549619
-0.0630545	0.3938427	-0.0940274	0.20299484	-0.3614806	4.30E-07
-0.126742	0.08559026	0.02596939	0.72567317	-0.0761078	0.30317145
-0.253955	0.00048645	-0.2612626	0.00032807	0.2138585	0.00346712

-0.2098214	0.00415019	-0.1580676	0.03164168	-0.2066228	0.00477489
-0.1823515	0.01298053	-0.1208218	0.10137534	-0.2904788	6.05E-05
-0.2647014	0.00027149	-0.2822394	9.93E-05	0.17905945	0.01473991
0.28859116	6.78E-05	0.20248903	0.00570669	-0.0895166	0.22561201
-0.1744488	0.01755285	0.03395447	0.64635422	-0.3077118	2.04E-05
-0.1300312	0.07771072	-0.0763102	0.3018839	-0.2581097	0.00038939
-0.3158553	1.19E-05	-0.3330464	3.62E-06	0.31888742	9.69E-06
-0.0952022	0.19738317	-0.3099373	1.76E-05	0.47292508	1.07E-11
-0.2660069	0.00025249	-0.0029414	0.96830369	-0.3725082	1.78E-07
0.46612068	2.29E-11	0.25372514	0.00049242	-0.0217005	0.76937504
0.34058081	2.10E-06	0.2923394	5.39E-05	-0.044623	0.54642522
0.03136037	0.6717406	0.02885977	0.69656913	-0.0780425	0.29100967
0.0089269	0.90400915	-0.0449675	0.54332911	0.12385751	0.09301572
0.00662224	0.92871422	-0.0163525	0.82515104	0.02617774	0.72356139
0.09523117	0.19724611	0.14375708	0.05091363	-0.2382078	0.00109407
0.17614507	0.01646792	0.07169462	0.33215045	-0.0067248	0.92761344
0.01109312	0.88087138	0.08012516	0.27828608	-0.0756599	0.30603438
-0.3224049	7.62E-06	-0.3060137	2.27E-05	0.4082306	8.01E-09
-0.0126309	0.86450582	-0.0690717	0.35018786	-0.1834863	0.01241827
0.08116218	0.27209283	-0.0991191	0.17948729	0.10519181	0.15415069
-0.2240324	0.00217249	-0.2326269	0.00144029	0.54730661	7.57E-16
0.19471581	0.00790875	0.07130902	0.33476415	-0.0458696	0.53526278
0.37636333	1.29E-07	0.17593682	0.01659789	-0.1384405	0.06020468
-0.0155746	0.83334445	-0.0814981	0.27010715	0.45389382	8.65E-11
-0.0082861	0.91087004	-0.0121374	0.86975156	0.04343529	0.55716557
-0.2882849	6.91E-05	-0.2589851	0.00037138	0.38841708	4.67E-08
0.22844535	0.00176242	0.12368959	0.09346332	-0.0347068	0.63906621
-0.0282006	0.70316978	-0.2251062	0.00206542	0.29883597	3.60E-05
-0.0639248	0.38733362	-0.1860718	0.01121647	0.30401158	2.59E-05
-0.1434502	0.05141541	-0.1026685	0.16432986	0.52730681	1.25E-14
0.13823118	0.06059734	-0.0498282	0.50059024	0.22738836	0.00185364
0.07035661	0.34127597	0.17106273	0.01990564	-0.1995875	0.00645466
-0.2463439	0.0007244	-0.2860163	7.93E-05	0.07229876	0.32808185
-0.1417548	0.05426232	-0.2143712	0.00338807	0.17576741	0.01670428
-0.1391528	0.0588844	-0.088184	0.23262272	-0.1301402	0.07746021
-0.1456124	0.04796565	-0.1193388	0.1056693	0.26693956	0.00023968
0.06408033	0.3861775	-0.0114601	0.87696065	-0.4366289	5.19E-10
-0.1904653	0.00940788	-0.2825181	9.77E-05	0.14185968	0.05408253
0.18720057	0.01072473	0.15237439	0.03839853	-0.3327657	3.69E-06
0.11289822	0.12599943	0.053068	0.4731173	0.02070837	0.77964234
-0.1633982	0.02625965	-0.0736805	0.3188974	-0.1083193	0.14220275
-0.113995	0.12233854	0.00919103	0.90118323	-0.2679368	0.00022666
-0.4179563	3.23E-09	-0.2774375	0.00013167	0.44328333	2.63E-10
0.08280389	0.26248091	0.07971036	0.28078978	-0.1534103	0.03708564
-0.2939497	4.88E-05	-0.256781	0.00041828	0.00354573	0.96179558
0.36227429	4.04E-07	0.06413835	0.38574669	-0.0346358	0.639752

-0.0358349	0.62820173	0.02861286	0.69903871	-0.1407484	0.05601312
-0.2100356	0.00411109	-0.2960181	4.29E-05	0.15556896	0.03447122
0.03482516	0.6379223	-0.0622206	0.40013983	0.29508688	4.55E-05
-0.2117797	0.00380503	-0.310272	1.72E-05	0.35518611	7.01E-07
-0.1692824	0.02124878	-0.1712182	0.01979193	0.34206332	1.88E-06
-0.0924462	0.21072905	-0.1540474	0.03629719	0.07134714	0.33450515
0.38582133	5.84E-08	0.11913502	0.10627039	-0.2876128	7.20E-05
6.58E-05	0.99929064	-0.0643455	0.38421078	0.25882012	0.00037471
-0.1768051	0.01606183	-0.2204444	0.00256787	0.27150092	0.00018531
-0.0226039	0.76006005	-0.0319064	0.6663649	0.10075581	0.17237447
0.04486193	0.54427714	0.15885833	0.03078847	0.00977074	0.89498527
0.02526781	0.73279906	-0.0002357	0.99745908	-0.5303824	8.19E-15
0.07066836	0.33913568	0.3249897	6.38E-06	-0.2569459	0.00041459
0.13201494	0.07324915	0.03518612	0.63444023	0.31537659	1.23E-05
-0.0614299	0.40616615	-0.1255962	0.08848079	-0.4250211	1.64E-09
-0.1983468	0.00680036	-0.2222084	0.00236598	0.21768955	0.00291454
-0.14443	0.04982751	0.1427747	0.05253452	-0.2687233	0.00021686
0.11672817	0.11357477	0.03628949	0.62384629	0.22015793	0.00260209
0.06739928	0.36200415	-0.0026106	0.97186658	0.38090911	8.85E-08
-0.2345881	0.0013086	-0.2905638	6.02E-05	0.46831721	1.79E-11
-0.0687689	0.35230865	-0.149796	0.04183741	0.03658764	0.62099667
-0.1885965	0.01014297	-0.0159835	0.8290347	0.35700904	6.09E-07
-0.3807822	8.95E-08	-0.3010469	3.13E-05	0.17678999	0.01607102
-0.0008931	0.99037373	-0.0859129	0.24492149	0.25642094	0.00042644
0.06918421	0.34940155	-0.0647182	0.38145749	0.27105404	0.00019008
-0.0630785	0.39366241	0.00076609	0.99174256	0.15690851	0.03292873
0.03537196	0.63265063	-0.0610004	0.40946229	-0.0857505	0.24581837
-0.0423496	0.56707144	-0.0653958	0.37648149	0.24348927	0.00083847
-0.3152635	1.24E-05	-0.2553653	0.00045124	0.38414594	6.74E-08
-0.3178189	1.04E-05	-0.2665526	0.00024492	0.22022898	0.00259356
-0.0861158	0.24380471	-0.1893952	0.00982282	0.27720112	0.0001335
0.01591412	0.82976576	0.01305594	0.85999236	-0.2363693	0.00119863
-0.0669052	0.36554184	-0.2078627	0.00452334	0.35280674	8.42E-07
-0.1035335	0.16078525	-0.1860093	0.01124425	0.19120296	0.00913093
0.35296278	8.32E-07	0.14221685	0.05347384	-0.0042276	0.95445595
0.38484657	6.35E-08	0.11356383	0.12376783	0.09152138	0.21534913
-0.1428926	0.05233777	-0.1074314	0.14552083	-0.0326232	0.6593333
-0.1005641	0.1731968	0.01682533	0.82017944	-0.1253671	0.08906807
0.2096399	0.00418355	0.10557427	0.15265023	0.10208782	0.16674199
0.03234623	0.66204696	-0.1439054	0.05067253	0.18455851	0.01190679
0.10108853	0.1709545	-0.0427697	0.56322852	-0.2563781	0.00042742
-0.1217293	0.09881641	-0.1757989	0.01668448	-0.1687951	0.02162984
-0.2956982	4.38E-05	-0.167174	0.02294008	-0.2230108	0.00227901
-0.1659097	0.02400895	-0.1520906	0.03876493	-0.4794773	5.02E-12
0.35572964	6.72E-07	0.21417572	0.00341801	-0.2995289	3.44E-05
0.22777104	0.00182013	0.0677402	0.35957548	0.19490813	0.00784624

-0.1331807	0.07072567	-0.2342119	0.00133297	0.16675529	0.02328947
0.38476509	6.39E-08	0.11904513	0.10653637	-0.2531096	0.00050875
0.03720473	0.61511666	-0.0317953	0.66745713	0.30435848	2.53E-05
0.43514936	6.03E-10	0.61975852	5.18E-21	-0.2118308	0.00379637
-0.3452401	1.49E-06	-0.1275543	0.08358721	-0.1223957	0.09696988
-0.09998	0.17571934	0.05256476	0.47732991	-0.2075643	0.00458277
-0.0743051	0.31480142	0.01709256	0.81737298	0.23110881	0.00155043
0.0319594	0.66584396	-0.0787727	0.28650496	0.21259057	0.00366984
-0.3387387	2.40E-06	-0.1128714	0.12609005	-0.0822758	0.26554689
0.36954256	2.26E-07	0.12309209	0.09506987	0.3223319	7.66E-06
-0.2211053	0.00249047	-0.0279049	0.70613729	-0.1744125	0.01757675
0.20494016	0.00513629	0.0502943	0.49658657	0.09497657	0.19845206
-0.1532564	0.03727827	-0.2348835	0.00128975	-0.1599866	0.02960498
0.09928929	0.17873762	0.00167301	0.98196842	-0.1598949	0.02969966
-0.0947638	0.19946409	-0.157871	0.03185697	0.2131728	0.00357546
-0.0397446	0.5911752	-0.0598513	0.4183578	-0.1059934	0.15101847
0.23596639	0.00122274	0.09681471	0.18986487	0.01878314	0.79967395
-0.0195473	0.79170656	-0.0955718	0.19564117	0.16698563	0.02309672
-0.0691386	0.34972011	-0.1525112	0.03822288	0.12735613	0.08407227
-0.2216766	0.00242528	-0.2331794	0.00140202	0.23197214	0.00148689
0.03269955	0.65858643	0.17862664	0.01498607	-0.303068	2.75E-05
-0.0198023	0.78905271	0.09054254	0.22031766	-0.012462	0.86630071
0.03178518	0.6675568	-0.0455604	0.53802116	0.13517306	0.06657688
-0.0256182	0.72923742	0.00693135	0.92539615	0.12497034	0.09009239
-0.2772642	0.00013301	-0.2474563	0.00068396	-0.4430031	2.71E-10
0.10392656	0.15919379	-0.0593327	0.4224084	-0.1751334	0.0171077
-0.1222181	0.09745919	-0.075095	0.30967	-0.1540779	0.03625982
-0.2050207	0.00511846	-0.0794449	0.28240016	-0.1983966	0.00678618
0.22969324	0.00166001	0.43206288	8.20E-10	-0.1344114	0.06813871
-0.1567608	0.03309593	-0.2140401	0.00343893	-0.3944676	2.76E-08
-0.2279559	0.00180414	-0.0949446	0.19860387	-0.1001974	0.17477713
-0.2125905	0.00366985	-0.2690406	0.00021302	0.40460229	1.12E-08
0.05316047	0.47234544	0.03516492	0.63464454	0.222577	0.00232566
-0.0184461	0.80319498	0.06012334	0.41624162	0.10746244	0.14540371
-0.0133795	0.85655973	-0.053492	0.46958406	0.04979168	0.50090433
-0.2616889	0.00032051	-0.1684902	0.02187125	0.12447856	0.09137508
0.02676485	0.71762202	-0.1047157	0.15603426	-0.0222251	0.76396171
0.54923691	5.72E-16	0.26287214	0.00030035	-0.1716969	0.01944528
0.13794811	0.06113161	0.02691561	0.71609964	-0.2612515	0.00032827
0.30776112	2.03E-05	0.06803452	0.35748693	-0.1989204	0.00663852
0.10333628	0.16158848	0.18174722	0.01328894	-0.2278469	0.00181356
-0.286604	7.65E-05	-0.3542156	7.56E-07	0.238962	0.00105365
-0.2052284	0.00507269	-0.3219836	7.84E-06	0.03343136	0.65144193
-0.1032853	0.16179645	-0.0154154	0.83502348	-0.2802326	0.0001118
-0.482685	3.46E-12	-0.472354	1.14E-11	0.29481655	4.63E-05
0.17195948	0.01925738	-0.0364696	0.6221244	0.18182931	0.01324667

-0.0838535	0.25645921	0.02604013	0.72495594	-0.1106875	0.13363487
-0.2895246	6.41E-05	-0.1623404	0.02726043	-0.2460643	0.00073491
-0.1341013	0.06878325	-0.036002	0.62659939	-0.0246348	0.739248
-0.1381222	0.0608026	-0.0302529	0.68269465	-0.0273765	0.71145212
-0.1675949	0.0225935	-0.1103643	0.13478032	0.19077479	0.00929078
0.50431403	2.50E-13	0.25413024	0.00048194	-0.1217803	0.09867399
0.06694088	0.3652857	0.09580384	0.19455309	0.18423016	0.01206141
-0.11428	0.12140095	-0.0884595	0.23116115	-0.2938872	4.90E-05
-0.1536267	0.03681618	-0.0564055	0.44569889	0.24564203	0.00075104
-0.2158144	0.00317418	-0.1715931	0.01951996	0.25464869	0.00046882
-0.2338436	0.00135724	-0.1351686	0.06658598	0.14838657	0.04382411
-0.2039922	0.00535053	-0.0747053	0.31219429	-0.305896	2.29E-05
-0.4085651	7.77E-09	-0.2919247	5.53E-05	0.1808881	0.01373848
-0.0722789	0.32821492	0.13535296	0.06621224	-0.1913159	0.00908919
-0.3604073	4.67E-07	-0.3145023	1.30E-05	0.34769471	1.24E-06
-0.1217389	0.09878951	-0.2327033	0.00143495	0.24037984	0.00098135
0.04435185	0.54886832	-0.1127985	0.12633648	0.13294191	0.07123673
-0.2170172	0.00300534	-0.3346947	3.22E-06	0.13249203	0.07220771
-0.2390674	0.00104812	-0.2486074	0.00064431	0.08554117	0.24697705
-0.1815786	0.01337615	-0.0687543	0.3524116	-0.1402979	0.0568119
-0.1593628	0.0302544	-0.0843873	0.2534329	-0.2237444	0.00220205
0.02753702	0.70983635	-0.016575	0.82281083	0.27489585	0.00015256
-0.2695114	0.00020743	-0.3147344	1.28E-05	0.45739505	5.94E-11
-0.1677158	0.02249475	-0.1891026	0.00993905	0.21202342	0.00376392
-0.160671	0.02890618	-0.0917191	0.2143551	-0.1585025	0.03116997
-0.0529381	0.4742031	0.0726972	0.3254161	-0.2042323	0.00529554
-0.1424653	0.05305385	-0.2325564	0.00144525	0.1830822	0.01261598
-0.1086397	0.14101979	-0.1744754	0.01753539	0.10455085	0.15669021
-0.1674454	0.02271609	-0.2228486	0.00229636	0.12013734	0.10333966
0.04635512	0.53094683	0.25701854	0.00041297	-0.3889329	4.47E-08
-0.0483091	0.51375464	0.01844985	0.80315547	0.1225356	0.09658585
0.12917108	0.07971238	0.09571629	0.19496308	0.39725361	2.16E-08
-0.1451131	0.04874466	-0.0870047	0.23895385	-0.0428439	0.56255127
-0.2168451	0.00302899	-0.0350091	0.63614728	-0.2928915	5.21E-05
-0.2357859	0.00123368	-0.1776638	0.01554658	-0.1757365	0.01672378
-0.2255764	0.00202007	-0.2505183	0.00058315	0.33974074	2.23E-06
-0.1500574	0.04147741	-0.0046301	0.9501249	-0.0477397	0.51873472
0.16603366	0.02390229	-0.0055749	0.93996531	0.15056062	0.04079164
0.04264173	0.56439795	-0.0399172	0.58956394	0.20541287	0.00503236
-0.2176983	0.00291338	-0.2437489	0.00082745	0.32444719	6.62E-06
0.0111467	0.88030023	0.05201389	0.4819645	0.41224428	5.53E-09
0.11888846	0.10700123	0.04654927	0.52922572	0.01464661	0.84314221
0.08976403	0.22432712	0.00283381	0.96946224	-0.1705697	0.02027004
0.00975103	0.89519588	-0.0803008	0.27723034	0.47820351	5.82E-12
-0.1640206	0.02568568	-0.1210875	0.10062073	-0.2198469	0.00263972
-0.1877903	0.01047548	-0.2225339	0.00233034	0.24991209	0.00060195

-0.1779874	0.01535621	-0.2000763	0.00632284	0.26869369	0.00021722
-0.3518423	9.06E-07	0.05317749	0.47220341	-0.2996398	3.42E-05
0.49188664	1.16E-12	0.36747014	2.67E-07	-0.355171	7.02E-07
-0.3279904	5.17E-06	-0.1327553	0.07163826	0.09847439	0.18234748
0.06199192	0.40187745	-0.0507571	0.49262817	0.2272723	0.00186391
0.20251339	0.00570076	0.06706548	0.36439194	0.23925578	0.00103828
-0.1458903	0.04753654	-0.3203022	8.80E-06	0.17412849	0.01776455
-0.1727751	0.01868376	-0.1574402	0.03233298	0.18589595	0.01129482
-0.0878955	0.234161	0.00800894	0.91383958	-0.252123	0.00053598
0.27579367	0.00014485	0.16599801	0.02393293	-0.3162186	1.16E-05
-0.0119518	0.87172603	-0.0393988	0.59440969	0.36320076	3.75E-07
-0.0033539	0.96386104	-0.0893865	0.22628982	-0.0904142	0.220975
-0.1541092	0.03622142	-0.0875146	0.23620192	-0.2195818	0.00267217
-0.029863	0.68656694	-0.2191803	0.00272201	0.20125844	0.0060139
-0.0550292	0.45689538	-0.2022544	0.00576415	0.15556327	0.03447791
-0.17909	0.01472265	-0.1808946	0.01373501	0.10751452	0.14520759
-0.2461672	0.00073103	-0.0551553	0.45586334	0.04179289	0.5721834
0.16573469	0.02416023	-0.0303049	0.68217923	0.17491506	0.01724861
-0.2548558	0.00046367	-0.3158108	1.19E-05	0.21990974	0.00263207
0.12311583	0.09500564	-0.0161003	0.82780487	0.19070082	0.00931864
0.0611775	0.40810143	-0.0205911	0.78085843	0.20437329	0.00526347
-0.1773718	0.01572012	-0.1892095	0.00989645	0.16814495	0.02214736
-0.3209307	8.43E-06	-0.3223047	7.67E-06	0.28292873	9.53E-05
-0.1860252	0.01123717	-0.0773986	0.29502072	0.09243445	0.21078713
0.11537366	0.11785465	0.00084982	0.99084007	-0.2334237	0.00138539
-0.3449257	1.52E-06	-0.1720321	0.01920569	-0.1577852	0.0319513
-0.2278128	0.0018165	-0.2674723	0.00023264	0.19680437	0.00725306
0.04511824	0.5419773	0.31829023	1.01E-05	-0.3241618	6.75E-06
0.02353499	0.75049645	0.04446196	0.54787568	-0.2575474	0.00040138
-0.0562882	0.44664733	0.04068082	0.58245904	-0.1214827	0.09950652
0.08650091	0.24169487	0.04632082	0.53125117	-0.1115481	0.13062133
0.32417006	6.75E-06	0.4945411	8.39E-13	-0.1858598	0.01131099
0.0750063	0.31024342	0.13701093	0.06292812	-0.1571473	0.03265998
-0.0728635	0.3243076	-0.0372208	0.61496366	0.18484451	0.01177352
-0.0024143	0.97398142	-0.1269435	0.08508979	0.28486937	8.49E-05
-0.2433656	0.00084376	-0.0937912	0.20413687	0.01970359	0.79007966
-0.4027047	1.33E-08	-0.3004085	3.26E-05	0.12710008	0.08470247
-0.1674324	0.02272677	-0.1124868	0.12739421	0.20514084	0.00509194
0.33987024	2.21E-06	0.15152642	0.03950219	-0.1868412	0.01087919
-0.2555709	0.0004463	-0.3765414	1.27E-07	0.23575298	0.00123569
-0.0437458	0.55434761	-0.0082969	0.91075466	-0.448885	1.47E-10
-0.1795236	0.01447987	-0.2050538	0.00511113	0.3920008	3.43E-08
-0.1126973	0.12667908	-0.0243508	0.74214767	-0.4403067	3.57E-10
0.21033696	0.00405668	0.04282293	0.56274259	0.27502932	0.00015139
-0.1609612	0.02861411	-0.240952	0.00095349	0.19500177	0.00781597
-0.2673452	0.0002343	-0.2566115	0.0004221	0.16015339	0.02943336

-0.23816	0.00109668	-0.2690211	0.00021325	0.34068961	2.08E-06
0.06409603	0.38606087	-0.0610923	0.40875618	0.3724706	1.78E-07
0.11191922	0.12933803	0.30294194	2.77E-05	-0.0033455	0.9639514
-0.1076345	0.1447567	-0.1505364	0.04082449	-0.144257	0.05010482
-0.0395493	0.5930017	-0.1449543	0.04899467	0.26743046	0.00023319
0.01096984	0.88218566	0.02706991	0.71454257	0.44156471	3.14E-10
-0.2543349	0.00047672	-0.0336301	0.64950727	-0.173673	0.01806944
-0.5550712	2.42E-16	-0.3310819	4.16E-06	0.05161725	0.48531649
0.0693034	0.34856996	0.06221372	0.40019225	-0.3637608	3.59E-07
0.42410042	1.79E-09	0.25360705	0.00049551	-0.2350341	0.00128023
-0.2172952	0.00296749	-0.0795602	0.28169978	-0.3571851	6.01E-07
-0.4288114	1.13E-09	-0.4347021	6.30E-10	0.21640762	0.00308987
0.03834415	0.60432396	-0.1196	0.10490278	0.13962297	0.05802601
0.26084565	0.00033563	0.20078335	0.0061364	-0.2617328	0.00031974
-0.3252558	6.26E-06	-0.2650452	0.00026636	0.21680165	0.00303499
0.12673017	0.08561963	-0.024968	0.7358514	0.13992773	0.05747515
0.15995052	0.02964221	0.06130877	0.40709439	0.15730128	0.03248771
-0.3206499	8.59E-06	-0.1599798	0.02961194	-0.1993703	0.00651401
-0.0722795	0.32821092	-0.1733631	0.0182794	0.2358265	0.00123121
-0.0340855	0.64508233	-0.0958127	0.19451164	0.24502133	0.00077534
-0.1274336	0.08388227	-0.1265879	0.08597456	0.2248652	0.00208902
0.11073317	0.13347353	-0.0586189	0.42802212	0.10594354	0.15121199
0.09363989	0.20487109	-0.0606027	0.41252854	0.13440771	0.06814645
0.10524282	0.15394996	-0.0352887	0.63345244	0.34514973	1.50E-06
-0.1353815	0.06615452	-0.1753718	0.01695505	0.25156114	0.00055208
0.00477102	0.94860916	-0.084967	0.25017498	0.13116312	0.07513887
0.18062338	0.01387966	0.0809926	0.27309919	0.22052294	0.00255856
0.18274809	0.01278154	0.04336856	0.55777203	0.26295572	0.00029897
0.24862729	0.00064365	0.19090454	0.00924208	-0.1322442	0.07274721
0.01346179	0.85568684	-0.0234563	0.75130275	0.1901996	0.00950942
-0.1513044	0.03979553	-0.0402836	0.58614961	-0.428865	1.13E-09
-0.0478539	0.51773369	0.00738485	0.92053054	-0.2138289	0.00347173
-0.0546541	0.45997391	-0.1075269	0.1451611	0.13472351	0.06749519
0.58239703	3.45E-18	0.31004045	1.75E-05	-0.1249053	0.09026125
-0.2222693	0.00235928	-0.1781558	0.01525791	-0.3125052	1.49E-05
0.23302397	0.00141269	0.29133561	5.74E-05	-0.0586491	0.4277833
0.05890602	0.42575893	-0.0876633	0.23540376	0.09418996	0.20221164
-0.1106529	0.13375687	0.1535507	0.03691063	-0.2865652	7.67E-05
-0.0534999	0.46951835	-0.1469763	0.04589054	0.07062703	0.3394189
-0.1705411	0.02029138	-0.1999661	0.00635236	0.28689232	7.52E-05
-0.2095071	0.00420813	-0.3349278	3.16E-06	0.35825515	5.53E-07
0.51840414	4.09E-14	0.26035554	0.00034472	-0.1287706	0.08065835
-0.2524279	0.00052742	-0.2023087	0.00575082	0.23854417	0.00107587
0.43128254	8.86E-10	0.16716807	0.02294504	-0.1145654	0.12046781
0.13205769	0.07315533	0.05502894	0.45689744	0.10012257	0.17510111
-0.0615224	0.40545844	-0.0275019	0.71018944	-0.4334628	7.13E-10

-0.2279179	0.00180742	-0.1351944	0.06653344	-0.1271674	0.08453653
-0.0448127	0.54471915	-0.2332893	0.00139451	0.06052651	0.41311723
-0.0124044	0.86691282	-0.236382	0.00119788	0.10392361	0.15920569
-0.0943153	0.2016093	-0.0978793	0.18501737	0.20159902	0.00592743
-0.4488627	1.47E-10	-0.1907333	0.00930641	-0.0763841	0.30141428
-0.1603041	0.02927899	-0.1021399	0.16652447	0.0854803	0.24731475
-0.4341222	6.68E-10	-0.1555649	0.03447598	0.43637927	5.32E-10
-0.0018576	0.97997914	0.07848185	0.28829367	-0.4726694	1.10E-11
-0.3087963	1.90E-05	-0.2910373	5.84E-05	-0.0273604	0.71161435
-0.0193703	0.79355022	0.08740059	0.2368153	-0.2178392	0.00289467
-0.2856945	8.08E-05	-0.1345864	0.06777738	-0.2437966	0.00082544
0.10894694	0.13989222	0.18379818	0.01226753	-0.4505599	1.23E-10
-0.2720211	0.0001799	-0.1962005	0.00743748	-0.2844304	8.72E-05
-0.0705446	0.33998415	-0.0366299	0.62059346	-0.4913938	1.23E-12
-0.1043036	0.15767836	-0.0518515	0.4833355	0.15327243	0.03725814
-0.0349623	0.63659887	-0.0995309	0.1776774	-0.4104942	6.50E-09
-0.1323805	0.07245016	-0.1679915	0.02227102	0.28904431	6.60E-05
-0.0818055	0.26829808	-0.1098933	0.13646273	-0.2270461	0.00188409
-0.1651108	0.02470622	-0.1190668	0.10647222	-0.2983377	3.71E-05
-0.0879118	0.23407384	-0.0182904	0.80482269	-0.2785605	0.00012332
-0.2252007	0.00205623	-0.2020771	0.00580792	-0.2069181	0.00471386
0.05819266	0.43139425	0.01602952	0.82855037	0.34698137	1.31E-06
0.08208634	0.26665308	-0.1181707	0.10915122	0.19400443	0.00814381
-0.1749904	0.01719986	-0.0055666	0.94005398	0.25924958	0.00036609
-0.0073751	0.92063471	0.00379532	0.95910859	-0.3544707	7.41E-07
-0.0848504	0.25082833	-0.0862988	0.24280057	-0.28163	0.00010295
0.03936756	0.59470259	-0.0282816	0.70235665	0.20565047	0.00498084
-0.0079296	0.91469043	-0.0502311	0.49712808	-0.0723275	0.32788898
-0.3133694	1.40E-05	-0.2917371	5.60E-05	0.03629629	0.62378124
0.00969055	0.89584216	-0.0769832	0.29762748	-0.068822	0.35193632
0.19633509	0.00739602	0.06404845	0.3864143	-0.1300222	0.07773156
0.17496737	0.01721476	0.31699018	1.10E-05	-0.181213	0.01356694
0.06307365	0.39369851	-0.1196393	0.10478774	0.20901541	0.00430024
0.32003117	8.96E-06	0.16287254	0.02675298	-0.2382773	0.00109029
-0.0371976	0.61518428	-0.060864	0.41051211	0.06902713	0.35049938
-0.2135769	0.00351125	-0.0701446	0.34273652	0.08406201	0.25527412
0.06336556	0.39150857	0.00970747	0.89566132	-0.2224433	0.00234022
-0.2751006	0.00015077	-0.2112053	0.00390347	0.17741213	0.01569607
0.19042626	0.00942272	0.01904756	0.79691458	0.22447515	0.00212775
-0.3313396	4.09E-06	-0.2033987	0.00548871	0.53846267	2.67E-15
-0.1993443	0.00652116	-0.110097	0.13573322	-0.2163215	0.00310198
0.12590879	0.08768458	0.30680123	2.16E-05	-0.2416689	0.0009196
0.48223477	3.64E-12	0.27206581	0.00017944	-0.1696906	0.02093411
-0.1109565	0.13268733	0.01072956	0.88474814	-0.0081887	0.91191303
-0.2899913	6.23E-05	-0.2306631	0.0015842	0.25120595	0.00056249
-0.2529316	0.00051357	-0.2433083	0.00084623	0.12871218	0.08079717

-0.0710774	0.33634019	0.03497461	0.63647964	0.08185656	0.26799863
-0.119841	0.10419943	-0.2657919	0.00025553	0.19991726	0.00636546
0.03161306	0.66925077	-0.0979544	0.18467872	0.24369113	0.00082989
0.3629655	3.82E-07	0.16927494	0.0212546	-0.1022595	0.16602599
-0.0990025	0.18000203	-0.0411905	0.5777387	-0.0242733	0.74293996
-0.0268691	0.71656963	-0.0046499	0.94991232	0.24576792	0.0007462
0.03530675	0.63327837	-0.0492733	0.50537819	-0.2784128	0.00012439
0.06057988	0.41270462	-0.068792	0.35214701	-0.1806246	0.01387902
-0.0843672	0.25354684	-0.097572	0.18640729	0.25019157	0.00059321
-0.1389754	0.05921099	-0.1798001	0.01432686	0.29373799	4.95E-05
-0.1688018	0.02162453	-0.0458648	0.53530583	-0.1240875	0.09240532
0.47565028	7.81E-12	0.28631465	7.78E-05	-0.0852261	0.24872852
-0.0432299	0.5590334	-0.0295121	0.69005912	-0.2491865	0.00062518
0.06927719	0.34875266	0.08194776	0.26746401	-0.157435	0.03233874
-0.0254234	0.7312171	-0.0699549	0.34404648	0.14960288	0.04210509
-0.0476053	0.51991374	-0.0339725	0.64617933	-0.2805648	0.00010964
-0.0823858	0.26490622	-0.1503728	0.0410465	0.1562119	0.0337234
-0.2709826	0.00019085	-0.2526879	0.00052023	-0.0407954	0.58139639
-0.050945	0.49102583	-0.1303361	0.07701125	0.1144059	0.12098861
-0.3174967	1.06E-05	-0.2099105	0.00413389	0.0803024	0.27722089
-0.136939	0.06306775	-0.087622	0.23562514	0.30658147	2.19E-05
0.57416287	1.29E-17	0.40123276	1.51E-08	-0.2346744	0.00130306
-0.2738817	0.00016173	-0.1955966	0.00762608	0.09649221	0.19135148
-0.2327082	0.0014346	-0.1285483	0.08118751	-0.3564166	6.38E-07
0.05623252	0.4470975	-0.0447152	0.54559601	0.19504014	0.00780359
-0.0686369	0.353236	-0.1296157	0.07867257	0.21052078	0.0040238
-0.1967465	0.00727056	0.01254907	0.8653754	-0.4068178	9.12E-09
-0.0180728	0.80709864	-0.2458789	0.00074195	0.11918503	0.10612263
0.65784436	2.62E-24	0.30016533	3.31E-05	-0.2723533	0.00017652
0.37823656	1.11E-07	0.29630729	4.22E-05	-0.154214	0.03609333
0.16115369	0.02842181	0.16058245	0.02899578	0.20366576	0.00542615
-0.0057975	0.93757277	0.01956599	0.79151198	-0.2371211	0.00115482
-0.2007869	0.00613546	-0.0045295	0.95120748	-0.0721559	0.32904101
0.04782785	0.51796206	-0.1451827	0.04863542	0.27547458	0.00014755
-0.0129183	0.86145329	-0.1957034	0.0075924	0.2659554	0.00025321
-0.2619773	0.00031548	0.01405473	0.84940424	-0.0090125	0.90309328
-0.086128	0.24373774	-0.231037	0.00155583	0.24700115	0.00070025
-0.2062057	0.00486228	-0.2852016	8.32E-05	0.21334426	0.00354809
-0.2340655	0.00134257	-0.2311597	0.00154662	-0.3808045	8.93E-08
-0.0129453	0.86116708	-0.1627934	0.02682794	0.17952641	0.0144783
0.29717255	3.99E-05	0.22309155	0.00227042	-0.0675505	0.3609259
0.04364859	0.55522926	-0.0605693	0.41278654	-0.3186864	9.82E-06
-0.2649056	0.00026843	-0.2542235	0.00047955	0.06610008	0.37135256
-0.1963154	0.00740206	-0.1412283	0.05517249	-0.2331922	0.00140114
-0.0862489	0.24307416	-0.0128377	0.86230941	-0.0525957	0.47707071
-0.2538902	0.00048812	-0.255051	0.00045887	-0.0025421	0.97260448

-0.0900665	0.2227633	-0.0934096	0.20599207	-0.470433	1.41E-11
0.48816168	1.81E-12	0.29083018	5.92E-05	-0.2967339	4.11E-05
0.31383938	1.36E-05	0.15004085	0.04150012	0.14532853	0.04840729
-0.1745756	0.01746968	-0.1903711	0.00944374	0.49287852	1.03E-12
-0.2992261	3.51E-05	-0.170373	0.02041704	-0.2945546	4.70E-05
0.10901294	0.13965093	0.13735696	0.06225983	-0.3970314	2.20E-08
-0.0523105	0.47946584	-0.1051348	0.15437536	0.19990787	0.00636799
0.42088973	2.44E-09	0.26422741	0.00027871	-0.2407064	0.00096536
-0.1816427	0.01334294	-0.2172493	0.00297371	-0.1845672	0.01190272
0.11162482	0.13035522	-0.0306421	0.67883794	0.15209468	0.03875967
-0.0694102	0.34782569	-0.2479098	0.00066808	0.08652894	0.24154179
0.00458539	0.9506061	-0.1338059	0.06940165	0.21445559	0.00337521
-0.0651126	0.37855674	-0.1049022	0.15529432	-0.2451381	0.00077072
0.1969781	0.00720076	0.07645851	0.30094236	-0.1169169	0.11298815
0.34309951	1.74E-06	0.16835561	0.02197851	0.05044201	0.49532141
-0.3055617	2.34E-05	-0.1780828	0.01530047	0.06612447	0.37117566
0.08754805	0.23602217	-0.2129557	0.00361039	0.16937204	0.02117936
-0.0678974	0.35845903	-0.0675953	0.36060632	0.26658063	0.00024454
-0.1535586	0.03690079	-0.221319	0.0024659	0.17994196	0.01424892
-0.4530355	9.48E-11	-0.3400207	2.19E-06	0.28617395	7.85E-05
0.24677794	0.00070837	0.10207647	0.16678942	0.04065741	0.58267628
-0.1686172	0.02177043	-0.1011962	0.17049673	0.33835042	2.47E-06
0.24744742	0.00068428	0.17704588	0.01591587	-0.2138807	0.00346366
-0.5144161	6.89E-14	-0.3804029	9.24E-08	0.26437343	0.00027646
-0.3103614	1.71E-05	-0.256662	0.00042096	-0.095246	0.19717613
-0.4520774	1.05E-10	-0.3512676	9.46E-07	0.11931578	0.10573706
0.12416291	0.09220603	-0.0202938	0.7839436	0.27118575	0.00018866
-0.211384	0.0038726	-0.3010944	3.12E-05	0.06435228	0.38416078
-0.3764421	1.28E-07	-0.2397719	0.00101178	0.53405142	4.94E-15
-0.1047635	0.15584409	-0.1901282	0.00953688	0.02165166	0.76987941
-0.1063497	0.14964211	-0.0832162	0.26010416	0.20551883	0.00500933
-0.2136087	0.00350624	-0.2535242	0.0004977	-0.2968191	4.08E-05
-0.2907098	5.96E-05	-0.1487181	0.04334985	-0.0929662	0.20816226
-0.3609611	4.48E-07	-0.2702852	0.00019855	0.03777116	0.60974084
-0.0559363	0.449498	-0.1758488	0.0166531	0.08947118	0.22584867
0.06329918	0.39200591	0.02003138	0.7866705	0.13436974	0.06822509
0.08773937	0.23499594	0.08854087	0.23073066	-0.1871152	0.01076125
0.16104572	0.02852955	0.15795239	0.03176768	-0.2794284	0.00011721
-0.1462016	0.04705979	-0.1744292	0.01756572	0.18051174	0.01393957
-0.3616597	4.24E-07	-0.0825769	0.26379597	-0.2881917	6.95E-05
-0.2938965	4.90E-05	-0.3390799	2.34E-06	0.09778195	0.18545676
0.01122454	0.87947073	-0.0519595	0.48242375	-0.0091308	0.90182712
-0.228609	0.00174867	-0.2842924	8.79E-05	0.26382008	0.00028505
0.10805844	0.14317145	0.0092037	0.90104763	0.31831413	1.01E-05
0.01898201	0.79759845	-0.0741309	0.31594038	0.18308275	0.01261571
-0.2398294	0.00100886	-0.2476378	0.00067756	0.48115931	4.13E-12

-0.2458836	0.00074177	-0.1740425	0.01782176	0.09266659	0.20963842
-0.2025183	0.00569957	-0.2047198	0.00518541	0.18685988	0.0108711
0.01692416	0.81914122	-0.1194295	0.10540276	0.38011516	9.46E-08
0.26337802	0.00029209	0.25103812	0.00056747	-0.3526807	8.50E-07
-0.1460549	0.04728396	0.04282722	0.56270348	-0.1987946	0.00667373
-0.1178245	0.11020052	-0.0617628	0.40362242	-0.1875626	0.01057111
-0.1610208	0.02855443	-0.1541991	0.03611149	0.04441409	0.54830712
0.13103573	0.07542484	-0.0774194	0.29489028	0.31167679	1.57E-05
0.19371136	0.00824244	0.36097496	4.47E-07	-0.2673335	0.00023446
-0.4145828	4.44E-09	-0.3831023	7.36E-08	0.16188501	0.0277012
-0.0132227	0.85822292	-0.0860059	0.24440946	0.26072066	0.00033793
0.05003949	0.49877309	-0.020821	0.77847437	0.21457321	0.00335736
-0.2439904	0.00081732	-0.3080238	2.00E-05	0.21701881	0.00300512
0.05522415	0.45529984	0.24801753	0.00066436	0.40827878	7.98E-09
-0.0013917	0.98499969	0.0174493	0.81363018	-0.1075339	0.14513483
-0.2184235	0.00281826	-0.2315993	0.00151404	0.13305493	0.07099448
0.07814351	0.29038364	0.1828942	0.0127089	-0.3446837	1.55E-06
-0.4053194	1.05E-08	-0.3601125	4.78E-07	0.14827287	0.04398777
-0.1875012	0.010597	-0.1967631	0.00726553	0.1663219	0.02365587
0.20724669	0.00464678	0.0382657	0.60506427	-0.2021042	0.00580122
0.22935294	0.00168739	0.12896072	0.08020819	-0.345434	1.47E-06
0.08177892	0.26845433	-0.0560614	0.44848334	0.21846433	0.00281299
-0.2712913	0.00018753	-0.1979522	0.00691371	0.00624498	0.93276546
-0.1408371	0.05585702	-0.2117888	0.00380348	0.218198	0.00284753
-0.0597395	0.41922914	-0.1116306	0.13033518	0.14638486	0.04678095
-0.192811	0.00855211	-0.2245197	0.00212329	0.1237069	0.09341709
-0.0799606	0.27927766	-0.1247437	0.09068163	0.17842594	0.01510142
-0.2253729	0.00203958	-0.3030256	2.76E-05	0.16323812	0.02640906
-0.1132355	0.12486487	-0.1117772	0.12982784	0.22665031	0.00191986
-0.0594805	0.42125185	-0.0952139	0.19732782	0.0108905	0.88303161
0.07951338	0.28198404	-0.0202353	0.78455212	0.21865464	0.00278855
0.16879304	0.02163145	-0.0080481	0.91342009	-0.2075647	0.00458269
0.16875732	0.02165962	0.03165117	0.66887554	0.26875137	0.00021652
-0.0529519	0.47408762	-0.2107411	0.00398472	0.21920533	0.00271888
0.14879463	0.04324096	0.08137057	0.2708597	-0.0184835	0.80280385
-0.0845369	0.25258958	-0.0819523	0.26743753	0.3184466	9.98E-06
0.05634143	0.44621662	-0.0607994	0.41100998	0.1520777	0.03878168
0.00573545	0.93823973	-0.0667727	0.36649447	0.191669	0.00895971
0.35158274	9.24E-07	0.28648843	7.70E-05	-0.157045	0.0327749
-0.0670738	0.3643326	-0.1482236	0.04405885	0.13263407	0.0719
0.14626318	0.04696594	0.1458898	0.04753739	-0.2964496	4.18E-05
-0.1199881	0.10377192	-0.1144478	0.12085168	0.04137751	0.57601158
-0.3426847	1.80E-06	-0.1958757	0.00753838	-0.0543792	0.46223688
0.14603095	0.04732068	0.09401155	0.20307146	0.25406325	0.00048365
0.05903414	0.42475139	-0.0418132	0.5719967	0.22870418	0.00174072
-0.3638606	3.56E-07	-0.300339	3.27E-05	0.29839113	3.70E-05

-0.1265821	0.08598895	-0.0888541	0.22907891	0.10487418	0.15540529
-0.0376999	0.61041557	-0.1092943	0.138626	0.16944723	0.02112125
-0.0527998	0.47535993	-0.1590836	0.03054898	-0.0102395	0.889978
-0.2017061	0.00590048	-0.1427811	0.05252378	-0.4382398	4.41E-10
0.12118282	0.10035105	0.00163443	0.9823842	0.13518447	0.0665537
-0.2764309	0.0001396	-0.0887669	0.22953783	-0.3244008	6.64E-06
-0.0901678	0.22224118	-0.2466305	0.00071378	0.20278949	0.00563385
0.13682832	0.06328316	-0.024657	0.73902152	0.03454938	0.64058805
0.24653556	0.00071728	0.28836327	6.88E-05	-0.1861924	0.011163
-0.0739242	0.3172952	-0.0316443	0.66894363	-0.3024747	2.86E-05
0.03250899	0.66045196	-0.087458	0.23650612	0.10225451	0.16604693
-0.0915841	0.21503345	-0.1320336	0.07320823	0.11170734	0.13006948
-0.3907363	3.83E-08	-0.309526	1.81E-05	0.52743209	1.22E-14
0.00933679	0.89962421	-0.0509613	0.49088722	0.23898616	0.00105238
-0.5681253	3.34E-17	-0.4315011	8.67E-10	0.31146912	1.59E-05
-0.0839916	0.25567351	-0.1153613	0.11789439	-0.1561459	0.0337995
0.10014467	0.17500543	-0.147423	0.04522739	-0.4027638	1.32E-08
-0.3820091	8.07E-08	-0.328412	5.02E-06	0.36482471	3.30E-07
0.15676378	0.03309251	0.00912576	0.90188138	0.19425834	0.00805921
-0.3191043	9.55E-06	-0.2413802	0.00093311	0.526721	1.35E-14
0.2382389	0.00109238	0.08816961	0.23269949	0.25759405	0.00040037
-0.2205133	0.0025597	-0.2332972	0.00139398	0.05108966	0.48979443
-0.0394375	0.59404786	-0.0725687	0.32627425	-0.1805185	0.01393592
0.63674943	2.00E-22	0.36563548	3.09E-07	-0.1823812	0.01296553
-0.1312421	0.07496204	-0.1674363	0.02272359	0.34687875	1.32E-06
-0.0750314	0.31008135	-0.1163909	0.11462902	0.24200598	0.00090405
0.44614449	1.96E-10	0.26014404	0.00034872	0.24996283	0.00060035
-0.4008414	1.57E-08	-0.2547376	0.00046661	0.00188567	0.97967688
-0.0909709	0.21813332	0.01107898	0.88102206	0.02023138	0.78459234
0.37838266	1.09E-07	0.13965991	0.05795902	-0.3259394	5.97E-06
-0.1261315	0.08712082	-0.2176388	0.00292131	0.1684394	0.02191166
0.09912001	0.17948314	0.12183685	0.09851644	-0.0614854	0.40574147
-0.2329182	0.00141999	-0.1139834	0.12237705	-0.1892096	0.00989639
0.04064485	0.58279284	-0.0729758	0.32356079	0.2722186	0.00017788
0.44874354	1.49E-10	0.29853368	3.67E-05	-0.2340093	0.00134627
0.02970134	0.6881755	0.02048513	0.78195788	0.03567176	0.62976808
-0.3975753	2.10E-08	-0.3467514	1.33E-06	0.44144247	3.18E-10
-0.3088188	1.89E-05	-0.1287247	0.08076748	-0.150242	0.04122468
-0.050435	0.49538117	-0.270901	0.00019174	0.25146515	0.00055487
0.02738105	0.71140657	-0.140985	0.05559733	0.27449265	0.00015615
0.11969286	0.10463134	0.03584767	0.62807927	-0.0780463	0.29098574
-0.0778988	0.29190151	-0.1670779	0.0230199	0.18239933	0.01295638
-0.3250037	6.37E-06	-0.3088148	1.90E-05	0.42749542	1.29E-09
-0.3024125	2.87E-05	-0.2716196	0.00018406	0.49022922	1.41E-12
-0.1132871	0.12469179	-0.1866651	0.01095558	0.11278347	0.12638728
0.19348213	0.00832032	0.06096437	0.40973958	0.27747616	0.00013138

0.14519937	0.04860933	0.01695829	0.81878276	0.08818326	0.2326269
-0.026498	0.72031993	-0.1645177	0.02523501	0.34770698	1.24E-06
0.04250675	0.56563265	-0.0492602	0.5054912	0.26230242	0.0003099
0.29942275	3.47E-05	0.08882634	0.22922473	-0.3299951	4.49E-06
-0.251303	0.00055962	-0.0845432	0.25255391	-0.3108703	1.66E-05
0.06158811	0.40495639	0.05190594	0.48287557	0.20466838	0.00519692
-0.0744021	0.31416777	0.12014281	0.10332384	-0.0839621	0.25584159
-0.0214624	0.77183512	-0.0663933	0.36922983	-0.5149847	6.40E-14
0.0392901	0.59542844	0.08163844	0.26928017	0.0230655	0.75531423
-0.276415	0.00013973	-0.2976015	3.89E-05	0.17838837	0.0151231
0.15790666	0.03181781	0.07472082	0.31209377	-0.2889031	6.66E-05
-0.2483775	0.00065206	-0.2824368	9.81E-05	0.44891007	1.47E-10
-0.1571458	0.03266166	-0.0820179	0.2670534	-0.2529807	0.00051224
0.00190226	0.97949809	-0.0159701	0.82917659	0.05726604	0.43877781
0.18133134	0.01350492	0.14217379	0.05354693	0.27933035	0.00011789
0.09169889	0.21445676	0.01143629	0.87721464	0.39076054	3.82E-08
-0.0004629	0.99501062	-0.0461085	0.53313687	-0.1376563	0.0616865
0.19695825	0.00720672	-0.0364769	0.62205446	-0.2781423	0.00012637
0.21759632	0.00292698	-0.0319394	0.666041	0.19247489	0.00867033
-0.1260908	0.08722359	0.01236795	0.86730045	-0.1286423	0.08096345
-0.0286244	0.69892313	-0.0839628	0.25583726	-0.121722	0.09883655
-0.0082521	0.91123456	0.04678876	0.52710656	-0.1182069	0.10904191
0.07893254	0.28552537	-0.006799	0.92681659	0.16051176	0.02906748
0.06743955	0.3617167	-0.0402893	0.58609733	0.15980117	0.02979677
-0.0739097	0.31739016	-0.124065	0.09246493	0.24869404	0.00064142
0.57330033	1.48E-17	0.28880476	6.70E-05	-0.2931304	5.14E-05
0.2256696	0.00201118	0.08714236	0.23820875	0.30696159	2.14E-05
-0.1456178	0.04795737	-0.162561	0.02704907	0.23275094	0.00143162
-0.009054	0.90264925	-0.1030424	0.16279071	0.27827971	0.00012536
0.18882983	0.0100485	0.34266285	1.80E-06	-0.3683008	2.50E-07
-0.0215273	0.7711647	-0.0584445	0.42940013	0.18649776	0.01102861
0.06689285	0.36563062	0.04211486	0.56922441	-0.3243374	6.67E-06
0.27878109	0.00012174	0.09280886	0.20893666	-0.2679264	0.00022679
0.15007938	0.04144725	-0.0045803	0.95066059	0.17671797	0.01611493
0.2009326	0.00609768	0.15477961	0.03540851	-0.240942	0.00095397
0.06425028	0.38491646	-0.0031843	0.96568795	0.2122372	0.00372821
-0.0441664	0.55054216	-0.1201673	0.10325312	0.25451663	0.00047213
0.32993381	4.51E-06	0.16164243	0.02793848	-0.289344	6.48E-05
0.15299652	0.03760543	0.02965746	0.68861219	0.26005166	0.00035048
0.346525	1.35E-06	0.02267004	0.75937977	0.3076508	2.04E-05
-0.398539	1.93E-08	-0.282108	0.00010008	-0.266495	0.00024571
-0.0779268	0.29172758	0.04375505	0.55426412	0.20302255	0.00557792
-0.1181306	0.10927252	-0.1799734	0.01423168	0.24950761	0.0006148
-0.1797441	0.01435773	-0.0340972	0.64496897	0.16373366	0.02594895
-0.388056	4.82E-08	-0.3321012	3.87E-06	0.2231943	0.00225954
-0.157169	0.03263571	-0.2941854	4.81E-05	0.30754655	2.06E-05

-0.0582045	0.43130066	-0.13294	0.07124081	0.11332169	0.12457608
-0.1131975	0.12499216	-0.106368	0.14957167	0.52301404	2.22E-14
-0.190942	0.00922807	-0.071996	0.33011676	0.32657943	5.71E-06
-0.0300764	0.68444686	-0.0867301	0.24044504	0.08365035	0.25761702
0.16551494	0.02435133	0.1723136	0.01900647	-0.438843	4.15E-10
-0.0882288	0.23238463	-0.0754063	0.30766299	-0.0879159	0.23405181
-0.4636868	2.99E-11	-0.3046048	2.49E-05	0.19847753	0.00676316
-0.1016327	0.16865073	-0.0937769	0.20420617	0.13724862	0.06246844
-0.1572503	0.03254468	-0.2512669	0.00056069	0.16621	0.02375128
0.00644868	0.93057777	-0.1390454	0.05908186	0.21457476	0.00335713
0.68418053	7.01E-27	0.45789978	5.63E-11	-0.2730583	0.00016955
-0.0991701	0.17926229	-0.1298776	0.07806527	0.20343269	0.00548071
-0.38609	5.71E-08	-0.2606736	0.0003388	0.10661961	0.14860541
-0.1062131	0.1501687	-0.0911051	0.21745214	0.05604724	0.4485984
-0.3015021	3.04E-05	-0.3839618	6.84E-08	0.14920059	0.04266728
0.02785153	0.70667371	-0.0600063	0.41715087	-0.0708158	0.33812607
0.0094201	0.89873333	-0.0343334	0.64267924	0.39610288	2.39E-08
-0.069011	0.35061196	-0.0734982	0.32009965	0.16955612	0.02103734
-0.1085708	0.14127324	-0.0219525	0.7667734	-0.2102385	0.00407439
0.03944044	0.5940201	-0.0690276	0.35049603	0.20666853	0.00476538
-0.1696488	0.02096612	-0.1260834	0.08724223	-0.2989429	3.57E-05
-0.2577057	0.00039797	-0.2950303	4.57E-05	0.08932423	0.22661493
-0.310248	1.73E-05	-0.2116789	0.00382213	-0.2138268	0.00347206
-0.2413703	0.00093358	-0.1638331	0.02585748	-0.1620422	0.02754842
-0.0230116	0.75586786	-0.1618218	0.02776289	0.27858037	0.00012318
-0.0909845	0.21806428	0.22437879	0.00213741	-0.4518663	1.07E-10
0.13213005	0.07299678	0.03050109	0.68023409	0.30919742	1.85E-05
0.00507562	0.94533311	-0.0820505	0.26686256	0.21231902	0.00371462
0.13660615	0.06371733	0.07550372	0.30703659	-0.377059	1.22E-07
0.17362987	0.01809852	0.03814447	0.60620925	-0.2147736	0.00332716
-0.2528696	0.00051525	-0.1993764	0.00651234	0.16696363	0.02311507
-0.1668122	0.02324173	-0.2870386	7.45E-05	0.20851534	0.00439578
0.11564953	0.11697297	0.09015842	0.22228951	-0.20179	0.00587943
-0.2725477	0.00017457	-0.1911022	0.00916834	0.20607283	0.00489041
0.20397155	0.00535529	0.08695237	0.2392376	-0.3080182	2.00E-05
0.42198066	2.20E-09	0.27161679	0.00018409	-0.2584686	0.00038191
0.06436705	0.3840514	0.25935395	0.00036402	-0.1719085	0.01929376
-0.0068097	0.92670175	0.00713789	0.92317987	-0.2794621	0.00011698
-0.1713575	0.01969052	-0.2262034	0.001961	-0.4021427	1.39E-08
-0.1852886	0.01156919	-0.1729207	0.01858289	0.34305121	1.75E-06
-0.2893588	6.47E-05	-0.2828434	9.58E-05	0.2539726	0.00048599
-0.0368544	0.61845214	-0.0772594	0.29589282	-0.36719	2.73E-07
0.07788058	0.29201475	-0.0091203	0.90193946	-0.3904558	3.92E-08
-0.3172957	1.08E-05	-0.2141982	0.00341455	-0.2026333	0.00567161
0.40950766	7.12E-09	0.07721646	0.29616183	-0.1842215	0.01206553
0.06867602	0.35296114	-0.0070785	0.9238176	-0.4182986	3.13E-09

-0.1427853	0.05251685	-0.162301	0.02729833	0.2139415	0.00345421
-0.1195954	0.10491634	-0.0888729	0.22898002	0.17882144	0.01487483
0.02638941	0.7214182	-0.0693507	0.34824028	0.26129873	0.00032742
0.13538293	0.06615165	-0.0193687	0.79356662	-0.1834305	0.01244539
0.59729235	2.86E-19	0.49247489	1.08E-12	-0.0948309	0.19914431
-0.1342727	0.06842635	-0.0280807	0.70437264	-0.3634564	3.68E-07
0.03685517	0.6184445	0.03519112	0.63439212	-0.4419089	3.03E-10
0.19427785	0.00805274	-0.0012548	0.98647542	-0.198212	0.00683887
0.18191945	0.01320039	0.21112228	0.00391789	-0.2305931	0.00158957
-0.419177	2.88E-09	-0.2229991	0.00228026	0.11233193	0.1279224
0.0659429	0.37249351	0.16575982	0.02413846	0.28237847	9.85E-05
0.27069429	0.000194	0.07552208	0.30691863	0.12605769	0.08730731
0.07714691	0.2965984	0.00069462	0.9925129	0.22767864	0.00182817
-0.2805061	0.00011002	-0.223565	0.00222066	0.11726329	0.11191778
-0.005269	0.94325419	-0.0762131	0.3025008	0.22377442	0.00219895
0.06505504	0.37897893	-0.0005664	0.99389469	0.16646659	0.02353299
-0.2161379	0.00312794	0.01111815	0.88060455	0.03588491	0.62772201
-0.0426788	0.56405912	-0.1463373	0.04685321	0.18414009	0.01210414
-0.0489321	0.50833386	-0.1769638	0.0159655	0.23667398	0.0011807
-0.1851514	0.01163198	-0.2942303	4.80E-05	0.45375705	8.78E-11
-0.259067	0.00036973	-0.2504278	0.00058592	-0.3902876	3.98E-08
-0.3417716	1.92E-06	-0.0642755	0.38472957	-0.2644313	0.00027558
0.11446483	0.12079598	0.08339987	0.25904981	-0.2076358	0.00456846
0.20818536	0.00445987	0.02497654	0.7357643	0.216867	0.00302597
0.52627905	1.43E-14	0.14893134	0.04304705	-0.3589429	5.24E-07
-0.1823322	0.01299027	-0.2729237	0.00017086	0.24319882	0.00085095
0.10829259	0.14230162	-0.0960726	0.19329848	0.20132909	0.00599587
-0.0986803	0.18143037	-0.1198947	0.10404313	-0.3641537	3.48E-07
0.37928417	1.01E-07	0.27220411	0.00017803	-0.1797825	0.01433654
-0.0373074	0.61414098	-0.084878	0.25067361	0.20519254	0.00508057
-0.2892925	6.50E-05	-0.2504381	0.00058561	-0.1045388	0.15673834
0.40081072	1.57E-08	0.20832084	0.00443346	-0.1359078	0.06509802
0.22521642	0.00205471	0.28056907	0.00010961	-0.0047898	0.94840681
0.22818944	0.00178413	0.10637718	0.14953616	-0.4914763	1.22E-12
0.22754405	0.00183994	0.0065805	0.92916237	-0.1592518	0.03037125
-0.1300147	0.07774883	-0.1493534	0.04245295	0.20659873	0.00477988
-0.0609035	0.4102079	0.24997897	0.00059985	0.1807696	0.01380152
-0.2243444	0.00214087	-0.3687198	2.41E-07	0.2013935	0.00597947
0.19372497	0.00823783	0.22941962	0.00168199	0.24738083	0.00068664
0.33066439	4.29E-06	0.15254993	0.03817333	-0.1914365	0.00904478
-0.015787	0.83110501	-0.0816058	0.26947249	0.18272459	0.01279325
-0.2705319	0.00019579	-0.2775362	0.00013092	0.1085556	0.1413294
0.30953061	1.81E-05	0.25694586	0.00041459	0.28759353	7.21E-05
-0.0750774	0.30978364	0.29874762	3.62E-05	-0.373606	1.62E-07
-0.155019	0.03512197	-0.2336512	0.00137007	0.13963661	0.05800127
-0.149355	0.04245082	-0.277122	0.00013411	0.39120437	3.67E-08

-0.0654881	0.37580682	-0.291088	5.83E-05	0.24210762	0.00089941
-0.2126883	0.00365384	0.00844802	0.90913577	-0.1101598	0.13550863
0.02317622	0.75417719	-0.0834666	0.25866773	0.16912204	0.02137355
-0.1643997	0.0253414	-0.1584452	0.03123174	-0.0445306	0.54725733
0.00937009	0.89926806	-0.0653636	0.37671743	-0.3559071	6.63E-07
-0.31807	1.02E-05	-0.1940319	0.00813461	0.31303655	1.43E-05
-0.1282448	0.08191406	-0.1982342	0.00683253	0.13719528	0.06257135
0.06924518	0.34897598	0.29348894	5.02E-05	0.05754936	0.43651264
0.20275646	0.00564182	0.13399202	0.06901151	-0.1945699	0.00795645
-0.1160589	0.11567407	-0.2320091	0.00148422	-0.0160728	0.82809421
-0.0410474	0.57906203	-0.022957	0.75642943	-0.1107795	0.13331022
0.62069058	4.36E-21	0.33466279	3.22E-06	-0.3165471	1.13E-05
-0.1608239	0.028752	-0.2358627	0.00122901	0.4080036	8.18E-09
-0.101569	0.16891951	0.05652739	0.4447148	-0.4382119	4.42E-10
-0.1548411	0.03533472	-0.0459067	0.53493246	-0.3686154	2.43E-07
0.22739516	0.00185304	0.07969961	0.28085488	-0.1994921	0.00648067
-0.3057534	2.31E-05	-0.228286	0.00177591	0.06314701	0.39314745
-0.2592405	0.00036627	-0.2167656	0.00303997	0.08439363	0.25339747
-0.0036062	0.96114475	-0.0429133	0.5619183	-0.0984928	0.18226516
0.60484074	7.69E-20	0.32488517	6.42E-06	-0.2556498	0.00044443
0.1870972	0.01076895	0.00571217	0.93848993	0.08371083	0.25727191
0.0002358	0.99745836	-0.0304037	0.68119898	0.12910818	0.07986038
0.05973023	0.41930133	-0.1380755	0.06089071	0.0644686	0.38330013
-0.2700739	0.00020094	-0.2874125	7.29E-05	0.0265708	0.71958326
0.52656852	1.38E-14	0.32302658	7.30E-06	-0.2643598	0.00027667
-0.1732418	0.01836222	-0.0946403	0.20005302	-0.0473034	0.52256709
-0.0119436	0.87181381	0.06288311	0.39513186	-0.1478537	0.04459546
-0.1683159	0.02201026	-0.2189934	0.0027455	0.15805234	0.03165837
-0.1166901	0.11369349	-0.0369649	0.61739898	-0.0625577	0.39758705
0.18796695	0.0104018	0.0323285	0.66222085	0.22205015	0.00238349
-0.056139	0.447855	-0.1315238	0.07433398	0.23978304	0.00101121
-0.024907	0.73647254	-0.0828955	0.2619517	0.27739182	0.00013202
0.41977189	2.72E-09	0.20220855	0.00577545	-0.3461328	1.39E-06
0.00908686	0.90229755	0.08586287	0.24519765	-0.3808048	8.93E-08
-0.2880038	7.03E-05	-0.0958668	0.19425859	-0.2483985	0.00065135
-0.0398487	0.59020314	0.12507318	0.08982598	-0.1944163	0.00800697
-0.0282179	0.70299597	-0.119321	0.10572179	-0.3517302	9.14E-07
-0.015203	0.83726469	-0.0296577	0.68860931	-0.0888028	0.22934844
-0.011266	0.87902891	-0.046207	0.53226152	-0.1853151	0.01155712
0.09059467	0.22005104	-0.044041	0.55167546	0.18269203	0.0128095
-0.0495917	0.50262812	-0.0658718	0.37301032	0.24997371	0.00060001
-0.0210187	0.77642689	-0.1043167	0.15762582	0.1826147	0.01284816
-0.0633803	0.39139797	0.01350611	0.85521693	0.27848872	0.00012384
0.21480295	0.00332275	0.38903073	4.43E-08	-0.2091903	0.00426728
-0.2367869	0.00117411	0.00083372	0.99101366	-0.3902302	4.00E-08
-0.4315646	8.62E-10	-0.303415	2.69E-05	0.26253085	0.00030604

-0.0651479	0.37829726	-0.1353916	0.06613405	0.15247102	0.03827442
-0.0309035	0.67625116	0.01774474	0.81053367	-0.2712862	0.00018759
-0.2646589	0.00027213	-0.0681058	0.35698207	-0.4239298	1.82E-09
0.02657274	0.7195636	-0.1385532	0.05999426	-0.0987078	0.18130811
0.17370949	0.01804484	0.16407278	0.02563805	0.30708441	2.12E-05
-0.2876292	7.19E-05	-0.2434778	0.00083896	0.33979511	2.22E-06
-0.0740546	0.31643957	-0.1183278	0.1086778	0.22000648	0.00262035
-0.0378352	0.60913475	-0.1185846	0.10790731	0.11140143	0.13113116
-0.0072542	0.92193212	-0.0081252	0.91259425	0.25149198	0.00055409
0.04812068	0.51539975	0.01389077	0.85114059	0.17532676	0.01698379
-0.1956427	0.00761153	0.05891168	0.42571439	0.22661421	0.00192315
0.14707925	0.04573691	-0.0804288	0.2764629	0.22616749	0.00196434
-0.3664705	2.89E-07	-0.3156697	1.20E-05	0.17755862	0.01560891
-0.0584618	0.429263	0.01567312	0.83230547	0.49049384	1.37E-12
-0.0392764	0.59555696	-0.1257293	0.088141	0.22862299	0.0017475
-0.0433658	0.55779708	-0.1320012	0.07327933	0.28878041	6.71E-05
-0.2123328	0.00371233	-0.1982484	0.00682845	0.23960858	0.00102009
-0.3423719	1.84E-06	-0.39209	3.40E-08	0.36765588	2.63E-07
-0.0441459	0.55072789	-0.025883	0.72654931	0.20983009	0.00414859
-0.2782394	0.00012566	-0.26289	0.00030005	0.1037601	0.15986637
0.42621076	1.46E-09	0.04326759	0.55869023	0.31488213	1.27E-05
0.10416188	0.15824658	-0.1016558	0.16855347	-0.0091184	0.90195982
0.02551628	0.73027271	0.0240283	0.7454445	-0.2405619	0.0009724
-0.0505456	0.49443484	-0.0959026	0.19409144	0.18778761	0.01047659
-0.1592992	0.03032135	-0.1797055	0.01437904	0.35305071	8.26E-07
-0.0541826	0.46385992	-0.1515504	0.03947058	-0.2391807	0.00104219
0.1180728	0.10944716	0.06505057	0.37901174	-0.4340851	6.70E-10
0.08288118	0.26203425	0.02534495	0.73201449	0.08506129	0.24964806
0.0576878	0.43540818	0.08455475	0.25248909	-0.2648719	0.00026893
0.42086766	2.45E-09	0.24805047	0.00066323	-0.2606598	0.00033905
-0.2188059	0.00276926	-0.1829379	0.01268723	0.07244002	0.32713514
-0.1338911	0.06922294	-0.1684122	0.02193331	0.10568565	0.15221535
0.31380037	1.36E-05	0.25424139	0.0004791	-0.1585103	0.03116152
0.24654958	0.00071676	0.10258645	0.16466919	-0.1925331	0.00864974
-0.2425578	0.00087912	-0.1895618	0.00975716	-0.2455095	0.00075617
-0.3104739	1.70E-05	-0.1868989	0.01085425	-0.2326178	0.00144093
-0.2022374	0.00576835	-0.2436655	0.00083097	0.17472457	0.01737235
-0.2618124	0.00031834	-0.139611	0.05804773	-0.1303238	0.07703923
0.05187892	0.48310375	0.05198446	0.48221273	-0.5627653	7.61E-17
0.14159902	0.05453036	0.02890468	0.69612025	0.35151006	9.29E-07
-0.2977282	3.86E-05	-0.2993678	3.48E-05	0.25367268	0.00049379
-0.1010812	0.17098564	-0.1473809	0.04528952	0.09739364	0.18721736
0.12586192	0.0878036	0.00680111	0.926794	0.10483096	0.15557658
-0.1358326	0.06524806	-0.0966482	0.19063142	0.20079205	0.00613413
-0.2224249	0.00234223	-0.2350961	0.00127633	-0.2384161	0.00108277
-0.015652	0.8325278	-0.1567576	0.03309947	0.36161847	4.25E-07

-0.0444212	0.54824309	-0.0873327	0.23718103	0.18416798	0.0120909
-0.040202	0.58690934	-0.0131753	0.85872578	-0.2693011	0.00020991
-0.2197714	0.00264892	-0.125761	0.0880604	0.01937561	0.7934948
-0.0758951	0.30452905	-0.0906676	0.21967849	-0.3745022	1.51E-07
-0.1745797	0.01746695	-0.2781897	0.00012602	0.17018943	0.02055502
-0.2670294	0.00023848	-0.0683863	0.35500041	-0.1352204	0.06648086
0.24401955	0.0008161	0.00426656	0.95403666	0.14675186	0.04622671
-0.072152	0.32906708	-0.0828439	0.26224978	-0.2150399	0.00328739
-0.329229	4.74E-06	-0.2848657	8.49E-05	0.3793491	1.01E-07
-0.1311622	0.07514086	-0.0855581	0.24688307	-0.2039665	0.00535646
0.16668898	0.02334521	-0.0399987	0.58880403	0.20808242	0.00448003
-0.0675237	0.36111642	-0.1174015	0.11149289	0.15904916	0.03058552
-0.1430976	0.05199714	-0.2264343	0.00193964	0.19224239	0.00875295
-0.188974	0.00999053	-0.1565335	0.03335451	0.55905098	1.34E-16
0.25815846	0.00038836	0.0519625	0.48239807	0.03977386	0.59090226
-0.0595096	0.4210246	0.01772495	0.81074106	-0.2604788	0.00034242
-0.2194336	0.00269047	-0.2051943	0.00508019	0.16100087	0.02857441
-0.1933274	0.00837326	-0.2951456	4.53E-05	-0.1715889	0.019523
0.06616328	0.37089439	-0.0342735	0.64325984	0.12234246	0.09711645
0.56634571	4.40E-17	0.38582047	5.84E-08	-0.3707168	2.05E-07
-0.2237588	0.00220057	-0.147929	0.04448585	0.24609761	0.00073365
0.03294073	0.65622837	-0.0492981	0.5051639	0.0436173	0.55551317
0.20884735	0.00433214	0.04365972	0.55512837	-0.054967	0.45740499
-0.0490444	0.5073601	-0.0278154	0.70703674	0.35145243	9.33E-07
-0.2007057	0.00615663	-0.094647	0.20002122	-0.3073308	2.09E-05
-0.1622354	0.0273615	-0.0965711	0.19098728	0.01113409	0.88043472
-0.237426	0.00113747	-0.1146622	0.12015245	-0.0115366	0.87614662
0.11402004	0.12225607	0.16485291	0.02493494	-0.1330996	0.0708989
-0.0514098	0.48707422	0.14726435	0.04546192	-0.5320852	6.49E-15
-0.1288632	0.08043881	-0.0352673	0.63365803	-0.1198476	0.10418011
0.25092132	0.00057096	0.13685354	0.06323403	-0.2934411	5.04E-05
0.2038268	0.00538873	-0.0213149	0.7733603	0.10391693	0.15923266
0.17255698	0.01883567	0.03085311	0.67674966	0.11450833	0.12065388
0.14920174	0.04266566	-0.0833033	0.25960384	0.23807516	0.00110133
0.00333809	0.96403135	-0.0310185	0.67511523	0.22682822	0.0019037
0.06400571	0.38673196	0.02024742	0.78442573	0.52147329	2.73E-14
0.30256023	2.84E-05	-0.0565354	0.44465044	0.05842118	0.42958436
0.65760319	2.76E-24	0.37069028	2.06E-07	-0.2812418	0.00010534
0.01676072	0.82085825	0.00856032	0.90793315	-0.1995033	0.00647762
-0.3438729	1.65E-06	-0.292347	5.39E-05	0.01024653	0.88990305
-0.2343167	0.00132614	-0.0379398	0.60814452	0.0676397	0.36029037
-0.1849425	0.01172819	-0.1715634	0.01954143	-0.3109061	1.65E-05
0.38314356	7.33E-08	0.54375575	1.26E-15	-0.2457292	0.00074768
-0.0707829	0.33835163	-0.0344216	0.64182433	-0.160701	0.02887588
-0.1098086	0.136767	-0.1494098	0.04237419	0.1003309	0.17420058
0.17385532	0.01794686	0.13777009	0.06146959	0.32211704	7.77E-06

0.03930061	0.59532994	-0.0082463	0.91129651	0.13689218	0.06315881
-0.1766491	0.01615701	-0.0811567	0.27212541	0.37821723	1.11E-07
0.02882469	0.69691975	0.15839692	0.03128394	0.20915543	0.00427383
-0.1240372	0.09253854	-0.1746914	0.01739399	0.2901125	6.18E-05
0.12027766	0.10293451	0.03360317	0.64976907	0.11917842	0.10614213
0.50817778	1.54E-13	0.41046222	6.52E-09	-0.1963587	0.00738877
-0.3318163	3.95E-06	-0.2122269	0.00372991	0.13926697	0.05867502
-0.0362356	0.62436193	-0.122075	0.09785515	0.18921977	0.00989236
0.15785492	0.03187461	-0.0432301	0.55903124	-0.2769863	0.00013517
0.02276088	0.75844532	-0.0290757	0.69441154	-0.1832302	0.01254325
0.00920264	0.90105905	-0.0858645	0.24518881	0.25203424	0.00053849
0.07542623	0.30753473	-0.0155035	0.83409361	0.01655851	0.82298381
-0.0174385	0.81374319	-0.0188995	0.79845911	-0.2145769	0.00335681
0.0855663	0.24683772	0.03105245	0.67477955	-0.2263065	0.00195144
-0.0499184	0.49981407	-0.077558	0.29402445	-0.2888978	6.66E-05
-0.1439358	0.05062324	-0.1323151	0.07259257	0.40365415	1.22E-08
0.20741434	0.00461289	-0.0026556	0.97138171	0.22525899	0.00205058
-0.0124808	0.86610061	-0.0146334	0.84328228	0.42329296	1.94E-09
0.16219329	0.02740215	0.01187608	0.8725321	-0.0265342	0.71995302
-0.1143561	0.12115172	-0.0033624	0.96376923	-0.1704642	0.02034878
-0.3742861	1.53E-07	-0.2578648	0.00039457	-0.4841295	2.92E-12
-0.1940998	0.00811193	-0.1879771	0.0103976	-0.0349546	0.63667257
-0.1031302	0.16243064	-0.148645	0.04345398	0.06253161	0.39778428
-0.1100781	0.13580067	-0.0126181	0.86464168	-0.0204232	0.78260079
-0.2827873	9.61E-05	-0.1083975	0.1419133	0.05530239	0.45466044
0.15851128	0.03116051	0.12068271	0.10177201	-0.0327323	0.65826637
-0.044079	0.55133221	-0.1433829	0.05152606	0.25947706	0.0003616
-0.0723716	0.32759351	-0.1560858	0.03386902	0.21256942	0.00367331
-0.2133936	0.00354025	-0.2309669	0.00156111	0.31076852	1.67E-05
-0.0700543	0.34335989	-0.1723007	0.01901555	0.07697517	0.29767821
0.05225103	0.47996639	-0.0399139	0.58959525	-0.1588781	0.03076736
0.11302141	0.1255841	0.04502617	0.54280291	-0.4282264	1.20E-09
-0.2734198	0.00016607	-0.2600055	0.00035136	0.06131567	0.40704154
0.14092619	0.05570051	-0.192627	0.00861667	0.2737402	0.00016305
-0.0767628	0.29901719	-0.1730989	0.01846017	0.24764727	0.00067723
-0.2908651	5.91E-05	-0.1660641	0.02387615	-0.1582146	0.03148161
-0.0329171	0.65645931	-0.1708097	0.02009191	-0.4941185	8.83E-13
-0.3365364	2.82E-06	-0.260141	0.00034878	0.14285808	0.05239531
0.00382002	0.9588427	-0.1537622	0.03664834	0.15170989	0.03926117
0.39235617	3.32E-08	0.18446687	0.01194977	-0.3820443	8.05E-08
0.06153141	0.4053898	-0.0732974	0.3214272	-0.0002059	0.99778101
0.48057146	4.42E-12	0.27694792	0.00013548	-0.0021988	0.97630315
0.2461688	0.00073096	0.13495056	0.0670301	-0.1664007	0.02358888
-0.0560144	0.44886473	0.00246622	0.97342187	-0.2861052	7.88E-05
-0.2633706	0.00029221	-0.2604212	0.00034349	0.01147077	0.87684738
-0.2295494	0.00167154	-0.2790637	0.00011974	0.14820819	0.04408109

-0.345873	1.42E-06	-0.1433599	0.05156393	-0.380189	9.40E-08
-0.4428593	2.75E-10	-0.3306417	4.29E-06	0.01539034	0.83528771
-0.1691838	0.02132547	-0.1607772	0.02879903	0.3507862	9.81E-07
0.03237674	0.66174785	0.1212818	0.10007167	-0.0430381	0.56077992
-0.3581628	5.57E-07	-0.2896493	6.36E-05	-0.4578095	5.68E-11
-0.2248519	0.00209033	-0.1129146	0.12594417	-0.1769905	0.01594935
-0.1078613	0.14390703	-0.1631477	0.02649374	0.01974358	0.78966349
0.2252517	0.00205129	-0.1196361	0.10479722	0.03314495	0.65423436
0.37357103	1.63E-07	0.24922019	0.00062409	-0.1720769	0.01917384
-0.195912	0.00752704	-0.2255872	0.00201904	0.12971832	0.07843416
0.02068517	0.77988288	0.08578967	0.24560172	-0.106477	0.14915234
-0.2622667	0.00031051	-0.0940696	0.20279159	-0.2389602	0.00105374
0.09871409	0.18128011	-0.0296146	0.68903918	0.15744675	0.03232565
-0.0256461	0.72895402	-0.0789491	0.28542415	-0.4732648	1.02E-11
-0.1601343	0.02945297	-0.2547101	0.00046729	0.14153941	0.05463321
0.04883098	0.50921191	0.12719717	0.08446308	-0.0409255	0.58019097
-0.0276647	0.70855172	-0.1755204	0.01686044	0.10817671	0.14273159
-0.1556725	0.03434988	-0.1348206	0.067296	-0.3498006	1.06E-06
-0.0948881	0.19887242	-0.1777292	0.01550793	0.21657679	0.0030662
-0.1337285	0.06956443	-0.1827404	0.01278536	0.19993158	0.00636161
-0.183425	0.01244806	-0.1461367	0.04715881	0.05412262	0.46435522
-0.1838637	0.01223606	-0.2458784	0.00074197	0.12373187	0.09335044
-0.3168791	1.11E-05	-0.3727264	1.74E-07	0.25922665	0.00036654
-0.328832	4.88E-06	-0.2887092	6.74E-05	0.2804227	0.00011056
-0.1118504	0.1295752	-0.1748632	0.0172822	0.17787122	0.01542431
0.40924088	7.30E-09	0.20129101	0.00600558	-0.3301056	4.46E-06
0.08347013	0.25864739	-0.2103927	0.00404669	0.23372579	0.00136508
0.41148142	5.93E-09	0.16201293	0.02757678	0.00413712	0.95542962
-0.3567232	6.23E-07	-0.2035446	0.00545444	-0.0850327	0.2498079
0.00859765	0.90753349	0.14645364	0.04667667	0.18786582	0.01044391
-0.0567252	0.4431204	-0.021067	0.77592684	-0.26536	0.00026174
-0.0754422	0.30743181	0.12429692	0.09185253	-0.2706138	0.00019489
-0.2828973	9.55E-05	-0.0546628	0.4599023	-0.196654	0.00729858
-0.1751239	0.0171138	-0.0841359	0.25485494	-0.2088729	0.00432727
-0.1088898	0.14010145	-0.1607398	0.02883673	0.18890335	0.0100189
-0.0165097	0.82349725	-0.0758283	0.30495573	0.09979455	0.17652606
-0.1829184	0.01269693	-0.0831988	0.26020406	-0.1466996	0.04630524
0.01950444	0.79215289	0.00550572	0.94070892	0.48769515	1.91E-12
-0.1580193	0.03169451	-0.1808679	0.01374922	0.2056519	0.00498053
-0.1332426	0.07059367	-0.0385475	0.60240655	-0.2413739	0.00093341
0.08154072	0.26985563	0.04844756	0.51254731	0.06509562	0.37868101
0.03937562	0.59462708	-0.0173795	0.81436188	0.31896503	9.64E-06
-0.0584663	0.42922765	-0.1786485	0.01497355	0.0654811	0.37585818
-0.0049995	0.94615183	-0.151372	0.03970605	0.20365844	0.00542785
-0.2353906	0.00125796	-0.0292707	0.69246633	-0.1312178	0.07501644
-0.2706282	0.00019473	-0.1601608	0.02942574	0.17082959	0.02007724

-0.208275	0.00444238	-0.0682261	0.3561313	-0.1528536	0.03778636
-0.1322246	0.07279006	-0.2444306	0.00079915	0.14285258	0.05240447
-0.209515	0.00420667	-0.1947565	0.00789547	0.61370863	1.57E-20
-0.0628866	0.39510561	-0.0502936	0.4965928	0.22545538	0.00203165
0.02278093	0.75823915	0.07283477	0.32449895	-0.1119646	0.12918191
0.00711979	0.92337409	-0.1420643	0.05373319	0.23583957	0.00123042
0.05683637	0.44222583	0.07227595	0.32823487	-0.1919256	0.00886664
0.23085594	0.00156951	0.07960411	0.28143351	0.2016989	0.00590228
-0.0866184	0.24105389	-0.1225796	0.09646516	0.03789314	0.60858586
0.37982112	9.70E-08	0.10428531	0.15775146	-0.1477961	0.04467959
-0.3515331	9.27E-07	-0.1209039	0.10114159	-0.4326135	7.76E-10
-0.2929782	5.19E-05	-0.2222765	0.00235848	0.16453605	0.0252185
-0.1863279	0.01110319	-0.1728376	0.0186404	-0.0013977	0.98493482
-0.2247771	0.00209771	-0.1265711	0.0860164	-0.3539546	7.71E-07
-0.2321407	0.00147477	-0.1434102	0.05148122	-0.0237135	0.74866706
-0.1291296	0.07980995	-0.2620997	0.00031337	0.17146899	0.01960965
-0.3895927	4.22E-08	-0.2374364	0.00113688	-0.4635583	3.03E-11
0.23357836	0.00137496	0.01015103	0.89092276	0.14565021	0.04790711
0.10363481	0.16037401	0.04284358	0.56255413	-0.2631021	0.00029657
0.15376016	0.03665086	-0.0316792	0.66859978	0.14967809	0.04200067
0.05293735	0.47420901	0.14713936	0.04564745	0.18648328	0.01103495
-0.1797609	0.01434849	0.07199053	0.33015359	-0.2002215	0.00628415
-0.0282734	0.70243943	-0.077987	0.29135411	0.20164177	0.00591666
-0.0928217	0.20887331	-0.2058268	0.00494291	0.11987633	0.10409664
0.05463547	0.46012693	-0.0156901	0.83212627	-0.2013127	0.00600005
-0.2787645	0.00012186	-0.1381206	0.06080565	-0.2392616	0.00103798
-0.1602937	0.02928961	-0.2633421	0.00029267	0.11939388	0.10550731
0.41576051	3.97E-09	0.29868461	3.63E-05	-0.3749278	1.46E-07
-0.1381029	0.06083902	-0.2514605	0.00055501	0.14722672	0.04551771
-0.3064986	2.20E-05	-0.3730044	1.70E-07	0.27437297	0.00015722
-0.1412361	0.05515898	-0.2075415	0.00458732	0.14250013	0.05299513
0.22447185	0.00212808	0.21064561	0.00400161	-0.3216303	8.04E-06
-0.0214327	0.77214276	0.11180967	0.12971582	-0.2871625	7.40E-05
-0.2185039	0.00280789	-0.2637977	0.00028541	-0.2734301	0.00016597
-0.3153739	1.23E-05	-0.218893	0.0027582	-0.0637617	0.38854855
0.04666658	0.52818714	-0.1404134	0.05660616	0.21040094	0.00404521
-0.1950454	0.00780191	-0.0934215	0.20593393	0.21173422	0.00381273
0.15249051	0.03824943	-0.0472127	0.52336598	-0.0390391	0.59778306
-0.08776	0.23488559	-0.1301736	0.07738341	0.16758314	0.02260313
-0.2814412	0.00010411	-0.1874092	0.01063594	0.26245934	0.00030724
-0.0799615	0.27927211	-0.0661332	0.3711126	0.1519708	0.03892053
-0.1194514	0.10533838	-0.185794	0.01134046	0.12606025	0.08730082
-0.2930721	5.16E-05	-0.2188015	0.00276981	0.02961268	0.68905791
-0.3008565	3.17E-05	-0.118664	0.10767007	0.09027078	0.22171147
-0.1142056	0.12164538	-0.0180891	0.80692832	0.01470598	0.84251465
-0.0993096	0.17864813	-0.1820448	0.01313628	0.23896911	0.00105328

0.05561299	0.45212701	0.00564856	0.93917362	0.1421014	0.05366997
-0.2581017	0.00038956	-0.3519179	9.01E-07	0.1200966	0.10345753
0.08390648	0.25615757	0.04489025	0.54402276	-0.3833256	7.22E-08
0.16245776	0.0271478	-0.0227302	0.75876062	0.17899097	0.01477862
0.05176548	0.48406235	0.08727907	0.23747031	0.15250793	0.03822711
-0.0537142	0.46773816	-0.2523648	0.00052918	-0.2151221	0.00327521
0.15829955	0.03138936	0.04509805	0.54215825	0.14735451	0.04532848
0.08742525	0.23668258	0.05863464	0.4278977	0.2238644	0.00218969
0.05542429	0.45366518	-0.039466	0.59378095	0.14281745	0.05246311
-0.1124821	0.12741035	-0.0154728	0.83441736	-0.1699512	0.02073533
-0.191867	0.00888782	-0.1987433	0.00668813	0.24170469	0.00091794
-0.2190102	0.00274338	-0.2137987	0.00347646	0.08990747	0.22358453
0.01098128	0.8820636	-0.0750514	0.30995156	0.20396447	0.00535692
0.03981075	0.59055773	-0.025655	0.72886387	0.24705647	0.00069825
-0.1407981	0.05592561	-0.119506	0.10517803	-0.1568255	0.03302252
-0.2334808	0.00138153	-0.2083177	0.00443406	0.10491921	0.15522695
-0.0565079	0.44487207	-0.0112998	0.87866888	0.20418495	0.00530634
0.02824764	0.70269762	-0.033773	0.64811745	0.14195126	0.05392592
-0.2635519	0.0002893	-0.2581789	0.00038794	0.14257915	0.05286222
-0.2376685	0.00112384	-0.1263096	0.08667196	-0.4002814	1.65E-08
-0.154472	0.03577952	-0.180638	0.01387181	0.09182188	0.21384001
-0.0664521	0.36880509	-0.1040384	0.15874314	-0.1874372	0.01062407
-0.1937435	0.00823157	-0.2371419	0.00115363	0.22952174	0.00167376
-0.2332336	0.00139831	-0.2187837	0.00277207	-0.433925	6.81E-10
-0.1019555	0.16729542	-0.1411111	0.05537691	0.11456033	0.12048423
-0.1788794	0.01484187	-0.1574294	0.0323449	0.1109699	0.13264011
-0.2993033	3.49E-05	-0.28711	7.42E-05	0.43592756	5.57E-10
-0.3372128	2.68E-06	-0.2500188	0.0005986	-0.122365	0.09705427
0.11702974	0.11263863	0.13923076	0.05874136	-0.2683897	0.00022097
-0.2917445	5.60E-05	-0.3033988	2.69E-05	0.39717117	2.18E-08
-0.0072873	0.92157696	-0.1186926	0.1075846	0.15069888	0.04060492
-0.1539516	0.03641485	-0.2620919	0.0003135	0.39572135	2.47E-08
-0.283817	9.04E-05	-0.346953	1.31E-06	0.38257157	7.70E-08
-0.1694865	0.02109095	-0.3003293	3.27E-05	0.27061509	0.00019487
0.1275074	0.08370172	0.1773346	0.01574238	0.02630336	0.72228919
-0.0445225	0.54733045	0.19582421	0.00755449	0.0014782	0.98406781
-0.2099101	0.00413395	-0.2977507	3.85E-05	0.36300579	3.81E-07
-0.0713729	0.33433008	-0.1385797	0.05994476	0.20009986	0.00631654
0.00715688	0.92297609	0.00578587	0.93769793	0.02102561	0.77635495
-0.1485018	0.04365876	-0.1088873	0.14011064	0.26641971	0.00024674
-0.0894984	0.22570688	-0.2065744	0.00478495	0.2346373	0.00130544
-0.0765863	0.300133	0.02117271	0.77483202	-0.3102367	1.73E-05
0.16813538	0.02215505	0.05580738	0.45054554	0.06744209	0.36169861
0.4255309	1.56E-09	0.21620179	0.00311889	-0.0829966	0.26136792
0.11631245	0.11487522	0.04423561	0.54991724	-0.2141508	0.00342184
-0.2034524	0.00547607	-0.2515854	0.00055137	0.18761136	0.01055055

0.2403249	0.00098407	0.07467517	0.3123903	0.26943578	0.00020832
0.35563832	6.77E-07	0.21162535	0.00383125	-0.0983439	0.18293068
0.12455612	0.09117184	-0.0735632	0.31967081	0.14142835	0.05482523
-0.2509568	0.00056989	-0.1373591	0.06225564	0.13621479	0.06448806
-0.0043978	0.95262454	0.08309094	0.26082447	-0.0935994	0.20506801
0.38523104	6.14E-08	0.29689023	4.07E-05	-0.1582557	0.03143698
0.01569326	0.8320932	0.0043909	0.95269867	-0.0812585	0.27152253
-0.0719628	0.33034049	-0.2542368	0.00047921	0.13997934	0.05738228
-0.0168717	0.81969276	0.14060321	0.05626954	-0.2595931	0.00035932
0.15224108	0.03857029	0.27982034	0.00011454	-0.5029381	2.98E-13
-0.114772	0.11979568	-0.0721822	0.32886405	0.57458828	1.21E-17
-0.1945408	0.00796599	-0.1606877	0.02888927	0.32898886	4.82E-06
0.0136247	0.8539598	0.17168071	0.01945691	0.45621811	6.75E-11
-0.0245454	0.74016069	-0.0023613	0.97455189	0.08077657	0.27438475
-0.4102616	6.64E-09	-0.290308	6.11E-05	0.27539357	0.00014824
-0.1388312	0.05947763	-0.0574185	0.43755772	-0.2662711	0.0002488
-0.0564277	0.44551969	0.25433584	0.0004767	-0.3630361	3.80E-07
0.14535722	0.0483625	0.04057279	0.58346183	0.14859552	0.04352468
-0.0111199	0.88058551	-0.0396514	0.59204696	0.05291575	0.47438957
0.07283255	0.32451374	-0.1103115	0.13496816	0.11604199	0.11572748
-0.0412419	0.57726386	-0.1287283	0.0807588	0.14577016	0.04772171
-0.1090431	0.13954078	-0.1296857	0.07850999	0.10948542	0.1379329
0.1528322	0.03781355	0.16583118	0.02407672	0.02663321	0.71895224
-0.2374206	0.00113778	-0.2496194	0.00061122	0.13845535	0.06017698
-0.2193551	0.00270022	-0.2519982	0.00053952	0.08122408	0.27172615
-0.4214447	2.31E-09	-0.3685489	2.45E-07	0.16044045	0.02913998
-0.0796209	0.28133148	-0.0219164	0.7671457	-0.2330582	0.00141033
-0.2954575	4.45E-05	-0.2266425	0.00192058	0.13558399	0.0657464
0.29276629	5.25E-05	0.18041387	0.01399229	-0.0471289	0.52410444
-0.1537993	0.03660246	-0.2292982	0.00169183	0.24642234	0.00072148
-0.1954731	0.00766517	-0.2608734	0.00033512	-0.1842554	0.01204948
-0.2191927	0.00272046	-0.1937195	0.0082397	0.15792297	0.03179992
-0.0625956	0.39730045	-0.1755742	0.01682636	0.22897931	0.00171792
-0.142064	0.0537336	-0.1657458	0.02415057	0.15092249	0.04030442
-0.2241328	0.00216227	-0.0041193	0.95562153	-0.4014388	1.49E-08
-0.0388294	0.59975376	-0.1229724	0.09539425	0.1877579	0.01048902
0.0451786	0.54143639	-0.0616903	0.40417613	0.11782349	0.11020346
-0.1020703	0.16681525	-0.0385619	0.60227135	-0.0168005	0.82044032
-0.124955	0.09013228	-0.0839501	0.25590974	-0.4247503	1.68E-09
0.0505852	0.49409659	0.10638039	0.14952382	0.0966965	0.19040879
-0.1004688	0.17360626	-0.1150913	0.11876256	0.3322695	3.82E-06
0.34316049	1.74E-06	0.26657962	0.00024455	-0.3074214	2.08E-05
-0.1084683	0.14165138	-0.1047925	0.1557291	0.22715293	0.00187453
0.39482221	2.68E-08	0.33641488	2.84E-06	0.21486095	0.00331407
0.26190331	0.00031676	0.10369163	0.16014364	0.36004558	4.81E-07
-0.2290636	0.00171099	-0.2578279	0.00039536	0.22159781	0.00243417

0.1211146	0.10054394	-0.1994687	0.00648706	-0.2215814	0.00243603
-0.0224853	0.76128163	-0.2849327	8.46E-05	0.26428848	0.00027777
-0.0276524	0.70867557	-0.1440738	0.05039993	0.28651312	7.69E-05
0.33497475	3.15E-06	0.2696151	0.00020622	0.21769799	0.00291342
0.0729189	0.32393892	0.17208516	0.019168	-0.3491885	1.11E-06
-0.1868784	0.01086311	-0.1199632	0.10384423	-0.247463	0.00068372
-0.3488094	1.14E-06	-0.2941865	4.81E-05	0.54376898	1.26E-15
-0.0196347	0.79079616	0.01037737	0.88850621	0.28572171	8.07E-05
-0.1373864	0.06220322	-0.1555822	0.03445563	0.21281172	0.00363373
-0.0425429	0.56530149	-0.1806834	0.01384753	0.25388856	0.00048816
0.39637548	2.33E-08	0.08802511	0.23346902	-0.2828897	9.55E-05
-0.3996844	1.74E-08	-0.2340743	0.00134199	0.35860275	5.38E-07
-0.1665991	0.02342096	-0.2093597	0.00423554	0.0595702	0.4205507
0.48915056	1.61E-12	0.23777687	0.0011178	-0.1694657	0.021107
0.12315103	0.09491044	0.0357355	0.62915589	0.06768084	0.35999761
-0.2265949	0.00192491	-0.1588914	0.03075321	0.13922723	0.05874782
-0.206676	0.00476383	-0.2054671	0.00502057	0.13678686	0.06336399
-0.0878653	0.23432228	-0.010898	0.88295181	-0.2079616	0.00450381
0.01034276	0.88887569	-0.0821152	0.26648413	0.26873836	0.00021667
0.1940691	0.00812219	0.01618595	0.82690327	0.28932094	6.49E-05
0.00469288	0.94944974	-0.0503691	0.49594551	0.30445475	2.52E-05
-0.0233446	0.75244899	-0.0395324	0.59315965	0.2455908	0.00075302
-0.4531602	9.36E-11	-0.3467065	1.33E-06	-0.4298891	1.02E-09
-0.0385895	0.60201142	-0.1110758	0.13226861	0.05759684	0.43613367
0.04707241	0.52460224	-0.1418345	0.05412559	0.19498712	0.0078207
-0.1634825	0.0261813	-0.1548968	0.03526803	0.22246705	0.00233763
0.0034862	0.9624365	-0.0388097	0.59993864	0.2611378	0.00033032
-0.2264712	0.00193625	-0.084489	0.2528595	-0.1728602	0.01862475
-0.1150256	0.11897445	-0.0391111	0.59710712	-0.3259049	5.98E-06
0.39233008	3.33E-08	0.45418032	8.39E-11	-0.2062969	0.00484304
-0.2042862	0.00528326	-0.0705748	0.33977715	-0.3389174	2.37E-06
-0.3354924	3.04E-06	-0.3237884	6.93E-06	0.00202591	0.97816578
-0.3823266	7.86E-08	-0.3803656	9.27E-08	0.41142613	5.96E-09
0.27293814	0.00017072	0.21590284	0.00316148	-0.3012436	3.09E-05
-0.1704343	0.02037111	-0.2567536	0.0004189	0.29289242	5.21E-05
-0.3011134	3.11E-05	-0.3469046	1.31E-06	0.4809075	4.25E-12
0.23072825	0.00157923	0.12836146	0.0816342	-0.219379	0.00269724
0.38696579	5.30E-08	0.32717167	5.48E-06	-0.2101325	0.00409353
0.14932191	0.04249708	-0.0402118	0.5868184	-0.2317042	0.00150635
-0.2220383	0.0023848	-0.1934115	0.00834446	0.17670799	0.01612102
-0.2258212	0.00199681	-0.1485469	0.04359427	-0.0658182	0.37340042
-0.2504149	0.00058632	-0.3312953	4.10E-06	0.11622097	0.11516294
-0.1017384	0.16820596	-0.0550124	0.45703254	-0.3074344	2.07E-05
-0.0904607	0.22073662	-0.0196374	0.79076883	-0.3451856	1.49E-06
-0.1304882	0.07666396	-0.1071189	0.14670237	-0.2673564	0.00023416
0.07985062	0.27994148	-0.0214348	0.77212108	0.14078916	0.05594135

-0.0599787	0.41736595	-0.1126019	0.12700294	-0.1883611	0.01023908
0.08526226	0.24852707	-0.0511133	0.48959318	-0.376527	1.28E-07
-0.12386	0.09300912	0.09790675	0.18489353	-0.5271598	1.27E-14
0.18115479	0.01359753	0.12298504	0.09536004	0.04077367	0.58159784
-0.1898916	0.00962836	-0.2375209	0.00113212	-0.3630206	3.80E-07
-0.1818641	0.01322882	-0.1247252	0.09072986	0.08949302	0.22573497
0.00946739	0.89822776	-0.0137362	0.8527779	-0.1896361	0.00972802
-0.1362405	0.06443724	-0.163647	0.02602887	-0.2978446	3.83E-05
-0.1321648	0.07292068	-0.0874644	0.23647208	0.25695444	0.0004144
-0.1869851	0.01081711	-0.212674	0.00365618	0.32518464	6.29E-06
-0.061932	0.4023335	-0.1203514	0.10272213	0.16035886	0.02922311
-0.352382	8.69E-07	-0.320429	8.72E-06	0.11048713	0.13434398
-0.2127395	0.00364549	-0.1247671	0.09062065	0.27215673	0.00017851
0.01926616	0.79463531	-0.0723349	0.32783965	0.34693852	1.31E-06
0.10075257	0.17238835	0.01235832	0.86740282	-0.2379243	0.00110963
-0.0871006	0.23843461	-0.1742221	0.01770247	-0.4356699	5.72E-10
-0.358358	5.49E-07	-0.3009055	3.16E-05	0.18202174	0.01314804
-0.0372853	0.61435092	-0.1418342	0.05412626	0.28273831	9.64E-05
0.18653475	0.01101242	0.07559031	0.30648059	-0.2793121	0.00011801
-0.2613533	0.00032645	-0.1464586	0.04666919	-0.2284459	0.00176238
-0.2106224	0.00400574	-0.0764613	0.30092501	-0.4179037	3.25E-09
-0.123561	0.09380735	-0.2005922	0.0061863	0.13326297	0.07055029
0.13421073	0.06855527	0.00455281	0.95095664	0.22039732	0.00257346
-0.1814211	0.01345806	-0.1629943	0.02663798	0.35222937	8.79E-07
-0.2359877	0.00122146	-0.1315682	0.07423527	0.2589238	0.00037261
-0.2328873	0.00142213	-0.1332821	0.07050965	0.1265909	0.08596698
-0.0353397	0.63296077	-0.1692248	0.02129351	0.25904885	0.0003701
0.20851747	0.00439537	0.06777304	0.35934207	0.23830296	0.0010889
-0.0725746	0.3262351	-0.0217698	0.7686589	0.22662548	0.00192212
-0.0761121	0.30314441	-0.1053773	0.1534217	0.21362943	0.00350298
-0.1247744	0.09060178	-0.1751549	0.01709393	0.07967622	0.28099652
-0.0125466	0.86540179	-0.0606064	0.41249972	0.18783504	0.01045676
-0.2713757	0.00018663	-0.0568456	0.44215145	0.20489599	0.0051461
-0.0869278	0.23937112	-0.0754678	0.3072674	-0.1564518	0.03344794
0.09341093	0.20598549	0.01927212	0.79457324	0.28407228	8.90E-05
0.32058909	8.63E-06	0.14392961	0.05063329	0.25629472	0.00042934
-0.0156842	0.83218846	0.02418877	0.74380352	0.40389436	1.19E-08
0.18919251	0.0099032	0.07889389	0.28576209	0.17939362	0.01455228
-0.1435509	0.05125038	-0.2646372	0.00027245	0.11002396	0.13599443
0.21524021	0.00325777	0.06697264	0.36505774	0.33953083	2.27E-06
0.08158584	0.26958981	0.02745398	0.71067216	0.23663048	0.00118324
0.18777527	0.01048175	0.0488034	0.50945145	0.27881286	0.00012151
-0.2966575	4.13E-05	-0.2001151	0.00631248	0.2949201	4.60E-05
0.07651704	0.30057143	-0.0415239	0.57466096	0.23829711	0.00108921
-0.0301539	0.68367705	-0.1228851	0.09563167	0.19418264	0.00808435
-0.2997942	3.39E-05	-0.1947446	0.00789937	0.33674124	2.78E-06

0.09105971	0.2176825	-0.0070143	0.92450622	0.19277469	0.00856481
-0.0137129	0.85302469	-0.1055194	0.15286468	0.14547562	0.04817804
0.17477613	0.01733878	-0.0158017	0.8309498	0.13553791	0.0658391
-0.2283856	0.00176747	-0.17908	0.01472831	-0.1230746	0.09511722
0.08336499	0.2592498	-0.0622257	0.40010125	0.17863091	0.01498362
-0.1135449	0.12383099	-0.1822764	0.01301849	0.06552053	0.37557015
0.20048203	0.00621524	-0.0024852	0.97321786	-0.0796278	0.28128994
-0.1968808	0.00723001	-0.2354984	0.0012513	-0.4233337	1.93E-09
-0.1528118	0.03783951	-0.1041689	0.15821834	-0.491384	1.23E-12
0.26098405	0.0003331	-0.011497	0.87656823	0.12323918	0.09467238
-0.2402336	0.00098859	-0.2336573	0.00136966	0.53260634	6.04E-15
-0.032407	0.6614508	-0.0564807	0.44509155	-0.2440789	0.00081363
0.19998144	0.00634823	0.13194626	0.07340007	0.24224298	0.00089327
0.10073847	0.17244872	0.0202388	0.78451531	0.00079017	0.99148308
-0.0979783	0.18457135	-0.014483	0.84487175	-0.2348423	0.00129236
-0.1817196	0.01330317	-0.1688952	0.02155106	-0.108187	0.1426933
-0.100101	0.1751946	-0.2121614	0.00374084	0.46901892	1.65E-11
0.08339983	0.25905005	-0.0300473	0.68473558	0.12812317	0.08220689
-0.0883772	0.23159735	-0.1397705	0.05775875	0.06462427	0.38215015
-0.1554681	0.03458984	-0.2965732	4.15E-05	-0.1847304	0.01182656
0.03222928	0.66319401	-0.0278467	0.70672193	0.0867698	0.24022921
-0.0950762	0.19797931	-0.1396959	0.05789375	0.18704079	0.01079315
-0.1503689	0.04105179	-0.0794592	0.28231312	-0.2636319	0.00028803
0.17606034	0.01652069	0.15997771	0.02961415	-0.458548	5.25E-11
-0.069446	0.34757661	-0.1602884	0.02929511	0.25951579	0.00036084
-0.2394317	0.00102917	-0.3552079	7.00E-07	0.20623515	0.00485606
-0.4069923	8.98E-09	-0.2165871	0.00306476	-0.1431141	0.05196969
-0.1074582	0.14541963	-0.1646134	0.02514899	-0.0328742	0.65687889
-0.1471963	0.04556285	-0.1554613	0.03459781	-0.4205263	2.53E-09
-0.1929361	0.00850849	-0.2099255	0.00413115	0.19463598	0.00793482
0.0574127	0.43760437	-0.0817649	0.26853637	0.02078923	0.77880398
-0.0807872	0.27432167	-0.1896824	0.0097099	0.40614794	9.70E-09
0.04167511	0.57326767	-0.0634399	0.39095216	0.13367236	0.06968281
0.11182508	0.12966264	-0.0243365	0.74229378	0.18603637	0.0112322
-0.1084076	0.14187582	-0.2573769	0.00040509	0.15900414	0.03063329
0.17530808	0.01699573	0.03720635	0.61510125	0.25413953	0.0004817
-0.3948959	2.66E-08	-0.3368064	2.76E-06	0.05979965	0.41876011
0.06147138	0.40584903	0.01280506	0.86265594	0.17039384	0.02040141
-0.2451053	0.00077201	-0.3196346	9.21E-06	0.33514639	3.11E-06
-0.2819495	0.00010103	-0.0663593	0.36947558	-0.201154	0.00604063
-0.3504671	1.01E-06	-0.2810853	0.00010632	0.15042529	0.04097512
0.27783564	0.00012865	0.0900643	0.22277459	0.14269877	0.05266157
-0.0675043	0.3612548	-0.0926413	0.20976346	0.19720211	0.00713382
-0.0108443	0.88352467	-0.1702441	0.02051384	0.30724603	2.10E-05
-0.1047854	0.15575748	0.16066012	0.02891716	-0.3471333	1.29E-06
-0.1345025	0.06795037	-0.1464922	0.04661821	0.18395319	0.01219322

-0.2188854	0.00275915	-0.0681974	0.35633414	-0.0971129	0.18849801
-0.0257515	0.72788369	-0.0508549	0.49179388	-0.2107551	0.00398224
-0.1685301	0.02183954	-0.0980718	0.18415042	-0.0682711	0.35581372
-0.0339249	0.64664156	-0.0937548	0.20431363	-0.3192384	9.46E-06
-0.1617268	0.02785578	-0.1078548	0.14393109	-0.0383695	0.60408508
-0.0685181	0.35407211	-0.0556354	0.4519443	-0.1082183	0.14257714
-0.0007854	0.99153458	0.25854641	0.0003803	-0.3566442	6.27E-07
0.18789619	0.01043125	0.06387898	0.38767478	0.12629736	0.08670279
0.24675152	0.00070933	0.02390198	0.74673708	-0.1929907	0.0084895
0.1243627	0.0916794	0.27534721	0.00014864	-0.1881746	0.0103158
-0.0986364	0.18162563	-0.1349973	0.06693475	-0.3508876	9.74E-07
-0.0313771	0.67157541	-0.0491062	0.50682437	0.18881562	0.01005424
-0.1618631	0.02772258	-0.2032608	0.00552127	0.05496005	0.45746195
-0.2472185	0.00069243	-0.2138301	0.00347155	-0.3316708	3.99E-06
-0.0441902	0.55032718	-0.1450165	0.04889661	0.07095878	0.33714952
-0.2295303	0.00167307	-0.2475668	0.00068006	0.26371807	0.00028666
0.06883495	0.35184549	0.12472633	0.09072704	0.27683771	0.00013635
0.01911734	0.79618689	-0.1131518	0.12514552	-0.329834	4.54E-06
-0.3919652	3.44E-08	-0.4003326	1.64E-08	0.11684674	0.11320596
0.11264069	0.12687115	0.05798273	0.43306071	-0.0280119	0.70506345
-0.2248869	0.00208689	-0.1488714	0.04313195	0.27552745	0.0001471
0.18513681	0.01163868	-0.0039673	0.95725706	0.10197021	0.16723373
0.16776255	0.02245671	0.04283491	0.56263323	0.22565537	0.00201254
0.14041423	0.05660472	0.09100554	0.21795746	-0.0097521	0.89518415
0.19092023	0.00923621	0.21262266	0.00366458	-0.0935443	0.205336
0.01125898	0.87910368	-0.0965132	0.19125443	-0.0218859	0.76746049
0.00500781	0.94606237	0.05432413	0.46269127	-0.2152939	0.00324987
-0.0867734	0.24020951	-0.0881255	0.23293411	0.06016543	0.41591478
0.053739	0.46753203	0.00436952	0.95292867	-0.1687323	0.02167933
0.26368743	0.00028715	0.01532231	0.83600547	0.04208886	0.56946313
-0.4845811	2.77E-12	-0.3671164	2.75E-07	0.48206173	3.72E-12
0.05424061	0.46338054	-0.035587	0.63058253	0.18560466	0.01142569
-0.2012922	0.00600528	-0.1259616	0.0875505	0.1815869	0.01337184
-0.3433367	1.71E-06	-0.3003784	3.26E-05	0.17002715	0.02067769
0.15000405	0.04155067	-0.0300554	0.6846553	0.22645252	0.00193797
-0.0365074	0.62176277	-0.075623	0.30627116	0.31624246	1.16E-05
-0.1112777	0.13156252	-0.1163211	0.11484795	-0.050388	0.49578347
-0.0196985	0.79013271	-0.0541741	0.4639301	-0.013757	0.8525579
-0.0595548	0.42067071	0.06831784	0.35548366	-0.2919143	5.54E-05
-0.1701797	0.02056236	-0.1080688	0.14313275	0.18371013	0.01230992
0.09883619	0.18073819	0.06828772	0.35569634	-0.2346192	0.0013066
-0.3469179	1.31E-06	-0.2116939	0.00381958	0.24718384	0.00069367
0.1068725	0.14763916	0.1213634	0.09984182	0.17302766	0.01850914
-0.0737262	0.31859663	-0.1479622	0.04443749	0.16191722	0.02766983
0.24408656	0.00081332	0.18429557	0.01203047	-0.3416494	1.94E-06
0.00698397	0.92483149	-0.0193711	0.79354214	0.01984678	0.78858987

-0.047993	0.51651663	0.12019849	0.10316295	-0.0425212	0.56550023
-0.2311673	0.00154605	-0.2079577	0.00450456	0.36473719	3.32E-07
0.03067885	0.67847367	-0.0631377	0.39321773	0.36082759	4.52E-07
-0.0063359	0.93178925	-0.0479884	0.51655654	-0.1873663	0.01065417
-0.0967679	0.19008009	-0.1989043	0.00664302	-0.2257668	0.00200196
-0.0589558	0.42536742	-0.2332513	0.0013971	0.22692714	0.00189477
-0.2171488	0.00298737	-0.239954	0.00100257	0.49950971	4.56E-13
-0.0601137	0.41631633	-0.1598435	0.02975289	0.28875027	6.72E-05
-0.104313	0.15764068	-0.0909568	0.2182052	0.09543456	0.19628657
-0.2548598	0.00046358	-0.2511866	0.00056306	-0.2465803	0.00071563
-0.1112668	0.13160055	0.01456198	0.84403706	-0.1378619	0.06129514
-0.2341306	0.00133829	-0.080341	0.27698925	-0.0050921	0.9451554
-0.2624128	0.00030803	-0.2109743	0.00394371	0.40473643	1.10E-08
0.08426176	0.25414256	0.12985826	0.07810999	0.05555412	0.4526066
-0.0324708	0.6608265	0.00469355	0.94944255	-0.2122561	0.00372507
-0.4109823	6.21E-09	-0.2528022	0.00051709	-0.3871219	5.22E-08
-0.0199398	0.78762213	-0.0587493	0.42699355	0.30635483	2.22E-05
-0.0352716	0.63361696	-0.1805722	0.0139071	0.31755994	1.06E-05
-0.3368297	2.76E-06	-0.2158205	0.00317331	0.46247895	3.42E-11
-0.4203645	2.57E-09	-0.1861265	0.01119215	0.40926523	7.28E-09
-0.171908	0.01929408	-0.2044698	0.00524163	0.16937789	0.02117483
-0.0555933	0.4522871	-0.2554189	0.00044995	0.25205256	0.00053797
-0.0432137	0.55918027	0.07884942	0.28603458	-0.0525166	0.47773395
-0.1037666	0.15984012	-0.2720346	0.00017976	0.25268291	0.00052037
-0.3186988	9.81E-06	-0.1025486	0.164826	-0.3392826	2.31E-06
0.04365047	0.55521228	0.02246578	0.76148228	0.27256618	0.00017439
0.33211645	3.87E-06	0.189655	0.00972061	0.04479458	0.54488233
0.13092845	0.07566634	0.28193259	0.00010113	-0.2819749	0.00010087
-0.1276199	0.08342702	-0.0752408	0.30872893	-0.2992482	3.51E-05
0.31805738	1.02E-05	0.05786251	0.43401672	-0.2111808	0.00390772
0.16387807	0.02581616	-0.0034871	0.96242666	0.17189656	0.01930226
0.25009987	0.00059607	-0.1027743	0.1638932	-0.3000188	3.34E-05
-0.0345796	0.640296	-0.205792	0.00495037	0.16233109	0.02726938
-0.3622418	4.05E-07	-0.2552689	0.00045357	0.51558283	5.92E-14
-0.0401782	0.587131	-0.0484876	0.5121981	0.33105422	4.17E-06
-0.0427624	0.56329563	-0.1391584	0.05887412	0.08968801	0.22472142
-0.2155487	0.00321262	-0.0084162	0.90947675	-0.2720682	0.00017942
0.19175726	0.0089276	0.11449387	0.12070112	-0.2139248	0.00345681
-0.1297419	0.07837956	-0.0279709	0.70547417	0.40343252	1.24E-08
0.31144752	1.59E-05	0.14217398	0.05354659	0.35093545	9.70E-07
0.3547381	7.26E-07	0.14770405	0.04481421	-0.2638327	0.00028485
0.08226182	0.26562857	0.10431654	0.15762637	-0.221017	0.00250069
-0.0108171	0.88381468	0.02471897	0.73838964	-0.2258219	0.00199675
-0.2407291	0.00096426	-0.2660556	0.00025181	0.14393607	0.05062282
-0.0070059	0.92459584	-0.0917143	0.21437926	0.0122383	0.86867888
-0.0754112	0.30763163	-0.1474977	0.04511726	0.29632074	4.21E-05

-0.1940504	0.00812844	-0.2163464	0.00309848	0.32686398	5.60E-06
0.21081715	0.0039713	0.07022043	0.34221357	-0.288653	6.76E-05
-0.1191484	0.10623096	-0.2561658	0.00043232	0.13381365	0.06938544
-0.0114018	0.87758165	-0.1137728	0.12307345	0.0051999	0.94399676
-0.2637199	0.00028663	-0.1113226	0.13140585	0.03356765	0.65011484
-0.2295002	0.00167549	-0.2355104	0.00125055	0.15993577	0.02965745
0.14206297	0.05373538	0.01461273	0.84350043	-0.2115449	0.003845
0.07390251	0.31743749	0.0468064	0.52695061	-0.4489353	1.46E-10
-0.3062354	2.24E-05	-0.1436066	0.05115918	0.17367681	0.01806685
0.12521206	0.08946722	0.22746064	0.00184727	-0.083232	0.26001329
0.0228126	0.75791341	0.09142811	0.21581911	-0.1638158	0.02587332
-0.2451818	0.00076899	-0.1368001	0.06333824	0.3923347	3.33E-08
-0.3374565	2.64E-06	-0.2603797	0.00034427	-0.1138653	0.12276717
0.49009618	1.44E-12	0.28519306	8.33E-05	-0.0508452	0.49187709
-0.0821292	0.26640244	-0.1225511	0.0965434	0.19241156	0.00869277
0.2168778	0.00302449	0.13012327	0.077499	-0.3128304	1.45E-05
-0.0534161	0.47021513	-0.1226097	0.096383	0.2672132	0.00023604
-0.0924723	0.2105996	-0.0780192	0.29115436	-0.1733047	0.01831923
0.19526219	0.00773232	0.03421802	0.64379717	0.08693888	0.23931079
-0.001333	0.98563258	-0.1156068	0.1171091	-0.1793731	0.01456373
-0.2237838	0.00219798	-0.2022342	0.00576912	-0.0086001	0.90750702
-0.1693489	0.02119729	-0.0628642	0.39527427	-0.0226476	0.75961081
-0.1616033	0.02797689	0.05247743	0.478063	-0.3094214	1.82E-05
-0.1163892	0.11463426	-0.0130876	0.85965586	-0.2756868	0.00014575
-0.0465082	0.5295898	-0.1055779	0.15263623	0.03114814	0.67383461
-0.1335482	0.06994494	-0.0661619	0.3709046	0.01544406	0.83472105
-0.051803	0.48374536	-0.106218	0.15014974	-0.0620897	0.40113414
0.02186744	0.76765115	-0.0835749	0.25804804	0.23095492	0.00156202
0.22040817	0.00257217	0.05647499	0.44513772	-0.3174279	1.07E-05
0.367792	2.60E-07	0.20190659	0.0058503	0.07157252	0.33297669
0.08749729	0.23629499	-0.0108152	0.88383479	0.05204584	0.48169502
-0.0443162	0.54918956	-0.2059472	0.00491716	0.1129475	0.12583315
-0.1785225	0.01504585	-0.0789477	0.28543271	0.30450006	2.51E-05
0.18668642	0.0109463	-0.0407509	0.58180941	0.17596885	0.01657784
-0.3123271	1.50E-05	-0.2206792	0.00254012	0.47131172	1.28E-11
-0.0931959	0.20703587	-0.238369	0.00108532	0.2724866	0.00017518
0.114051	0.12215401	0.03002088	0.68499837	0.31637572	1.15E-05
0.57317856	1.51E-17	0.25452435	0.00047194	-0.2579058	0.0003937
-0.1329966	0.07111951	-0.2115306	0.00384744	0.23101691	0.00155734
0.00223807	0.97587979	0.06759598	0.36060161	-0.336849	2.75E-06
-0.0187451	0.80007059	0.12364296	0.09358791	0.07799767	0.29128763
0.22501577	0.00207425	0.10382588	0.15960034	0.49748485	5.85E-13
0.59616013	3.47E-19	0.23988397	0.0010061	0.27772265	0.0001295
0.0322402	0.66308688	0.05237822	0.47889656	0.04894824	0.50819407
-0.1979601	0.00691143	-0.2545067	0.00047238	-0.1210679	0.1006762
-0.3007876	3.18E-05	-0.2292343	0.00169703	-0.322219	7.72E-06

-0.066506	0.36841614	-0.0357376	0.62913569	-0.224871	0.00208846
-0.0143991	0.84575936	-0.1586629	0.03099752	0.37024164	2.13E-07
-0.0293758	0.69141754	-0.0912486	0.21672564	0.31576483	1.20E-05
0.03921648	0.59611858	-0.0509055	0.49136272	0.19099721	0.00920744
-0.0315318	0.67005153	-0.0893287	0.22659169	-0.2079287	0.00451028
0.09596434	0.19380316	0.06184932	0.40296313	0.01581185	0.83084332
0.1641648	0.02555424	0.01988825	0.78815864	0.24699806	0.00070036
-0.3079617	2.00E-05	-0.208034	0.00448954	0.08786717	0.23431211
-0.1385824	0.05993973	-0.1061271	0.15050105	-0.1287752	0.08064739
-0.1433617	0.051561	-0.0565944	0.44417415	-0.1059041	0.15136522
0.04189682	0.57122744	0.11395781	0.12246146	-0.2692559	0.00021045
0.07098536	0.33696812	-0.0897349	0.22447812	0.31915953	9.51E-06
0.40249948	1.35E-08	0.23249191	0.00144979	-0.3148699	1.27E-05
0.17750579	0.0156403	-0.0017576	0.98105673	0.13024353	0.07722302
0.39270361	3.22E-08	0.13626594	0.0643869	-0.1698122	0.02084116
-0.3461821	1.39E-06	-0.1010427	0.17114975	0.26445224	0.00027526
0.15786163	0.03186723	0.04336654	0.5577904	-0.1640033	0.02570145
-0.1895375	0.00976673	-0.1800514	0.01418905	0.26484296	0.00026936
-0.0142622	0.84720778	0.0185074	0.80255402	-0.2158641	0.00316704
0.04301365	0.56100278	-0.0818368	0.26811479	0.38670877	5.41E-08
-0.3251696	6.30E-06	-0.2819205	0.0001012	0.18877643	0.01007006
-0.1043306	0.15757004	-0.1928035	0.00855472	0.20578185	0.00495255
0.08185775	0.26799161	-0.0568208	0.44235109	-0.4028113	1.31E-08
-0.393754	2.94E-08	-0.396037	2.40E-08	0.25937883	0.00036353
-0.1418501	0.0540989	-0.0693099	0.34852468	-0.1592548	0.03036803
0.33095838	4.20E-06	0.25875402	0.00037605	-0.4428095	2.77E-10
-0.0875541	0.23598962	-0.0976135	0.1862189	0.35951532	5.01E-07
0.03069182	0.67834526	0.05366244	0.46816748	-0.0491775	0.5062069
-0.3028622	2.79E-05	-0.1819809	0.01316894	-0.257256	0.00040773
-0.1941206	0.008105	-0.092858	0.20869464	0.15255901	0.0381617
-0.3081068	1.99E-05	-0.1748211	0.01730954	-0.1974102	0.00707214
0.63273698	4.39E-22	0.23437127	0.0013226	0.10245127	0.16522922
-0.2514474	0.00055539	-0.1184329	0.10836203	-0.0269141	0.71611508
0.4674305	1.98E-11	0.18466298	0.01185795	0.08415224	0.25476251
0.5450443	1.05E-15	0.41186751	5.72E-09	0.17809164	0.0152953
0.33425185	3.32E-06	0.05698825	0.44100531	0.15407922	0.03625814
0.22210264	0.00237767	0.02871495	0.69801726	-0.0614186	0.40625324
0.34614563	1.39E-06	0.20071079	0.0061553	-0.0539016	0.46618426
0.67702846	3.72E-26	0.37457122	1.50E-07	0.14722388	0.04552192
0.49982438	4.38E-13	0.23019198	0.00162062	0.13112688	0.07522013
0.50392454	2.63E-13	0.24582052	0.00074418	0.03835366	0.60423424
0.47012248	1.46E-11	0.21485856	0.00331442	0.22315019	0.0022642
0.69064105	1.49E-27	0.3634193	3.69E-07	0.12936885	0.07924853
-0.4137227	4.81E-09	-0.1717335	0.01941899	-0.2179748	0.00287678
0.49181885	1.17E-12	0.20250114	0.00570374	0.20598216	0.0049097
0.56538799	5.09E-17	0.28228469	9.90E-05	0.06602116	0.37192512

-0.366186	2.96E-07	-0.1910966	0.00917041	-0.2055697	0.0049983
0.44948172	1.38E-10	0.22337528	0.00224048	-0.0381342	0.60630641
0.33287938	3.66E-06	0.15308587	0.03749267	0.17944003	0.01452639
0.36252211	3.96E-07	0.17843408	0.01509673	0.06065956	0.41208918
0.46272576	3.33E-11	0.31189351	1.55E-05	0.05946803	0.42134945
0.53649125	3.52E-15	0.24282236	0.00086739	0.06534031	0.37688766
0.42158946	2.28E-09	0.22087851	0.00251679	0.07747451	0.29454588
0.36165226	4.24E-07	0.14601762	0.04734111	0.09118074	0.21706904
0.24576911	0.00074615	0.11801443	0.10962385	0.1258829	0.0877503
0.66792098	2.92E-25	0.37506748	1.44E-07	0.16302567	0.02660846
0.40941938	7.18E-09	0.19001705	0.00957975	0.1214796	0.09951522
0.33835082	2.47E-06	0.16287209	0.0267534	0.08351551	0.25838764
-0.4545789	8.04E-11	-0.4351539	6.02E-10	-0.156412	0.03349349
0.21415037	0.00342191	0.11966676	0.10470757	0.12540623	0.08896754
0.42397588	1.81E-09	0.20597711	0.00491077	0.1398076	0.05769176
0.66226314	1.01E-24	0.34124561	2.00E-06	0.12525312	0.08936137
0.67884482	2.45E-26	0.43210363	8.17E-10	0.19614764	0.00745382
0.43529405	5.94E-10	0.19671295	0.00728072	0.22253837	0.00232986
0.4069039	9.05E-09	0.17482117	0.01730951	0.01783435	0.80959507
0.46877768	1.70E-11	0.11325503	0.12479932	0.07477228	0.31175965
0.35615845	6.51E-07	0.10600138	0.15098765	0.11105482	0.13234212
0.31618459	1.16E-05	0.07794582	0.2916095	0.01312468	0.85926287
0.28495551	8.45E-05	0.24033779	0.00098343	0.07475543	0.31186902
-0.3044442	2.52E-05	-0.1477834	0.04469808	-0.1974426	0.00706257
0.16792924	0.02232141	0.11501251	0.11901673	0.13174812	0.07383688
0.49042315	1.38E-12	0.18384785	0.01224367	0.19474539	0.0078991
-0.3627971	3.87E-07	-0.2140674	0.0034347	-0.086426	0.24210413
0.57581535	9.96E-18	0.34253494	1.82E-06	0.09925244	0.1788997
0.47967534	4.91E-12	0.17872522	0.01492968	0.12642752	0.08637591
-0.2815628	0.00010336	-0.2063033	0.0048417	-0.1746561	0.01741702
-0.3076737	2.04E-05	-0.2065925	0.00478118	0.02312009	0.75475362
0.40444467	1.13E-08	0.23774565	0.00111954	0.28752308	7.24E-05
0.59484418	4.34E-19	0.27811735	0.00012656	0.17490899	0.01725254
0.26035575	0.00034472	0.09854455	0.1820346	0.12608286	0.08724367
0.33637095	2.85E-06	0.10736423	0.14577406	0.14421604	0.05017071
0.3071706	2.11E-05	0.13615314	0.06461015	0.09717971	0.18819249
0.23151618	0.00152015	0.08453729	0.25258744	0.16981735	0.02083722
0.45566124	7.16E-11	0.22255347	0.00232822	0.19140432	0.0090566
0.45926007	4.86E-11	0.11618773	0.11526764	0.13852585	0.06004526
0.34646435	1.36E-06	0.09908395	0.17964225	-0.0420326	0.56997934
0.42864924	1.15E-09	0.07360818	0.31937401	0.15371007	0.03671284
0.3626057	3.93E-07	0.14539608	0.04830189	0.08357631	0.25803999
0.23676582	0.00117534	0.01695803	0.81878554	0.25839615	0.00038341
0.28991746	6.26E-05	0.05380021	0.46702435	0.2111869	0.00390667
0.60120966	1.45E-19	0.43375396	6.93E-10	0.0148598	0.84088915
0.45192813	1.07E-10	0.17283269	0.01864379	0.1770661	0.01590367

0.34794178	1.22E-06	0.09594172	0.19390873	-0.0491635	0.50632802
0.19959731	0.00645199	0.0119259	0.87200195	0.12355237	0.09383035
0.23006773	0.00163036	0.1069005	0.1475325	0.1754472	0.01690699
0.59774338	2.64E-19	0.4876508	1.92E-12	0.11415774	0.12180261
0.28220189	9.95E-05	0.15030699	0.04113607	0.08635172	0.24251078
0.41198332	5.66E-09	0.23309152	0.00140804	0.06934451	0.34828338
0.28294526	9.52E-05	0.03436276	0.64239461	0.01684428	0.81998033
0.57941391	5.59E-18	0.42260777	2.07E-09	0.22623989	0.00195761
0.22690132	0.0018971	0.05687802	0.44189097	0.06621098	0.37054877
