# Supplementary material for: Down-Regulation of Insulin Like Growth Factor 1 Involved in Alzheimer's Disease via MAPK, Ras, and FoxO Signaling Pathways
Source: Oxid Med Cell Longev. 2022 May 4;2022:8169981. doi: 10.1155/2022/8169981 (PMC9096571; doi:10.1155/2022/8169981)
Supplement: Supplementary 3 — Supplementary Table 3: Identification of pathway representative genes by PCC analysis: pathway representative genes are highlighted in yellow. PCC: Pearson's correlation coefficient. [file 8169981.f3.pdf]

MAPK_genes

PCC	Gene.symb	VEGFB	TNFRSF1A	TGFB3	STK3
51.21325635	PPP3CB	-0.746511391	-0.750550294	-0.73715911	-0.637183716
51.03983217	PAK1	-0.697228619	-0.743502262	-0.76122483	-0.706622161
50.96263013	PRKCB	-0.689738457	-0.733684119	-0.759813553	-0.563065519
50.13050038	MAP2K1	-0.612322566	-0.68065422	-0.723242824	-0.543363494
49.9529	MAP2K4	-0.666933372	-0.697651062	-0.73241601	-0.58396974
49.54982906	BRAF	-0.606096164	-0.750400812	-0.724306948	-0.473672896
49.05815507	CACNA2D	-0.761580777	-0.763648959	-0.729307045	-0.528912236
48.71918091	MAPKAPK	0.647125366	0.716167949	0.72958075	0.409254419
48.71475669	RASA1	-0.60883811	-0.702376036	-0.731340143	-0.39502695
48.46468028	MAPK9	-0.56368769	-0.705524485	-0.712430861	-0.580980102
48.39576275	PDGFRB	0.688908431	0.730297093	0.645157606	0.635085121
47.69240613	GNA12	0.710710614	0.736656752	0.672947789	0.680474357
47.12805511	RAPGEF2	-0.540278869	-0.645360989	-0.725061448	-0.354589315
46.67733357	PPP3CA	-0.533921002	-0.671667425	-0.6921142	-0.417615427
46.21744257	AKT1	0.570498577	0.672857974	0.696310575	0.47179211
46.14072209	CACNB2	-0.692029844	-0.650171935	-0.687755346	-0.565378394
46.12035778	KITLG	-0.554415335	-0.650219992	-0.747276559	-0.416693417
45.27312879	MECOM	0.578479305	0.734376213	0.58968412	0.592976285
45.21117001	PPP3R1	-0.711079349	-0.711486006	-0.644161708	-0.48997824
45.17908892	MAP3K11	0.563750452	0.664252141	0.772169958	0.378651418
45.05743947	MAPK10	-0.407247249	-0.634018634	-0.651069534	-0.535725506
45.05644011	MAPK8	-0.777053467	-0.74404825	-0.681128549	-0.592097882
44.74899075	BDNF	-0.670095256	-0.690765114	-0.65875316	-0.556595119
44.63081796	NLK	-0.695118169	-0.726114152	-0.673496393	-0.536507081
44.43469911	RASGRP1	-0.676077715	-0.711295464	-0.643684374	-0.420020081
44.39439423	PDGFD	-0.59154751	-0.672354394	-0.725757267	-0.529844413
44.36807343	TNFRSF1A	0.605008167	1	0.607388987	0.673690067
43.8465017	RPS6KA1	0.493048246	0.64949409	0.694812629	0.44426527
43.79379389	PRKACB	-0.531407256	-0.681088292	-0.650046856	-0.462126358
43.32635753	MEF2C	-0.762274045	-0.669299427	-0.742691117	-0.60669179
43.20119331	TGFB3	0.625767554	0.607388987	1	0.514475957
43.02589423	MAPK13	-0.696841323	-0.623483439	-0.632969789	-0.529284405
42.93146498	NFKB1	0.473123086	0.678737911	0.585607024	0.680189043
42.31966561	MAPK1	-0.714212087	-0.718651924	-0.65087028	-0.601693801
42.11794942	CSF1R	0.641252713	0.696267568	0.579638875	0.579142435
41.73279516	IGF1	-0.554759631	-0.673005197	-0.688715863	-0.422469619
41.67826043	NFATC3	0.484557496	0.504161416	0.649104823	0.35356857

41.47085249	MAP4K3	-0.37576389	-0.571467517	-0.638389261	-0.2851745
40.94267628	MAP4K4	0.649547853	0.54575889	0.838183552	0.512397688
40.16399845	KIT	-0.666625572	-0.630618608	-0.650274149	-0.535600385
39.47490645	HSPB1	0.490045	0.773436424	0.567577582	0.53338214
39.04665603	PPM1A	-0.270774309	-0.586777438	-0.54513592	-0.219908584
38.40625922	MAPT	-0.568017291	-0.499828995	-0.520659133	-0.586329037
38.29910071	KRAS	-0.328894392	-0.553419478	-0.469320622	-0.485789551
38.22025899	VEGFB	1	0.605008167	0.625767554	0.530604096
38.16946338	MAP4K2	0.432009095	0.407763487	0.581610295	0.364430104
37.28549456	CACNB4	-0.738475719	-0.590883015	-0.605026066	-0.539711916
36.63104924	LAMTOR:	-0.275826239	-0.459874497	-0.543628445	-0.146531413
36.45170728	FGFR3	0.408242805	0.396922004	0.486134017	0.373745885
36.32286543	MAPK14	0.398574618	0.396573803	0.522220536	0.252670984
34.62438369	MAP3K5	0.333605066	0.566812865	0.344540212	0.442813056
34.47858877	CACNA1C	-0.574995769	-0.452079348	-0.625418649	-0.33730996
34.31265689	STK3	0.530604096	0.673690067	0.514475957	1
34.24026191	CDC25B	0.324141934	0.567476569	0.501875875	0.209982303
33.63514466	EFNA1	0.247239466	0.659908704	0.582175555	0.672927895
32.60690735	FGF9	-0.723282185	-0.557584815	-0.546375915	-0.497128138
32.51662069	DAXX	0.425233154	0.504239464	0.513771995	0.093254778
32.32896066	FGF1	0.558356209	0.423313778	0.670847242	0.387697707
31.41870434	MET	-0.447459861	-0.565173646	-0.504178341	-0.592368526
31.16721424	FLNB	0.358467906	0.460959238	0.576749946	0.370615197
29.9571187	ANGPT2	0.407808201	0.616523499	0.402463534	0.544157388
29.93582246	MAPK3	0.319212803	0.366746155	0.425317007	0.228133382
29.68905122	CACNB1	-0.595080599	-0.510236741	-0.398056145	-0.629075941
29.44999021	RAF1	0.249246938	0.444414919	0.39222197	0.534680131
29.14073729	HSPA8	-0.076052657	-0.267977528	-0.384012714	-0.099656718
28.39430039	ELK1	0.12252351	0.420740675	0.298470448	0.239984574
27.17595572	CACNA1I	0.06608007	0.261551963	0.329449888	0.191565881
25.91287858	DUSP8	0.281766802	0.449219564	0.250742674	0.073079994
25.72920645	DUSP3	0.084446149	0.27284528	0.358683136	0.109138818
25.34129412	FGF2	0.475037527	0.571814025	0.255055534	0.722301227
24.92538031	CRK	0.512325013	0.437177117	0.313678451	0.276055014
22.93357098	CD14	0.274314659	0.641791789	0.233859527	0.478535431
18.98546667	HSPA1A	0.278751111	0.428260542	0.290218464	0.445948181
18.53935834	DUSP1	0.207905257	0.437521843	0.165430101	0.30629765

RPS6KA1	RASGRP1	RASA1	RAPGEF2	RAF1	PRKCB
-0.723344726	0.819802692	0.8674229	0.827649573	-0.474050613	0.938464299
-0.714262918	0.774124929	0.828046666	0.786526713	-0.573600699	0.912433154
-0.797859716	0.799101651	0.897049191	0.896004804	-0.442341262	1
-0.782121604	0.749388607	0.910376528	0.891819745	-0.45740358	0.943185559
-0.763179183	0.771336908	0.877835864	0.887598564	-0.416441756	0.955174208
-0.779649057	0.758747708	0.90544797	0.884025167	-0.381486364	0.881169653
-0.690150395	0.860747139	0.854476305	0.817010354	-0.359960835	0.891841254
0.824772578	-0.796648516	-0.903548419	-0.870323791	0.373452094	-0.90013872
-0.765175745	0.773831605	1	0.915715478	-0.366000142	0.897049191
-0.750635256	0.651027697	0.855575279	0.834440526	-0.509499074	0.886592296
0.652650417	-0.748906147	-0.747662089	-0.712479115	0.647218952	-0.836595925
0.651569824	-0.732625983	-0.763878046	-0.71675601	0.565926497	-0.843818848
-0.768541654	0.74696292	0.915715478	1	-0.308632737	0.896004804
-0.737869471	0.798580551	0.816134209	0.860923724	-0.465747313	0.842462836
0.759445861	-0.686438422	-0.80770624	-0.802801034	0.376527303	-0.783871547
-0.63213766	0.755388804	0.761978877	0.747569249	-0.489892948	0.838011216
-0.714598562	0.708400679	0.848514225	0.86370344	-0.371733371	0.812641854
0.652571694	-0.71238726	-0.705559477	-0.689164695	0.541012325	-0.824901649
-0.672991293	0.784386727	0.759679282	0.738725306	-0.376371249	0.862427974
0.806531772	-0.682832371	-0.855853818	-0.846588744	0.309829331	-0.826426195
-0.789635437	0.588118119	0.782217466	0.803108683	-0.482758782	0.838608054
-0.596451632	0.791015328	0.729436637	0.689183796	-0.343282858	0.795745929
-0.64374242	0.753402874	0.731967876	0.683472164	-0.420377542	0.782022591
-0.58911736	0.836394659	0.707419541	0.715630593	-0.398807192	0.75819189
-0.608349528	1	0.773831605	0.74696292	-0.305999367	0.799101651
-0.623162758	0.615185112	0.723755199	0.689381157	-0.459816724	0.735799243
0.64949409	-0.711295464	-0.702376036	-0.645360989	0.444414919	-0.733684119
1	-0.608349528	-0.765175745	-0.768541654	0.34414722	-0.797859716
-0.658287211	0.576147957	0.79006557	0.71189816	-0.531425787	0.742066617
-0.637909505	0.700211865	0.722368783	0.691475805	-0.299114874	0.80850705
0.694812629	-0.643684374	-0.731340143	-0.725061448	0.39222197	-0.759813553
-0.553611233	0.750184402	0.73888151	0.734681731	-0.264363153	0.830422416
0.634625161	-0.579984083	-0.635967566	-0.574657216	0.765239677	-0.720291591
-0.495179473	0.756808462	0.665266851	0.614672352	-0.403722825	0.742306262
0.732933603	-0.672285372	-0.640099069	-0.613140288	0.385526605	-0.724090342
-0.546048826	0.722824674	0.684512891	0.680938167	-0.366943766	0.684401376
0.74954637	-0.627501881	-0.782677987	-0.773925585	0.337418466	-0.78948526

-0.718100005	0.559322677	0.83391627	0.830509924	-0.350434144	0.766021787
0.602000643	-0.64031557	-0.639440986	-0.634154731	0.45030224	-0.686019617
-0.514139308	0.656112776	0.606483492	0.57400675	-0.376842267	0.692802027
0.467330993	-0.605113691	-0.643332437	-0.544074302	0.563357825	-0.606371947
-0.709799704	0.529270554	0.763731764	0.805046021	-0.336108823	0.678870752
-0.598033974	0.615896311	0.619815938	0.588299516	-0.386679169	0.760024875
-0.607643721	0.495825727	0.636406226	0.659134121	-0.575310897	0.679045504
0.493048246	-0.676077715	-0.60883811	-0.540278869	0.249246938	-0.689738457
0.747298069	-0.475838274	-0.654542551	-0.681786085	0.390350158	-0.689510008
-0.555369322	0.573235476	0.593244799	0.584663136	-0.242555619	0.657185835
-0.753201894	0.469927417	0.788289246	0.770722853	-0.217043894	0.696852595
0.532632024	-0.52982231	-0.670335614	-0.723105505	0.293460283	-0.718034286
0.76870077	-0.520434754	-0.716597374	-0.696557851	0.211619246	-0.721240948
0.386992043	-0.582624006	-0.546905909	-0.516069561	0.455415546	-0.572985288
-0.563348165	0.653332157	0.550584145	0.630028207	-0.091140033	0.670299029
0.44426527	-0.420020081	-0.39502695	-0.354589315	0.534680131	-0.563065519
0.611421616	-0.488514151	-0.662614811	-0.658460503	0.24340573	-0.558740675
0.545065877	-0.376021495	-0.478096766	-0.496008075	0.448945817	-0.579396077
-0.341232813	0.564270711	0.429070775	0.382161912	-0.310968249	0.549061655
0.548437767	-0.566754898	-0.689260935	-0.60482299	0.204719575	-0.556284094
0.447068702	-0.549982354	-0.540994005	-0.58805546	0.114500517	-0.577907549
-0.301682188	0.382033391	0.384130562	0.42645465	-0.463950052	0.462161965
0.400999238	-0.459316471	-0.482220445	-0.551807397	0.338303143	-0.583153981
0.489994857	-0.419791433	-0.356319111	-0.285959854	0.487776759	-0.430878761
0.585551543	-0.386372394	-0.528875575	-0.523680173	0.32045941	-0.538319706
-0.250715164	0.507467601	0.346273259	0.22930008	-0.51057175	0.482172179
0.34414722	-0.305999367	-0.366000142	-0.308632737	1	-0.442341262
-0.603125485	0.282193388	0.576173214	0.632863213	-0.157578933	0.520062298
0.556801539	-0.335093682	-0.518323143	-0.416375964	0.486365271	-0.425646353
0.521733773	-0.21081325	-0.514478104	-0.484090475	0.410824366	-0.416566098
0.290787194	-0.533872168	-0.531735086	-0.411561016	0.265840199	-0.357310962
0.521415569	-0.251321105	-0.551707626	-0.500327647	0.315601463	-0.432823181
0.132401709	-0.354201356	-0.240824786	-0.181762413	0.413181517	-0.367740128
0.270567235	-0.508232748	-0.36312805	-0.291444441	0.462438642	-0.417785536
0.454939051	-0.389452971	-0.254331903	-0.259965438	0.169213517	-0.325962197
0.115544224	-0.196998892	-0.217524371	-0.119562882	0.523189052	-0.234529645
0.207998167	-0.317764316	-0.22536087	-0.189812308	0.32489886	-0.240108512

PRKACB	PPP3R1	PPP3CB	PPP3CA	PPM1A	PDGFRB
0.733207848	0.853402389	1	0.776899805	0.632148118	-0.879315133
0.760968746	0.825634854	0.953291023	0.778094464	0.606688389	-0.886177268
0.742066617	0.862427974	0.938464299	0.842462836	0.678870752	-0.836595925
0.801373186	0.785074861	0.919952603	0.806054382	0.730979188	-0.816833531
0.753352802	0.782618129	0.932770142	0.798646706	0.693573312	-0.817949975
0.820602184	0.758157455	0.866750025	0.803893504	0.752607657	-0.763736432
0.68116253	0.850387109	0.92027585	0.801469487	0.624737649	-0.830440534
-0.715200463	-0.814987744	-0.833869914	-0.846600098	-0.69818157	0.755458783
0.79006557	0.759679282	0.8674229	0.816134209	0.763731764	-0.747662089
0.849228528	0.693724307	0.863174895	0.745594052	0.749278729	-0.779767601
-0.719580826	-0.804280274	-0.879315133	-0.776516936	-0.586682259	1
-0.709909851	-0.752955566	-0.858469738	-0.76252299	-0.519188156	0.843424386
0.71189816	0.738725306	0.827649573	0.860923724	0.805046021	-0.712479115
0.637850168	0.757110955	0.776899805	1	0.72308182	-0.776516936
-0.783023741	-0.633579376	-0.788245725	-0.764159329	-0.728592013	0.747658445
0.573228948	0.809561875	0.897937717	0.757505145	0.531114695	-0.832420974
0.741054491	0.651865349	0.773890222	0.860176725	0.716949625	-0.723604666
-0.614604982	-0.748835176	-0.817488809	-0.720245035	-0.545134126	0.83377568
0.565779536	1	0.853402389	0.757110955	0.532836856	-0.804280274
-0.710386707	-0.674537145	-0.764098037	-0.758822664	-0.68045932	0.64888198
0.7345614	0.642345018	0.802783944	0.699029001	0.771023778	-0.697663702
0.602126368	0.812483299	0.862013536	0.674836859	0.490968015	-0.757561789
0.613458895	0.732793794	0.818332212	0.686941199	0.470374315	-0.761597111
0.517912981	0.772726509	0.809690215	0.851756794	0.483416646	-0.791904934
0.576147957	0.784386727	0.819802692	0.798580551	0.529270554	-0.748906147
0.716018949	0.60239218	0.777935552	0.644464217	0.574856093	-0.738719451
-0.681088292	-0.711486006	-0.750550294	-0.671667425	-0.586777438	0.730297093
-0.658287211	-0.672991293	-0.723344726	-0.737869471	-0.709799704	0.652650417
1	0.565779536	0.733207848	0.637850168	0.710889859	-0.719580826
0.613475559	0.7124386	0.819213715	0.650428147	0.457493754	-0.68606949
-0.650046856	-0.644161708	-0.73715911	-0.6921142	-0.54513592	0.645157606
0.501656926	0.813023883	0.837424917	0.681710304	0.420652819	-0.737316884
-0.66195976	-0.665694146	-0.766798665	-0.678859297	-0.560375896	0.784478661
0.50877803	0.760564616	0.818401554	0.62148761	0.405185837	-0.774504894
-0.55957313	-0.703358057	-0.776199888	-0.643444166	-0.488017477	0.757846038
0.61918547	0.592264865	0.704384465	0.739535024	0.549269097	-0.676277585
-0.673712552	-0.588194727	-0.698891237	-0.732887887	-0.648399209	0.596675634

0.832098178	0.547816718	0.690390178	0.683621997	0.783655482	-0.582390207
-0.480912185	-0.652808717	-0.721254846	-0.689390355	-0.440806272	0.700783068
0.560331543	0.618243668	0.73862228	0.611545486	0.376083453	-0.72482854
-0.714929267	-0.556433629	-0.64992345	-0.551217254	-0.490594582	0.634205604
0.710889859	0.532836856	0.632148118	0.72308182	1	-0.586682259
0.50594361	0.676725139	0.77230856	0.552800633	0.402379135	-0.673743441
0.54446556	0.555026742	0.666982979	0.611747452	0.5912117	-0.665623174
-0.531407256	-0.711079349	-0.746511391	-0.533921002	-0.270774309	0.688908431
-0.679909329	-0.543024859	-0.633211514	-0.666687168	-0.657806882	0.629498887
0.561180024	0.514814779	0.693394112	0.519623559	0.372406162	-0.602824851
0.700473396	0.487971577	0.602721672	0.603385834	0.795104434	-0.476256387
-0.562480362	-0.552755118	-0.677754539	-0.647030947	-0.545649431	0.628309413
-0.610002179	-0.536599154	-0.605684415	-0.628987878	-0.573768867	0.498807308
-0.474374771	-0.583071916	-0.629575897	-0.561531413	-0.436288771	0.637211958
0.315720691	0.671199125	0.638740613	0.629257355	0.402965642	-0.555880503
-0.462126358	-0.48997824	-0.637183716	-0.417615427	-0.219908584	0.635085121
-0.606756242	-0.457406329	-0.516642349	-0.572859173	-0.624661043	0.482640596
-0.477299232	-0.435105978	-0.58486429	-0.461051	-0.447856327	0.505093266
0.337081729	0.6436427	0.628955014	0.463344764	0.182784153	-0.585704974
-0.475261341	-0.532036235	-0.514332875	-0.5857574	-0.51443786	0.486233421
-0.261760672	-0.527644497	-0.612251161	-0.550427283	-0.317712235	0.481001345
0.424459901	0.463824845	0.571804876	0.48834608	0.361362694	-0.606753502
-0.399310142	-0.459353352	-0.562865042	-0.543421369	-0.430809979	0.540653112
-0.451091724	-0.483495224	-0.524953264	-0.360218684	-0.352214255	0.576522961
-0.616811318	-0.368252702	-0.456634487	-0.52181912	-0.565115107	0.441166356
0.308509666	0.561122221	0.610803039	0.338082112	0.051981034	-0.621525043
-0.531425787	-0.376371249	-0.474050613	-0.465747313	-0.336108823	0.647218952
0.547785429	0.258656884	0.430731271	0.469580156	0.686168621	-0.363024897
-0.621373258	-0.285547208	-0.393809551	-0.436636647	-0.532669224	0.436935246
-0.605276935	-0.208743139	-0.372060009	-0.431027171	-0.525885176	0.379580997
-0.448310072	-0.452862953	-0.408198777	-0.488146932	-0.476297252	0.501653357
-0.601330756	-0.315582866	-0.331758807	-0.466835139	-0.607698317	0.331195022
-0.335080393	-0.394784206	-0.509085524	-0.235891338	-0.101141808	0.516735454
-0.304811331	-0.500514483	-0.425490764	-0.476538106	-0.186998558	0.575069291
-0.233152292	-0.349505764	-0.395072071	-0.312999849	-0.320513489	0.360762603
-0.43378169	-0.244707611	-0.30448604	-0.139131492	-0.178882263	0.310923597
-0.223343193	-0.326993888	-0.258494873	-0.360731493	-0.184165677	0.43014162

PDGFD	PAK1	NLK	NFKB1	NFATC3	MET
0.777935552	0.953291023	0.809690215	-0.766798665	-0.698891237	0.571804876
0.777199535	1	0.795102214	-0.810787632	-0.703603027	0.59971707
0.735799243	0.912433154	0.75819189	-0.720291591	-0.78948526	0.462161965
0.748148812	0.908625801	0.682378961	-0.714174104	-0.812054178	0.417558221
0.750203226	0.920407706	0.724860336	-0.682809147	-0.776799859	0.486776663
0.758507552	0.857048067	0.738144451	-0.640092676	-0.766270968	0.470610347
0.717244329	0.876074206	0.838593748	-0.669079061	-0.662923014	0.544224916
-0.702995881	-0.810482358	-0.728218229	0.649312032	0.85018517	-0.330261913
0.723755199	0.828046666	0.707419541	-0.635967566	-0.782677987	0.384130562
0.733287652	0.877711058	0.623322479	-0.711727794	-0.738624352	0.486374325
-0.738719451	-0.886177268	-0.791904934	0.784478661	0.596675634	-0.606753502
-0.723796317	-0.901484531	-0.773998748	0.744340696	0.670057674	-0.572795039
0.689381157	0.786526713	0.715630593	-0.574657216	-0.773925585	0.42645465
0.644464217	0.778094464	0.851756794	-0.678859297	-0.732887887	0.48834608
-0.766517191	-0.802816398	-0.703363972	0.620857693	0.70474365	-0.472789939
0.677207892	0.860317304	0.813379341	-0.715918612	-0.602132534	0.605471739
0.776199434	0.758829917	0.768491899	-0.593259506	-0.716129668	0.545288347
-0.692597633	-0.824920557	-0.706143522	0.712217799	0.612633081	-0.475210619
0.60239218	0.825634854	0.772726509	-0.665694146	-0.588194727	0.463824845
-0.680057715	-0.714568861	-0.64165602	0.545820762	0.7508516	-0.350647284
0.651246008	0.808566233	0.539878712	-0.720690936	-0.736615727	0.380521762
0.673307032	0.841849726	0.79669062	-0.632448814	-0.557842283	0.528693939
0.702682071	0.802681367	0.742307787	-0.602543024	-0.582451338	0.545600364
0.67264093	0.795102214	1	-0.648676026	-0.510528035	0.664756427
0.615185112	0.774124929	0.836394659	-0.579984083	-0.627501881	0.382033391
1	0.777199535	0.67264093	-0.649708749	-0.612392277	0.58539749
-0.672354394	-0.743502262	-0.726114152	0.678737911	0.504161416	-0.565173646
-0.623162758	-0.714262918	-0.58911736	0.634625161	0.74954637	-0.301682188
0.716018949	0.760968746	0.517912981	-0.66195976	-0.673712552	0.424459901
0.64705849	0.801584317	0.704204473	-0.587079468	-0.602351693	0.494948356
-0.725757267	-0.76122483	-0.673496393	0.585607024	0.649104823	-0.504178341
0.611088681	0.811649757	0.74612476	-0.516288548	-0.573061827	0.492030366
-0.649708749	-0.810787632	-0.648676026	1	0.575545434	-0.545456408
0.680410942	0.787054702	0.795471049	-0.623804392	-0.455078184	0.592026739
-0.618383861	-0.75743694	-0.716571926	0.621059976	0.510614169	-0.526154739
0.759708493	0.688718663	0.771202111	-0.558289584	-0.576015771	0.565875168
-0.612392277	-0.703603027	-0.510528035	0.575545434	1	-0.17875448

0.642070909	0.693210213	0.470947052	-0.530853662	-0.770865222	0.254078752
-0.670816376	-0.709808763	-0.754765045	0.616745925	0.518017408	-0.601699883
0.731464282	0.731200789	0.724655356	-0.532601566	-0.501251816	0.560055186
-0.643030466	-0.691035229	-0.576979296	0.672572897	0.503233138	-0.489423591
0.574856093	0.606688389	0.483416646	-0.560375896	-0.648399209	0.361362694
0.507463151	0.799823132	0.55637728	-0.632103514	-0.625493029	0.288514554
0.524639313	0.645630136	0.494116136	-0.636744938	-0.591465404	0.353846984
-0.59154751	-0.697228619	-0.695118169	0.473123086	0.484557496	-0.447459861
-0.607083649	-0.673564904	-0.475694459	0.578676254	0.72984235	-0.253291323
0.588615118	0.641361026	0.617455296	-0.484885331	-0.491785686	0.490495793
0.537922645	0.540317601	0.321841397	-0.447019744	-0.727940712	0.124917904
-0.516275669	-0.682070089	-0.515933913	0.445263635	0.588000214	-0.363524781
-0.502481047	-0.597858919	-0.375141191	0.442785401	0.889060536	-0.009269763
-0.532678	-0.664330466	-0.585410826	0.586801865	0.416868163	-0.433475508
0.484418934	0.597524033	0.634303688	-0.340216826	-0.511782002	0.359679761
-0.529844413	-0.706622161	-0.536507081	0.680189043	0.35356857	-0.592368526
-0.539111092	-0.451562295	-0.442024599	0.418358054	0.51049773	-0.240103176
-0.563659125	-0.622671297	-0.43178467	0.622594318	0.393434455	-0.446416572
0.457040548	0.60738066	0.67955108	-0.471561486	-0.246718216	0.589732506
-0.482037647	-0.429278502	-0.505294269	0.336896661	0.49419232	-0.175757523
-0.491147964	-0.568286358	-0.666599945	0.360535693	0.382378887	-0.519453483
0.58539749	0.59971707	0.664756427	-0.545456408	-0.17875448	1
-0.45764852	-0.529824593	-0.531817205	0.41259106	0.329409406	-0.479719099
-0.499560566	-0.544529911	-0.445602904	0.6315131	0.281689978	-0.37750087
-0.412574593	-0.48975527	-0.308540973	0.390133165	0.623165287	-0.188732276
0.472529482	0.617545301	0.539908638	-0.586306107	-0.19372792	0.488280868
-0.459816724	-0.573600699	-0.398807192	0.765239677	0.337418466	-0.463950052
0.426717691	0.408588783	0.19743299	-0.271567145	-0.558311713	0.089779683
-0.420364043	-0.437120864	-0.226706769	0.482441131	0.527152488	-0.121573636
-0.457871723	-0.433332762	-0.200163864	0.445482673	0.602932091	-0.143020668
-0.412496255	-0.407437973	-0.471896447	0.337622803	0.347062419	-0.234762624
-0.381701313	-0.403225912	-0.155283867	0.407184422	0.606634903	0.019527973
-0.466336357	-0.556752898	-0.449836165	0.504691833	0.125862292	-0.600388935
-0.318955009	-0.393415718	-0.507140873	0.486622067	0.282320087	-0.311082737
-0.383573369	-0.358606201	-0.429963008	0.412074343	0.155908514	-0.339073028
-0.290336283	-0.395975178	-0.185068954	0.525932555	0.18019596	-0.295504112
-0.182794684	-0.309140762	-0.441667921	0.341507148	0.142090986	-0.271933048

MEF2C	MECOM	MAPT	MAPKAPK2	MAPK9	MAPK8
0.819213715	-0.817488809	0.77230856	-0.833869914	0.863174895	0.862013536
0.801584317	-0.824920557	0.799823132	-0.810482358	0.877711058	0.841849726
0.80850705	-0.824901649	0.760024875	-0.90013872	0.886592296	0.795745929
0.739649693	-0.767872574	0.728409273	-0.870009306	0.940092956	0.72894455
0.790961643	-0.800360673	0.772751783	-0.839542811	0.922244758	0.759030112
0.710853111	-0.710108621	0.584475579	-0.880128269	0.884040511	0.766257458
0.832004552	-0.763556244	0.697076868	-0.849712605	0.753817163	0.8852259
-0.70173476	0.754456009	-0.647115024	1	-0.778059823	-0.744235093
0.722368783	-0.705559477	0.619815938	-0.903548419	0.855575279	0.729436637
0.705397168	-0.718917852	0.644458311	-0.778059823	1	0.669374071
-0.68606949	0.83377568	-0.673743441	0.755458783	-0.779767601	-0.757561789
-0.724047201	0.807858107	-0.716666113	0.773995901	-0.818364783	-0.748602517
0.691475805	-0.689164695	0.588299516	-0.870323791	0.834440526	0.689183796
0.650428147	-0.720245035	0.552800633	-0.846600098	0.745594052	0.674836859
-0.666633605	0.64105004	-0.534108629	0.762699197	-0.858277378	-0.655821843
0.728907508	-0.758952285	0.718454698	-0.756242674	0.686722536	0.836798216
0.65586354	-0.643591756	0.459455053	-0.796020668	0.79142886	0.651110677
-0.65536262	1	-0.701915238	0.754456009	-0.718917852	-0.694290027
0.7124386	-0.748835176	0.676725139	-0.814987744	0.693724307	0.812483299
-0.702388265	0.628420638	-0.46957372	0.855222174	-0.764224123	-0.658107249
0.608840854	-0.713711645	0.665998305	-0.757921866	0.887100248	0.602774749
0.790444499	-0.694290027	0.684271977	-0.744235093	0.669374071	1
0.722037163	-0.69477614	0.590816654	-0.73094696	0.72040973	0.739799585
0.704204473	-0.706143522	0.55637728	-0.728218229	0.623322479	0.79669062
0.700211865	-0.71238726	0.615896311	-0.796648516	0.651027697	0.791015328
0.64705849	-0.692597633	0.507463151	-0.702995881	0.733287652	0.673307032
-0.669299427	0.734376213	-0.499828995	0.716167949	-0.705524485	-0.74404825
-0.637909505	0.652571694	-0.598033974	0.824772578	-0.750635256	-0.596451632
0.613475559	-0.614604982	0.50594361	-0.715200463	0.849228528	0.602126368
1	-0.65536262	0.671480188	-0.70173476	0.705397168	0.790444499
-0.742691117	0.58968412	-0.520659133	0.72958075	-0.712430861	-0.681128549
0.753775498	-0.708930992	0.712749716	-0.746309027	0.679454064	0.787181591
-0.587079468	0.712217799	-0.632103514	0.649312032	-0.711727794	-0.632448814
0.751433218	-0.740724238	0.623827056	-0.658920631	0.558737524	0.850239276
-0.690369084	0.69718438	-0.640359575	0.653571955	-0.68093095	-0.710842293
0.588450804	-0.605052241	0.394046194	-0.686035347	0.640574638	0.652543205
-0.602351693	0.612633081	-0.625493029	0.85018517	-0.738624352	-0.557842283

0.529581	-0.549861583	0.44957102	-0.767486465	0.855708934	0.53149969
-0.66847551	0.596009455	-0.477559538	0.668525929	-0.554429332	-0.697165288
0.701923589	-0.626043994	0.571872306	-0.618403308	0.5967923	0.742154405
-0.543070502	0.592849325	-0.375826716	0.642052279	-0.627206553	-0.651075137
0.457493754	-0.545134126	0.402379135	-0.69818157	0.749278729	0.490968015
0.671480188	-0.701915238	1	-0.647115024	0.644458311	0.684271977
0.427847364	-0.671629586	0.526661544	-0.661636556	0.684502908	0.499454212
-0.762274045	0.578479305	-0.568017291	0.647125366	-0.56368769	-0.777053467
-0.505260246	0.536921582	-0.519806136	0.672885321	-0.758788577	-0.466618342
0.825798164	-0.531286259	0.494624671	-0.571484164	0.600940809	0.644233758
0.461746685	-0.458447134	0.354916089	-0.723285876	0.755007434	0.382049945
-0.473312761	0.574826986	-0.516746504	0.604527759	-0.761451352	-0.451981658
-0.494580752	0.528417557	-0.591031351	0.791190931	-0.677773735	-0.441224381
-0.294230406	0.632911336	-0.507710785	0.567400931	-0.558212177	-0.538915277
0.678302018	-0.516613485	0.558684612	-0.631372525	0.494217891	0.614878842
-0.60669179	0.592976285	-0.586329037	0.409254419	-0.580980102	-0.592097882
-0.456041781	0.432861317	-0.135929752	0.642912375	-0.56565655	-0.450836326
-0.479762296	0.592314553	-0.443391573	0.456773028	-0.627676362	-0.490932776
0.747838019	-0.52730391	0.496687257	-0.464725854	0.359636561	0.710732033
-0.454529804	0.435795931	-0.237104665	0.721782611	-0.420293922	-0.48179507
-0.606416675	0.451140478	-0.377082148	0.53119199	-0.416606274	-0.603914182
0.494948356	-0.475210619	0.288514554	-0.330261913	0.486374325	0.528693939
-0.543655648	0.519331285	-0.309308064	0.433815597	-0.514297631	-0.489254698
-0.483161688	0.613725256	-0.470965457	0.401478247	-0.40654851	-0.517644545
-0.364211039	0.374999976	-0.378517496	0.541988361	-0.671587257	-0.279742184
0.52943205	-0.561851701	0.562106018	-0.376321342	0.347180909	0.62943631
-0.299114874	0.541012325	-0.386679169	0.373452094	-0.509499074	-0.343282858
0.262507291	-0.348773628	0.250206672	-0.510119159	0.669652264	0.181289241
-0.235865026	0.382790475	-0.250696619	0.503944943	-0.584059458	-0.216056544
-0.141037977	0.306935956	-0.223035554	0.477196936	-0.601339417	-0.148674256
-0.116715659	0.410331397	-0.171351942	0.530175807	-0.356684199	-0.404883061
-0.120846379	0.356173086	-0.242431474	0.571227577	-0.56674026	-0.183274935
-0.405169987	0.500699269	-0.448129471	0.242142797	-0.399040101	-0.519007181
-0.391025634	0.484024811	-0.3393358	0.428163775	-0.245178833	-0.408570772
-0.322875825	0.402829931	-0.325815867	0.313170793	-0.285243221	-0.461037901
-0.265254399	0.296248439	-0.233631333	0.217899541	-0.298268231	-0.351052303
-0.188356052	0.327166751	-0.170251417	0.284251288	-0.199121838	-0.280893361

MAPK3	MAPK14	MAPK13	MAPK10	MAPK1	MAP4K4
-0.456634487	-0.605684415	0.837424917	0.802783944	0.818401554	-0.721254846
-0.48975527	-0.597858919	0.811649757	0.808566233	0.787054702	-0.709808763
-0.538319706	-0.721240948	0.830422416	0.838608054	0.742306262	-0.686019617
-0.609741011	-0.75384384	0.75602701	0.876154772	0.63896411	-0.605981589
-0.594513982	-0.70133763	0.800059277	0.846442215	0.686358186	-0.617625359
-0.563487242	-0.687649911	0.723329583	0.813365664	0.664997651	-0.614139993
-0.384309934	-0.546052444	0.840094484	0.686202055	0.851853235	-0.732234043
0.541988361	0.791190931	-0.746309027	-0.757921866	-0.658920631	0.668525929
-0.528875575	-0.716597374	0.73888151	0.782217466	0.665266851	-0.639440986
-0.671587257	-0.677773735	0.679454064	0.887100248	0.558737524	-0.554429332
0.441166356	0.498807308	-0.737316884	-0.697663702	-0.774504894	0.700783068
0.553643255	0.583021131	-0.755330355	-0.696124386	-0.679167376	0.639396312
-0.523680173	-0.696557851	0.734681731	0.803108683	0.614672352	-0.634154731
-0.52181912	-0.628987878	0.681710304	0.699029001	0.62148761	-0.689390355
0.636612894	0.640747461	-0.595183778	-0.756107595	-0.534816443	0.583226664
-0.249196971	-0.498655452	0.807726689	0.669564088	0.844526202	-0.771348413
-0.546128579	-0.618090113	0.645611733	0.695901707	0.592321323	-0.686667958
0.374999976	0.528417557	-0.708930992	-0.713711645	-0.740724238	0.596009455
-0.368252702	-0.536599154	0.813023883	0.642345018	0.760564616	-0.652808717
0.437115722	0.700106472	-0.656606233	-0.746079292	-0.614353908	0.714735921
-0.581655983	-0.689081801	0.602243715	1	0.511856214	-0.543227865
-0.279742184	-0.441224381	0.787181591	0.602774749	0.850239276	-0.697165288
-0.436117409	-0.519042378	0.783613346	0.616336168	0.7202537	-0.660514318
-0.308540973	-0.375141191	0.74612476	0.539878712	0.795471049	-0.754765045
-0.386372394	-0.520434754	0.750184402	0.588118119	0.756808462	-0.64031557
-0.412574593	-0.502481047	0.611088681	0.651246008	0.680410942	-0.670816376
0.366746155	0.396573803	-0.623483439	-0.634018634	-0.718651924	0.54575889
0.585551543	0.76870077	-0.553611233	-0.789635437	-0.495179473	0.602000643
-0.616811318	-0.610002179	0.501656926	0.7345614	0.50877803	-0.480912185
-0.364211039	-0.494580752	0.753775498	0.608840854	0.751433218	-0.66847551
0.425317007	0.522220536	-0.632969789	-0.651069534	-0.65087028	0.838183552
-0.316629696	-0.539182451	1	0.602243715	0.7635539	-0.657315833
0.390133165	0.442785401	-0.516288548	-0.720690936	-0.623804392	0.616745925
-0.068891384	-0.30244961	0.7635539	0.511856214	1	-0.736194245
0.468185447	0.48362118	-0.67362375	-0.614049622	-0.650297286	0.573174272
-0.373151321	-0.429242706	0.560424626	0.526042146	0.651745193	-0.642322673
0.623165287	0.889060536	-0.573061827	-0.736615727	-0.455078184	0.518017408

-0.624704147	-0.726315462	0.509671742	0.796579893	0.395249508	-0.412836773
0.25184401	0.396853155	-0.657315833	-0.543227865	-0.736194245	1
-0.270193108	-0.39655622	0.656492771	0.475893975	0.723556383	-0.621692299
0.304875672	0.358078977	-0.476483662	-0.545989033	-0.678475642	0.513161135
-0.565115107	-0.573768867	0.420652819	0.771023778	0.405185837	-0.440806272
-0.378517496	-0.591031351	0.712749716	0.665998305	0.623827056	-0.477559538
-0.355434552	-0.553304481	0.526913856	0.773192974	0.503226879	-0.526889647
0.319212803	0.398574618	-0.696841323	-0.407247249	-0.714212087	0.649547853
0.684305942	0.706579974	-0.428803241	-0.714095529	-0.308916884	0.443603126
-0.370555674	-0.396026103	0.608918884	0.486930837	0.625808852	-0.604499292
-0.609222541	-0.750673534	0.425649868	0.768953941	0.275668538	-0.389731165
0.572976177	0.589170307	-0.626187108	-0.666891022	-0.324422633	0.443824608
0.645030821	1	-0.539182451	-0.689081801	-0.30244961	0.396853155
0.336183055	0.355077334	-0.552750175	-0.527279496	-0.53232301	0.409842
-0.323142156	-0.451025868	0.715206156	0.462318513	0.570132481	-0.575720763
0.228133382	0.252670984	-0.529284405	-0.535725506	-0.601693801	0.512397688
0.329747412	0.489686045	-0.391004341	-0.558683092	-0.416268184	0.491832485
0.226315043	0.311855732	-0.432497829	-0.67100305	-0.473499812	0.479540923
-0.07089842	-0.11867418	0.645695664	0.260086515	0.77272764	-0.635297233
0.218335821	0.457075771	-0.464421417	-0.422717659	-0.519655033	0.582817766
0.094782576	0.291254595	-0.648886573	-0.396362589	-0.645005569	0.806912737
-0.188732276	-0.009269763	0.492030366	0.380521762	0.592026739	-0.601699883
0.13821484	0.198664674	-0.496115369	-0.525572869	-0.567273651	0.597861663
0.202919216	0.186794931	-0.319270007	-0.439653012	-0.544093109	0.404616775
1	0.645030821	-0.316629696	-0.581655983	-0.068891384	0.25184401
0.025238772	-0.104811596	0.554889671	0.290641668	0.727815695	-0.51457968
0.32045941	0.211619246	-0.264363153	-0.482758782	-0.403722825	0.45030224
-0.627987748	-0.609816179	0.289064994	0.695530506	0.036414539	-0.215536971
0.651987407	0.572810271	-0.220366359	-0.553350641	-0.127195195	0.225396672
0.631692283	0.61621789	-0.144768214	-0.5866199	-0.046710285	0.232324875
0.198799347	0.285101509	-0.317503056	-0.287319447	-0.404127581	0.318010742
0.600823082	0.636689339	-0.162969217	-0.559793599	-0.017311206	0.156406522
0.139301106	0.024609506	-0.432037078	-0.252891099	-0.510448372	0.280121932
0.101585811	0.154628412	-0.383809972	-0.152268103	-0.519159927	0.446196351
0.147611321	0.114637171	-0.268391268	-0.366383679	-0.413987678	0.276179998
0.056831853	0.038350206	-0.138047093	-0.25776113	-0.410776043	0.246231922
0.088486604	0.094514353	-0.227368224	-0.158459059	-0.346780504	0.246111939

MAP4K3	MAP4K2	MAP3K5	MAP3K11	MAP2K4	MAP2K1
0.690390178	-0.633211514	-0.629575897	-0.764098037	0.932770142	0.919952603
0.693210213	-0.673564904	-0.664330466	-0.714568861	0.920407706	0.908625801
0.766021787	-0.689510008	-0.572985288	-0.826426195	0.955174208	0.943185559
0.837967095	-0.769920387	-0.594509913	-0.806977624	0.952866705	1
0.784907697	-0.725755398	-0.579141209	-0.758347919	1	0.952866705
0.864043576	-0.714103137	-0.580283864	-0.852948302	0.875178923	0.906727111
0.634478961	-0.544734019	-0.570035226	-0.770681556	0.851240655	0.830592211
-0.767486465	0.672885321	0.567400931	0.855222174	-0.839542811	-0.870009306
0.83391627	-0.654542551	-0.546905909	-0.855853818	0.877835864	0.910376528
0.855708934	-0.758788577	-0.558212177	-0.764224123	0.922244758	0.940092956
-0.582390207	0.629498887	0.637211958	0.64888198	-0.817949975	-0.816833531
-0.610034221	0.610857231	0.674596439	0.614370982	-0.861712533	-0.829493875
0.830509924	-0.681786085	-0.516069561	-0.846588744	0.887598564	0.891819745
0.683621997	-0.666687168	-0.561531413	-0.758822664	0.798646706	0.806054382
-0.817884361	0.814270244	0.470221553	0.764667207	-0.82273276	-0.848265842
0.517397557	-0.45965372	-0.592946496	-0.669507263	0.79446612	0.771100505
0.785960182	-0.69745972	-0.466809064	-0.813087441	0.807673259	0.812067163
-0.549861583	0.536921582	0.632911336	0.628420638	-0.800360673	-0.767872574
0.547816718	-0.543024859	-0.583071916	-0.674537145	0.782618129	0.785074861
-0.799787377	0.635334355	0.351161295	1	-0.758347919	-0.806977624
0.796579893	-0.714095529	-0.527279496	-0.746079292	0.846442215	0.876154772
0.53149969	-0.466618342	-0.538915277	-0.658107249	0.759030112	0.72894455
0.552880191	-0.480284539	-0.552147213	-0.683639623	0.772266356	0.750344688
0.470947052	-0.475694459	-0.585410826	-0.64165602	0.724860336	0.682378961
0.559322677	-0.475838274	-0.582624006	-0.682832371	0.771336908	0.749388607
0.642070909	-0.607083649	-0.532678	-0.680057715	0.750203226	0.748148812
-0.571467517	0.407763487	0.566812865	0.664252141	-0.697651062	-0.68065422
-0.718100005	0.747298069	0.386992043	0.806531772	-0.763179183	-0.782121604
0.832098178	-0.679909329	-0.474374771	-0.710386707	0.753352802	0.801373186
0.529581	-0.505260246	-0.294230406	-0.702388265	0.790961643	0.739649693
-0.638389261	0.581610295	0.344540212	0.772169958	-0.73241601	-0.723242824
0.509671742	-0.428803241	-0.552750175	-0.656606233	0.800059277	0.75602701
-0.530853662	0.578676254	0.586801865	0.545820762	-0.682809147	-0.714174104
0.395249508	-0.308916884	-0.53232301	-0.614353908	0.686358186	0.63896411
-0.479164096	0.554929855	0.525860539	0.549921049	-0.744706243	-0.697969235
0.578519008	-0.499650769	-0.480415024	-0.66927628	0.66371841	0.658999058
-0.770865222	0.72984235	0.416868163	0.7508516	-0.776799859	-0.812054178

1	-0.758330115	-0.413315572	-0.799787377	0.784907697	0.837967095
-0.412836773	0.443603126	0.409842	0.714735921	-0.617625359	-0.605981589
0.439014919	-0.425867688	-0.435186905	-0.56665487	0.671583202	0.632466916
-0.556701558	0.333627691	0.583982607	0.587865763	-0.562926879	-0.607688422
0.783655482	-0.657806882	-0.436288771	-0.68045932	0.693573312	0.730979188
0.44957102	-0.519806136	-0.507710785	-0.46957372	0.772751783	0.728409273
0.578811926	-0.487266171	-0.584735503	-0.626461263	0.660403095	0.697969161
-0.37576389	0.432009095	0.333605066	0.563750452	-0.666933372	-0.612322566
-0.758330115	1	0.344531885	0.635334355	-0.725755398	-0.769920387
0.432722976	-0.449118355	-0.208030617	-0.621631196	0.660395498	0.602836655
0.85188115	-0.669881143	-0.294300478	-0.790180043	0.685504453	0.760973299
-0.649823115	0.651093179	0.516416343	0.569413182	-0.774674917	-0.778841453
-0.726315462	0.706579974	0.355077334	0.700106472	-0.70133763	-0.75384384
-0.413315572	0.344531885	1	0.351161295	-0.579141209	-0.594509913
0.378687913	-0.446602649	-0.292118949	-0.552310699	0.645428713	0.589229301
-0.2851745	0.364430104	0.442813056	0.378651418	-0.58396974	-0.543363494
-0.676856601	0.478289173	0.269852927	0.792376174	-0.4874065	-0.573691252
-0.510654828	0.407211356	0.361655757	0.554842713	-0.58244629	-0.570371891
0.122126197	-0.12835656	-0.282154215	-0.393038425	0.484055193	0.399938205
-0.506228719	0.306592487	0.320707863	0.768503781	-0.439997295	-0.515429966
-0.302265581	0.240439606	0.322875449	0.585359692	-0.553163549	-0.494395552
0.254078752	-0.253291323	-0.433475508	-0.350647284	0.486776663	0.417558221
-0.425007633	0.304813559	0.215630348	0.585245652	-0.551191413	-0.498317754
-0.243826831	0.336452727	0.336375502	0.335276624	-0.406513513	-0.402181813
-0.624704147	0.684305942	0.336183055	0.437115722	-0.594513982	-0.609741011
0.067847001	-0.118014626	-0.443569468	-0.251009804	0.441570684	0.399465542
-0.350434144	0.390350158	0.455415546	0.309829331	-0.416441756	-0.45740358
0.764601529	-0.682840124	-0.25325366	-0.582878209	0.584429846	0.633422484
-0.59205723	0.516842045	0.38392321	0.459511296	-0.441207949	-0.523551134
-0.668617502	0.665137676	0.39677328	0.476318741	-0.455351608	-0.567696917
-0.393111438	0.254278803	0.637371562	0.349931275	-0.329856305	-0.408171742
-0.69184617	0.679301802	0.368533248	0.448807567	-0.445239408	-0.569319812
-0.092452127	0.129975305	0.548577854	0.05695296	-0.438973862	-0.360583536
-0.084206215	0.118824732	0.350522403	0.286911937	-0.32645045	-0.310631272
-0.172598981	0.205877109	0.308320719	0.259124038	-0.326044664	-0.267038236
-0.184378044	0.087139786	0.320429039	0.163942291	-0.20392654	-0.241718074
-0.08969154	0.13578905	0.289891406	0.200224434	-0.194910887	-0.203991342

LAMTOR3	KRAS	KITLG	KIT	IGF1	HSPB1
0.602721672	0.666982979	0.773890222	0.73862228	0.704384465	-0.64992345
0.540317601	0.645630136	0.758829917	0.731200789	0.688718663	-0.691035229
0.696852595	0.679045504	0.812641854	0.692802027	0.684401376	-0.606371947
0.760973299	0.697969161	0.812067163	0.632466916	0.658999058	-0.607688422
0.685504453	0.660403095	0.807673259	0.671583202	0.66371841	-0.562926879
0.744920722	0.646452581	0.862907717	0.648459696	0.737253417	-0.689822972
0.532279545	0.56274154	0.756922644	0.722966244	0.704394654	-0.641488944
-0.723285876	-0.661636556	-0.796020668	-0.618403308	-0.686035347	0.642052279
0.788289246	0.636406226	0.848514225	0.606483492	0.684512891	-0.643332437
0.755007434	0.684502908	0.79142886	0.5967923	0.640574638	-0.627206553
-0.476256387	-0.665623174	-0.723604666	-0.72482854	-0.676277585	0.634205604
-0.444919893	-0.614214399	-0.711389514	-0.658704303	-0.630311758	0.669056376
0.770722853	0.659134121	0.86370344	0.57400675	0.680938167	-0.544074302
0.603385834	0.611747452	0.860176725	0.611545486	0.739535024	-0.551217254
-0.71044664	-0.493148842	-0.839873886	-0.625570675	-0.738843452	0.561930067
0.43273762	0.648676568	0.686080489	0.708161131	0.64228773	-0.578909309
0.694782702	0.542768248	1	0.655958278	0.794405844	-0.556018365
-0.458447134	-0.671629586	-0.643591756	-0.626043994	-0.605052241	0.592849325
0.487971577	0.555026742	0.651865349	0.618243668	0.592264865	-0.556433629
-0.790180043	-0.626461263	-0.813087441	-0.56665487	-0.66927628	0.587865763
0.768953941	0.773192974	0.695901707	0.475893975	0.526042146	-0.545989033
0.382049945	0.499454212	0.651110677	0.742154405	0.652543205	-0.651075137
0.497762434	0.538843406	0.699587952	0.64828244	0.64790607	-0.600744353
0.321841397	0.494116136	0.768491899	0.724655356	0.771202111	-0.576979296
0.469927417	0.495825727	0.708400679	0.656112776	0.722824674	-0.605113691
0.537922645	0.524639313	0.776199434	0.731464282	0.759708493	-0.643030466
-0.459874497	-0.553419478	-0.650219992	-0.630618608	-0.673005197	0.773436424
-0.753201894	-0.607643721	-0.714598562	-0.514139308	-0.546048826	0.467330993
0.700473396	0.54446556	0.741054491	0.560331543	0.61918547	-0.714929267
0.461746685	0.427847364	0.65586354	0.701923589	0.588450804	-0.543070502
-0.543628445	-0.469320622	-0.747276559	-0.650274149	-0.688715863	0.567577582
0.425649868	0.526913856	0.645611733	0.656492771	0.560424626	-0.476483662
-0.447019744	-0.636744938	-0.593259506	-0.532601566	-0.558289584	0.672572897
0.275668538	0.503226879	0.592321323	0.723556383	0.651745193	-0.678475642
-0.409243702	-0.492809062	-0.601216613	-0.603597911	-0.523581598	0.483521727
0.480544233	0.434570871	0.794405844	0.765480236	1	-0.646622506
-0.727940712	-0.591465404	-0.716129668	-0.501251816	-0.576015771	0.503233138

0.85188115	0.578811926	0.785960182	0.439014919	0.578519008	-0.556701558
-0.389731165	-0.526889647	-0.686667958	-0.621692299	-0.642322673	0.513161135
0.322859626	0.409574775	0.655958278	1	0.765480236	-0.560143444
-0.411218047	-0.505090121	-0.556018365	-0.560143444	-0.646622506	1
0.795104434	0.5912117	0.716949625	0.376083453	0.549269097	-0.490594582
0.354916089	0.526661544	0.459455053	0.571872306	0.394046194	-0.375826716
0.568888995	1	0.542768248	0.409574775	0.434570871	-0.505090121
-0.275826239	-0.328894392	-0.554415335	-0.666625572	-0.554759631	0.490045
-0.669881143	-0.487266171	-0.69745972	-0.425867688	-0.499650769	0.333627691
0.402767111	0.357320426	0.576832726	0.601169306	0.531845492	-0.480289392
1	0.568888995	0.694782702	0.322859626	0.480544233	-0.411218047
-0.575431655	-0.543571524	-0.648038868	-0.42554866	-0.448976665	0.267540838
-0.750673534	-0.553304481	-0.618090113	-0.39655622	-0.429242706	0.358078977
-0.294300478	-0.584735503	-0.466809064	-0.435186905	-0.480415024	0.583982607
0.37590156	0.311248912	0.529589705	0.587105358	0.548197398	-0.242780595
-0.146531413	-0.485789551	-0.416693417	-0.535600385	-0.422469619	0.53338214
-0.719617577	-0.535984349	-0.660294507	-0.362824419	-0.56182708	0.55646938
-0.430301682	-0.537392082	-0.502883431	-0.445278801	-0.470166204	0.47916178
0.019725958	0.227312572	0.365006237	0.618065095	0.457403622	-0.489856154
-0.598091701	-0.469990331	-0.561668777	-0.407410544	-0.522003584	0.494645311
-0.306643174	-0.417614713	-0.575382153	-0.534088125	-0.535952083	0.359927279
0.124917904	0.353846984	0.545288347	0.560055186	0.565875168	-0.489423591
-0.384004665	-0.533545914	-0.556552099	-0.486944629	-0.520392899	0.357284539
-0.19818127	-0.362677603	-0.334830861	-0.455474453	-0.389750993	0.516797788
-0.609222541	-0.355434552	-0.546128579	-0.270193108	-0.373151321	0.304875672
-0.009735792	0.293331341	0.265970091	0.584563454	0.388448503	-0.502072621
-0.217043894	-0.575310897	-0.371733371	-0.376842267	-0.366943766	0.563357825
0.808478016	0.530930433	0.570440555	0.17155696	0.327245941	-0.146111274
-0.653385192	-0.49153468	-0.473086744	-0.238968907	-0.363600342	0.50066118
-0.664935768	-0.484751508	-0.548065974	-0.210697487	-0.38331265	0.430372802
-0.318338444	-0.365599581	-0.448881754	-0.299502472	-0.479990222	0.527830486
-0.630379416	-0.412923801	-0.500964264	-0.092119779	-0.290815135	0.362846668
0.093555822	-0.251508554	-0.227564926	-0.493698658	-0.33177086	0.477854684
-0.073998757	-0.243277238	-0.274302809	-0.418477177	-0.391745577	0.393991368
-0.166337244	-0.310040854	-0.290662571	-0.339681384	-0.342044682	0.336734799
-0.026489544	-0.201720869	-0.09573329	-0.238848533	-0.237351001	0.735876071
-0.028299621	-0.240411585	-0.232507102	-0.335477028	-0.338195908	0.401696919

HSPA8	HSPA1A	GNA12	FLNB	FGFR3	FGF9
0.430731271	-0.30448604	-0.858469738	-0.562865042	-0.677754539	0.628955014
0.408588783	-0.395975178	-0.901484531	-0.529824593	-0.682070089	0.60738066
0.520062298	-0.234529645	-0.843818848	-0.583153981	-0.718034286	0.549061655
0.633422484	-0.241718074	-0.829493875	-0.498317754	-0.778841453	0.399938205
0.584429846	-0.20392654	-0.861712533	-0.551191413	-0.774674917	0.484055193
0.607070234	-0.255749355	-0.7695523	-0.52038992	-0.676155601	0.429042521
0.333970781	-0.276548287	-0.79488071	-0.570786922	-0.581956851	0.723366769
-0.510119159	0.217899541	0.773995901	0.433815597	0.604527759	-0.464725854
0.576173214	-0.217524371	-0.763878046	-0.482220445	-0.670335614	0.429070775
0.669652264	-0.298268231	-0.818364783	-0.514297631	-0.761451352	0.359636561
-0.363024897	0.310923597	0.843424386	0.540653112	0.628309413	-0.585704974
-0.353100537	0.36476695	1	0.403486634	0.701892597	-0.582680973
0.632863213	-0.119562882	-0.71675601	-0.551807397	-0.723105505	0.382161912
0.469580156	-0.139131492	-0.76252299	-0.543421369	-0.647030947	0.463344764
-0.649715039	0.162409576	0.725604991	0.434801636	0.684363462	-0.317717051
0.213108822	-0.301079593	-0.775434603	-0.565088206	-0.525796024	0.69298979
0.570440555	-0.09573329	-0.711389514	-0.556552099	-0.648038868	0.365006237
-0.348773628	0.296248439	0.807858107	0.519331285	0.574826986	-0.52730391
0.258656884	-0.244707611	-0.752955566	-0.459353352	-0.552755118	0.6436427
-0.582878209	0.163942291	0.614370982	0.585245652	0.569413182	-0.393038425
0.695530506	-0.25776113	-0.696124386	-0.525572869	-0.666891022	0.260086515
0.181289241	-0.351052303	-0.748602517	-0.489254698	-0.451981658	0.710732033
0.336206236	-0.209217594	-0.757128838	-0.470449968	-0.533303112	0.568126754
0.19743299	-0.185068954	-0.773998748	-0.531817205	-0.515933913	0.67955108
0.282193388	-0.196998892	-0.732625983	-0.459316471	-0.52982231	0.564270711
0.426717691	-0.290336283	-0.723796317	-0.45764852	-0.516275669	0.457040548
-0.267977528	0.428260542	0.736656752	0.460959238	0.396922004	-0.557584815
-0.603125485	0.115544224	0.651569824	0.400999238	0.532632024	-0.341232813
0.547785429	-0.43378169	-0.709909851	-0.399310142	-0.562480362	0.337081729
0.262507291	-0.265254399	-0.724047201	-0.543655648	-0.473312761	0.747838019
-0.384012714	0.290218464	0.672947789	0.576749946	0.486134017	-0.546375915
0.289064994	-0.138047093	-0.755330355	-0.496115369	-0.626187108	0.645695664
-0.271567145	0.525932555	0.744340696	0.41259106	0.445263635	-0.471561486
0.036414539	-0.410776043	-0.679167376	-0.567273651	-0.324422633	0.77272764
-0.316401228	0.196047345	0.767023965	0.386072262	0.487114602	-0.616763411
0.327245941	-0.237351001	-0.630311758	-0.520392899	-0.448976665	0.457403622
-0.558311713	0.18019596	0.670057674	0.329409406	0.588000214	-0.246718216

0.764601529	-0.184378044	-0.610034221	-0.425007633	-0.649823115	0.122126197
-0.215536971	0.246231922	0.639396312	0.597861663	0.443824608	-0.635297233
0.17155696	-0.238848533	-0.658704303	-0.486944629	-0.42554866	0.618065095
-0.146111274	0.735876071	0.669056376	0.357284539	0.267540838	-0.489856154
0.686168621	-0.178882263	-0.519188156	-0.430809979	-0.545649431	0.182784153
0.250206672	-0.233631333	-0.716666113	-0.309308064	-0.516746504	0.496687257
0.530930433	-0.201720869	-0.614214399	-0.533545914	-0.543571524	0.227312572
-0.076052657	0.278751111	0.710710614	0.358467906	0.408242805	-0.723282185
-0.682840124	0.087139786	0.610857231	0.304813559	0.651093179	-0.12835656
0.262666244	-0.186225248	-0.628583902	-0.447883983	-0.41086039	0.635356902
0.808478016	-0.026489544	-0.444919893	-0.384004665	-0.575431655	0.019725958
-0.659281948	-0.028739962	0.701892597	0.404565462	1	-0.181632724
-0.609816179	0.038350206	0.583021131	0.198664674	0.589170307	-0.11867418
-0.25325366	0.320429039	0.674596439	0.215630348	0.516416343	-0.282154215
0.300696421	0.07417993	-0.517580033	-0.46925698	-0.429852278	0.568946099
-0.099656718	0.445948181	0.680474357	0.370615197	0.373745885	-0.497128138
-0.562133923	0.121949722	0.372833004	0.451970595	0.366958619	-0.178851229
-0.410237989	0.284805943	0.505949895	0.505581241	0.409606704	-0.250390777
-0.201867937	-0.374212235	-0.582680973	-0.429629075	-0.181632724	1
-0.342893091	0.07314689	0.374385011	0.366412333	0.289940516	-0.352519774
-0.201225009	0.03212308	0.522629759	0.602019465	0.433187933	-0.551260752
0.089779683	-0.295504112	-0.572795039	-0.479719099	-0.363524781	0.589732506
-0.338119878	0.134917124	0.403486634	1	0.404565462	-0.429629075
-0.072595234	0.411369994	0.486889359	0.251759012	0.07264513	-0.473315761
-0.627987748	0.056831853	0.553643255	0.13821484	0.572976177	-0.07089842
-0.211657111	-0.457074737	-0.566368368	-0.307517759	-0.130904101	0.673008232
-0.157578933	0.523189052	0.565926497	0.338303143	0.293460283	-0.310968249
1	0.232478941	-0.353100537	-0.338119878	-0.659281948	-0.201867937
-0.557799207	0.18775615	0.451227179	0.116258013	0.357022596	0.026054047
-0.647070331	0.173837439	0.407780587	0.100339101	0.474537254	0.229558364
-0.180178172	0.235215162	0.413771163	0.089630346	0.270848756	-0.166315655
-0.615524312	0.184426521	0.395782379	0.04030661	0.479642926	0.184007048
0.099259144	0.427610155	0.607883545	0.142328413	0.295085271	-0.490030418
0.190849354	0.31267058	0.460851	0.192636949	0.135527892	-0.549914765
-0.129813189	0.134696507	0.331733809	0.203345568	0.012526344	-0.284537227
0.232478941	1	0.36476695	0.134917124	-0.028739962	-0.374212235
0.053687345	0.268852978	0.318416666	0.209347857	0.104586107	-0.282834351

FGF2	FGF1	ELK1	EFNA1	DUSP8	DUSP3
-0.509085524	-0.612251161	-0.393809551	-0.58486429	-0.408198777	-0.331758807
-0.556752898	-0.568286358	-0.437120864	-0.622671297	-0.407437973	-0.403225912
-0.367740128	-0.577907549	-0.425646353	-0.579396077	-0.357310962	-0.432823181
-0.360583536	-0.494395552	-0.523551134	-0.570371891	-0.408171742	-0.569319812
-0.438973862	-0.553163549	-0.441207949	-0.58244629	-0.329856305	-0.445239408
-0.321607039	-0.517498248	-0.528698803	-0.529577112	-0.528057412	-0.563793227
-0.431374166	-0.640642373	-0.291750517	-0.472679558	-0.479426673	-0.270529361
0.242142797	0.53119199	0.503944943	0.456773028	0.530175807	0.571227577
-0.240824786	-0.540994005	-0.518323143	-0.478096766	-0.531735086	-0.551707626
-0.399040101	-0.416606274	-0.584059458	-0.627676362	-0.356684199	-0.56674026
0.516735454	0.481001345	0.436935246	0.505093266	0.501653357	0.331195022
0.607883545	0.522629759	0.451227179	0.505949895	0.413771163	0.395782379
-0.181762413	-0.58805546	-0.416375964	-0.496008075	-0.411561016	-0.500327647
-0.235891338	-0.550427283	-0.436636647	-0.461051	-0.488146932	-0.466835139
0.285120275	0.442910321	0.558093056	0.541718501	0.409218307	0.519172823
-0.44168599	-0.647252494	-0.249389114	-0.478058256	-0.426821561	-0.189070116
-0.227564926	-0.575382153	-0.473086744	-0.502883431	-0.448881754	-0.500964264
0.500699269	0.451140478	0.382790475	0.592314553	0.410331397	0.356173086
-0.394784206	-0.527644497	-0.285547208	-0.435105978	-0.452862953	-0.315582866
0.05695296	0.585359692	0.459511296	0.554842713	0.349931275	0.448807567
-0.252891099	-0.396362589	-0.553350641	-0.67100305	-0.287319447	-0.559793599
-0.519007181	-0.603914182	-0.216056544	-0.490932776	-0.404883061	-0.183274935
-0.446372218	-0.569903414	-0.45367842	-0.466143562	-0.377713796	-0.223883068
-0.449836165	-0.666599945	-0.226706769	-0.43178467	-0.471896447	-0.155283867
-0.354201356	-0.549982354	-0.335093682	-0.376021495	-0.533872168	-0.251321105
-0.466336357	-0.491147964	-0.420364043	-0.563659125	-0.412496255	-0.381701313
0.571814025	0.423313778	0.420740675	0.659908704	0.449219564	0.27284528
0.132401709	0.447068702	0.556801539	0.545065877	0.290787194	0.521415569
-0.335080393	-0.261760672	-0.621373258	-0.477299232	-0.448310072	-0.601330756
-0.405169987	-0.606416675	-0.235865026	-0.479762296	-0.116715659	-0.120846379
0.255055534	0.670847242	0.298470448	0.582175555	0.250742674	0.358683136
-0.432037078	-0.648886573	-0.220366359	-0.432497829	-0.317503056	-0.162969217
0.504691833	0.360535693	0.482441131	0.622594318	0.337622803	0.407184422
-0.510448372	-0.645005569	-0.127195195	-0.473499812	-0.404127581	-0.017311206
0.471456107	0.447059873	0.377015995	0.427354498	0.31724071	0.194337441
-0.33177086	-0.535952083	-0.363600342	-0.470166204	-0.479990222	-0.290815135
0.125862292	0.382378887	0.527152488	0.393434455	0.347062419	0.606634903

-0.092452127	-0.302265581	-0.59205723	-0.510654828	-0.393111438	-0.69184617
0.280121932	0.806912737	0.225396672	0.479540923	0.318010742	0.156406522
-0.493698658	-0.534088125	-0.238968907	-0.445278801	-0.299502472	-0.092119779
0.477854684	0.359927279	0.50066118	0.47916178	0.527830486	0.362846668
-0.101141808	-0.317712235	-0.532669224	-0.447856327	-0.476297252	-0.607698317
-0.448129471	-0.377082148	-0.250696619	-0.443391573	-0.171351942	-0.242431474
-0.251508554	-0.417614713	-0.49153468	-0.537392082	-0.365599581	-0.412923801
0.475037527	0.558356209	0.12252351	0.247239466	0.281766802	0.084446149
0.129975305	0.240439606	0.516842045	0.407211356	0.254278803	0.679301802
-0.371715197	-0.571082234	-0.259793506	-0.399134559	-0.020939596	-0.009550639
0.093555822	-0.306643174	-0.653385192	-0.430301682	-0.318338444	-0.630379416
0.295085271	0.433187933	0.357022596	0.409606704	0.270848756	0.479642926
0.024609506	0.291254595	0.572810271	0.311855732	0.285101509	0.636689339
0.548577854	0.322875449	0.38392321	0.361655757	0.637371562	0.368533248
-0.210480585	-0.563000568	-0.089112808	-0.338317455	-0.084799735	-0.083452649
0.722301227	0.387697707	0.239984574	0.672927895	0.073079994	0.109138818
-0.055632764	0.392540475	0.544559385	0.442815268	0.371447996	0.434021351
0.411879978	0.367696175	0.318999095	1	0.011464755	0.236873194
-0.490030418	-0.551260752	0.026054047	-0.250390777	-0.166315655	0.184007048
-0.083190683	0.463323723	0.36879488	0.239460447	0.513601041	0.285543476
0.242557869	1	0.02616654	0.367696175	0.186457873	-0.019326102
-0.600388935	-0.519453483	-0.121573636	-0.446416572	-0.234762624	0.019527973
0.142328413	0.602019465	0.116258013	0.505581241	0.089630346	0.04030661
0.446165091	0.207558903	0.347369756	0.474849936	0.275780709	0.161483672
0.139301106	0.094782576	0.651987407	0.226315043	0.198799347	0.600823082
-0.639810645	-0.367621795	-0.057692567	-0.35983222	-0.223995537	0.107626496
0.413181517	0.114500517	0.486365271	0.448945817	0.265840199	0.315601463
0.099259144	-0.201225009	-0.557799207	-0.410237989	-0.180178172	-0.615524312
0.081104323	0.02616654	1	0.318999095	0.377022966	0.573809963
0.003745658	0.072168992	0.761729427	0.311897228	0.336626945	0.696195023
0.22387058	0.186457873	0.377022966	0.011464755	1	0.470215844
-0.002818281	-0.019326102	0.573809963	0.236873194	0.470215844	1
1	0.242557869	0.081104323	0.411879978	0.22387058	-0.002818281
0.306391153	0.19544744	0.113714354	0.095875419	0.394506983	-0.023292251
0.387664746	0.20952394	0.215063321	0.522553735	0.144807232	-0.030604241
0.427610155	0.03212308	0.18775615	0.284805943	0.235215162	0.184426521
0.246479359	0.141188921	0.199119658	0.20584511	0.336371722	0.039864413

DUSP1	DAXX	CSF1R	CRK	CDC25B	CD14
-0.258494873	-0.514332875	-0.776199888	-0.425490764	-0.516642349	-0.395072071
-0.309140762	-0.429278502	-0.75743694	-0.393415718	-0.451562295	-0.358606201
-0.240108512	-0.556284094	-0.724090342	-0.417785536	-0.558740675	-0.325962197
-0.203991342	-0.515429966	-0.697969235	-0.310631272	-0.573691252	-0.267038236
-0.194910887	-0.439997295	-0.744706243	-0.32645045	-0.4874065	-0.326044664
-0.257497847	-0.586626966	-0.68009742	-0.235224226	-0.685763056	-0.348191732
-0.292207376	-0.612612648	-0.748457428	-0.507081193	-0.548256649	-0.391658911
0.284251288	0.721782611	0.653571955	0.428163775	0.642912375	0.313170793
-0.22536087	-0.689260935	-0.640099069	-0.36312805	-0.662614811	-0.254331903
-0.199121838	-0.420293922	-0.68093095	-0.245178833	-0.56565655	-0.285243221
0.43014162	0.486233421	0.757846038	0.575069291	0.482640596	0.360762603
0.318416666	0.374385011	0.767023965	0.460851	0.372833004	0.331733809
-0.189812308	-0.60482299	-0.613140288	-0.291444441	-0.658460503	-0.259965438
-0.360731493	-0.5857574	-0.643444166	-0.476538106	-0.572859173	-0.312999849
0.286112586	0.481869804	0.663661236	0.226295551	0.62654572	0.349016552
-0.274281736	-0.508694184	-0.705378968	-0.531536198	-0.417439834	-0.342487455
-0.232507102	-0.561668777	-0.601216613	-0.274302809	-0.660294507	-0.290662571
0.327166751	0.435795931	0.69718438	0.484024811	0.432861317	0.402829931
-0.326993888	-0.532036235	-0.703358057	-0.500514483	-0.457406329	-0.349505764
0.200224434	0.768503781	0.549921049	0.286911937	0.792376174	0.259124038
-0.158459059	-0.422717659	-0.614049622	-0.152268103	-0.558683092	-0.366383679
-0.280893361	-0.48179507	-0.710842293	-0.408570772	-0.450836326	-0.461037901
-0.214767796	-0.515639446	-0.709446699	-0.412800506	-0.521490198	-0.35241148
-0.441667921	-0.505294269	-0.716571926	-0.507140873	-0.442024599	-0.429963008
-0.317764316	-0.566754898	-0.672285372	-0.508232748	-0.488514151	-0.389452971
-0.182794684	-0.482037647	-0.618383861	-0.318955009	-0.539111092	-0.383573369
0.437521843	0.504239464	0.696267568	0.437177117	0.567476569	0.641791789
0.207998167	0.548437767	0.732933603	0.270567235	0.611421616	0.454939051
-0.223343193	-0.475261341	-0.55957313	-0.304811331	-0.606756242	-0.233152292
-0.188356052	-0.454529804	-0.690369084	-0.391025634	-0.456041781	-0.322875825
0.165430101	0.513771995	0.579638875	0.313678451	0.501875875	0.233859527
-0.227368224	-0.464421417	-0.67362375	-0.383809972	-0.391004341	-0.268391268
0.341507148	0.336896661	0.621059976	0.486622067	0.418358054	0.412074343
-0.346780504	-0.519655033	-0.650297286	-0.519159927	-0.416268184	-0.413987678
0.300652889	0.344636281	1	0.391440503	0.346262073	0.545325851
-0.338195908	-0.522003584	-0.523581598	-0.391745577	-0.56182708	-0.342044682
0.142090986	0.49419232	0.510614169	0.282320087	0.51049773	0.155908514

-0.08969154	-0.506228719	-0.479164096	-0.084206215	-0.676856601	-0.172598981
0.246111939	0.582817766	0.573174272	0.446196351	0.491832485	0.276179998
-0.335477028	-0.407410544	-0.603597911	-0.418477177	-0.362824419	-0.339681384
0.401696919	0.494645311	0.483521727	0.393991368	0.55646938	0.336734799
-0.184165677	-0.51443786	-0.488017477	-0.186998558	-0.624661043	-0.320513489
-0.170251417	-0.237104665	-0.640359575	-0.3393358	-0.135929752	-0.325815867
-0.240411585	-0.469990331	-0.492809062	-0.243277238	-0.535984349	-0.310040854
0.207905257	0.425233154	0.641252713	0.512325013	0.324141934	0.274314659
0.13578905	0.306592487	0.554929855	0.118824732	0.478289173	0.205877109
-0.174581806	-0.412222104	-0.629304555	-0.332428586	-0.496415344	-0.340311838
-0.028299621	-0.598091701	-0.409243702	-0.073998757	-0.719617577	-0.166337244
0.104586107	0.289940516	0.487114602	0.135527892	0.366958619	0.012526344
0.094514353	0.457075771	0.48362118	0.154628412	0.489686045	0.114637171
0.289891406	0.320707863	0.525860539	0.350522403	0.269852927	0.308320719
-0.168708244	-0.411086906	-0.577334176	-0.320506627	-0.340377113	-0.289583116
0.30629765	0.093254778	0.579142435	0.276055014	0.209982303	0.478535431
0.203138031	0.709005039	0.346262073	0.150937226	1	0.277033043
0.20584511	0.239460447	0.427354498	0.095875419	0.442815268	0.522553735
-0.282834351	-0.352519774	-0.616763411	-0.549914765	-0.178851229	-0.284537227
0.242823704	1	0.344636281	0.429466467	0.709005039	0.159940973
0.141188921	0.463323723	0.447059873	0.19544744	0.392540475	0.20952394
-0.271933048	-0.175757523	-0.526154739	-0.311082737	-0.240103176	-0.339073028
0.209347857	0.366412333	0.386072262	0.192636949	0.451970595	0.203345568
0.31863118	0.256200429	0.549277116	0.37417027	0.318409606	0.60115247
0.088486604	0.218335821	0.468185447	0.101585811	0.329747412	0.147611321
-0.27120628	-0.24754964	-0.529886931	-0.556842968	-0.064516873	-0.318291146
0.32489886	0.204719575	0.385526605	0.462438642	0.24340573	0.169213517
0.053687345	-0.342893091	-0.316401228	0.190849354	-0.562133923	-0.129813189
0.199119658	0.36879488	0.377015995	0.113714354	0.544559385	0.215063321
0.134704817	0.2791427	0.247131194	-0.078801682	0.512751327	0.058538394
0.336371722	0.513601041	0.31724071	0.394506983	0.371447996	0.144807232
0.039864413	0.285543476	0.194337441	-0.023292251	0.434021351	-0.030604241
0.246479359	-0.083190683	0.471456107	0.306391153	-0.055632764	0.387664746
0.327535859	0.429466467	0.391440503	1	0.150937226	0.129554899
0.266962316	0.159940973	0.545325851	0.129554899	0.277033043	1
0.268852978	0.07314689	0.196047345	0.31267058	0.121949722	0.134696507
1	0.242823704	0.300652889	0.327535859	0.203138031	0.266962316

CACNB4	CACNB2	CACNB1	CACNA2D3	CACNA1I	CACNA1G
0.693394112	0.897937717	0.610803039	0.92027585	-0.372060009	0.638740613
0.641361026	0.860317304	0.617545301	0.876074206	-0.433332762	0.597524033
0.657185835	0.838011216	0.482172179	0.891841254	-0.416566098	0.670299029
0.602836655	0.771100505	0.399465542	0.830592211	-0.567696917	0.589229301
0.660395498	0.79446612	0.441570684	0.851240655	-0.455351608	0.645428713
0.602871293	0.731738362	0.311904985	0.845823173	-0.555296427	0.548198994
0.688655057	0.878524924	0.556862538	1	-0.211461195	0.682260093
-0.571484164	-0.756242674	-0.376321342	-0.849712605	0.477196936	-0.631372525
0.593244799	0.761978877	0.346273259	0.854476305	-0.514478104	0.550584145
0.600940809	0.686722536	0.347180909	0.753817163	-0.601339417	0.494217891
-0.602824851	-0.832420974	-0.621525043	-0.830440534	0.379580997	-0.555880503
-0.628583902	-0.775434603	-0.566368368	-0.79488071	0.407780587	-0.517580033
0.584663136	0.747569249	0.22930008	0.817010354	-0.484090475	0.630028207
0.519623559	0.757505145	0.338082112	0.801469487	-0.431027171	0.629257355
-0.631100945	-0.611028258	-0.247490393	-0.733021943	0.602790176	-0.557740095
0.582989	1	0.665307124	0.878524924	-0.223486129	0.614884921
0.576832726	0.686080489	0.265970091	0.756922644	-0.548065974	0.529589705
-0.531286259	-0.758952285	-0.561851701	-0.763556244	0.306935956	-0.516613485
0.514814779	0.809561875	0.561122221	0.850387109	-0.208743139	0.671199125
-0.621631196	-0.669507263	-0.251009804	-0.770681556	0.476318741	-0.552310699
0.486930837	0.669564088	0.290641668	0.686202055	-0.5866199	0.462318513
0.644233758	0.836798216	0.62943631	0.8852259	-0.148674256	0.614878842
0.662036407	0.747599465	0.585414865	0.780407359	-0.329406226	0.602754976
0.617455296	0.813379341	0.539908638	0.838593748	-0.200163864	0.634303688
0.573235476	0.755388804	0.507467601	0.860747139	-0.21081325	0.653332157
0.588615118	0.677207892	0.472529482	0.717244329	-0.457871723	0.484418934
-0.590883015	-0.650171935	-0.510236741	-0.763648959	0.261551963	-0.452079348
-0.555369322	-0.63213766	-0.250715164	-0.690150395	0.521733773	-0.563348165
0.561180024	0.573228948	0.308509666	0.68116253	-0.605276935	0.315720691
0.825798164	0.728907508	0.52943205	0.832004552	-0.141037977	0.678302018
-0.605026066	-0.687755346	-0.398056145	-0.729307045	0.329449888	-0.625418649
0.608918884	0.807726689	0.554889671	0.840094484	-0.144768214	0.715206156
-0.484885331	-0.715918612	-0.586306107	-0.669079061	0.445482673	-0.340216826
0.625808852	0.844526202	0.727815695	0.851853235	-0.046710285	0.570132481
-0.629304555	-0.705378968	-0.529886931	-0.748457428	0.247131194	-0.577334176
0.531845492	0.64228773	0.388448503	0.704394654	-0.38331265	0.548197398
-0.491785686	-0.602132534	-0.19372792	-0.662923014	0.602932091	-0.511782002

0.432722976	0.517397557	0.067847001	0.634478961	-0.668617502	0.378687913
-0.604499292	-0.771348413	-0.51457968	-0.732234043	0.232324875	-0.575720763
0.601169306	0.708161131	0.584563454	0.722966244	-0.210697487	0.587105358
-0.480289392	-0.578909309	-0.502072621	-0.641488944	0.430372802	-0.242780595
0.372406162	0.531114695	0.051981034	0.624737649	-0.525885176	0.402965642
0.494624671	0.718454698	0.562106018	0.697076868	-0.223035554	0.558684612
0.357320426	0.648676568	0.293331341	0.56274154	-0.484751508	0.311248912
-0.738475719	-0.692029844	-0.595080599	-0.761580777	0.06608007	-0.574995769
-0.449118355	-0.45965372	-0.118014626	-0.544734019	0.665137676	-0.446602649
1	0.582989	0.420531375	0.688655057	-0.183345859	0.593379229
0.402767111	0.43273762	-0.009735792	0.532279545	-0.664935768	0.37590156
-0.41086039	-0.525796024	-0.130904101	-0.581956851	0.474537254	-0.429852278
-0.396026103	-0.498655452	-0.104811596	-0.546052444	0.61621789	-0.451025868
-0.208030617	-0.592946496	-0.443569468	-0.570035226	0.39677328	-0.292118949
0.593379229	0.614884921	0.390913181	0.682260093	-0.007048046	1
-0.539711916	-0.565378394	-0.629075941	-0.528912236	0.191565881	-0.33730996
-0.496415344	-0.417439834	-0.064516873	-0.548256649	0.512751327	-0.340377113
-0.399134559	-0.478058256	-0.35983222	-0.472679558	0.311897228	-0.338317455
0.635356902	0.69298979	0.673008232	0.723366769	0.229558364	0.568946099
-0.412222104	-0.508694184	-0.24754964	-0.612612648	0.2791427	-0.411086906
-0.571082234	-0.647252494	-0.367621795	-0.640642373	0.072168992	-0.563000568
0.490495793	0.605471739	0.488280868	0.544224916	-0.143020668	0.359679761
-0.447883983	-0.565088206	-0.307517759	-0.570786922	0.100339101	-0.46925698
-0.386268901	-0.474039678	-0.515101771	-0.494008161	0.150502315	-0.263095812
-0.370555674	-0.249196971	0.025238772	-0.384309934	0.631692283	-0.323142156
0.420531375	0.665307124	1	0.556862538	0.064946348	0.390913181
-0.242555619	-0.489892948	-0.51057175	-0.359960835	0.410824366	-0.091140033
0.262666244	0.213108822	-0.211657111	0.333970781	-0.647070331	0.300696421
-0.259793506	-0.249389114	-0.057692567	-0.291750517	0.761729427	-0.089112808
-0.183345859	-0.223486129	0.064946348	-0.211461195	1	-0.007048046
-0.020939596	-0.426821561	-0.223995537	-0.479426673	0.336626945	-0.084799735
-0.009550639	-0.189070116	0.107626496	-0.270529361	0.696195023	-0.083452649
-0.371715197	-0.44168599	-0.639810645	-0.431374166	0.003745658	-0.210480585
-0.332428586	-0.531536198	-0.556842968	-0.507081193	-0.078801682	-0.320506627
-0.340311838	-0.342487455	-0.318291146	-0.391658911	0.058538394	-0.289583116
-0.186225248	-0.301079593	-0.457074737	-0.276548287	0.173837439	0.07417993
-0.174581806	-0.274281736	-0.27120628	-0.292207376	0.134704817	-0.168708244

BRAF	BDNF	ANGPT2	AKT1
0.866750025	0.818332212	-0.524953264	-0.788245725
0.857048067	0.802681367	-0.544529911	-0.802816398
0.881169653	0.782022591	-0.430878761	-0.783871547
0.906727111	0.750344688	-0.402181813	-0.848265842
0.875178923	0.772266356	-0.406513513	-0.82273276
1	0.757727773	-0.430187838	-0.858127527
0.845823173	0.780407359	-0.494008161	-0.733021943
-0.880128269	-0.73094696	0.401478247	0.762699197
0.90544797	0.731967876	-0.356319111	-0.80770624
0.884040511	0.72040973	-0.40654851	-0.858277378
-0.763736432	-0.761597111	0.576522961	0.747658445
-0.7695523	-0.757128838	0.486889359	0.725604991
0.884025167	0.683472164	-0.285959854	-0.802801034
0.803893504	0.686941199	-0.360218684	-0.764159329
-0.858127527	-0.681097854	0.412960722	1
0.731738362	0.747599465	-0.474039678	-0.611028258
0.862907717	0.699587952	-0.334830861	-0.839873886
-0.710108621	-0.69477614	0.613725256	0.64105004
0.758157455	0.732793794	-0.483495224	-0.633579376
-0.852948302	-0.683639623	0.335276624	0.764667207
0.813365664	0.616336168	-0.439653012	-0.756107595
0.766257458	0.739799585	-0.517644545	-0.655821843
0.757727773	1	-0.451552311	-0.681097854
0.738144451	0.742307787	-0.445602904	-0.703363972
0.758747708	0.753402874	-0.419791433	-0.686438422
0.758507552	0.702682071	-0.499560566	-0.766517191
-0.750400812	-0.690765114	0.616523499	0.672857974
-0.779649057	-0.64374242	0.489994857	0.759445861
0.820602184	0.613458895	-0.451091724	-0.783023741
0.710853111	0.722037163	-0.483161688	-0.666633605
-0.724306948	-0.65875316	0.402463534	0.696310575
0.723329583	0.783613346	-0.319270007	-0.595183778
-0.640092676	-0.602543024	0.6315131	0.620857693
0.664997651	0.7202537	-0.544093109	-0.534816443
-0.68009742	-0.709446699	0.549277116	0.663661236
0.737253417	0.64790607	-0.389750993	-0.738843452
-0.766270968	-0.582451338	0.281689978	0.70474365

0.864043576	0.552880191	-0.243826831	-0.817884361
-0.614139993	-0.660514318	0.404616775	0.583226664
0.648459696	0.64828244	-0.455474453	-0.625570675
-0.689822972	-0.600744353	0.516797788	0.561930067
0.752607657	0.470374315	-0.352214255	-0.728592013
0.584475579	0.590816654	-0.470965457	-0.534108629
0.646452581	0.538843406	-0.362677603	-0.493148842
-0.606096164	-0.670095256	0.407808201	0.570498577
-0.714103137	-0.480284539	0.336452727	0.814270244
0.602871293	0.662036407	-0.386268901	-0.631100945
0.744920722	0.497762434	-0.19818127	-0.71044664
-0.676155601	-0.533303112	0.07264513	0.684363462
-0.687649911	-0.519042378	0.186794931	0.640747461
-0.580283864	-0.552147213	0.336375502	0.470221553
0.548198994	0.602754976	-0.263095812	-0.557740095
-0.473672896	-0.556595119	0.544157388	0.47179211
-0.685763056	-0.521490198	0.318409606	0.62654572
-0.529577112	-0.466143562	0.474849936	0.541718501
0.429042521	0.568126754	-0.473315761	-0.317717051
-0.586626966	-0.515639446	0.256200429	0.481869804
-0.517498248	-0.569903414	0.207558903	0.442910321
0.470610347	0.545600364	-0.37750087	-0.472789939
-0.52038992	-0.470449968	0.251759012	0.434801636
-0.430187838	-0.451552311	1	0.412960722
-0.563487242	-0.436117409	0.202919216	0.636612894
0.311904985	0.585414865	-0.515101771	-0.247490393
-0.381486364	-0.420377542	0.487776759	0.376527303
0.607070234	0.336206236	-0.072595234	-0.649715039
-0.528698803	-0.45367842	0.347369756	0.558093056
-0.555296427	-0.329406226	0.150502315	0.602790176
-0.528057412	-0.377713796	0.275780709	0.409218307
-0.563793227	-0.223883068	0.161483672	0.519172823
-0.321607039	-0.446372218	0.446165091	0.285120275
-0.235224226	-0.412800506	0.37417027	0.226295551
-0.348191732	-0.35241148	0.60115247	0.349016552
-0.255749355	-0.209217594	0.411369994	0.162409576
-0.257497847	-0.214767796	0.31863118	0.286112586

Ras_genes

PCC	Gene.symb	VEGFB	TBK1	SHC1	RGL2
43.7401675	PAK1	-0.697228619	0.733621855	-0.655889331	-0.642159516
43.63625454	PRKCB	-0.689738457	0.829053022	-0.723529489	-0.652555718
43.19225555	PAK5	-0.7310636	0.807851631	-0.707192304	-0.651508918
43.01893124	MAP2K1	-0.612322566	0.82495208	-0.685851928	-0.554559896
42.50999552	GNG2	-0.558148283	0.752269608	-0.733383624	-0.588469488
42.03464092	MAPK9	-0.56368769	0.761361657	-0.693410406	-0.502877904
42.00568316	PDGFRB	0.688908431	-0.617628303	0.643933098	0.783811599
41.98901661	CALM1	-0.650732884	0.752665196	-0.685454714	-0.614422468
41.67154361	FOXO4	0.680784551	-0.678321967	0.597487016	0.618462058
41.32101479	RASA1	-0.60883811	0.869515322	-0.730006839	-0.578282198
40.92820454	CALM3	-0.703368162	0.705114538	-0.611012722	-0.698315162
39.91801657	PIK3CB	-0.481597017	0.753955966	-0.700280991	-0.432062155
39.36546159	CALM2	-0.510036545	0.816307374	-0.682415196	-0.553426143
39.31523282	KITLG	-0.554415335	0.738820931	-0.676272584	-0.517996811
39.30155275	AKT1	0.570498577	-0.724857573	0.637951947	0.424277852
38.64006785	MAPK10	-0.407247249	0.682772931	-0.661823528	-0.418294577
38.22900821	BDNF	-0.670095256	0.684435804	-0.586016449	-0.647156951
38.17131273	PDGFD	-0.59154751	0.610558839	-0.636373532	-0.56502146
38.10149492	PRKACB	-0.531407256	0.639364433	-0.676342392	-0.50983234
37.96269388	MAPK8	-0.777053467	0.704708587	-0.626062284	-0.633111847
37.43002525	RASGRP1	-0.676077715	0.768706215	-0.622313654	-0.62842356
37.25166418	NFKB1	0.473123086	-0.476046382	0.572170813	0.582961862
36.53212731	GNB5	-0.674416688	0.636295873	-0.56429191	-0.45819668
36.20225132	TBK1	-0.56191422	1	-0.64776259	-0.428067042
36.09009473	RASSF1	0.520185831	-0.593771714	0.693009393	0.669724151
36.0112191	GRIN2A	-0.677448338	0.649128716	-0.627213355	-0.669339062
35.9626535	CSF1R	0.641252713	-0.594491304	0.530701967	0.585705437
35.96186803	MAPK1	-0.714212087	0.620500623	-0.630588223	-0.77473426
35.57705954	PAK4	0.602706421	-0.756345186	0.629545412	0.543379597
35.51664348	IGF1	-0.554759631	0.63722109	-0.625327329	-0.543394356
35.46888495	SHC1	0.452304815	-0.64776259	1	0.521992388
34.29937201	RALGDS	0.343110618	-0.547747258	0.669512718	0.38607044
34.23079018	KIT	-0.666625572	0.557069566	-0.560393351	-0.603676852
33.97070865	GAB2	0.568225588	-0.624581669	0.531648002	0.484388422
33.60277719	PLCG1	0.610031406	-0.397644395	0.41229509	0.784397856
33.24926558	KRAS	-0.328894392	0.513961123	-0.610364144	-0.574975322
33.2215488	RAPGEF5	-0.544982999	0.782697411	-0.633641066	-0.380728826

33.11463415	RGL2	0.709905429	-0.428067042	0.521992388	1
32.820862	GNG7	0.591600826	-0.622229911	0.562125902	0.559738237
32.62603582	VEGFB	1	-0.56191422	0.452304815	0.709905429
32.06277493	ETS1	0.578212049	-0.418777299	0.465581146	0.636791931
31.55903823	FGFR3	0.408242805	-0.60258196	0.417621794	0.332840543
31.32602138	PLCE1	0.477909735	-0.346134217	0.554741844	0.632156998
31.15215783	RALB	-0.193699058	0.625849452	-0.594081793	-0.212922427
29.310903	PIK3CD	0.313049852	-0.524756003	0.442400904	0.4094257
29.15984411	RAB5A	-0.151676188	0.6331311	-0.561727226	-0.202018467
28.99844049	GNG10	-0.19787369	0.552644783	-0.495657583	-0.291832579
28.97771569	EFNA1	0.247239466	-0.388918495	0.628153907	0.260227297
28.49900479	RALA	0.458219639	-0.321032608	0.311860191	0.4759942
28.28281117	KSR2	0.274582079	-0.656858135	0.489381914	0.239732917
27.99702704	PLA1A	0.469910769	-0.356029999	0.58914724	0.53567761
27.54564222	GNG3	-0.534248177	0.406686695	-0.465762359	-0.656797992
27.37518292	MET	-0.447459861	0.308310881	-0.441314271	-0.507945022
27.0075728	FGF9	-0.723282185	0.404969571	-0.383651794	-0.671814079
26.84753826	CALML4	0.388143459	-0.357764714	0.391362073	0.61124131
26.71974862	FGF1	0.558356209	-0.501442748	0.395349599	0.52110981
26.65913148	RAF1	0.249246938	-0.122872197	0.387716191	0.546051005
26.39576544	MAPK3	0.319212803	-0.479997022	0.369504117	0.199768119
25.68517767	ANGPT2	0.407808201	-0.290395627	0.503951171	0.473744004
24.71701564	ELK1	0.12252351	-0.401797022	0.40824564	0.252675321
22.806073	PLCG2	0.439603673	-0.177692812	0.343093358	0.546842569
22.03412867	FGF2	0.475037527	-0.219288071	0.380686334	0.42061941

RASSF1	RASGRP1	RASA1	RAPGEF5	RALGDS	RALB
-0.653566957	0.774124929	0.828046666	0.676700632	-0.651077316	0.588405492
-0.739242023	0.799101651	0.897049191	0.766012083	-0.670629057	0.680808995
-0.723636027	0.832968109	0.88484408	0.771363802	-0.624258208	0.626237117
-0.700558107	0.749388607	0.910376528	0.757984439	-0.692414275	0.76336469
-0.721428548	0.692524326	0.88687596	0.698102839	-0.692984262	0.772217812
-0.669947589	0.651027697	0.855575279	0.732305379	-0.692922767	0.820380841
0.77318732	-0.748906147	-0.747662089	-0.599541125	0.604572014	-0.488579821
-0.660596132	0.730802535	0.828687107	0.687469945	-0.629796522	0.647454551
0.665176894	-0.733530967	-0.796487373	-0.579221739	0.712411309	-0.60614302
-0.722859951	0.773831605	1	0.754121411	-0.671742585	0.716215509
-0.692514997	0.751465099	0.779185158	0.652617564	-0.52527258	0.493859299
-0.631968906	0.610870345	0.844657841	0.720527072	-0.684497373	0.857060347
-0.721022627	0.698748439	0.877374789	0.713725767	-0.603519254	0.743898756
-0.621927516	0.708400679	0.848514225	0.671993886	-0.711004063	0.67958737
0.592210448	-0.686438422	-0.80770624	-0.702526732	0.709489477	-0.70883458
-0.65034083	0.588118119	0.782217466	0.619684693	-0.71998089	0.818770534
-0.597485591	0.753402874	0.731967876	0.611927998	-0.562117544	0.462393816
-0.560584035	0.615185112	0.723755199	0.540886632	-0.628859902	0.543402894
-0.605919706	0.576147957	0.79006557	0.636212628	-0.582538479	0.715422706
-0.582699562	0.791015328	0.729436637	0.590998944	-0.524441105	0.362439194
-0.628898621	1	0.773831605	0.670373666	-0.553979901	0.383791384
0.642283915	-0.579984083	-0.635967566	-0.401509171	0.675097669	-0.497913403
-0.470601522	0.572617853	0.689107801	0.566895561	-0.618469269	0.575589362
-0.593771714	0.768706215	0.869515322	0.782697411	-0.547747258	0.625849452
1	-0.628898621	-0.722859951	-0.555177222	0.656412693	-0.562548046
-0.627556333	0.834414156	0.706418456	0.543981038	-0.581362029	0.308414353
0.598973372	-0.672285372	-0.640099069	-0.617358111	0.478461242	-0.388501427
-0.586220402	0.756808462	0.665266851	0.496166373	-0.444635354	0.244788112
0.618469777	-0.734004931	-0.809032862	-0.603540374	0.568766755	-0.552232432
-0.555016869	0.722824674	0.684512891	0.503698377	-0.623829095	0.475496835
0.693009393	-0.622313654	-0.730006839	-0.633641066	0.669512718	-0.594081793
0.656412693	-0.553979901	-0.671742585	-0.471346606	1	-0.721600602
-0.483385269	0.656112776	0.606483492	0.438969954	-0.433721229	0.302750575
0.608591131	-0.558832305	-0.697136356	-0.456814422	0.603398939	-0.543296364
0.601593301	-0.552914129	-0.586455873	-0.361031719	0.431259085	-0.35484647
-0.683946085	0.495825727	0.636406226	0.412892331	-0.627364073	0.607592924
-0.555177222	0.670373666	0.754121411	1	-0.471346606	0.593026778

0.669724151	-0.62842356	-0.578282198	-0.380728826	0.38607044	-0.212922427
0.548286005	-0.691438061	-0.688572495	-0.536960853	0.519094566	-0.421785976
0.520185831	-0.676077715	-0.60883811	-0.544982999	0.343110618	-0.193699058
0.561824066	-0.608908536	-0.522774494	-0.495866767	0.436252652	-0.231348131
0.48375836	-0.52982231	-0.670335614	-0.678712572	0.552568615	-0.654930282
0.606963653	-0.49041654	-0.425701033	-0.408908342	0.512900968	-0.275648902
-0.562548046	0.383791384	0.716215509	0.593026778	-0.721600602	1
0.609950018	-0.423903871	-0.651714887	-0.518101408	0.509861982	-0.616259619
-0.559397005	0.384316894	0.725332513	0.557269777	-0.605189023	0.884194831
-0.555253839	0.325991422	0.684781792	0.479022335	-0.568918422	0.847566029
0.50368632	-0.376021495	-0.478096766	-0.298468871	0.673920918	-0.560597213
0.444200181	-0.4022223	-0.474659344	-0.285118093	0.491668824	-0.360543129
0.548730029	-0.465206595	-0.736552294	-0.620702367	0.540077251	-0.704451524
0.524074382	-0.5366008	-0.434706557	-0.322052965	0.433589	-0.209854466
-0.433003676	0.615705809	0.429904146	0.23138453	-0.314043077	0.060867311
-0.381182149	0.382033391	0.384130562	0.320300439	-0.431015613	0.245902392
-0.408788988	0.564270711	0.429070775	0.320118786	-0.207572151	-0.014942779
0.439653911	-0.535641401	-0.448815217	-0.374891982	0.358919208	-0.24591115
0.399931318	-0.549982354	-0.540994005	-0.35099882	0.438637426	-0.238635503
0.492073223	-0.305999367	-0.366000142	-0.122303456	0.454494047	-0.31614599
0.42136265	-0.386372394	-0.528875575	-0.627143405	0.478946124	-0.598473958
0.436515999	-0.419791433	-0.356319111	-0.214568879	0.403741302	-0.184577898
0.42925533	-0.335093682	-0.518323143	-0.371998182	0.537892341	-0.605514452
0.426094546	-0.410896388	-0.251743291	-0.253238529	0.23697941	-0.002615818
0.232869667	-0.354201356	-0.240824786	-0.314660133	0.262414532	-0.036971339

RALA	RAF1	RAB5A	PRKCB	PRKACB	PLCG2
-0.610924337	-0.573600699	0.499422914	0.912433154	0.760968746	-0.501454668
-0.597947595	-0.442341262	0.625266	1	0.742066617	-0.412561597
-0.52994016	-0.407024219	0.570422599	0.945543058	0.771610841	-0.431846236
-0.597678043	-0.45740358	0.721666078	0.943185559	0.801373186	-0.314453308
-0.613640582	-0.574566248	0.760292111	0.905618812	0.856578834	-0.289627818
-0.565640761	-0.509499074	0.741595908	0.886592296	0.849228528	-0.306556644
0.581154005	0.647218952	-0.427106819	-0.836595925	-0.719580826	0.580143214
-0.547945074	-0.482677438	0.565203848	0.918176016	0.732463056	-0.406014919
0.669228657	0.548582276	-0.602079014	-0.824224998	-0.782891805	0.351219946
-0.474659344	-0.366000142	0.725332513	0.897049191	0.79006557	-0.251743291
-0.617463382	-0.525730067	0.452796737	0.922507693	0.646740199	-0.537531562
-0.504984017	-0.453905739	0.795615191	0.836931818	0.867496	-0.210995231
-0.472545103	-0.395472818	0.767272153	0.887315567	0.752199295	-0.25310218
-0.510073566	-0.371733371	0.641119657	0.812641854	0.741054491	-0.272014026
0.470704722	0.376527303	-0.648023694	-0.783871547	-0.783023741	0.242829285
-0.468109469	-0.482758782	0.721659661	0.838608054	0.7345614	-0.340599481
-0.441369933	-0.420377542	0.405659569	0.782022591	0.613458895	-0.422237692
-0.488842264	-0.459816724	0.458937761	0.735799243	0.716018949	-0.344787793
-0.501626354	-0.531425787	0.726464044	0.742066617	1	-0.268129586
-0.420085147	-0.343282858	0.293116011	0.795745929	0.602126368	-0.537673033
-0.4022223	-0.305999367	0.384316894	0.799101651	0.576147957	-0.410896388
0.651875056	0.765239677	-0.432433356	-0.720291591	-0.66195976	0.507953374
-0.481812472	-0.331264017	0.450312516	0.733356234	0.675225966	-0.360244145
-0.321032608	-0.122872197	0.6331311	0.829053022	0.639364433	-0.177692812
0.444200181	0.492073223	-0.559397005	-0.739242023	-0.605919706	0.426094546
-0.452427616	-0.348947116	0.2658506	0.72894981	0.514275414	-0.465773728
0.433625282	0.385526605	-0.314594996	-0.724090342	-0.55957313	0.706561068
-0.368926827	-0.403722825	0.17060265	0.742306262	0.50877803	-0.574957402
0.336724023	0.260386719	-0.585265517	-0.730582644	-0.726181477	0.152675525
-0.414028092	-0.366943766	0.419474703	0.684401376	0.61918547	-0.244139706
0.311860191	0.387716191	-0.561727226	-0.723529489	-0.676342392	0.343093358
0.491668824	0.454494047	-0.605189023	-0.670629057	-0.582538479	0.23697941
-0.440615616	-0.376842267	0.237628891	0.692802027	0.560331543	-0.409223253
0.479627946	0.289678858	-0.475842474	-0.735299932	-0.554257805	0.301436764
0.641774404	0.727207696	-0.36733514	-0.642595179	-0.634296108	0.386834657
-0.426061597	-0.575310897	0.569297709	0.679045504	0.54446556	-0.39119624
-0.285118093	-0.122303456	0.557269777	0.766012083	0.636212628	-0.253238529

0.4759942	0.546051005	-0.202018467	-0.652555718	-0.50983234	0.546842569
0.377836773	0.302313408	-0.439868641	-0.590983987	-0.671185124	0.191378815
0.458219639	0.249246938	-0.151676188	-0.689738457	-0.531407256	0.439603673
0.450498514	0.491932811	-0.143221903	-0.647829332	-0.48048181	0.573502435
0.504240008	0.293460283	-0.554216909	-0.718034286	-0.562480362	0.174003463
0.496838041	0.629277646	-0.194159632	-0.607997532	-0.451565257	0.619413058
-0.360543129	-0.31614599	0.884194831	0.680808995	0.715422706	-0.002615818
0.485588976	0.40580499	-0.718953018	-0.616641579	-0.661632863	0.171575991
-0.390627363	-0.330115458	1	0.625266	0.726464044	0.08766535
-0.47793223	-0.439813711	0.901425332	0.628643654	0.735857952	0.066967286
0.360442299	0.448945817	-0.420067187	-0.579396077	-0.477299232	0.340932508
1	0.606405872	-0.390627363	-0.597947595	-0.501626354	0.279604766
0.402747718	0.208205285	-0.789131026	-0.655655775	-0.590466448	-0.012355796
0.356051437	0.454039439	-0.185248946	-0.522005415	-0.460077517	0.585166686
-0.216076664	-0.381506069	-0.051591708	0.535142364	0.293911025	-0.579744765
-0.311489253	-0.463950052	0.041441182	0.462161965	0.424459901	-0.520059441
-0.369960288	-0.310968249	-0.090518829	0.549061655	0.337081729	-0.611616467
0.293862256	0.463327209	-0.169246863	-0.532770653	-0.346007054	0.46541799
0.331884264	0.114500517	-0.11258664	-0.577907549	-0.261760672	0.307797596
0.606405872	1	-0.330115458	-0.442341262	-0.531425787	0.408863968
0.451588965	0.32045941	-0.615841477	-0.538319706	-0.616811318	0.079884229
0.327831788	0.487776759	-0.160013937	-0.430878761	-0.451091724	0.544579199
0.348545469	0.486365271	-0.657169798	-0.425646353	-0.621373258	0.079264287
0.279604766	0.408863968	0.08766535	-0.412561597	-0.268129586	1
0.296031231	0.413181517	0.130121564	-0.367740128	-0.335080393	0.492704428

PLCG1	PLCE1	PLA1A	PIK3CD	PIK3CB	PDGFRB
-0.701843262	-0.664005516	-0.572389007	-0.505633378	0.812968922	-0.886177268
-0.642595179	-0.607997532	-0.522005415	-0.616641579	0.836931818	-0.836595925
-0.638421829	-0.575154203	-0.5041312	-0.597276749	0.842395151	-0.835883458
-0.653031565	-0.536297668	-0.466345712	-0.656441368	0.907665108	-0.816833531
-0.689141422	-0.553817829	-0.461271804	-0.658655438	0.925049281	-0.820315943
-0.631459721	-0.547423068	-0.444705369	-0.636046803	0.954636302	-0.779767601
0.773362414	0.726089239	0.604296756	0.515200182	-0.715959222	1
-0.646397629	-0.569922828	-0.507142769	-0.557489017	0.833916908	-0.818948558
0.688498811	0.539766984	0.50073351	0.628601649	-0.766037618	0.806352345
-0.586455873	-0.425701033	-0.434706557	-0.651714887	0.844657841	-0.747662089
-0.686311367	-0.682729518	-0.543894337	-0.548903425	0.70804254	-0.833267668
-0.568285186	-0.446685918	-0.376307854	-0.633554942	1	-0.715959222
-0.587264134	-0.457715414	-0.399596815	-0.72054177	0.82799205	-0.711597227
-0.526191531	-0.411724468	-0.409795885	-0.58474619	0.811693066	-0.723604666
0.510408197	0.433717153	0.402403387	0.603714866	-0.85420046	0.747658445
-0.518685359	-0.529396005	-0.438653094	-0.629354601	0.85930795	-0.697663702
-0.604733252	-0.590751666	-0.495866254	-0.461025135	0.650855403	-0.761597111
-0.59931364	-0.490322249	-0.447423171	-0.515859209	0.738978435	-0.738719451
-0.634296108	-0.451565257	-0.460077517	-0.661632863	0.867496	-0.719580826
-0.544720802	-0.57134542	-0.574048649	-0.307458168	0.600845606	-0.757561789
-0.552914129	-0.49041654	-0.5366008	-0.423903871	0.610870345	-0.748906147
0.694065971	0.726827528	0.637161678	0.518356338	-0.638947072	0.784478661
-0.496182971	-0.440621385	-0.436649309	-0.464373069	0.729756524	-0.654867031
-0.397644395	-0.346134217	-0.356029999	-0.524756003	0.753955966	-0.617628303
0.601593301	0.606963653	0.524074382	0.609950018	-0.631968906	0.77318732
-0.538725043	-0.516101003	-0.570683874	-0.314871653	0.531007	-0.750251382
0.550073433	0.605913762	0.534058982	0.50147791	-0.588992735	0.757846038
-0.589583123	-0.597796825	-0.611048023	-0.248477641	0.506711645	-0.774504894
0.504615135	0.30062654	0.403439149	0.559830777	-0.675195647	0.672694002
-0.515321535	-0.417748972	-0.506717201	-0.404013444	0.635314778	-0.676277585
0.41229509	0.554741844	0.58914724	0.442400904	-0.700280991	0.643933098
0.431259085	0.512900968	0.433589	0.509861982	-0.684497373	0.604572014
-0.549316811	-0.477982364	-0.48602798	-0.319878431	0.552840375	-0.72482854
0.417370129	0.412151521	0.37432692	0.398356056	-0.636730721	0.587522052
1	0.536691651	0.439745511	0.51027444	-0.568285186	0.773362414
-0.546881527	-0.601767525	-0.438817572	-0.498997513	0.64261335	-0.665623174
-0.361031719	-0.408908342	-0.322052965	-0.518101408	0.720527072	-0.599541125

0.784397856	0.632156998	0.53567761	0.4094257	-0.432062155	0.783811599
0.523887075	0.314510082	0.403308874	0.382458176	-0.60579246	0.675587452
0.610031406	0.477909735	0.469910769	0.313049852	-0.481597017	0.688908431
0.570717556	0.685250299	0.563728447	0.361358077	-0.47556737	0.755269481
0.450494729	0.400477483	0.192613313	0.460931642	-0.747843512	0.628309413
0.536691651	1	0.606586765	0.315962152	-0.446685918	0.726089239
-0.35484647	-0.275648902	-0.209854466	-0.616259619	0.857060347	-0.488579821
0.51027444	0.315962152	0.245867716	1	-0.633554942	0.515200182
-0.36733514	-0.194159632	-0.185248946	-0.718953018	0.795615191	-0.427106819
-0.514722238	-0.224999564	-0.173293772	-0.677292716	0.799729966	-0.479075796
0.300802567	0.544844763	0.452558409	0.274558708	-0.604048813	0.505093266
0.641774404	0.496838041	0.356051437	0.485588976	-0.504984017	0.581154005
0.381783249	0.177083728	0.123113548	0.729781718	-0.683485101	0.442086631
0.439745511	0.606586765	1	0.245867716	-0.376307854	0.604296756
-0.425117139	-0.557300175	-0.556457917	0.002392148	0.274578251	-0.659603266
-0.457635729	-0.531375896	-0.466559264	-0.084334866	0.452118076	-0.606753502
-0.493115266	-0.500653571	-0.530394208	-0.131161364	0.263164392	-0.585704974
0.539900204	0.574147266	0.418398901	0.238133517	-0.41112986	0.607558396
0.317500986	0.376989349	0.30063812	0.15074782	-0.365112073	0.481001345
0.727207696	0.629277646	0.454039439	0.40580499	-0.453905739	0.647218952
0.412650096	0.30873168	0.142647484	0.674961042	-0.653374843	0.441166356
0.393657101	0.564424312	0.594349275	0.344366936	-0.337368426	0.576522961
0.43278634	0.327583406	0.234402209	0.651277775	-0.564489834	0.436935246
0.386834657	0.619413058	0.585166686	0.171575991	-0.210995231	0.580143214
0.438577008	0.623273272	0.430193822	-0.006284802	-0.319319822	0.516735454

PDGFD	PAK5	PAK4	PAK1	NFKB1	MET
0.777199535	0.901346791	-0.67864019	1	-0.810787632	0.59971707
0.735799243	0.945543058	-0.730582644	0.912433154	-0.720291591	0.462161965
0.759065557	1	-0.742555817	0.901346791	-0.680002941	0.493077368
0.748148812	0.912384176	-0.710247185	0.908625801	-0.714174104	0.417558221
0.764555682	0.890852249	-0.702866509	0.880766512	-0.734458017	0.45093873
0.733287652	0.873355212	-0.668545956	0.877711058	-0.711727794	0.486374325
-0.738719451	-0.835883458	0.672694002	-0.886177268	0.784478661	-0.606753502
0.773513948	0.889708216	-0.670122207	0.905379134	-0.721419266	0.480670189
-0.788293061	-0.834018418	0.76265632	-0.866757548	0.744330661	-0.484358194
0.723755199	0.88484408	-0.809032862	0.828046666	-0.635967566	0.384130562
0.664056509	0.880245865	-0.593168285	0.89070214	-0.75139852	0.466199973
0.738978435	0.842395151	-0.675195647	0.812968922	-0.638947072	0.452118076
0.644190371	0.838266297	-0.726668477	0.770225601	-0.624500976	0.287825004
0.776199434	0.822625856	-0.776897105	0.758829917	-0.593259506	0.545288347
-0.766517191	-0.822345798	0.755050802	-0.802816398	0.620857693	-0.472789939
0.651246008	0.78582346	-0.566950729	0.808566233	-0.720690936	0.380521762
0.702682071	0.776960336	-0.662294047	0.802681367	-0.602543024	0.545600364
1	0.759065557	-0.67787149	0.777199535	-0.649708749	0.58539749
0.716018949	0.771610841	-0.726181477	0.760968746	-0.66195976	0.424459901
0.673307032	0.806695156	-0.686018773	0.841849726	-0.632448814	0.528693939
0.615185112	0.832968109	-0.734004931	0.774124929	-0.579984083	0.382033391
-0.649708749	-0.680002941	0.495744884	-0.810787632	1	-0.545456408
0.722343783	0.744021576	-0.655907099	0.788855834	-0.607717637	0.554651948
0.610558839	0.807851631	-0.756345186	0.733621855	-0.476046382	0.308310881
-0.560584035	-0.723636027	0.618469777	-0.653566957	0.642283915	-0.381182149
0.63859806	0.752794338	-0.719472731	0.723509764	-0.582319276	0.606263644
-0.618383861	-0.744276739	0.545297611	-0.75743694	0.621059976	-0.526154739
0.680410942	0.749135487	-0.647338399	0.787054702	-0.623804392	0.592026739
-0.67787149	-0.742555817	1	-0.67864019	0.495744884	-0.34648649
0.759708493	0.715086997	-0.734549872	0.688718663	-0.558289584	0.565875168
-0.636373532	-0.707192304	0.629545412	-0.655889331	0.572170813	-0.441314271
-0.628859902	-0.624258208	0.568766755	-0.651077316	0.675097669	-0.431015613
0.731464282	0.71246464	-0.649836781	0.731200789	-0.532601566	0.560055186
-0.649300621	-0.732408749	0.586896009	-0.684245106	0.504757868	-0.501984943
-0.59931364	-0.638421829	0.504615135	-0.701843262	0.694065971	-0.457635729
0.524639313	0.61503239	-0.40451472	0.645630136	-0.636744938	0.353846984
0.540886632	0.771363802	-0.603540374	0.676700632	-0.401509171	0.320300439

-0.56502146	-0.651508918	0.543379597	-0.642159516	0.582961862	-0.507945022
-0.674403229	-0.65032837	0.807773026	-0.634444121	0.429014343	-0.456102552
-0.59154751	-0.7310636	0.602706421	-0.697228619	0.473123086	-0.447459861
-0.547403854	-0.630813482	0.431847479	-0.730342125	0.708502531	-0.507926153
-0.516275669	-0.69119411	0.415993768	-0.682070089	0.445263635	-0.363524781
-0.490322249	-0.575154203	0.30062654	-0.664005516	0.726827528	-0.531375896
0.543402894	0.626237117	-0.552232432	0.588405492	-0.497913403	0.245902392
-0.515859209	-0.597276749	0.559830777	-0.505633378	0.518356338	-0.084334866
0.458937761	0.570422599	-0.585265517	0.499422914	-0.432433356	0.041441182
0.516090796	0.566924399	-0.508564422	0.541101948	-0.483616325	0.10807189
-0.563659125	-0.553394078	0.322005242	-0.622671297	0.622594318	-0.446416572
-0.488842264	-0.52994016	0.336724023	-0.610924337	0.651875056	-0.311489253
-0.469837213	-0.607284135	0.546476723	-0.528843256	0.403740306	0.016786546
-0.447423171	-0.5041312	0.403439149	-0.572389007	0.637161678	-0.466559264
0.515639777	0.533088691	-0.432696553	0.633915192	-0.524346674	0.573237692
0.58539749	0.493077368	-0.34648649	0.59971707	-0.545456408	1
0.457040548	0.576582128	-0.467704342	0.60738066	-0.471561486	0.589732506
-0.409895045	-0.53488046	0.249242676	-0.571055738	0.496256459	-0.45598816
-0.491147964	-0.587913896	0.450182928	-0.568286358	0.360535693	-0.519453483
-0.459816724	-0.407024219	0.260386719	-0.573600699	0.765239677	-0.463950052
-0.412574593	-0.54232132	0.437514163	-0.48975527	0.390133165	-0.188732276
-0.499560566	-0.433251567	0.394405441	-0.544529911	0.6315131	-0.37750087
-0.420364043	-0.393825198	0.482062728	-0.437120864	0.482441131	-0.121573636
-0.344787793	-0.431846236	0.152675525	-0.501454668	0.507953374	-0.520059441
-0.466336357	-0.405329782	0.137750665	-0.556752898	0.504691833	-0.600388935

MAPK9	MAPK8	MAPK3	MAPK10	MAPK1	MAP2K1
0.877711058	0.841849726	-0.48975527	0.808566233	0.787054702	0.908625801
0.886592296	0.795745929	-0.538319706	0.838608054	0.742306262	0.943185559
0.873355212	0.806695156	-0.54232132	0.78582346	0.749135487	0.912384176
0.940092956	0.72894455	-0.609741011	0.876154772	0.63896411	1
0.929755455	0.681106312	-0.569335334	0.841310175	0.641209834	0.94203639
1	0.669374071	-0.671587257	0.887100248	0.558737524	0.940092956
-0.779767601	-0.757561789	0.441166356	-0.697663702	-0.774504894	-0.816833531
0.865775984	0.788574328	-0.476900181	0.833933295	0.741731192	0.905321467
-0.80791976	-0.744608879	0.554270871	-0.725383552	-0.680130742	-0.830123122
0.855575279	0.729436637	-0.528875575	0.782217466	0.665266851	0.910376528
0.792023851	0.79498516	-0.472114585	0.768813058	0.756179535	0.854734904
0.954636302	0.600845606	-0.653374843	0.85930795	0.506711645	0.907665108
0.844243146	0.628265416	-0.590285256	0.844726246	0.573134597	0.890614336
0.79142886	0.651110677	-0.546128579	0.695901707	0.592321323	0.812067163
-0.858277378	-0.655821843	0.636612894	-0.756107595	-0.534816443	-0.848265842
0.887100248	0.602774749	-0.581655983	1	0.511856214	0.876154772
0.72040973	0.739799585	-0.436117409	0.616336168	0.7202537	0.750344688
0.733287652	0.673307032	-0.412574593	0.651246008	0.680410942	0.748148812
0.849228528	0.602126368	-0.616811318	0.7345614	0.50877803	0.801373186
0.669374071	1	-0.279742184	0.602774749	0.850239276	0.72894455
0.651027697	0.791015328	-0.386372394	0.588118119	0.756808462	0.749388607
-0.711727794	-0.632448814	0.390133165	-0.720690936	-0.623804392	-0.714174104
0.772867363	0.704444247	-0.47851706	0.660351755	0.608731098	0.74949387
0.761361657	0.704708587	-0.479997022	0.682772931	0.620500623	0.82495208
-0.669947589	-0.582699562	0.42136265	-0.65034083	-0.586220402	-0.700558107
0.574687073	0.770179018	-0.274873402	0.4683897	0.764389237	0.642970312
-0.68093095	-0.710842293	0.468185447	-0.614049622	-0.650297286	-0.697969235
0.558737524	0.850239276	-0.068891384	0.511856214	1	0.63896411
-0.668545956	-0.686018773	0.437514163	-0.566950729	-0.647338399	-0.710247185
0.640574638	0.652543205	-0.373151321	0.526042146	0.651745193	0.658999058
-0.693410406	-0.626062284	0.369504117	-0.661823528	-0.630588223	-0.685851928
-0.692922767	-0.524441105	0.478946124	-0.71998089	-0.444635354	-0.692414275
0.5967923	0.742154405	-0.270193108	0.475893975	0.723556383	0.632466916
-0.653816413	-0.648598079	0.305555368	-0.643712347	-0.623132176	-0.666561807
-0.631459721	-0.544720802	0.412650096	-0.518685359	-0.589583123	-0.653031565
0.684502908	0.499454212	-0.355434552	0.773192974	0.503226879	0.697969161
0.732305379	0.590998944	-0.627143405	0.619684693	0.496166373	0.757984439

-0.502877904	-0.633111847	0.199768119	-0.418294577	-0.77473426	-0.554559896
-0.599179152	-0.619350723	0.384797909	-0.438689009	-0.605328723	-0.616135164
-0.56368769	-0.777053467	0.319212803	-0.407247249	-0.714212087	-0.612322566
-0.547226527	-0.598386052	0.306455184	-0.556621262	-0.669786898	-0.582447599
-0.761451352	-0.451981658	0.572976177	-0.666891022	-0.324422633	-0.778841453
-0.547423068	-0.57134542	0.30873168	-0.529396005	-0.597796825	-0.536297668
0.820380841	0.362439194	-0.598473958	0.818770534	0.244788112	0.76336469
-0.636046803	-0.307458168	0.674961042	-0.629354601	-0.248477641	-0.656441368
0.741595908	0.293116011	-0.615841477	0.721659661	0.17060265	0.721666078
0.75192654	0.275152906	-0.554960421	0.694560386	0.226506382	0.727004871
-0.627676362	-0.490932776	0.226315043	-0.67100305	-0.473499812	-0.570371891
-0.565640761	-0.420085147	0.451588965	-0.468109469	-0.368926827	-0.597678043
-0.673085836	-0.363320912	0.640742337	-0.671764123	-0.197253861	-0.731338865
-0.444705369	-0.574048649	0.142647484	-0.438653094	-0.611048023	-0.466345712
0.34032463	0.727732034	0.157174022	0.342706433	0.833475189	0.41893035
0.486374325	0.528693939	-0.188732276	0.380521762	0.592026739	0.417558221
0.359636561	0.710732033	-0.07089842	0.260086515	0.77272764	0.399938205
-0.479330472	-0.48592967	0.246038875	-0.46939492	-0.527472967	-0.504089019
-0.416606274	-0.603914182	0.094782576	-0.396362589	-0.645005569	-0.494395552
-0.509499074	-0.343282858	0.32045941	-0.482758782	-0.403722825	-0.45740358
-0.671587257	-0.279742184	1	-0.581655983	-0.068891384	-0.609741011
-0.40654851	-0.517644545	0.202919216	-0.439653012	-0.544093109	-0.402181813
-0.584059458	-0.216056544	0.651987407	-0.553350641	-0.127195195	-0.523551134
-0.306556644	-0.537673033	0.079884229	-0.340599481	-0.574957402	-0.314453308
-0.399040101	-0.519007181	0.139301106	-0.252891099	-0.510448372	-0.360583536

KSR2	KRAS	KITLG	KIT	IGF1	GRIN2A
-0.528843256	0.645630136	0.758829917	0.731200789	0.688718663	0.723509764
-0.655655775	0.679045504	0.812641854	0.692802027	0.684401376	0.72894981
-0.607284135	0.61503239	0.822625856	0.71246464	0.715086997	0.752794338
-0.731338865	0.697969161	0.812067163	0.632466916	0.658999058	0.642970312
-0.677583226	0.696805132	0.822392184	0.628246672	0.666361959	0.612030824
-0.673085836	0.684502908	0.79142886	0.5967923	0.640574638	0.574687073
0.442086631	-0.665623174	-0.723604666	-0.72482854	-0.676277585	-0.750251382
-0.609342349	0.666836526	0.749742655	0.706659795	0.681700389	0.650057684
0.560968785	-0.581984029	-0.799278081	-0.675699789	-0.748919504	-0.72055267
-0.736552294	0.636406226	0.848514225	0.606483492	0.684512891	0.706418456
-0.544308256	0.652711093	0.659484297	0.668519786	0.580216855	0.678303262
-0.683485101	0.64261335	0.811693066	0.552840375	0.635314778	0.531007
-0.778769555	0.718197073	0.757330933	0.535462092	0.606282416	0.565340355
-0.59559326	0.542768248	1	0.655958278	0.794405844	0.756377371
0.615445815	-0.493148842	-0.839873886	-0.625570675	-0.738843452	-0.64510823
-0.671764123	0.773192974	0.695901707	0.475893975	0.526042146	0.4683897
-0.437482792	0.538843406	0.699587952	0.64828244	0.64790607	0.693951069
-0.469837213	0.524639313	0.776199434	0.731464282	0.759708493	0.63859806
-0.590466448	0.54446556	0.741054491	0.560331543	0.61918547	0.514275414
-0.363320912	0.499454212	0.651110677	0.742154405	0.652543205	0.770179018
-0.465206595	0.495825727	0.708400679	0.656112776	0.722824674	0.834414156
0.403740306	-0.636744938	-0.593259506	-0.532601566	-0.558289584	-0.582319276
-0.423669982	0.424301621	0.712872398	0.660717044	0.640832091	0.570761945
-0.656858135	0.513961123	0.738820931	0.557069566	0.63722109	0.649128716
0.548730029	-0.683946085	-0.621927516	-0.483385269	-0.555016869	-0.627556333
-0.334416848	0.440511582	0.756377371	0.70582632	0.805382445	1
0.406116959	-0.492809062	-0.601216613	-0.603597911	-0.523581598	-0.644799217
-0.197253861	0.503226879	0.592321323	0.723556383	0.651745193	0.764389237
0.546476723	-0.40451472	-0.776897105	-0.649836781	-0.734549872	-0.719472731
-0.400148247	0.434570871	0.794405844	0.765480236	1	0.805382445
0.489381914	-0.610364144	-0.676272584	-0.560393351	-0.625327329	-0.627213355
0.540077251	-0.627364073	-0.711004063	-0.433721229	-0.623829095	-0.581362029
-0.287064912	0.409574775	0.655958278	1	0.765480236	0.70582632
0.398529215	-0.543306145	-0.7211674	-0.551214266	-0.589886366	-0.612906044
0.381783249	-0.546881527	-0.526191531	-0.549316811	-0.515321535	-0.538725043
-0.535359959	1	0.542768248	0.409574775	0.434570871	0.440511582
-0.620702367	0.412892331	0.671993886	0.438969954	0.503698377	0.543981038

0.239732917	-0.574975322	-0.517996811	-0.603676852	-0.543394356	-0.669339062
0.392396049	-0.399599012	-0.710497604	-0.592239759	-0.732141826	-0.701541179
0.274582079	-0.328894392	-0.554415335	-0.666625572	-0.554759631	-0.677448338
0.223179076	-0.487190712	-0.475898808	-0.505746821	-0.456896296	-0.596373109
0.588391453	-0.543571524	-0.648038868	-0.42554866	-0.448976665	-0.472586607
0.177083728	-0.601767525	-0.411724468	-0.477982364	-0.417748972	-0.516101003
-0.704451524	0.607592924	0.67958737	0.302750575	0.475496835	0.308414353
0.729781718	-0.498997513	-0.58474619	-0.319878431	-0.404013444	-0.314871653
-0.789131026	0.569297709	0.641119657	0.237628891	0.419474703	0.2658506
-0.722470675	0.562965009	0.602657723	0.256363279	0.396566162	0.221359232
0.296078637	-0.537392082	-0.502883431	-0.445278801	-0.470166204	-0.382004488
0.402747718	-0.426061597	-0.510073566	-0.440615616	-0.414028092	-0.452427616
1	-0.535359959	-0.59559326	-0.287064912	-0.400148247	-0.334416848
0.123113548	-0.438817572	-0.409795885	-0.48602798	-0.506717201	-0.570683874
0.018135237	0.42837311	0.385147235	0.600627133	0.532044312	0.682270758
0.016786546	0.353846984	0.545288347	0.560055186	0.565875168	0.606263644
-0.00721499	0.227312572	0.365006237	0.618065095	0.457403622	0.692430869
0.195574732	-0.601307962	-0.398804178	-0.440628429	-0.421923657	-0.476272506
0.189132837	-0.417614713	-0.575382153	-0.534088125	-0.535952083	-0.62672611
0.208205285	-0.575310897	-0.371733371	-0.376842267	-0.366943766	-0.348947116
0.640742337	-0.355434552	-0.546128579	-0.270193108	-0.373151321	-0.274873402
0.129980109	-0.362677603	-0.334830861	-0.455474453	-0.389750993	-0.397207317
0.551041193	-0.49153468	-0.473086744	-0.238968907	-0.363600342	-0.205847514
-0.012355796	-0.39119624	-0.272014026	-0.409223253	-0.244139706	-0.465773728
-0.041770781	-0.251508554	-0.227564926	-0.493698658	-0.33177086	-0.378686912

GNG7	GNG3	GNG2	GNG10	GNB5	GAB2
-0.634444121	0.633915192	0.880766512	0.541101948	0.788855834	-0.684245106
-0.590983987	0.535142364	0.905618812	0.628643654	0.733356234	-0.735299932
-0.65032837	0.533088691	0.890852249	0.566924399	0.744021576	-0.732408749
-0.616135164	0.41893035	0.94203639	0.727004871	0.74949387	-0.666561807
-0.619765455	0.398642843	1	0.788665251	0.696463914	-0.67748447
-0.599179152	0.34032463	0.929755455	0.75192654	0.772867363	-0.653816413
0.675587452	-0.659603266	-0.820315943	-0.479075796	-0.654867031	0.587522052
-0.569495301	0.54366253	0.874987838	0.594299413	0.724989558	-0.649134458
0.756328809	-0.491451276	-0.832917592	-0.599577274	-0.773349301	0.766228402
-0.688572495	0.429904146	0.88687596	0.684781792	0.689107801	-0.697136356
-0.457379363	0.600510848	0.807831257	0.489325971	0.65588209	-0.63470621
-0.60579246	0.274578251	0.925049281	0.799729966	0.729756524	-0.636730721
-0.55307538	0.340230175	0.854560151	0.728046471	0.595435049	-0.613980271
-0.710497604	0.385147235	0.822392184	0.602657723	0.712872398	-0.7211674
0.715565062	-0.340721469	-0.820494142	-0.594116137	-0.78923263	0.618690592
-0.438689009	0.342706433	0.841310175	0.694560386	0.660351755	-0.643712347
-0.624512667	0.585794441	0.717571691	0.422498276	0.678062386	-0.621006599
-0.674403229	0.515639777	0.764555682	0.516090796	0.722343783	-0.649300621
-0.671185124	0.293911025	0.856578834	0.735857952	0.675225966	-0.554257805
-0.619350723	0.727732034	0.681106312	0.275152906	0.704444247	-0.648598079
-0.691438061	0.615705809	0.692524326	0.325991422	0.572617853	-0.558832305
0.429014343	-0.524346674	-0.734458017	-0.483616325	-0.607717637	0.504757868
-0.559548893	0.410806708	0.696463914	0.489155087	1	-0.649000823
-0.622229911	0.406686695	0.752269608	0.552644783	0.636295873	-0.624581669
0.548286005	-0.433003676	-0.721428548	-0.555253839	-0.470601522	0.608591131
-0.701541179	0.682270758	0.612030824	0.221359232	0.570761945	-0.612906044
0.516899806	-0.520882888	-0.616427944	-0.265080373	-0.668420199	0.510823417
-0.605328723	0.833475189	0.641209834	0.226506382	0.608731098	-0.623132176
0.807773026	-0.432696553	-0.702866509	-0.508564422	-0.655907099	0.586896009
-0.732141826	0.532044312	0.666361959	0.396566162	0.640832091	-0.589886366
0.562125902	-0.465762359	-0.733383624	-0.495657583	-0.56429191	0.531648002
0.519094566	-0.314043077	-0.692984262	-0.568918422	-0.618469269	0.603398939
-0.592239759	0.600627133	0.628246672	0.256363279	0.660717044	-0.551214266
0.571011417	-0.474512906	-0.67748447	-0.457982629	-0.649000823	1
0.523887075	-0.425117139	-0.689141422	-0.514722238	-0.496182971	0.417370129
-0.399599012	0.42837311	0.696805132	0.562965009	0.424301621	-0.543306145
-0.536960853	0.23138453	0.698102839	0.479022335	0.566895561	-0.456814422

0.559738237	-0.656797992	-0.588469488	-0.291832579	-0.45819668	0.484388422
1	-0.47540305	-0.619765455	-0.393387378	-0.559548893	0.571011417
0.591600826	-0.534248177	-0.558148283	-0.19787369	-0.674416688	0.568225588
0.433372997	-0.563067476	-0.565567739	-0.196138695	-0.465654646	0.419741433
0.440122059	-0.200047501	-0.71799696	-0.569811049	-0.542509772	0.52451617
0.314510082	-0.557300175	-0.553817829	-0.224999564	-0.440621385	0.412151521
-0.421785976	0.060867311	0.772217812	0.847566029	0.575589362	-0.543296364
0.382458176	0.002392148	-0.658655438	-0.677292716	-0.464373069	0.398356056
-0.439868641	-0.051591708	0.760292111	0.901425332	0.450312516	-0.475842474
-0.393387378	0.006470032	0.788665251	1	0.489155087	-0.457982629
0.249614534	-0.416935192	-0.613696377	-0.433933319	-0.561496079	0.597519026
0.377836773	-0.216076664	-0.613640582	-0.47793223	-0.481812472	0.479627946
0.392396049	0.018135237	-0.677583226	-0.722470675	-0.423669982	0.398529215
0.403308874	-0.556457917	-0.461271804	-0.173293772	-0.436649309	0.37432692
-0.47540305	1	0.398642843	0.006470032	0.410806708	-0.474512906
-0.456102552	0.573237692	0.45093873	0.10807189	0.554651948	-0.501984943
-0.428567783	0.728481907	0.361719229	-0.040206457	0.544151222	-0.510089031
0.315853438	-0.534262687	-0.471549549	-0.220149845	-0.339192584	0.312110141
0.482076424	-0.547706074	-0.439294443	-0.103884361	-0.543164397	0.76969196
0.302313408	-0.381506069	-0.574566248	-0.439813711	-0.331264017	0.289678858
0.384797909	0.157174022	-0.569335334	-0.554960421	-0.47851706	0.305555368
0.348582204	-0.496749022	-0.416741926	-0.148169268	-0.467167375	0.290099448
0.400500922	-0.009379468	-0.520205987	-0.598653609	-0.406234283	0.205680495
0.191378815	-0.579744765	-0.289627818	0.066967286	-0.360244145	0.301436764
0.244893609	-0.498233437	-0.35981869	0.022729539	-0.410003799	0.139205453

FOXO4	FGFR3	FGF9	FGF2	FGF1	ETS1
-0.866757548	-0.682070089	0.60738066	-0.556752898	-0.568286358	-0.730342125
-0.824224998	-0.718034286	0.549061655	-0.367740128	-0.577907549	-0.647829332
-0.834018418	-0.69119411	0.576582128	-0.405329782	-0.587913896	-0.630813482
-0.830123122	-0.778841453	0.399938205	-0.360583536	-0.494395552	-0.582447599
-0.832917592	-0.71799696	0.361719229	-0.35981869	-0.439294443	-0.565567739
-0.80791976	-0.761451352	0.359636561	-0.399040101	-0.416606274	-0.547226527
0.806352345	0.628309413	-0.585704974	0.516735454	0.481001345	0.755269481
-0.798390458	-0.632163152	0.501749982	-0.412216889	-0.498519287	-0.617019507
1	0.577771257	-0.530367999	0.316544798	0.566433189	0.573173512
-0.796487373	-0.670335614	0.429070775	-0.240824786	-0.540994005	-0.522774494
-0.759208666	-0.609957441	0.647438637	-0.449983294	-0.516266141	-0.688571164
-0.766037618	-0.747843512	0.263164392	-0.319319822	-0.365112073	-0.47556737
-0.730757846	-0.62802477	0.317292599	-0.154710871	-0.419020238	-0.48034021
-0.799278081	-0.648038868	0.365006237	-0.227564926	-0.575382153	-0.475898808
0.830879396	0.684363462	-0.317717051	0.285120275	0.442910321	0.501927053
-0.725383552	-0.666891022	0.260086515	-0.252891099	-0.396362589	-0.556621262
-0.75224171	-0.533303112	0.568126754	-0.446372218	-0.569903414	-0.562490766
-0.788293061	-0.516275669	0.457040548	-0.466336357	-0.491147964	-0.547403854
-0.782891805	-0.562480362	0.337081729	-0.335080393	-0.261760672	-0.48048181
-0.744608879	-0.451981658	0.710732033	-0.519007181	-0.603914182	-0.598386052
-0.733530967	-0.52982231	0.564270711	-0.354201356	-0.549982354	-0.608908536
0.744330661	0.445263635	-0.471561486	0.504691833	0.360535693	0.708502531
-0.773349301	-0.542509772	0.544151222	-0.410003799	-0.543164397	-0.465654646
-0.678321967	-0.60258196	0.404969571	-0.219288071	-0.501442748	-0.418777299
0.665176894	0.48375836	-0.408788988	0.232869667	0.399931318	0.561824066
-0.72055267	-0.472586607	0.692430869	-0.378686912	-0.62672611	-0.596373109
0.681768587	0.487114602	-0.616763411	0.471456107	0.447059873	0.602011011
-0.680130742	-0.324422633	0.77272764	-0.510448372	-0.645005569	-0.669786898
0.76265632	0.415993768	-0.467704342	0.137750665	0.450182928	0.431847479
-0.748919504	-0.448976665	0.457403622	-0.33177086	-0.535952083	-0.456896296
0.597487016	0.417621794	-0.383651794	0.380686334	0.395349599	0.465581146
0.712411309	0.552568615	-0.207572151	0.262414532	0.438637426	0.436252652
-0.675699789	-0.42554866	0.618065095	-0.493698658	-0.534088125	-0.505746821
0.766228402	0.52451617	-0.510089031	0.139205453	0.76969196	0.419741433
0.688498811	0.450494729	-0.493115266	0.438577008	0.317500986	0.570717556
-0.581984029	-0.543571524	0.227312572	-0.251508554	-0.417614713	-0.487190712
-0.579221739	-0.678712572	0.320118786	-0.314660133	-0.35099882	-0.495866767

0.618462058	0.332840543	-0.671814079	0.42061941	0.52110981	0.636791931
0.756328809	0.440122059	-0.428567783	0.244893609	0.482076424	0.433372997
0.680784551	0.408242805	-0.723282185	0.475037527	0.558356209	0.578212049
0.573173512	0.435863004	-0.518279978	0.560311663	0.413874096	1
0.577771257	1	-0.181632724	0.295085271	0.433187933	0.435863004
0.539766984	0.400477483	-0.500653571	0.623273272	0.376989349	0.685250299
-0.60614302	-0.654930282	-0.014942779	-0.036971339	-0.238635503	-0.231348131
0.628601649	0.460931642	-0.131161364	-0.006284802	0.15074782	0.361358077
-0.602079014	-0.554216909	-0.090518829	0.130121564	-0.11258664	-0.143221903
-0.599577274	-0.569811049	-0.040206457	0.022729539	-0.103884361	-0.196138695
0.547344553	0.409606704	-0.250390777	0.411879978	0.367696175	0.426907473
0.669228657	0.504240008	-0.369960288	0.296031231	0.331884264	0.450498514
0.560968785	0.588391453	-0.00721499	-0.041770781	0.189132837	0.223179076
0.50073351	0.192613313	-0.530394208	0.430193822	0.30063812	0.563728447
-0.491451276	-0.200047501	0.728481907	-0.498233437	-0.547706074	-0.563067476
-0.484358194	-0.363524781	0.589732506	-0.600388935	-0.519453483	-0.507926153
-0.530367999	-0.181632724	1	-0.490030418	-0.551260752	-0.518279978
0.448673855	0.409773178	-0.390599036	0.411365927	0.430168431	0.501369382
0.566433189	0.433187933	-0.551260752	0.242557869	1	0.413874096
0.548582276	0.293460283	-0.310968249	0.413181517	0.114500517	0.491932811
0.554270871	0.572976177	-0.07089842	0.139301106	0.094782576	0.306455184
0.516573156	0.07264513	-0.473315761	0.446165091	0.207558903	0.580811253
0.535063474	0.357022596	0.026054047	0.081104323	0.02616654	0.237542796
0.351219946	0.174003463	-0.611616467	0.492704428	0.307797596	0.573502435
0.316544798	0.295085271	-0.490030418	1	0.242557869	0.560311663

ELK1	EFNA1	CSF1R	CALML4	CALM3	CALM2
-0.437120864	-0.622671297	-0.75743694	-0.571055738	0.89070214	0.770225601
-0.425646353	-0.579396077	-0.724090342	-0.532770653	0.922507693	0.887315567
-0.393825198	-0.553394078	-0.744276739	-0.53488046	0.880245865	0.838266297
-0.523551134	-0.570371891	-0.697969235	-0.504089019	0.854734904	0.890614336
-0.520205987	-0.613696377	-0.616427944	-0.471549549	0.807831257	0.854560151
-0.584059458	-0.627676362	-0.68093095	-0.479330472	0.792023851	0.844243146
0.436935246	0.505093266	0.757846038	0.607558396	-0.833267668	-0.711597227
-0.410813512	-0.592020598	-0.706953859	-0.538629	0.885858342	0.83966946
0.535063474	0.547344553	0.681768587	0.448673855	-0.759208666	-0.730757846
-0.518323143	-0.478096766	-0.640099069	-0.448815217	0.779185158	0.877374789
-0.345795498	-0.530241088	-0.748316688	-0.585422462	1	0.810747087
-0.564489834	-0.604048813	-0.588992735	-0.41112986	0.70804254	0.82799205
-0.599900138	-0.473491986	-0.60227849	-0.464847493	0.810747087	1
-0.473086744	-0.502883431	-0.601216613	-0.398804178	0.659484297	0.757330933
0.558093056	0.541718501	0.663661236	0.396358197	-0.657989504	-0.73905009
-0.553350641	-0.67100305	-0.614049622	-0.46939492	0.768813058	0.844726246
-0.45367842	-0.466143562	-0.709446699	-0.521098819	0.755529772	0.689268937
-0.420364043	-0.563659125	-0.618383861	-0.409895045	0.664056509	0.644190371
-0.621373258	-0.477299232	-0.55957313	-0.346007054	0.646740199	0.752199295
-0.216056544	-0.490932776	-0.710842293	-0.48592967	0.79498516	0.628265416
-0.335093682	-0.376021495	-0.672285372	-0.535641401	0.751465099	0.698748439
0.482441131	0.622594318	0.621059976	0.496256459	-0.75139852	-0.624500976
-0.406234283	-0.561496079	-0.668420199	-0.339192584	0.65588209	0.595435049
-0.401797022	-0.388918495	-0.594491304	-0.357764714	0.705114538	0.816307374
0.42925533	0.50368632	0.598973372	0.439653911	-0.692514997	-0.721022627
-0.205847514	-0.382004488	-0.644799217	-0.476272506	0.678303262	0.565340355
0.377015995	0.427354498	1	0.456470345	-0.748316688	-0.60227849
-0.127195195	-0.473499812	-0.650297286	-0.527472967	0.756179535	0.573134597
0.482062728	0.322005242	0.545297611	0.249242676	-0.593168285	-0.726668477
-0.363600342	-0.470166204	-0.523581598	-0.421923657	0.580216855	0.606282416
0.40824564	0.628153907	0.530701967	0.391362073	-0.611012722	-0.682415196
0.537892341	0.673920918	0.478461242	0.358919208	-0.52527258	-0.603519254
-0.238968907	-0.445278801	-0.603597911	-0.440628429	0.668519786	0.535462092
0.205680495	0.597519026	0.510823417	0.312110141	-0.63470621	-0.613980271
0.43278634	0.300802567	0.550073433	0.539900204	-0.686311367	-0.587264134
-0.49153468	-0.537392082	-0.492809062	-0.601307962	0.652711093	0.718197073
-0.371998182	-0.298468871	-0.617358111	-0.374891982	0.652617564	0.713725767

0.252675321	0.260227297	0.585705437	0.61124131	-0.698315162	-0.553426143
0.400500922	0.249614534	0.516899806	0.315853438	-0.457379363	-0.55307538
0.12252351	0.247239466	0.641252713	0.388143459	-0.703368162	-0.510036545
0.237542796	0.426907473	0.602011011	0.501369382	-0.688571164	-0.48034021
0.357022596	0.409606704	0.487114602	0.409773178	-0.609957441	-0.62802477
0.327583406	0.544844763	0.605913762	0.574147266	-0.682729518	-0.457715414
-0.605514452	-0.560597213	-0.388501427	-0.24591115	0.493859299	0.743898756
0.651277775	0.274558708	0.50147791	0.238133517	-0.548903425	-0.72054177
-0.657169798	-0.420067187	-0.314594996	-0.169246863	0.452796737	0.767272153
-0.598653609	-0.433933319	-0.265080373	-0.220149845	0.489325971	0.728046471
0.318999095	1	0.427354498	0.317212635	-0.530241088	-0.473491986
0.348545469	0.360442299	0.433625282	0.293862256	-0.617463382	-0.472545103
0.551041193	0.296078637	0.406116959	0.195574732	-0.544308256	-0.778769555
0.234402209	0.452558409	0.534058982	0.418398901	-0.543894337	-0.399596815
-0.009379468	-0.416935192	-0.520882888	-0.534262687	0.600510848	0.340230175
-0.121573636	-0.446416572	-0.526154739	-0.45598816	0.466199973	0.287825004
0.026054047	-0.250390777	-0.616763411	-0.390599036	0.647438637	0.317292599
0.334916399	0.317212635	0.456470345	1	-0.585422462	-0.464847493
0.02616654	0.367696175	0.447059873	0.430168431	-0.516266141	-0.419020238
0.486365271	0.448945817	0.385526605	0.463327209	-0.525730067	-0.395472818
0.651987407	0.226315043	0.468185447	0.246038875	-0.472114585	-0.590285256
0.347369756	0.474849936	0.549277116	0.271184337	-0.504433307	-0.353316324
1	0.318999095	0.377015995	0.334916399	-0.345795498	-0.599900138
0.079264287	0.340932508	0.706561068	0.46541799	-0.537531562	-0.25310218
0.081104323	0.411879978	0.471456107	0.411365927	-0.449983294	-0.154710871

CALM1	BDNF	ANGPT2	AKT1
0.905379134	0.802681367	-0.544529911	-0.802816398
0.918176016	0.782022591	-0.430878761	-0.783871547
0.889708216	0.776960336	-0.433251567	-0.822345798
0.905321467	0.750344688	-0.402181813	-0.848265842
0.874987838	0.717571691	-0.416741926	-0.820494142
0.865775984	0.72040973	-0.40654851	-0.858277378
-0.818948558	-0.761597111	0.576522961	0.747658445
1	0.720276379	-0.487657528	-0.798740637
-0.798390458	-0.75224171	0.516573156	0.830879396
0.828687107	0.731967876	-0.356319111	-0.80770624
0.885858342	0.755529772	-0.504433307	-0.657989504
0.833916908	0.650855403	-0.337368426	-0.85420046
0.83966946	0.689268937	-0.353316324	-0.73905009
0.749742655	0.699587952	-0.334830861	-0.839873886
-0.798740637	-0.681097854	0.412960722	1
0.833933295	0.616336168	-0.439653012	-0.756107595
0.720276379	1	-0.451552311	-0.681097854
0.773513948	0.702682071	-0.499560566	-0.766517191
0.732463056	0.613458895	-0.451091724	-0.783023741
0.788574328	0.739799585	-0.517644545	-0.655821843
0.730802535	0.753402874	-0.419791433	-0.686438422
-0.721419266	-0.602543024	0.6315131	0.620857693
0.724989558	0.678062386	-0.467167375	-0.78923263
0.752665196	0.684435804	-0.290395627	-0.724857573
-0.660596132	-0.597485591	0.436515999	0.592210448
0.650057684	0.693951069	-0.397207317	-0.64510823
-0.706953859	-0.709446699	0.549277116	0.663661236
0.741731192	0.7202537	-0.544093109	-0.534816443
-0.670122207	-0.662294047	0.394405441	0.755050802
0.681700389	0.64790607	-0.389750993	-0.738843452
-0.685454714	-0.586016449	0.503951171	0.637951947
-0.629796522	-0.562117544	0.403741302	0.709489477
0.706659795	0.64828244	-0.455474453	-0.625570675
-0.649134458	-0.621006599	0.290099448	0.618690592
-0.646397629	-0.604733252	0.393657101	0.510408197
0.666836526	0.538843406	-0.362677603	-0.493148842
0.687469945	0.611927998	-0.214568879	-0.702526732

-0.614422468	-0.647156951	0.473744004	0.424277852
-0.569495301	-0.624512667	0.348582204	0.715565062
-0.650732884	-0.670095256	0.407808201	0.570498577
-0.617019507	-0.562490766	0.580811253	0.501927053
-0.632163152	-0.533303112	0.07264513	0.684363462
-0.569922828	-0.590751666	0.564424312	0.433717153
0.647454551	0.462393816	-0.184577898	-0.70883458
-0.557489017	-0.461025135	0.344366936	0.603714866
0.565203848	0.405659569	-0.160013937	-0.648023694
0.594299413	0.422498276	-0.148169268	-0.594116137
-0.592020598	-0.466143562	0.474849936	0.541718501
-0.547945074	-0.441369933	0.327831788	0.470704722
-0.609342349	-0.437482792	0.129980109	0.615445815
-0.507142769	-0.495866254	0.594349275	0.402403387
0.54366253	0.585794441	-0.496749022	-0.340721469
0.480670189	0.545600364	-0.37750087	-0.472789939
0.501749982	0.568126754	-0.473315761	-0.317717051
-0.538629	-0.521098819	0.271184337	0.396358197
-0.498519287	-0.569903414	0.207558903	0.442910321
-0.482677438	-0.420377542	0.487776759	0.376527303
-0.476900181	-0.436117409	0.202919216	0.636612894
-0.487657528	-0.451552311	1	0.412960722
-0.410813512	-0.45367842	0.347369756	0.558093056
-0.406014919	-0.422237692	0.544579199	0.242829285
-0.412216889	-0.446372218	0.446165091	0.285120275

FoxO_genes

PCC	Gene.symb	TGFB3	STK11	STAT3	RAF1
25.74082826	MAP2K1	-0.723242824	-0.727991659	-0.334046417	-0.45740358
25.4721925	GABARAI	-0.724387263	-0.631408503	-0.394877773	-0.587783909
25.14773706	BRAF	-0.724306948	-0.755938858	-0.399936871	-0.381486364
25.03119314	MAPK9	-0.712430861	-0.666654656	-0.333274598	-0.509499074
24.56207475	HOMER1	-0.777378327	-0.612232846	-0.518136041	-0.42209983
24.41332866	FOXO4	0.860945344	0.697919598	0.346225913	0.548582276
23.98382273	PIK3CB	-0.691756704	-0.681112387	-0.294397866	-0.453905739
23.60644451	MAPK10	-0.651069534	-0.63876868	-0.263623169	-0.482758782
23.59720667	AKT1	0.696310575	0.672065632	0.330647512	0.376527303
23.44892193	BNIP3	-0.64955689	-0.772375423	-0.295479498	-0.436329187
23.26905688	PDPK1	-0.688518538	-0.662093835	-0.351630882	-0.354945036
22.59513017	PLK2	-0.693312446	-0.494391221	-0.547946902	-0.482949872
22.28375747	HOMER3	0.592627257	0.818825519	0.195867292	0.406753199
21.70301462	CCND1	0.610255582	0.604230613	0.296293703	0.305218618
21.68915949	MAPK8	-0.681128549	-0.487726462	-0.607567373	-0.343282858
21.59817513	TGFB3	1	0.571064894	0.336020279	0.39222197
21.50484023	NLK	-0.673496393	-0.446695745	-0.620516649	-0.398807192
21.02255816	MAPK13	-0.632969789	-0.47942327	-0.50286086	-0.264363153
20.97784832	BCL6	0.56126748	0.500274321	0.571703147	0.653767518
20.69134682	FOXO1	0.569078744	0.409690382	0.439349907	0.660864546
20.67168894	GABARAI	-0.546029716	-0.781640446	-0.028037219	-0.389399819
20.64837286	IGF1	-0.688715863	-0.54523765	-0.481146811	-0.366943766
20.42663468	KLF2	0.46737385	0.450101617	0.359703648	0.562207794
20.15437503	STK11	0.571064894	1	0.1733545	0.234945815
20.00069503	KRAS	-0.469320622	-0.558806076	-0.344693217	-0.575310897
19.92320206	MAPK1	-0.65087028	-0.393623525	-0.723871782	-0.403722825
19.58603814	MAPK14	0.522220536	0.736845826	-0.076013588	0.211619246
18.39535814	PIK3CD	0.485204466	0.696696222	0.003631867	0.40580499
18.31489235	CDK2	0.500559322	0.293621312	0.700967521	0.434424457
17.53017559	CDKN1A	0.482594174	0.352441405	0.595399095	0.327911041
17.39908994	FOXG1	-0.55637582	-0.526456969	-0.201967881	-0.206082891
17.16105966	CCND2	-0.590246278	-0.518290169	-0.121907153	-0.360660619
16.52741915	MAPK3	0.425317007	0.561428998	-0.068931192	0.32045941
15.04774211	RAF1	0.39222197	0.234945815	0.328698534	1
13.18872666	STAT3	0.336020279	0.1733545	1	0.328698534

PLK2	PIK3CD	PIK3CB	PDPK1	NLK	MAPK9
0.770209142	-0.656441368	0.907665108	0.847589673	0.682378961	0.940092956
0.807505305	-0.649436402	0.839347079	0.787480429	0.697471484	0.90895793
0.73797596	-0.586414829	0.882620817	0.795596061	0.738144451	0.884040511
0.694806314	-0.636046803	0.954636302	0.772828143	0.623322479	1
0.792369652	-0.514993562	0.803592635	0.801264395	0.848383391	0.81695767
-0.769951511	0.628601649	-0.766037618	-0.683118176	-0.749623933	-0.80791976
0.619270891	-0.633554942	1	0.765195166	0.565330777	0.954636302
0.612572687	-0.629354601	0.85930795	0.779539516	0.539878712	0.887100248
-0.660458455	0.603714866	-0.85420046	-0.686455258	-0.703363972	-0.858277378
0.618860915	-0.703765014	0.75275238	0.77320125	0.606102592	0.776542567
0.675595564	-0.635641905	0.765195166	1	0.623499027	0.772828143
1	-0.408706398	0.619270891	0.675595564	0.831171098	0.694806314
-0.616660611	0.764994298	-0.692799629	-0.790635086	-0.526512684	-0.722512718
-0.563430877	0.59619396	-0.739009528	-0.745781337	-0.598365448	-0.757461642
0.82286693	-0.307458168	0.600845606	0.664916251	0.79669062	0.669374071
-0.693312446	0.485204466	-0.691756704	-0.688518538	-0.673496393	-0.712430861
0.831171098	-0.323034862	0.565330777	0.623499027	1	0.623322479
0.823440073	-0.334014698	0.618639547	0.71866448	0.74612476	0.679454064
-0.691723346	0.472145743	-0.639117268	-0.50727373	-0.603390725	-0.68394852
-0.7669047	0.452179965	-0.602686734	-0.561382532	-0.672639625	-0.703596524
0.418682262	-0.771383424	0.834954264	0.700819127	0.310560318	0.807583358
0.69926643	-0.404013444	0.635314778	0.612669003	0.771202111	0.640574638
-0.626247927	0.499500622	-0.629369198	-0.624049952	-0.598908427	-0.703718035
-0.494391221	0.696696222	-0.681112387	-0.662093835	-0.446695745	-0.666654656
0.544540635	-0.498997513	0.64261335	0.671320464	0.494116136	0.684502908
0.799310698	-0.248477641	0.506711645	0.648310271	0.795471049	0.558737524
-0.505888526	0.740613637	-0.666533313	-0.699049586	-0.375141191	-0.677773735
-0.408706398	1	-0.633554942	-0.635641905	-0.323034862	-0.636046803
-0.676676813	0.293199802	-0.48503287	-0.477916443	-0.664456284	-0.54332114
-0.552049372	0.277154185	-0.506639151	-0.471293996	-0.575433438	-0.55508377
0.442565617	-0.442907433	0.592350468	0.633668569	0.544690777	0.582601212
0.478668817	-0.416117818	0.613145712	0.62889289	0.451373945	0.605973777
-0.348152203	0.674961042	-0.653374843	-0.428220307	-0.308540973	-0.671587257
-0.482949872	0.40580499	-0.453905739	-0.354945036	-0.398807192	-0.509499074
-0.547946902	0.003631867	-0.294397866	-0.351630882	-0.620516649	-0.333274598

MAPK8	MAPK3	MAPK14	MAPK13	MAPK10	MAPK1
0.72894455	-0.609741011	-0.75384384	0.75602701	0.876154772	0.63896411
0.764930439	-0.595532653	-0.704870937	0.733567758	0.85377652	0.700683557
0.766257458	-0.563487242	-0.687649911	0.723329583	0.813365664	0.664997651
0.669374071	-0.671587257	-0.677773735	0.679454064	0.887100248	0.558737524
0.760852228	-0.456749949	-0.54018808	0.765386943	0.725690714	0.744024007
-0.744608879	0.554270871	0.638113205	-0.657998537	-0.725383552	-0.680130742
0.600845606	-0.653374843	-0.666533313	0.618639547	0.85930795	0.506711645
0.602774749	-0.581655983	-0.689081801	0.602243715	1	0.511856214
-0.655821843	0.636612894	0.640747461	-0.595183778	-0.756107595	-0.534816443
0.606917394	-0.604361563	-0.681985278	0.602759979	0.762989585	0.557858709
0.664916251	-0.428220307	-0.699049586	0.71866448	0.779539516	0.648310271
0.82286693	-0.348152203	-0.505888526	0.823440073	0.612572687	0.799310698
-0.53959994	0.563195491	0.783645466	-0.585269995	-0.728676408	-0.46271032
-0.621197426	0.559497218	0.630275088	-0.582327896	-0.724309214	-0.513700448
1	-0.279742184	-0.441224381	0.787181591	0.602774749	0.850239276
-0.681128549	0.425317007	0.522220536	-0.632969789	-0.651069534	-0.65087028
0.79669062	-0.308540973	-0.375141191	0.74612476	0.539878712	0.795471049
0.787181591	-0.316629696	-0.539182451	1	0.602243715	0.7635539
-0.618604861	0.439074938	0.420535617	-0.506086244	-0.640488839	-0.598903533
-0.616786026	0.447184438	0.478509549	-0.657064808	-0.606246691	-0.596361479
0.377835372	-0.678832806	-0.784390702	0.407342897	0.82217277	0.241275225
0.652543205	-0.373151321	-0.429242706	0.560424626	0.526042146	0.651745193
-0.601885291	0.459249093	0.505161653	-0.595405864	-0.669223036	-0.581840618
-0.487726462	0.561428998	0.736845826	-0.47942327	-0.63876868	-0.393623525
0.499454212	-0.355434552	-0.553304481	0.526913856	0.773192974	0.503226879
0.850239276	-0.068891384	-0.30244961	0.7635539	0.511856214	1
-0.441224381	0.645030821	1	-0.539182451	-0.689081801	-0.30244961
-0.307458168	0.674961042	0.740613637	-0.334014698	-0.629354601	-0.248477641
-0.685097847	0.273137589	0.262293083	-0.574545319	-0.521002414	-0.696965757
-0.592937713	0.342290627	0.309445225	-0.47913059	-0.522765045	-0.54827442
0.435421326	-0.387557678	-0.465400424	0.432262649	0.574322074	0.327965599
0.476444415	-0.275133921	-0.487767184	0.472783777	0.65294816	0.452659744
-0.279742184	1	0.645030821	-0.316629696	-0.581655983	-0.068891384
-0.343282858	0.32045941	0.211619246	-0.264363153	-0.482758782	-0.403722825
-0.607567373	-0.068931192	-0.076013588	-0.50286086	-0.263623169	-0.723871782

MAP2K1	KRAS	KLF2	IGF1	HOMER3	HOMER1
1	0.697969161	-0.72649002	0.658999058	-0.787999138	0.837398532
0.927379744	0.705947367	-0.760211059	0.646971365	-0.737343523	0.794608221
0.906727111	0.646452581	-0.646912455	0.737253417	-0.753985921	0.853440938
0.940092956	0.684502908	-0.703718035	0.640574638	-0.722512718	0.81695767
0.837398532	0.601103366	-0.648307251	0.795097873	-0.66738914	1
-0.830123122	-0.581984029	0.640266498	-0.748919504	0.726904414	-0.819358633
0.907665108	0.64261335	-0.629369198	0.635314778	-0.692799629	0.803592635
0.876154772	0.773192974	-0.669223036	0.526042146	-0.728676408	0.725690714
-0.848265842	-0.493148842	0.598502597	-0.738843452	0.671863975	-0.821719025
0.808626333	0.641361002	-0.64516291	0.61049498	-0.828781352	0.767749973
0.847589673	0.671320464	-0.624049952	0.612669003	-0.790635086	0.801264395
0.770209142	0.544540635	-0.626247927	0.69926643	-0.616660611	0.792369652
-0.787999138	-0.686855637	0.588772455	-0.531087991	1	-0.66738914
-0.774077403	-0.580475293	0.638397695	-0.516093826	0.698131057	-0.714933144
0.72894455	0.499454212	-0.601885291	0.652543205	-0.53959994	0.760852228
-0.723242824	-0.469320622	0.46737385	-0.688715863	0.592627257	-0.777378327
0.682378961	0.494116136	-0.598908427	0.771202111	-0.526512684	0.848383391
0.75602701	0.526913856	-0.595405864	0.560424626	-0.585269995	0.765386943
-0.665080377	-0.55618145	0.543452696	-0.580496429	0.596477246	-0.613580438
-0.724670258	-0.611162222	0.692402089	-0.517551403	0.555127871	-0.670821734
0.797292284	0.645575711	-0.512054341	0.444766685	-0.795370339	0.582106518
0.658999058	0.434570871	-0.499703631	1	-0.531087991	0.795097873
-0.72649002	-0.651894842	1	-0.499703631	0.588772455	-0.648307251
-0.727991659	-0.558806076	0.450101617	-0.54523765	0.818825519	-0.612232846
0.697969161	1	-0.651894842	0.434570871	-0.686855637	0.601103366
0.63896411	0.503226879	-0.581840618	0.651745193	-0.46271032	0.744024007
-0.75384384	-0.553304481	0.505161653	-0.429242706	0.783645466	-0.54018808
-0.656441368	-0.498997513	0.499500622	-0.404013444	0.764994298	-0.514993562
-0.547099433	-0.383840121	0.524817321	-0.538087562	0.390829993	-0.623047635
-0.54116884	-0.378049867	0.411825186	-0.553803664	0.418651814	-0.566451707
0.609191676	0.460964577	-0.360593884	0.522098254	-0.570829033	0.666401603
0.641532951	0.547409316	-0.402921124	0.535299203	-0.48607066	0.618258753
-0.609741011	-0.355434552	0.459249093	-0.373151321	0.563195491	-0.456749949
-0.45740358	-0.575310897	0.562207794	-0.366943766	0.406753199	-0.42209983
-0.334046417	-0.344693217	0.359703648	-0.481146811	0.195867292	-0.518136041

GABARAPL2	GABARAPL1	FOXO4	FOXO1	FOXG1	CDKN1A
0.797292284	0.927379744	-0.830123122	-0.724670258	0.609191676	-0.54116884
0.703614582	1	-0.845508062	-0.81351895	0.49049113	-0.571271643
0.770972244	0.841104518	-0.840045862	-0.587084376	0.637384749	-0.535767072
0.807583358	0.90895793	-0.80791976	-0.703596524	0.582601212	-0.55508377
0.582106518	0.794608221	-0.819358633	-0.670821734	0.666401603	-0.566451707
-0.663918754	-0.845508062	1	0.683780386	-0.531663044	0.488791079
0.834954264	0.839347079	-0.766037618	-0.602686734	0.592350468	-0.506639151
0.82217277	0.85377652	-0.725383552	-0.606246691	0.574322074	-0.522765045
-0.71143757	-0.793144885	0.830879396	0.558987178	-0.602786302	0.538199888
0.757749953	0.774245306	-0.789182925	-0.561357657	0.565674704	-0.496330464
0.700819127	0.787480429	-0.683118176	-0.561382532	0.633668569	-0.471293996
0.418682262	0.807505305	-0.769951511	-0.7669047	0.442565617	-0.552049372
-0.795370339	-0.737343523	0.726904414	0.555127871	-0.570829033	0.418651814
-0.685669542	-0.72790638	0.687093568	0.47994505	-0.6130343	0.488021298
0.377835372	0.764930439	-0.744608879	-0.616786026	0.435421326	-0.592937713
-0.546029716	-0.724387263	0.860945344	0.569078744	-0.55637582	0.482594174
0.310560318	0.697471484	-0.749623933	-0.672639625	0.544690777	-0.575433438
0.407342897	0.733567758	-0.657998537	-0.657064808	0.432262649	-0.47913059
-0.522833153	-0.74218661	0.695401013	0.723618998	-0.388199436	0.768919463
-0.411133385	-0.81351895	0.683780386	1	-0.331359577	0.531473199
1	0.703614582	-0.663918754	-0.411133385	0.561482835	-0.360001551
0.444766685	0.646971365	-0.748919504	-0.517551403	0.522098254	-0.553803664
-0.512054341	-0.760211059	0.640266498	0.692402089	-0.360593884	0.411825186
-0.781640446	-0.631408503	0.697919598	0.409690382	-0.526456969	0.352441405
0.645575711	0.705947367	-0.581984029	-0.611162222	0.460964577	-0.378049867
0.241275225	0.700683557	-0.680130742	-0.596361479	0.327965599	-0.54827442
-0.784390702	-0.704870937	0.638113205	0.478509549	-0.465400424	0.309445225
-0.771383424	-0.649436402	0.628601649	0.452179965	-0.442907433	0.277154185
-0.277096012	-0.620655986	0.556662018	0.61775806	-0.293032783	0.76730716
-0.360001551	-0.571271643	0.488791079	0.531473199	-0.418373474	1
0.561482835	0.49049113	-0.531663044	-0.331359577	1	-0.418373474
0.567673758	0.594065227	-0.593414782	-0.379067778	0.422931194	-0.204924973
-0.678832806	-0.595532653	0.554270871	0.447184438	-0.387557678	0.342290627
-0.389399819	-0.587783909	0.548582276	0.660864546	-0.206082891	0.327911041
-0.028037219	-0.394877773	0.346225913	0.439349907	-0.201967881	0.595399095

CDK2	CCND2	CCND1	BRAF	BNIP3	BCL6
-0.547099433	0.641532951	-0.774077403	0.906727111	0.808626333	-0.665080377
-0.620655986	0.594065227	-0.72790638	0.841104518	0.774245306	-0.74218661
-0.553952411	0.643581246	-0.7823536	1	0.798685285	-0.648356568
-0.54332114	0.605973777	-0.757461642	0.884040511	0.776542567	-0.68394852
-0.623047635	0.618258753	-0.714933144	0.853440938	0.767749973	-0.613580438
0.556662018	-0.593414782	0.687093568	-0.840045862	-0.789182925	0.695401013
-0.48503287	0.613145712	-0.739009528	0.882620817	0.75275238	-0.639117268
-0.521002414	0.65294816	-0.724309214	0.813365664	0.762989585	-0.640488839
0.575499393	-0.534392431	0.742261487	-0.858127527	-0.783045441	0.634790014
-0.500569432	0.530788362	-0.775785387	0.798685285	1	-0.611492238
-0.477916443	0.62889289	-0.745781337	0.795596061	0.77320125	-0.50727373
-0.676676813	0.478668817	-0.563430877	0.73797596	0.618860915	-0.691723346
0.390829993	-0.48607066	0.698131057	-0.753985921	-0.828781352	0.596477246
0.505782598	-0.347830621	1	-0.7823536	-0.775785387	0.497673779
-0.685097847	0.476444415	-0.621197426	0.766257458	0.606917394	-0.618604861
0.500559322	-0.590246278	0.610255582	-0.724306948	-0.64955689	0.56126748
-0.664456284	0.451373945	-0.598365448	0.738144451	0.606102592	-0.603390725
-0.574545319	0.472783777	-0.582327896	0.723329583	0.602759979	-0.506086244
0.764278069	-0.31652451	0.497673779	-0.648356568	-0.611492238	1
0.61775806	-0.379067778	0.47994505	-0.587084376	-0.561357657	0.723618998
-0.277096012	0.567673758	-0.685669542	0.770972244	0.757749953	-0.522833153
-0.538087562	0.535299203	-0.516093826	0.737253417	0.61049498	-0.580496429
0.524817321	-0.402921124	0.638397695	-0.646912455	-0.64516291	0.543452696
0.293621312	-0.518290169	0.604230613	-0.755938858	-0.772375423	0.500274321
-0.383840121	0.547409316	-0.580475293	0.646452581	0.641361002	-0.55618145
-0.696965757	0.452659744	-0.513700448	0.664997651	0.557858709	-0.598903533
0.262293083	-0.487767184	0.630275088	-0.687649911	-0.681985278	0.420535617
0.293199802	-0.416117818	0.59619396	-0.586414829	-0.703765014	0.472145743
1	-0.191358393	0.505782598	-0.553952411	-0.500569432	0.764278069
0.76730716	-0.204924973	0.488021298	-0.535767072	-0.496330464	0.768919463
-0.293032783	0.422931194	-0.6130343	0.637384749	0.565674704	-0.388199436
-0.191358393	1	-0.347830621	0.643581246	0.530788362	-0.31652451
0.273137589	-0.275133921	0.559497218	-0.563487242	-0.604361563	0.439074938
0.434424457	-0.360660619	0.305218618	-0.381486364	-0.436329187	0.653767518
0.700967521	-0.121907153	0.296293703	-0.399936871	-0.295479498	0.571703147

AKT1
-0.848265842
-0.793144885
-0.858127527
-0.858277378
-0.821719025
0.830879396
-0.85420046
-0.756107595
1
-0.783045441
-0.686455258
-0.660458455
0.671863975
0.742261487
-0.655821843
0.696310575
-0.703363972
-0.595183778
0.634790014
0.558987178
-0.71143757
-0.738843452
0.598502597
0.672065632
-0.493148842
-0.534816443
0.640747461
0.603714866
0.575499393
0.538199888
-0.602786302
-0.534392431
0.636612894
0.376527303
0.330647512
