# Supplementary figures and images for: Down-Regulation of Insulin Like Growth Factor 1 Involved in Alzheimer's Disease via MAPK, Ras, and FoxO Signaling Pathways
Source: Oxid Med Cell Longev. 2022 May 4;2022:8169981. doi: 10.1155/2022/8169981 (PMC9096571; doi:10.1155/2022/8169981)

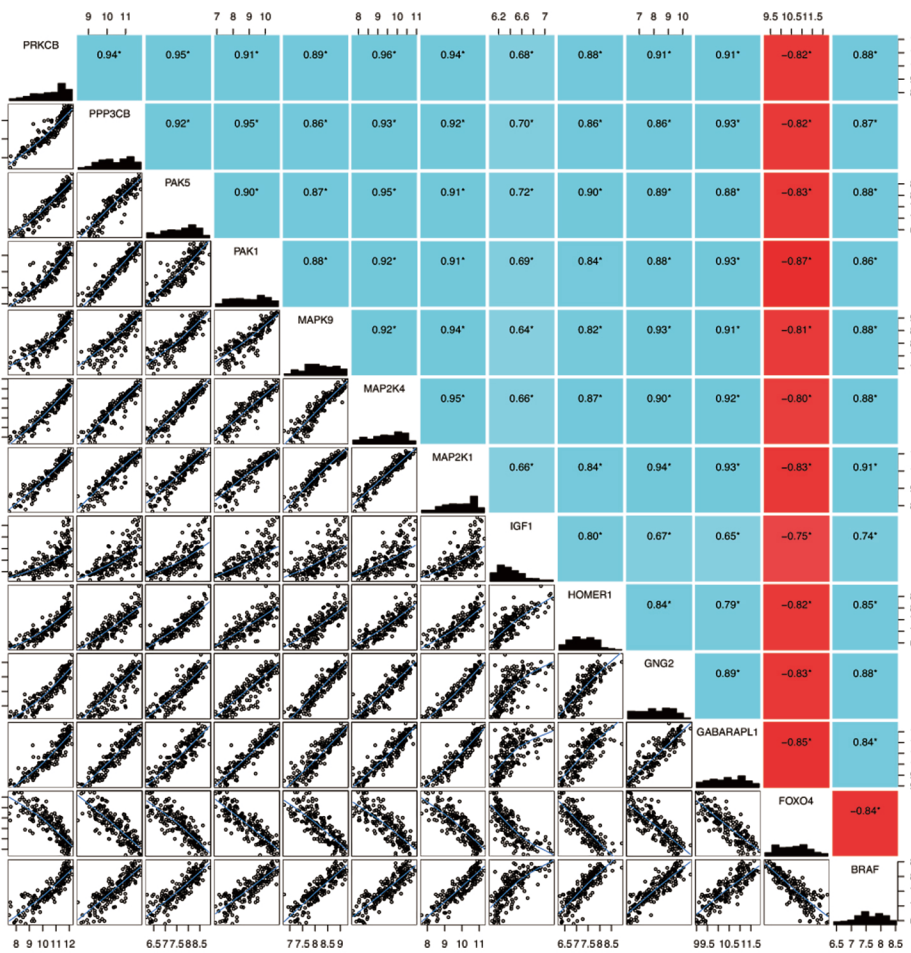

Supplement: Supplementary 4 — Supplementary Figure 1: Correlation between IGF1 and pathway signature genes. ∗p < 0.05. [file 8169981.f4.pdf]
